# Hyperglycemic conditions induce rapid cell dysfunction-promoting transcriptional alterations in human aortic endothelial cells

**DOI:** 10.1038/s41598-022-24999-5

**Published:** 2022-12-03

**Authors:** Odmaa Bayaraa, Claire K. Inman, Sneha A. Thomas, Fatima Al Jallaf, Manar Alshaikh, Youssef Idaghdour, Louise Ashall

**Affiliations:** 1 Public Health Research Center, New York University Abu Dhabi, Abu Dhabi, United Arab Emirates; 2 Program in Biology, Division of Science and Mathematics, New York University Abu Dhabi, Abu Dhabi, United Arab Emirates

**Keywords:** Transcriptomics, Diabetes complications

## Abstract

Hyperglycemia is a major risk factor in the development of diabetic complications and promotes vascular complications through dysregulation of endothelial cell function. Various mechanisms have been proposed for endothelial cell dysregulation but the early transcriptomic alterations of endothelial cells under hyperglycemic conditions are not well documented. Here we use deep time-series RNA-seq profiling of human aortic endothelial cells (HAECs) following exposure to normal (NG) and high glucose (HG) conditions over a time course from baseline to 24 h to identify the early and transient transcriptomic changes, alteration of molecular networks, and their temporal dynamics. The analysis revealed that the most significant pathway activation/inhibition events take place in the 1- to 4-h transition and identified distinct clusters of genes that underlie a cascade of coordinated transcriptional events unique to HG conditions. Temporal co-expression and causal network analysis implicate the activation of type 2 diabetes (T2D) and growth factor signalling pathways including STAT3 and NF-κB. These results document HAEC transcriptional changes induced by hyperglycemic conditions and provide basic insight into the rapid molecular alterations that promote endothelial cell dysfunction.

## Introduction

Type 2 diabetes (T2D) is a worldwide health problem with increasing numbers of prediabetic individuals^1^. The ultimate effect of uncontrolled hyperglycaemia is the development of chronic complications that affect the entire body. Specifically, elevated glucose levels are a major risk factor in the development of vascular complications in diabetic patients, including stroke, ischemic heart disease and peripheral vascular disease^2^. It is the complications of diabetes that reduce the quality of life and lead to mortality.

It is well-established that endothelial cell (EC) dysfunction is an early and pivotal triggering event in diabetes that primes the organism for the development of diabetic complications^3,4^. During hyperglycaemic conditions, endothelial cells (ECs) undergo multiple mechanistic changes. A reduction in EC proliferation and increase in apoptosis has been observed in response to high glucose exposure^5,6^. Furthermore, hyperglycaemia results in increased local inflammation and elevated intracellular reactive oxygen species (ROS)^7,8^. This in turn causes activation of major pathways associated with the development of diabetic complications, such as the polyol pathway, hexosamine pathway, protein kinase C (PKC), increased formation of advanced glycation end products (AGEs) and upregulation of the receptor for AGEs (RAGE)^9^. These changes result in disruption of normal vascular homeostasis and activation of proinflammatory pathways. The transcriptional changes that ECs undergo during hyperglycaemia ultimately lead to EC dysfunction.

The duration of hyperglycaemic exposure has both a transcriptional and epigenetic impact on cells. Increasing evidence suggests that equally long term and transient exposure of cells to high glucose conditions results in epigenetic changes that play a key role in vascular complications of diabetes and metabolic memory^10^. Previous transcriptional studies in ECs exposed to high glucose have focused on treatment durations of 24 h up to 6 days. Additionally, these analyses have been determined at a single time point^11–15^. We aimed to document the dynamics of gene expression by using a time-series RNA-sequencing (seq) analysis of human aortic endothelial cells (HAECs) with a focus on transcriptional changes up to 24 h post exposure to high and normal glucose levels. We tested the hypothesis that HAECs are transcriptionally responsive to differential glucose exposure and that high glucose treatment of HAECs causes a distinct early transcriptional response in comparison to normal glucose conditions. We aimed to identify groups of genes based on the duration of high glucose treatment and determine which signaling pathways are activated or inhibited, specific to each target group of genes.

## Results

### Glucose treatment causes significant transcriptional changes in HAECs

**Figure 1:**
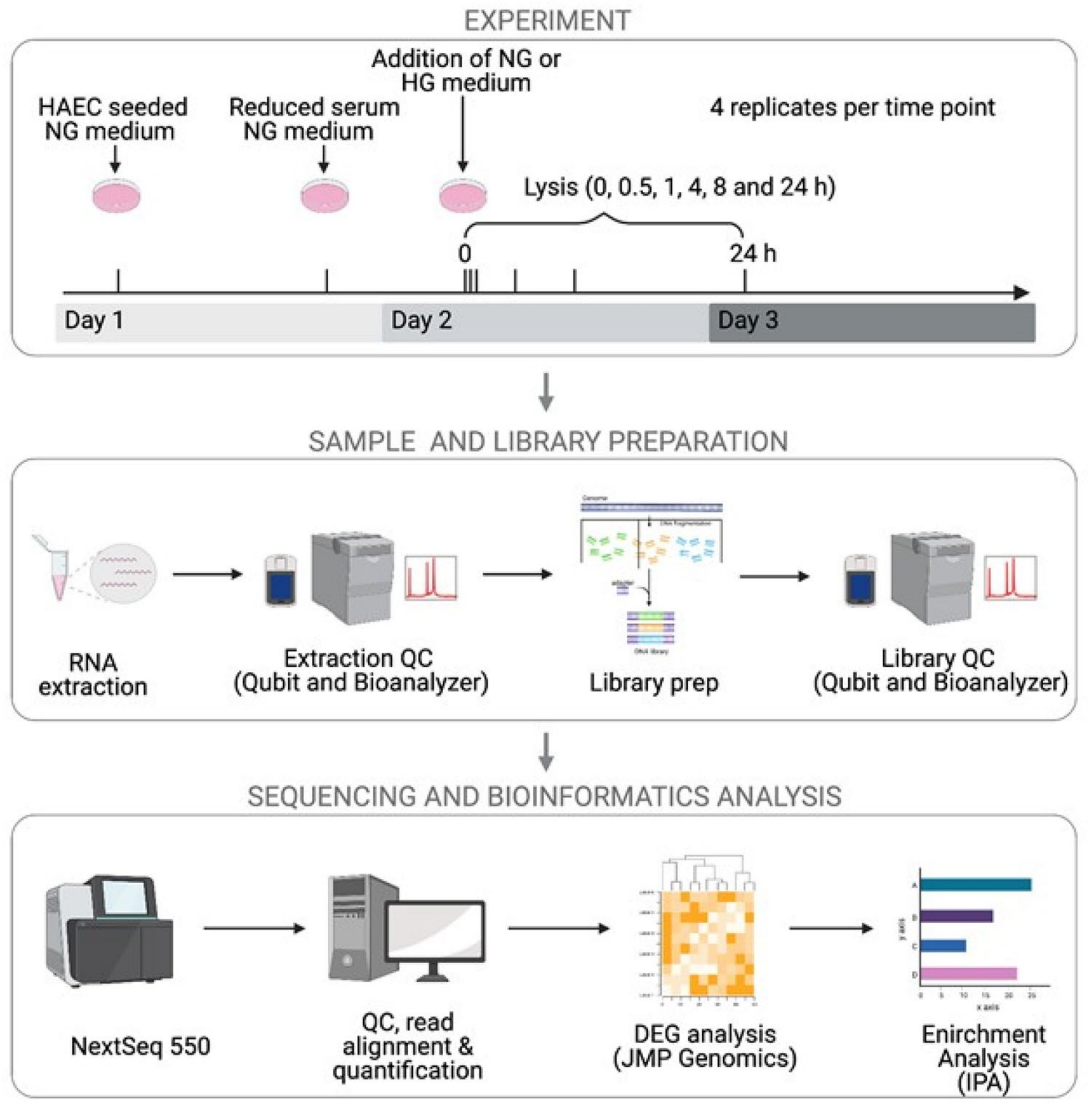
Schematic of time-series RNA-seq experimental design. Post overnight exposure to reduced serum medium, HAECs were treated with either NG (5.5 mM d-glucose) or HG (25 mM d-glucose) for 0.5, 1, 4, 8 and 24 h. Sample and library preparation was followed by NextSeq 550 sequencing and bioinformatics analysis. Figure created with BioRender.com.

**Figure 2:**
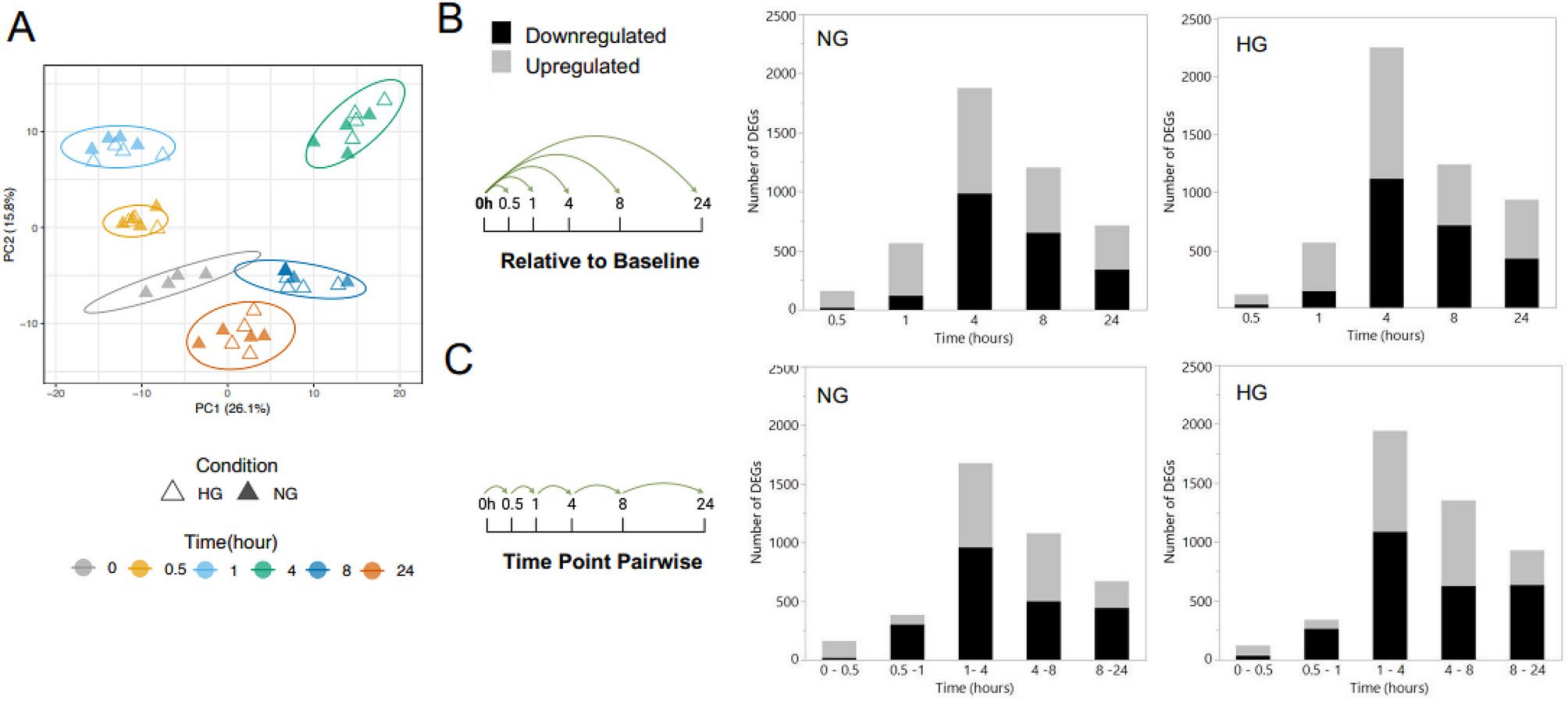
The temporal effect of glucose treatment on the HAEC transcriptome. (**A**) PCA of each time point in NG (5.5 mM d-glucose) or HG (25 mM d-glucose) conditions. Schematics show the pairwise comparisons of the baseline sample (NG 0 h) with each treatment time point (NG/HG 0.5, 1, 4, 8 or 24 h); (**B**) or between time points (0–0.5, 0.5–1, 1–4, 4–8 and 8–24 h); (**C**) that was applied for differential gene expression analysis evaluated by analysis of variance (ANOVA), using the Basic Expression workflow implemented in JMP Genomics 8 (SAS Institute). Graphs show the number of DEGs (± 1.2 fold change, adjusted p-value < 0.05 as statistical significance threshold) that are up (grey bars) or downregulated (black bars) in either NG or HG conditions compared to baseline (**B**) or the previous time point (**C**).

To investigate how the transcriptome profile of HAECs is altered upon treatment with either normal glucose (NG, 5.5 mM d-glucose) or diabetes-relevant high glucose (HG, 25 mM d-glucose), a time-series RNA-seq experiment was designed and performed as illustrated in Fig. 1. Following overnight exposure to reduced serum medium, HAECs were treated with either NG or HG for 0.5, 1, 4, 8 and 24 h. Four biological replicates were generated for each time point (with exception to 0.5 h HG, which has three replicates) to improve statistical power in detecting differentially expressed genes. Principle component analysis (PCA) showed distinct clusters based on the duration of glucose treatment (Fig. 2A) as opposed to concentration of glucose treatment (Supplemental Fig. 1). This analysis clearly indicates that major transcriptional changes take place as early as 0.5 h post exposure to glucose treatment and indicate that the magnitude of transcriptional changes is highest at the 4 h time point (Fig. 2A).

Initially, differential gene expression analysis using pairwise comparisons of the baseline sample (NG 0 h) with each treatment time point (NG/HG 0.5, 1, 4, 8 or 24 h) was applied. Under NG conditions, the number of differentially expressed genes (DEGs) increased with increasing treatment duration, with the peak number of DEGs observed at 4 h (1875, total number of genes, ± 1.2 fold change, adjusted p-value < 0.05 as statistical significance threshold). By 8 h NG conditions, the number of DEGs decreased (1203 genes) compared to 4 h and further reduced by 24 h (713 genes). At the early time points, 0.5 and 1 h NG conditions, upregulation of genes dominated, with 88 and 79% of genes upregulated respectively. At the subsequent time points, 4, 8 and 24 h the ratio of upregulated vs. downregulated genes was more evenly split (42–54%, Fig. 2B). Under HG conditions, a similar pattern was observed, however the number of DEGs was greater at all time points (except at 0.5 h HG) with a total of 2250 genes at the peak at 4 h HG (Fig. 2B).

For both NG and HG conditions, DEGs were also assessed using pairwise comparisons between time points (0–0.5, 0.5–1, 1–4, 4–8 and 8–24 h) to investigate the changes in the number of DEGs relative to the previous time point (Fig. 2C). In both NG and HG conditions, a similar pattern of expression was observed to that described previously for the analysis of each time point compared to baseline. However, this analysis highlighted that more genes expressed between 0.5 h and 1 h were downregulated (79 and 78% for NG and HG respectively) as opposed to upregulated, which was observed when analysing the 1 h time point to the baseline sample (NG 0 h). Also, when comparing the 8 h and 24 h time point, a greater number of downregulated genes (66 and 68% NG and HG respectively) was observed compared to 0 h and 24 h comparison analysis. Further differential expression analysis was focused on comparing time points to the baseline sample opposed to the previous time point as this highlighted more activation rather than inhibition of DEGs and pathways (see supplemental Figs. 2 and 3).

### HAECs transcriptional changes associated with differential glucose treatment

**Figure 3:**
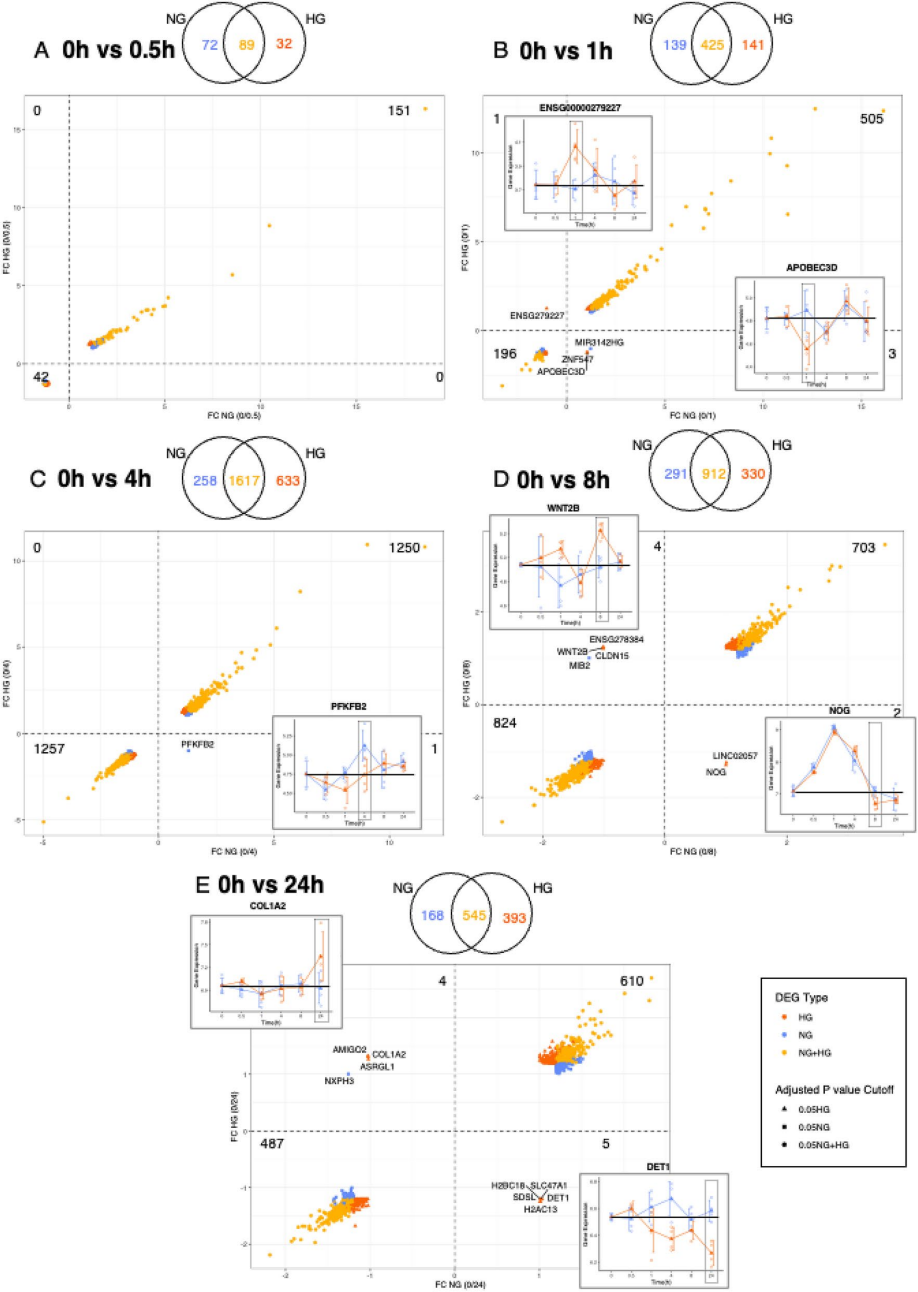
Differential glucose treatment results in transcriptional changes in HAECs. Venn analysis and scatterplots of fold change of DEGs (± 1.2 fold change, adjusted p-value < 0.05 for significance) with pairwise comparisons of each time point A) 0 h vs 0.5 h NG/HG, B) 0 h vs 1 h NG/HG, C) 0 h vs 4 h NG/HG, D) 0 h vs 8 h NG/HG, and E) 0 h vs 24 h NG/HG. Colour coding shows DEGs specific to HG (orange), NG (blue) or both NG and HG (yellow) conditions. Graph inserts plot fold change of individual genes that show opposite transcriptional responses in NG and HG conditions. Each replicate for each time point is plotted together with the mean average fold change, error bars show standard deviation. Red boxes highlight the baseline expression and where the difference in gene expression occurs between NG and HG conditions.

Venn analysis comparing each time point with the baseline (NG 0 h, Fig. 3) or the previous time point (Supplemental Fig. 2), showed both similar and unique gene expression patterns in NG and HG conditions. Scatter plots (and volcano plots, supplemental Fig. 4) of fold change were used to visualize and assess the level of consistency of transcriptional changes between NG and HG conditions (Fig. 3). The plots show the number of significant (± 1.2 fold change, adjusted p-value < 0.05) DEGs either up or downregulated, which are similar or unique to each treatment relative to baseline at each of the five time points. The transcriptional changes at NG and HG conditions at all time points were consistent, indicating that glucose treatment effects are systematic and that differences between NG and HG conditions are largely of  a quantitative nature. Next, we focused on genes that show opposite transcriptional responses in NG and HG conditions. Seven genes were identified as significantly upregulated in HG conditions yet showed a downregulated trend in NG conditions, six of those genes were observed at the later time points (*ENSG00000279227* (1 h)*, ENSG00000278384, WNT2B, CLDN15* (8 h)*, AMIGO2, COL1A2, ASRGL1* (24 h)). Additionally, nine genes were significantly downregulated in HG conditions but were significantly upregulated in NG conditions, again with more genes identified at the late time point (*ZNF547*, *APOBEC3D* (1 h), *LINC02057, NOG* (8 h), *H2BC18, SLC47A1, SDSL, DET1, H2AC13* (24 h). The reverse was also observed, with genes significantly upregulated in NG but a downregulated trend in HG conditions (*MIR3142HG* (1 h), *PFKFB2* (4 h)). Furthermore, two significantly downregulated genes in NG conditions yet with an upregulated trend under HG conditions were identified (*MIB2* (8 h), *NXPH3* (24 h)). We validated twelve DEGs identified by RNA-Seq using RT-qPCR analysis (supplemental Fig. 5 and data in supplemental Table 13). The genes were selected based on DE in HG compared to NG conditions and associated with signaling pathways that result in EC dysfunction, T2D and its complications.

### HG treatment causes differential pathway activation/inhibition compared to NG conditions

**Figure 4:**
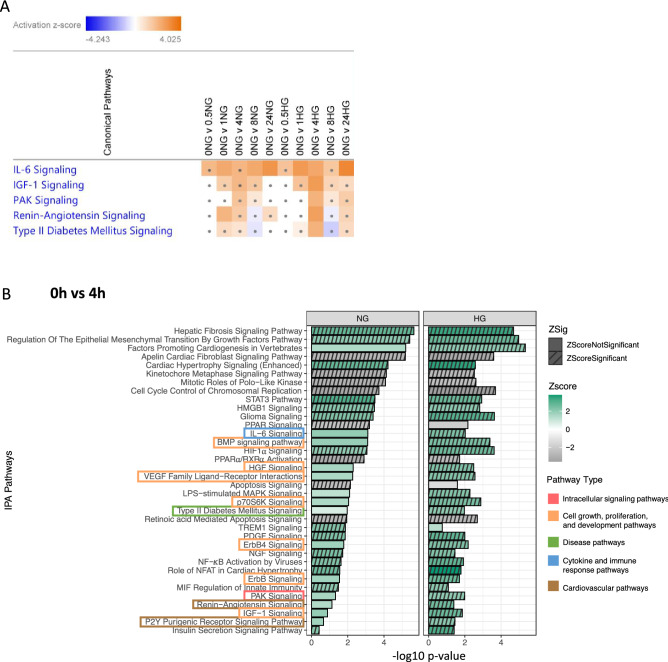
Differential pathway activation/inhibition in HG compared to NG conditions. (**A**) Key pathways highlighted from pathway enrichment analysis of the DEGs (± 1.2 fold change, adjusted p-value < 0.05 for significance) determined at each of the pairwise comparisons of each time point, 0 h vs 0.5 h NG/HG, 0 h vs 1 h NG/HG, 0 h vs 4 h NG/HG, 0 h vs 8 h NG/HG, and 0 h vs 24 h NG/HG using IPA. Dots show non-significant enrichment. (**B**) The 0 vs 4 h pairwise comparison, where most significant pathways are activated/inhibited. A right-tailed Fisher’s Exact test was used to measure significance of the pathway enrichment in the gene set. The horizontal bars show the level of significance (−log10 p-value). The colour coding of bars indicates activation/inhibition determined by the z-score (generated using fold change and curated pathway relationships from the Ingenuity Knowledge Base), > or < 2 respectively. Hashed bars show significant z-scores. Signalling pathways of interest are highlighted by colour coded boxes.

We sought to determine if transcriptional differences induced under NG and HG conditions result in differential pathway activation/inhibition patterns using IPA Pathway enrichment analysis of DEGs identified by pairwise differential expression analysis at each time point relative to the baseline condition (0 h). We highlight five pathways associated with the pathogenesis of T2D, identified by pathway enrichment analysis (Fig. 4A) and heat maps of the DEGs within these pathways (supplemental Fig. 6). Interestingly, the most differences between NG and HG conditions were observed at the 4 h time point where the number of activated/inhibited pathways increased (Fig. 4B). The type 2 diabetes mellitus signalling pathway was significantly activated in HG yet not in NG conditions. Growth factor pathways (hepatocyte growth factor (HGF), vascular endothelial growth factor (VEGF) family ligand-receptor, Erb-B2 Receptor Tyrosine Kinase 4 (ErbB4), insulin-like growth factor (IGF-1), bone morphogenetic protein (BMP), and p70S6K signalling) all were activated in HG but not NG conditions. Additionally, in HG conditions only, the IL-6 signalling pathway was activated along with p21-activated kinase (PAK), Renin-Angiotensin and P2Y purinergic receptor signalling pathways. At the earlier time points of 0.5 h and 1 h (supplemental Fig. 6B,C respectively), most enriched pathways were activated as opposed to inhibited. However, little difference between the activated/inhibited (z-score > or < 2 respectively) pathways was observed in HG compared to NG conditions. Protein kinase A (PKA) signalling is one of the few exceptions and was significantly inhibited at 0.5 h under HG yet not in NG conditions. Also, at the 1 h time point Platelet-derived growth factor (PDGF) signalling was significantly activated in HG but not in NG conditions. At the later time points, 8 and 24 h, the number of activated/inhibited pathways reduced (supplemental Fig. 6D,E). In comparison to NG, high mobility group box 1 (HMGB1) and Endothelin-1 signalling was significantly activated in HG conditions.

### K-means clustering analysis further elucidates the unique effects of the HG condition

**Figure 5:**
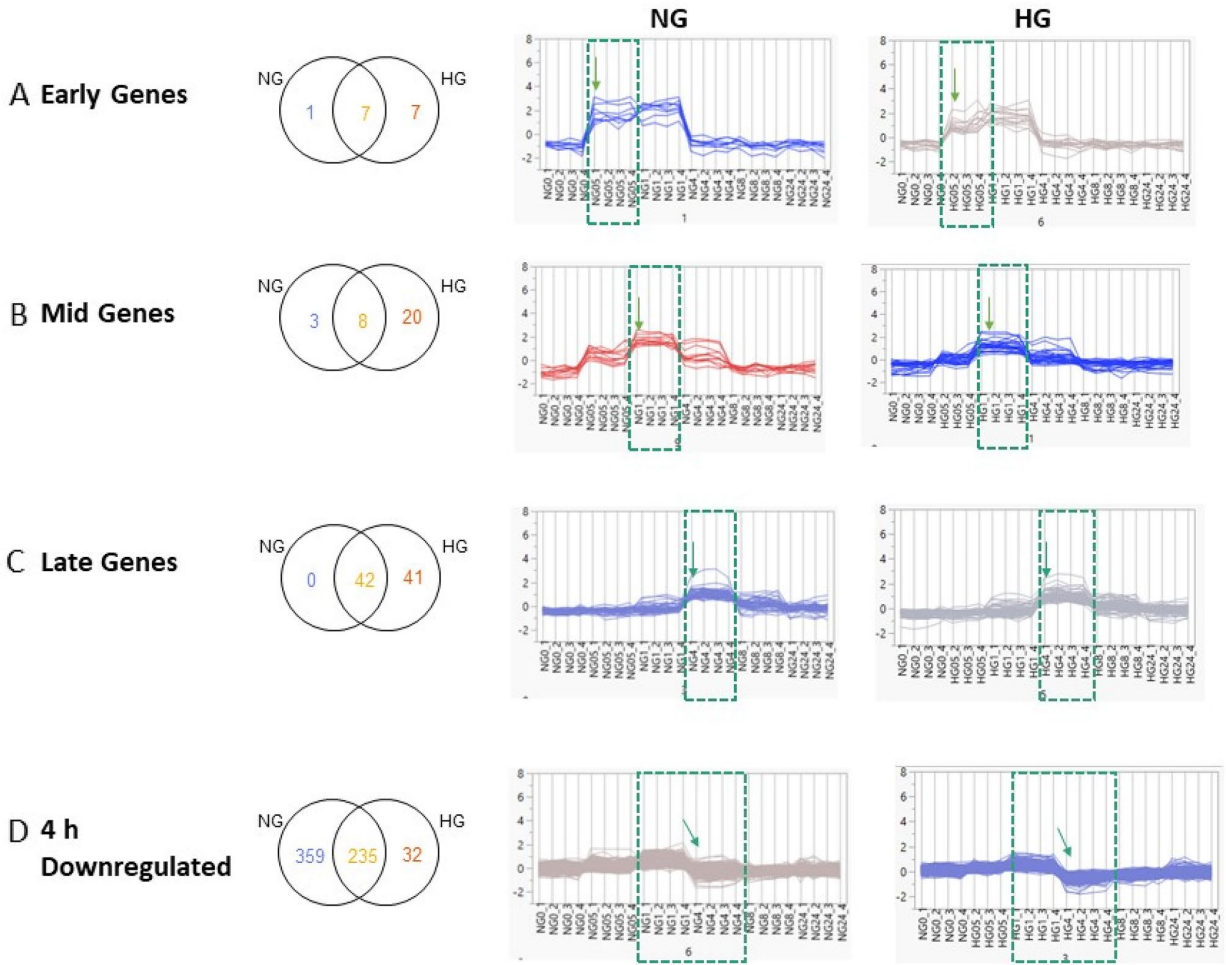
K-means clustering of DEGs in NG and HG conditions. Venn analysis and the expression profiles from K-means clustering of DEGs (± 1.2 fold change, adjusted p-value < 0.05 for significance) in both NG and HG conditions are shown. DEGs from the following pairwise comparisons were analysed for (**A**) early genes, 0 h vs 0.5 h NG/HG, (**B**) mid genes, 0 h vs 1 h NG/HG, (**C**) late genes, 0 h vs 4 h NG/HG (**D**) 4 h downregulated genes, 1 h vs 4 h NG/HG. Green dashed box and arrow highlights the timing of peak expression or switch to downregulation.

K-means clustering analysis was used to identify clusters of DEGs that changed temporarily in a similar manner in each glucose treatment condition. This analysis revealed four major clusters centred on the timing of peak gene expression. Clusters reflected the trend observed in the PCA, in that the cluster profiles were based on the duration of glucose treatment. Three of the cluster profiles grouped genes that displayed a transient response and were termed ‘early’, ‘mid’, and ‘late’, based on the time of peak gene expression post glucose treatment. The fourth cluster highlighted an alternative profile, where a distinct switch to downregulation of the grouped genes was observed. The ‘early’ cluster showed peak expression at 0.5 h post glucose treatment. In HG conditions, the ‘early’ cluster contained 14 genes, with only 8 genes observed in NG conditions, 7 of which were common to both NG and HG conditions (Fig. 5A). A delayed expression peak at 1 h post glucose treatment was observed with the ‘mid’ cluster, where 28 genes where clustered in HG conditions compared to 11 genes in NG conditions, 8 of which were also DE in HG conditions (Fig. 5B). The ‘late’ cluster profile, with the most delayed expression peak at 4 h, also had a larger number of genes in HG conditions, 83, compared to 42 genes in NG conditions, all of which were common to HG condition (Fig. 5C). The ‘downregulated’ cluster profile grouped genes that were downregulated in a clear switch between 1 and 4 h post glucose treatment. In HG conditions only 267 genes were downregulated in this cluster opposed to 594 genes in NG conditions with 235 genes common to both conditions. (Fig. 5D).

**Figure 6:**
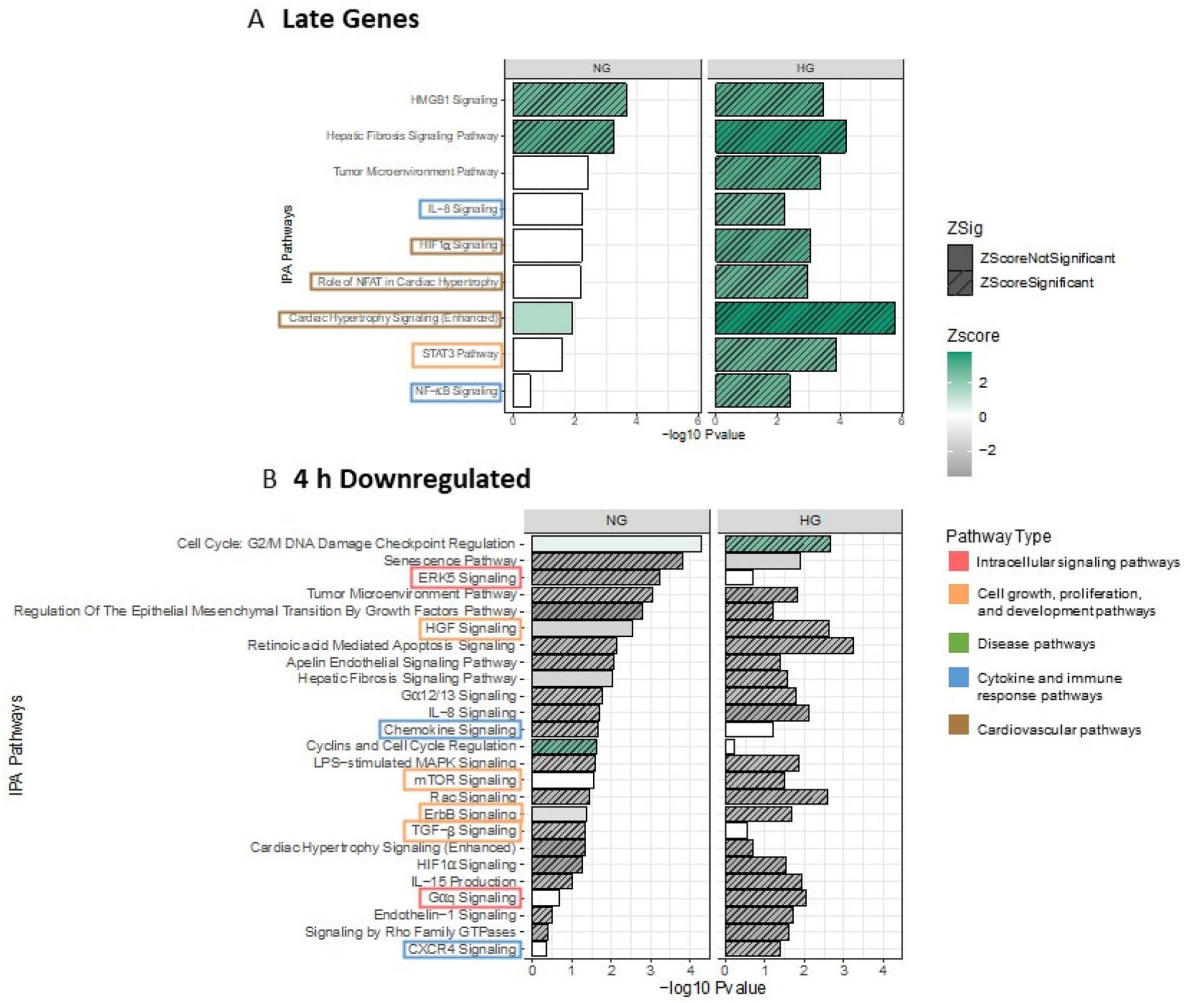
Temporally correlated clusters of DEGs in K-means clusters show differential pathway activation/inhibition in HG compared to NG conditions. (**A**) Signalling pathways obtained from enrichment analysis using IPA of the ‘late’ DEGs cluster determined from the pairwise comparison of 0 h vs 4 h NG/HG. (**B**) Signalling pathways obtained from enrichment analysis using IPA of the ‘4 h downregulated’ DEGs cluster determined at the pairwise comparison of 1 h vs 4 h NG/HG. A right-tailed Fisher’s Exact test was used to measure significance of the pathway enrichment in the gene sets. The horizontal bars show the level of significance (−log10 p-value). The colour coding of bars indicates activation/inhibition determined by the z-score (generated using fold change and curated pathway relationships from the Ingenuity Knowledge Base), > or < 2 respectively. Hashed bars show significant z-scores. Signalling pathways of interest are highlighted by colour coded boxes.

Next, IPA of the temporally correlated cluster of DEGs identified in the K-means clustering analysis was used to identify the signalling pathways activated/inhibited under each glucose treatment. This analysis was possible only for the ‘late’ genes cluster as the ‘early’ and ‘mid’ gene cluster profiles had grouped a small number of genes. The analysis revealed the consistent activation signal in both NG and HG conditions only of three pathways including the High Mobility Group Box 1 (HMGB1) signalling pathway (Fig. 6A). On the other hand, a group of pathways was significantly activated only in the HG condition. These HG-specific activated pathways include the proinflammatory interleukin-8 (IL-8) signalling, hypoxia-inducible factor-1alpha (HIF-1α) signalling, cardiac hypertrophy signalling (enhanced) and the role of nuclear factor of activated T-cells (NFAT) in cardiac hypertrophy (Fig. 6A). Additionally, signalling pathways involved in cell proliferation and inflammation including the signal transducer and activator of transcription 3 (STAT3) pathway and nuclear factor-kappaB (NF-κB) signalling were activated. Heat maps of the DEGs associated with the pathways identified in HG conditions from the ‘late’ cluster are shown (supplementary Fig. 7). The ‘downregulated’ cluster profile grouped the largest set of genes, which was reflected in the pathway analysis where a greater number of pathways were enriched. Interestingly, using the 1 h to 4 h pairwise comparison for the ‘downregulated’ cluster, resulted in most of the enrichment showing inhibition of pathways in both NG and HG conditions. Pathways that were significantly inhibited in HG but not NG conditions included HGF, mTOR, ErbB, Gαq and CXCR4 signalling. Additionally, inhibition of three pathways, chemokine, transforming growth factor-beta (TGF-β) and Extracellular signal regulated kinase 5 (ERK5) signalling were significantly inhibited in NG conditions only (Fig. 6B). Also, Cyclins and cell cycle regulation was activated in NG but not HG conditions.

**Figure 7:**
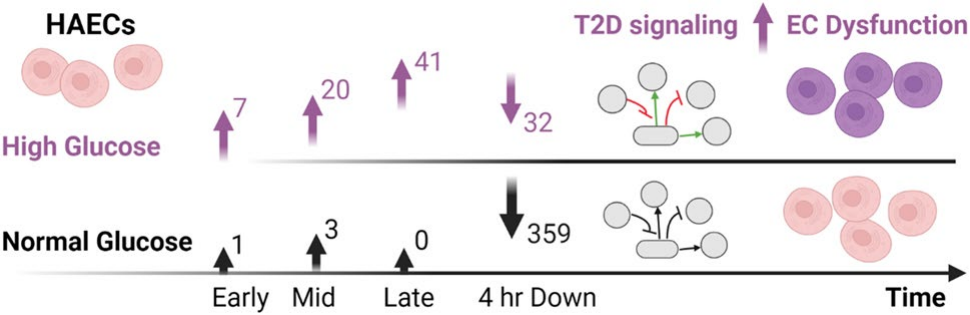
Schematic summary. Unique number of DEGs in each K-means cluster in NG/HG conditions and the resultant downstream effects. Number of unique DEGs are shown in the early, middle, late or 4 h downregulated K-means cluster. The DEGs unique to HG conditions result in activation of pathways that lead to EC dysfunction. The genes associated with the pathways identified from the K-means clusters are listed in supplementary Tables S10 and S11. Figure created with BioRender.com.

The schematic (Fig. 7) highlights the difference in the number of unique DEGs in HG compared to NG conditions in each of the K-means clusters. The DEGs specific to HG conditions result in activation of pathways that lead to EC dysfunction.

## Discussion

It is well-documented that hyperglycaemia induces EC dysfunction, which plays a critical role in the manifestation and development of diabetic complications. Here, we demonstrated for the first time, to our knowledge, the temporal effect of glucose on the HAEC transcriptome. Our results revealed both consistent and opposing temporal transcriptional changes in response to normal and high glucose levels that ultimately lead to differential signalling pathway activation and/or inhibition.

Given the dynamic nature of biological processes, gene expression studies using a time-series approach enables the detection of transient transcriptional changes and temporal patterns that are often missed by cross-sectional designs. Thus, we chose this approach to better understand the timing of gene expression changes and whether genes expressed at early, middle, or late time points, activate/inhibit specific pathways within these time frames and differentially under HG. The number of DEGs at each time point followed a similar trend under both NG and HG conditions. From the treatment durations that we selected (0.5, 1, 4, 8 and 24 h), 4 h of either NG or HG conditions resulted in the peak number of DEGs. It is possible that due to the HAECs being exposed to reduced serum conditions overnight, (serum starving cells prior to beginning experiments is well documented^16^), the addition of either NG or HG treatment containing full serum at the start of the experiment, resulted in an initial burst of transcription, supported by the observance that the majority of DEGs were upregulated at 0.5 and 1 h time points compared to the baseline. Equally, comparative analysis showed that HG treatment induced specific changes from the earliest time point investigated (i.e. 0.5 h). By 4 h and subsequent time points of either NG or HG treatment, the even ratio of upregulated and downregulated DEGs suggests that by 4 h the cells reach a state of balanced up and downregulation following the initial burst of transcription. This is further supported by the analysis between time points, whereby the majority of DEGs observed between 0.5–1 h are downregulated compensating for the earlier upregulation at 0.5 h.

This study investigated changes in the transcriptome under both NG and HG conditions. Our initial focus was to determine which genes were differentially expressed in HG compared to NG conditions and the timing of their expression. Venn analysis first established groups of DEGs that are either unique or common to NG and HG conditions. Interestingly, it was mainly the later time points (8 and 24 h), where we identified genes that were significantly up or downregulated in HG conditions yet showed the reverse trend in NG conditions. One of the significantly upregulated genes observed following 8 h under HG was the *WNT2B* gene. Previous studies have shown that Wnt ligands and the subsequent Wnt signalling pathways activated are implicated in the control of various processes that are critical in the development of T2DM and its complications^17^. Our data, observing the effect of HG in HAECs, suggest that Wnt2b is also implicated in the cardiovascular health of T2DM and warrants further investigation. Additionally, we observed significant upregulation of the *AMIGO2* gene at 24 h under HG, as opposed to a downregulated trend under NG conditions. A gene expression profiling study in mice lungs also showed upregulation of *AMIGO2* in the hyperglycaemic lung^18^. Moreover, at 24 h under HG, upregulation of *COL1A2* a major component of extracellular matrix was observed. Previously, *COL1A2* upregulation has been measured in both mice with diabetic nephropathy and HK2 cells treated with HG^19^.

Further to identifying DEGs specific to HG conditions, our pathway enrichment analysis revealed differential activation and inhibition of signalling pathways under HG compared to NG conditions, which was also influenced by the duration of glucose treatment. We were able to see the effects of HG conditions at the earlier time points, 0.5 h and 1 h, where significant inhibition of PKA and activation of PDGF signalling was observed respectively. PDGFs are synthesized by platelets and are growth factors that regulate cell growth and division^20^. It is known that PDGFs can affect the processes of T2DM and its complications via various signalling pathways (including PKC Ө and PKCε, NF-κB, PI3K, PLCγ, Src/ Smad1/Col4, JAK/STAT, PI3K/Akt/mTOR, p38 MAPKSHP-1 and ERK/Akt pathways). Particularly, through the inflammatory and angiogenic effects of PDGFs, endothelial migration and proliferation is impaired^21–23^.

Corresponding with the peak number of DEGs observed at 4 h in both NG and HG conditions, we also observed the greatest effect in terms of signalling pathways activated/inhibited at the 4 h time point. A key pathway that was significantly activated at 4 h HG conditions yet not in NG conditions was the T2DM signalling pathway. As predicted, upregulation of *MAPK*, *PI3K*, and *NF-κB* subunits were detected. Furthermore, at the 4 h time point we detected significant activation of multiple growth factor and cell proliferation signalling pathways (HGF, VEGF, ErbB4, IGF-1, BMP, and p70S6K) in HG but not NG conditions. The growth factor signalling pathways identified here have been shown to have clear implications in the development of T2DM and its complications^24–31^. Additionally, we observed significant activation of PAK signalling, which has been shown to be involved in metabolic processes, including glucose homeostasis with a role in regulating cell proliferation and in the progression of vascular disorders^32^. The 4 h time point enrichment analysis also highlighted significant activation of the interleukin-6 (IL-6) signalling pathway, specific to HG conditions only. IL-6 has been hypothesized to play a critical role in the pathophysiology of T2DM^33^ (33). Activation of the P2Y purinergic receptor signalling pathway at 4 h HG treatment was also observed. Purinergic signalling involves multiple receptors and extracellular enzymes that provides a system of cell–cell communication through the recognition and degradation of extracellular nucleotides and adenosine. An increasing number of studies have placed the purinergic system as a key player in numerous physiopathological conditions, including those involved in the inflammatory response such as T2DM^34^. Moreover, our data show significant activation of renin-angiotensin signalling at 4 h post HG treatment, however not in NG conditions. It is well known that the renin-angiotensin system (RAS) is activated and initiates the progression of T2DM and its complications^35^.

The analysis showed a reduction in the number of enriched pathways at the later time points of 8 and 24 h in both NG and HG conditions. A marked difference that we observed at the 24 h point was the significant activation of Endothelin-1 signalling in HG but not in NG conditions. Our data are in line with the evidence that hyperglycaemia-induced endothelial dysfunction is partially mediated through increased activation of the endothelin system, which plays an important role in the pathophysiology of diabetes-associated cardiovascular diseases^36^. Interestingly, we observed significant activation of HMGB1 signalling in both NG and HG conditions at the 0.5, 1, and 4 h time points. However, at the 24 h time point activation was no longer significant in NG conditions. As a late mediator of inflammation, HMBG1 has been shown to be a critical facilitator in the pathogenesis of a variety of diseases including T2DM^37^. Only a longer time course would highlight whether HMGB1 signalling remains activated in HG conditions only.

The time-series analysis of glucose treatment in HAECs aimed to identify sets of DEGs according to their temporal gene expression profile. Therefore, potentially identifying groups of coregulated genes at specific time points to improve the understanding of the biological processes that are activated or inhibited. To this end, K-means clustering classified NG or HG DEGs into four clear temporal profiles based on the timing of peak, or a clear switch to downregulation of gene expression. In the ‘early’, ‘middle’ and ‘late’ gene clusters, all of which grouped upregulated genes, we observed a larger number of DEGs in HG compared to NG conditions. Within each cluster, the majority of DEGs were common to both NG and HG conditions, with few unique DEGs in NG conditions, therefore indicating a significant effect of HG conditions on the transcriptional response at each time point. Indeed, pathway enrichment analysis of the ‘late’ gene cluster identified multiple pathways previously implicated in the cellular response to hyperglycaemic conditions and development of T2D complications. Activation of the IL-8 signalling pathway was observed in HG conditions. Studies in vitro have shown that glucose causes increased endothelial production of IL-8^38^ and in vivo, circulating IL-8 levels are increased in patients with T2D, who also displayed a more severe inflammatory and cardiometabolic profile^39^. Furthermore, the ‘late’ gene cluster significantly activated several transcription factors. Interestingly, HIF-1α signalling was activated in HG conditions. Other reports have detailed the insufficient activation of HIF-1α signalling due to the lack of HIF-1α stability and function from hyperglycaemic conditions^40^. In contradiction, through a carbohydrate response element binding protein-mediated mechanism, HG has also been shown to activate HIF-1α signalling in glomerular mesangial cells^41^, therefore meriting further investigation into the dysregulation of HIF-1α signalling in hyperglycaemic conditions. Additionally, the role of NFAT in cardiac hypertrophy signalling was significantly activated in cells treated with HG. An increasing number of studies have reported vital roles for NFAT in the development of diabetes and atherosclerosis through endothelial cell damage, foam cell formation and plaque calcification^42^. Moreover, two master transcription factors, STAT3 and NF-κB were activated in HG conditions, both of which are well documented in the development of diabetes and its complications. STAT3 signalling mediates the effects of multiple cytokines, resulting in the transcription of genes that control cell survival, proliferation, and immune response^43^. Activation of NF-κB through prolonged hyperglycaemia, induces expression of various cytokines, chemokines and cell adhesion molecules leading to endothelial dysfunction and further vascular complications^44^. Equally, short durations of HG treatment have been shown to activate NF-κB in endothelial cells^45^. Potential for STAT3 and NF-κB crosstalk could occur under hyperglycaemic conditions, as has been shown in other diseases, with collaboration of the two transcription factors resulting in the development of diabetic complications^46^.

In reverse to the other clusters, which grouped upregulated genes, the ‘downregulated’ group of DEGs showed a transcriptional profile demonstrating a switch to downregulation between 1 and 4 h and contained more genes in NG compared to HG conditions. Indeed, the previous pathway enrichment analysis of the DEGs at the 4 h time point compared to baseline resulted in more ‘activation’ pathway hits in HG compared to NG conditions. This is supported by the analysis of the ‘downregulated’ cluster, which uses the 1 h and 4 h time points pairwise comparison for DEGs and highlights the increased number of DEGs that are downregulated in NG compared to HG conditions. The data showed that most significantly enriched pathways in this cluster were inhibited. Interestingly, Chemokine, TGF-β and ERK5 signalling were inhibited in NG conditions only. Previous studies have shown that TGF-β is elevated in hyperglycaemic conditions and has a role in the pathogenesis of obesity and T2D through Smad signalling^47^. The downregulation of ERK5 signalling observed in NG conditions is interesting as it has been formerly shown that ERK5 regulates glucose-induced endothelin-1 expression^48^.

Our results show that many of the DEGs and activated pathways in HG conditions identified from our data have been previously implicated in the pathology and development of complications of T2D. Characterized by two main deficiencies; impaired secretion of insulin by β-cells and the inability of insulin-sensitive tissues to respond to insulin (insulin resistance), the pathology of T2D reverts from insulin release and activity that are essential processes for glucose homeostasis. Our analysis showed that some of the pathways activated predominantly at the 4 h time point in HG conditions have direct roles in mediating blood glucose homeostasis and have been implicated in insulin resistance, particularly PAK1, HGF, BMP and IGF-1 signaling pathways^24,28,30,32^. Dysregulation of these pathways in hyperglycaemic conditions can perturb glucose homeostasis, thus contributing to the pathology of T2D. Concurrent with disruption of the metabolic balance is an increase in inflammation through direct activation of proinflammatory pathways, including IL-6 and IL-8 signaling pathways, which we identified in our data. Additionally, signaling pathways may indirectly increase inflammation through activation of other pathways, for example HMGB1 signaling through NF-κB activation. A key element of our data is the activation of genes and ultimately signaling pathways that are implicated in the pathology of T2D after a short exposure to HG conditions and it is likely that these transcriptional changes are consolidated during and after longer exposures to hyperglycemic conditions.

In summary, pathway enrichment analysis of DEGs in NG and HG conditions identified numerous pathways specific to HG conditions that have previously been implicated in hyperglycaemia and the development of T2DM and its complications. Our results show that HAECs respond quickly to HG conditions and from the time course of HG applied that 1–4 h of HG treatment caused the strongest and most significant alteration of the HAEC transcriptome profile. These results clearly show that up/downregulation of particular genes takes place at earlier time points and suggest that these early events may influence the regulation of genes at the later time points ultimately leading to activation/inhibition of pathways. Contrasting the transcriptional changes that take place only under HG conditions revealed a set of genes and temporal patterns that implicate genes and pathways associated with hyperglycaemic conditions and development of T2D complications. The results shown here warrant the inclusion of earlier time points when studying glucose exposure in both cell-based and animal model studies, including in investigations of biomarkers of early dysfunction-associated transcriptional events. Unlike the prolonged and repeated exposure to HG that endothelial cells experience in vivo under disease conditions, this study is limited in that HAECs were only exposed to a short duration of HG in vitro. However, the observations made here show that endothelial cells can exhibit a fast temporal transcriptional response to glucose with differences that can be attributed only to hyperglycaemic conditions.

Although early transient differences in expression seem to fade with time, their consequences on protein levels and subsequently cell processes through activation and/or inhibition of pathways likely remain as supported by IPA analysis. It might be revealing to investigate if these early transcriptional changes are epigenetically regulated and if they result in rapid changes at the protein and metabolic levels as well, both in vitro and under long-term and repetitive exposure to HG as seen in diabetics. Future study of the effects of HG exposure in endothelial cells could build on the mounting evidence of epigenetic changes that take place following HG exposure and metabolic memory. Moreover, investigations are warranted to elucidate whether HG exposure leaves epigenetic marks that influence the transcriptional response of endothelial cells in both the continued presence or interestingly, the absence of HG (mimicking management of the disease state). This investigation enhances our understanding of the genes and pathways that are transcriptionally responsive in the early stages of endothelial cell dysfunction that might ultimately lead to the progression of diabetic complications.

## Materials and methods

### Cell culture

Human aortic endothelial cells (HAECs) obtained from Lonza were grown in normal physiological levels of glucose (NG) (5.5 mM d-glucose) EGM™ 2 Endothelial Cell Growth Medium-2 BulletKit™ (Lonza) containing 2% fetal bovine serum (FBS) as per manufacturer’s instructions, and maintained at 37 °C, 5% CO_2_.

### Glucose treatment

For the RNA-seq glucose time course experiment, HAECs were initially cultured in NG EGM™ 2 Endothelial Cell Growth Medium-2 BulletKit™ (Lonza) containing 2% fetal bovine serum (and all essential growth factors and supplements). HAECs were then exposed to NG EGM™ 2 Endothelial Cell Growth Medium-2 BulletKit™ (Lonza) containing reduced serum (0.2% FBS) overnight (growth factors and supplements remained in the media). HAECs were then treated with EGM™ 2 Endothelial Cell Growth Medium-2 BulletKit™ (Lonza) containing 2% FBS and either 5.5 mM d-glucose as is found in the EBM™-2 Basal Medium for normal glucose conditions (with no additional d-glucose supplementation required) or supplemented with additional d-glucose to a total of 25 mM d-glucose for high glucose conditions. HAECs were then harvested at 0, 0.5, 1, 4, 8 and 24 h post treatment. As the culture media contains supplements and growth factors, to compensate for any effects the culture media may have on the transcriptome, samples were harvested at the same time points in both NG and HG conditions. There was no difference in the glucose concentration of the baseline sample, NG 0 h and the subsequent time points. Four biological replicates were harvested for RNA-seq analysis.

### RNA extraction and RNA-seq

Total RNA was isolated from HAECs (cultured and treated as described above) using the RNeasy Mini Kit (Qiagen) according to manufacturer’s instructions. RNA was quantified using a Qubit (ThermoFisher Scientific) and integrity (RNA Integrity Number, RIN) assessed using an Agilent 2100 Bioanalyzer (Agilent). Throughout the experimental protocols, careful attention was paid to minimize batch effects. Importantly, the samples and libraries were randomized and processed the same way from total RNA extraction and library preparation, to the sequencing. Sequencing libraries were prepared using 500 ng of RNA (RIN > 8) and the KAPA Stranded RNA-Seq kit with RiboErase (Kapa Biosystems). The libraries were quality-checked using an Agilent 2100 Bioanalyzer (Agilent) and quantified using qPCR prior to multiplexing and paired-end sequencing using NextSeq 500/550 High Output v2 kit (150 cycles) with a NextSeq 550 instrument (Illumina) within the Core Technology Platform at New York University Abu Dhabi.

### Bioinformatic analyses of RNA-seq data

Raw sequencing reads were quality controlled using the FASTQC tool prior to processing using Trimmomatic v0.36 to remove barcodes, adapter sequences and low-quality bases (Phred quality score < 22). Filtered reads were then mapped to the human reference genome (Ensembl GRCh38 release-84) using STAR v2.5.2a. Genes not expressed or with very low expression levels (less than five reads in 2 or more samples in any given condition) were removed, which resulted in retention of 18,676 genes for downstream analysis.

### RNA-seq data normalization, differential expression, and pathway enrichment analyses

Filtered raw counts were log2 transformed prior to normalization using JMP Genomics 8 (SAS Institute). Data were batch normalized to reduce any potential technical batch effects. Differential expression analysis, evaluated by analysis of variance (ANOVA), was done using the Basic Expression workflow implemented in JMP Genomics 8 (SAS Institute). For each of the effects tested (Glucose Level and Time), a P value measuring significance of differential expression and fold change were obtained for each gene. False discovery rates (FDR) were calculated according to the Benjamini–Hochberg method implemented in JMP Genomics 8. An FDR of 5% was used as a threshold for statistical significance. K-means clustering analysis was done to identify clusters of co-expressed genes across time points using JMP genomics 8 (SAS Institute). The algorithm iteratively optimizes the cluster seeds by creating temporary clusters and calculating their means until the points converge, and the clusters are optimally separated. The IPA algorithm uses the Ingenuity Knowledge Base and is based on a collection of experimental observations manually curated from the literature of other databases. Differentially expressed genes that have an FDR < 5% and an absolute fold change greater than 1.2 were included in this analysis. A right-tailed Fisher’s Exact test was used to measure significance of the pathway enrichment in the gene set. Cause–effect relationships with a direction of the causal effect (activation or inhibition) of pathways is measured by a z-score generated using fold change and curated pathway relationships from the Ingenuity Knowledge Base^49^.

### qPCR validation

To validate the expression level of 12 genes, quantitative PCR was performed using TaqMan gene expression assays (ThermoFisher assays; SMAD6, Hs00178579_m1; CXCL8, Hs00174103_m1; TGFA, Hs00899865_m1; IRF6, Hs01062178_m1; WNT2B, Hs00921614_m1; CXCL12, Hs03676656_mH; IL11, Hs01055414_ml; VEGFC, Hs01099203_m1; CCL2, Hs00234140_m1; CXXC5, Hs00212840_m1; PDGFA; Hs00234994_m1, EDN1; Hs00174961_m1). In total, 43 samples representing 3–4 replicates under both HG and NG conditions at five time points were assayed in duplicates. Reverse transcription was done using 15 ng of total RNA and 1 µl of reverse-transcription master mix in a total reaction volume of 5 µl followed by a standard 192.24 dynamic array gene expression TaqMan workflow procedure (PN68000088, Fluidigm) using a BioMark HD instrument (Fluidigm). Relative expression levels were measured using the 2–ΔΔCt analysis method and normalized using the housekeeping gene ACTB”.

## Supplementary Information

Supplementary Information.

## Data Availability

Gene expression data is deposited in the Gene Expression Omnibus database under accession no. GSE203284.
