# Supplementary material for: Hyperglycemic conditions induce rapid cell dysfunction-promoting transcriptional alterations in human aortic endothelial cells
Source: Sci Rep. 2022 Dec 3;12:20912. doi: 10.1038/s41598-022-24999-5 (PMC9719474; doi:10.1038/s41598-022-24999-5)
Supplement: Supplementary file 1 — Supplementary Information. [file 41598_2022_24999_MOESM1_ESM.pdf]

Supplementary Information

Hyperglycemic conditions induce rapid cell dysfunction-promoting transcriptional alterations in human aortic endothelial cells

Odmaa Bayaraa[2] , Claire K. Inman[1], Sneha A. Thomas[1], Fatima Al Jallaf[2], Manar AlShaikh[1], Youssef Idaghdour[1,2†*], Louise Ashall[1†*]

1 Public Health Research Center, New York University Abu Dhabi, Abu Dhabi, United Arab Emirates.

2 Program in Biology, Division of Science and Mathematics, New York University Abu Dhabi, Abu Dhabi, United Arab Emirates.

† These authors contributed equally to this work.

* Co-correspondence: youssef.idaghdour@nyu.edu, louise.ashall@nyu.edu

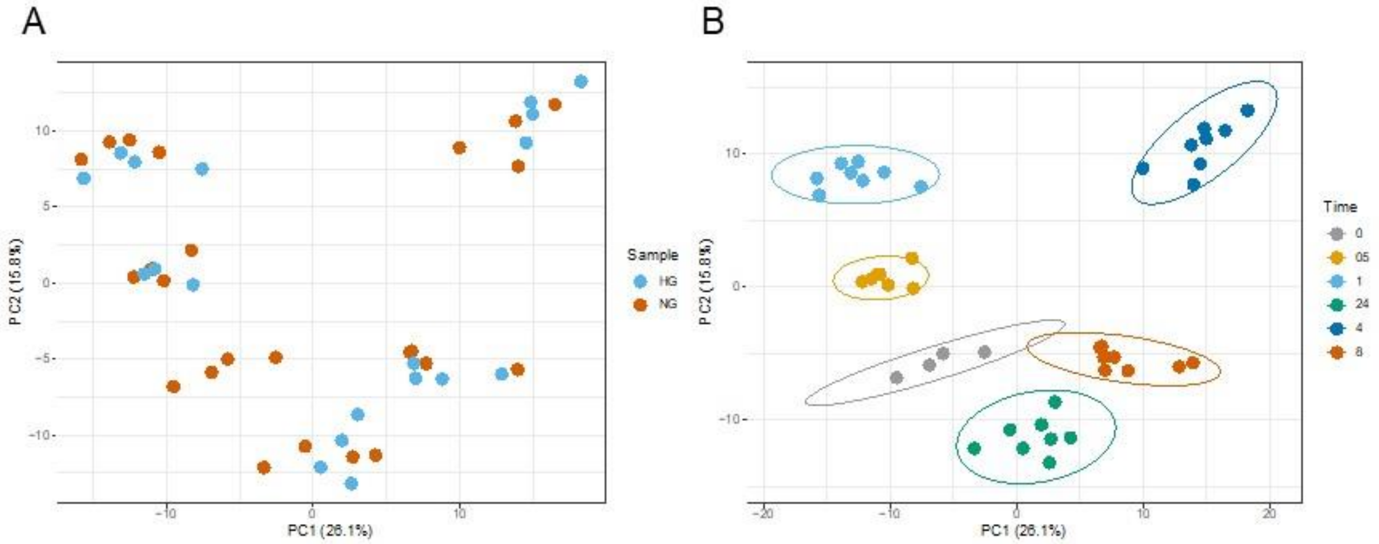

Supplemental figure 1. PCA plots of glucose and time. PCA of each time point in the HAEC transcriptome according to A) glucose condition and B) time.

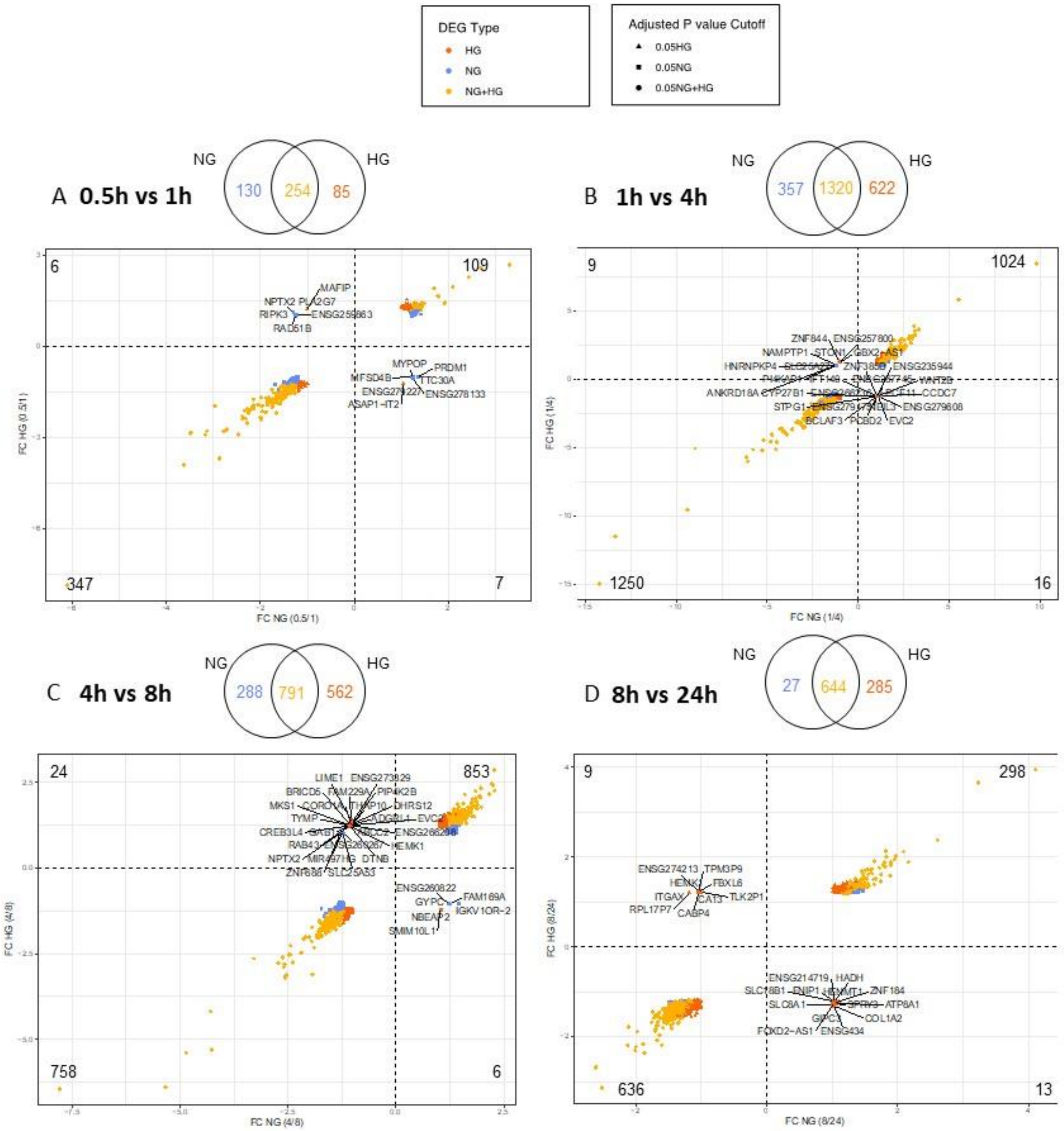

Supplemental figure 2. Transcriptional changes in HAECs between time points from differential glucose treatment. Venn analysis and scatterplots of fold change of DEGs (±1.2 fold change, adjusted p-value <0.05 for significance) with pairwise comparisons of A) 0.5 h vs 1 h NG/HG, B) 1 h vs 4 h NG/HG, C) 4 h vs 8 h NG/HG and D) 8 h vs 24 h NG.

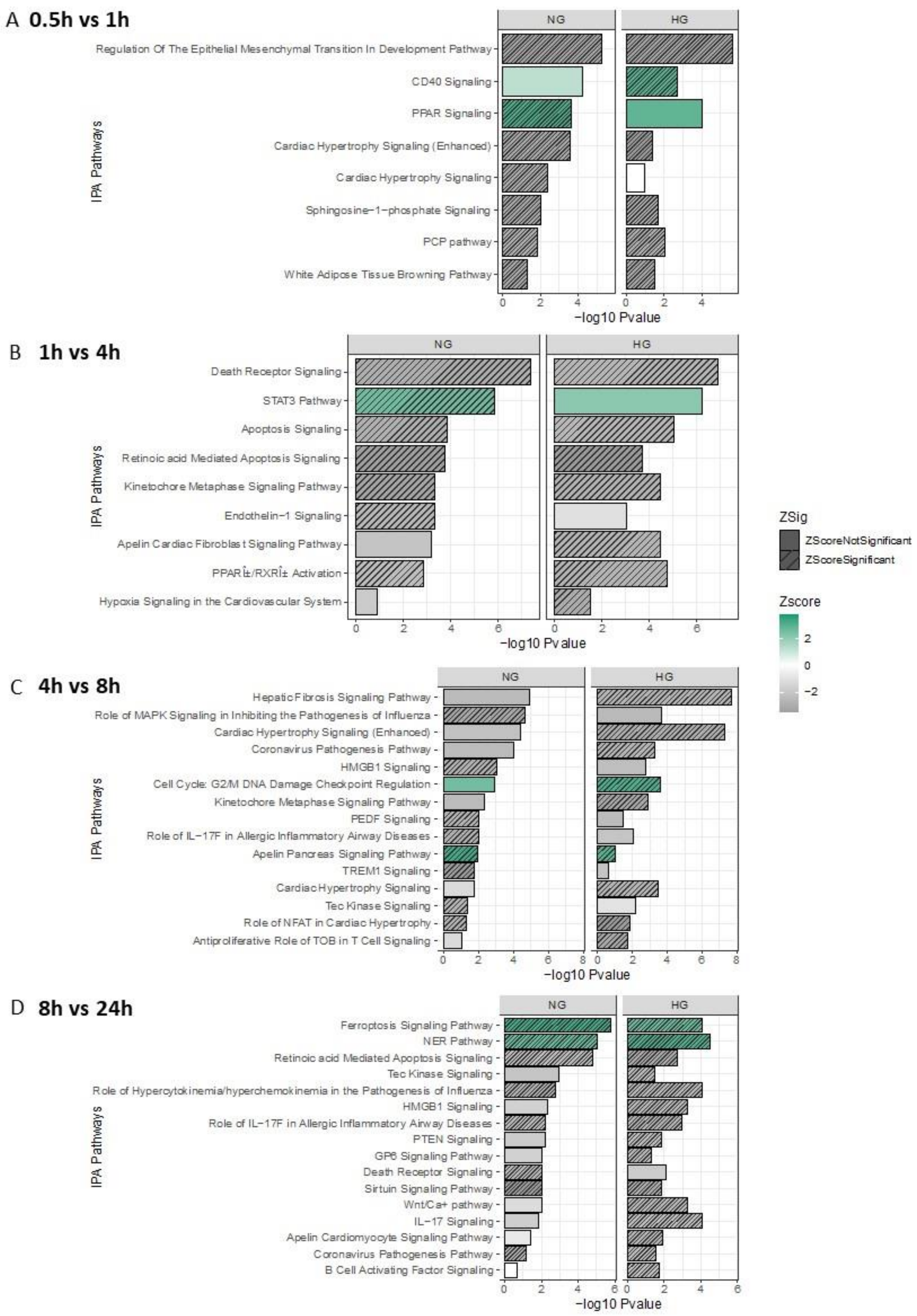

A 0.5h vs 1h
NG
HG
Regulation Of The Epithelial Mesenchymal Transition In Development Pathway
CD40 Signaling
PPAR Signaling
Cardiac Hypertrophy Signaling (Enhanced)
Cardiac Hypertrophy Signaling
Sphingosine−1−phosphate Signaling
PCP pathway
White Adipose Tissue Browning Pathway
IPA Pathways
−log10 Pvalue
B 1h vs 4h
Death Receptor Signaling
STAT3 Pathway
Apoptosis Signaling
Retinoic acid Mediated Apoptosis Signaling
Kinetochore Metaphase Signaling Pathway
Endothelin−1 Signaling
Apelin Cardiac Fibroblast Signaling Pathway
PPARα/RXRα Activation
Hypoxia Signaling in the Cardiovascular System
ZSig
ZScoreNotSignificant
ZScoreSignificant
Zscore
C 4h vs 8h
Hepatic Fibrosis Signaling Pathway
Role of MAPK Signaling in Inhibiting the Pathogenesis of Influenza
Cardiac Hypertrophy Signaling (Enhanced)
Coronavirus Pathogenesis Pathway
HMGB1 Signaling
Cell Cycle: G2/M DNA Damage Checkpoint Regulation
Kinetochore Metaphase Signaling Pathway
PEDF Signaling
Role of IL−17F in Allergic Inflammatory Airway Diseases
Apelin Pancreas Signaling Pathway
TREM1 Signaling
Cardiac Hypertrophy Signaling
Tec Kinase Signaling
Role of NFAT in Cardiac Hypertrophy
Antiproliferative Role of TOB in T Cell Signaling
D 8h vs 24h
Ferroptosis Signaling Pathway
NER Pathway
Retinoic acid Mediated Apoptosis Signaling
Tec Kinase Signaling
Role of Hypercytokinemia/hyperchemokinemia in the Pathogenesis of Influenza
HMGB1 Signaling
Role of IL−17F in Allergic Inflammatory Airway Diseases
PTEN Signaling
GP6 Signaling Pathway
Death Receptor Signaling
Sirtuin Signaling Pathway
Wnt/Ca+ pathway
IL−17 Signaling
Apelin Cardiomyocyte Signaling Pathway
Coronavirus Pathogenesis Pathway
B Cell Activating Factor Signaling

Supplemental figure 3. Differential pathway activation/inhibition in between time point analysis in HG compared to NG conditions. Signalling pathways obtained from enrichment analysis using IPA of the DEGs determined at each of the pairwise comparisons of A) 0.5 h vs 1 h NG/HG, B) 1 h vs 4 h NG/HG, C) 4 h vs 8 h NG/HG and D) 8 h vs 24 h NG. The horizontal bars show the level of significance (-log10 p-value). The colour coding of bars indicates activation/inhibition determined by the z-score, > or < 2 respectively Hashed bars show significant z-scores.

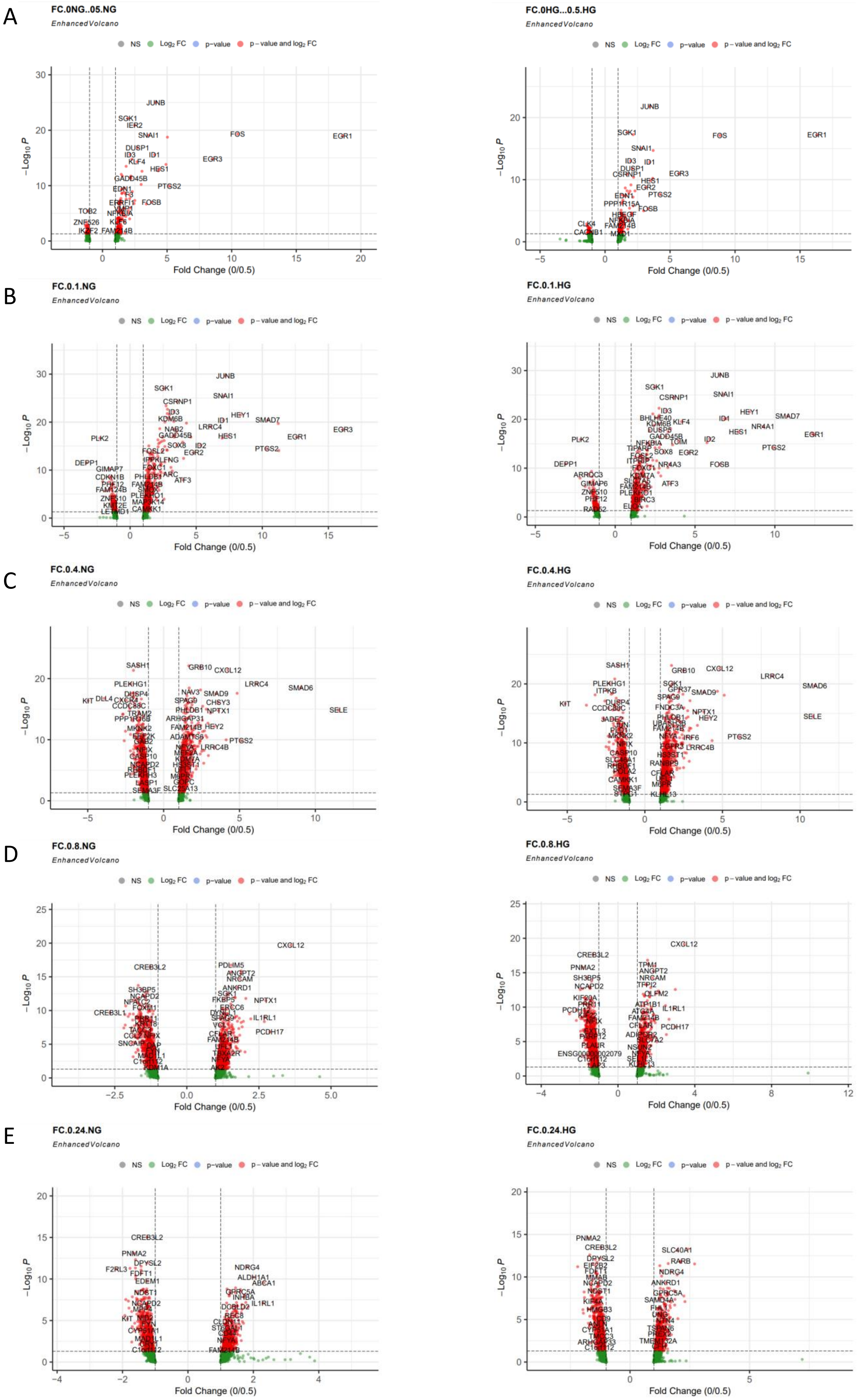

A
FC.0NG..05.NG
EnhancedVolcano
NS Log2 FC p-value p-value and log2 FC
-Log10 P
Fold Change (0/0.5)
JUNB
SGK1
IER2
SNAI1
FOS
EGR1
DUSP1
ID3
ID1
KLF4
EGR3
HES1
GADD45B
PTGS2
EDN1
FOSB
TOB2
ZNF526
FC.0HG...0.5.HG
EnhancedVolcano
JUNB
SGK1
FOS
EGR1
SNAI1
ID3
ID1
DUSP1
CSRNP1
EGR3
HES1
EGR2
PTGS2
EDN1
PPP1R15A
FOSB
HBEGF
NFKBIA
FAM214B
CLK4
CACNB1
MXD1
B
FC.0.1.NG
EnhancedVolcano
JUNB
SGK1
SNAI1
CSRNP1
HEY1
SMAD7
ID3
ID1
KDM6B
LRRC4
EGR3
PLK2
NAB2
GADD45B
HES1
EGR1
SOX8
ID2
EGR2
PTGS2
DEPP1
GIMAP7
CDKN1B
ATF3
FC.0.1.HG
EnhancedVolcano
JUNB
SGK1
SNAI1
CSRNP1
HEY1
SMAD7
ID3
BHLHE40
KDM6B
KLF4
ID1
NR4A1
PLK2
DUSP5
GADD45B
ID2
HES1
EGR1
NFKBIA
SOX8
EGR2
PTGS2
DEPP1
NR4A3
FOSB
ARRDC3
GIMAP6
ATF3
C
FC.0.4.NG
EnhancedVolcano
SASH1
GRB10
CXCL12
LRRC4
SMAD6
PLEKHG1
NAV3
SMAD9
KIT
DLL4
DUSP4
SPAG9
CHSY3
SELE
NPTX1
HEY2
PTGS2
LRRC4B
FC.0.4.HG
EnhancedVolcano
SASH1
GRB10
CXCL12
LRRC4
SMAD6
PLEKHG1
SGK1
SMAD9
SPAG9
KIT
NPTX1
HEY2
SELE
PTGS2
LRRC4B
IRF6
D
FC.0.8.NG
EnhancedVolcano
CXCL12
CREB3L2
PDLIM5
ANGPT2
NRCAM
ANKRD1
SGK1
NPTX1
CREB3L1
IL1RL1
PCDH17
FC.0.8.HG
EnhancedVolcano
CXCL12
CREB3L2
PNMA2
TPM1
ANGPT2
NRCAM
SH3BP5
NCAPD2
OLFM2
IL1RL1
PCDH17
E
FC.0.24.NG
EnhancedVolcano
CREB3L2
PNMA2
DPYSL2
F2RL3
NDRG4
ALDH1A1
ABCA1
EDEM1
IL1RL1
FC.0.24.HG
EnhancedVolcano
PNMA2
CREB3L2
SLC40A1
RARB
NDRG4
ANKRD1

Supplemental figure 4. Volcano plot of RNA-seq data in both NG and HG conditions for each pairwise comparison. Significant DEGs (±1.2 fold change, adjusted p-value <0.05 for significance) are shown in red. Each pairwise comparison is shown, A) 0 h vs 0.5 h NG/HG, B) 0 h vs 1 h NG/HG, C) 0 h vs 4 h NG/HG, D) 0 h vs 8 h NG/HG, and E) 0 h vs 24 h NG/HG.

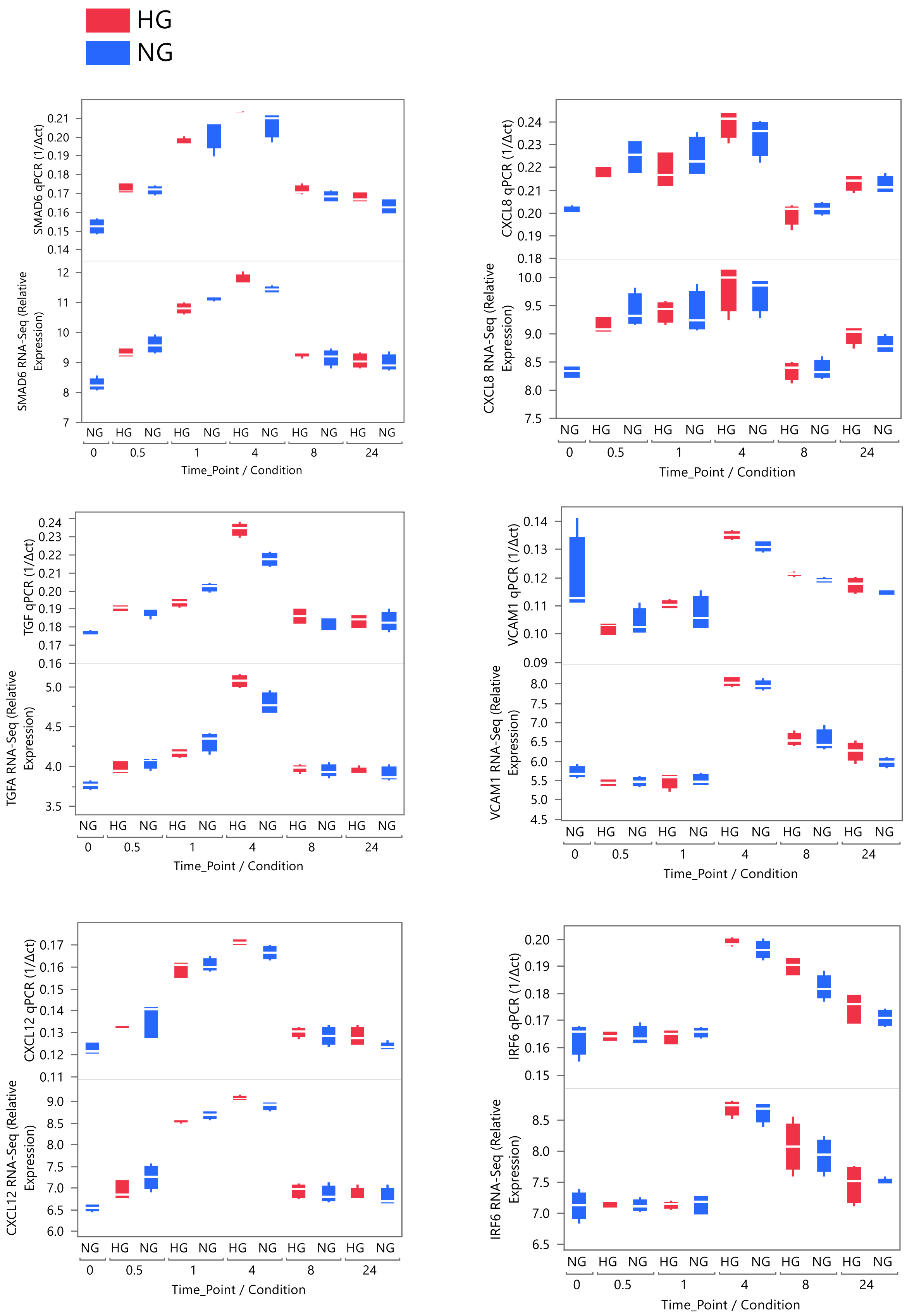
HG
NG
SMAD6 qPCR (1/Δct)
SMAD6 RNA-Seq (Relative Expression)
CXCL8 qPCR (1/Δct)
CXCL8 RNA-Seq (Relative Expression)
TGF qPCR (1/Δct)
TGFA RNA-Seq (Relative Expression)
VCAM1 qPCR (1/Δct)
VCAM1 RNA-Seq (Relative Expression)
CXCL12 qPCR (1/Δct)
CXCL12 RNA-Seq (Relative Expression)
IRF6 qPCR (1/Δct)
IRF6 RNA-Seq (Relative Expression)
NG HG NG HG NG HG NG HG NG HG NG
0 0.5 1 4 8 24
Time_Point / Condition

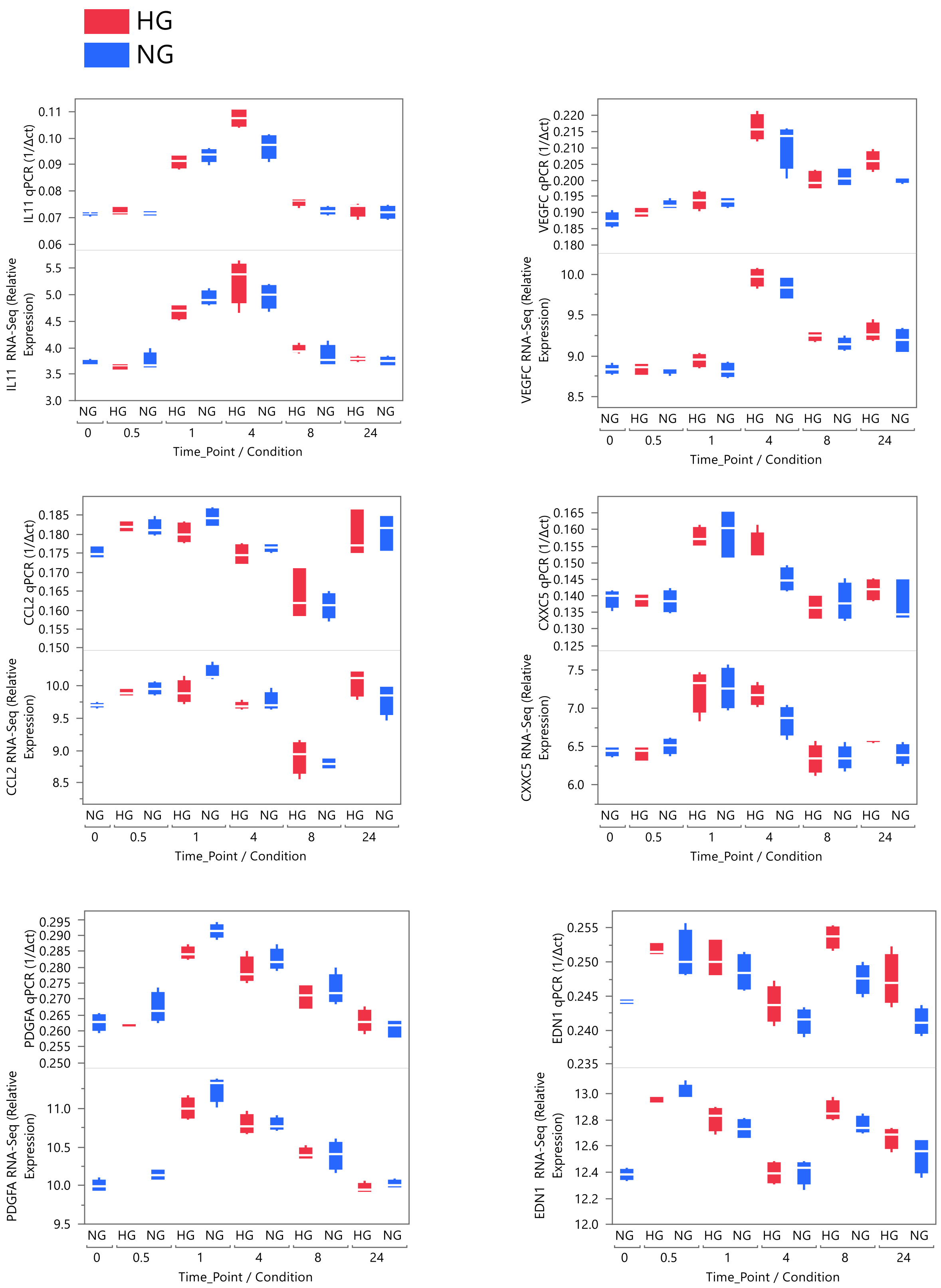

Supplemental figure 5. RT-qPCR validation of RNA sequencing data. Box plots show relative expression levels of 12 genes in Normal Glucose (NG, blue) and High Glucose (HG, red) conditions at five time-points (0, 0.5, 1, 4, 8 and 24 h post treatment) quantified using RNASeq and RT-qPCR. Bar and whiskers represent mean ± SD. The qPCR data is provided in Supplementary Table 13.

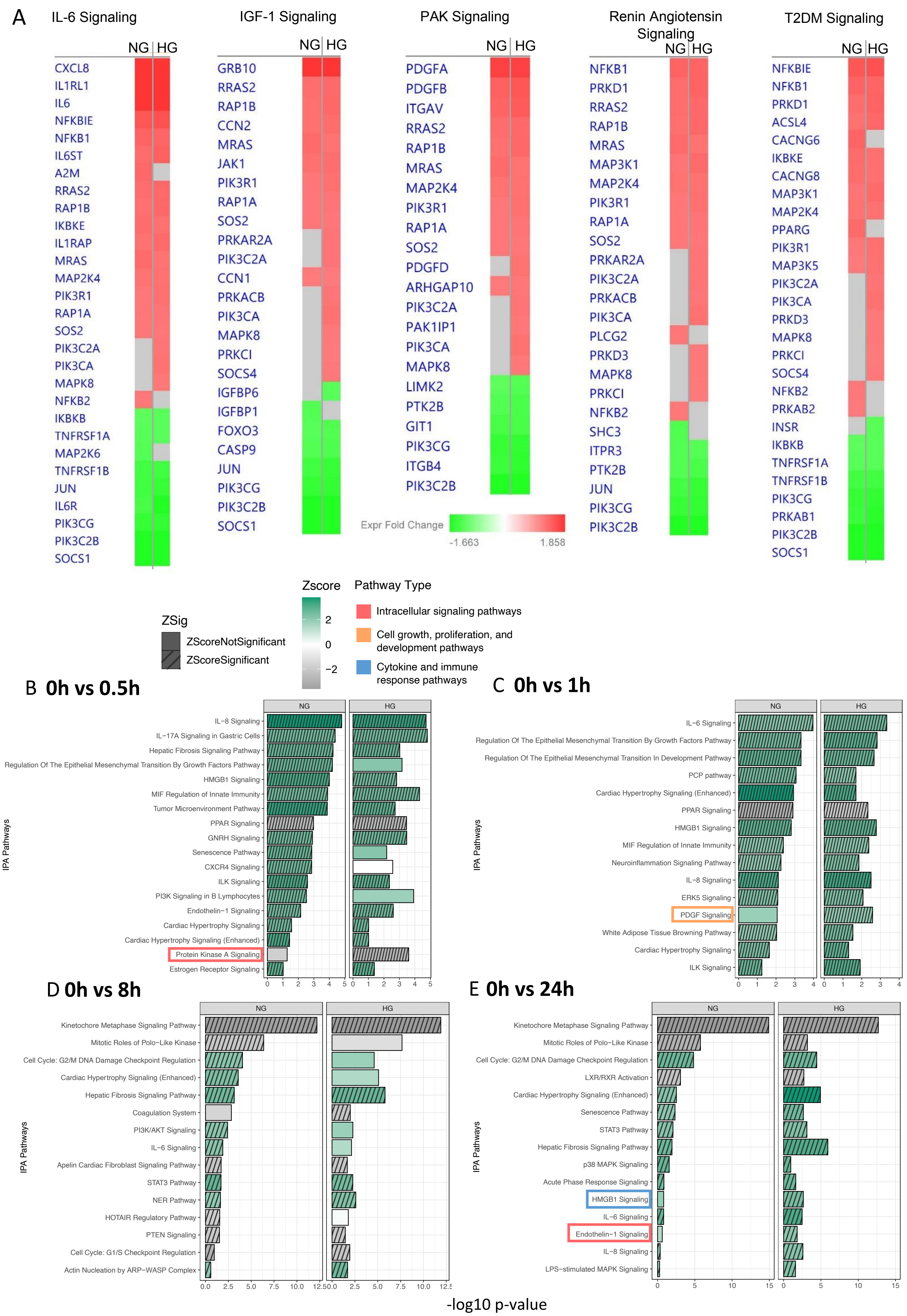
A
IL-6 Signaling
IGF-1 Signaling
PAK Signaling
Renin Angiotensin Signaling
T2DM Signaling
NG HG
CXCL8
IL1RL1
IL6
NFKBIE
NFKB1
IL6ST
A2M
RRAS2
RAP1B
IKBKE
IL1RAP
MRAS
MAP2K4
PIK3R1
RAP1A
SOS2
PIK3C2A
PIK3CA
MAPK8
NFKB2
IKBKB
TNFRSF1A
MAP2K6
TNFRSF1B
JUN
IL6R
PIK3CG
PIK3C2B
SOCS1
GRB10
CCN2
JAK1
PRKAR2A
CCN1
PRKACB
PRKCI
SOCS4
IGFBP6
IGFBP1
FOXO3
CASP9
PDGFA
PDGFB
ITGAV
PDGFD
ARHGAP10
PAK1IP1
LIMK2
PTK2B
GIT1
ITGB4
PRKD1
MAP3K1
PLCG2
PRKD3
SHC3
ITPR3
ACSL4
CACNG6
CACNG8
PPARG
MAP3K5
PRKAB2
INSR
PRKAB1
Expr Fold Change
-1.663
1.858
ZSig
ZScoreNotSignificant
ZScoreSignificant
Zscore
2
0
−2
Pathway Type
Intracellular signaling pathways
Cell growth, proliferation, and development pathways
Cytokine and immune response pathways
B 0h vs 0.5h
C 0h vs 1h
D 0h vs 8h
E 0h vs 24h
IPA Pathways
IL−8 Signaling
IL−17A Signaling in Gastric Cells
Hepatic Fibrosis Signaling Pathway
Regulation Of The Epithelial Mesenchymal Transition By Growth Factors Pathway
HMGB1 Signaling
MIF Regulation of Innate Immunity
Tumor Microenvironment Pathway
PPAR Signaling
GNRH Signaling
Senescence Pathway
CXCR4 Signaling
ILK Signaling
PI3K Signaling in B Lymphocytes
Endothelin−1 Signaling
Cardiac Hypertrophy Signaling
Cardiac Hypertrophy Signaling (Enhanced)
Protein Kinase A Signaling
Estrogen Receptor Signaling
IL−6 Signaling
Regulation Of The Epithelial Mesenchymal Transition In Development Pathway
PCP pathway
Neuroinflammation Signaling Pathway
ERK5 Signaling
PDGF Signaling
White Adipose Tissue Browning Pathway
Kinetochore Metaphase Signaling Pathway
Mitotic Roles of Polo−Like Kinase
Cell Cycle: G2/M DNA Damage Checkpoint Regulation
Coagulation System
PI3K/AKT Signaling
Apelin Cardiac Fibroblast Signaling Pathway
STAT3 Pathway
NER Pathway
HOTAIR Regulatory Pathway
PTEN Signaling
Cell Cycle: G1/S Checkpoint Regulation
Actin Nucleation by ARP−WASP Complex
LXR/RXR Activation
p38 MAPK Signaling
Acute Phase Response Signaling
LPS−stimulated MAPK Signaling
-log10 p-value

Supplemental figure 6. Heat maps of pathway DEGs and differential pathway activation/inhibition in HG compared to NG conditions.

(A) DEGs (±1.2 fold change, adjusted p-value <0.05 for significance) at the 0 h vs 4 h NG/HG pairwise comparison for each of the key significantly activated pathways as shown in figure 4. Pathway enrichment analysis of the DEGs (±1.2 fold change, adjusted p-value <0.05 for significance) determined at each of the pairwise comparisons B) 0 h vs 0.5 h NG/HG, C) 0 h vs 1 h NG/HG, D) 0 h vs 8 h NG/HG, and E) 0 h vs 24 h NG/HG using IPA. A right-tailed Fisher's Exact test was used to measure significance of the pathway enrichment in the gene set. The colour coding of bars indicates activation/inhibition determined by the z-score (generated using fold change and curated pathway relationships from the Ingenuity Knowledge Base), > or < 2 respectively. Hashed bars show significant z-scores. Signalling pathways of interest are highlighted by colour coded boxes.

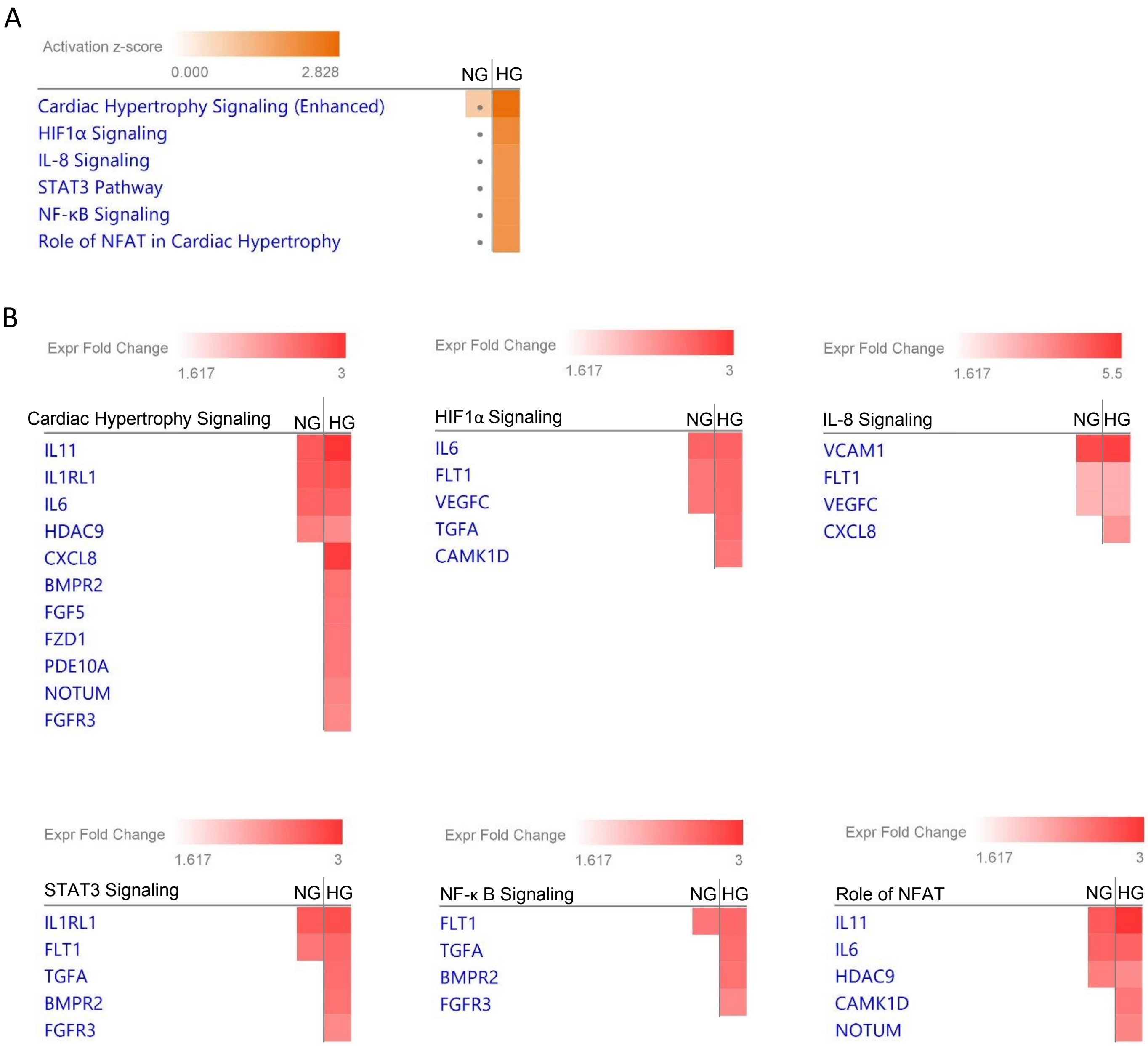

Supplemental figure 7. Heat maps determining differential pathway activation and DEGs of significantly activated pathways identified from the 'late' K-means cluster in HG compared to NG conditions. The heat map shows (A) pathway enrichment analysis of the DEGs (±1.2 fold change, adjusted p-value <0.05 for significance) determined at 0 h vs 4 h pairwise comparison in HG conditions from the 'late' cluster by K-means analysis. A right-tailed Fisher's Exact test was used to measure significance of the pathway enrichment in the gene set. The colour coding of bars indicates activation determined by the z-score (generated using fold change and curated pathway relationships from the Ingenuity Knowledge Base), >

or < 2 respectively. Dots show non-significant enrichment. (B) fold change of DEGs identified in each of the signaling pathways by IPA at 0 h vs 4 h pairwise comparison in HG conditions from the ‘late’ cluster by K-means analysis. FC values for NG genes is NA as they are not clustered in the ‘late’ category.

Supplementary Table 1a. Differentially expressed genes between 0hr and 0.5hr normal glucose samples

Ensembl gene IDs, Fold change (baseline-0.5NG) and FDR adjusted p-values of DEGs

Gene ID	FC 0NG - 0.5NG	adj p-value 0NG - 0.5NG
ENSG00000023902	1.2120792	0.00215907
ENSG00000057657	1.2997944	0.02205377
ENSG00000059728	1.20239721	0.01411433
ENSG00000062716	1.64100438	1.19E-06
ENSG00000067082	1.22995054	0.00033666
ENSG00000067191	-1.2323706	0.04393621
ENSG00000073756	5.17878301	1.19E-10
ENSG00000078401	1.53921048	3.92E-10
ENSG00000087074	1.2931216	1.84E-07
ENSG00000099860	2.20098441	5.34E-12
ENSG00000100650	1.20303593	0.00013987
ENSG00000100906	1.35597867	1.07E-05
ENSG00000101665	1.7067718	0.00032575
ENSG00000106089	1.24890736	0.04140602
ENSG00000108342	1.41330731	0.0048044
ENSG00000108551	1.68297832	1.59E-09
ENSG00000113070	1.76962521	3.08E-09
ENSG00000114315	4.4160313	9.68E-14
ENSG00000115738	2.15606829	1.02E-06
ENSG00000115963	1.2479917	0.00539222
ENSG00000116285	1.49514912	1.15E-07
ENSG00000117318	2.13076594	2.47E-16
ENSG00000117525	2.07338422	4.46E-09
ENSG00000118515	1.96156618	7.01E-23
ENSG00000118523	1.23387787	1.38E-07
ENSG00000119669	1.40892668	2.61E-06
ENSG00000120129	2.70721528	1.44E-17
ENSG00000120738	18.5920979	1.02E-19
ENSG00000122877	4.31911908	2.06E-13
ENSG00000123358	4.91306107	1.51E-14
ENSG00000124216	3.5641518	8.16E-20
ENSG00000124635	-1.2627063	0.03650594

Gene ID	FC 0NG - 0.5NG	adj p-value 0NG - 0.5NG
ENSG00000125740	3.79498024	1.03E-07
ENSG00000125968	4.02993246	2.70E-16
ENSG00000127528	1.52473548	1.02E-05
ENSG00000128016	5.02597791	1.79E-19
ENSG00000128594	2.10924666	2.18E-08
ENSG00000130513	1.35584763	4.51E-08
ENSG00000132510	1.37813613	5.35E-06
ENSG00000134107	1.42676707	2.03E-08
ENSG00000134668	1.23996733	0.01035041
ENSG00000135637	-1.2527313	0.04747172
ENSG00000136158	1.71566997	2.78E-10
ENSG00000136244	1.42955263	0.00470981
ENSG00000136826	2.67437675	4.96E-15
ENSG00000137834	2.50204847	4.81E-08
ENSG00000138166	2.27288012	1.71E-15
ENSG00000139318	1.33484274	6.21E-08
ENSG00000142178	1.30321504	0.0166887
ENSG00000142396	1.37623728	0.02651858
ENSG00000142871	1.45593723	5.03E-10
ENSG00000143384	1.26324803	1.45E-07
ENSG00000143878	1.28161733	0.00447807
ENSG00000144655	1.82934033	3.26E-14
ENSG00000144802	1.99154391	3.00E-06
ENSG00000148677	1.26148242	6.29E-05
ENSG00000148841	1.24556233	9.55E-07
ENSG00000153234	1.74545082	0.03950175
ENSG00000155090	2.15892791	3.25E-12
ENSG00000158050	1.54367575	6.61E-06
ENSG00000159200	1.46730941	0.03173965
ENSG00000160201	1.30589547	0.01203459
ENSG00000160888	2.49407085	1.32E-21
ENSG00000161835	1.30566567	0.00015651

Gene ID	FC 0NG - 0.5NG	adj p-value 0NG - 0.5NG
ENSG00000161960	1.31425404	0.02133212
ENSG00000162772	3.39010133	2.07E-07
ENSG00000163660	1.34726105	0.00019299
ENSG00000163739	1.59897562	3.11E-05
ENSG00000163874	1.51119889	9.20E-05
ENSG00000164683	2.30615209	1.20E-09
ENSG00000164736	1.23168329	0.00020832
ENSG00000167470	1.54814496	2.00E-12
ENSG00000167625	-1.23796	0.00040089
ENSG00000167840	-1.2297497	0.0410514
ENSG00000169429	2.10394175	9.97E-05
ENSG00000170345	10.4580351	4.77E-20
ENSG00000170889	1.24767304	0.00536104
ENSG00000171223	4.12643281	9.79E-26
ENSG00000173334	1.64311069	1.42E-06
ENSG00000175197	1.29447771	0.01943303
ENSG00000175592	1.2123749	0.01514503
ENSG00000175906	-1.220925	0.0309962
ENSG00000176907	3.05725218	2.54E-13
ENSG00000177606	1.45349836	9.14E-13
ENSG00000178184	1.2252376	0.01332288
ENSG00000178878	1.40233178	0.03998466
ENSG00000179388	8.52560425	1.52E-15
ENSG00000183598	-1.2600871	0.00416364
ENSG00000183691	1.67558102	2.04E-05
ENSG00000184557	2.19970192	1.93E-12
ENSG00000184678	-1.2112437	0.01863013
ENSG00000185022	1.31723526	2.11E-06
ENSG00000187479	1.37801336	0.0078176
ENSG00000187678	1.511588	2.07E-09
ENSG00000188295	1.25877751	0.01575509
ENSG00000188483	1.28828144	8.09E-05

Gene ID	FC 0NG - 0.5NG	adj p-value 0NG - 0.5NG
ENSG00000196449	1.21623624	5.87E-05
ENSG00000196866	-1.2233515	0.01605613
ENSG00000197019	1.49929813	2.77E-09
ENSG00000198435	1.53143659	3.21E-06
ENSG00000198517	1.36293368	2.57E-07
ENSG00000198551	-1.205337	0.01200883
ENSG00000198576	2.44608221	1.72E-07
ENSG00000198840	1.27163669	0.0367666
ENSG00000203804	1.48862543	0.00013924
ENSG00000204103	1.37269138	0.00104953
ENSG00000206573	1.4142279	0.00671322
ENSG00000207808	1.37258015	4.86E-05
ENSG00000207870	1.22943991	0.03031443
ENSG00000207980	1.55300707	0.00014046
ENSG00000212195	1.39898138	0.0001486
ENSG00000215417	1.39106286	0.01309257
ENSG00000218792	1.237898	0.00723889
ENSG00000219201	1.2280798	0.01589043
ENSG00000221883	1.26751849	0.01587313
ENSG00000224959	1.31826479	0.00448378
ENSG00000225630	1.24862586	0.04011
ENSG00000226380	1.48419175	0.00214525
ENSG00000228742	1.33062451	0.00585719
ENSG00000229372	-1.26481	0.04553694
ENSG00000231856	1.26404896	0.01242558
ENSG00000232034	-1.2295404	0.02557819
ENSG00000239948	1.25392752	0.00037183
ENSG00000241343	1.39454673	0.00195412
ENSG00000253669	1.36587078	0.0001268
ENSG00000255112	1.24615338	4.64E-06
ENSG00000255176	1.4657875	0.01686678
ENSG00000257298	1.28831055	0.00024002

Gene ID	FC 0NG - 0.5NG	adj p-value 0NG - 0.5NG
ENSG00000259943	1.24965515	0.02183713
ENSG00000260996	1.83445256	2.67E-08
ENSG00000261490	1.39737218	0.0166408
ENSG00000261889	1.20786113	0.01105304
ENSG00000262074	1.25531113	0.01915727
ENSG00000264175	1.20758734	0.03426498
ENSG00000264395	1.39409772	6.64E-08
ENSG00000265666	1.4058357	0.00091042
ENSG00000265972	-1.2815171	0.00116831
ENSG00000267519	2.99182926	6.09E-11
ENSG00000267598	2.04296354	8.69E-06
ENSG00000269609	-1.3208735	0.00177187
ENSG00000269688	1.22251085	0.02171694
ENSG00000269929	1.40044849	0.01787821
ENSG00000270640	1.27205355	0.01238798
ENSG00000273568	1.20484274	0.04111261
ENSG00000274322	1.21505035	0.03075793
ENSG00000274963	1.24840944	6.30E-05
ENSG00000275713	-1.2959015	0.02597654
ENSG00000275932	-1.2243237	0.00203475
ENSG00000275993	1.54994042	8.46E-06
ENSG00000276107	1.41524136	0.04450321
ENSG00000276230	1.2332374	0.04860953
ENSG00000276797	1.20571726	0.00029322
ENSG00000276852	1.24792279	0.00165413
ENSG00000277224	-1.2308835	0.04524814
ENSG00000278463	-1.2341952	0.03685999
ENSG00000278828	-1.2220745	0.04422186
ENSG00000279838	1.32860029	0.00280441
ENSG00000279891	1.21387761	0.03787827
ENSG00000280138	1.39896177	0.01826229
ENSG00000280302	1.22558345	0.00165903

Gene ID	FC 0NG - 0.5NG	adj p-value 0NG - 0.5NG
ENSG00000281881	1.80436367	3.84E-05

Supplementary Table 1b. Differentially expressed genes between 0hr and 1hr normal glucose samples

Ensembl gene IDs, Fold change (baseline-1NG) and FDR adjusted p-values of DEGs

Gene ID	FC 0NG - 1NG	adj p-value 0NG - 1NG
ENSG00000004660	1.266403905	0.012603265
ENSG00000005238	1.295926646	9.74E-08
ENSG00000005513	3.533425332	6.62E-16
ENSG00000006062	1.370476498	0.000437738
ENSG00000006327	1.306217489	2.16E-07
ENSG00000006459	1.534468587	4.61E-07
ENSG00000007944	-1.21670885	0.009911132
ENSG00000011422	1.20512099	0.003761542
ENSG00000015568	1.396336701	0.001674672
ENSG00000019144	1.389167227	2.10E-09
ENSG00000019549	1.703306152	3.15E-07
ENSG00000023445	1.964298654	5.49E-05
ENSG00000023902	1.306036076	1.69E-05
ENSG00000041988	-1.235798405	0.002676587
ENSG00000047346	1.279367697	0.024913552
ENSG00000049130	1.466525845	0.022410587
ENSG00000049883	-1.200467367	0.015630753
ENSG00000054598	1.83489809	2.65E-11
ENSG00000054967	1.832832908	6.99E-10
ENSG00000056558	1.532131584	0.037911364
ENSG00000059728	1.529333623	1.91E-08
ENSG00000062716	1.260465268	0.03435717
ENSG00000066923	-1.227839065	0.004609891
ENSG00000069399	1.505724592	4.16E-07
ENSG00000070404	1.267919055	0.001198596
ENSG00000070610	1.445037513	9.78E-05
ENSG00000071575	-1.200664383	0.043182732
ENSG00000073756	10.34147941	3.43E-15
ENSG00000075426	1.761901409	9.50E-15
ENSG00000078401	1.277487173	7.39E-05
ENSG00000079337	1.464232366	1.49E-05
ENSG00000080546	-1.221552177	0.009073605

Gene ID	FC 0NG - 1NG	adj p-value 0NG - 1NG
ENSG00000080947	-1.2643891	0.04524496
ENSG00000081386	-1.260744771	7.75E-05
ENSG00000083812	1.275630663	0.000175964
ENSG00000084093	1.410583584	4.21E-07
ENSG00000087074	1.257558294	2.08E-06
ENSG00000087494	1.734169759	4.48E-06
ENSG00000088826	1.365164768	1.16E-06
ENSG00000090339	1.407086275	0.016185721
ENSG00000095539	1.207336677	0.035975244
ENSG00000095739	1.399372736	0.00014942
ENSG00000095752	2.346362415	3.44E-10
ENSG00000096654	-1.501572933	2.55E-05
ENSG00000099860	3.422027773	4.94E-18
ENSG00000100147	-1.213215816	0.041587829
ENSG00000100276	1.575345235	2.93E-07
ENSG00000100292	1.334009888	6.87E-05
ENSG00000100311	1.572939768	9.54E-06
ENSG00000100650	1.227049103	2.62E-05
ENSG00000100906	2.155882579	3.05E-16
ENSG00000101384	1.543761552	1.37E-07
ENSG00000101665	10.41000605	3.72E-21
ENSG00000102890	1.550481278	0.000628065
ENSG00000102984	1.306392514	0.032500926
ENSG00000103241	1.384686844	0.005302605
ENSG00000103257	1.459860565	5.94E-10
ENSG00000103343	-1.262700625	0.001116936
ENSG00000104081	1.575418066	0.012285607
ENSG00000104221	-1.200447119	0.018298828
ENSG00000104419	1.318097474	1.20E-07
ENSG00000104856	1.226835428	0.049993037
ENSG00000104903	1.28455955	0.001403486
ENSG00000105643	1.23704337	0.044373859

Gene ID	FC 0NG - 1NG	adj p-value 0NG - 1NG
ENSG00000105656	1.207048922	0.001025843
ENSG00000105722	1.617745399	3.48E-11
ENSG00000106003	2.960723486	6.44E-13
ENSG00000106089	1.932126515	1.20E-09
ENSG00000106560	-1.200190408	0.031580702
ENSG00000106799	1.24846332	0.006296142
ENSG00000107104	1.260293958	2.00E-08
ENSG00000107562	1.386863701	0.000130936
ENSG00000107731	1.349610395	0.008581976
ENSG00000107807	1.706507077	9.25E-07
ENSG00000107821	2.014009522	2.62E-07
ENSG00000107968	1.449935536	0.005809908
ENSG00000108312	1.244882627	2.85E-06
ENSG00000108551	1.84260566	2.17E-11
ENSG00000108691	1.411798016	0.006232503
ENSG00000109118	-1.309552855	8.35E-08
ENSG00000109787	1.28160847	1.39E-07
ENSG00000110330	1.470725119	4.23E-06
ENSG00000110888	1.331668768	0.000255449
ENSG00000111276	-1.532128773	2.95E-09
ENSG00000111859	1.89331422	2.46E-09
ENSG00000112658	1.297038798	6.74E-11
ENSG00000112715	2.806369892	7.56E-15
ENSG00000113070	2.426703585	1.06E-14
ENSG00000113369	-1.226025482	0.015303848
ENSG00000113742	1.367604074	6.29E-07
ENSG00000113916	1.205285678	0.015022423
ENSG00000114019	1.67465688	3.79E-13
ENSG00000114315	7.368859721	7.72E-18
ENSG00000114529	1.6189437	4.74E-07
ENSG00000114739	-1.273083431	0.007688459
ENSG00000115520	1.206709601	0.040374224

Gene ID	FC 0NG - 1NG	adj p-value 0NG - 1NG
ENSG00000115548	1.479722241	5.20E-11
ENSG00000115738	5.335963423	7.17E-16
ENSG00000115844	2.772852395	5.74E-15
ENSG00000115963	1.294631365	0.000892902
ENSG00000116017	1.262182149	0.03143768
ENSG00000116205	-1.231669609	0.00413907
ENSG00000116285	2.444313503	1.49E-17
ENSG00000116604	1.333596493	3.45E-06
ENSG00000116679	1.274762388	4.27E-06
ENSG00000116717	-1.441536714	3.03E-09
ENSG00000116731	1.264876692	2.10E-07
ENSG00000117036	1.245904636	4.52E-07
ENSG00000117143	1.280438421	6.29E-07
ENSG00000117318	3.311571752	7.01E-23
ENSG00000117479	1.352009943	0.000537878
ENSG00000117525	2.286215843	1.76E-10
ENSG00000118263	1.456164737	1.91E-09
ENSG00000118503	1.520708738	0.000138869
ENSG00000118515	2.553638124	7.93E-28
ENSG00000118523	1.36696822	5.26E-12
ENSG00000119508	4.045008117	3.55E-13
ENSG00000119669	2.263152911	1.77E-16
ENSG00000120129	2.57977477	6.91E-17
ENSG00000120149	1.271262617	0.021881527
ENSG00000120217	1.230347866	0.003554248
ENSG00000120279	1.207010926	0.003714236
ENSG00000120616	1.233159644	0.000149772
ENSG00000120738	12.63940474	1.13E-17
ENSG00000120784	-1.357901264	0.000622646
ENSG00000121406	-1.232697894	0.014491248
ENSG00000121966	-1.397778677	1.05E-05
ENSG00000122386	-1.296364368	0.001584179

Gene ID	FC 0NG - 1NG	adj p-value 0NG - 1NG
ENSG00000122644	-1.361488074	0.000180538
ENSG00000122861	1.256227305	0.005742138
ENSG00000122863	1.850033717	5.92E-12
ENSG00000122877	4.818615841	2.20E-14
ENSG00000123095	1.451408497	0.001281856
ENSG00000123358	11.21008237	1.95E-20
ENSG00000124019	-1.417311042	1.26E-06
ENSG00000124216	7.066715107	3.44E-26
ENSG00000124575	-1.27460798	0.003599574
ENSG00000124610	-1.276484323	0.004164977
ENSG00000124762	1.417628114	8.94E-16
ENSG00000125266	1.396044065	0.000047139
ENSG00000125378	-1.370262976	0.000519504
ENSG00000125740	11.2455517	8.26E-15
ENSG00000125772	1.206254379	0.043103293
ENSG00000125812	1.438235647	2.73E-09
ENSG00000125845	1.296211837	0.004130473
ENSG00000125898	1.219728032	0.03081319
ENSG00000125945	-1.412016451	1.23E-07
ENSG00000125968	7.043779852	4.10E-21
ENSG00000126368	-1.372275213	0.000887386
ENSG00000126391	1.224573077	4.73E-06
ENSG00000126562	1.212118496	0.014553445
ENSG00000126603	1.27924731	0.001655793
ENSG00000126778	1.526035724	0.000034317
ENSG00000127080	1.620879116	6.64E-13
ENSG00000127528	1.666998877	2.09E-07
ENSG00000127533	1.274421158	0.018591504
ENSG00000128016	2.630134568	2.12E-12
ENSG00000128283	1.245608041	5.29E-06
ENSG00000128342	1.78832153	7.12E-08
ENSG00000128594	6.070342027	8.79E-20

Gene ID	FC 0NG - 1NG	adj p-value 0NG - 1NG
ENSG00000128917	1.345355439	0.011113342
ENSG00000129465	1.355158843	0.002861763
ENSG00000129467	1.302600433	0.000610338
ENSG00000129514	1.355936188	3.21E-05
ENSG00000129911	1.210538683	0.000739589
ENSG00000130164	1.238567558	0.001410821
ENSG00000130222	1.697684329	2.10E-07
ENSG00000130479	1.257215754	8.88E-07
ENSG00000130513	1.662803667	3.70E-14
ENSG00000130522	1.203948524	0.000888827
ENSG00000131016	1.208503019	5.45E-05
ENSG00000131634	1.263975371	0.000374093
ENSG00000131759	1.491933983	2.78E-06
ENSG00000132326	1.487633054	3.53E-05
ENSG00000132481	1.309326795	0.000688877
ENSG00000132510	3.059087861	2.10E-21
ENSG00000132801	-1.227987391	0.047338024
ENSG00000132825	-1.256689201	0.001536777
ENSG00000133561	-1.318709606	7.87E-08
ENSG00000133574	-1.254155731	0.007804386
ENSG00000134107	2.812849287	1.67E-22
ENSG00000134222	-1.361649985	0.001672472
ENSG00000134531	1.292991277	1.47E-14
ENSG00000134668	1.458895356	3.37E-06
ENSG00000135547	2.600858425	4.83E-10
ENSG00000135625	2.088792967	1.36E-13
ENSG00000135723	1.258679772	7.05E-07
ENSG00000136158	1.857265552	6.16E-12
ENSG00000136490	1.277330171	6.29E-05
ENSG00000136603	1.850869207	6.57E-11
ENSG00000136826	4.262427678	1.61E-20
ENSG00000137193	1.42065393	0.006316284

Gene ID	FC 0NG - 1NG	adj p-value 0NG - 1NG
ENSG00000137507	1.29384556	0.000402929
ENSG00000137834	6.959559096	2.15E-17
ENSG00000138166	3.313614539	7.44E-21
ENSG00000138623	2.167635767	6.01E-08
ENSG00000138675	1.531587206	1.45E-05
ENSG00000138835	1.553579956	1.24E-07
ENSG00000139263	-1.251077879	4.74E-05
ENSG00000139278	1.245001877	0.000317747
ENSG00000139517	1.232617582	0.000445723
ENSG00000140265	-1.216467695	0.008269926
ENSG00000140416	1.290601147	1.12E-09
ENSG00000140534	-1.355417096	0.001020628
ENSG00000141034	-1.301047163	9.21E-08
ENSG00000141526	1.427386915	6.15E-11
ENSG00000141582	1.365135984	0.000259949
ENSG00000141858	1.208020329	0.000505622
ENSG00000142178	1.888820965	1.14E-08
ENSG00000142279	1.507405849	6.61E-08
ENSG00000142396	1.455526507	0.006358037
ENSG00000142408	1.329630774	0.000590524
ENSG00000142556	1.217886203	0.001150997
ENSG00000142627	1.383827096	3.97E-09
ENSG00000142871	1.530970947	1.64E-11
ENSG00000143067	1.824153556	2.75E-10
ENSG00000143322	1.227678221	3.39E-06
ENSG00000143363	-1.280668761	0.002873356
ENSG00000143367	1.587159148	8.23E-08
ENSG00000143384	1.498862984	5.84E-14
ENSG00000143479	1.301441639	0.000909168
ENSG00000143751	1.225702712	0.000110338
ENSG00000143878	1.625258343	5.53E-08
ENSG00000144120	-1.206466144	0.020041823

Gene ID	FC 0NG - 1NG	adj p-value 0NG - 1NG
ENSG00000144136	1.298204532	4.88E-08
ENSG00000144560	1.264610662	5.03E-07
ENSG00000144655	3.598479579	5.00E-25
ENSG00000144802	1.431018605	0.021488124
ENSG00000145632	-2.289229915	2.15E-17
ENSG00000146232	2.10609638	4.90E-17
ENSG00000146676	1.287512871	0.004143103
ENSG00000148339	1.459745626	1.27E-06
ENSG00000148677	1.64718951	1.12E-12
ENSG00000148841	1.480636599	3.30E-13
ENSG00000148926	-1.616616558	1.51E-06
ENSG00000149289	1.354669573	9.83E-06
ENSG00000149591	1.357802588	0.00011404
ENSG00000150510	-1.21274026	0.009430249
ENSG00000150907	1.218584109	9.63E-06
ENSG00000151014	1.441030456	1.22E-11
ENSG00000151458	1.21110863	0.009239515
ENSG00000151748	1.38477943	2.94E-07
ENSG00000153094	1.305244654	0.034073329
ENSG00000153234	2.574281644	0.00011443
ENSG00000153885	1.427244614	3.06E-09
ENSG00000155090	3.505736226	6.60E-19
ENSG00000155760	1.28836517	6.25E-05
ENSG00000156030	1.438246747	1.37E-08
ENSG00000156273	1.74601738	4.24E-12
ENSG00000156509	-1.216761022	0.045568316
ENSG00000156966	1.309157638	0.008235227
ENSG00000156983	1.302285531	5.04E-08
ENSG00000157240	1.390889994	0.000498105
ENSG00000157514	-1.29161276	0.001602872
ENSG00000158050	1.615824957	9.04E-07
ENSG00000158615	1.225024721	3.11E-08

Gene ID	FC 0NG - 1NG	adj p-value 0NG - 1NG
ENSG00000158792	1.20246743	0.015022423
ENSG00000158805	1.243491523	0.001491935
ENSG00000158859	1.40018463	2.39E-06
ENSG00000159200	1.578553855	0.007100594
ENSG00000159640	1.221831332	0.033627311
ENSG00000160712	-1.221030558	0.048434566
ENSG00000160867	1.230265104	0.01960984
ENSG00000160888	2.825636301	2.05E-23
ENSG00000161835	2.123009047	1.37E-15
ENSG00000161940	1.485028239	6.79E-13
ENSG00000162063	-1.260892303	0.007233146
ENSG00000162241	1.26143979	0.043223373
ENSG00000162407	1.322658986	0.007743955
ENSG00000162413	1.573710558	2.96E-13
ENSG00000162545	1.200392292	0.024730948
ENSG00000162702	1.284086346	2.73E-05
ENSG00000162772	3.997446245	1.11E-08
ENSG00000163026	-1.243671458	0.000357476
ENSG00000163155	-1.402663337	0.001079278
ENSG00000163376	1.377141752	0.009259152
ENSG00000163462	-1.266379853	0.023704129
ENSG00000163545	2.284860081	1.81E-09
ENSG00000163635	1.215174897	9.80E-07
ENSG00000163659	1.491841195	2.21E-14
ENSG00000163660	1.396542346	3.07E-05
ENSG00000163739	1.398243042	0.003527958
ENSG00000163874	2.357209262	5.98E-12
ENSG00000164056	-2.070842012	2.39E-08
ENSG00000164683	8.350199905	3.14E-22
ENSG00000164741	1.223572916	1.61E-06
ENSG00000164743	-1.340997713	0.002111158
ENSG00000164855	1.362756385	0.000937857

Gene ID	FC 0NG - 1NG	adj p-value 0NG - 1NG
ENSG00000164933	1.220027939	0.004450572
ENSG00000165030	1.272073354	0.006092287
ENSG00000165507	-3.285559764	3.18E-12
ENSG00000165572	-1.223378609	0.000148823
ENSG00000165655	1.698459756	1.74E-07
ENSG00000166289	-1.581635818	3.11E-08
ENSG00000166401	1.33832614	2.64E-10
ENSG00000166483	1.557281747	1.34E-07
ENSG00000166592	1.473450166	8.90E-06
ENSG00000166860	1.221855085	0.000305385
ENSG00000166886	3.32280338	2.70E-19
ENSG00000167173	1.262527802	2.54E-05
ENSG00000167470	2.722596128	4.09E-24
ENSG00000167550	-1.271123862	0.00867796
ENSG00000167565	1.78125269	2.17E-13
ENSG00000167625	-1.333974466	2.46E-06
ENSG00000167657	1.28344653	1.33E-08
ENSG00000167772	2.112203012	6.64E-10
ENSG00000167840	-1.232924825	0.0377279
ENSG00000167874	1.36950453	0.000774464
ENSG00000168026	1.282632775	0.023599136
ENSG00000168209	1.696221042	6.48E-06
ENSG00000168386	1.481448369	6.26E-05
ENSG00000168672	-1.370317801	2.98E-05
ENSG00000168795	1.330243204	0.000392466
ENSG00000168874	2.303168902	1.52E-11
ENSG00000168994	1.331502793	2.04E-08
ENSG00000169016	1.237409635	0.001952538
ENSG00000169155	1.218511985	0.002158498
ENSG00000169242	1.450928486	0.00288519
ENSG00000169429	2.036713901	0.00019876
ENSG00000169914	1.30188357	0.000412575

Gene ID	FC 0NG - 1NG	adj p-value 0NG - 1NG
ENSG00000169926	1.429153827	3.66E-08
ENSG00000169991	1.387298937	6.33E-09
ENSG00000170345	3.186600937	1.61E-10
ENSG00000170525	1.415508085	1.87E-12
ENSG00000170775	1.321463516	1.03E-06
ENSG00000170852	1.465716066	1.15E-10
ENSG00000171115	-1.241069944	2.87E-05
ENSG00000171150	-1.23157979	0.007649954
ENSG00000171223	7.217511085	2.20E-30
ENSG00000171246	2.239333705	1.40E-09
ENSG00000171522	-1.231442504	0.000121314
ENSG00000171604	1.776257292	4.98E-07
ENSG00000171606	1.212940653	0.004635397
ENSG00000171617	1.577943244	2.32E-09
ENSG00000171621	1.532236007	0.000294511
ENSG00000171889	1.263123299	0.013677417
ENSG00000172059	1.520504429	1.04E-06
ENSG00000172216	1.263756409	0.000764626
ENSG00000172602	1.579018286	6.26E-05
ENSG00000173013	1.352474899	0.004940847
ENSG00000173276	1.32138749	1.54E-05
ENSG00000173281	1.462966383	5.08E-11
ENSG00000173334	1.402791789	0.00085961
ENSG00000173846	1.653685586	4.08E-10
ENSG00000175105	1.563476014	0.000565169
ENSG00000175505	1.336627948	0.003168977
ENSG00000175592	1.754097289	5.62E-11
ENSG00000175906	-1.35748679	0.000323422
ENSG00000176170	1.454345258	7.80E-09
ENSG00000176209	1.260559899	0.010043628
ENSG00000176692	2.060190469	3.06E-14
ENSG00000176749	1.340520196	0.001150273

Gene ID	FC 0NG - 1NG	adj p-value 0NG - 1NG
ENSG00000176842	1.40352398	0.00022807
ENSG00000176845	1.5314156	1.11E-06
ENSG00000176907	4.591797006	1.37E-17
ENSG00000177125	1.438234628	6.75E-08
ENSG00000177283	1.253256856	0.000612193
ENSG00000177303	1.231678079	3.94E-05
ENSG00000177426	-1.389802023	1.14E-08
ENSG00000177427	-1.265452031	0.001699827
ENSG00000177606	1.245874319	6.93E-07
ENSG00000177679	1.474991464	0.000136549
ENSG00000178726	1.224362215	0.004643836
ENSG00000178996	1.22882103	2.18E-05
ENSG00000179094	1.294773845	0.00011494
ENSG00000179144	-1.551443987	5.53E-11
ENSG00000179348	2.119303292	6.08E-13
ENSG00000179361	1.358057763	0.000225541
ENSG00000179388	16.11143996	3.34E-19
ENSG00000179403	1.57132249	0.000110164
ENSG00000179431	1.261674542	0.005485635
ENSG00000179604	1.21325317	0.00195303
ENSG00000179820	1.238319945	4.95E-08
ENSG00000179833	1.278040743	1.63E-06
ENSG00000179922	1.230599293	0.016947474
ENSG00000180035	1.209871228	0.006180474
ENSG00000180592	1.452804702	0.000165548
ENSG00000180730	1.4209606	3.18E-06
ENSG00000180884	-1.245385277	0.005763298
ENSG00000181007	-1.256472579	0.011587668
ENSG00000181274	-1.385713874	3.35E-05
ENSG00000181472	1.809251347	5.00E-10
ENSG00000181541	-1.311196438	0.004551272
ENSG00000181773	1.271120144	0.033147711

Gene ID	FC 0NG - 1NG	adj p-value 0NG - 1NG
ENSG00000182704	1.249574523	8.14E-05
ENSG00000183072	1.34444261	0.000174792
ENSG00000183161	-1.359248321	0.00031051
ENSG00000183307	1.525735536	3.13E-05
ENSG00000183337	2.301726777	4.91E-12
ENSG00000183691	3.978807549	1.46E-16
ENSG00000183935	1.245933147	0.001098088
ENSG00000184058	1.241398713	0.006159555
ENSG00000184307	1.70192548	4.87E-06
ENSG00000184402	1.317335422	1.27E-05
ENSG00000184557	3.238794013	6.91E-18
ENSG00000184635	1.334393973	0.047939321
ENSG00000184678	-1.204568292	0.023585369
ENSG00000184898	-1.214568331	0.038287651
ENSG00000184916	1.35471983	5.06E-07
ENSG00000185015	1.26456863	0.010068025
ENSG00000185022	1.712580769	7.26E-14
ENSG00000185215	1.296168283	0.000337813
ENSG00000185252	-1.202283061	0.016566736
ENSG00000185829	-1.250103052	0.04654483
ENSG00000186026	-1.255854223	0.043726131
ENSG00000186314	1.203986085	0.025699294
ENSG00000186480	1.449396718	7.98E-05
ENSG00000186603	1.230620716	0.025749299
ENSG00000186951	-1.246921857	0.003108836
ENSG00000187479	1.797652825	8.15E-07
ENSG00000187626	-1.251837825	0.022133189
ENSG00000187678	2.629233993	9.97E-21
ENSG00000188042	1.363243588	0.004649597
ENSG00000188064	1.214633617	7.02E-05
ENSG00000188171	-1.290519199	0.024552682
ENSG00000188290	1.308153008	0.002384489

Gene ID	FC 0NG - 1NG	adj p-value 0NG - 1NG
ENSG00000188295	1.423187607	8.70E-05
ENSG00000188483	2.984956916	3.02E-22
ENSG00000188522	1.733517905	2.86E-11
ENSG00000188997	-1.472880564	4.42E-07
ENSG00000189298	-1.405343816	0.000559077
ENSG00000196150	-1.264223107	0.002214959
ENSG00000196227	-1.329367994	0.002827848
ENSG00000196371	1.313997571	1.55E-07
ENSG00000196418	1.274630038	0.000973393
ENSG00000196428	1.496957194	3.09E-13
ENSG00000196449	1.593315089	4.36E-14
ENSG00000196456	-1.270660133	0.002386411
ENSG00000196458	-1.247380621	0.013388721
ENSG00000196544	-1.206846211	0.032116612
ENSG00000196670	-1.256601496	0.000389815
ENSG00000196756	1.266333308	0.005718439
ENSG00000196843	1.8308044	8.15E-11
ENSG00000196866	-1.23139437	0.012140249
ENSG00000197019	2.77065116	1.28E-21
ENSG00000197050	-1.369859479	0.000249235
ENSG00000197063	1.355951611	3.54E-09
ENSG00000197182	1.306564454	0.041639378
ENSG00000197183	1.327837108	5.45E-10
ENSG00000197329	1.358594446	3.40E-05
ENSG00000197380	1.362199624	0.012030975
ENSG00000197461	2.396033721	1.01E-16
ENSG00000197622	1.230965962	1.03E-10
ENSG00000197841	-1.284132504	0.04971784
ENSG00000197951	-1.286497888	0.000736401
ENSG00000197961	1.206077744	0.025054599
ENSG00000198105	-1.300399766	0.006051488
ENSG00000198142	1.468229796	1.50E-05

Gene ID	FC 0NG - 1NG	adj p-value 0NG - 1NG
ENSG00000198182	-1.396688676	6.89E-05
ENSG00000198324	1.221001514	0.000546577
ENSG00000198331	-1.236531561	0.004778317
ENSG00000198355	1.432307442	3.39E-08
ENSG00000198435	2.629718063	1.21E-15
ENSG00000198517	2.247089171	2.31E-19
ENSG00000198576	3.068798801	7.61E-10
ENSG00000198774	-1.46447865	0.000113387
ENSG00000198799	-1.266132542	0.005024837
ENSG00000198938	1.210251045	0.040941986
ENSG00000203709	-1.864775959	2.01E-06
ENSG00000203804	1.288503927	0.024326024
ENSG00000203883	1.658450888	7.57E-06
ENSG00000204103	1.453079248	9.91E-05
ENSG00000204442	1.245264234	0.013668122
ENSG00000204524	1.221837062	0.007854005
ENSG00000204611	-1.285068375	9.79E-06
ENSG00000204758	1.286404512	0.008547377
ENSG00000205710	1.261184025	0.035274064
ENSG00000206573	1.366262918	0.017625032
ENSG00000207808	1.30883154	0.000569374
ENSG00000207980	1.763105558	1.50E-06
ENSG00000213203	-1.454690464	6.62E-07
ENSG00000215271	-1.234928684	0.007862017
ENSG00000215417	1.348577305	0.029265521
ENSG00000215769	1.251632441	0.045085764
ENSG00000218891	1.239672495	0.008243029
ENSG00000221883	1.264385981	0.017356292
ENSG00000222009	1.261883024	0.006399995
ENSG00000222455	1.267496019	0.002851208
ENSG00000224959	1.335613884	0.002685094
ENSG00000225614	1.273585712	9.98E-06

Gene ID	FC 0NG - 1NG	adj p-value 0NG - 1NG
ENSG00000225630	1.242000115	0.047264989
ENSG00000227124	-1.240549392	0.037152673
ENSG00000228495	1.232284895	0.000243324
ENSG00000228768	1.251092458	0.013935113
ENSG00000229372	-1.287190597	0.027218294
ENSG00000229729	1.604237226	4.97E-07
ENSG00000230183	1.315942791	4.44E-05
ENSG00000231856	1.28819411	0.005884117
ENSG00000232034	-1.222867847	0.03126922
ENSG00000232956	1.222024008	0.00569416
ENSG00000234284	-1.295625951	0.006673322
ENSG00000235316	1.42990294	0.002549559
ENSG00000235831	1.259190851	0.002961543
ENSG00000237181	1.23890303	0.013923333
ENSG00000237989	1.253246984	0.00053851
ENSG00000240891	-1.277455837	0.01178121
ENSG00000241852	1.317707292	0.001557846
ENSG00000242829	1.541042996	1.67E-07
ENSG00000251493	1.548085849	7.58E-05
ENSG00000253368	1.418161379	4.81E-09
ENSG00000253522	1.212431481	0.014592073
ENSG00000253669	1.581822056	7.49E-08
ENSG00000253958	1.438494633	3.31E-05
ENSG00000254470	1.291394696	1.04E-07
ENSG00000254665	-1.251407204	0.010285382
ENSG00000255112	1.403326513	2.21E-10
ENSG00000255176	2.321268395	8.96E-08
ENSG00000256448	1.261880542	0.000014731
ENSG00000258137	1.204551907	0.00626534
ENSG00000259330	1.322139693	0.000601139
ENSG00000259863	1.234462463	0.037407519
ENSG00000259943	1.23456483	0.033446278

Gene ID	FC 0NG - 1NG	adj p-value 0NG - 1NG
ENSG00000260231	1.23333592	0.017549209
ENSG00000260428	1.261666874	0.008249213
ENSG00000260807	1.268316176	0.028775739
ENSG00000260992	1.251425833	0.001826316
ENSG00000260996	1.824249362	3.33E-08
ENSG00000262074	1.243982038	0.026421521
ENSG00000262601	1.304984103	0.01201425
ENSG00000264395	1.3325055	1.56E-06
ENSG00000264635	1.310913788	0.000809653
ENSG00000265666	1.416504955	0.000682994
ENSG00000265972	-1.270929258	0.00177998
ENSG00000265982	1.366764036	0.000390726
ENSG00000267041	1.323816561	0.005893625
ENSG00000267519	3.089591914	2.75E-11
ENSG00000267534	1.214188726	0.040849394
ENSG00000267598	2.184602306	1.51E-06
ENSG00000269318	1.2622137	0.038794355
ENSG00000269906	1.37006441	0.000052505
ENSG00000270194	1.206872702	0.025695588
ENSG00000271270	-1.329540839	0.002257127
ENSG00000272086	1.257303628	0.020423644
ENSG00000272574	1.435658421	3.98E-06
ENSG00000272768	1.270538328	0.011211162
ENSG00000273489	2.201583059	5.30E-09
ENSG00000273729	1.321458048	0.001722154
ENSG00000273820	1.344073895	0.002734544
ENSG00000273983	-1.230460519	0.047774098
ENSG00000274026	-1.264034833	0.0130733
ENSG00000274290	-1.202885805	0.041817925
ENSG00000274349	-1.293607194	0.012362631
ENSG00000274565	1.236096372	4.30E-05
ENSG00000274636	1.569831871	0.000441218

Gene ID	FC 0NG - 1NG	adj p-value 0NG - 1NG
ENSG00000274995	1.771353798	7.50E-06
ENSG00000275031	1.601691486	9.95E-07
ENSG00000275111	-1.338433449	0.000927997
ENSG00000275126	-1.228559973	0.038008458
ENSG00000275202	1.621942857	2.50E-06
ENSG00000275393	-1.218364953	0.046959976
ENSG00000275713	-1.268739489	0.047728604
ENSG00000275793	1.311309655	1.97E-05
ENSG00000275993	3.335051419	1.34E-17
ENSG00000277224	-1.259644409	0.020830152
ENSG00000277462	1.294789107	0.01126022
ENSG00000277639	-1.216905269	0.043703601
ENSG00000278970	-1.20311134	0.002499434
ENSG00000279637	1.222837405	1.99E-05
ENSG00000279692	1.267414217	0.016477726
ENSG00000280138	1.660709975	0.00014452
ENSG00000280143	1.40700946	0.000461331
ENSG00000280832	-1.36211815	0.003482529
ENSG00000281881	2.111442313	4.04E-07
ENSG00000282057	1.303849082	0.02167646

Supplementary Table 1c. Differentially expressed genes between 0hr and 4hr normal glucose samples

Ensembl gene IDs, Fold change (baseline-4NG) and FDR adjusted p-values of DEGs

Gene ID	FC 0NG - 4NG	adj p-value 0NG - 4NG
ENSG00000001167	1.396364368	1.61E-09
ENSG00000002587	1.498498594	1.22E-06
ENSG00000003400	-1.337442601	5.21E-08
ENSG00000003989	2.046114883	3.50E-09
ENSG00000004660	-1.225857458	0.040500872
ENSG00000004799	-1.329403563	0.00321364
ENSG00000005238	1.496597904	9.02E-13
ENSG00000005700	1.240690984	0.020070198
ENSG00000006016	1.254651056	0.006086321
ENSG00000006327	1.231142321	3.00E-05
ENSG00000006459	1.573072514	1.35E-07
ENSG00000006468	-1.295285023	0.002032922
ENSG00000006576	1.246109693	0.021225059
ENSG00000006607	1.216536302	2.95E-06
ENSG00000006638	1.241744837	0.017657583
ENSG00000006652	1.360357363	0.000268371
ENSG00000007384	-1.436643425	7.78E-06
ENSG00000007908	11.53047197	1.35E-15
ENSG00000007944	-1.382394926	9.97E-06
ENSG00000007968	-1.499634684	0.000642923
ENSG00000008083	-1.422609476	3.03E-06
ENSG00000008294	1.470706339	3.57E-17
ENSG00000008441	-1.263044986	4.19E-09
ENSG00000010292	-1.243397715	1.02E-06
ENSG00000010810	-1.314755359	2.65E-09
ENSG00000011201	1.239834716	0.006846852
ENSG00000011426	-1.254376646	0.00185473
ENSG00000011523	-1.231247554	5.28E-06
ENSG00000011638	-1.260741119	4.54E-05
ENSG00000012048	-1.331998998	0.000762141
ENSG00000012174	1.215899774	0.00705679
ENSG00000012660	1.226353402	2.63E-05

Gene ID	FC 0NG - 4NG	adj p-value 0NG - 4NG
ENSG00000013588	-1.304536414	0.000308848
ENSG00000013810	-1.367394261	0.000272038
ENSG00000014123	1.299719906	9.58E-06
ENSG00000014138	-1.278659337	3.20E-05
ENSG00000015133	-2.274284963	2.82E-16
ENSG00000015171	1.211238539	3.53E-05
ENSG00000015532	-1.223967101	1.14E-05
ENSG00000015568	1.801653737	8.45E-08
ENSG00000019144	1.70815672	1.44E-15
ENSG00000020577	1.445738363	1.03E-15
ENSG00000021762	-1.215897145	0.000328767
ENSG00000023445	2.1405202	6.54E-06
ENSG00000026559	1.268903424	0.01371352
ENSG00000026950	-1.288105171	3.94E-05
ENSG00000027869	1.322680629	0.018101858
ENSG00000028137	-1.4000567	6.78E-06
ENSG00000028277	-1.411384811	0.000183265
ENSG00000031081	1.41333413	3.21E-14
ENSG00000033327	-1.329193911	1.78E-10
ENSG00000034677	-1.203512812	0.034904183
ENSG00000035499	-1.395353927	3.42E-05
ENSG00000038295	1.458793988	5.82E-05
ENSG00000038382	1.231417695	8.84E-08
ENSG00000039319	1.245768884	0.030865249
ENSG00000040199	1.240918647	0.000150823
ENSG00000042088	-1.226342585	3.28E-06
ENSG00000042286	-1.212125878	0.01321621
ENSG00000043143	-2.227487792	9.19E-16
ENSG00000047230	-1.205201675	0.000720511
ENSG00000047617	-1.207646824	0.039290168
ENSG00000047621	1.299772022	0.000503321
ENSG00000047648	-1.284394146	0.015969759

Gene ID	FC 0NG - 4NG	adj p-value 0NG - 4NG
ENSG00000047932	1.336105121	0.000803408
ENSG00000048052	1.88790457	1.65E-06
ENSG00000048162	1.252996347	1.73E-05
ENSG00000049130	2.663856525	5.84E-09
ENSG00000049192	1.539623649	3.53E-11
ENSG00000049283	-1.940262729	3.78E-06
ENSG00000049759	1.747329048	6.08E-13
ENSG00000050820	-1.401959439	5.59E-11
ENSG00000051180	-1.325823675	0.004756304
ENSG00000051341	-1.317499834	0.002124687
ENSG00000052749	1.400233483	2.80E-10
ENSG00000052795	1.21626287	0.0016765
ENSG00000053524	1.348306218	0.00570257
ENSG00000053747	-1.348817543	7.76E-06
ENSG00000056558	2.949091953	3.93E-08
ENSG00000057019	1.369545603	7.60E-06
ENSG00000057252	1.246479834	0.004004207
ENSG00000057294	-1.653517988	1.18E-05
ENSG00000057704	-1.892662195	2.88E-16
ENSG00000058091	1.333181516	3.24E-06
ENSG00000058453	-1.233649117	0.006498854
ENSG00000059145	-1.217559135	0.000709131
ENSG00000059378	-1.203602102	0.001135463
ENSG00000060491	-1.209546551	0.000378105
ENSG00000062282	-1.332007123	0.008563017
ENSG00000062716	1.260445562	0.034375753
ENSG00000062822	-1.248649387	0.00048586
ENSG00000064225	1.431031091	0.000214264
ENSG00000064687	-1.307292783	0.014858524
ENSG00000064692	-2.012587279	1.89E-06
ENSG00000064989	1.585394674	3.14E-05
ENSG00000064999	-1.327579753	1.56E-05

Gene ID	FC 0NG - 4NG	adj p-value 0NG - 4NG
ENSG00000065054	1.277405062	0.000255926
ENSG00000065057	-1.301347178	2.41E-05
ENSG00000065183	1.207778175	2.13E-07
ENSG00000065308	-1.589847352	5.34E-15
ENSG00000065559	1.266796249	3.90E-05
ENSG00000066027	-1.237009935	0.004104196
ENSG00000066084	1.290979711	3.99E-06
ENSG00000066735	-1.540901948	1.21E-08
ENSG00000067064	1.292765964	0.020739383
ENSG00000067182	-1.235570325	2.54E-07
ENSG00000067798	1.773498048	1.62E-18
ENSG00000067955	1.991655709	2.24E-12
ENSG00000068024	-1.290525965	1.17E-06
ENSG00000068078	1.677172461	2.81E-09
ENSG00000068137	-1.514660457	6.53E-05
ENSG00000068305	1.480607559	1.49E-08
ENSG00000068366	1.27888172	6.40E-06
ENSG00000068489	-1.211953675	0.000235218
ENSG00000068724	-1.20692575	0.002300254
ENSG00000069702	-1.44266358	1.54E-05
ENSG00000069812	-1.209785155	0.000816675
ENSG00000070404	1.475362243	2.39E-07
ENSG00000070610	-1.510086248	1.43E-05
ENSG00000070614	-1.336263576	1.59E-10
ENSG00000070961	1.512444439	6.70E-07
ENSG00000071073	-1.263589004	9.42E-05
ENSG00000071205	1.204888545	0.000551276
ENSG00000071246	-1.585791224	6.36E-06
ENSG00000071575	-1.276638427	0.003358962
ENSG00000072110	1.207212061	5.83E-06
ENSG00000072201	-1.296869023	0.002667488
ENSG00000072422	1.310508883	1.60E-07

Gene ID	FC 0NG - 4NG	adj p-value 0NG - 4NG
ENSG00000072609	1.223208787	7.72E-07
ENSG00000072756	1.224700732	0.002329639
ENSG00000072840	-1.237660862	3.72E-05
ENSG00000073008	1.551774133	3.50E-15
ENSG00000073111	-1.281851351	0.000561441
ENSG00000073417	1.669148279	7.96E-17
ENSG00000073711	-1.284749082	0.001301095
ENSG00000073712	1.40424778	1.59E-12
ENSG00000073756	5.110277602	1.49E-10
ENSG00000073921	1.469444253	8.22E-08
ENSG00000074047	2.815924031	1.01E-15
ENSG00000074855	-1.276130228	0.004550611
ENSG00000075218	-1.376831495	0.000189351
ENSG00000075340	1.287823065	0.022149665
ENSG00000075643	-1.244939344	0.026861258
ENSG00000075651	-1.524859397	9.79E-11
ENSG00000075702	-1.478860294	0.000016821
ENSG00000075711	-1.307694523	4.02E-06
ENSG00000076351	-1.687828777	1.59E-09
ENSG00000076382	-1.252133104	0.000658055
ENSG00000076662	-1.264913929	0.013023704
ENSG00000077147	1.282286391	0.034451205
ENSG00000077150	1.218556392	0.003950105
ENSG00000077514	-1.297058505	8.34E-06
ENSG00000078043	1.211471759	0.00416996
ENSG00000078177	1.282806038	0.009815369
ENSG00000078687	-1.22931049	4.36E-05
ENSG00000078747	1.220823736	3.81E-05
ENSG00000078804	1.20487057	0.012459516
ENSG00000079102	1.357133892	0.001329933
ENSG00000079335	1.402897606	6.49E-07
ENSG00000079432	-1.236762306	5.75E-06

Gene ID	FC 0NG - 4NG	adj p-value 0NG - 4NG
ENSG00000079435	-1.245781194	0.0259684
ENSG00000079616	-1.357399582	1.16E-05
ENSG00000080561	-1.375812382	7.38E-09
ENSG00000080608	1.363252424	1.16E-09
ENSG00000080709	1.293578373	1.97E-05
ENSG00000080839	-1.234019727	0.031542759
ENSG00000080986	-1.36264833	0.000476088
ENSG00000081059	1.801135297	2.44E-08
ENSG00000081177	-1.214282363	4.36E-06
ENSG00000081320	-1.250689028	0.016148356
ENSG00000081665	1.297872982	0.004489831
ENSG00000081913	1.241399557	0.045916357
ENSG00000082014	1.39636632	0.001689038
ENSG00000082126	-1.229543164	0.000954295
ENSG00000082438	1.280606551	0.000116748
ENSG00000082512	-1.346456444	0.002311054
ENSG00000084093	1.662110877	2.78E-11
ENSG00000084710	1.78163804	1.29E-07
ENSG00000085185	-1.435144663	1.16E-05
ENSG00000085840	-1.34320194	0.005248276
ENSG00000085999	-1.286711762	0.034960318
ENSG00000086015	-1.241433874	0.000014261
ENSG00000086991	1.24315164	0.004300643
ENSG00000087095	1.344608259	2.10E-07
ENSG00000087157	-1.240672556	0.000747163
ENSG00000087266	-1.395225782	8.04E-08
ENSG00000087494	1.629466217	3.95E-05
ENSG00000087586	-1.229980742	0.002286716
ENSG00000087903	1.427637776	0.004291914
ENSG00000088179	-1.262718116	0.00091032
ENSG00000088325	-1.33400796	3.27E-05
ENSG00000088387	-1.358286596	8.67E-11

Gene ID	FC 0NG - 4NG	adj p-value 0NG - 4NG
ENSG00000088448	-1.236145808	0.013507749
ENSG00000088808	-1.228927836	0.000530427
ENSG00000088836	-1.223555371	0.005780182
ENSG00000088970	-1.283746956	0.01524709
ENSG00000088986	1.247929914	8.36E-10
ENSG00000089041	-1.921626036	1.53E-07
ENSG00000089060	-1.21218661	0.000310226
ENSG00000089123	1.21807467	0.013977604
ENSG00000089127	-1.434571589	0.02729229
ENSG00000089685	-1.211229304	0.002251543
ENSG00000089818	1.256245108	9.68E-07
ENSG00000089820	-1.223121519	0.039813867
ENSG00000090020	-1.20388998	0.00003534
ENSG00000090238	-1.290170018	0.000520198
ENSG00000090339	1.428843953	0.010972713
ENSG00000090376	-1.23421938	0.009120558
ENSG00000090530	1.311008337	0.00933204
ENSG00000090776	-1.242507063	5.05E-05
ENSG00000090889	-1.358546766	6.54E-05
ENSG00000091129	1.534374445	7.13E-11
ENSG00000091137	-1.302179382	0.004656839
ENSG00000091428	1.497219956	9.60E-07
ENSG00000091656	1.467228481	5.84E-07
ENSG00000091844	-1.395847218	0.00042216
ENSG00000091879	1.498510038	1.68E-10
ENSG00000091972	-1.286095975	0.006924001
ENSG00000092445	1.211498723	0.001170251
ENSG00000092470	-1.305154487	0.000797514
ENSG00000092621	-1.230122111	0.005781626
ENSG00000092820	-1.210182051	7.47E-08
ENSG00000092853	-1.365083367	0.001648195
ENSG00000092969	1.499834128	0.001578473

Gene ID	FC 0NG - 4NG	adj p-value 0NG - 4NG
ENSG00000093009	-1.325463427	0.009323758
ENSG00000094804	-1.274904957	0.002997527
ENSG00000094916	-1.226431713	7.20E-05
ENSG00000095015	1.248970626	0.000174325
ENSG00000095303	-1.214013256	0.000183713
ENSG00000095370	-1.503760726	5.10E-10
ENSG00000095383	-1.359678054	0.000281807
ENSG00000095397	1.361595759	9.81E-05
ENSG00000095637	-1.305241288	0.018466004
ENSG00000095713	-1.250295488	0.007317576
ENSG00000095752	2.416998566	1.38E-10
ENSG00000096433	-1.274888119	2.27E-08
ENSG00000097046	-1.268968206	0.006309402
ENSG00000099204	-1.362785384	2.46E-07
ENSG00000099246	1.27190514	0.010817761
ENSG00000099282	-1.279302306	0.001718317
ENSG00000099381	-1.205125605	0.001238773
ENSG00000099822	1.514174737	0.000548473
ENSG00000099875	-1.592950491	1.41E-12
ENSG00000099889	-1.686117604	1.63E-10
ENSG00000099910	-1.314143528	0.00036793
ENSG00000099998	-1.201854765	0.023450122
ENSG00000100034	-1.344257804	1.41E-10
ENSG00000100036	-1.529676755	2.88E-09
ENSG00000100060	-1.245830308	5.11E-06
ENSG00000100065	-1.436581972	2.81E-08
ENSG00000100147	-1.245011467	0.015883415
ENSG00000100276	1.247325309	0.014821134
ENSG00000100297	-1.21257813	0.000855675
ENSG00000100311	1.446509933	0.000280582
ENSG00000100350	-1.245412013	0.000447433
ENSG00000100368	1.264396458	0.000482136

Gene ID	FC 0NG - 4NG	adj p-value 0NG - 4NG
ENSG00000100439	-1.43887927	9.58E-06
ENSG00000100485	1.222390442	0.009739961
ENSG00000100490	-1.428227526	3.07E-05
ENSG00000100522	1.257798858	0.002845842
ENSG00000100523	1.235670551	0.004162818
ENSG00000100554	1.273597405	1.00E-08
ENSG00000100592	1.420570811	2.30E-11
ENSG00000100629	-1.363654599	0.001276598
ENSG00000100647	-1.201384613	6.05E-07
ENSG00000100890	-1.223052587	0.018321941
ENSG00000101003	-1.28940782	0.004322165
ENSG00000101019	-1.213739685	1.27E-05
ENSG00000101057	-1.216466929	0.020202019
ENSG00000101096	-1.43112368	2.28E-07
ENSG00000101187	-1.262997333	0.023891605
ENSG00000101188	-1.235268792	0.001293248
ENSG00000101224	-1.426401646	1.19E-08
ENSG00000101255	-1.473994778	1.63E-09
ENSG00000101265	-1.614713768	2.26E-10
ENSG00000101298	-1.263866455	1.48E-05
ENSG00000101384	1.582856255	3.78E-08
ENSG00000101412	-1.310238659	0.006508795
ENSG00000101445	-2.043642578	3.81E-14
ENSG00000101447	-1.411602325	1.47E-05
ENSG00000101558	1.206616882	0.000858253
ENSG00000101577	-1.328859727	9.78E-10
ENSG00000101665	2.514024676	7.75E-09
ENSG00000101670	2.434432155	7.18E-19
ENSG00000101868	-1.200462152	0.004148448
ENSG00000101871	-1.686112486	1.43E-14
ENSG00000101945	-1.292417431	0.002645388
ENSG00000102010	-1.678199931	2.77E-12

Gene ID	FC 0NG - 4NG	adj p-value 0NG - 4NG
ENSG00000102024	1.223926067	4.75E-06
ENSG00000102098	-1.263344977	0.007065305
ENSG00000102178	-1.254880177	1.56E-05
ENSG00000102384	-1.270649096	0.007301629
ENSG00000102531	1.534759957	1.03E-15
ENSG00000102554	1.31594366	0.000172157
ENSG00000102699	-1.266118535	6.14E-11
ENSG00000102755	2.001444118	1.47E-17
ENSG00000102935	1.851279573	3.60E-07
ENSG00000103111	-1.265128035	3.13E-09
ENSG00000103254	-1.207055345	0.037667794
ENSG00000103319	-1.305598179	2.54E-11
ENSG00000103365	-1.210515201	1.20E-05
ENSG00000103404	-1.275232363	2.60E-07
ENSG00000103522	1.243204378	1.58E-05
ENSG00000103888	1.306470259	0.000106273
ENSG00000104081	-1.547290549	0.017449588
ENSG00000104147	-1.263745347	0.024997017
ENSG00000104312	1.27545465	0.00011325
ENSG00000104365	-1.209995349	1.90E-05
ENSG00000104369	-1.2129278	0.002337389
ENSG00000104419	1.356045299	1.22E-08
ENSG00000104447	1.210855805	0.026453963
ENSG00000104626	-1.222597443	0.018076736
ENSG00000104643	1.262328467	0.000464548
ENSG00000104671	1.214847512	0.005603948
ENSG00000104695	1.224530811	8.77E-06
ENSG00000104856	1.623620473	4.40E-07
ENSG00000104863	1.238393474	0.013220791
ENSG00000104889	-1.22385818	0.02701855
ENSG00000104903	1.225697873	0.012812426
ENSG00000104951	1.604687127	4.96E-06

Gene ID	FC 0NG - 4NG	adj p-value 0NG - 4NG
ENSG00000105011	-1.274738917	0.001818392
ENSG00000105088	1.874439953	1.72E-11
ENSG00000105281	1.227514139	0.000152663
ENSG00000105287	-1.485738207	4.60E-11
ENSG00000105329	1.219534376	0.000045526
ENSG00000105486	-1.25021104	3.40E-06
ENSG00000105514	-1.314063924	1.74E-07
ENSG00000105662	-1.378487584	2.05E-05
ENSG00000105722	1.276806883	0.000104857
ENSG00000105738	-1.240685118	4.46E-06
ENSG00000105793	1.311118985	0.005176483
ENSG00000105810	1.859952228	7.27E-12
ENSG00000105825	1.331993195	1.00E-09
ENSG00000105851	-1.497919435	2.75E-09
ENSG00000105855	2.198556696	3.70E-13
ENSG00000105976	-1.243397168	2.68E-07
ENSG00000106070	2.416173667	1.33E-22
ENSG00000106100	-1.408733127	3.14E-08
ENSG00000106351	-1.785518161	8.51E-12
ENSG00000106366	1.314679394	1.22E-09
ENSG00000106462	-1.232007568	0.007628601
ENSG00000106537	1.422712456	1.15E-07
ENSG00000106560	-1.401561778	1.58E-05
ENSG00000106571	-1.489050491	3.49E-11
ENSG00000106688	-1.251882794	2.52E-07
ENSG00000106723	1.235547048	0.013998922
ENSG00000106799	1.277042867	0.002175601
ENSG00000106829	-1.248303012	3.34E-06
ENSG00000106852	-1.706450162	2.75E-08
ENSG00000106948	-1.376437535	6.48E-06
ENSG00000106993	1.233062724	0.004731724
ENSG00000107099	1.275422455	0.013011353

Gene ID	FC 0NG - 4NG	adj p-value 0NG - 4NG
ENSG00000107104	1.403095386	7.23E-13
ENSG00000107175	1.217885826	0.000642732
ENSG00000107185	-1.26928261	1.72E-06
ENSG00000107485	-1.470265903	1.04E-07
ENSG00000107551	-1.541491606	6.90E-05
ENSG00000107560	1.272574507	0.000954796
ENSG00000107562	4.234515165	3.91E-22
ENSG00000107679	1.330306797	2.00E-08
ENSG00000107719	-1.71374388	3.74E-09
ENSG00000107731	3.0862448	4.44E-15
ENSG00000107771	1.34212361	1.85E-05
ENSG00000107779	1.202729152	0.003532955
ENSG00000107789	1.226135985	0.004275743
ENSG00000107821	1.735145093	0.000028767
ENSG00000107863	1.518554561	4.20E-16
ENSG00000107937	1.20765039	3.68E-07
ENSG00000107957	-1.211103664	7.07E-08
ENSG00000107984	1.79646984	2.07E-10
ENSG00000108219	-1.312066927	4.59E-11
ENSG00000108262	-1.362502843	6.32E-10
ENSG00000108312	1.562838033	3.03E-14
ENSG00000108433	1.297285217	2.96E-06
ENSG00000108556	1.31301195	0.004103081
ENSG00000108622	-1.272749658	1.98E-09
ENSG00000108733	1.203931373	0.043211285
ENSG00000108799	-1.205869069	0.007524248
ENSG00000108846	-1.275186064	0.035002693
ENSG00000108960	1.609213744	1.48E-06
ENSG00000108984	-1.261560819	0.047521771
ENSG00000109062	-1.264136621	9.61E-07
ENSG00000109133	1.201149845	0.037087302
ENSG00000109189	1.313347157	9.79E-05

Gene ID	FC 0NG - 4NG	adj p-value 0NG - 4NG
ENSG00000109320	1.416566171	2.53E-16
ENSG00000109321	1.313601363	2.71E-05
ENSG00000109458	-1.321642041	0.000843781
ENSG00000109501	-1.513456409	1.26E-08
ENSG00000109618	1.237508405	0.027876971
ENSG00000109685	-1.281826133	3.91E-09
ENSG00000109756	1.258076517	5.67E-06
ENSG00000109805	-1.292251521	0.015210317
ENSG00000109814	1.202689885	3.52E-07
ENSG00000110047	-1.276679559	1.14E-06
ENSG00000110080	-1.333090584	1.53E-07
ENSG00000110318	1.376749699	0.003712842
ENSG00000110330	1.795930368	2.86E-10
ENSG00000110888	1.378255593	4.29E-05
ENSG00000110931	-1.272610326	2.55E-10
ENSG00000111057	-1.227567267	0.00027834
ENSG00000111077	-1.474659177	5.08E-10
ENSG00000111110	-1.556713358	0.002322614
ENSG00000111145	-1.274664182	1.28E-09
ENSG00000111199	1.234320309	0.03848507
ENSG00000111206	-1.228114908	3.95E-05
ENSG00000111247	-1.211760104	0.025239132
ENSG00000111331	-1.239420784	0.00031226
ENSG00000111335	-1.378282336	0.005144507
ENSG00000111339	1.464418278	9.59E-07
ENSG00000111445	-1.219591443	0.005203108
ENSG00000111490	1.289514837	0.000498156
ENSG00000111602	-1.25549519	9.45E-05
ENSG00000111647	1.401122524	0.001621776
ENSG00000111665	-1.329592607	0.00632164
ENSG00000111671	1.235516379	0.03903747
ENSG00000111725	-1.452186885	1.62E-07

Gene ID	FC 0NG - 4NG	adj p-value 0NG - 4NG
ENSG00000111801	-1.327130334	0.004275743
ENSG00000111817	1.303530876	3.98E-06
ENSG00000111846	-1.421657019	1.77E-08
ENSG00000111859	1.826130085	1.03E-08
ENSG00000111860	1.22378688	0.012173325
ENSG00000111907	1.339765516	0.001795191
ENSG00000111912	1.285557295	1.99E-06
ENSG00000111961	-1.686581427	6.48E-23
ENSG00000112033	1.316828947	6.47E-08
ENSG00000112137	-1.267772883	0.000240799
ENSG00000112208	1.282348741	0.005131546
ENSG00000112210	1.667389663	1.38E-11
ENSG00000112245	1.29356954	1.94E-05
ENSG00000112297	-1.813141542	1.73E-09
ENSG00000112305	1.296680068	2.64E-06
ENSG00000112414	-1.32053447	0.000497444
ENSG00000112541	1.924369077	4.42E-09
ENSG00000112559	1.593999353	2.77E-08
ENSG00000112742	-1.369401525	0.026564851
ENSG00000112773	2.153970039	2.07E-06
ENSG00000112837	1.248919661	0.001028643
ENSG00000112851	1.316658359	0.000660014
ENSG00000112972	1.312405293	0.01734894
ENSG00000112983	-1.20200424	0.000360488
ENSG00000112984	-1.327247232	1.66E-05
ENSG00000113161	1.256890855	0.007810549
ENSG00000113368	-1.332443263	3.66E-08
ENSG00000113369	1.429549852	7.95E-06
ENSG00000113384	1.211257125	2.57E-05
ENSG00000113532	1.766134108	1.19E-06
ENSG00000113552	-1.271060334	1.34E-09
ENSG00000113555	-1.820055121	1.44E-07

Gene ID	FC 0NG - 4NG	adj p-value 0NG - 4NG
ENSG00000113575	1.220782334	0.003644455
ENSG00000113580	1.858324124	1.63E-13
ENSG00000113594	1.203338117	0.019006408
ENSG00000113742	1.340400419	2.47E-06
ENSG00000113749	-1.353048826	0.01935706
ENSG00000113916	1.313878202	0.000167919
ENSG00000114125	1.219768269	2.68E-05
ENSG00000114166	-1.303453805	0.001475662
ENSG00000114315	-1.934862642	3.51E-05
ENSG00000114346	-1.257388117	0.003350708
ENSG00000114423	1.411627126	2.56E-06
ENSG00000114529	1.757098574	1.34E-08
ENSG00000114626	-1.409007306	0.002210474
ENSG00000114698	-1.235568499	0.007694594
ENSG00000114767	1.220707642	0.002620943
ENSG00000114770	-1.210932079	0.005495389
ENSG00000114812	-1.885020965	2.75E-06
ENSG00000115084	1.220403491	0.010352474
ENSG00000115091	1.219524342	4.12E-07
ENSG00000115163	-1.290809858	0.004121239
ENSG00000115170	1.30083634	6.17E-10
ENSG00000115183	-1.202984622	2.07E-05
ENSG00000115290	1.227731952	0.013573422
ENSG00000115368	1.242532827	0.000305161
ENSG00000115520	1.232061422	0.018237361
ENSG00000115525	-1.466830966	0.000240743
ENSG00000115540	1.261660056	0.009514604
ENSG00000115548	1.322177208	2.27E-07
ENSG00000115602	2.410268454	2.19E-08
ENSG00000115604	1.529764551	4.17E-06
ENSG00000115641	-1.412507906	3.00E-10
ENSG00000115738	1.91800321	2.40E-05

Gene ID	FC 0NG - 4NG	adj p-value 0NG - 4NG
ENSG00000115875	1.22666988	1.39E-07
ENSG00000115884	-1.239219059	0.027038365
ENSG00000115896	1.587380014	8.08E-05
ENSG00000115902	-1.40688422	1.46E-08
ENSG00000115946	1.369271185	6.92E-09
ENSG00000115963	1.223182002	0.013437763
ENSG00000116095	1.221114857	0.008986416
ENSG00000116106	-1.250858696	1.33E-05
ENSG00000116273	-1.269938369	3.91E-05
ENSG00000116473	1.225303404	5.61E-07
ENSG00000116514	-1.31861804	2.73E-07
ENSG00000116584	-1.437345157	2.90E-12
ENSG00000116641	1.382905463	7.17E-06
ENSG00000116704	1.254536003	0.000389043
ENSG00000116711	2.387326425	1.73E-10
ENSG00000116815	1.432123242	0.001044151
ENSG00000116991	-1.372892832	2.00E-06
ENSG00000117114	1.247643805	7.54E-07
ENSG00000117143	1.596510277	3.07E-14
ENSG00000117152	1.339169634	2.95E-05
ENSG00000117174	1.22617697	0.001651563
ENSG00000117318	1.69227337	1.72E-11
ENSG00000117399	-1.398787288	1.16E-05
ENSG00000117475	1.210225665	0.002869194
ENSG00000117479	1.258838969	0.011605744
ENSG00000117595	2.859389857	4.08E-11
ENSG00000117597	1.34823943	2.45E-09
ENSG00000117650	-1.359305359	0.000159624
ENSG00000117724	-1.416277623	7.20E-06
ENSG00000117758	1.224941726	9.38E-06
ENSG00000118058	-1.288928135	0.000220243
ENSG00000118193	-1.368115974	0.00025129

Gene ID	FC 0NG - 4NG	adj p-value 0NG - 4NG
ENSG00000118246	1.25432875	0.016116466
ENSG00000118257	-1.218761313	0.000289842
ENSG00000118263	1.333665846	8.94E-07
ENSG00000118402	1.646796637	3.26E-06
ENSG00000118407	-1.265824287	0.026143093
ENSG00000118496	1.231896739	0.006710608
ENSG00000118503	1.49197686	0.000278356
ENSG00000118508	1.219553474	1.32E-05
ENSG00000118515	1.683443215	3.40E-19
ENSG00000118523	1.292409446	1.17E-09
ENSG00000118689	-1.241905023	8.60E-05
ENSG00000118777	-1.243347915	0.02707837
ENSG00000118922	-1.308813003	0.000824101
ENSG00000118946	4.119788152	1.05E-10
ENSG00000118965	1.209804664	0.001278014
ENSG00000118971	-1.29037174	0.02994867
ENSG00000118985	1.795865207	5.03E-16
ENSG00000119138	1.210959705	0.001169234
ENSG00000119139	1.297869178	4.70E-08
ENSG00000119227	-1.219792683	0.026490254
ENSG00000119397	-1.213983464	0.001519629
ENSG00000119446	-1.236065712	0.00118514
ENSG00000119522	-1.226879605	0.000144454
ENSG00000119630	-1.613743224	7.07E-10
ENSG00000119661	1.205551229	0.022395915
ENSG00000119686	-1.246703235	0.010390041
ENSG00000119771	1.368412994	1.08E-09
ENSG00000119778	1.267510593	0.000482838
ENSG00000119801	1.212045839	0.026562189
ENSG00000119900	-1.238759218	0.012868085
ENSG00000119917	-1.322839536	0.017004395
ENSG00000119938	1.657430981	5.87E-07

Gene ID	FC 0NG - 4NG	adj p-value 0NG - 4NG
ENSG00000119965	1.348601584	0.000104674
ENSG00000120217	1.283188577	0.000343193
ENSG00000120278	-2.167811872	7.20E-20
ENSG00000120457	-1.270696665	0.001613428
ENSG00000120539	-1.232216941	0.008731109
ENSG00000120549	-1.264627075	0.021520824
ENSG00000120686	1.208214668	0.005791718
ENSG00000120693	3.456347063	2.90E-18
ENSG00000120705	1.220287968	7.09E-07
ENSG00000120742	1.200566042	0.04606351
ENSG00000120800	1.223639802	0.000362274
ENSG00000120802	-1.226939924	0.000204126
ENSG00000120875	-1.805606532	3.11E-18
ENSG00000120899	-1.269173409	0.005157554
ENSG00000121060	-1.265460289	2.97E-08
ENSG00000121152	-1.418497828	6.34E-05
ENSG00000121236	-1.372355772	0.004350632
ENSG00000121350	1.332726237	0.005677045
ENSG00000121644	1.213883721	0.02332217
ENSG00000121797	-1.304346372	0.000432775
ENSG00000121858	-2.187192729	6.77E-09
ENSG00000121957	-1.497913896	9.99E-08
ENSG00000121964	-1.245676019	0.002338635
ENSG00000121966	-2.469520762	3.41E-17
ENSG00000121988	-1.236749817	0.016125533
ENSG00000122068	1.367017136	1.42E-09
ENSG00000122386	-1.448487508	6.34E-06
ENSG00000122641	1.984567536	2.12E-11
ENSG00000122644	-1.32243452	0.000734981
ENSG00000122678	1.219601718	0.008077695
ENSG00000122786	1.304468994	1.18E-10
ENSG00000122861	-1.838678559	3.87E-11

Gene ID	FC 0NG - 4NG	adj p-value 0NG - 4NG
ENSG00000122863	1.575021245	1.73E-08
ENSG00000122870	1.483272157	7.81E-06
ENSG00000122912	1.218776703	0.001142798
ENSG00000122966	-1.36927507	5.98E-06
ENSG00000123066	1.264394704	4.10E-08
ENSG00000123080	-1.423507653	1.46E-06
ENSG00000123091	1.209335391	0.001871205
ENSG00000123094	1.314429658	2.24E-05
ENSG00000123095	1.427233412	0.002254512
ENSG00000123146	-1.288646981	0.001458304
ENSG00000123358	1.854614376	0.000122819
ENSG00000123384	-1.370333739	0.000340569
ENSG00000123473	-1.312704349	0.000151316
ENSG00000123485	-1.440536432	0.000849352
ENSG00000123545	1.331818263	0.00340623
ENSG00000123700	1.794434814	7.58E-05
ENSG00000123836	1.294605635	0.032503592
ENSG00000124006	-1.245255647	0.028991172
ENSG00000124019	-1.230167729	0.004151925
ENSG00000124120	1.231977375	4.51E-07
ENSG00000124212	1.382133127	0.026059775
ENSG00000124216	1.226134779	0.035398124
ENSG00000124225	1.678963262	5.25E-07
ENSG00000124440	-1.443837823	0.000592736
ENSG00000124575	-1.411863664	2.46E-05
ENSG00000124635	-1.285356942	0.020921878
ENSG00000124772	1.791069712	2.64E-08
ENSG00000125089	-1.342177677	4.63E-06
ENSG00000125257	1.202386966	0.00103857
ENSG00000125266	1.768157488	4.26E-10
ENSG00000125347	-1.40378304	0.001271845
ENSG00000125629	-1.286464895	0.025073224
ENSG00000125772	1.283471819	0.003479095
ENSG00000125845	1.693659411	1.95E-08
ENSG00000125864	-1.232026122	0.02816938
ENSG00000125871	-1.262459524	2.44E-05
ENSG00000125898	1.417290567	3.73E-05
ENSG00000125965	-1.311136748	0.003018823
ENSG00000125966	1.678162541	1.20E-06
ENSG00000125968	2.566743563	4.09E-11
ENSG00000126001	-1.252577991	7.17E-05
ENSG00000126351	-1.515149653	1.93E-11
ENSG00000126368	-1.288821885	0.010429393
ENSG00000126391	1.239300167	1.51E-06
ENSG00000126562	1.287570086	0.00072803
ENSG00000126705	-1.592974714	2.46E-08
ENSG00000126790	1.207369127	0.001211619
ENSG00000126822	-1.262888363	0.008728198
ENSG00000126878	-1.273197861	0.000325662
ENSG00000126882	-1.907329905	3.75E-08
ENSG00000126953	1.216278809	0.006509295
ENSG00000127080	1.510167689	6.25E-11
ENSG00000127124	1.435085219	3.03E-05
ENSG00000127220	-1.466226164	3.70E-05
ENSG00000127314	1.294982786	0.006966899
ENSG00000127328	-1.334638156	7.49E-08
ENSG00000127418	1.245879598	0.000634262
ENSG00000127511	-1.215263042	1.28E-06
ENSG00000127528	1.322254684	0.004075466
ENSG00000127533	-2.670012952	6.39E-15
ENSG00000127564	-1.288231184	0.012610603
ENSG00000127824	-1.25599496	0.005741344
ENSG00000128052	-1.264759686	3.42E-08
ENSG00000128203	-1.244701051	0.0227091
ENSG00000128266	-1.205554469	0.049990162
ENSG00000128284	-1.477228102	2.69E-06
ENSG00000128294	-1.201161794	1.53E-07
ENSG00000128309	-1.240662897	4.01E-06
ENSG00000128394	-1.32166786	0.002378893
ENSG00000128487	1.246354578	0.022229131
ENSG00000128567	1.525144038	1.02E-12
ENSG00000128590	1.304683264	0.003040459
ENSG00000128594	6.134623757	7.29E-20
ENSG00000128606	1.220301384	0.002254512
ENSG00000128641	-1.252757108	0.001531271
ENSG00000128805	-1.518869953	3.93E-13
ENSG00000128815	-1.417366495	5.25E-05
ENSG00000128833	-1.318424433	0.001954265
ENSG00000128849	-1.543135521	3.03E-11
ENSG00000128881	-1.210371032	0.008658695
ENSG00000128917	-3.912425793	1.70E-17
ENSG00000128923	-1.355955221	7.15E-06
ENSG00000128965	-1.300548324	0.044689496
ENSG00000129116	1.22964563	0.010253926
ENSG00000129173	-1.459797939	5.41E-06
ENSG00000129195	-1.227605735	0.015496961
ENSG00000129292	1.234681911	9.56E-06
ENSG00000129355	-1.343201412	0.002378202
ENSG00000129422	-1.630374755	6.29E-14
ENSG00000129474	-1.498832838	1.04E-10
ENSG00000129646	-1.262649479	0.025518291
ENSG00000129810	-1.394035445	0.000142599
ENSG00000130024	-1.21708105	0.004162726
ENSG00000130035	-1.301932206	0.032711052
ENSG00000130066	1.584013568	2.01E-07
ENSG00000130201	2.039628648	4.07E-11

Gene ID	FC 0NG - 4NG	adj p-value 0NG - 4NG
ENSG00000130270	-1.300856592	0.001028685
ENSG00000130300	1.524375111	0.002098689
ENSG00000130338	1.242878987	6.11E-05
ENSG00000130433	1.394176949	0.004273304
ENSG00000130449	-1.337125481	1.52E-14
ENSG00000130517	-1.531905858	2.07E-09
ENSG00000130544	1.314446502	0.000308647
ENSG00000130590	-1.4079368	0.000220526
ENSG00000130702	-1.475211398	2.36E-09
ENSG00000130706	1.238679332	1.88E-06
ENSG00000130751	1.26487169	0.000193081
ENSG00000130775	-1.216873607	0.020671519
ENSG00000130816	-1.242240572	9.09E-05
ENSG00000130956	1.562896343	1.36E-10
ENSG00000131015	1.248584489	0.024560889
ENSG00000131016	1.345651558	4.68E-09
ENSG00000131149	1.816393476	1.01E-11
ENSG00000131153	-1.276569424	0.003073245
ENSG00000131188	-1.274403998	0.010831495
ENSG00000131370	-1.774728071	2.15E-17
ENSG00000131378	1.344675466	1.03E-09
ENSG00000131386	-1.464721741	0.002041695
ENSG00000131409	3.372899709	1.73E-09
ENSG00000131435	1.235716473	0.000102071
ENSG00000131459	1.279826827	0.004191838
ENSG00000131634	1.491549034	1.25E-08
ENSG00000131669	-1.211021711	0.000443398
ENSG00000131724	1.325283584	1.60E-05
ENSG00000131732	1.207561643	0.002236449
ENSG00000131747	-1.407781356	0.000131201
ENSG00000131759	-1.36437914	0.000220657
ENSG00000131791	1.203293594	0.011358684

Gene ID	FC 0NG - 4NG	adj p-value 0NG - 4NG
ENSG00000131831	1.914992346	2.39E-10
ENSG00000131943	-1.208742736	0.001202313
ENSG00000131979	1.296088776	0.019800176
ENSG00000132003	-1.211501389	0.010487437
ENSG00000132024	-1.201067516	0.007244675
ENSG00000132170	1.353064662	0.00107219
ENSG00000132205	-1.36250102	0.000537878
ENSG00000132294	1.30928942	2.40E-05
ENSG00000132326	1.299042191	0.008758792
ENSG00000132432	1.21022227	0.039722964
ENSG00000132436	-1.486590489	5.52E-06
ENSG00000132470	-1.463961779	2.11E-05
ENSG00000132485	1.222348763	0.001885101
ENSG00000132563	-1.339516726	0.003527958
ENSG00000132589	-1.245403233	3.76E-06
ENSG00000132603	1.233011899	6.84E-06
ENSG00000132613	-1.416421117	2.30E-08
ENSG00000132622	-1.519302428	3.88E-07
ENSG00000132640	1.227640611	2.04E-05
ENSG00000132646	-1.209604406	0.000270836
ENSG00000132819	-1.280004615	4.06E-06
ENSG00000132906	-1.288847381	9.92E-05
ENSG00000133056	-1.756446479	8.96E-11
ENSG00000133401	-1.377427164	0.003871339
ENSG00000133561	-1.309663333	1.39E-07
ENSG00000133574	-1.369221334	0.000126026
ENSG00000133612	-1.349806265	8.36E-09
ENSG00000133657	2.314462155	7.75E-12
ENSG00000133773	1.254613892	0.000421776
ENSG00000133789	1.292659568	2.47E-08
ENSG00000133794	1.448908184	2.51E-07
ENSG00000133805	-1.403065807	0.000545747

Gene ID	FC 0NG - 4NG	adj p-value 0NG - 4NG
ENSG00000133816	1.215022837	7.40E-06
ENSG00000133818	1.318392203	6.91E-10
ENSG00000133874	1.348147723	8.05E-05
ENSG00000134057	-1.218209622	0.007873266
ENSG00000134107	-1.431346843	1.64E-08
ENSG00000134138	-1.332674596	5.32E-05
ENSG00000134222	-1.642185787	6.43E-07
ENSG00000134278	1.300179643	9.65E-11
ENSG00000134291	-1.261375718	0.000226172
ENSG00000134294	1.726185565	4.52E-06
ENSG00000134352	1.338587755	0.000374241
ENSG00000134363	1.763767393	9.88E-10
ENSG00000134371	1.228323421	1.47E-05
ENSG00000134375	1.253425748	0.000246687
ENSG00000134480	1.218770734	0.00236571
ENSG00000134508	-1.423490571	7.59E-06
ENSG00000134531	1.308664907	3.43E-15
ENSG00000134574	-1.210507473	0.000024598
ENSG00000134668	1.331524994	0.000361741
ENSG00000134690	-1.397753117	0.000119301
ENSG00000134769	1.231622834	0.001863762
ENSG00000134802	-1.315116435	1.25E-06
ENSG00000135069	-1.234111416	2.22E-06
ENSG00000135074	-1.388156131	2.12E-06
ENSG00000135083	-1.385473074	1.06E-05
ENSG00000135362	-1.331647469	1.76E-05
ENSG00000135365	-1.326477952	4.75E-07
ENSG00000135451	-1.338044439	0.007916935
ENSG00000135476	-1.554779664	0.000037134
ENSG00000135480	1.302998082	0.000394174
ENSG00000135547	3.338143855	7.04E-13
ENSG00000135604	-1.523732024	2.82E-09

Gene ID	FC 0NG - 4NG	adj p-value 0NG - 4NG
ENSG00000135723	1.228893824	6.37E-06
ENSG00000135919	1.225731552	0.000026039
ENSG00000135966	-1.97127446	8.58E-18
ENSG00000136044	1.428352138	4.81E-09
ENSG00000136108	-1.22968035	0.040466119
ENSG00000136114	-1.405908539	1.38E-07
ENSG00000136153	-1.233385209	7.95E-05
ENSG00000136158	1.200172183	0.027224642
ENSG00000136237	-1.486008049	6.50E-08
ENSG00000136244	2.278323951	1.18E-09
ENSG00000136381	1.212253036	0.012326144
ENSG00000136383	1.373019362	1.62E-06
ENSG00000136404	1.310004435	7.26E-05
ENSG00000136444	-1.206225847	0.000115415
ENSG00000136451	-1.267396864	1.52E-11
ENSG00000136490	1.52278232	5.82E-10
ENSG00000136574	-1.257256917	0.013917622
ENSG00000136603	1.231510017	0.017782358
ENSG00000136717	-1.357517607	9.18E-07
ENSG00000136770	1.326275208	1.35E-08
ENSG00000136826	1.365900331	0.001279054
ENSG00000136828	-1.270292491	0.027551737
ENSG00000136856	1.254266262	0.009927408
ENSG00000136859	-1.298961892	7.73E-07
ENSG00000136861	-1.282505842	2.36E-06
ENSG00000136869	1.367625341	3.27E-07
ENSG00000136870	1.209930633	0.001936406
ENSG00000136888	1.226242679	1.50E-06
ENSG00000137094	1.262275537	0.000133293
ENSG00000137135	-1.294015457	0.004309272
ENSG00000137177	1.557185885	7.76E-16
ENSG00000137203	1.413363901	2.17E-05

Gene ID	FC 0NG - 4NG	adj p-value 0NG - 4NG
ENSG00000137266	-1.323005483	0.000161844
ENSG00000137310	-1.32261535	0.001271591
ENSG00000137331	-1.296300406	0.000400388
ENSG00000137414	1.326748888	0.000308321
ENSG00000137463	-1.25666252	0.045293471
ENSG00000137486	-1.596949958	2.19E-12
ENSG00000137494	1.315021749	8.14E-06
ENSG00000137497	-1.225322909	4.64E-08
ENSG00000137502	1.377833468	0.000272503
ENSG00000137522	1.292389914	1.12E-05
ENSG00000137692	1.346998739	2.51E-09
ENSG00000137693	1.475044386	1.02E-06
ENSG00000137713	1.310429197	4.25E-09
ENSG00000137727	1.648328861	2.96E-08
ENSG00000137760	1.352517301	1.60E-05
ENSG00000137767	-1.222318787	0.028097152
ENSG00000137801	1.207839216	2.66E-05
ENSG00000137802	-1.288417809	1.14E-06
ENSG00000137804	-1.391580886	1.48E-05
ENSG00000137807	-1.373481276	0.000017133
ENSG00000137812	-1.321755406	0.012768514
ENSG00000137825	-1.232482215	0.019351426
ENSG00000137831	1.641624645	4.60E-09
ENSG00000137834	9.040173624	3.37E-19
ENSG00000137872	2.551816715	6.52E-14
ENSG00000137878	-1.342718786	0.004843542
ENSG00000137955	1.200821387	0.018238817
ENSG00000138018	1.380123829	0.000742431
ENSG00000138035	1.22169699	0.006641167
ENSG00000138134	-1.345091094	0.000844001
ENSG00000138135	1.202235969	0.000440146
ENSG00000138160	-1.263739168	0.02848296

Gene ID	FC 0NG - 4NG	adj p-value 0NG - 4NG
ENSG00000138162	-1.503163676	3.05E-07
ENSG00000138166	1.212380558	0.020144785
ENSG00000138180	-1.272768504	0.004213113
ENSG00000138347	-1.394326349	0.004668272
ENSG00000138376	-1.205155813	0.030042157
ENSG00000138411	1.508031524	2.36E-11
ENSG00000138434	-1.209922462	8.45E-06
ENSG00000138442	1.201207868	0.000204854
ENSG00000138448	1.397893101	5.91E-06
ENSG00000138449	1.261186704	0.001389845
ENSG00000138496	-1.32759384	0.001006784
ENSG00000138623	1.645821683	0.000248532
ENSG00000138639	1.41015897	7.70E-13
ENSG00000138650	1.428713548	9.13E-05
ENSG00000138675	1.921135279	1.25E-09
ENSG00000138678	-1.370241514	1.06E-05
ENSG00000138835	-1.210817899	0.027033047
ENSG00000139154	1.215880936	0.000430544
ENSG00000139163	1.209026447	0.024419545
ENSG00000139173	1.277674266	0.011460204
ENSG00000139174	-1.200001201	0.006686602
ENSG00000139278	1.46381288	6.06E-09
ENSG00000139289	-1.307329197	0.000160176
ENSG00000139354	-1.435412273	7.56E-06
ENSG00000139372	1.298064939	1.58E-05
ENSG00000139505	1.246072992	0.009688278
ENSG00000139618	-1.445960176	0.012248246
ENSG00000139687	1.202134525	0.034236098
ENSG00000139734	-1.209622975	0.013335151
ENSG00000139737	-1.362988176	0.020374788
ENSG00000139874	-1.314951971	0.024003411
ENSG00000139926	-1.449744638	4.37E-11

Gene ID	FC 0NG - 4NG	adj p-value 0NG - 4NG
ENSG00000140006	1.239601201	0.014592073
ENSG00000140009	-1.24288858	0.000846782
ENSG00000140280	1.2530375	0.02795456
ENSG00000140403	1.233880539	0.000093265
ENSG00000140416	1.547051022	1.34E-16
ENSG00000140450	1.299296553	0.008793808
ENSG00000140451	-1.448514579	0.00125625
ENSG00000140464	-1.23892137	3.52E-07
ENSG00000140471	1.21177582	0.029714243
ENSG00000140534	-1.462699686	3.66E-05
ENSG00000140564	1.377945165	2.80E-09
ENSG00000140598	-1.219952364	1.15E-05
ENSG00000140691	-1.232529619	0.001732505
ENSG00000140853	-1.279287669	0.003716021
ENSG00000140873	-1.328460235	0.0004924
ENSG00000141040	1.269903024	0.00246398
ENSG00000141068	-1.417956876	2.39E-08
ENSG00000141298	-1.2000812	0.000112245
ENSG00000141384	1.226206359	0.040320203
ENSG00000141401	-1.312691831	0.017288972
ENSG00000141441	1.392920879	0.000210133
ENSG00000141480	-1.21861576	0.018381138
ENSG00000141519	1.204416717	0.042866619
ENSG00000141526	1.418476628	9.98E-11
ENSG00000141560	-1.249919677	2.77E-05
ENSG00000141577	-1.205262168	0.002054066
ENSG00000141655	-1.404191559	2.08E-05
ENSG00000141664	-1.303221903	9.18E-07
ENSG00000141668	1.865578807	6.35E-05
ENSG00000141858	1.484813185	1.21E-10
ENSG00000142197	-1.209668063	0.000313214
ENSG00000142279	1.434693866	1.08E-06

Gene ID	FC 0NG - 4NG	adj p-value 0NG - 4NG
ENSG00000142408	1.327215718	0.000643986
ENSG00000142556	1.267639705	8.12E-05
ENSG00000142599	-1.213760205	3.88E-06
ENSG00000142627	-1.314909871	2.18E-07
ENSG00000142661	1.631901812	6.14E-06
ENSG00000142731	-1.253074735	0.038007415
ENSG00000142871	1.207411737	0.000539482
ENSG00000142945	-1.294138811	0.005557747
ENSG00000143153	1.612913783	5.13E-12
ENSG00000143228	-1.306928903	0.007634644
ENSG00000143322	1.25571988	3.95E-07
ENSG00000143341	1.327589507	0.011602987
ENSG00000143344	-1.34363716	1.00E-07
ENSG00000143369	-1.224627629	0.035956012
ENSG00000143375	-1.303546141	0.007040229
ENSG00000143409	-1.317125965	0.000735954
ENSG00000143434	-1.283498132	0.000480121
ENSG00000143469	1.309671624	0.036179553
ENSG00000143476	-1.322890818	0.004819882
ENSG00000143479	1.330494495	0.000302069
ENSG00000143622	1.372749601	2.97E-09
ENSG00000143643	-1.417248549	3.35E-07
ENSG00000143772	-2.262138581	4.62E-18
ENSG00000143801	-1.292751564	0.00044557
ENSG00000143816	-2.598197696	1.48E-10
ENSG00000143851	1.537445528	0.000603769
ENSG00000143878	1.388124673	0.000114877
ENSG00000144063	1.629130405	7.11E-12
ENSG00000144136	1.343851268	2.45E-09
ENSG00000144161	1.31097064	0.00078683
ENSG00000144320	1.234375061	0.002056195
ENSG00000144366	2.030992182	6.09E-13

Gene ID	FC 0NG - 4NG	adj p-value 0NG - 4NG
ENSG00000144560	-1.258669789	7.73E-07
ENSG00000144583	-1.224132414	8.80E-08
ENSG00000144644	1.666223424	1.45E-05
ENSG00000144645	-1.336278202	7.72E-10
ENSG00000144655	-1.248910355	0.000385764
ENSG00000144677	-1.441532792	1.73E-10
ENSG00000144712	-1.334420324	3.64E-05
ENSG00000144749	-1.771837812	4.39E-13
ENSG00000144802	1.577781068	0.001971929
ENSG00000144824	-1.519038192	2.87E-09
ENSG00000144893	1.269350215	0.000222464
ENSG00000144935	1.213254071	0.045985442
ENSG00000145014	-1.472974435	2.52E-10
ENSG00000145386	-1.223930979	0.005854711
ENSG00000145391	1.289365767	1.10E-08
ENSG00000145555	1.237638885	1.44E-06
ENSG00000145623	-1.219666196	0.000927163
ENSG00000145632	-1.236565746	0.002142046
ENSG00000145675	1.22490222	0.007924575
ENSG00000145723	1.2876731	0.009232425
ENSG00000145740	1.235380009	0.005156681
ENSG00000145817	1.74342134	1.11E-11
ENSG00000145860	1.240375371	1.09E-06
ENSG00000145911	-2.824479115	3.00E-17
ENSG00000145916	-1.480686934	3.25E-08
ENSG00000146021	-1.704726786	1.29E-09
ENSG00000146054	-1.30631735	3.66E-05
ENSG00000146094	-1.458900504	0.000974212
ENSG00000146232	1.53491715	7.66E-10
ENSG00000146242	1.239433791	2.24E-05
ENSG00000146374	1.892578021	8.39E-07
ENSG00000146386	1.317937617	7.67E-08

Gene ID	FC 0NG - 4NG	adj p-value 0NG - 4NG
ENSG00000146414	1.208672869	0.010048159
ENSG00000146670	-1.346427231	0.001071328
ENSG00000146676	1.266930311	0.008132277
ENSG00000146678	-1.280328581	0.004928675
ENSG00000146776	-1.208285125	0.009172683
ENSG00000146834	-1.459071274	5.43E-11
ENSG00000146918	-1.239440308	0.001144998
ENSG00000146950	-1.450614922	1.14E-09
ENSG00000147119	-1.442575227	8.22E-08
ENSG00000147130	-1.296716477	3.37E-06
ENSG00000147180	1.23618924	0.036665961
ENSG00000147526	-1.200675557	2.53E-10
ENSG00000147650	1.459206811	7.40E-08
ENSG00000147852	-1.453505069	0.000222606
ENSG00000147854	-1.240868872	9.89E-07
ENSG00000147862	-1.221050438	0.000155121
ENSG00000148082	-1.291196773	0.000154541
ENSG00000148400	-1.265430128	1.48E-06
ENSG00000148429	-1.265617335	1.05E-07
ENSG00000148468	1.337073636	4.36E-14
ENSG00000148660	-1.314986772	2.24E-08
ENSG00000148677	1.78107402	1.27E-14
ENSG00000148700	-1.206936343	0.00200664
ENSG00000148773	-1.523760088	4.97E-07
ENSG00000148848	1.328818678	0.023234379
ENSG00000148926	-2.698730901	7.03E-15
ENSG00000148943	1.239238272	0.001607496
ENSG00000149150	-1.233513414	0.023720365
ENSG00000149177	1.284716539	3.53E-06
ENSG00000149313	1.244488462	0.007252767
ENSG00000149503	-1.483326976	2.51E-07
ENSG00000149591	1.601191263	2.21E-08

Gene ID	FC 0NG - 4NG	adj p-value 0NG - 4NG
ENSG00000149636	-1.30941084	7.89E-06
ENSG00000149679	-1.228251741	0.000795416
ENSG00000149798	-1.247937494	1.00E-06
ENSG00000149929	-1.218570795	0.020723966
ENSG00000149948	1.490821005	6.02E-07
ENSG00000150347	2.06967299	1.01E-08
ENSG00000150456	-1.274945546	0.004193785
ENSG00000150457	-1.21586838	1.40E-05
ENSG00000150510	-1.219165969	0.007249737
ENSG00000150630	2.003470488	1.57E-15
ENSG00000150764	1.559898425	1.45E-08
ENSG00000150787	1.323022007	1.83E-05
ENSG00000151012	1.78483247	1.34E-08
ENSG00000151014	1.28551752	1.61E-07
ENSG00000151023	1.528101572	0.000193832
ENSG00000151093	1.26227195	0.011505312
ENSG00000151117	1.328662163	0.014985334
ENSG00000151136	2.5781483	4.58E-12
ENSG00000151151	1.459518362	0.002249396
ENSG00000151229	1.403121155	0.00084736
ENSG00000151247	1.38726454	1.14E-06
ENSG00000151414	1.442517559	5.61E-05
ENSG00000151458	1.348877277	2.35E-05
ENSG00000151491	-1.209814219	0.001210057
ENSG00000151503	-1.200976215	0.000902359
ENSG00000151689	-1.329686547	1.73E-07
ENSG00000151718	1.534067119	6.05E-18
ENSG00000151725	-1.317256787	0.000498701
ENSG00000151748	1.450724992	1.37E-08
ENSG00000151914	-1.253471234	0.033746748
ENSG00000151929	1.20903834	1.92E-05
ENSG00000151967	-1.514099703	3.01E-05

Gene ID	FC 0NG - 4NG	adj p-value 0NG - 4NG
ENSG00000152213	-1.217428583	0.003982024
ENSG00000152219	1.25030623	0.012425327
ENSG00000152229	1.322136697	0.000591558
ENSG00000152256	1.286526296	2.48E-05
ENSG00000152284	-1.564583464	1.27E-09
ENSG00000152413	1.245635152	0.01188017
ENSG00000152465	1.410207282	1.29E-09
ENSG00000152518	-1.390039403	6.36E-09
ENSG00000152558	1.248486166	0.002046782
ENSG00000152601	1.212850157	0.001262705
ENSG00000152689	1.921650293	1.60E-11
ENSG00000153048	-1.202138137	0.003719116
ENSG00000153071	1.234473199	3.46E-09
ENSG00000153094	-1.776125959	9.96E-07
ENSG00000153162	1.253504806	2.56E-09
ENSG00000153201	1.27636396	0.001708666
ENSG00000153208	-1.77335006	4.58E-11
ENSG00000153250	1.291887719	3.33E-06
ENSG00000153443	-1.226170848	0.011576794
ENSG00000153721	1.297304284	0.002089556
ENSG00000153814	1.647028872	4.69E-10
ENSG00000153885	1.400520513	1.16E-08
ENSG00000153989	1.22704566	0.018319442
ENSG00000154016	1.208498493	0.012129022
ENSG00000154122	1.50240626	2.49E-10
ENSG00000154127	1.47611765	4.59E-12
ENSG00000154134	-1.22538618	0.023931009
ENSG00000154217	1.351772142	0.000560613
ENSG00000154310	1.228529257	1.06E-06
ENSG00000154380	1.442084217	5.03E-15
ENSG00000154553	1.311457939	5.07E-05
ENSG00000154639	-1.436187169	0.001758515

Gene ID	FC 0NG - 4NG	adj p-value 0NG - 4NG
ENSG00000154734	2.777184231	2.00E-11
ENSG00000154864	-1.281989378	0.000015311
ENSG00000154920	-1.332535697	0.002188658
ENSG00000154930	-1.462767609	2.06E-05
ENSG00000155011	1.716492369	4.51E-07
ENSG00000155034	-1.243700124	0.000390221
ENSG00000155096	1.254134897	0.007605223
ENSG00000155111	-1.293681709	0.003850036
ENSG00000155307	1.405550352	0.000664226
ENSG00000155438	1.20275684	0.003850589
ENSG00000155465	-1.22505143	0.001101848
ENSG00000155868	1.210328098	0.012895674
ENSG00000155966	1.419527889	0.000124795
ENSG00000155970	1.296013156	0.00837747
ENSG00000155975	1.309751398	6.90E-07
ENSG00000156011	1.237187747	0.001842361
ENSG00000156030	-1.328452964	3.05E-06
ENSG00000156381	-1.234223218	0.000220751
ENSG00000156463	-1.382772502	0.00227568
ENSG00000156466	-1.591251685	8.69E-05
ENSG00000156509	-1.218556989	0.043319639
ENSG00000156671	1.270373447	0.000717018
ENSG00000156675	-1.633276353	5.42E-06
ENSG00000156876	-1.45529312	0.000296486
ENSG00000156970	-1.466562158	3.29E-06
ENSG00000157111	-1.408768198	1.76E-06
ENSG00000157168	1.4820629	3.54E-10
ENSG00000157181	1.22691256	0.031810074
ENSG00000157214	1.461154456	2.24E-05
ENSG00000157240	1.664726139	2.01E-07
ENSG00000157404	-4.98013595	4.07E-17
ENSG00000157456	-1.269007815	0.000399472

Gene ID	FC 0NG - 4NG	adj p-value 0NG - 4NG
ENSG00000157483	1.624020392	9.25E-17
ENSG00000157510	-1.509999697	2.60E-14
ENSG00000157554	-1.201668377	3.09E-08
ENSG00000157557	-1.552868118	1.08E-16
ENSG00000157613	-1.457454937	0.008406245
ENSG00000157617	-1.546457771	3.39E-12
ENSG00000157933	1.284289548	2.65E-10
ENSG00000158106	-1.385129318	0.009563129
ENSG00000158169	-1.309819533	4.97E-05
ENSG00000158186	1.341388895	0.00013754
ENSG00000158195	-1.414199819	1.87E-10
ENSG00000158352	1.368598366	6.84E-10
ENSG00000158373	-1.307618434	0.002499562
ENSG00000158402	-1.410257017	2.69E-05
ENSG00000158406	-1.315241474	0.00055562
ENSG00000158458	-1.317145294	0.001498328
ENSG00000158555	-1.317358915	3.28E-08
ENSG00000158716	1.39659444	1.37E-05
ENSG00000158769	-1.207264401	6.29E-05
ENSG00000158856	-1.223360417	0.001346172
ENSG00000158966	1.470925215	1.47E-08
ENSG00000159167	-1.745469681	1.16E-05
ENSG00000159231	-1.259186726	0.000125216
ENSG00000159388	-1.268396048	8.83E-06
ENSG00000159399	1.231926587	7.51E-06
ENSG00000159433	-1.394252806	6.61E-05
ENSG00000159733	-1.698220888	1.90E-06
ENSG00000159784	1.51262526	0.00074808
ENSG00000159788	-1.219439444	3.04E-07
ENSG00000159840	1.286337949	9.75E-08
ENSG00000159884	-1.323385559	0.0005873
ENSG00000160007	-1.309314012	3.95E-10

Gene ID	FC 0NG - 4NG	adj p-value 0NG - 4NG
ENSG00000160013	-1.96883302	1.31E-08
ENSG00000160179	-1.856244971	1.84E-09
ENSG00000160223	-1.265810606	0.001108575
ENSG00000160271	-1.369055811	3.32E-05
ENSG00000160298	-1.333930678	0.026383539
ENSG00000160299	-1.317711849	3.01E-07
ENSG00000160360	1.207829086	0.000762141
ENSG00000160447	-1.285598884	1.99E-06
ENSG00000160539	-1.381040233	0.004199889
ENSG00000160712	-1.377391264	0.000418167
ENSG00000160796	-1.480146204	1.14E-09
ENSG00000160813	-1.293369236	7.02E-05
ENSG00000160949	-1.356768109	0.000145458
ENSG00000160957	-1.294287405	0.045967697
ENSG00000161277	-1.2390292	0.032352786
ENSG00000161653	-1.510148596	3.79E-05
ENSG00000161714	-1.391051962	7.67E-07
ENSG00000161800	-1.299828971	0.000414127
ENSG00000161835	1.468903308	1.60E-07
ENSG00000161940	-1.387094172	1.49E-10
ENSG00000162063	-1.392532108	0.000070343
ENSG00000162066	-1.253414847	0.001783318
ENSG00000162367	-1.239530762	4.47E-08
ENSG00000162390	1.557582245	9.56E-07
ENSG00000162407	2.45182868	4.55E-13
ENSG00000162434	1.288609361	1.01E-09
ENSG00000162437	1.340223365	2.60E-05
ENSG00000162496	-1.788272672	2.52E-09
ENSG00000162522	-1.983275584	1.25E-17
ENSG00000162551	1.243523057	0.014763399
ENSG00000162614	1.426102507	2.06E-06
ENSG00000162623	1.201690788	0.007020691

Gene ID	FC 0NG - 4NG	adj p-value 0NG - 4NG
ENSG00000162636	1.357293069	0.000479786
ENSG00000162645	1.264858376	0.017950812
ENSG00000162692	4.845028798	2.53E-18
ENSG00000162695	1.354511037	0.001474048
ENSG00000162746	1.452938921	6.21E-05
ENSG00000162757	1.262479146	0.014302551
ENSG00000162775	-1.225728973	2.10E-05
ENSG00000162783	-1.304039961	7.48E-06
ENSG00000162817	-1.460028675	3.48E-09
ENSG00000162851	1.23088542	7.68E-05
ENSG00000162869	-1.207908292	0.001393608
ENSG00000162944	-1.358426122	0.026344386
ENSG00000163050	-1.414090556	0.000297877
ENSG00000163083	-1.298441572	0.0158239
ENSG00000163110	1.532428803	2.23E-17
ENSG00000163162	-1.201987187	0.000155055
ENSG00000163171	-2.094208359	2.60E-18
ENSG00000163219	-1.608211288	0.001632027
ENSG00000163235	1.879002972	1.86E-08
ENSG00000163251	-1.552282513	6.83E-06
ENSG00000163291	1.382484725	2.05E-05
ENSG00000163462	-1.239255335	0.047400959
ENSG00000163491	-1.340772345	0.025657927
ENSG00000163577	1.285746261	0.015805194
ENSG00000163637	1.317901673	0.000352157
ENSG00000163638	-1.439524933	6.74E-07
ENSG00000163659	-1.248747983	1.73E-07
ENSG00000163734	1.346110143	0.000147808
ENSG00000163820	-1.219171409	1.78E-06
ENSG00000163840	-1.386204421	1.60E-05
ENSG00000163918	-1.305976713	0.003115402
ENSG00000163935	1.524708164	4.55E-05

Gene ID	FC 0NG - 4NG	adj p-value 0NG - 4NG
ENSG00000163947	-1.353271667	6.64E-06
ENSG00000164022	1.243799069	9.46E-06
ENSG00000164023	1.229405753	0.005723434
ENSG00000164024	1.20305583	5.13E-05
ENSG00000164038	1.240023387	1.51E-05
ENSG00000164070	-1.223911788	0.004023796
ENSG00000164087	-1.338146001	0.000587704
ENSG00000164104	-1.421546599	3.61E-06
ENSG00000164125	1.241944787	0.011466074
ENSG00000164134	1.218873289	0.023090867
ENSG00000164161	1.214426847	0.000244214
ENSG00000164197	1.247577671	0.029706837
ENSG00000164211	1.369028429	0.015501723
ENSG00000164283	1.878335498	3.20E-12
ENSG00000164292	1.465571651	2.25E-09
ENSG00000164306	-1.245364291	0.001880443
ENSG00000164338	1.30652716	1.41E-06
ENSG00000164442	1.260231008	0.024022295
ENSG00000164484	1.220014097	0.00112466
ENSG00000164506	1.233068126	0.000307946
ENSG00000164512	2.00178501	6.02E-09
ENSG00000164532	1.283330534	0.002455486
ENSG00000164649	-1.372813357	2.60E-13
ENSG00000164683	1.606880635	0.000146803
ENSG00000164684	2.302924696	1.77E-13
ENSG00000164736	1.557950966	1.37E-11
ENSG00000164823	1.712000122	8.90E-11
ENSG00000164849	-1.690111215	0.000735538
ENSG00000164867	-1.39929357	2.03E-07
ENSG00000164885	-1.209276419	0.033231276
ENSG00000164897	-1.245762732	0.000130499
ENSG00000164933	1.287968256	0.00020395

Gene ID	FC 0NG - 4NG	adj p-value 0NG - 4NG
ENSG00000164976	-1.258475301	5.48E-05
ENSG00000165030	1.360472201	0.000293915
ENSG00000165046	1.231446882	0.018245145
ENSG00000165138	-1.292300625	1.04E-08
ENSG00000165233	-1.241065818	0.000126075
ENSG00000165259	1.380044978	0.000219183
ENSG00000165271	1.326733795	2.97E-08
ENSG00000165272	-1.2732056	0.01621143
ENSG00000165304	-1.342466055	1.74E-06
ENSG00000165338	1.551641126	7.43E-05
ENSG00000165424	-1.253433602	0.002007537
ENSG00000165434	1.284492464	0.001737474
ENSG00000165480	-1.319766735	0.006357681
ENSG00000165507	-1.553253085	0.00251117
ENSG00000165644	-1.25746116	0.004854078
ENSG00000165732	1.398137546	5.12E-09
ENSG00000165757	-1.467863716	2.19E-17
ENSG00000165801	1.552609954	8.33E-06
ENSG00000165806	-1.337208381	2.87E-13
ENSG00000165832	1.227507181	0.001210826
ENSG00000165886	-1.453537893	2.05E-10
ENSG00000165891	1.361594624	3.28E-06
ENSG00000165895	1.488786585	1.40E-05
ENSG00000165912	-1.203131278	0.00980195
ENSG00000165959	-1.591256255	2.61E-10
ENSG00000166068	1.265211392	0.000070343
ENSG00000166073	1.310196779	6.63E-06
ENSG00000166140	-1.270465871	0.000917002
ENSG00000166250	1.249000389	0.016369321
ENSG00000166289	-1.560935017	6.18E-08
ENSG00000166398	1.391600965	2.29E-08
ENSG00000166401	1.465358397	1.08E-13

Gene ID	FC 0NG - 4NG	adj p-value 0NG - 4NG
ENSG00000166439	-1.403137747	5.61E-11
ENSG00000166446	1.323845242	7.18E-10
ENSG00000166471	1.410813551	6.12E-06
ENSG00000166503	1.317754613	3.22E-06
ENSG00000166510	1.912491752	2.38E-07
ENSG00000166532	1.207586659	0.039375397
ENSG00000166592	1.229680603	0.027520555
ENSG00000166770	1.22519602	0.035379816
ENSG00000166801	-1.422209045	4.80E-06
ENSG00000166831	1.207460881	0.024513556
ENSG00000166851	-1.362458052	0.000374507
ENSG00000166886	1.521932201	1.85E-06
ENSG00000166900	1.304706659	9.42E-09
ENSG00000166924	-1.509339543	7.16E-05
ENSG00000166925	-1.625166998	3.86E-12
ENSG00000166938	-1.311899549	5.99E-07
ENSG00000166949	-1.327182288	7.28E-11
ENSG00000166971	-1.350239869	4.02E-07
ENSG00000167077	-1.23842529	0.018041979
ENSG00000167081	1.240607209	3.00E-05
ENSG00000167106	-1.749382387	4.09E-12
ENSG00000167191	-1.820184531	2.91E-09
ENSG00000167202	-1.301629337	3.42E-09
ENSG00000167363	-1.478385701	1.16E-05
ENSG00000167378	1.214590165	3.24E-07
ENSG00000167513	-1.391697931	0.000774521
ENSG00000167536	-1.301984257	0.019529836
ENSG00000167550	-1.47554771	1.07E-05
ENSG00000167554	1.334391519	0.00151553
ENSG00000167562	1.275502206	0.00342842
ENSG00000167600	-1.459255934	0.000283027
ENSG00000167635	1.212372388	0.001409461

Gene ID	FC 0NG - 4NG	adj p-value 0NG - 4NG
ENSG00000167670	-1.398142155	0.000275077
ENSG00000167680	-1.478160634	3.75E-10
ENSG00000167799	-1.201363543	0.014564224
ENSG00000167861	-1.369739716	1.65E-05
ENSG00000167895	-1.338918435	0.007757859
ENSG00000167972	-1.280912662	5.59E-07
ENSG00000168016	-1.365642994	9.14E-06
ENSG00000168061	-1.278756445	0.002062689
ENSG00000168209	-1.666602256	1.21E-05
ENSG00000168298	-1.299393958	0.00030934
ENSG00000168309	-1.426874451	4.00E-07
ENSG00000168310	-1.318174905	1.61E-07
ENSG00000168374	1.230336477	6.07E-07
ENSG00000168386	1.923951447	1.28E-09
ENSG00000168461	1.534144854	3.34E-13
ENSG00000168476	-1.206742682	0.000795736
ENSG00000168497	-2.003824661	2.25E-18
ENSG00000168575	1.420240193	2.32E-10
ENSG00000168672	-1.601399049	6.30E-09
ENSG00000168675	1.297719175	0.008030919
ENSG00000168685	1.741557421	0.000269393
ENSG00000168763	-1.351342422	0.000041882
ENSG00000168769	1.286542981	1.74E-08
ENSG00000168792	-1.320121493	3.14E-05
ENSG00000168795	1.205115542	0.033954067
ENSG00000168874	2.048857589	9.13E-10
ENSG00000169184	-1.576495448	7.25E-05
ENSG00000169220	-1.309178512	0.004700773
ENSG00000169242	-2.030615394	4.20E-08
ENSG00000169247	-1.420917981	0.001117882
ENSG00000169255	-1.368848513	2.53E-05
ENSG00000169429	2.644009813	7.07E-07

Gene ID	FC 0NG - 4NG	adj p-value 0NG - 4NG
ENSG00000169504	1.24265536	0.020072019
ENSG00000169554	1.329303029	2.13E-10
ENSG00000169583	1.317624153	0.049732292
ENSG00000169594	-1.28854468	2.95E-08
ENSG00000169607	-1.383701585	0.000654754
ENSG00000169629	1.309746695	0.004613081
ENSG00000169679	-1.314113431	0.000319232
ENSG00000169683	-1.303331198	3.71E-05
ENSG00000169756	1.308649253	0.000719468
ENSG00000169851	1.580000976	3.17E-06
ENSG00000169902	1.233704265	5.53E-05
ENSG00000169914	1.213231158	0.014099028
ENSG00000169946	1.256687486	2.32E-06
ENSG00000169967	1.336877431	9.35E-05
ENSG00000170011	-1.422312341	0.000243233
ENSG00000170264	-1.273991617	0.002656332
ENSG00000170271	-1.448679356	0.000465498
ENSG00000170293	1.710657033	7.20E-09
ENSG00000170364	1.280979527	0.001519337
ENSG00000170365	-1.735914016	9.32E-13
ENSG00000170425	-1.262020281	0.011638865
ENSG00000170485	-1.284507397	3.46E-08
ENSG00000170537	1.263169145	0.004802868
ENSG00000170558	1.348010625	3.77E-11
ENSG00000170775	1.928652352	5.87E-17
ENSG00000170873	-1.214157516	0.003461943
ENSG00000170921	1.44220633	5.80E-07
ENSG00000171056	-1.331719201	1.32E-05
ENSG00000171130	-1.237569451	0.001914833
ENSG00000171227	-1.609391038	3.29E-07
ENSG00000171241	-1.210051592	0.043471859
ENSG00000171246	3.613250748	1.74E-15

Gene ID	FC 0NG - 4NG	adj p-value 0NG - 4NG
ENSG00000171295	1.258282557	0.001714319
ENSG00000171310	1.625207583	5.64E-09
ENSG00000171316	1.270887496	3.59E-05
ENSG00000171345	-1.229721012	0.020463343
ENSG00000171388	-1.579415055	1.12E-06
ENSG00000171435	-1.371320471	3.95E-06
ENSG00000171469	1.228896893	0.000564415
ENSG00000171488	1.430216396	1.39E-06
ENSG00000171552	-1.349517371	1.11E-08
ENSG00000171604	1.325634395	0.016963927
ENSG00000171617	1.234388696	0.003203884
ENSG00000171621	-1.837873811	5.07E-07
ENSG00000171658	1.55569717	2.17E-05
ENSG00000171729	-1.354897561	2.37E-05
ENSG00000171848	-1.213660563	0.010425878
ENSG00000171889	1.373678265	0.000416515
ENSG00000171940	1.295961732	2.08E-06
ENSG00000172031	-1.446247587	0.000328298
ENSG00000172062	1.244861675	0.020894492
ENSG00000172081	-1.283828248	1.62E-08
ENSG00000172086	1.302407221	4.10E-05
ENSG00000172159	1.405177443	1.08E-06
ENSG00000172331	1.27840942	0.005297589
ENSG00000172578	1.30811481	2.30E-08
ENSG00000172602	1.713566875	3.21E-06
ENSG00000172731	-1.381575482	8.50E-05
ENSG00000172765	-1.228591773	2.08E-06
ENSG00000172819	1.367568149	0.000163733
ENSG00000173068	-1.216258765	0.000203181
ENSG00000173145	1.214940465	0.023654184
ENSG00000173153	1.223043952	6.31E-06
ENSG00000173207	-1.261150112	0.000163225

Gene ID	FC 0NG - 4NG	adj p-value 0NG - 4NG
ENSG00000173210	-1.21907683	3.47E-09
ENSG00000173281	1.212959343	0.000132254
ENSG00000173334	-1.429946907	0.000408212
ENSG00000173535	-1.560052964	8.78E-10
ENSG00000173548	-1.348928483	7.38E-09
ENSG00000173559	1.467598287	0.00055562
ENSG00000173597	1.459024104	0.000661488
ENSG00000173599	-1.21236161	0.003683235
ENSG00000173638	-1.219416033	0.002331355
ENSG00000173706	-1.200178158	0.000291564
ENSG00000173821	-1.23825841	0.004982297
ENSG00000173868	1.312801115	0.000012149
ENSG00000173894	-1.213388182	0.00883949
ENSG00000174004	-1.705087507	5.67E-07
ENSG00000174059	-1.930730247	1.53E-05
ENSG00000174348	-1.235296697	0.045912826
ENSG00000174370	-1.555761069	0.000019627
ENSG00000174684	-1.213112977	0.004820281
ENSG00000174705	-1.231073029	1.78E-06
ENSG00000174796	1.344329172	0.004923899
ENSG00000174804	1.253615426	3.70E-09
ENSG00000175040	1.313006888	9.58E-06
ENSG00000175063	-1.284632622	0.005603013
ENSG00000175216	-1.203023865	0.000014961
ENSG00000175264	-2.126095403	4.63E-17
ENSG00000175274	-1.306282075	8.33E-06
ENSG00000175348	1.208501799	1.59E-05
ENSG00000175352	1.291622475	0.000350363
ENSG00000175426	1.330923284	0.002124807
ENSG00000175471	1.211948298	0.014227314
ENSG00000175591	-1.318607439	0.009154582
ENSG00000175643	-1.289996263	0.003471877

Gene ID	FC 0NG - 4NG	adj p-value 0NG - 4NG
ENSG00000175746	1.451830575	0.000025777
ENSG00000175832	-1.246449592	0.00023973
ENSG00000175899	1.37284203	0.024449347
ENSG00000175938	-1.280281136	0.015579876
ENSG00000176102	-1.20711288	0.003544851
ENSG00000176170	1.316176374	6.61E-06
ENSG00000176273	1.419008359	0.004219878
ENSG00000176438	-1.274366065	5.93E-05
ENSG00000176749	-1.228082181	0.036666149
ENSG00000176834	1.204140938	0.00001272
ENSG00000176845	1.334720623	0.000795499
ENSG00000176890	-1.239297757	6.23E-05
ENSG00000176907	2.372197677	3.86E-10
ENSG00000176974	-1.287659916	2.98E-07
ENSG00000177000	-1.218361065	0.007083943
ENSG00000177076	-1.231494728	0.029056764
ENSG00000177084	-1.228020714	0.001382358
ENSG00000177191	-1.310101176	0.001843935
ENSG00000177337	-1.219134978	0.047813593
ENSG00000177374	-2.181581112	1.39E-11
ENSG00000177426	-1.374140608	2.62E-08
ENSG00000177432	1.265946441	0.048733305
ENSG00000177602	-1.385188415	0.000555389
ENSG00000177606	-1.412920096	9.17E-12
ENSG00000177663	1.225440485	4.93E-05
ENSG00000177679	1.985112206	1.03E-09
ENSG00000177706	1.261429486	2.05E-08
ENSG00000177732	-1.516358261	8.25E-10
ENSG00000178038	-1.296790662	0.003815513
ENSG00000178105	1.294702919	3.68E-08
ENSG00000178175	-1.419085654	0.001652045
ENSG00000178209	-1.299339037	0.000192819

Gene ID	FC 0NG - 4NG	adj p-value 0NG - 4NG
ENSG00000178381	1.214290048	0.038728714
ENSG00000178401	1.319925973	0.013837077
ENSG00000178409	1.350452758	2.88E-07
ENSG00000178445	-1.294278117	0.001025665
ENSG00000178567	1.365989973	0.000764588
ENSG00000178685	-1.219128308	0.011594723
ENSG00000178695	-1.318308091	3.89E-06
ENSG00000178718	-1.32312303	7.76E-07
ENSG00000178726	-1.375057818	5.92E-06
ENSG00000178764	1.31723128	0.000408538
ENSG00000178882	1.642505272	6.90E-06
ENSG00000178971	-1.285139392	8.33E-05
ENSG00000178999	-1.335932507	0.001819315
ENSG00000179044	1.4413656	7.45E-05
ENSG00000179094	1.243340356	0.001266074
ENSG00000179144	-1.314540222	3.90E-06
ENSG00000179314	-1.761880853	3.29E-11
ENSG00000179403	2.178707554	1.31E-09
ENSG00000179428	1.339758907	0.035728448
ENSG00000179456	-1.414120466	1.38E-09
ENSG00000179546	-1.516311404	0.000038628
ENSG00000179630	1.339480825	0.007625901
ENSG00000179750	-1.262868852	0.010471351
ENSG00000179820	1.409138452	1.45E-13
ENSG00000179833	1.205695107	0.000196465
ENSG00000179862	-1.377494058	0.00177214
ENSG00000179941	1.299262626	0.018791859
ENSG00000179981	-1.327582969	1.45E-07
ENSG00000180354	1.448921364	0.000111192
ENSG00000180448	-1.492153128	0.00068587
ENSG00000180530	-1.229610229	0.003050183
ENSG00000180537	-1.222887909	0.021278369
ENSG00000180596	-1.264164675	0.004515876
ENSG00000180694	1.256485785	0.002267278
ENSG00000180884	-1.347681799	0.000107651
ENSG00000180891	-1.203184871	0.002461967
ENSG00000180998	2.699371728	3.54E-12
ENSG00000181444	-1.242575885	0.038249498
ENSG00000181472	1.570018832	3.41E-07
ENSG00000181541	1.730031827	2.63E-08
ENSG00000181690	1.223693791	0.030295561
ENSG00000181751	1.231344518	0.007764318
ENSG00000181754	-1.254987439	0.039298936
ENSG00000182022	-1.989418772	4.45E-22
ENSG00000182095	-1.237986972	4.25E-06
ENSG00000182118	-1.614610462	5.71E-05
ENSG00000182197	1.657292867	7.99E-23
ENSG00000182253	1.498409637	4.77E-07
ENSG00000182405	1.288660724	0.019114882
ENSG00000182481	-1.28143331	2.54E-08
ENSG00000182541	-1.211632727	0.000294027
ENSG00000182568	1.356572945	0.006644709
ENSG00000182575	-1.380017158	0.001631203
ENSG00000182986	1.202212217	0.00499848
ENSG00000183020	-1.200598346	0.016450002
ENSG00000183049	1.789794144	3.81E-08
ENSG00000183054	1.335612726	0.012901431
ENSG00000183077	-1.217284658	0.012419499
ENSG00000183090	1.262608316	0.001773337
ENSG00000183208	-1.315370946	0.00412579
ENSG00000183337	-1.276967904	0.026602468
ENSG00000183340	1.205572752	0.000531856
ENSG00000183386	-1.22310452	0.001455731
ENSG00000183496	-1.585392696	5.67E-07
ENSG00000183578	1.392647043	2.74E-07
ENSG00000183580	-1.416694939	1.32E-12
ENSG00000183598	-1.266960328	0.003223066
ENSG00000183688	1.241462338	0.000635155
ENSG00000183691	1.928614418	1.65E-07
ENSG00000183775	1.262590176	0.000318432
ENSG00000183779	-1.354760752	0.000489648
ENSG00000183856	-1.295706231	0.000594214
ENSG00000183873	-1.277392182	0.000675452
ENSG00000183935	1.248849489	0.000958419
ENSG00000183943	-1.203016549	3.49E-08
ENSG00000184009	1.253485422	2.61E-12
ENSG00000184014	1.218403921	3.22E-10
ENSG00000184113	-2.021955107	8.23E-14
ENSG00000184226	1.862278952	2.11E-09
ENSG00000184260	-1.247049784	0.005916634
ENSG00000184270	-1.407498148	0.001356272
ENSG00000184304	1.319254821	1.12E-06
ENSG00000184307	2.092780361	3.35E-09
ENSG00000184357	-1.339731222	0.001330163
ENSG00000184384	1.336800201	2.73E-06
ENSG00000184445	-1.235235029	0.035878148
ENSG00000184465	-1.307420431	0.00542432
ENSG00000184661	-1.381323658	0.000406375
ENSG00000184678	-1.23749706	0.007094966
ENSG00000184702	1.326893996	0.006901073
ENSG00000184792	-1.235161581	0.005803029
ENSG00000184916	1.496161547	6.33E-10
ENSG00000184988	1.252853677	0.000895352
ENSG00000185015	1.490717352	5.89E-06
ENSG00000185022	-1.491285571	3.75E-10
ENSG00000185033	-1.378844085	5.17E-05

Gene ID	FC 0NG - 4NG	adj p-value 0NG - 4NG
ENSG00000185070	-1.267151192	0.001198246
ENSG00000185112	-1.923460463	8.05E-17
ENSG00000185130	-1.375201127	0.000828968
ENSG00000185187	-1.208377365	0.00372595
ENSG00000185222	1.216256887	0.000364507
ENSG00000185262	-1.358246002	5.78E-07
ENSG00000185269	1.810543799	1.13E-11
ENSG00000185338	-1.83244975	1.72E-07
ENSG00000185361	-1.690160816	8.03E-12
ENSG00000185432	-1.33729344	5.75E-07
ENSG00000185453	-1.336785755	0.000746965
ENSG00000185483	1.265033798	0.005177834
ENSG00000185567	-1.302963353	0.000808877
ENSG00000185630	-1.26858991	0.014505376
ENSG00000185697	-1.312494684	0.043027625
ENSG00000185716	1.218074489	0.020374788
ENSG00000185728	1.285032792	2.27E-06
ENSG00000185909	-1.280387975	0.007602272
ENSG00000185972	1.30352674	0.040246277
ENSG00000186185	-1.431401074	0.000168556
ENSG00000186193	-1.527134942	4.71E-09
ENSG00000186432	1.24115387	0.000179793
ENSG00000186479	-1.570674104	1.52E-07
ENSG00000186577	-1.212588104	0.021123774
ENSG00000186591	-1.256838304	1.16E-08
ENSG00000186603	1.251894345	0.013386436
ENSG00000186635	-1.276700762	1.61E-06
ENSG00000186638	-1.279846086	0.032627748
ENSG00000186642	-1.334593506	0.000347536
ENSG00000186665	-1.334920853	0.000697988
ENSG00000186812	1.423156812	0.000640461
ENSG00000186827	-1.205983694	0.034972406

Gene ID	FC 0NG - 4NG	adj p-value 0NG - 4NG
ENSG00000186871	-1.325705906	0.002865913
ENSG00000186918	-1.736214515	1.46E-09
ENSG00000186951	-1.317271361	0.000165647
ENSG00000187189	1.308987605	8.56E-08
ENSG00000187266	-1.369625479	9.43E-06
ENSG00000187555	1.231150666	7.24E-09
ENSG00000187605	-1.307411966	5.23E-07
ENSG00000187609	-1.200955312	0.04272498
ENSG00000187634	1.547692747	0.000173488
ENSG00000187741	-1.267679837	0.008027989
ENSG00000187742	1.235270451	1.49E-06
ENSG00000187764	-1.932803047	2.18E-11
ENSG00000187800	-1.42096452	2.69E-11
ENSG00000187801	1.331595733	0.003180838
ENSG00000187815	1.2891057	0.015145449
ENSG00000187837	-1.289959588	0.001242152
ENSG00000187840	-1.203024076	0.001313559
ENSG00000187951	-1.30127586	0.036635016
ENSG00000188033	1.23775046	0.025699251
ENSG00000188042	-1.979319085	3.51E-09
ENSG00000188277	-2.114422229	4.37E-05
ENSG00000188290	1.255555189	0.013065431
ENSG00000188312	-1.237777846	0.018056845
ENSG00000188315	-1.228722198	0.027480124
ENSG00000188428	1.552369988	1.79E-06
ENSG00000188486	-1.230171794	0.002830626
ENSG00000188549	-1.271080441	0.001545048
ENSG00000188732	-1.531449583	0.001536191
ENSG00000188811	-1.322855458	0.002475125
ENSG00000188827	-1.217497607	0.002074795
ENSG00000189067	1.360433542	0.002226361
ENSG00000189223	-1.240617694	0.03965391

Gene ID	FC 0NG - 4NG	adj p-value 0NG - 4NG
ENSG00000196072	1.292205778	0.000121071
ENSG00000196083	1.276210116	0.001491935
ENSG00000196110	1.243731165	0.038047938
ENSG00000196169	-1.920999214	1.08E-09
ENSG00000196187	-1.263157502	0.001087929
ENSG00000196268	1.219488844	0.031684908
ENSG00000196352	1.305313845	5.26E-06
ENSG00000196411	1.22497017	7.16E-06
ENSG00000196421	-1.264216741	0.006624266
ENSG00000196422	-1.286285887	0.001040228
ENSG00000196449	1.222555465	3.78E-05
ENSG00000196468	-1.307598738	0.011173237
ENSG00000196526	1.202967772	1.02E-08
ENSG00000196576	-1.241705782	8.41E-06
ENSG00000196646	1.215489288	0.030686731
ENSG00000196730	-1.539236665	3.13E-16
ENSG00000196747	-1.32154341	0.003911165
ENSG00000196781	-1.445317742	4.87E-13
ENSG00000196782	-1.553593489	5.24E-14
ENSG00000196787	-1.289103493	0.008373154
ENSG00000196792	1.325551727	7.61E-07
ENSG00000196866	-1.344328019	0.000178219
ENSG00000196876	-1.403676759	1.41E-05
ENSG00000196890	-1.490133488	0.00001204
ENSG00000196967	1.293845684	0.033323954
ENSG00000196981	1.318387288	0.005876239
ENSG00000197019	1.282317775	7.16E-05
ENSG00000197061	-1.350550462	0.000364974
ENSG00000197128	1.211858421	0.013080973
ENSG00000197153	-1.27871163	0.038521257
ENSG00000197238	-1.252666991	0.002415673
ENSG00000197256	-1.372519521	1.79E-11

Gene ID	FC 0NG - 4NG	adj p-value 0NG - 4NG
ENSG00000197261	-1.635611979	2.05E-06
ENSG00000197321	-1.200843773	6.44E-07
ENSG00000197363	1.361532991	0.001434191
ENSG00000197380	1.380308581	0.008108795
ENSG00000197409	-1.306825174	0.00479239
ENSG00000197442	1.206313825	0.004771191
ENSG00000197461	1.726776809	1.50E-10
ENSG00000197498	1.240305885	0.00107658
ENSG00000197530	-1.232982431	0.024191109
ENSG00000197536	-1.32515238	0.005166385
ENSG00000197557	1.256095267	0.010943463
ENSG00000197566	1.273799945	0.006694768
ENSG00000197608	1.20012233	0.048477101
ENSG00000197619	1.282470912	0.020466396
ENSG00000197632	1.581665024	6.64E-05
ENSG00000197771	1.219442851	9.14E-06
ENSG00000197780	1.404592154	0.001937985
ENSG00000197852	-1.763358395	2.80E-14
ENSG00000197857	1.392160309	4.11E-05
ENSG00000197860	1.306166546	0.000121723
ENSG00000197872	-1.267058891	6.14E-08
ENSG00000197903	-1.275883422	0.008860503
ENSG00000197928	1.358624148	3.46E-05
ENSG00000197943	1.22798937	0.026999587
ENSG00000198018	1.347070091	3.84E-07
ENSG00000198042	1.380356211	1.16E-09
ENSG00000198060	1.353434709	4.43E-08
ENSG00000198108	3.595458217	7.83E-17
ENSG00000198342	1.36730325	0.000555305
ENSG00000198346	1.336194402	0.000594986
ENSG00000198355	-1.574121993	9.40E-11
ENSG00000198369	-1.340140218	1.04E-06

Gene ID	FC 0NG - 4NG	adj p-value 0NG - 4NG
ENSG00000198435	-1.633331021	1.69E-07
ENSG00000198440	1.286734242	0.016220133
ENSG00000198464	1.227594765	0.013508057
ENSG00000198538	1.26792667	0.000719583
ENSG00000198604	1.301077822	1.60E-06
ENSG00000198624	-1.210190866	0.003124794
ENSG00000198646	-1.248265556	8.80E-08
ENSG00000198648	1.200297804	0.006198853
ENSG00000198722	-1.27729003	1.41E-10
ENSG00000198805	1.344694017	1.06E-11
ENSG00000198846	1.486756876	0.000109336
ENSG00000198855	1.221952617	0.000121105
ENSG00000198873	-1.426001681	2.18E-10
ENSG00000198901	-1.256564391	0.000482771
ENSG00000198909	-1.280628321	3.46E-10
ENSG00000198929	-1.259801383	0.036085574
ENSG00000198933	-1.223473209	0.001739211
ENSG00000203485	-1.316031871	1.31E-07
ENSG00000203805	1.402005252	0.000469098
ENSG00000203814	-1.310501759	0.000216333
ENSG00000203883	-2.514064291	4.08E-12
ENSG00000204131	-1.256800121	6.59E-06
ENSG00000204152	1.275993525	0.000400732
ENSG00000204217	1.823010117	1.54E-12
ENSG00000204381	1.2646817	0.027839662
ENSG00000204406	-1.487292337	1.94E-08
ENSG00000204442	1.842445099	1.44E-10
ENSG00000204604	1.297925119	5.47E-06
ENSG00000204682	1.323021468	0.022180414
ENSG00000204758	1.244054599	0.028833503
ENSG00000204767	1.302558878	4.05E-08
ENSG00000204856	1.209289682	0.023792893

Gene ID	FC 0NG - 4NG	adj p-value 0NG - 4NG
ENSG00000204977	1.21642296	0.035853223
ENSG00000204991	-1.262674602	0.002697164
ENSG00000205090	-1.24070796	0.007174601
ENSG00000205213	-1.323667632	0.00047514
ENSG00000205250	1.216652652	7.28E-05
ENSG00000205269	-1.523875764	0.001289332
ENSG00000205356	-1.383145524	1.79E-09
ENSG00000205476	-1.475427413	6.07E-10
ENSG00000205683	-1.346300495	0.000012943
ENSG00000205710	1.268635312	0.029342332
ENSG00000205808	1.250749207	0.010949418
ENSG00000205978	-1.259679094	0.001507641
ENSG00000206538	2.572878856	3.35E-16
ENSG00000206560	-1.289656397	2.34E-07
ENSG00000211455	1.780341321	1.44E-08
ENSG00000212719	-1.203950522	0.008500122
ENSG00000213047	1.396317503	0.001399471
ENSG00000213064	1.280722163	0.000253283
ENSG00000213085	1.363751805	0.00222478
ENSG00000213160	-1.446279976	0.000607904
ENSG00000213190	1.27196593	0.000337253
ENSG00000213203	-1.363272411	2.59E-05
ENSG00000213347	-1.550009032	4.88E-07
ENSG00000213516	1.246805263	0.005920348
ENSG00000213626	1.856316735	1.79E-08
ENSG00000213672	-1.235901996	0.005312696
ENSG00000213693	1.265710788	0.04823392
ENSG00000213694	-1.659329522	2.34E-15
ENSG00000213853	-1.239168424	0.001725651
ENSG00000214357	-1.859704494	3.47E-06
ENSG00000214595	1.261257045	0.024590727
ENSG00000214944	-1.680348686	3.68E-13

Gene ID	FC 0NG - 4NG	adj p-value 0NG - 4NG
ENSG00000214960	1.265918848	0.040449617
ENSG00000215012	-1.279918431	0.000270302
ENSG00000215458	-1.418045538	0.001417977
ENSG00000216775	-1.859350601	5.50E-10
ENSG00000221829	-1.334251683	3.79E-05
ENSG00000221866	1.324630531	0.018745366
ENSG00000221926	-1.346619625	3.67E-08
ENSG00000223764	1.383568816	5.21E-08
ENSG00000224051	-1.243316004	0.000511638
ENSG00000224080	1.280123904	0.01164522
ENSG00000224531	1.36692395	5.92E-06
ENSG00000225206	1.287716347	0.022435863
ENSG00000225377	-1.216336586	0.02420319
ENSG00000225830	1.422131744	1.84E-08
ENSG00000225968	2.917196729	1.99E-13
ENSG00000226137	-1.2104094	0.009898223
ENSG00000226808	1.317650317	0.000869822
ENSG00000227517	1.416852699	0.00099053
ENSG00000228495	1.279921201	0.000015846
ENSG00000229372	-1.263838242	0.046543082
ENSG00000229619	1.366489793	0.000847208
ENSG00000229953	-1.450952736	0.000103952
ENSG00000230266	-1.529572987	7.01E-06
ENSG00000230438	-1.428436232	0.000540433
ENSG00000230479	-1.284540491	8.76E-07
ENSG00000230753	-1.248464453	0.005522983
ENSG00000231856	1.358611479	0.000609135
ENSG00000232533	1.253622066	0.000491666
ENSG00000233370	1.302680644	0.000241508
ENSG00000233766	-1.464654387	0.006013042
ENSG00000233822	-1.433581491	7.80E-05
ENSG00000234072	1.260187491	0.039698943

Gene ID	FC 0NG - 4NG	adj p-value 0NG - 4NG
ENSG00000235257	-1.244818999	0.005559969
ENSG00000235884	1.249315124	0.006071458
ENSG00000235954	-1.255697055	0.02346355
ENSG00000237036	1.301256663	0.001559082
ENSG00000237172	1.230363907	0.009096349
ENSG00000237181	1.326658785	0.000643521
ENSG00000237440	1.214669271	0.044282011
ENSG00000239305	1.209194907	0.005050166
ENSG00000239306	1.316199115	5.05E-10
ENSG00000239887	-1.637310516	1.03E-08
ENSG00000240225	1.215140614	0.001925427
ENSG00000240694	-1.631992562	1.37E-13
ENSG00000240891	-1.363764322	0.000859211
ENSG00000241288	1.234968372	0.034355674
ENSG00000241764	1.233448664	0.008705895
ENSG00000241978	-1.248358479	0.005097144
ENSG00000244300	1.269812998	0.000651143
ENSG00000244462	1.233685779	4.77E-05
ENSG00000247287	-1.342979737	0.030941456
ENSG00000247679	-1.300200601	0.025906714
ENSG00000248008	1.221250119	0.0426795
ENSG00000248441	-1.304268441	9.42E-05
ENSG00000248866	1.211549539	0.045967224
ENSG00000249087	1.214023673	0.035063185
ENSG00000249279	-1.627623981	4.83E-07
ENSG00000249471	1.296503265	0.004331114
ENSG00000250303	1.372318003	9.23E-05
ENSG00000250510	-1.506950821	1.09E-06
ENSG00000250899	-1.350142283	1.89E-06
ENSG00000251493	1.386421253	0.003829988
ENSG00000253368	1.871625893	1.89E-16
ENSG00000254004	1.26402311	0.003467419

Gene ID	FC 0NG - 4NG	adj p-value 0NG - 4NG
ENSG00000254087	1.332979787	1.49E-08
ENSG00000254230	-1.217747871	0.032227208
ENSG00000255248	1.402175108	0.000560493
ENSG00000255690	-1.31983286	0.006278461
ENSG00000256268	1.255424399	0.03085691
ENSG00000256628	1.351131751	0.000174644
ENSG00000257923	1.253498817	2.67E-06
ENSG00000258376	1.335747936	0.000373252
ENSG00000259943	1.285050192	0.007631172
ENSG00000260231	1.290357015	0.002523974
ENSG00000260267	1.251402288	0.006550788
ENSG00000260604	1.220077408	0.001019566
ENSG00000260920	1.308994288	0.002238356
ENSG00000260996	1.366659282	0.00292799
ENSG00000261253	-1.446896803	2.50E-05
ENSG00000261373	1.46802161	2.17E-08
ENSG00000262601	1.376317257	0.001750702
ENSG00000263155	-1.478514238	0.000123391
ENSG00000263528	1.338221035	8.76E-07
ENSG00000265808	1.209905387	2.60E-05
ENSG00000265972	1.543351942	5.21E-08
ENSG00000267045	1.276390649	0.001761566
ENSG00000267316	1.201864612	0.02397128
ENSG00000267365	1.539987312	0.003893508
ENSG00000267583	-1.505965186	3.91E-10
ENSG00000269906	1.298201495	0.000845909
ENSG00000269973	-1.252412378	0.03469579
ENSG00000270959	1.404111715	0.0062994
ENSG00000271936	-1.210053839	0.049590277
ENSG00000272398	1.359724846	7.97E-05
ENSG00000272405	-1.446231337	0.005831868
ENSG00000272447	1.207991825	0.02146437

Gene ID	FC 0NG - 4NG	adj p-value 0NG - 4NG
ENSG00000272768	1.248325595	0.021598792
ENSG00000272886	1.22582669	7.81E-06
ENSG00000273489	1.408444637	0.008643112
ENSG00000273802	-1.20861616	0.039346059
ENSG00000273983	-1.387438531	0.000503649
ENSG00000274267	-1.307229236	0.010227446
ENSG00000274272	-1.282236624	0.016680544
ENSG00000274276	-1.255957256	0.041878924
ENSG00000274290	-1.322169677	0.000693233
ENSG00000274641	-1.338682677	0.004185081
ENSG00000274897	1.238214035	0.017004476
ENSG00000274995	1.412556279	0.008772823
ENSG00000274997	-1.328547689	0.006918754
ENSG00000275074	1.269324913	0.026617924
ENSG00000275126	-1.39914464	0.000193149
ENSG00000275342	-1.472961029	4.78E-07
ENSG00000275379	-1.314359587	0.006342666
ENSG00000275481	1.253550558	0.006699984
ENSG00000275713	-1.482792908	0.000256968
ENSG00000275993	-1.421617921	0.000317398
ENSG00000276043	-1.318481527	0.00002037
ENSG00000276368	-1.296387991	0.011146778
ENSG00000276410	-1.285261033	0.01080646
ENSG00000276600	1.256056226	0.017610724
ENSG00000276644	1.467792487	0.000203517
ENSG00000276836	-1.295326693	0.026036095
ENSG00000276849	-1.513020366	0.002880527
ENSG00000276903	-1.304670396	0.015088591
ENSG00000277075	-1.326918114	0.001084254
ENSG00000277157	-1.339673719	0.00254757
ENSG00000277224	-1.386249809	0.000476767
ENSG00000277639	-1.281544039	0.006163497

Gene ID	FC 0NG - 4NG	adj p-value 0NG - 4NG
ENSG00000277775	-1.435632193	0.000278179
ENSG00000278272	-1.361365902	0.001273731
ENSG00000278463	-1.303952577	0.004866978
ENSG00000278588	-1.322721215	0.003191295
ENSG00000278705	-1.220516966	0.013762985
ENSG00000278828	-1.329023225	0.001732969
ENSG00000279095	1.340365662	2.06E-07
ENSG00000279117	-1.650511934	7.29E-14
ENSG00000279207	-1.220789262	0.019690021
ENSG00000279289	1.445733194	0.003907265
ENSG00000279365	-1.311798206	5.60E-05
ENSG00000279369	1.242284104	0.02566512
ENSG00000279496	-1.277393726	0.012447846
ENSG00000279881	1.216213557	0.000298849
ENSG00000279948	-1.228346097	0.049293253
ENSG00000280143	1.315014773	0.006479195
ENSG00000280351	-1.208526305	0.04639392
ENSG00000281614	-1.276263227	0.005000911
ENSG00000282057	1.391709256	0.002560324

Supplementary Table 1d. Differentially expressed genes between 0hr and 8hr normal glucose samples

Ensembl gene IDs, Fold change (baseline-8NG) and FDR adjusted p-values of DEGs

Gene ID	FC 0NG - 8NG	adj p-value 0NG - 8NG
ENSG00000000460	-1.345441942	0.003269094
ENSG00000002079	-1.431907022	0.001459356
ENSG00000002822	-1.216590603	0.000494645
ENSG00000003989	1.751661287	9.45E-07
ENSG00000005108	-1.264450938	0.014700012
ENSG00000005238	1.25312727	1.89E-06
ENSG00000006576	1.248371261	0.01986632
ENSG00000006638	1.371725438	0.00022807
ENSG00000007168	1.206647732	3.44E-07
ENSG00000007384	-1.240023179	0.010532872
ENSG00000007968	1.482883403	0.000929653
ENSG00000008294	1.240508435	1.24E-09
ENSG00000008405	-1.247873572	0.000387447
ENSG00000008441	-1.207693309	5.66E-07
ENSG00000010292	-1.4630945	7.15E-13
ENSG00000010818	-1.369923289	0.001438706
ENSG00000011405	1.395352292	5.26E-06
ENSG00000011422	-1.229223663	0.001137918
ENSG00000011426	-1.449485448	5.13E-07
ENSG00000012232	-1.218607758	9.84E-08
ENSG00000013016	1.345787066	0.00153262
ENSG00000013293	1.398869297	0.000436389
ENSG00000013810	-1.62337317	7.39E-08
ENSG00000014123	1.284336086	2.22E-05
ENSG00000019144	1.253674178	8.13E-06
ENSG00000020577	1.200004726	2.64E-07
ENSG00000021645	1.818495132	1.39E-09
ENSG00000022567	1.265679338	0.031413887
ENSG00000023902	-1.203719056	0.003286654
ENSG00000026950	-1.365002687	7.86E-07
ENSG00000029993	-1.201239809	0.000177779
ENSG00000035499	-1.567945937	8.46E-08

Gene ID	FC 0NG - 8NG	adj p-value 0NG - 8NG
ENSG00000038295	1.534984185	6.15E-06
ENSG00000042088	-1.22706256	3.10E-06
ENSG00000046889	1.330037069	3.78E-06
ENSG00000047617	1.238103606	0.015005443
ENSG00000047621	1.250992391	0.003557356
ENSG00000048052	1.711930017	0.00003737
ENSG00000048405	1.208474539	0.030359282
ENSG00000049192	1.372830056	8.53E-08
ENSG00000049759	1.464609948	1.70E-08
ENSG00000051128	-1.286407242	1.60E-06
ENSG00000051341	-1.294451282	0.004477733
ENSG00000052749	1.259498669	1.91E-06
ENSG00000053747	-1.283035839	0.000172278
ENSG00000054967	-1.240817225	0.023870729
ENSG00000056097	1.207200964	4.16E-07
ENSG00000057019	1.36381699	9.73E-06
ENSG00000057704	-1.267396951	3.32E-05
ENSG00000058085	-1.266604775	0.0095379
ENSG00000058091	1.392375906	1.70E-07
ENSG00000059378	-1.307824159	2.87E-06
ENSG00000060140	1.217855172	0.000196343
ENSG00000060656	-1.24297409	0.017546899
ENSG00000062822	-1.217192316	0.002306564
ENSG00000064692	-1.92021745	7.31E-06
ENSG00000064961	-1.22112792	0.000448596
ENSG00000064989	1.412278045	0.002055524
ENSG00000065054	1.220227471	0.003635916
ENSG00000065135	1.21283417	6.27E-05
ENSG00000065308	-1.228626178	6.85E-06
ENSG00000065717	-1.283125094	0.001161448
ENSG00000065989	1.254570398	0.004407387
ENSG00000066279	-1.467024177	0.010538029

Gene ID	FC 0NG - 8NG	adj p-value 0NG - 8NG
ENSG00000066923	-1.250452094	0.001743908
ENSG00000067208	1.287790913	5.31E-06
ENSG00000067221	1.256501227	0.00130994
ENSG00000067533	1.29311249	6.01E-06
ENSG00000067715	1.288666627	0.013195071
ENSG00000067992	-1.268293339	0.021776946
ENSG00000068078	1.250638796	0.006917382
ENSG00000068366	1.237576241	7.91E-05
ENSG00000068489	-1.414111881	1.48E-09
ENSG00000070404	1.357830568	2.71E-05
ENSG00000070610	-1.276064045	0.0147097
ENSG00000070614	-1.439935331	2.17E-13
ENSG00000070669	-1.371551404	1.34E-05
ENSG00000070961	1.268020477	0.004324644
ENSG00000071246	-1.668032155	7.97E-07
ENSG00000071539	-1.340302394	2.45E-06
ENSG00000072110	1.215295337	2.97E-06
ENSG00000072201	-1.230417403	0.023436744
ENSG00000072571	-1.309862698	0.016732743
ENSG00000073008	1.327969146	1.19E-09
ENSG00000073150	-1.217617469	0.015737274
ENSG00000073464	1.201046002	0.003098261
ENSG00000073711	-1.302844218	0.000644541
ENSG00000073803	1.213420621	0.009414383
ENSG00000073921	1.370543093	5.41E-06
ENSG00000074410	1.259068234	0.019569741
ENSG00000074590	1.238360068	0.025038008
ENSG00000075218	-1.644667278	3.72E-08
ENSG00000075702	-1.466037973	2.51E-05
ENSG00000075711	-1.24150966	0.000167076
ENSG00000075790	1.26303559	0.038610744
ENSG00000076382	-1.549823086	1.30E-09

Gene ID	FC 0NG - 8NG	adj p-value 0NG - 8NG
ENSG00000077147	1.353418968	0.006367063
ENSG00000078018	1.884414803	5.93E-09
ENSG00000078043	1.238110823	0.001179647
ENSG00000078114	1.31991823	0.020074753
ENSG00000078401	1.298041987	2.54E-05
ENSG00000078487	1.202138316	0.014718039
ENSG00000079257	-1.356591878	3.86E-05
ENSG00000079435	-1.365036704	0.000691709
ENSG00000079616	-1.587172816	1.28E-09
ENSG00000079841	1.289400494	0.006573465
ENSG00000080561	-1.240823063	2.76E-05
ENSG00000080986	-1.52193207	2.82E-06
ENSG00000081059	1.336507486	0.004526216
ENSG00000081320	-1.309058627	0.002518786
ENSG00000082126	-1.357303602	1.44E-06
ENSG00000082438	1.20220331	0.005356757
ENSG00000085117	-1.403160898	1.82E-07
ENSG00000085563	1.265046161	0.02561497
ENSG00000085871	-1.221098873	1.85E-05
ENSG00000085999	-1.347607868	0.00857168
ENSG00000087586	-1.4357245	1.86E-07
ENSG00000087842	-1.39439294	1.62E-09
ENSG00000088305	-1.247405932	0.032639585
ENSG00000088325	-1.585224379	1.35E-09
ENSG00000088836	-1.215349129	0.008097443
ENSG00000088986	1.262793277	2.10E-10
ENSG00000089195	1.215975735	0.001678835
ENSG00000089280	1.253586779	4.37E-09
ENSG00000089351	-1.200154511	0.004426077
ENSG00000089685	-1.467520069	7.58E-09
ENSG00000089820	-1.390876447	0.000201585
ENSG00000090020	-1.233480337	3.73E-06

Gene ID	FC 0NG - 8NG	adj p-value 0NG - 8NG
ENSG00000090238	-1.371849145	1.71E-05
ENSG00000090530	1.426491671	0.00038149
ENSG00000090889	-1.631542422	3.87E-09
ENSG00000091129	1.836063179	1.93E-15
ENSG00000091137	-1.265449845	0.01411776
ENSG00000091622	1.457335148	0.006606026
ENSG00000091656	1.395006617	9.66E-06
ENSG00000091844	-1.231399308	0.048415384
ENSG00000091879	1.87324766	3.00E-16
ENSG00000092108	1.234677442	0.005329028
ENSG00000092531	1.207146447	5.70E-07
ENSG00000092621	-1.542015464	2.07E-08
ENSG00000094914	-1.209344902	8.13E-05
ENSG00000095303	-1.245277905	2.46E-05
ENSG00000095383	1.280947862	0.004306113
ENSG00000096060	1.271705157	2.46E-12
ENSG00000096433	-1.23824194	4.09E-07
ENSG00000099204	-1.346758742	5.68E-07
ENSG00000099260	1.207742158	1.50E-05
ENSG00000099282	-1.366427461	5.88E-05
ENSG00000099822	1.31229584	0.03967801
ENSG00000099860	1.296183622	0.010390543
ENSG00000099889	-1.411537021	2.74E-06
ENSG00000100027	-1.408775282	0.003217183
ENSG00000100034	-1.273001075	2.38E-08
ENSG00000100162	-1.285015518	0.004027116
ENSG00000100292	-1.363226582	0.000019939
ENSG00000100342	-1.280390671	0.001648364
ENSG00000100368	1.353082146	7.75E-06
ENSG00000100399	1.272455972	1.41E-05
ENSG00000100439	-1.292194362	0.001939391
ENSG00000100522	1.211471028	0.01667191

Gene ID	FC 0NG - 8NG	adj p-value 0NG - 8NG
ENSG00000100526	-1.434820643	9.16E-06
ENSG00000100629	-1.646328845	4.03E-07
ENSG00000100697	1.253074637	3.36E-05
ENSG00000100889	-1.324130167	5.45E-05
ENSG00000101057	-1.317595881	0.000486997
ENSG00000101096	-1.72607752	5.16E-12
ENSG00000101188	-1.206612471	0.005040576
ENSG00000101224	-1.317611816	3.09E-06
ENSG00000101255	-1.254065534	0.00011575
ENSG00000101265	-1.343897395	1.23E-05
ENSG00000101445	-1.378669911	1.71E-05
ENSG00000101447	-1.718904731	6.68E-10
ENSG00000101665	1.411499171	0.03421907
ENSG00000101670	1.365988502	3.17E-06
ENSG00000101844	1.263432544	9.21E-09
ENSG00000101871	-1.430706759	8.02E-10
ENSG00000101945	-1.271562882	0.005419855
ENSG00000102010	-1.63773243	1.13E-11
ENSG00000102024	1.252560024	5.25E-07
ENSG00000102125	-1.209957689	0.006263014
ENSG00000102384	-1.297172737	0.003047972
ENSG00000102743	1.231210507	0.001718147
ENSG00000102755	1.440682575	2.75E-09
ENSG00000102935	1.325859708	0.026091166
ENSG00000102981	-1.279741938	0.040610051
ENSG00000103018	-1.213661123	5.98E-08
ENSG00000103152	-1.220597913	1.89E-05
ENSG00000103381	1.219126922	0.000133429
ENSG00000103642	1.245625075	0.004871116
ENSG00000103647	-1.346127394	6.99E-07
ENSG00000104047	1.334255208	0.039681182
ENSG00000104093	1.211947567	0.031391018

Gene ID	FC 0NG - 8NG	adj p-value 0NG - 8NG
ENSG00000104147	-1.373241994	0.001146969
ENSG00000104213	-1.210206822	0.015022662
ENSG00000104219	1.25832	2.97E-05
ENSG00000104368	-1.238351028	0.011243614
ENSG00000104812	1.228368456	0.00007608
ENSG00000104835	-1.238566705	0.038019561
ENSG00000104884	1.219400663	1.13E-05
ENSG00000104889	-1.295529113	0.002558669
ENSG00000104915	-1.204055181	0.005489967
ENSG00000104951	1.390115613	0.001451972
ENSG00000105088	1.730271415	6.97E-10
ENSG00000105173	1.395244994	6.06E-05
ENSG00000105325	-1.285873909	2.76E-07
ENSG00000105443	-1.274449035	0.000339628
ENSG00000105486	-1.378329859	8.96E-10
ENSG00000105497	1.206016361	0.00200664
ENSG00000105499	1.44864555	0.000263707
ENSG00000105810	1.517624742	1.78E-07
ENSG00000105825	1.338726666	6.40E-10
ENSG00000105851	-1.631742022	1.64E-11
ENSG00000105855	1.755901786	3.90E-09
ENSG00000105887	1.237269219	3.90E-05
ENSG00000105939	1.258394637	9.97E-08
ENSG00000106070	1.207487147	0.000188771
ENSG00000106100	-1.279987692	2.64E-05
ENSG00000106236	-1.321795592	0.017787104
ENSG00000106366	1.288470157	8.15E-09
ENSG00000106462	-1.212972955	0.015620976
ENSG00000106537	1.446012495	4.08E-08
ENSG00000106560	-1.289670124	0.001185131
ENSG00000106692	1.20008713	0.040466119
ENSG00000106829	-1.275250509	5.07E-07

Gene ID	FC 0NG - 8NG	adj p-value 0NG - 8NG
ENSG00000106852	-1.512432961	6.47E-06
ENSG00000107282	1.305700163	0.010657234
ENSG00000107438	-1.225033362	3.21E-06
ENSG00000107560	1.231674141	0.00525819
ENSG00000107562	3.606511158	2.06E-20
ENSG00000107719	-1.452012658	1.11E-05
ENSG00000107731	-1.390283263	0.003214814
ENSG00000107897	1.205576627	6.86E-05
ENSG00000107984	1.360604695	0.000170739
ENSG00000108106	-1.29156937	3.90E-05
ENSG00000108551	1.362428016	0.000085459
ENSG00000108556	1.268147025	0.015443745
ENSG00000108622	-1.25038798	1.29E-08
ENSG00000108691	-1.872176015	5.69E-07
ENSG00000109062	-1.217252605	2.75E-05
ENSG00000109270	1.239817144	0.002851107
ENSG00000109452	-1.317626752	0.001692733
ENSG00000109458	-1.466451452	5.18E-06
ENSG00000109501	-1.57407614	1.35E-09
ENSG00000109685	-1.245164398	7.58E-08
ENSG00000109805	-1.431469414	0.000327501
ENSG00000109881	-1.258181243	0.000251549
ENSG00000109906	1.960787285	4.02E-09
ENSG00000109944	1.233274421	0.004730581
ENSG00000110092	1.270368011	4.16E-07
ENSG00000110318	1.300849927	0.023208627
ENSG00000110330	1.320847238	0.000836089
ENSG00000110675	1.285751282	0.013557923
ENSG00000110921	-1.248897652	0.000522167
ENSG00000111057	-1.41393552	9.80E-09
ENSG00000111145	-1.267723951	2.28E-09
ENSG00000111206	-1.461419127	3.94E-11

Gene ID	FC 0NG - 8NG	adj p-value 0NG - 8NG
ENSG00000111224	1.243695109	0.005545536
ENSG00000111331	-1.37589247	2.26E-07
ENSG00000111335	-1.405765767	0.002669655
ENSG00000111339	1.660978213	1.28E-09
ENSG00000111602	-1.319717404	2.74E-06
ENSG00000111665	-1.715404282	2.44E-07
ENSG00000111727	1.2957968	0.000671489
ENSG00000111801	-1.411090972	0.00037064
ENSG00000111859	1.301539286	0.010318472
ENSG00000112137	-1.289214901	8.43E-05
ENSG00000112297	-1.637131091	1.53E-07
ENSG00000112742	-1.483386719	0.003190334
ENSG00000112773	1.469592282	0.024686847
ENSG00000112874	1.261548323	0.025262724
ENSG00000112972	-1.313188886	0.017031537
ENSG00000112983	-1.30851357	4.07E-07
ENSG00000112984	-1.695563	7.27E-12
ENSG00000113083	-1.222083085	0.007257793
ENSG00000113161	-1.211417903	0.034427226
ENSG00000113319	1.214612393	0.001962615
ENSG00000113368	-1.349566818	1.31E-08
ENSG00000113441	1.231687589	0.016001727
ENSG00000113522	1.228130311	1.47E-05
ENSG00000113555	-2.145830931	3.95E-10
ENSG00000113739	1.262615538	0.003862034
ENSG00000113810	-1.291301815	0.009389303
ENSG00000114480	1.391561138	6.06E-11
ENSG00000114573	1.262267142	3.48E-06
ENSG00000114796	-1.256796411	0.007852731
ENSG00000114812	-1.684136931	9.11E-05
ENSG00000115084	1.276398561	0.001104172
ENSG00000115091	1.243593868	5.09E-08

Gene ID	FC 0NG - 8NG	adj p-value 0NG - 8NG
ENSG00000115163	-1.638785659	6.06E-08
ENSG00000115290	1.299698939	0.00094484
ENSG00000115525	-2.033722787	7.71E-10
ENSG00000115594	1.221686752	0.001704158
ENSG00000115602	2.704543027	1.07E-09
ENSG00000115604	1.369059561	0.000601365
ENSG00000115641	-1.276934253	1.04E-06
ENSG00000115758	1.228724365	4.72E-06
ENSG00000115902	-1.319313319	1.50E-06
ENSG00000116062	1.250806382	5.76E-11
ENSG00000116473	1.265830597	1.97E-08
ENSG00000116514	-1.224251541	0.000100031
ENSG00000116641	1.223739349	0.006353553
ENSG00000116711	2.218005755	1.73E-09
ENSG00000116747	1.256790872	0.04001323
ENSG00000116991	-1.496325681	9.54E-09
ENSG00000117143	1.236440583	1.27E-05
ENSG00000117152	1.328314902	4.79E-05
ENSG00000117318	1.337142224	1.67E-05
ENSG00000117399	-1.667753284	1.04E-09
ENSG00000117595	1.759255703	4.58E-05
ENSG00000117632	-1.267780772	2.86E-09
ENSG00000117650	-1.732989218	9.08E-10
ENSG00000117724	-1.649666045	2.34E-09
ENSG00000118193	-1.580834026	2.39E-07
ENSG00000118263	1.202308636	0.001438819
ENSG00000118515	1.405168701	2.87E-13
ENSG00000118523	1.38858872	1.26E-12
ENSG00000118600	1.205447387	0.00897763
ENSG00000118777	-1.238713125	0.030868379
ENSG00000118855	1.211305339	0.007105316
ENSG00000118939	1.262798745	0.045308298

Gene ID	FC 0NG - 8NG	adj p-value 0NG - 8NG
ENSG00000118946	2.874475511	1.49E-07
ENSG00000118965	1.259228247	8.45E-05
ENSG00000118985	1.312633567	6.22E-07
ENSG00000119138	1.356284492	4.25E-07
ENSG00000119333	-1.222265018	2.12E-05
ENSG00000119397	-1.353658459	9.95E-07
ENSG00000119403	-1.40766175	1.22E-09
ENSG00000119522	-1.215241798	0.000294027
ENSG00000119630	-1.555708752	5.19E-09
ENSG00000119718	-1.261658899	4.64E-07
ENSG00000119729	1.29531685	0.016977578
ENSG00000119917	-1.276080171	0.047606919
ENSG00000119927	1.226345248	0.003188531
ENSG00000119938	1.356343963	0.002309248
ENSG00000120217	1.496893281	3.86E-08
ENSG00000120334	-1.259255048	0.010425878
ENSG00000120457	-1.309333751	0.00033845
ENSG00000120647	-1.275594659	3.62E-05
ENSG00000120693	1.414729046	0.000302293
ENSG00000120742	1.221220299	0.02420319
ENSG00000120800	1.275357396	1.82E-05
ENSG00000120802	-1.206291601	0.000693969
ENSG00000121152	-1.476309979	9.56E-06
ENSG00000121350	1.265231567	0.0324104
ENSG00000121579	1.286622444	0.023092292
ENSG00000121621	-1.307644895	0.040316375
ENSG00000121858	-2.135838145	1.43E-08
ENSG00000121957	-1.665677945	3.32E-10
ENSG00000121966	-1.393632296	1.24E-05
ENSG00000122376	1.207339105	0.04386161
ENSG00000122378	-1.323339287	8.51E-11
ENSG00000122386	-1.274671575	0.003473992

Gene ID	FC 0NG - 8NG	adj p-value 0NG - 8NG
ENSG00000122641	1.603770985	2.33E-07
ENSG00000122786	1.291361501	3.34E-10
ENSG00000122861	-1.570903647	7.35E-08
ENSG00000122952	-1.231303261	0.000227663
ENSG00000122966	-1.648585144	1.31E-10
ENSG00000123080	-1.624239042	9.65E-10
ENSG00000123091	1.200666768	0.00293836
ENSG00000123095	1.296246885	0.040689122
ENSG00000123384	-1.221644081	0.038727628
ENSG00000123473	-1.239137146	0.003481703
ENSG00000123485	-1.712509569	1.28E-06
ENSG00000123975	-1.382702633	1.76E-05
ENSG00000124120	1.209691608	2.98E-06
ENSG00000124216	1.276366226	0.007801779
ENSG00000124575	-1.329970953	0.000484876
ENSG00000124635	-1.314568083	0.009913104
ENSG00000124772	1.478860069	7.65E-05
ENSG00000124802	1.442004397	0.026435828
ENSG00000125266	1.590219047	6.66E-08
ENSG00000125347	-1.424465497	0.000739021
ENSG00000125378	1.435557357	6.53E-05
ENSG00000125772	1.20134201	0.049901609
ENSG00000125966	1.352052945	0.004980483
ENSG00000125968	1.69836564	2.14E-05
ENSG00000126070	1.217746015	0.01450204
ENSG00000126351	-1.265941383	1.10E-05
ENSG00000126453	-1.302598107	0.001603971
ENSG00000126562	1.554534223	2.33E-08
ENSG00000126787	-1.362617273	0.002244863
ENSG00000126822	-1.266039991	0.007866787
ENSG00000126878	-1.455416793	9.04E-08
ENSG00000127314	1.282558681	0.010068025

Gene ID	FC 0NG - 8NG	adj p-value 0NG - 8NG
ENSG00000127528	1.469217535	5.14E-05
ENSG00000127533	-2.119928352	2.09E-11
ENSG00000128059	1.204141836	0.021519718
ENSG00000128203	-1.220128066	0.045547894
ENSG00000128284	-1.257883974	0.006785997
ENSG00000128294	-1.359868048	1.69E-13
ENSG00000128309	-1.223821971	1.36E-05
ENSG00000128567	1.410570612	3.24E-10
ENSG00000128641	-1.618703	6.67E-10
ENSG00000128654	1.204942352	0.000468215
ENSG00000128815	-1.281878093	0.005100648
ENSG00000128833	-1.856290846	3.35E-10
ENSG00000128849	-1.552536455	2.05E-11
ENSG00000128917	-1.747474066	9.75E-07
ENSG00000128944	-1.483113913	4.27E-09
ENSG00000129128	1.209275552	0.045989401
ENSG00000129195	-1.561845632	9.54E-08
ENSG00000129484	-1.241175165	0.000500455
ENSG00000129493	1.206172188	0.001419397
ENSG00000129810	-1.483066178	7.69E-06
ENSG00000130024	-1.385067598	1.73E-06
ENSG00000130164	-1.277685032	0.000222905
ENSG00000130270	-1.410121612	1.71E-05
ENSG00000130300	1.34661576	0.048723057
ENSG00000130433	1.389946381	0.004720938
ENSG00000130590	-1.245669816	0.028924038
ENSG00000130669	-1.249895506	0.00010334
ENSG00000130775	-1.210907597	0.025259634
ENSG00000130787	-1.226860345	5.33E-05
ENSG00000130830	-1.253492608	6.14E-06
ENSG00000131016	1.39294606	2.83E-10
ENSG00000131037	-1.202465887	0.038766938

Gene ID	FC 0NG - 8NG	adj p-value 0NG - 8NG
ENSG00000131370	-1.558813991	7.57E-14
ENSG00000131409	2.018621325	0.000140022
ENSG00000131634	1.218705123	0.003202229
ENSG00000131652	-1.369091879	1.10E-05
ENSG00000131669	-1.425659039	2.27E-09
ENSG00000131724	1.37421859	1.58E-06
ENSG00000131747	-1.609131146	2.73E-07
ENSG00000131831	1.231907178	0.038120329
ENSG00000132003	-1.50489918	5.63E-08
ENSG00000132196	-1.219609103	0.002237902
ENSG00000132432	1.258647496	0.008548608
ENSG00000132470	-1.22782249	0.036543045
ENSG00000132510	-1.207257679	0.009703365
ENSG00000132563	-1.355222957	0.002261751
ENSG00000132622	-1.242003721	0.009370655
ENSG00000132623	1.234793933	0.034774915
ENSG00000132640	1.206102525	9.33E-05
ENSG00000132950	1.252117607	0.01147885
ENSG00000132963	1.234277348	0.011154078
ENSG00000133056	-1.265478442	0.002416196
ENSG00000133107	1.290513489	0.023659043
ENSG00000133110	1.206313688	0.002874506
ENSG00000133302	1.322579579	0.045861798
ENSG00000133424	1.494630506	1.55E-05
ENSG00000133639	-1.229161287	0.000140634
ENSG00000133657	1.677627613	1.17E-06
ENSG00000133789	1.246709574	7.38E-07
ENSG00000133800	-1.54029973	0.000109899
ENSG00000134057	-1.568995691	5.33E-09
ENSG00000134107	-1.441584438	1.02E-08
ENSG00000134222	-1.877285035	2.59E-09
ENSG00000134291	-1.352493923	2.36E-06

Gene ID	FC 0NG - 8NG	adj p-value 0NG - 8NG
ENSG00000134294	1.377996227	0.008920142
ENSG00000134324	-1.300297781	1.10E-08
ENSG00000134352	1.449700311	6.94E-06
ENSG00000134363	1.367116657	0.000204574
ENSG00000134508	-1.377799355	4.28E-05
ENSG00000134574	-1.330790064	4.74E-09
ENSG00000134668	1.20357238	0.035083103
ENSG00000134690	-1.708510204	9.42E-09
ENSG00000134769	1.233629954	0.001695401
ENSG00000134802	-1.251576527	5.13E-05
ENSG00000134982	1.203293691	0.000509613
ENSG00000135069	-1.418929	9.21E-12
ENSG00000135074	-1.599098189	5.35E-10
ENSG00000135272	-1.268389672	0.000836265
ENSG00000135363	-1.221620515	0.01411364
ENSG00000135451	-1.624184299	5.47E-06
ENSG00000135476	-1.689697422	1.46E-06
ENSG00000135480	1.21694972	0.012271787
ENSG00000135537	1.25806523	0.009245527
ENSG00000135709	1.300254048	0.000087928
ENSG00000135842	-1.342682066	1.60E-06
ENSG00000135913	1.277207616	0.000326738
ENSG00000135966	-1.25812599	2.46E-05
ENSG00000136108	-1.401709534	0.000217926
ENSG00000136122	-1.286976764	0.002767269
ENSG00000136153	-1.291009365	2.27E-06
ENSG00000136205	-1.237590316	0.000226243
ENSG00000136237	-1.235525401	0.002971851
ENSG00000136244	1.403447803	0.00803904
ENSG00000136379	-1.268348707	0.001204472
ENSG00000136630	-1.412778898	0.009932897
ENSG00000136717	-1.367000378	5.71E-07

Gene ID	FC 0NG - 8NG	adj p-value 0NG - 8NG
ENSG00000136824	-1.225180811	0.002688909
ENSG00000136826	1.290399405	0.011342336
ENSG00000136859	-1.353482346	2.99E-08
ENSG00000136861	-1.329369684	1.37E-07
ENSG00000137135	-1.437006515	3.92E-05
ENSG00000137177	1.265372956	4.26E-08
ENSG00000137216	-1.229562561	0.004902699
ENSG00000137393	1.271825956	0.000431854
ENSG00000137486	-1.320305363	1.05E-06
ENSG00000137492	1.21789891	0.045106191
ENSG00000137497	-1.223419944	5.57E-08
ENSG00000137522	1.281477486	2.05E-05
ENSG00000137727	1.264109531	0.008153837
ENSG00000137767	-1.206884109	0.044795788
ENSG00000137801	1.248711939	1.22E-06
ENSG00000137804	-1.665186992	1.07E-09
ENSG00000137807	-1.618144219	2.05E-09
ENSG00000137812	-1.464087609	0.000344582
ENSG00000137825	-1.27882993	0.004141808
ENSG00000137831	1.580993688	3.07E-08
ENSG00000137834	1.860158038	0.00010129
ENSG00000137872	1.591946162	3.69E-06
ENSG00000137880	-1.425095244	0.00274693
ENSG00000138018	1.235474329	0.045663849
ENSG00000138092	-1.203199688	0.002100529
ENSG00000138101	-1.213410553	0.019504112
ENSG00000138160	-1.372339387	0.00146152
ENSG00000138162	-1.388956341	2.13E-05
ENSG00000138180	-1.46492716	4.43E-06
ENSG00000138347	-1.2918374	0.043703835
ENSG00000138386	-1.253657471	0.000117094
ENSG00000138411	1.299974979	1.27E-06

Gene ID	FC 0NG - 8NG	adj p-value 0NG - 8NG
ENSG00000138448	1.421765367	2.26E-06
ENSG00000138449	1.286029818	0.000482842
ENSG00000138496	-1.250231693	0.013325855
ENSG00000138600	1.217087185	0.01892064
ENSG00000138639	1.216180875	1.19E-06
ENSG00000138675	1.331034991	0.004381074
ENSG00000138685	1.325280676	0.000457025
ENSG00000138764	-1.379807175	0.003879595
ENSG00000138771	1.234659405	0.016464201
ENSG00000139132	1.228323705	0.015218993
ENSG00000139211	-1.314814101	0.003626697
ENSG00000139278	1.466823876	5.31E-09
ENSG00000139354	-1.525106799	3.28E-07
ENSG00000139734	-1.300932038	0.000313819
ENSG00000139998	-1.318957913	0.000206012
ENSG00000140416	1.539337986	1.93E-16
ENSG00000140451	-1.873860842	1.23E-07
ENSG00000140534	-1.383643625	0.000424045
ENSG00000140564	1.279707368	1.12E-06
ENSG00000140678	-1.213232139	0.035522165
ENSG00000140853	-1.354588317	0.000256934
ENSG00000140873	-1.434280644	1.05E-05
ENSG00000140961	1.227238806	0.002513308
ENSG00000141526	1.356494593	3.49E-09
ENSG00000141542	1.290224071	0.000907867
ENSG00000141668	2.688624582	4.43E-09
ENSG00000141858	1.265822069	1.50E-05
ENSG00000142408	1.218553897	0.027169017
ENSG00000142494	-1.22611277	0.021848742
ENSG00000142661	1.499557952	0.000158015
ENSG00000142731	-1.25070002	0.040278801
ENSG00000142875	1.265777846	0.000953354

Gene ID	FC 0NG - 8NG	adj p-value 0NG - 8NG
ENSG00000142945	-1.658778769	8.33E-08
ENSG00000143153	1.654965764	1.11E-12
ENSG00000143228	-1.511865205	2.05E-05
ENSG00000143469	1.353335676	0.014663519
ENSG00000143476	1.319018976	0.005379318
ENSG00000143507	-1.381928448	0.000043818
ENSG00000143643	-1.204705043	0.006515298
ENSG00000143669	1.258437384	0.014876707
ENSG00000143772	-1.470516951	1.61E-08
ENSG00000143816	-1.703354262	5.46E-05
ENSG00000143850	-1.314530674	0.000326533
ENSG00000144063	1.679101922	1.23E-12
ENSG00000144320	1.325838755	2.96E-05
ENSG00000144354	1.243257751	0.002591524
ENSG00000144366	1.404449173	2.69E-05
ENSG00000144426	1.374360041	0.000377196
ENSG00000144554	-1.316271857	0.000226646
ENSG00000144560	-1.288094707	9.63E-08
ENSG00000144642	1.35701677	4.63E-06
ENSG00000144644	1.585930129	8.28E-05
ENSG00000144645	-1.284872101	2.73E-08
ENSG00000144677	-1.203899457	0.000260385
ENSG00000144749	-1.510890313	3.53E-09
ENSG00000144824	-1.322751501	0.000016821
ENSG00000144843	1.245425346	0.021543568
ENSG00000145012	1.23375607	0.027060358
ENSG00000145365	-1.295221999	0.001235959
ENSG00000145386	-1.513470346	3.38E-08
ENSG00000145723	1.232052793	0.043389865
ENSG00000145725	1.236086143	0.036707684
ENSG00000145740	1.226916883	0.007198253
ENSG00000145817	1.274657653	0.000537754

Gene ID	FC 0NG - 8NG	adj p-value 0NG - 8NG
ENSG00000145911	-1.430785813	3.48E-05
ENSG00000145916	-1.239943709	0.001541988
ENSG00000146021	-1.274649862	0.002917801
ENSG00000146083	-1.293584016	2.52E-06
ENSG00000146094	-1.286011933	0.047586535
ENSG00000146374	1.454544824	0.003778645
ENSG00000146386	1.296021441	3.08E-07
ENSG00000146410	-1.289261338	0.018776013
ENSG00000146587	1.387206251	0.000372157
ENSG00000146670	-1.302979066	0.004256684
ENSG00000146678	1.246172087	0.01495613
ENSG00000146918	-1.388516292	9.65E-07
ENSG00000146950	-1.306965781	2.12E-06
ENSG00000147082	1.263678344	0.001988054
ENSG00000147119	-1.237618504	0.001197811
ENSG00000147202	1.325167721	0.002348063
ENSG00000147408	-1.615777526	2.02E-07
ENSG00000147536	-1.233761361	0.008399425
ENSG00000147852	-1.692698951	4.48E-07
ENSG00000148677	1.745326345	3.93E-14
ENSG00000148700	-1.395323735	1.07E-07
ENSG00000148773	-1.709976608	1.75E-09
ENSG00000148926	-1.542622662	1.10E-05
ENSG00000149503	-1.609831414	3.01E-09
ENSG00000149531	1.252445879	0.005895102
ENSG00000149564	-1.246279213	2.51E-07
ENSG00000149591	1.519482762	3.23E-07
ENSG00000149929	-1.242901691	0.008940923
ENSG00000149948	1.546443462	8.82E-08
ENSG00000150551	1.264200525	0.001526544
ENSG00000150630	1.246092047	0.000820339
ENSG00000150764	1.40847547	4.21E-06

Gene ID	FC 0NG - 8NG	adj p-value 0NG - 8NG
ENSG00000150787	1.237528989	0.001154882
ENSG00000151012	1.522786036	1.21E-05
ENSG00000151023	1.478888401	0.000608944
ENSG00000151093	1.239490751	0.022964061
ENSG00000151247	1.250555726	0.000727469
ENSG00000151276	-1.290911541	0.000341403
ENSG00000151322	1.215949726	0.033243674
ENSG00000151338	1.358497104	8.85E-05
ENSG00000151414	1.553830884	1.88E-06
ENSG00000151422	1.236275553	0.004076655
ENSG00000151458	1.352241346	2.03E-05
ENSG00000151623	1.276800799	0.014748661
ENSG00000151690	-1.414602356	0.000249762
ENSG00000151692	-1.319018578	0.017166916
ENSG00000151725	-1.309271056	0.000674818
ENSG00000151773	1.428979155	0.008396753
ENSG00000151835	1.396775709	0.006001293
ENSG00000151881	1.277268367	0.012707457
ENSG00000151967	-1.500678332	4.36E-05
ENSG00000152127	-1.299128704	1.10E-10
ENSG00000152256	1.22406452	0.000727795
ENSG00000152284	-1.427302367	2.91E-07
ENSG00000152332	1.231264658	0.025203659
ENSG00000152404	-1.203217052	0.000644583
ENSG00000152465	1.287684761	1.51E-06
ENSG00000152558	1.261131246	0.001183214
ENSG00000152601	1.435510611	1.20E-08
ENSG00000152689	1.330195286	0.000635284
ENSG00000152953	-1.650489254	6.35E-11
ENSG00000153029	1.212086918	0.023021976
ENSG00000153048	-1.217528996	0.001717613
ENSG00000153094	-1.791358605	7.33E-07

Gene ID	FC 0NG - 8NG	adj p-value 0NG - 8NG
ENSG00000153208	-1.425795011	3.81E-06
ENSG00000153443	-1.277110481	0.001674133
ENSG00000153487	-1.262529189	0.000132959
ENSG00000153551	-1.233753284	9.54E-05
ENSG00000153707	1.461410549	0.000883457
ENSG00000153721	1.280029615	0.003814583
ENSG00000153814	1.233688233	0.004455258
ENSG00000153956	1.22706266	0.00221955
ENSG00000153993	1.502180088	2.04E-05
ENSG00000154114	1.210881978	0.000237232
ENSG00000154310	1.320495248	9.93E-10
ENSG00000154380	1.217275322	1.29E-07
ENSG00000154556	1.627330743	1.67E-07
ENSG00000154639	-1.511045099	0.000308902
ENSG00000154734	1.626355318	0.00020943
ENSG00000154864	-1.284874268	1.30E-05
ENSG00000155008	1.205911419	0.038494822
ENSG00000155111	-1.351551163	0.000559439
ENSG00000155158	-1.231375411	0.023849881
ENSG00000155307	1.259849308	0.03423548
ENSG00000155465	-1.251959867	0.000275436
ENSG00000155744	1.219388952	0.009429252
ENSG00000155846	1.354696983	0.001088717
ENSG00000155849	-1.287397351	2.07E-11
ENSG00000155850	1.263443485	0.000175857
ENSG00000155966	1.281229163	0.00929088
ENSG00000156265	1.26991076	0.007256706
ENSG00000156313	1.386616224	6.67E-08
ENSG00000156463	-1.372800009	0.002953304
ENSG00000156466	-1.34998619	0.017059033
ENSG00000156509	-1.310498365	0.002572498
ENSG00000156587	-1.221547705	0.001085395

Gene ID	FC 0NG - 8NG	adj p-value 0NG - 8NG
ENSG00000156970	-1.628580012	1.68E-08
ENSG00000157168	1.413524458	8.96E-09
ENSG00000157214	1.541261679	1.87E-06
ENSG00000157227	-1.289968604	6.28E-07
ENSG00000157240	1.291948403	0.00940072
ENSG00000157404	-2.184445474	3.94E-08
ENSG00000157456	-1.601569248	3.51E-10
ENSG00000157510	-1.325013398	1.41E-09
ENSG00000157570	-1.409622263	2.63E-11
ENSG00000157600	1.203048406	0.000186244
ENSG00000157613	-2.665413102	2.20E-10
ENSG00000158106	-1.351075952	0.019283992
ENSG00000158122	1.205475499	0.000223812
ENSG00000158186	1.279783849	0.00153262
ENSG00000158195	-1.222725769	3.24E-05
ENSG00000158270	-1.280046648	0.00651192
ENSG00000158352	1.253019968	1.43E-06
ENSG00000158373	-1.392480209	0.000147436
ENSG00000158402	-1.737718949	8.96E-10
ENSG00000158406	-1.390846819	3.19E-05
ENSG00000158555	-1.512145513	6.54E-13
ENSG00000158715	-1.216860693	0.009325919
ENSG00000158716	1.283039012	0.001215765
ENSG00000158856	-1.378331822	5.44E-07
ENSG00000158859	-1.352379025	0.000018538
ENSG00000159167	-1.774049768	6.80E-06
ENSG00000159231	-1.298857227	1.49E-05
ENSG00000159388	-1.228222783	0.000107789
ENSG00000159399	1.25555912	1.35E-06
ENSG00000159433	-1.236661202	0.016191288
ENSG00000159733	1.34617278	0.009036374
ENSG00000160013	-1.914764776	3.54E-08

Gene ID	FC 0NG - 8NG	adj p-value 0NG - 8NG
ENSG00000160111	-1.208526777	0.044332235
ENSG00000160179	-1.477341885	3.48E-05
ENSG00000160298	-1.31820766	0.03603482
ENSG00000160299	-1.271998322	4.99E-06
ENSG00000160447	-1.311768695	3.99E-07
ENSG00000160539	-1.396097665	0.002914656
ENSG00000160588	1.275406854	0.016132398
ENSG00000160796	-1.367544442	2.40E-07
ENSG00000161542	-1.242700813	3.88E-09
ENSG00000161692	-1.326863434	0.001298754
ENSG00000161800	-1.610314647	3.24E-09
ENSG00000161813	1.299987049	1.42E-05
ENSG00000161888	-1.430073421	9.30E-07
ENSG00000161940	-1.339259807	2.85E-09
ENSG00000162032	-1.221564955	0.003323762
ENSG00000162063	-1.573454708	1.64E-07
ENSG00000162073	1.234915942	0.00015408
ENSG00000162337	-1.276044967	7.07E-06
ENSG00000162426	-1.263215852	0.024037485
ENSG00000162434	1.395099181	4.94E-13
ENSG00000162437	1.215645039	0.006532682
ENSG00000162496	-1.676355654	4.30E-08
ENSG00000162551	1.220571654	0.030248988
ENSG00000162614	1.564989687	1.19E-08
ENSG00000162616	1.3926787	0.000415078
ENSG00000162618	1.2641584	0.036441296
ENSG00000162692	1.780381895	2.91E-06
ENSG00000162695	1.26837716	0.018158602
ENSG00000162746	1.284597477	0.010217742
ENSG00000163083	-1.422179116	0.000585508
ENSG00000163110	1.537008448	1.79E-17
ENSG00000163346	-1.298589752	0.000145959

Gene ID	FC 0NG - 8NG	adj p-value 0NG - 8NG
ENSG00000163428	1.308261393	0.002357512
ENSG00000163527	1.251030153	0.001095417
ENSG00000163554	-1.371565607	0.004738757
ENSG00000163633	1.242373346	0.008929051
ENSG00000163661	1.427251126	2.92E-06
ENSG00000163814	-1.246105765	0.000054849
ENSG00000163947	-1.215695748	0.004179058
ENSG00000163950	1.237232028	0.000024239
ENSG00000164023	1.386004605	7.94E-06
ENSG00000164035	1.268582317	0.031904933
ENSG00000164038	1.215055975	8.27E-05
ENSG00000164074	1.248413241	0.001015249
ENSG00000164087	-1.486884665	3.57E-06
ENSG00000164104	-1.641606078	1.58E-09
ENSG00000164105	-1.345947646	0.000026857
ENSG00000164109	-1.264672156	0.014575115
ENSG00000164134	1.217626563	0.024056025
ENSG00000164161	1.524083766	1.08E-11
ENSG00000164209	1.296258394	0.007296004
ENSG00000164236	1.343880171	0.000343564
ENSG00000164283	1.671149423	9.15E-10
ENSG00000164292	1.473245825	1.60E-09
ENSG00000164294	1.201157846	0.006949923
ENSG00000164442	1.342679065	0.002293534
ENSG00000164611	-1.319311229	1.84E-07
ENSG00000164736	1.247901924	0.000081012
ENSG00000164754	-1.240738169	4.14E-08
ENSG00000164877	-1.222929495	0.03388258
ENSG00000164951	1.298480933	1.91E-05
ENSG00000164985	-1.212469152	0.000180535
ENSG00000165046	1.256206724	0.007991548
ENSG00000165097	1.277954316	0.000112862

Gene ID	FC 0NG - 8NG	adj p-value 0NG - 8NG
ENSG00000165118	1.280933398	0.001773521
ENSG00000165240	1.213457772	0.009793964
ENSG00000165244	1.504401985	8.81E-06
ENSG00000165271	1.237199804	0.000011491
ENSG00000165272	-1.346559487	0.001931199
ENSG00000165304	-1.341733442	1.81E-06
ENSG00000165338	1.33716886	0.012797055
ENSG00000165480	-1.289367065	0.014534709
ENSG00000165507	-1.54493486	0.002889182
ENSG00000165806	-1.265292525	1.23E-10
ENSG00000165886	-1.39964984	3.18E-09
ENSG00000165899	1.455754765	0.000011877
ENSG00000165912	-1.226883805	0.003507446
ENSG00000165959	-1.602683825	1.73E-10
ENSG00000166073	1.239879649	0.000298249
ENSG00000166250	1.298021407	0.003435229
ENSG00000166347	-1.237359249	0.010224155
ENSG00000166439	-1.202524913	4.50E-05
ENSG00000166503	1.285259515	1.89E-05
ENSG00000166510	1.751108973	4.83E-06
ENSG00000166801	-1.273788508	0.001682454
ENSG00000166803	-1.228299159	0.003285065
ENSG00000166833	-1.213541781	0.002029499
ENSG00000166851	-1.686352909	1.60E-08
ENSG00000166912	1.391980573	0.005058079
ENSG00000167106	-1.489052888	3.03E-08
ENSG00000167191	-1.557403082	2.58E-06
ENSG00000167220	1.208522458	0.022676981
ENSG00000167508	-1.231297312	0.004510952
ENSG00000167550	-1.319099116	0.001837943
ENSG00000167680	-1.393947059	2.15E-08
ENSG00000167703	1.308657755	0.004724065

Gene ID	FC 0NG - 8NG	adj p-value 0NG - 8NG
ENSG00000167772	1.411375043	0.001841527
ENSG00000167861	-1.226546031	0.006629545
ENSG00000167900	-1.247123937	0.000589054
ENSG00000168077	-1.222348461	2.73E-05
ENSG00000168078	-1.424786149	0.000289955
ENSG00000168298	-1.441636419	8.49E-07
ENSG00000168309	-1.381857956	2.83E-06
ENSG00000168374	1.220752889	1.35E-06
ENSG00000168386	1.263672663	0.027784631
ENSG00000168393	-1.211479139	1.30E-05
ENSG00000168476	-1.41341158	7.63E-09
ENSG00000168887	-1.216823516	0.002537761
ENSG00000169083	-1.309604442	2.23E-06
ENSG00000169242	-1.394616443	0.009306751
ENSG00000169499	1.218013231	0.000627506
ENSG00000169504	1.319306522	0.001725644
ENSG00000169604	-1.235099251	0.000562977
ENSG00000169607	-1.553025872	4.56E-06
ENSG00000169679	-1.60537956	6.64E-09
ENSG00000169715	1.310890007	0.000156361
ENSG00000169744	1.235741985	5.92E-05
ENSG00000169760	1.481454738	1.04E-06
ENSG00000169851	1.281214547	0.015726338
ENSG00000169914	1.325404936	0.000157476
ENSG00000169967	1.203501731	0.019656865
ENSG00000170011	-1.406178666	0.000391629
ENSG00000170017	1.311309815	2.31E-08
ENSG00000170037	-1.213737212	0.000315682
ENSG00000170088	1.224590232	0.002519359
ENSG00000170175	-1.298703261	0.000679396
ENSG00000170260	-1.240616976	0.007664066
ENSG00000170271	-1.517638711	0.000079128

Gene ID	FC 0NG - 8NG	adj p-value 0NG - 8NG
ENSG00000170312	-1.537180846	3.65E-05
ENSG00000170323	1.458344684	0.007117448
ENSG00000170365	-1.426526651	9.60E-08
ENSG00000170385	1.37146405	0.003935116
ENSG00000170421	-1.214415929	0.038003157
ENSG00000170485	-1.317417543	3.41E-09
ENSG00000170525	-1.29879908	4.18E-09
ENSG00000170540	-1.281032194	0.028858797
ENSG00000170558	1.339551426	6.74E-11
ENSG00000170684	1.322162954	0.007713636
ENSG00000170775	1.223396075	0.000304797
ENSG00000170873	-1.237114223	0.001154882
ENSG00000170962	1.264100554	0.041495812
ENSG00000171033	1.219671786	0.001476652
ENSG00000171056	-1.229443153	0.001805386
ENSG00000171241	-1.289733478	0.003423996
ENSG00000171246	2.735299808	3.25E-12
ENSG00000171388	-1.357860505	0.000935702
ENSG00000171488	1.272142972	0.001001053
ENSG00000171608	-1.201168533	0.001807239
ENSG00000171621	-1.354669186	0.014862876
ENSG00000171848	-1.3071588	0.000209628
ENSG00000171889	1.255898823	0.016944478
ENSG00000172031	-1.562823412	1.50E-05
ENSG00000172071	-1.274912873	0.000027335
ENSG00000172086	1.23126825	0.001350201
ENSG00000172159	1.264547638	0.000610752
ENSG00000172380	1.204569193	0.015854883
ENSG00000172985	-1.267387071	0.000221905
ENSG00000173083	1.200807147	0.006059344
ENSG00000173207	-1.505020418	1.42E-09
ENSG00000173456	-1.268582773	6.16E-05

Gene ID	FC 0NG - 8NG	adj p-value 0NG - 8NG
ENSG00000173530	1.267915607	4.24E-06
ENSG00000173535	-1.434244975	1.35E-07
ENSG00000173548	-1.206976447	9.37E-05
ENSG00000173597	1.917466895	2.65E-08
ENSG00000173599	-1.202635104	0.005807013
ENSG00000173801	-1.212652312	0.002781967
ENSG00000173852	1.212409118	0.020144785
ENSG00000173898	-1.205452338	0.000427572
ENSG00000174059	-1.768149161	0.000167488
ENSG00000174306	1.205433679	9.59E-05
ENSG00000174370	-1.489077602	0.000112901
ENSG00000174501	1.311088992	0.044550254
ENSG00000174606	1.243280471	0.006599372
ENSG00000174684	-1.258816465	0.00057938
ENSG00000174695	1.244747033	0.049254515
ENSG00000174705	-1.229057905	2.08E-06
ENSG00000175048	1.262344806	0.000642293
ENSG00000175063	-1.675904234	2.69E-08
ENSG00000175216	-1.248375845	4.05E-07
ENSG00000175264	-1.608957033	6.26E-11
ENSG00000175274	-1.665190577	1.23E-12
ENSG00000175305	1.455186926	0.000591558
ENSG00000175348	1.235934535	1.88E-06
ENSG00000175426	1.318231474	0.003150084
ENSG00000175567	-1.216256058	0.041090182
ENSG00000175643	-1.228479284	0.025426537
ENSG00000175691	-1.216776852	0.001057141
ENSG00000175832	-1.326558049	3.34E-06
ENSG00000175899	1.386127339	0.019253897
ENSG00000175938	-1.314347739	0.006028302
ENSG00000176102	1.326313503	9.09E-06
ENSG00000176194	-1.29909644	0.002996197

Gene ID	FC 0NG - 8NG	adj p-value 0NG - 8NG
ENSG00000176244	-1.227416534	0.042809465
ENSG00000176438	1.23906952	0.000389028
ENSG00000176619	-1.250337485	4.49E-07
ENSG00000176641	1.238856924	0.006812459
ENSG00000176890	-1.316647942	5.74E-07
ENSG00000176974	-1.26125634	1.79E-06
ENSG00000177191	-1.244033359	0.016255727
ENSG00000177200	1.217705747	0.004186156
ENSG00000177303	1.211838124	0.000146499
ENSG00000177337	-1.312742365	0.00291055
ENSG00000177374	-1.419717382	0.000402743
ENSG00000177426	-1.436344427	1.07E-09
ENSG00000177432	1.414848351	0.001317172
ENSG00000177602	-1.26570017	0.018928283
ENSG00000177663	1.203141218	0.000218999
ENSG00000177853	1.34489163	0.012840899
ENSG00000178031	1.31189069	7.19E-08
ENSG00000178445	-1.351992887	0.000110934
ENSG00000178685	-1.204401428	0.020010159
ENSG00000178726	1.204747413	0.010591776
ENSG00000178882	1.655110345	5.17E-06
ENSG00000178904	1.205089665	0.039325317
ENSG00000178999	-1.597206397	6.55E-07
ENSG00000179044	1.454023747	5.05E-05
ENSG00000179094	1.373121213	3.07E-06
ENSG00000179314	-1.56667427	1.33E-08
ENSG00000179403	1.834109298	4.61E-07
ENSG00000179532	1.370089595	0.000515056
ENSG00000179598	1.329847215	0.000299739
ENSG00000179750	-1.35370715	0.000527013
ENSG00000179820	1.300590774	2.99E-10
ENSG00000179833	1.213013929	0.000120513

Gene ID	FC 0NG - 8NG	adj p-value 0NG - 8NG
ENSG00000179941	1.347292961	0.005645962
ENSG00000179981	-1.294011896	1.11E-06
ENSG00000180346	1.263809981	0.03279166
ENSG00000180354	1.308754663	0.006736837
ENSG00000180448	1.312356037	0.034593989
ENSG00000180530	-1.243026092	0.001671369
ENSG00000180537	-1.208405923	0.033891222
ENSG00000180573	-1.212291353	0.007523408
ENSG00000180596	-1.312894712	0.000751832
ENSG00000180730	-1.28020652	0.000962193
ENSG00000180758	1.269562009	0.00339728
ENSG00000181381	1.328521571	0.021889817
ENSG00000181744	1.208252695	0.015641411
ENSG00000181826	-1.232562589	9.04E-10
ENSG00000182022	-1.342771452	1.72E-10
ENSG00000182118	-1.495593533	0.000756923
ENSG00000182134	-1.254040877	0.00143473
ENSG00000182158	-1.279120143	3.47E-17
ENSG00000182247	1.232686198	0.000777568
ENSG00000182253	1.217375888	0.017955175
ENSG00000182378	1.217436009	0.0426795
ENSG00000182481	-1.439228297	8.96E-13
ENSG00000182575	-1.265332947	0.033281248
ENSG00000182621	1.228179765	0.041513611
ENSG00000182704	-1.280692073	1.35E-05
ENSG00000183018	-1.270628381	0.000684259
ENSG00000183044	1.240621168	0.044513471
ENSG00000183049	1.521334095	3.02E-05
ENSG00000183287	1.249590294	0.016031127
ENSG00000183496	-1.622493879	1.97E-07
ENSG00000183578	1.204894212	0.003398345
ENSG00000183580	-1.404781002	2.73E-12

Gene ID	FC 0NG - 8NG	adj p-value 0NG - 8NG
ENSG00000183598	-1.200143329	0.034346384
ENSG00000183688	1.237427718	0.000777028
ENSG00000183765	-1.213431102	0.003619721
ENSG00000183856	-1.616006334	3.83E-09
ENSG00000183873	-1.234605656	0.004145741
ENSG00000184009	1.223632277	6.40E-11
ENSG00000184083	1.230129135	0.00127093
ENSG00000184113	-1.866290851	3.06E-12
ENSG00000184154	-1.257326088	0.012239422
ENSG00000184182	1.222908614	0.032066285
ENSG00000184226	1.315953838	0.00499848
ENSG00000184254	-1.270236605	7.67E-07
ENSG00000184260	-1.317365914	0.000399597
ENSG00000184270	-1.545773961	3.90E-05
ENSG00000184357	-1.436303677	6.16E-05
ENSG00000184384	1.30064809	1.76E-05
ENSG00000184588	-1.223994588	0.000165713
ENSG00000184661	-1.618021821	3.24E-07
ENSG00000184678	-1.315423689	0.000336442
ENSG00000184792	-1.505399374	1.24E-07
ENSG00000184867	1.227318807	0.000731854
ENSG00000184897	-1.33329693	1.33E-08
ENSG00000184916	1.309064615	5.71E-06
ENSG00000184988	1.209682641	0.00634304
ENSG00000184992	1.234806389	0.001252105
ENSG00000185130	-1.346606771	0.001953537
ENSG00000185215	1.23850597	0.003843926
ENSG00000185262	-1.4742794	2.37E-09
ENSG00000185269	1.328818523	0.000141501
ENSG00000185278	1.290319575	0.004096698
ENSG00000185338	-1.544518148	0.000096826
ENSG00000185352	1.650855714	5.95E-07

Gene ID	FC 0NG - 8NG	adj p-value 0NG - 8NG
ENSG00000185361	-1.494119869	9.47E-09
ENSG00000185432	-1.411664787	1.19E-08
ENSG00000185630	-1.366380106	0.000737358
ENSG00000185955	-1.244982829	0.017897133
ENSG00000186130	1.312468597	0.033513659
ENSG00000186185	-1.455665152	8.23E-05
ENSG00000186193	-1.752123742	2.37E-12
ENSG00000186470	-1.235988376	0.000197245
ENSG00000186479	-1.517911382	8.25E-07
ENSG00000186480	-1.435835445	0.000119823
ENSG00000186567	1.354727242	0.000079112
ENSG00000186638	-1.388805056	0.002209328
ENSG00000186642	-1.265914621	0.004347222
ENSG00000186827	-1.214442061	0.026614674
ENSG00000186871	-1.250890324	0.025518291
ENSG00000187118	1.218319672	0.032490441
ENSG00000187189	1.26245567	1.89E-06
ENSG00000187266	-1.501261781	4.43E-08
ENSG00000187398	1.202010773	0.011142947
ENSG00000187498	1.23260508	4.82E-05
ENSG00000187513	-1.477214442	0.041348844
ENSG00000187741	-1.229974578	0.026153958
ENSG00000187764	-1.220122719	0.031636044
ENSG00000187824	1.243268891	0.003766191
ENSG00000187837	-1.402461792	1.66E-05
ENSG00000187840	-1.292216136	8.22E-06
ENSG00000187951	-1.302262107	0.035881059
ENSG00000188042	-1.274476249	0.039085066
ENSG00000188277	-1.860953438	0.000724925
ENSG00000188312	-1.216541045	0.034742639
ENSG00000188428	1.324624363	0.002199033
ENSG00000188486	-1.367550643	5.44E-06

Gene ID	FC 0NG - 8NG	adj p-value 0NG - 8NG
ENSG00000188596	1.395608101	0.000451454
ENSG00000188647	1.215044302	0.030601089
ENSG00000188921	1.279040409	0.049056508
ENSG00000189056	1.252909812	0.015164126
ENSG00000189057	1.567032434	0.000259435
ENSG00000189221	1.260897255	0.002126638
ENSG00000196159	1.280818428	0.005701713
ENSG00000196169	-1.980739916	3.28E-10
ENSG00000196187	-1.260126927	0.001239594
ENSG00000196209	-1.369874795	0.001408347
ENSG00000196421	-1.356344139	0.000262364
ENSG00000196422	-1.208747356	0.020048404
ENSG00000196468	-1.500278605	5.80E-05
ENSG00000196476	-1.203546972	0.021719622
ENSG00000196586	1.205011571	0.001694544
ENSG00000196730	-1.245722611	6.02E-08
ENSG00000196739	-1.229859887	0.001570969
ENSG00000196747	-1.40046598	0.000365657
ENSG00000196787	-1.29666973	0.006690474
ENSG00000196839	-1.370435288	0.000147262
ENSG00000196843	-1.202926633	0.04079137
ENSG00000196866	-1.340803959	0.000203902
ENSG00000196967	1.303699731	0.026817984
ENSG00000197019	1.241846267	0.000561186
ENSG00000197061	-1.415949213	3.73E-05
ENSG00000197238	-1.309116731	0.00022813
ENSG00000197261	-1.420993108	0.000587069
ENSG00000197343	1.282492307	0.007835947
ENSG00000197409	-1.400755512	0.000262669
ENSG00000197461	1.310895097	0.000339921
ENSG00000197498	1.206197783	0.005390405
ENSG00000197530	-1.239688779	0.019722914

Gene ID	FC 0NG - 8NG	adj p-value 0NG - 8NG
ENSG00000197632	1.366691053	0.009109414
ENSG00000197780	1.27158855	0.04602668
ENSG00000197837	-1.235088441	0.001781366
ENSG00000197852	-1.257128633	9.01E-05
ENSG00000197857	1.221015735	0.020562457
ENSG00000197863	1.297143152	0.000907954
ENSG00000197903	-1.241976142	0.024440984
ENSG00000197930	1.201406307	0.041601001
ENSG00000198015	1.21586156	0.016364268
ENSG00000198042	1.247484423	5.47E-06
ENSG00000198108	1.398813376	0.002635226
ENSG00000198331	-1.290469847	0.000513729
ENSG00000198431	1.242384237	0.000319479
ENSG00000198720	-1.231560027	8.32E-05
ENSG00000198794	-1.257627119	0.007779828
ENSG00000198832	1.216418687	0.023519629
ENSG00000198846	1.802749282	4.73E-08
ENSG00000198873	-1.384758337	2.05E-09
ENSG00000198901	-1.584019455	3.08E-10
ENSG00000198909	-1.247023331	6.16E-09
ENSG00000198959	-1.2837903	1.88E-07
ENSG00000203814	-1.41203937	3.24E-06
ENSG00000203883	-1.788915171	4.57E-07
ENSG00000204103	1.252128774	0.031806524
ENSG00000204217	1.380072418	6.18E-06
ENSG00000204406	1.21883938	0.003593904
ENSG00000204442	2.04631577	1.63E-12
ENSG00000204776	1.348128656	0.000790607
ENSG00000205476	-1.30534908	3.45E-06
ENSG00000206417	-1.266043206	0.013239503
ENSG00000206538	1.621479518	5.74E-08
ENSG00000212766	1.27021527	0.000109345

Gene ID	FC 0NG - 8NG	adj p-value 0NG - 8NG
ENSG00000213064	1.212189327	0.00579109
ENSG00000213190	1.240360865	0.001469018
ENSG00000213347	-1.665437158	1.57E-08
ENSG00000213445	-1.22008999	2.15E-05
ENSG00000213853	-1.329487161	2.66E-05
ENSG00000214357	-1.83701173	5.12E-06
ENSG00000214655	-1.268861869	1.98E-05
ENSG00000214960	1.335198234	0.00744372
ENSG00000216775	-1.480653178	1.42E-05
ENSG00000220785	-1.402966264	0.019456229
ENSG00000221829	-1.223294236	0.005057414
ENSG00000221866	1.435028718	0.001424481
ENSG00000221926	-1.232308688	4.63E-05
ENSG00000221963	-1.310729836	0.001017747
ENSG00000225151	-1.252469624	0.015648621
ENSG00000225177	-1.297433682	0.005274157
ENSG00000225830	1.567577147	3.46E-11
ENSG00000226328	1.327132604	0.002396448
ENSG00000226808	1.230174775	0.018303468
ENSG00000227825	1.305088418	0.039682793
ENSG00000228495	1.334647745	7.51E-07
ENSG00000228794	1.241881925	0.004597907
ENSG00000229809	-1.279691	0.016086981
ENSG00000230266	-1.349425712	0.001561854
ENSG00000230487	1.283667009	0.011544705
ENSG00000230753	-1.241433012	0.007153943
ENSG00000232931	1.393083934	0.041513611
ENSG00000233117	1.419418352	0.001435812
ENSG00000233251	1.359892339	6.03E-05
ENSG00000233369	-1.22520747	0.000218668
ENSG00000233370	1.234497889	0.004348189
ENSG00000233450	-1.247555713	0.001083842

Gene ID	FC 0NG - 8NG	adj p-value 0NG - 8NG
ENSG00000233818	-1.224333674	0.001408779
ENSG00000233822	-1.421694458	0.000113167
ENSG00000234373	1.255669742	0.030805243
ENSG00000235217	1.275284366	0.014543329
ENSG00000235770	1.295746217	0.000013304
ENSG00000235823	-1.289363899	0.010200373
ENSG00000235884	1.284597981	0.001620289
ENSG00000239306	1.255696848	5.08E-08
ENSG00000239887	-1.372595214	8.24E-05
ENSG00000240694	-1.684791596	1.91E-14
ENSG00000240891	-1.283392967	0.009902614
ENSG00000242779	1.200974859	0.024162282
ENSG00000249242	1.23457128	0.018363854
ENSG00000249464	1.258090429	0.028867842
ENSG00000249992	-1.302585457	0.000121604
ENSG00000250303	1.344560216	0.000255746
ENSG00000250510	-1.260302231	0.006598099
ENSG00000250899	1.205588138	0.00305936
ENSG00000253368	1.883387943	1.37E-16
ENSG00000253661	1.412483475	0.000163915
ENSG00000254726	-1.247025267	0.000571733
ENSG00000255248	1.401744402	0.000567623
ENSG00000255690	-1.243875922	0.046139036
ENSG00000256304	-1.204043845	0.023680884
ENSG00000258441	1.467411573	1.09E-05
ENSG00000259330	1.250089845	0.008105809
ENSG00000259518	-1.383291833	0.00829695
ENSG00000260604	1.208585058	0.001872887
ENSG00000260766	1.310540604	0.004342969
ENSG00000260804	1.262236299	0.000695414
ENSG00000260920	1.25587368	0.012584036
ENSG00000261253	-1.239193072	0.022300803

Gene ID	FC 0NG - 8NG	adj p-value 0NG - 8NG
ENSG00000261373	1.296594195	0.00005892
ENSG00000261468	1.368592164	0.012008832
ENSG00000262001	-1.418195513	1.08E-05
ENSG00000262580	1.274696626	0.011443486
ENSG00000263155	-1.307465824	0.012345447
ENSG00000263528	1.254690817	8.84E-05
ENSG00000267532	-1.254569495	0.013753351
ENSG00000267583	-1.439134538	7.58E-09
ENSG00000270547	-1.271029009	0.036427776
ENSG00000271590	1.268233716	0.001690377
ENSG00000272455	1.262645852	0.030672946
ENSG00000273703	-1.265481484	0.029074469
ENSG00000273802	-1.310827198	0.001284303
ENSG00000273850	-1.253637472	0.022748039
ENSG00000273983	-1.347324678	0.001697564
ENSG00000274180	-1.268393556	0.022678693
ENSG00000274267	-1.409010344	0.000613199
ENSG00000274290	-1.401694883	3.92E-05
ENSG00000274618	-1.349918134	0.005005891
ENSG00000274641	-1.369764043	0.001754978
ENSG00000274997	-1.329463799	0.006752241
ENSG00000275126	-1.426402323	8.10E-05
ENSG00000275379	-1.356494165	0.001897495
ENSG00000275713	-1.573275646	2.72E-05
ENSG00000275714	-1.208932226	0.012205624
ENSG00000276256	1.217815923	0.036748447
ENSG00000276368	-1.348684789	0.002571794
ENSG00000276410	-1.364222494	0.001031194
ENSG00000276849	-1.48489006	0.00480015
ENSG00000276903	-1.381008178	0.002081393
ENSG00000276966	-1.237663579	0.000294937
ENSG00000277075	-1.401223426	8.67E-05

Gene ID	FC 0NG - 8NG	adj p-value 0NG - 8NG
ENSG00000277157	-1.407406071	0.000339342
ENSG00000277224	-1.4075699	0.00024796
ENSG00000277775	-1.813962972	1.80E-08
ENSG00000278272	-1.364951515	0.001145389
ENSG00000278463	-1.298937256	0.005665801
ENSG00000278588	-1.327002401	0.002803789
ENSG00000278637	-1.274217334	0.002212301
ENSG00000278705	-1.273475706	0.001846221
ENSG00000278828	-1.351267677	0.000847165
ENSG00000279095	1.330521613	3.59E-07
ENSG00000279117	-1.314110499	5.84E-07
ENSG00000279348	1.204225054	0.041769895
ENSG00000280128	1.223792923	0.021503967
ENSG00000280145	1.258000342	0.043228456
ENSG00000280173	1.244798942	0.022087724
ENSG00000281005	1.200462046	0.047950873
ENSG00000281840	1.32186875	0.003690557
ENSG00000282057	1.301426214	0.022922767
ENSG00000282105	-1.230190565	0.019629569

Supplementary Table 1e. Differentially expressed genes between 0hr and 24hr normal glucose samples

Ensembl gene IDs, Fold change (baseline-24NG) and FDR adjusted p-values of DEGs

Gene ID	FC 0NG - 24NG	adj p-value 0NG - 24NG
ENSG00000000460	-1.258469565	0.03344643
ENSG00000001630	-1.351088097	0.00016346
ENSG00000002079	-1.296543275	0.033513659
ENSG00000004799	1.30215913	0.007128494
ENSG00000006638	1.230480799	0.024952291
ENSG00000007384	-1.244015682	0.009162901
ENSG00000010292	-1.276921414	8.35E-08
ENSG00000011426	-1.261962926	0.001340883
ENSG00000013293	1.331629757	0.003212047
ENSG00000013588	1.603538248	3.42E-09
ENSG00000013810	-1.355242352	0.000412644
ENSG00000021645	1.362971183	0.000602383
ENSG00000026508	1.203227541	0.000353861
ENSG00000026559	1.323562713	0.002625783
ENSG00000027869	1.277713785	0.048191973
ENSG00000029993	-1.208539801	0.000107651
ENSG00000033867	-1.274865726	0.002292257
ENSG00000035499	-1.422293637	1.27E-05
ENSG00000038295	1.307668085	0.005559969
ENSG00000040608	-1.220694034	0.043847267
ENSG00000047346	1.275470253	0.027384366
ENSG00000050344	1.219329142	8.53E-05
ENSG00000054654	-1.333770262	0.007429509
ENSG00000057019	1.453417392	2.22E-07
ENSG00000058085	-1.329143067	0.001234543
ENSG00000058404	-1.406540356	0.000227723
ENSG00000060656	-1.248851308	0.014610117
ENSG00000064042	1.651496643	4.47E-07
ENSG00000066279	-1.483481312	0.008009943
ENSG00000066923	-1.221159022	0.006092125
ENSG00000067064	-1.344357793	0.005649819
ENSG00000068489	-1.237555134	4.64E-05

Gene ID	FC 0NG - 24NG	adj p-value 0NG - 24NG
ENSG00000069399	1.202712078	0.030886068
ENSG00000069702	1.280381642	0.004291914
ENSG00000070404	1.367712342	1.79E-05
ENSG00000070614	-1.290311594	4.37E-09
ENSG00000070669	-1.339791013	5.16E-05
ENSG00000071073	-1.215711798	0.001222316
ENSG00000071246	-1.308972745	0.01113976
ENSG00000071539	-1.204815673	0.002782936
ENSG00000072071	1.262549979	0.000751832
ENSG00000072195	1.237871282	0.018198655
ENSG00000072274	-1.205012465	0.000106069
ENSG00000073849	1.20446776	7.55E-05
ENSG00000074410	1.422735177	0.000134912
ENSG00000074527	1.266819272	0.011280141
ENSG00000074855	1.250782852	0.010674166
ENSG00000075218	-1.415113888	5.14E-05
ENSG00000075223	1.285047806	0.040818519
ENSG00000076248	1.239767016	5.25E-06
ENSG00000076382	-1.220252677	0.00297505
ENSG00000076662	-1.230629855	0.03518764
ENSG00000077092	1.513647339	3.46E-06
ENSG00000078596	1.223601478	2.29E-05
ENSG00000079385	1.345102721	0.001817091
ENSG00000079459	-1.427999607	2.12E-11
ENSG00000079616	-1.359678896	1.05E-05
ENSG00000080986	-1.390966175	0.00018779
ENSG00000081320	-1.293621424	0.004166923
ENSG00000082126	-1.383466588	4.05E-07
ENSG00000087303	-1.309361655	4.63E-06
ENSG00000087494	1.345151537	0.019184823
ENSG00000087586	-1.331725292	1.95E-05
ENSG00000088280	1.295461599	0.000193832

Gene ID	FC 0NG - 24NG	adj p-value 0NG - 24NG
ENSG00000088320	1.221676009	0.000423419
ENSG00000088325	-1.354048343	0.000013304
ENSG00000088367	1.244136727	0.02921596
ENSG00000089685	-1.268912591	0.000115499
ENSG00000090339	1.470641016	0.005118202
ENSG00000090530	1.422763157	0.000423647
ENSG00000090889	-1.466502527	1.05E-06
ENSG00000091129	1.300994569	6.02E-06
ENSG00000091137	-1.361907029	0.000709079
ENSG00000091409	-1.262144259	6.38E-06
ENSG00000092470	1.201424254	0.033855834
ENSG00000092621	-1.531908752	2.97E-08
ENSG00000092964	-1.229628895	1.17E-12
ENSG00000092969	1.426574382	0.006839021
ENSG00000094916	-1.245726549	2.05E-05
ENSG00000095383	1.314617182	0.0013489
ENSG00000095539	1.356851484	0.000191828
ENSG00000096433	-1.242221538	2.98E-07
ENSG00000099194	-1.241085594	2.65E-07
ENSG00000099251	-1.265699736	0.049909426
ENSG00000099937	1.844522045	2.27E-07
ENSG00000100034	-1.247978831	1.71E-07
ENSG00000100092	1.294417607	0.020664305
ENSG00000100100	1.767719294	6.64E-08
ENSG00000100490	-1.228137527	0.02575039
ENSG00000100526	-1.242961941	0.009992
ENSG00000100629	-1.369255572	0.001081528
ENSG00000100784	1.241109346	0.036074544
ENSG00000100918	1.413988128	2.45E-06
ENSG00000101096	-1.285332426	0.000173692
ENSG00000101224	-1.268200671	0.000046851
ENSG00000101265	-1.315770976	4.56E-05

Gene ID	FC 0NG - 24NG	adj p-value 0NG - 24NG
ENSG00000101335	1.257835601	0.010715636
ENSG00000101447	-1.403589628	1.98E-05
ENSG00000101665	1.451239947	0.018819365
ENSG00000102265	-1.204296394	0.000355193
ENSG00000102287	1.357108001	0.021397241
ENSG00000102384	-1.248006369	0.015074227
ENSG00000102760	-1.431475893	0.002483385
ENSG00000102802	1.478702673	0.0000504
ENSG00000103018	-1.224081694	2.16E-08
ENSG00000103034	1.811423554	3.68E-12
ENSG00000103044	1.463972779	0.000236075
ENSG00000104147	-1.255867849	0.030683462
ENSG00000104213	-1.312873576	0.000241297
ENSG00000104312	1.264599946	0.000196266
ENSG00000104361	1.335561706	0.000233647
ENSG00000104369	-1.242719356	0.000518954
ENSG00000104447	1.301896861	0.001010065
ENSG00000104549	-1.392671552	3.13E-08
ENSG00000104689	-1.259213803	4.27E-07
ENSG00000104884	1.228309955	5.78E-06
ENSG00000105011	-1.249601603	0.004804551
ENSG00000105173	1.248915731	0.010503989
ENSG00000105270	1.363262512	0.003486001
ENSG00000105325	-1.212449514	4.76E-05
ENSG00000105497	1.200805569	0.002641586
ENSG00000105499	1.479820091	0.000113373
ENSG00000105639	1.466233289	0.001858946
ENSG00000105808	1.404850116	0.003735962
ENSG00000105825	-1.216792585	5.12E-06
ENSG00000105855	1.723539879	8.88E-09
ENSG00000105967	1.287682566	0.017782358
ENSG00000105991	1.312442957	0.001800549

Gene ID	FC 0NG - 24NG	adj p-value 0NG - 24NG
ENSG00000106392	1.255599059	0.015312308
ENSG00000106733	1.216934888	0.039894682
ENSG00000106772	1.236501174	0.000479198
ENSG00000107719	-1.498513987	2.35E-06
ENSG00000107984	1.310242572	0.001056137
ENSG00000108106	-1.2823718	6.26E-05
ENSG00000108984	1.476554695	0.000209087
ENSG00000109084	-1.288696589	3.98E-06
ENSG00000109107	-1.23906705	0.005934438
ENSG00000109458	-1.332192197	0.000580331
ENSG00000109501	-1.288263486	0.000203066
ENSG00000109738	1.295648249	0.010016637
ENSG00000109805	-1.342124539	0.003971773
ENSG00000110031	1.318538091	0.012595879
ENSG00000110318	1.301372774	0.022927022
ENSG00000110330	1.210405916	0.035778491
ENSG00000110799	1.206689434	0.001023964
ENSG00000110900	1.29599525	0.002325217
ENSG00000110921	-1.29239179	6.03E-05
ENSG00000111206	-1.281737659	1.13E-06
ENSG00000111339	1.478186086	5.76E-07
ENSG00000111341	-1.420063303	4.75E-08
ENSG00000111665	-1.406527686	0.000771428
ENSG00000111885	-1.246437331	0.000204269
ENSG00000112137	-1.20849044	0.004252391
ENSG00000112297	-1.444653584	4.73E-05
ENSG00000112541	1.308072915	0.013564542
ENSG00000112742	-1.452538547	0.005748777
ENSG00000112874	1.238715294	0.045520818
ENSG00000112972	-1.71981021	8.32E-07
ENSG00000112984	-1.392261356	8.01E-07
ENSG00000113161	-1.465223929	5.02E-06

Gene ID	FC 0NG - 24NG	adj p-value 0NG - 24NG
ENSG00000113368	-1.200866997	0.000210462
ENSG00000113555	-1.8989804	2.98E-08
ENSG00000114126	1.255151416	0.000199298
ENSG00000114346	-1.234147474	0.008112353
ENSG00000114529	1.248565941	0.027484142
ENSG00000114698	1.465535148	1.02E-06
ENSG00000114812	-1.650477421	0.00016858
ENSG00000115163	-1.433035525	3.54E-05
ENSG00000115290	1.279743291	0.002019856
ENSG00000115594	1.323180394	1.04E-05
ENSG00000115602	2.297987248	8.07E-08
ENSG00000115604	1.307876135	0.004093783
ENSG00000115963	1.284526563	0.001322895
ENSG00000116133	-1.251039397	2.37E-07
ENSG00000116991	-1.324376717	1.95E-05
ENSG00000117152	1.483878244	6.84E-08
ENSG00000117298	-1.226854267	1.75E-06
ENSG00000117318	1.406942828	7.24E-07
ENSG00000117399	-1.281269801	0.001228174
ENSG00000117643	1.21900236	0.021882682
ENSG00000117650	-1.503940121	9.36E-07
ENSG00000117724	-1.564862153	3.46E-08
ENSG00000118193	-1.473220454	7.22E-06
ENSG00000118308	1.248350698	0.042376177
ENSG00000118407	1.285354874	0.015713176
ENSG00000118515	1.26323298	8.36E-09
ENSG00000118564	1.210110408	0.01354032
ENSG00000118596	1.2711198	0.034511076
ENSG00000118985	1.214017102	0.000265854
ENSG00000119138	1.235954135	0.000295973
ENSG00000119280	-1.201227409	0.001069758
ENSG00000119397	-1.275136132	5.98E-05

Gene ID	FC 0NG - 24NG	adj p-value 0NG - 24NG
ENSG00000119403	-1.278022154	2.39E-06
ENSG00000119630	-1.771818867	5.21E-12
ENSG00000119686	1.227770268	0.019480262
ENSG00000119714	1.228449951	0.032849632
ENSG00000119718	-1.433105424	8.30E-12
ENSG00000119801	1.202893022	0.035804263
ENSG00000119900	-1.301351361	0.001415557
ENSG00000119922	1.206567156	0.021614186
ENSG00000119938	1.631805719	1.13E-06
ENSG00000119950	1.279865602	0.024690574
ENSG00000120278	-1.265527447	1.24E-05
ENSG00000120437	-1.598079087	4.17E-11
ENSG00000120457	-1.267534583	0.001831783
ENSG00000120693	1.317274531	0.005282387
ENSG00000121104	1.204470259	0.034397291
ENSG00000121152	-1.295358522	0.003790539
ENSG00000121653	1.241932704	0.036388694
ENSG00000121797	-1.239901241	0.005645558
ENSG00000121957	-1.402749154	4.14E-06
ENSG00000121966	-1.312612291	0.000318403
ENSG00000122420	1.220363034	0.047304234
ENSG00000122483	-1.337320037	0.001245431
ENSG00000122641	1.702046138	1.53E-08
ENSG00000122952	-1.207958604	0.000871316
ENSG00000122966	-1.539556936	5.77E-09
ENSG00000123080	-1.494481022	9.09E-08
ENSG00000123095	1.403817475	0.003862006
ENSG00000123342	1.239422928	0.040435938
ENSG00000123384	-1.402427536	0.000118156
ENSG00000123473	-1.217032303	0.008621334
ENSG00000123485	-1.544116241	6.59E-05
ENSG00000123689	1.480009361	0.001496241

Gene ID	FC 0NG - 24NG	adj p-value 0NG - 24NG
ENSG00000123700	1.430047598	0.024718329
ENSG00000124212	1.487083	0.003982024
ENSG00000124374	1.209388818	0.039366665
ENSG00000124610	1.383884078	9.28E-05
ENSG00000124875	1.662643835	1.31E-05
ENSG00000125148	1.205941915	0.000337558
ENSG00000125266	1.507804458	9.51E-07
ENSG00000125378	1.649311794	1.16E-07
ENSG00000125848	1.480413592	0.004557327
ENSG00000125864	1.255380116	0.01398521
ENSG00000125871	-1.205069606	0.00074441
ENSG00000125968	1.698178635	2.14E-05
ENSG00000126562	1.499972497	1.63E-07
ENSG00000126787	-1.246240757	0.048028274
ENSG00000126878	-1.211354513	0.005611648
ENSG00000126950	1.300170312	0.00948238
ENSG00000127418	1.245425395	0.000648672
ENSG00000127533	-2.184627246	6.78E-12
ENSG00000127603	-1.282038907	0.00658281
ENSG00000128567	-1.259554423	3.28E-06
ENSG00000128602	1.336745889	0.002263738
ENSG00000128606	1.329076993	1.07E-05
ENSG00000128641	-1.389242163	3.95E-06
ENSG00000128833	-1.371613721	0.000337809
ENSG00000128917	-1.801525193	3.16E-07
ENSG00000128944	-1.281414473	6.43E-05
ENSG00000129116	1.211072486	0.019986816
ENSG00000129195	-1.344245802	0.000216282
ENSG00000129810	-1.261445068	0.01122678
ENSG00000129946	1.272343837	0.024195915
ENSG00000130024	-1.286231378	0.000167644
ENSG00000130052	-1.220178199	0.001681283

Gene ID	FC 0NG - 24NG	adj p-value 0NG - 24NG
ENSG00000130158	-1.202308432	2.16E-05
ENSG00000130164	-1.305381924	6.02E-05
ENSG00000130300	-1.403280283	0.018714746
ENSG00000130589	1.211152779	0.029533593
ENSG00000131016	-1.361974052	1.74E-09
ENSG00000131069	-1.280386484	0.001776964
ENSG00000131370	-1.365388563	1.69E-09
ENSG00000131386	1.452336661	0.002659487
ENSG00000131459	1.295626143	0.002433454
ENSG00000131652	-1.272527511	0.000724645
ENSG00000131724	1.240125547	0.00100097
ENSG00000131747	-1.465917479	2.04E-05
ENSG00000131773	-1.292310067	0.005211745
ENSG00000132182	-1.321560757	0.004929879
ENSG00000132196	-1.3190577	1.64E-05
ENSG00000132530	1.407125846	0.010618439
ENSG00000132541	1.238295365	0.028617151
ENSG00000132846	1.229873476	0.000553506
ENSG00000132879	1.214648237	0.029425663
ENSG00000133101	1.238191332	0.002666323
ENSG00000133106	1.370483251	0.024845245
ENSG00000133863	1.460077909	0.003023992
ENSG00000133935	-1.205605748	0.000423376
ENSG00000134057	-1.331384276	6.33E-05
ENSG00000134107	-1.309092513	7.57E-06
ENSG00000134109	-1.233829886	1.79E-10
ENSG00000134222	-1.488987975	4.23E-05
ENSG00000134242	1.509927115	0.000484239
ENSG00000134317	1.200383263	0.034162287
ENSG00000134324	-1.316122446	3.64E-09
ENSG00000134352	1.272690031	0.00402887
ENSG00000134508	1.232966225	0.010517877

Gene ID	FC 0NG - 24NG	adj p-value 0NG - 24NG
ENSG00000134690	-1.511002918	2.96E-06
ENSG00000134780	1.256398368	0.003461817
ENSG00000134824	-1.229059681	3.83E-05
ENSG00000134986	1.218937498	0.001681076
ENSG00000135451	-1.44894211	0.000456842
ENSG00000135476	-1.36668981	0.004360193
ENSG00000135547	1.380468683	0.032335616
ENSG00000135643	1.319678696	0.004601937
ENSG00000135905	-1.21175762	0.000125112
ENSG00000135929	1.315667241	0.000507493
ENSG00000135966	-1.231090821	0.000127483
ENSG00000136108	-1.312216014	0.003609164
ENSG00000136111	-1.206581239	8.98E-06
ENSG00000136122	-1.312183598	0.001125888
ENSG00000136153	-1.601643663	4.60E-13
ENSG00000136205	-1.605543858	4.66E-12
ENSG00000136237	-1.216085543	0.006811393
ENSG00000136305	1.267411259	0.029359746
ENSG00000136383	1.271138607	0.000218132
ENSG00000136490	1.357158626	1.03E-06
ENSG00000136630	-1.364793042	0.024477064
ENSG00000136861	-1.22262268	0.000106821
ENSG00000136869	1.220844887	0.000825568
ENSG00000137135	-1.280901429	0.006544598
ENSG00000137203	1.266616283	0.004584228
ENSG00000137507	1.289318942	0.000488167
ENSG00000137522	1.25854566	0.000074003
ENSG00000137727	1.421816623	3.74E-05
ENSG00000137804	-1.570593826	2.14E-08
ENSG00000137807	-1.420000441	2.63E-06
ENSG00000137812	-1.3501724	0.006324665
ENSG00000137825	-1.20504142	0.045096995

Gene ID	FC 0NG - 24NG	adj p-value 0NG - 24NG
ENSG00000137834	1.630755754	0.002607892
ENSG00000137841	1.203739206	0.032120349
ENSG00000137872	1.284516675	0.017675014
ENSG00000137880	-1.412812559	0.003617528
ENSG00000137968	1.404125751	0.007599873
ENSG00000138160	-1.395273765	0.000760612
ENSG00000138180	-1.342398684	0.000346481
ENSG00000138347	-1.411209475	0.003161733
ENSG00000138356	1.480849963	1.59E-06
ENSG00000138434	-1.217173106	4.71E-06
ENSG00000138449	1.604586041	1.83E-09
ENSG00000138640	1.248451045	0.016972416
ENSG00000138675	1.300284691	0.010282205
ENSG00000138685	1.324587963	0.000468837
ENSG00000138778	-1.32856121	0.027070471
ENSG00000139278	1.307609959	1.17E-05
ENSG00000139354	-1.289313073	0.001701511
ENSG00000139428	-1.287637704	1.11E-07
ENSG00000139734	-1.239359165	0.004089925
ENSG00000139880	1.202003658	0.012304788
ENSG00000140092	-1.369339516	1.48E-06
ENSG00000140105	1.216577785	0.002847173
ENSG00000140416	1.261575141	1.13E-08
ENSG00000140451	-1.626352881	2.10E-05
ENSG00000140534	-1.316841959	0.003382749
ENSG00000140848	-1.208598915	6.99E-06
ENSG00000141448	1.203850796	0.003907161
ENSG00000141449	1.270049196	0.049202288
ENSG00000141519	1.222533087	0.024452796
ENSG00000142731	-1.261365593	0.030882478
ENSG00000142945	-1.427099157	8.09E-05
ENSG00000143153	1.229299256	0.000432778

Gene ID	FC 0NG - 24NG	adj p-value 0NG - 24NG
ENSG00000143228	-1.272490794	0.01966997
ENSG00000143344	-1.414366548	2.18E-09
ENSG00000143434	1.33502821	5.16E-05
ENSG00000143469	1.41503999	0.003807155
ENSG00000143507	-1.243410957	0.007988227
ENSG00000143772	-1.427142038	1.06E-07
ENSG00000143816	-1.375165474	0.025218524
ENSG00000144554	-1.254378725	0.002826959
ENSG00000144645	-1.310840598	4.30E-09
ENSG00000144749	-1.374285195	1.33E-06
ENSG00000144843	1.222262495	0.041775845
ENSG00000145386	-1.418705553	1.40E-06
ENSG00000145431	1.275844403	0.031332752
ENSG00000145911	-1.471945463	8.92E-06
ENSG00000145916	-1.223114135	0.003333956
ENSG00000146859	1.334083509	0.003381355
ENSG00000147027	-1.209652878	0.026259594
ENSG00000147113	1.256908713	0.009502047
ENSG00000147133	-1.258902901	1.59E-09
ENSG00000147155	-1.222677011	1.06E-06
ENSG00000147202	-1.233971525	0.03540964
ENSG00000147383	-1.250614942	2.27E-06
ENSG00000147852	-1.455369781	0.000211746
ENSG00000147872	1.337985878	1.96E-06
ENSG00000148143	1.213607083	0.004091765
ENSG00000148677	1.445590946	4.33E-09
ENSG00000148700	-1.356064946	7.51E-07
ENSG00000148773	-1.564629659	1.32E-07
ENSG00000148842	1.353686173	0.002800197
ENSG00000148926	-1.53625205	1.31E-05
ENSG00000149212	1.428071699	4.50E-07
ENSG00000149269	1.413112753	0.000797049

Gene ID	FC 0NG - 24NG	adj p-value 0NG - 24NG
ENSG00000149380	-1.218230887	0.000291977
ENSG00000149503	-1.414840969	3.55E-06
ENSG00000149809	-1.45783085	0.001108565
ENSG00000149929	-1.240971678	0.009577052
ENSG00000150347	1.517934227	0.000441725
ENSG00000150551	1.374941153	1.50E-05
ENSG00000150630	1.28279946	0.000140366
ENSG00000150764	1.256092704	0.002268562
ENSG00000151117	1.285436579	0.039171423
ENSG00000151150	1.233518271	0.00076333
ENSG00000151458	1.211837204	0.00895961
ENSG00000151690	-1.247182268	0.03271913
ENSG00000152127	-1.223568789	7.23E-08
ENSG00000152137	1.212741124	0.003818701
ENSG00000152229	1.260520205	0.005563953
ENSG00000152409	1.267517105	0.038878761
ENSG00000152422	-1.269744854	0.026206485
ENSG00000152778	1.21708622	0.012554558
ENSG00000152953	-1.265910292	0.000452883
ENSG00000154016	-1.31961546	0.000112585
ENSG00000154127	1.212902338	4.96E-05
ENSG00000154258	1.263226489	0.013521852
ENSG00000154310	1.297276901	5.13E-09
ENSG00000154639	-1.298706364	0.037880245
ENSG00000154678	1.233541749	0.012179833
ENSG00000154734	1.4749697	0.003779834
ENSG00000155846	1.247891333	0.026458604
ENSG00000156011	1.212322485	0.005655051
ENSG00000156049	1.285710056	0.028228537
ENSG00000156265	-1.30896524	0.001977973
ENSG00000156711	1.557196266	9.05E-05
ENSG00000156970	-1.421485975	1.61E-05
ENSG00000157168	1.456008596	1.17E-09
ENSG00000157214	1.403295148	0.000144391
ENSG00000157227	-1.211839075	0.00011335
ENSG00000157404	-1.850396444	6.23E-06
ENSG00000157456	-1.380979541	2.27E-06
ENSG00000157510	-1.265268525	1.04E-07
ENSG00000157557	-1.278715871	2.96E-09
ENSG00000157613	-1.929724663	2.48E-06
ENSG00000158023	1.354161468	0.000406818
ENSG00000158195	-1.261802448	2.16E-06
ENSG00000158286	1.502440422	0.007383145
ENSG00000158402	-1.465180053	3.87E-06
ENSG00000158458	1.231199648	0.024695424
ENSG00000158683	1.30846713	0.014991842
ENSG00000158716	1.290981839	0.000889045
ENSG00000158856	-1.297111696	3.11E-05
ENSG00000158859	-1.245387013	0.002057849
ENSG00000160013	-1.404423425	0.002813602
ENSG00000160117	1.34354666	0.002075
ENSG00000160223	-1.226849237	0.005724737
ENSG00000160285	-1.315133751	1.47E-05
ENSG00000160299	-1.202279331	0.000418191
ENSG00000160447	-1.229957615	6.80E-05
ENSG00000160752	-1.258241341	3.95E-07
ENSG00000161011	1.220313766	5.49E-05
ENSG00000161243	1.304167373	0.003897028
ENSG00000161558	-1.253388728	0.013521486
ENSG00000161682	1.251406396	0.014712965
ENSG00000161692	-1.232181697	0.026872774
ENSG00000161791	-1.257854761	3.10E-09
ENSG00000161800	-1.45473574	8.01E-07
ENSG00000161888	-1.244808316	0.002507791
ENSG00000161940	-1.234371237	4.19E-06
ENSG00000162063	-1.421619807	2.52E-05
ENSG00000162437	1.239941284	0.002286099
ENSG00000162496	1.263468704	0.013170238
ENSG00000162545	1.270160147	0.001727659
ENSG00000162576	1.260664124	0.000515277
ENSG00000162595	1.298961917	0.035330242
ENSG00000162614	1.362232967	2.73E-05
ENSG00000162645	1.227077246	0.049805111
ENSG00000162746	1.252748127	0.025117601
ENSG00000163171	-1.44696002	2.63E-09
ENSG00000163633	1.21858862	0.02035855
ENSG00000163637	1.519438857	1.78E-07
ENSG00000163659	-1.212663445	3.33E-06
ENSG00000163661	1.356475074	4.87E-05
ENSG00000163689	1.250203569	0.034012086
ENSG00000163739	1.413635801	0.002453586
ENSG00000163762	-1.289901856	0.01173049
ENSG00000163884	1.264663827	0.005425785
ENSG00000164023	1.213685835	0.010719665
ENSG00000164087	-1.344179894	0.000476653
ENSG00000164104	-1.395389652	1.01E-05
ENSG00000164109	-1.228297891	0.040758805
ENSG00000164292	1.250483906	0.000136425
ENSG00000164647	1.264079525	0.010072399
ENSG00000164663	1.305824687	0.001690602
ENSG00000164687	1.213391003	0.015100918
ENSG00000164754	-1.224628941	1.72E-07
ENSG00000165029	2.323754172	2.89E-10
ENSG00000165092	2.006609934	6.24E-11
ENSG00000165304	-1.236800844	0.000455225
ENSG00000165821	1.367516649	0.000918579

Gene ID	FC 0NG - 24NG	adj p-value 0NG - 24NG
ENSG00000165886	-1.215242413	0.000192573
ENSG00000165895	1.368232368	0.000608136
ENSG00000165912	1.29756828	0.000144186
ENSG00000166250	1.393920269	0.000141138
ENSG00000166265	1.227462524	0.006326057
ENSG00000166510	1.373865414	0.012786894
ENSG00000166670	-1.699169096	1.41E-08
ENSG00000166803	-1.234396583	0.002507229
ENSG00000166851	-1.463795931	1.28E-05
ENSG00000166897	1.459738137	6.43E-05
ENSG00000166900	1.212818639	9.38E-06
ENSG00000166938	1.206814616	0.000394184
ENSG00000166963	1.319970393	0.009258726
ENSG00000166979	1.201239881	0.001483404
ENSG00000167107	1.327146381	0.000264157
ENSG00000167191	-1.42521393	0.000133495
ENSG00000167508	-1.583823617	3.04E-09
ENSG00000167772	1.579941762	2.95E-05
ENSG00000167799	-1.268448055	0.000870716
ENSG00000167874	1.417970246	0.000175494
ENSG00000168016	1.29088486	0.000251028
ENSG00000168300	1.257960856	0.001175264
ENSG00000168386	1.375111544	0.001291162
ENSG00000168476	-1.299702052	3.49E-06
ENSG00000168646	1.274582752	0.037273937
ENSG00000168672	1.211384564	0.016388272
ENSG00000168685	1.46629016	0.018299367
ENSG00000168961	1.464929597	0.000179077
ENSG00000169126	1.271829609	0.010492554
ENSG00000169282	1.431557272	2.35E-06
ENSG00000169604	-1.231377903	0.000684168
ENSG00000169607	-1.388783917	0.000561715
ENSG00000169679	-1.442760223	0.000002017
ENSG00000169710	-1.287119833	2.46E-05
ENSG00000169715	1.3471595	3.29E-05
ENSG00000169760	1.344632782	0.000169025
ENSG00000169851	1.542077879	8.97E-06
ENSG00000169908	-1.240370647	7.48E-05
ENSG00000169914	1.21068975	0.015508867
ENSG00000169933	1.205241256	0.04375165
ENSG00000170175	-1.299998237	0.00064579
ENSG00000170271	1.3860559	0.002357215
ENSG00000170312	-1.489313067	0.000126772
ENSG00000170323	1.590602993	0.000631325
ENSG00000170365	-1.342711578	4.82E-06
ENSG00000170385	1.330737847	0.010795364
ENSG00000170456	1.353830947	9.44E-06
ENSG00000170522	-1.284592618	1.21E-05
ENSG00000170540	-1.291866501	0.022299986
ENSG00000170743	1.356363627	0.012752221
ENSG00000170775	1.203212776	0.000986215
ENSG00000170915	-1.207195845	0.010629392
ENSG00000171017	1.246450988	0.027452619
ENSG00000171241	-1.233470865	0.021451015
ENSG00000171345	1.273971235	0.004702212
ENSG00000171388	-1.564673539	1.72E-06
ENSG00000171621	-1.305780145	0.040374224
ENSG00000171843	1.233177211	0.033367155
ENSG00000172183	-1.255174792	0.015374191
ENSG00000172216	1.22768861	0.003778973
ENSG00000172260	1.233843389	0.03759847
ENSG00000172819	1.217675181	0.029031167
ENSG00000172893	-1.355450753	2.58E-08
ENSG00000173068	1.238096477	5.13E-05
ENSG00000173207	-1.434861796	2.94E-08
ENSG00000173597	1.386150406	0.003894518
ENSG00000174370	-1.317921026	0.010554505
ENSG00000174442	-1.205708365	0.0147677
ENSG00000174516	1.359740155	0.001234543
ENSG00000174738	1.263581164	0.001437278
ENSG00000174791	1.322844159	0.002254035
ENSG00000175063	-1.365131204	0.000395258
ENSG00000175216	-1.250763494	3.38E-07
ENSG00000175264	-1.598215225	9.23E-11
ENSG00000175274	-1.325311932	3.03E-06
ENSG00000175352	1.220448906	0.00717559
ENSG00000175471	1.458311977	7.18E-07
ENSG00000175567	-1.217850079	0.039281051
ENSG00000175745	1.315295748	2.40E-07
ENSG00000175764	1.202588908	0.018736064
ENSG00000175832	-1.27425207	5.39E-05
ENSG00000175899	1.492599152	0.002584224
ENSG00000176046	1.31876197	0.026344746
ENSG00000176244	-1.264127318	0.015300487
ENSG00000176658	-1.259771936	2.00E-06
ENSG00000176692	1.264475919	0.001925427
ENSG00000176697	1.398535511	0.032667075
ENSG00000176907	1.347273271	0.025598838
ENSG00000177374	-1.326907836	0.005510863
ENSG00000177406	1.250447229	0.006593989
ENSG00000178031	1.222635141	3.03E-05
ENSG00000178184	1.310784514	0.000527605
ENSG00000178498	1.202776718	0.04666917
ENSG00000178695	1.250604469	0.00015408
ENSG00000178752	-1.263076947	0.016156323
ENSG00000178882	1.544325287	7.10E-05

Gene ID	FC 0NG - 24NG	adj p-value 0NG - 24NG
ENSG00000178922	1.2323137	0.00076888
ENSG00000178999	-1.319314014	0.003053079
ENSG00000179094	1.270324686	0.000361107
ENSG00000179750	-1.273545971	0.007447194
ENSG00000179922	1.216010349	0.027182755
ENSG00000180346	1.261829345	0.034449277
ENSG00000180354	1.278091111	0.015875038
ENSG00000181444	1.265787168	0.02066315
ENSG00000181634	1.46323113	0.02142794
ENSG00000182013	1.252532486	0.006327924
ENSG00000182022	-1.222721298	1.45E-06
ENSG00000182158	-1.248945769	9.33E-16
ENSG00000182481	-1.28711012	1.67E-08
ENSG00000182568	1.373757995	0.004374872
ENSG00000182575	-1.257131014	0.040533277
ENSG00000182809	-1.255246074	1.21E-06
ENSG00000182916	1.351653107	0.001206831
ENSG00000183763	-1.290496888	0.025608731
ENSG00000183856	-1.466125642	6.98E-07
ENSG00000183873	-1.403592473	3.16E-06
ENSG00000184254	-1.320772041	2.63E-08
ENSG00000184292	1.342673097	0.033534761
ENSG00000184371	1.289065572	0.001579947
ENSG00000184661	-1.439471377	6.54E-05
ENSG00000184719	1.267529141	0.008226612
ENSG00000184792	-1.2000062	0.02207784
ENSG00000184867	1.201765667	0.002799101
ENSG00000184898	1.264893761	0.007896557
ENSG00000185022	-1.24481492	0.000126428
ENSG00000185070	1.300514512	0.000292766
ENSG00000185215	1.402871246	3.79E-06
ENSG00000185262	-1.347441628	1.01E-06

Gene ID	FC 0NG - 24NG	adj p-value 0NG - 24NG
ENSG00000185291	1.292025201	0.025751003
ENSG00000185338	-1.297766987	0.032280003
ENSG00000185361	-1.212092383	0.004012191
ENSG00000185477	1.237071599	0.00479239
ENSG00000185551	1.210175649	1.37E-05
ENSG00000185813	-1.274697203	4.20E-06
ENSG00000185972	1.292896903	0.049668398
ENSG00000186185	-1.340715143	0.002487655
ENSG00000186193	-1.374433718	2.80E-06
ENSG00000186281	-1.35617677	0.000418196
ENSG00000186480	-1.266289432	0.01856266
ENSG00000186603	1.327622839	0.00109176
ENSG00000186638	-1.329132322	0.010028357
ENSG00000187266	-1.269911248	0.000739589
ENSG00000187479	1.393615017	0.005542337
ENSG00000187678	-1.32231662	1.10E-05
ENSG00000187951	-1.325729592	0.02190134
ENSG00000188277	-1.717437946	0.003873835
ENSG00000188343	1.272661292	1.01E-05
ENSG00000188486	-1.233559019	0.00242956
ENSG00000188921	1.315422398	0.022504417
ENSG00000189056	-1.227020033	0.032759033
ENSG00000189057	1.356561851	0.020428628
ENSG00000189152	-1.301380829	0.049581769
ENSG00000189221	1.293710683	0.000556608
ENSG00000196155	1.400181757	1.68E-07
ENSG00000196421	-1.244366772	0.012885544
ENSG00000196468	-1.27361927	0.026532314
ENSG00000196605	1.227469523	0.030366457
ENSG00000196611	-1.820180584	4.57E-08
ENSG00000196878	1.475458804	0.001065667
ENSG00000197119	1.242922569	0.004972801

Gene ID	FC 0NG - 24NG	adj p-value 0NG - 24NG
ENSG00000197555	1.302042749	3.67E-06
ENSG00000197632	1.393671758	0.004968556
ENSG00000198055	-1.202922121	0.000701799
ENSG00000198093	-1.215142199	0.033717437
ENSG00000198355	-1.23113835	0.000786083
ENSG00000198435	-1.223173109	0.044611418
ENSG00000198680	1.291918633	0.004883161
ENSG00000198721	1.297155127	2.58E-09
ENSG00000198844	-1.248123808	0.000936593
ENSG00000198846	1.729726517	2.44E-07
ENSG00000198873	-1.349374207	1.56E-08
ENSG00000198901	-1.419433762	2.24E-07
ENSG00000203883	-1.547147556	0.000097881
ENSG00000204103	1.500576365	2.49E-05
ENSG00000204381	1.326984164	0.005277173
ENSG00000204403	1.218875568	0.007360617
ENSG00000204442	1.363674557	0.000234756
ENSG00000205133	-1.286232328	0.012244205
ENSG00000206538	1.267231632	0.00699034
ENSG00000213347	-1.487683752	3.65E-06
ENSG00000213462	1.302851308	0.01191127
ENSG00000214357	-1.703293299	0.000054892
ENSG00000215481	1.200909587	0.00909512
ENSG00000216775	-1.344084616	0.001103676
ENSG00000221866	1.518381446	0.000196161
ENSG00000222365	1.244438467	0.011178659
ENSG00000225830	1.260478818	8.41E-05
ENSG00000226415	1.222302622	0.019013271
ENSG00000226823	1.219243101	0.045171153
ENSG00000227517	1.400538117	0.001518224
ENSG00000227825	1.416450636	0.003697797
ENSG00000228672	1.227005256	0.020332764

Gene ID	FC 0NG - 24NG	adj p-value 0NG - 24NG
ENSG00000230043	1.229501585	0.023265067
ENSG00000232034	-1.251587935	0.012911377
ENSG00000232931	-1.399992741	0.037522283
ENSG00000233117	-1.521122319	0.00011544
ENSG00000234964	1.245260933	0.027328269
ENSG00000235109	1.251641258	0.0158206
ENSG00000235217	1.422240895	0.000182634
ENSG00000235750	-1.336974116	2.63E-06
ENSG00000235823	-1.293497779	0.009058434
ENSG00000239282	1.205354475	0.018495123
ENSG00000240694	-1.644184687	8.70E-14
ENSG00000241529	1.293404133	0.025496318
ENSG00000242265	-1.431428179	2.90E-12
ENSG00000244300	1.235164227	0.002992594
ENSG00000247746	1.231513295	0.03248546
ENSG00000248187	1.245058701	0.013132694
ENSG00000249867	1.368043457	0.015805194
ENSG00000250303	1.279661676	0.002775175
ENSG00000250903	-1.251492986	0.02184865
ENSG00000253522	1.215838464	0.012852566
ENSG00000255248	1.384389524	0.000933565
ENSG00000256235	1.265813857	0.006039704
ENSG00000256940	-1.210419377	0.007896557
ENSG00000257702	1.275419757	0.023106574
ENSG00000258376	1.281453329	0.002714897
ENSG00000259943	1.233700437	0.034266494
ENSG00000261253	-1.409526191	8.60E-05
ENSG00000261373	1.308515731	3.29E-05
ENSG00000261801	1.258050209	0.047356628
ENSG00000262580	1.230091864	0.040457141
ENSG00000267583	-1.262176023	7.20E-05
ENSG00000267710	1.207001429	0.006986232
ENSG00000272941	1.241241131	0.022882157
ENSG00000274016	1.392916959	0.000833722
ENSG00000274180	1.371369272	0.001259425
ENSG00000275713	-1.290775605	0.029208559
ENSG00000276644	-1.302847179	0.015666307
ENSG00000277594	1.20915652	0.030322182
ENSG00000277775	-1.290964247	0.015295013
ENSG00000279041	-1.237315253	0.011768906
ENSG00000279948	-1.256259966	0.023409389

Supplementary Table 2a. Differentially expressed genes between 0.5hr and 1hr normal glucose samples

Ensembl gene IDs, Fold change (0.5NG-1NG) and FDR adjusted p-values of DEGs

Gene ID	FC 0.5NG - 1NG	adj p-value 0.5NG - 1NG
ENSG00000004660	-1.313932	0.00289409
ENSG00000005513	-3.1777821	1.07E-14
ENSG00000006062	-1.3609973	0.00059399
ENSG00000006327	-1.236008	2.15E-05
ENSG00000006459	-1.3577904	0.00020984
ENSG00000015568	-1.2826593	0.02908615
ENSG00000019144	-1.265172	3.82E-06
ENSG00000019549	-1.73174	1.61E-07
ENSG00000023445	-1.8866722	0.00014667
ENSG00000049130	-1.4621146	0.02386575
ENSG00000049883	1.22500556	0.00580991
ENSG00000054598	-1.671323	2.23E-09
ENSG00000054967	-1.5380597	1.85E-06
ENSG00000056558	-1.617995	0.0148115
ENSG00000057657	1.34213626	0.00793936
ENSG00000059728	-1.2719038	0.00076179
ENSG00000062716	1.30190369	0.01192892
ENSG00000067082	1.40136595	3.12E-08
ENSG00000069399	-1.5101852	3.57E-07
ENSG00000070404	-1.2680011	0.00119479
ENSG00000073756	-1.9968937	0.00254461
ENSG00000075426	-1.592493	3.84E-12
ENSG00000078401	1.20487353	0.00309457
ENSG00000079337	-1.2833753	0.00579601
ENSG00000083812	-1.352249	4.33E-06
ENSG00000084093	-1.3790345	1.74E-06
ENSG00000084112	-1.2085209	9.16E-05
ENSG00000084710	1.26623662	0.04236749
ENSG00000087494	-1.6139248	5.52E-05
ENSG00000088826	-1.3946411	2.87E-07
ENSG00000095739	-1.2524127	0.01683185
ENSG00000095752	-2.2795334	8.51E-10

Gene ID	FC 0.5NG - 1NG	adj p-value 0.5NG - 1NG
ENSG00000096654	1.3540942	0.00188566
ENSG00000099860	-1.5547715	6.26E-06
ENSG00000100276	-1.4744514	7.17E-06
ENSG00000100292	-1.2582369	0.00170566
ENSG00000100311	-1.4823629	0.00010629
ENSG00000100625	-1.242585	0.04173657
ENSG00000100906	-1.5899089	9.59E-10
ENSG00000101384	-1.4260921	8.57E-06
ENSG00000101665	-6.0992372	2.08E-17
ENSG00000102034	-1.2438995	2.20E-05
ENSG00000102890	-1.386396	0.01590878
ENSG00000103241	-1.3469981	0.0125113
ENSG00000103257	-1.3910677	1.75E-08
ENSG00000103343	1.21636505	0.00794786
ENSG00000104081	-1.512306	0.02679656
ENSG00000104419	-1.2686318	2.79E-06
ENSG00000104903	-1.2363787	0.00868404
ENSG00000105722	-1.5884035	1.00E-10
ENSG00000106003	-2.2568867	1.85E-09
ENSG00000106089	-1.5470535	1.0792E-05
ENSG00000106236	-1.2979709	0.0302307
ENSG00000106799	-1.2284878	0.01294177
ENSG00000107104	-1.2349468	1.67E-07
ENSG00000107562	-1.4444027	1.85E-05
ENSG00000107731	-1.3639312	0.00609345
ENSG00000107807	-1.5123699	0.00010144
ENSG00000107821	-1.5607661	0.0007467
ENSG00000107968	-1.3589764	0.03109202
ENSG00000108312	-1.2244218	1.26E-05
ENSG00000108342	1.39786913	0.00668129
ENSG00000109118	1.27090474	1.07E-06
ENSG00000110330	-1.2444467	0.01202104

Gene ID	FC 0.5NG - 1NG	adj p-value 0.5NG - 1NG
ENSG00000110888	-1.2108389	0.02306993
ENSG00000111276	1.31708649	3.24E-05
ENSG00000111859	-1.7467121	6.29E-08
ENSG00000112406	-1.2237363	0.01164827
ENSG00000112658	-1.2345369	1.49E-08
ENSG00000112715	-2.3342004	3.13E-12
ENSG00000113070	-1.3713093	0.00037341
ENSG00000113369	1.27264825	0.00285753
ENSG00000113742	-1.244298	0.00036227
ENSG00000114019	-1.4114333	2.01E-08
ENSG00000114315	-1.6686611	0.00145234
ENSG00000114529	-1.5265699	6.54E-06
ENSG00000114739	1.22781829	0.03087023
ENSG00000115548	-1.3340841	1.13E-07
ENSG00000115738	-2.4748583	2.62E-08
ENSG00000115844	-2.7077339	1.22E-14
ENSG00000116017	-1.2979695	0.01251581
ENSG00000116273	-1.2600606	6.81E-05
ENSG00000116285	-1.6348292	9.17E-10
ENSG00000116604	-1.2627189	0.00013746
ENSG00000116717	1.43560164	4.01E-09
ENSG00000116731	-1.2960028	2.19E-08
ENSG00000117036	-1.2874449	1.95E-08
ENSG00000117143	-1.2259579	2.63E-05
ENSG00000117318	-1.5541696	1.94E-09
ENSG00000117479	-1.2274398	0.03021995
ENSG00000118263	-1.3543183	2.96E-07
ENSG00000118503	-1.4906073	0.00028795
ENSG00000118515	-1.3018363	3.95E-10
ENSG00000119508	-3.4764867	1.04E-11
ENSG00000119669	-1.6062957	1.68E-09
ENSG00000120616	-1.2001422	0.00104855

Gene ID	FC 0.5NG - 1NG	adj p-value 0.5NG - 1NG
ENSG00000120784	1.23281846	0.03111878
ENSG00000121671	-1.200209	0.02209736
ENSG00000121966	1.33526017	0.00012809
ENSG00000122386	1.22046434	0.02248306
ENSG00000122644	1.26370368	0.00592244
ENSG00000122863	-1.8288954	1.03E-11
ENSG00000123358	-2.28169	5.94E-07
ENSG00000124019	1.38656816	4.61E-06
ENSG00000124216	-1.98272	2.31E-11
ENSG00000124762	-1.2213081	7.92E-09
ENSG00000125266	-1.3055875	0.00124013
ENSG00000125378	1.33485284	0.00160036
ENSG00000125740	-2.9632702	7.25E-06
ENSG00000125812	-1.2181018	0.00045354
ENSG00000125898	-1.2530097	0.01076062
ENSG00000125945	1.26797448	0.00014752
ENSG00000125968	-1.7478655	8.21E-06
ENSG00000126368	1.24098217	0.03848857
ENSG00000126391	-1.2255408	4.39E-06
ENSG00000126603	-1.2402351	0.00728981
ENSG00000126778	-1.4025384	0.00096011
ENSG00000127080	-1.5141658	5.26E-11
ENSG00000128016	1.91092044	7.45E-08
ENSG00000128283	-1.2444132	5.74E-06
ENSG00000128342	-1.6854626	7.47E-07
ENSG00000128594	-2.8779669	2.15E-12
ENSG00000129465	-1.2723695	0.02581545
ENSG00000129514	-1.3423495	5.67E-05
ENSG00000130164	-1.2091207	0.00550379
ENSG00000130222	-1.3837705	0.00103689
ENSG00000130479	-1.2511224	1.38E-06
ENSG00000130513	-1.2263942	0.00011281

Gene ID	FC 0.5NG - 1NG	adj p-value 0.5NG - 1NG
ENSG00000130810	-1.2432297	0.02446797
ENSG00000131016	-1.2434337	4.39E-06
ENSG00000131634	-1.2833434	0.00014798
ENSG00000131759	-1.4590172	8.35E-06
ENSG00000132326	-1.3794932	0.00084007
ENSG00000132481	-1.2910298	0.00137518
ENSG00000132510	-2.2197284	1.48E-16
ENSG00000132570	1.22031369	0.04289303
ENSG00000133561	1.23157103	2.43E-05
ENSG00000133574	1.20002677	0.04565676
ENSG00000134107	-1.9714846	1.72E-16
ENSG00000135547	-2.3826869	5.45E-09
ENSG00000135625	-1.7598926	2.74E-10
ENSG00000135723	-1.2156886	1.71E-05
ENSG00000136244	1.41238368	0.00669777
ENSG00000136490	-1.2395029	0.0004577
ENSG00000136603	-1.6880589	4.46E-09
ENSG00000136826	-1.5938022	1.84E-06
ENSG00000137331	1.4068601	4.08E-06
ENSG00000137507	-1.2060074	0.01485526
ENSG00000137834	-2.7815445	3.50E-09
ENSG00000138166	-1.4578924	1.77E-06
ENSG00000138623	-2.0510231	3.14E-07
ENSG00000138675	-1.3731591	0.00131253
ENSG00000138835	-1.3247602	0.00044606
ENSG00000139263	1.3071007	1.74E-06
ENSG00000139289	1.22450849	0.00566981
ENSG00000139517	-1.2373037	0.00034497
ENSG00000140534	1.22450317	0.04872544
ENSG00000141526	-1.2940728	1.82E-07
ENSG00000141582	-1.3897302	0.00011166
ENSG00000141682	1.2647315	0.00226756

Gene ID	FC 0.5NG - 1NG	adj p-value 0.5NG - 1NG
ENSG00000142178	-1.4493548	0.00037451
ENSG00000142279	-1.414406	2.45E-06
ENSG00000142408	-1.2638817	0.00606046
ENSG00000142627	-1.288932	1.09E-06
ENSG00000143067	-1.6654391	1.69E-08
ENSG00000143322	-1.2673298	1.67E-07
ENSG00000143363	1.26829369	0.00447471
ENSG00000143367	-1.4517361	7.16E-06
ENSG00000143479	-1.2903456	0.00138509
ENSG00000143878	-1.2681308	0.00700574
ENSG00000144136	-1.2683762	3.92E-07
ENSG00000144655	-1.9670914	8.88E-16
ENSG00000144802	1.39169673	0.03933659
ENSG00000145632	2.41576809	2.82E-18
ENSG00000146232	-1.8099421	7.03E-14
ENSG00000148339	-1.3404181	0.00012781
ENSG00000148677	-1.305757	5.37E-06
ENSG00000148926	1.77013813	3.41E-08
ENSG00000149289	-1.2625859	0.00065881
ENSG00000149591	-1.2086125	0.02734273
ENSG00000151014	-1.2998546	6.06E-08
ENSG00000151748	-1.4239498	4.61E-08
ENSG00000152782	1.25325462	0.03187895
ENSG00000153879	-1.2238269	0.00167504
ENSG00000153885	-1.360165	9.76E-08
ENSG00000154553	-1.2065868	0.00659749
ENSG00000155090	-1.623832	3.99E-07
ENSG00000155760	-1.2679703	0.00017447
ENSG00000156030	-1.3182245	5.22E-06
ENSG00000156273	-1.6390002	1.25E-10
ENSG00000156500	1.27417669	0.02064984
ENSG00000156983	-1.2783603	2.59E-07

Gene ID	FC 0.5NG - 1NG	adj p-value 0.5NG - 1NG
ENSG00000157240	-1.2907405	0.00972846
ENSG00000158792	-1.2148692	0.00917664
ENSG00000158859	-1.2747588	0.00056769
ENSG00000161835	-1.6259975	5.58E-10
ENSG00000161940	-1.3438185	2.13E-09
ENSG00000162407	-1.2888285	0.01865799
ENSG00000162413	-1.4499415	9.21E-11
ENSG00000162616	1.25661272	0.02339868
ENSG00000162702	-1.2389001	0.00031771
ENSG00000163132	-1.2874577	0.00397657
ENSG00000163376	-1.353815	0.01523734
ENSG00000163545	-2.1427975	1.32E-08
ENSG00000163659	-1.2670579	4.06E-08
ENSG00000163874	-1.5598273	2.67E-05
ENSG00000164056	2.08805106	1.83E-08
ENSG00000164086	-1.2503952	4.15E-06
ENSG00000164442	1.25444458	0.02798932
ENSG00000164532	-1.2885403	0.0020308
ENSG00000164683	-3.6208366	4.25E-15
ENSG00000164743	1.35029111	0.00159329
ENSG00000164855	-1.3043646	0.00557103
ENSG00000165507	2.68822185	5.35E-10
ENSG00000165655	-1.4401986	0.00018543
ENSG00000166289	1.43160004	5.80E-06
ENSG00000166401	-1.2488364	1.91E-07
ENSG00000166483	-1.3029105	0.00112823
ENSG00000166592	-1.5028687	3.45E-06
ENSG00000166860	-1.2529595	4.87E-05
ENSG00000166886	-3.1322613	1.50E-18
ENSG00000166924	1.27306501	0.03308276
ENSG00000167470	-1.7586184	7.10E-16
ENSG00000167565	-1.537435	8.92E-10

Gene ID	FC 0.5NG - 1NG	adj p-value 0.5NG - 1NG
ENSG00000167772	-1.9830842	6.01E-09
ENSG00000168026	-1.3223663	0.00861299
ENSG00000168209	-1.4111802	0.00371112
ENSG00000168386	-1.3490454	0.00273055
ENSG00000168646	1.47390999	0.00024954
ENSG00000168672	1.28936446	0.00078026
ENSG00000168795	-1.3650531	0.00010779
ENSG00000168874	-1.8480193	4.02E-08
ENSG00000168994	-1.2727792	9.34E-07
ENSG00000169242	-1.3277643	0.03494292
ENSG00000169635	-1.2717133	0.00741009
ENSG00000169926	-1.3939083	1.91E-07
ENSG00000169991	-1.3692843	1.70E-08
ENSG00000170100	-1.2414249	0.00187111
ENSG00000170345	3.28187788	8.13E-11
ENSG00000170385	-1.2743474	0.04027477
ENSG00000170525	-1.3106546	1.79E-09
ENSG00000170775	-1.3227949	9.55E-07
ENSG00000170852	-1.3019996	8.39E-07
ENSG00000171044	-1.2974793	0.00082151
ENSG00000171150	1.33438016	0.00012598
ENSG00000171223	-1.7490921	1.79E-12
ENSG00000171246	-2.1403079	5.90E-09
ENSG00000171522	1.2434908	5.83E-05
ENSG00000171604	-1.697147	2.74E-06
ENSG00000171617	-1.3696357	8.33E-06
ENSG00000171621	-1.4507675	0.00180964
ENSG00000172059	-1.3528377	0.00031997
ENSG00000172602	-1.5358709	0.0001683
ENSG00000172819	-1.2694972	0.00526487
ENSG00000173013	-1.2842841	0.02686126
ENSG00000173214	1.21082735	0.03696823

Gene ID	FC 0.5NG - 1NG	adj p-value 0.5NG - 1NG
ENSG00000173276	-1.2909386	6.94E-05
ENSG00000173281	-1.2530588	9.31E-06
ENSG00000173846	-1.3781988	1.01E-05
ENSG00000175105	-1.5091044	0.00164036
ENSG00000175376	-1.2072289	2.01E-05
ENSG00000175505	-1.3699634	0.00119384
ENSG00000175592	-1.4468275	1.34E-06
ENSG00000176170	-1.2318551	0.00057921
ENSG00000176182	1.23833592	0.01309005
ENSG00000176678	-1.2816718	0.0094202
ENSG00000176692	-1.8004577	1.45E-11
ENSG00000176842	-1.3090945	0.00429191
ENSG00000176845	-1.5928198	1.69E-07
ENSG00000176907	-1.501936	0.00109092
ENSG00000177125	-1.4672295	1.92E-08
ENSG00000177303	-1.2664775	4.02E-06
ENSG00000177426	1.24812415	4.05E-05
ENSG00000177679	-1.3708863	0.00230303
ENSG00000179094	-1.215349	0.00458451
ENSG00000179144	1.31281741	4.28E-06
ENSG00000179348	-1.8346311	2.57E-10
ENSG00000179361	-1.3628641	0.00019045
ENSG00000179388	-1.889771	0.00180949
ENSG00000179403	-1.3546249	0.01388532
ENSG00000179604	-1.2221092	0.00123657
ENSG00000180035	-1.3128221	5.41E-05
ENSG00000180592	-1.3517298	0.0028506
ENSG00000180730	-1.3087165	0.00029956
ENSG00000180884	1.28391927	0.00130496
ENSG00000181274	1.35130627	0.00012402
ENSG00000181472	-1.6522819	3.01E-08
ENSG00000182185	-1.2602248	0.04028815

Gene ID	FC 0.5NG - 1NG	adj p-value 0.5NG - 1NG
ENSG00000182310	-1.2939935	0.01653732
ENSG00000182704	-1.2174128	0.00052057
ENSG00000183054	-1.3082975	0.02394706
ENSG00000183072	-1.3464462	0.00016204
ENSG00000183161	1.31746084	0.00131228
ENSG00000183307	-1.4858008	9.19E-05
ENSG00000183337	-2.2524675	1.05E-11
ENSG00000183691	-2.3745838	1.97E-10
ENSG00000184307	-1.4456482	0.001524
ENSG00000184402	-1.2188536	0.00185144
ENSG00000184557	-1.4723786	3.5179E-05
ENSG00000184588	1.32680896	3.34E-07
ENSG00000184898	1.24017694	0.01752304
ENSG00000184916	-1.36321	3.28E-07
ENSG00000185022	-1.3001328	5.49E-06
ENSG00000185269	-1.2215968	0.01047135
ENSG00000186480	-1.3692711	0.00089293
ENSG00000187479	-1.304525	0.03744891
ENSG00000187678	-1.7393853	6.38E-13
ENSG00000188064	-1.2229826	3.94E-05
ENSG00000188290	-1.2713977	0.00790108
ENSG00000188483	-2.3170068	2.00E-18
ENSG00000188522	-1.7287134	3.30E-11
ENSG00000188997	1.31186876	0.00026871
ENSG00000189298	1.312415	0.00772623
ENSG00000196227	1.25400889	0.02502816
ENSG00000196428	-1.3933037	8.72E-11
ENSG00000196449	-1.3100375	1.07E-07
ENSG00000196456	1.23929988	0.00775449
ENSG00000196843	-1.6542594	9.13E-09
ENSG00000197019	-1.8479655	1.98E-14
ENSG00000197050	1.2605483	0.00987635
ENSG00000197063	-1.2683027	1.02E-06
ENSG00000197182	-1.3036661	0.04408613
ENSG00000197183	-1.3013983	3.50E-09
ENSG00000197329	-1.2593486	0.00205593
ENSG00000197461	-2.1746285	4.33E-15
ENSG00000197557	1.25940387	0.00982847
ENSG00000197566	1.21503048	0.04118216
ENSG00000197951	1.2809524	0.00092248
ENSG00000198142	-1.2844815	0.00602499
ENSG00000198182	1.25249134	0.01033343
ENSG00000198324	-1.2819239	1.75E-05
ENSG00000198331	1.20160054	0.01865547
ENSG00000198355	-1.2162363	0.00169038
ENSG00000198435	-1.7171577	1.79E-08
ENSG00000198468	-1.2174203	0.03875876
ENSG00000198517	-1.648715	1.09E-12
ENSG00000203709	1.68794655	4.98E-05
ENSG00000203883	-1.4333936	0.00146393
ENSG00000212195	1.2568459	0.0145791
ENSG00000213203	1.31058237	0.0002319
ENSG00000213859	-1.2207811	0.00953891
ENSG00000223039	-1.2308261	0.00128561
ENSG00000223086	1.20340062	0.01089607
ENSG00000225614	-1.2107398	0.00044791
ENSG00000229729	-1.5614126	1.70E-06
ENSG00000237440	1.25364939	0.01387053
ENSG00000237989	-1.2455243	0.00078128
ENSG00000240891	1.29738154	0.00654056
ENSG00000241839	-1.2158441	0.0003902
ENSG00000241852	-1.2102474	0.04737952
ENSG00000242829	-1.3445999	0.00019694
ENSG00000248476	-1.2000941	0.00693981
ENSG00000251493	-1.381758	0.00429042
ENSG00000253368	-1.3686977	6.22E-08
ENSG00000253958	-1.2741693	0.00753529
ENSG00000254470	-1.2708721	4.30E-07
ENSG00000255176	-1.5836323	0.00271514
ENSG00000256325	1.27809421	0.00478948
ENSG00000256448	-1.2005675	0.00065377
ENSG00000259330	-1.234081	0.01407036
ENSG00000259863	-1.2299296	0.04218538
ENSG00000260428	-1.2472198	0.01324838
ENSG00000263394	-1.2926154	0.01090127
ENSG00000264175	1.20192854	0.04085895
ENSG00000266533	-1.2598207	0.00107293
ENSG00000269906	-1.2073328	0.02467462
ENSG00000272447	-1.2282995	0.01033535
ENSG00000272574	-1.2858478	0.00128761
ENSG00000273489	-1.8559085	1.31E-06
ENSG00000273820	-1.3369602	0.00334911
ENSG00000274349	1.38739803	0.00087431
ENSG00000274636	-1.5610035	0.00052489
ENSG00000274963	1.2034221	0.00089332
ENSG00000274995	-1.747399	1.17E-05
ENSG00000275031	-1.413993	0.00023406
ENSG00000275202	-1.3698719	0.0021636
ENSG00000275993	-2.1517288	1.60E-11
ENSG00000278133	1.23697245	0.04207857
ENSG00000278576	-1.2175494	0.03398816
ENSG00000279407	-1.2732192	0.04608071
ENSG00000279637	-1.2178943	2.85E-05
ENSG00000279673	1.27279341	0.01887308
ENSG00000279692	-1.304791	0.00556057
ENSG00000279838	1.41019484	0.00023192

Supplementary Table 2b. Differentially expressed genes between 1hr and 4hr normal glucose samples

Ensembl gene IDs, Fold change (1NG-4NG) and FDR adjusted p-values of DEGs

Gene ID	FC 1NG - 4NG	adj p-value 1NG - 4NG
ENSG00000000457	1.32672022	0.00049112
ENSG00000001167	1.32695775	8.64E-08
ENSG00000002016	1.2888564	0.01858958
ENSG00000002587	1.66694035	6.20E-09
ENSG00000003400	-1.3054985	3.62E-07
ENSG00000003989	1.89264974	5.56E-08
ENSG00000004139	-1.3025197	0.00066842
ENSG00000004660	-1.5524307	1.60E-06
ENSG00000004799	-1.2982462	0.00797289
ENSG00000005513	-2.7506822	6.69E-13
ENSG00000006016	1.21026147	0.02795851
ENSG00000006062	-1.3064754	0.0034375
ENSG00000006576	1.25104516	0.01836165
ENSG00000007341	1.2140244	0.02072397
ENSG00000007384	-1.5397685	2.18E-07
ENSG00000007908	9.79817179	1.21E-14
ENSG00000007968	-1.3295743	0.0257927
ENSG00000008083	-1.5430226	3.42E-08
ENSG00000008283	-1.2380044	1.79E-06
ENSG00000008294	1.45114762	1.11E-16
ENSG00000010810	-1.3182772	2.07E-09
ENSG00000011201	1.21231159	0.01874102
ENSG00000011422	-1.2583615	0.00026264
ENSG00000012048	-1.2698384	0.00640241
ENSG00000012174	1.21090367	0.00869009
ENSG00000013588	-1.3806822	1.32E-05
ENSG00000013810	-1.2759843	0.0059974
ENSG00000014123	1.22594782	0.00055159
ENSG00000015133	-2.2414317	5.07E-16
ENSG00000015568	1.29027171	0.02432301
ENSG00000019144	1.22962642	4.01E-05
ENSG00000019549	-1.5188974	3.50E-05

Gene ID	FC 1NG - 4NG	adj p-value 1NG - 4NG
ENSG00000020577	1.34042229	1.30E-12
ENSG00000023902	-1.2025328	0.00348502
ENSG00000026508	1.22355847	9.6004E-05
ENSG00000026559	1.24350366	0.02823091
ENSG00000026950	-1.2373122	0.00055455
ENSG00000028137	-1.2824014	0.00083874
ENSG00000028277	-1.4797807	2.23E-05
ENSG00000031081	1.44394607	4.74E-15
ENSG00000033327	-1.4544693	6.07E-14
ENSG00000034677	-1.2778629	0.00262725
ENSG00000035499	-1.250739	0.00711886
ENSG00000038295	1.5183233	9.95E-06
ENSG00000038382	1.24783514	2.10E-08
ENSG00000043143	-1.9889611	1.13E-13
ENSG00000047617	-1.238026	0.01504024
ENSG00000047932	1.35377156	0.00044149
ENSG00000048052	1.9024184	1.29E-06
ENSG00000048405	-1.2135462	0.0257093
ENSG00000049130	1.81644022	0.00013176
ENSG00000049192	1.37605307	7.22E-08
ENSG00000049283	-1.6754888	0.00026769
ENSG00000049759	1.63894623	2.10E-11
ENSG00000049883	1.25746494	0.00147331
ENSG00000050820	-1.3283376	5.16E-09
ENSG00000051180	-1.2642298	0.02552674
ENSG00000052749	1.28265565	3.93E-07
ENSG00000053747	-1.2208788	0.00320359
ENSG00000054523	1.24333545	3.35E-07
ENSG00000054598	-1.7815162	1.04E-10
ENSG00000054967	-2.0716239	4.34E-12
ENSG00000056558	1.92482942	0.00046494
ENSG00000057019	1.37862653	5.13E-06

Gene ID	FC 1NG - 4NG	adj p-value 1NG - 4NG
ENSG00000057294	-1.5415153	0.00014809
ENSG00000057704	-1.7624024	1.28E-14
ENSG00000058091	1.32000049	6.37E-06
ENSG00000058453	-1.2113027	0.01525107
ENSG00000059378	-1.2156265	0.00057177
ENSG00000059728	-1.295918	0.00026865
ENSG00000063438	-1.2341081	0.00442346
ENSG00000064692	-1.8736294	1.47E-05
ENSG00000064989	1.5813165	3.46E-05
ENSG00000064999	-1.2351827	0.0013442
ENSG00000065057	-1.2370125	0.0006562
ENSG00000065308	-1.599087	3.61E-15
ENSG00000065559	1.21177972	0.00087421
ENSG00000065802	-1.243084	1.58E-08
ENSG00000066027	-1.2799783	0.0006758
ENSG00000066084	1.29209573	3.75E-06
ENSG00000066735	-1.6488268	3.12E-10
ENSG00000067082	1.22158102	0.00053948
ENSG00000067182	-1.2250821	6.13E-07
ENSG00000067221	1.23234646	0.00372663
ENSG00000067798	1.80721297	5.47E-19
ENSG00000067955	1.69484055	3.15E-09
ENSG00000068024	-1.2002077	0.00039094
ENSG00000068078	1.59993192	2.82E-08
ENSG00000068137	-1.5328673	4.07E-05
ENSG00000068305	1.42111169	1.90E-07
ENSG00000068366	1.22647244	0.00015578
ENSG00000068724	-1.2193321	0.00120341
ENSG00000069399	-1.4116019	1.26E-05
ENSG00000069667	-1.3894936	0.00543354
ENSG00000069702	-1.3667103	0.00021418
ENSG00000069812	-1.3350365	6.55E-07

Gene ID	FC 1NG - 4NG	adj p-value 1NG - 4NG
ENSG00000070444	-1.2090372	0.00662978
ENSG00000070610	-2.1821313	3.70E-12
ENSG00000070614	-1.2913307	4.05E-09
ENSG00000070718	1.24699957	0.00031334
ENSG00000070961	1.52929049	3.82E-07
ENSG00000071073	-1.3274055	3.15E-06
ENSG00000071246	-1.5253512	3.13E-05
ENSG00000072201	-1.3253983	0.0009973
ENSG00000072422	1.3002404	3.05E-07
ENSG00000072756	1.24860742	0.0007462
ENSG00000072840	-1.2139345	0.00017213
ENSG00000073008	1.33862729	5.86E-10
ENSG00000073111	-1.2209579	0.00728977
ENSG00000073417	1.64617698	1.89E-16
ENSG00000073711	-1.2627259	0.00305974
ENSG00000073712	1.215063	1.68E-06
ENSG00000073756	-2.0236629	0.00203247
ENSG00000073921	1.33723829	2.39E-05
ENSG00000074047	2.90053388	4.04E-16
ENSG00000074855	-1.3390994	0.00050233
ENSG00000075218	-1.2165744	0.0376062
ENSG00000075340	1.44070276	0.00040191
ENSG00000075426	-1.9231916	9.63E-17
ENSG00000075539	1.22186267	0.00385642
ENSG00000075651	-1.4763951	8.21E-10
ENSG00000075702	-1.3554003	0.00083359
ENSG00000075711	-1.2917993	9.72E-06
ENSG00000076351	-1.5515496	1.06E-07
ENSG00000076356	1.21693037	8.90E-06
ENSG00000076382	-1.2240165	0.00249571
ENSG00000078401	-1.256175	0.00022247
ENSG00000078747	1.21949011	4.19E-05
ENSG00000079102	1.45651648	6.71E-05
ENSG00000079308	1.21706328	0.00049491
ENSG00000079335	1.22618125	0.00248158
ENSG00000079337	-1.50479	4.12E-06
ENSG00000079616	-1.2243388	0.00440415
ENSG00000079841	1.22756004	0.04007252
ENSG00000080561	-1.3269782	1.24E-07
ENSG00000080608	1.25005092	2.10E-06
ENSG00000080709	1.25945258	0.00012272
ENSG00000080986	-1.2709533	0.00927981
ENSG00000081014	1.23190787	0.01322079
ENSG00000081059	1.6202553	1.89E-06
ENSG00000081386	1.24524147	0.00018266
ENSG00000082126	-1.2665279	0.00014405
ENSG00000082438	1.2755699	0.00014971
ENSG00000082512	-1.31517	0.00571818
ENSG00000082516	1.20611716	0.00030079
ENSG00000083812	-1.2033549	0.00567619
ENSG00000084112	-1.2044456	0.0001216
ENSG00000084710	1.98855854	2.04E-09
ENSG00000085185	-1.3834893	7.41E-05
ENSG00000085721	1.20932572	0.03785138
ENSG00000086015	-1.2227358	5.0874E-05
ENSG00000087095	1.2588579	2.99E-05
ENSG00000087266	-1.3939846	8.53E-08
ENSG00000087903	1.41445713	0.0056712
ENSG00000088179	-1.2631664	0.00089271
ENSG00000088325	-1.2404561	0.00215907
ENSG00000088387	-1.3304837	5.65E-10
ENSG00000088448	-1.2880038	0.00218158
ENSG00000088808	-1.2517098	0.00015375
ENSG00000088836	-1.272924	0.00069611
ENSG00000088970	-1.353917	0.00212719
ENSG00000088986	1.25558429	4.07E-10
ENSG00000089041	-1.7698575	2.62E-06
ENSG00000089335	1.21126354	0.03339966
ENSG00000089351	-1.2211072	0.00155847
ENSG00000089820	-1.2401513	0.02444775
ENSG00000090776	-1.3717359	2.50E-08
ENSG00000090889	-1.2653942	0.00255549
ENSG00000091129	1.56520831	2.04E-11
ENSG00000091428	1.43133521	9.77E-06
ENSG00000091656	1.64271434	1.40E-09
ENSG00000091844	-1.3598858	0.00124298
ENSG00000091879	1.49998884	1.57E-10
ENSG00000091972	-1.2371465	0.02959153
ENSG00000092470	-1.224011	0.0158138
ENSG00000092969	1.43513673	0.00578282
ENSG00000094804	-1.2787537	0.00260475
ENSG00000095015	1.26907576	5.82E-05
ENSG00000095066	-1.3590591	0.01765958
ENSG00000095370	-1.5716252	3.18E-11
ENSG00000095383	-1.3172472	0.00123078
ENSG00000095397	1.240079	0.00922593
ENSG00000096093	-1.2496849	0.0024239
ENSG00000096433	-1.2416775	3.11E-07
ENSG00000096654	1.385417	0.00075183
ENSG00000097096	-1.4234341	0.00057833
ENSG00000099204	-1.3592171	2.96E-07
ENSG00000099282	-1.2903112	0.0011216
ENSG00000099625	-1.2653345	0.01273157
ENSG00000099822	1.34076484	0.02257033
ENSG00000099860	-3.2674095	1.71E-17
ENSG00000099875	-1.4239848	2.67E-09

Gene ID	FC 1NG - 4NG	adj p-value 1NG - 4NG
ENSG00000099889	-1.6864785	1.61E-10
ENSG00000099910	-1.2080838	0.0212908
ENSG00000100027	-1.3823306	0.00592024
ENSG00000100034	-1.3621723	4.27E-11
ENSG00000100036	-1.584154	3.80E-10
ENSG00000100060	-1.2140828	4.82E-05
ENSG00000100065	-1.4254671	4.68E-08
ENSG00000100077	1.25920325	0.01190783
ENSG00000100151	-1.2072137	0.02973847
ENSG00000100276	-1.2629787	0.00897558
ENSG00000100292	-1.4907155	1.21E-07
ENSG00000100307	-1.2997643	0.02647355
ENSG00000100344	1.39105256	0.00066121
ENSG00000100350	-1.2185301	0.0017449
ENSG00000100368	1.22827122	0.00257069
ENSG00000100439	-1.2956143	0.00171136
ENSG00000100490	-1.2701994	0.00674859
ENSG00000100523	1.21002327	0.01174951
ENSG00000100580	1.23006407	0.00011368
ENSG00000100592	1.38522039	1.76E-10
ENSG00000100625	-1.3628381	0.00147272
ENSG00000100647	-1.3351041	7.18E-12
ENSG00000100650	-1.2172058	5.23E-05
ENSG00000100906	-1.8870069	1.29E-13
ENSG00000100968	-1.232764	0.00150376
ENSG00000101096	-1.4620962	6.23E-08
ENSG00000101187	-1.3322345	0.00344076
ENSG00000101188	-1.3110526	3.31E-05
ENSG00000101224	-1.3599467	3.27E-07
ENSG00000101255	-1.6133912	5.56E-12
ENSG00000101265	-1.6279973	1.43E-10
ENSG00000101412	-1.360897	0.00149355

Gene ID	FC 1NG - 4NG	adj p-value 1NG - 4NG
ENSG00000101445	-2.2087472	1.43E-15
ENSG00000101577	-1.3386739	5.03E-10
ENSG00000101665	-4.1407732	4.47E-14
ENSG00000101670	2.23169628	2.09E-17
ENSG00000101850	-1.2838081	0.01636427
ENSG00000101871	-1.6634144	3.31E-14
ENSG00000102010	-1.6353892	1.22E-11
ENSG00000102034	-1.2028131	0.00031863
ENSG00000102098	-1.3807378	0.00011715
ENSG00000102287	-1.4059685	0.00809744
ENSG00000102384	-1.2099395	0.04723423
ENSG00000102531	1.51885594	2.27E-15
ENSG00000102554	1.23782344	0.00445191
ENSG00000102699	-1.2359134	1.08E-09
ENSG00000102755	1.87795393	3.14E-16
ENSG00000102760	-1.3421696	0.01776551
ENSG00000102890	-1.5829245	0.00033019
ENSG00000102935	1.81623349	7.04E-07
ENSG00000103111	-1.2448608	1.77E-08
ENSG00000103241	-1.5827261	5.20E-05
ENSG00000103257	-1.6864538	5.56E-14
ENSG00000103343	1.30689385	0.00016448
ENSG00000103888	1.30525843	0.00011223
ENSG00000103995	-1.2308858	0.0480193
ENSG00000104081	-2.4376295	5.17E-07
ENSG00000104147	-1.2458839	0.03960053
ENSG00000104221	1.20073441	0.01810186
ENSG00000104312	1.31625036	1.44E-05
ENSG00000104611	1.33899059	0.00200449
ENSG00000104643	1.21467818	0.00429191
ENSG00000104689	-1.2365772	2.34E-06
ENSG00000104856	1.32342157	0.00321345

Gene ID	FC 1NG - 4NG	adj p-value 1NG - 4NG
ENSG00000104951	1.4517265	0.00027483
ENSG00000105011	-1.2269439	0.0111765
ENSG00000105072	1.24914224	0.00844121
ENSG00000105088	1.80566367	9.38E-11
ENSG00000105287	-1.5168855	1.10E-11
ENSG00000105321	-1.2616983	0.00056409
ENSG00000105339	-1.2953473	2.21E-07
ENSG00000105443	-1.3012715	9.83E-05
ENSG00000105514	-1.2272792	4.81E-05
ENSG00000105643	-1.2671437	0.02006921
ENSG00000105656	-1.328102	1.07E-06
ENSG00000105662	-1.3508229	6.25E-05
ENSG00000105722	-1.2670243	0.00017194
ENSG00000105738	-1.2107838	3.94E-05
ENSG00000105793	1.26325448	0.02029859
ENSG00000105810	1.87472266	5.04E-12
ENSG00000105825	1.37166642	7.44E-11
ENSG00000105829	1.22265339	0.01207465
ENSG00000105849	1.21379544	0.03639646
ENSG00000105851	-1.346963	2.64E-06
ENSG00000105855	2.27275077	1.05E-13
ENSG00000105875	1.20422737	0.009879
ENSG00000105976	-1.2099769	4.21E-06
ENSG00000106003	-3.0140296	3.92E-13
ENSG00000106070	2.36142081	3.02E-22
ENSG00000106089	-1.6291806	1.22E-06
ENSG00000106100	-1.3528417	5.23E-07
ENSG00000106351	-1.6569452	3.80E-10
ENSG00000106537	1.35762543	2.37E-06
ENSG00000106546	1.27492772	9.72E-07
ENSG00000106571	-1.4915604	3.10E-11
ENSG00000106789	-1.2826703	0.01521899

Gene ID	FC 1NG - 4NG	adj p-value 1NG - 4NG
ENSG00000106829	-1.2803066	3.60E-07
ENSG00000106852	-1.7626909	6.71E-09
ENSG00000106948	-1.3026728	0.00016428
ENSG00000106993	1.21875905	0.00843032
ENSG00000107099	1.31923635	0.00355575
ENSG00000107185	-1.29098	4.11E-07
ENSG00000107485	-1.478977	7.40E-08
ENSG00000107551	-1.468779	0.00041461
ENSG00000107562	3.05330305	2.12E-18
ENSG00000107679	1.21901419	3.63E-05
ENSG00000107719	-1.7332165	2.25E-09
ENSG00000107731	2.28676721	4.78E-11
ENSG00000107771	1.31571653	6.14E-05
ENSG00000107807	-1.3782693	0.00312666
ENSG00000107863	1.5519926	8.19E-17
ENSG00000107984	1.85647577	4.71E-11
ENSG00000108219	-1.3570121	1.61E-12
ENSG00000108262	-1.3890207	1.26E-10
ENSG00000108312	1.25540995	1.33E-06
ENSG00000108433	1.20345164	0.00078719
ENSG00000108551	-1.5627114	6.49E-08
ENSG00000108622	-1.2072829	6.13E-07
ENSG00000108691	-1.3639047	0.01662599
ENSG00000108733	1.33306028	0.00053338
ENSG00000108846	-1.2961468	0.02138189
ENSG00000108932	-1.2770614	0.00029907
ENSG00000108960	1.43214546	0.00021139
ENSG00000108984	-1.278558	0.03219403
ENSG00000109046	-1.2448939	0.0016995
ENSG00000109118	1.32076897	4.05E-08
ENSG00000109133	1.22869005	0.01486887
ENSG00000109189	1.27126188	0.00063156

Gene ID	FC 1NG - 4NG	adj p-value 1NG - 4NG
ENSG00000109320	1.41850462	2.22E-16
ENSG00000109321	1.27587881	0.00017112
ENSG00000109458	-1.3303987	0.00061828
ENSG00000109501	-1.4926009	2.83E-08
ENSG00000109618	1.2403847	0.02568225
ENSG00000109685	-1.2005899	3.57E-06
ENSG00000109689	1.244577	0.00012357
ENSG00000109787	-1.4531937	3.67E-12
ENSG00000109971	1.22942299	2.86E-10
ENSG00000110047	-1.2871804	5.76E-07
ENSG00000110080	-1.30558	7.74E-07
ENSG00000110318	1.39287168	0.0024832
ENSG00000110330	1.22111899	0.02558953
ENSG00000110852	1.34873491	0.01627017
ENSG00000110931	-1.2737291	2.31E-10
ENSG00000111057	-1.2308975	0.00022969
ENSG00000111077	-1.4369481	2.94E-09
ENSG00000111110	-1.6019264	0.00107448
ENSG00000111145	-1.3118036	6.61E-11
ENSG00000111252	-1.2732421	8.78E-13
ENSG00000111276	1.32833796	1.90E-05
ENSG00000111335	-1.3439516	0.01145556
ENSG00000111339	1.51717118	1.42E-07
ENSG00000111647	1.26912725	0.04191205
ENSG00000111665	-1.2576819	0.03962587
ENSG00000111671	1.32202184	0.00324676
ENSG00000111674	-1.2426374	0.03736336
ENSG00000111725	-1.329125	3.3991E-05
ENSG00000111727	1.370463	3.41E-05
ENSG00000111817	1.25542795	6.19E-05
ENSG00000111846	-1.4344094	9.69E-09
ENSG00000111907	1.3654544	0.00081229

Gene ID	FC 1NG - 4NG	adj p-value 1NG - 4NG
ENSG00000111912	1.23969061	3.63E-05
ENSG00000111961	-1.6804475	7.91E-23
ENSG00000112033	1.34313192	1.26E-08
ENSG00000112137	-1.351138	4.18E-06
ENSG00000112208	1.23036198	0.02639712
ENSG00000112210	1.68597893	7.41E-12
ENSG00000112297	-1.7276655	1.39E-08
ENSG00000112305	1.32538176	4.97E-07
ENSG00000112414	-1.2506046	0.00664115
ENSG00000112541	1.77810179	9.37E-08
ENSG00000112559	1.34331471	0.00019789
ENSG00000112715	-2.4431358	6.70E-13
ENSG00000112773	1.70773184	0.0008082
ENSG00000112837	1.28348631	0.00020677
ENSG00000112851	1.24359074	0.00941817
ENSG00000112855	1.20328965	0.00010682
ENSG00000112984	-1.2335617	0.00150322
ENSG00000113070	-2.0786792	3.71E-12
ENSG00000113319	1.23697561	0.00062643
ENSG00000113368	-1.254197	5.65E-06
ENSG00000113369	1.75266455	2.80E-10
ENSG00000113448	-1.2406913	0.00728115
ENSG00000113532	1.66663314	9.75E-06
ENSG00000113552	-1.2379538	2.33E-08
ENSG00000113555	-1.7730339	3.83E-07
ENSG00000113580	1.88374925	8.47E-14
ENSG00000113594	1.2164743	0.01160475
ENSG00000113749	-1.3999332	0.00737171
ENSG00000114019	-1.6481753	9.69E-13
ENSG00000114166	-1.3902331	6.57E-05
ENSG00000114315	-14.257731	5.49E-22
ENSG00000114346	-1.2211454	0.01314324

Gene ID	FC 1NG - 4NG	adj p-value 1NG - 4NG
ENSG00000114423	1.6384172	6.73E-10
ENSG00000114626	-1.2919632	0.03400417
ENSG00000114698	-1.2738138	0.00176504
ENSG00000114812	-1.7452352	3.03E-05
ENSG00000115084	1.21173322	0.01438806
ENSG00000115170	1.2518159	2.99E-08
ENSG00000115183	-1.2699031	1.18E-07
ENSG00000115290	1.36816476	6.9969E-05
ENSG00000115368	1.23189677	0.00053948
ENSG00000115525	-1.515937	6.70E-05
ENSG00000115602	2.25518572	1.35E-07
ENSG00000115604	1.56115901	1.65E-06
ENSG00000115641	-1.5070221	2.45E-12
ENSG00000115738	-2.7820409	1.35E-09
ENSG00000115844	-2.6094053	4.04E-14
ENSG00000115896	1.40810231	0.00443484
ENSG00000115902	-1.2510663	7.39E-05
ENSG00000115946	1.21378174	0.00012202
ENSG00000116017	-1.463567	0.00012395
ENSG00000116106	-1.2022215	0.00031872
ENSG00000116273	-1.374827	1.37E-07
ENSG00000116285	-2.25919	2.83E-16
ENSG00000116514	-1.2498011	1.95E-05
ENSG00000116584	-1.2780899	7.60E-08
ENSG00000116604	-1.381966	3.09E-07
ENSG00000116641	1.3725879	1.11E-05
ENSG00000116711	2.36449824	2.34E-10
ENSG00000116717	1.24756013	9.70E-05
ENSG00000116731	-1.2099182	1.41E-05
ENSG00000116741	1.23208249	0.03090727
ENSG00000116991	-1.3835764	1.22E-06
ENSG00000117036	-1.2179898	4.17E-06

Gene ID	FC 1NG - 4NG	adj p-value 1NG - 4NG
ENSG00000117114	1.23178853	2.55E-06
ENSG00000117143	1.24684659	6.19E-06
ENSG00000117152	1.39010539	3.21E-06
ENSG00000117174	1.22553194	0.00170423
ENSG00000117318	-1.9568775	1.11E-14
ENSG00000117399	-1.2654614	0.00230287
ENSG00000117525	-2.2492119	3.02E-10
ENSG00000117595	2.77531175	8.96E-11
ENSG00000117597	1.23568663	5.25E-06
ENSG00000117650	-1.243857	0.01031094
ENSG00000117724	-1.2451363	0.00567637
ENSG00000118193	-1.2915714	0.00342871
ENSG00000118260	1.22025979	0.00218114
ENSG00000118402	1.46516528	0.00030516
ENSG00000118515	-1.5169137	4.84E-16
ENSG00000118922	-1.3500476	0.00017701
ENSG00000118946	3.3115781	7.82E-09
ENSG00000118971	-1.2814999	0.03658706
ENSG00000118985	1.52073562	1.41E-11
ENSG00000119138	1.21474195	0.00094982
ENSG00000119139	1.26976829	3.37E-07
ENSG00000119508	-3.3565757	2.34E-11
ENSG00000119522	-1.2028973	0.00062449
ENSG00000119599	1.2096539	0.00311581
ENSG00000119630	-1.8123371	1.69E-12
ENSG00000119669	-2.5003631	4.46E-18
ENSG00000119686	-1.259288	0.00674802
ENSG00000119771	1.36772109	1.12E-09
ENSG00000119778	1.21506001	0.00523077
ENSG00000119900	-1.2753343	0.00360754
ENSG00000119927	1.26765044	0.00048475
ENSG00000119938	1.65845421	5.74E-07

Gene ID	FC 1NG - 4NG	adj p-value 1NG - 4NG
ENSG00000119965	1.24717614	0.00542052
ENSG00000120129	-2.4978321	2.09E-16
ENSG00000120278	-2.4481096	5.24E-22
ENSG00000120539	-1.2046193	0.02372037
ENSG00000120693	2.94058459	2.57E-16
ENSG00000120696	1.2922274	0.04266669
ENSG00000120738	-9.3988949	5.93E-16
ENSG00000120784	1.34127384	0.00106639
ENSG00000120800	1.20293619	0.00118321
ENSG00000120875	-1.5998431	4.79E-15
ENSG00000120899	-1.2071891	0.03925495
ENSG00000121060	-1.2117965	2.49E-06
ENSG00000121152	-1.3043459	0.00282922
ENSG00000121236	-1.2783977	0.03976697
ENSG00000121406	1.38842056	5.1279E-05
ENSG00000121486	1.21524595	0.0122018
ENSG00000121797	-1.2849569	0.00094118
ENSG00000121858	-2.0063189	1.06E-07
ENSG00000121903	1.34016812	0.00493549
ENSG00000121957	-1.3500886	3.68E-05
ENSG00000121964	-1.2307829	0.00439817
ENSG00000121966	-1.7667466	4.95E-11
ENSG00000122068	1.24389374	4.57E-06
ENSG00000122257	-1.2305667	7.09E-07
ENSG00000122435	1.30256198	0.00194098
ENSG00000122641	1.72875909	7.63E-09
ENSG00000122786	1.28842579	4.23E-10
ENSG00000122861	-2.3097982	2.63E-15
ENSG00000122870	1.45262937	2.10E-05
ENSG00000122877	-4.804349	2.33E-14
ENSG00000122966	-1.255872	0.00098952
ENSG00000123066	1.24090867	2.69E-07

Gene ID	FC 1NG - 4NG	adj p-value 1NG - 4NG
ENSG00000123080	-1.2779709	0.00068106
ENSG00000123146	-1.3528853	0.00012678
ENSG00000123358	-6.0444276	3.00E-16
ENSG00000123473	-1.2147208	0.00946269
ENSG00000123485	-1.3157897	0.01786201
ENSG00000123545	1.28236866	0.01391875
ENSG00000123700	1.93571	9.08E-06
ENSG00000123836	1.28339617	0.0413806
ENSG00000123870	1.21984407	0.01267658
ENSG00000124216	-5.7634081	1.60E-24
ENSG00000124225	1.45030403	0.00021552
ENSG00000124593	-1.2753019	0.04049097
ENSG00000124610	1.25205031	0.009588
ENSG00000124762	-1.3287158	5.51E-13
ENSG00000124772	1.66639368	5.13E-07
ENSG00000125089	-1.6045504	6.91E-11
ENSG00000125257	1.20251321	0.00103154
ENSG00000125266	1.26654848	0.00502881
ENSG00000125347	-1.3734482	0.00283687
ENSG00000125378	1.66217926	8.20E-08
ENSG00000125629	-1.2838492	0.0266455
ENSG00000125740	-8.965634	1.80E-13
ENSG00000125812	-1.2565604	4.84E-05
ENSG00000125845	1.30662239	0.0029513
ENSG00000125846	1.26971985	0.00225888
ENSG00000125945	1.42528818	6.69E-08
ENSG00000125966	1.65851312	1.92E-06
ENSG00000125968	-2.7442476	5.85E-12
ENSG00000126351	-1.4233276	1.52E-09
ENSG00000126603	-1.2629064	0.00311071
ENSG00000126705	-1.5072288	4.28E-07
ENSG00000126778	-1.2684321	0.03248663

Gene ID	FC 1NG - 4NG	adj p-value 1NG - 4NG
ENSG00000126878	-1.2159528	0.00457102
ENSG00000126882	-1.6381106	1.04E-05
ENSG00000127124	1.33032837	0.00103312
ENSG00000127220	-1.4487816	6.30E-05
ENSG00000127328	-1.3899805	3.21E-09
ENSG00000127334	-1.2035817	0.00370733
ENSG00000127511	-1.2273543	4.55E-07
ENSG00000127528	-1.2607245	0.02325261
ENSG00000127533	-3.402721	5.54E-18
ENSG00000127564	-1.2404068	0.04444947
ENSG00000128016	-2.1234089	1.99E-09
ENSG00000128284	-1.4130067	2.5504E-05
ENSG00000128294	-1.2310482	8.07E-09
ENSG00000128342	-1.7894955	6.93E-08
ENSG00000128394	-1.2195547	0.04949947
ENSG00000128487	1.30024867	0.00433021
ENSG00000128567	1.41119438	3.14E-10
ENSG00000128641	-1.2616184	0.00103872
ENSG00000128805	-1.4649757	5.65E-12
ENSG00000128815	-1.3551779	0.0004311
ENSG00000128833	-1.3326909	0.00121829
ENSG00000128849	-1.4193357	8.02E-09
ENSG00000128881	-1.2409235	0.00235066
ENSG00000128917	-5.2636033	2.50E-20
ENSG00000128923	-1.2862389	0.00018081
ENSG00000129116	1.21331204	0.01846913
ENSG00000129173	-1.2279803	0.01946881
ENSG00000129195	-1.2088142	0.02899808
ENSG00000129422	-1.4899087	2.62E-11
ENSG00000129474	-1.3428877	2.46E-07
ENSG00000129514	-1.4436509	9.12E-07
ENSG00000129667	-1.2192568	0.00041891

Gene ID	FC 1NG - 4NG	adj p-value 1NG - 4NG
ENSG00000129810	-1.2617028	0.01113976
ENSG00000129911	-1.2428486	0.00011443
ENSG00000130024	-1.3094016	5.65E-05
ENSG00000130066	1.37186439	0.00021024
ENSG00000130201	1.87296869	1.21E-09
ENSG00000130222	-1.5993538	2.58E-06
ENSG00000130270	-1.2425055	0.00880925
ENSG00000130300	1.59128098	0.00061208
ENSG00000130338	1.29288894	3.00E-06
ENSG00000130433	1.29729604	0.03687746
ENSG00000130449	-1.4957509	4.09E-19
ENSG00000130479	-1.4901806	6.38E-13
ENSG00000130513	-1.8823197	3.46E-17
ENSG00000130517	-1.4375535	9.80E-08
ENSG00000130522	-1.2511788	5.53E-05
ENSG00000130590	-1.4014811	0.00026881
ENSG00000130702	-1.4301475	1.79E-08
ENSG00000130751	1.27066409	0.00014413
ENSG00000130940	-1.2653166	0.02020817
ENSG00000130956	1.4469238	1.66E-08
ENSG00000131149	1.83814472	5.65E-12
ENSG00000131153	-1.2347614	0.01340327
ENSG00000131188	-1.2685381	0.01290867
ENSG00000131323	1.23671357	2.85E-09
ENSG00000131370	-1.758376	3.55E-17
ENSG00000131378	1.31428768	7.61E-09
ENSG00000131386	-1.4065969	0.00694227
ENSG00000131409	3.12096825	8.82E-09
ENSG00000131459	1.23575215	0.01807453
ENSG00000131669	-1.3123107	1.02E-06
ENSG00000131724	1.27528132	0.00018237
ENSG00000131725	1.22834287	3.72E-05

Gene ID	FC 1NG - 4NG	adj p-value 1NG - 4NG
ENSG00000131747	-1.2510647	0.0189229
ENSG00000131759	-2.0355636	1.89E-12
ENSG00000131779	1.30657541	4.89E-08
ENSG00000131791	1.37216268	6.84E-06
ENSG00000131831	1.63247445	2.23E-07
ENSG00000131941	-1.2532284	0.03103252
ENSG00000131943	-1.2339906	0.00029664
ENSG00000132003	-1.4105042	2.31E-06
ENSG00000132170	1.28959333	0.00757405
ENSG00000132294	1.29575914	4.74E-05
ENSG00000132330	1.31656539	0.02446331
ENSG00000132334	-1.3750665	9.21E-10
ENSG00000132436	-1.3323535	0.0009577
ENSG00000132470	-1.3800803	0.0003134
ENSG00000132481	-1.3566982	0.0001143
ENSG00000132510	-2.8756627	1.47E-20
ENSG00000132563	-1.3148409	0.00703441
ENSG00000132613	-1.3512044	6.03E-07
ENSG00000132622	-1.3912946	3.73E-05
ENSG00000132661	-1.2010576	0.00887475
ENSG00000132669	-1.2058241	0.00052065
ENSG00000132763	1.27586495	0.00880641
ENSG00000132819	-1.3363916	1.41E-07
ENSG00000132825	1.30937129	0.00015862
ENSG00000132906	-1.2523375	0.00058045
ENSG00000133056	-1.6618811	1.37E-09
ENSG00000133401	-1.3674456	0.00496551
ENSG00000133424	1.24190844	0.03191977
ENSG00000133612	-1.3709457	2.42E-09
ENSG00000133624	1.27616148	0.04315179
ENSG00000133657	2.09036916	2.79E-10
ENSG00000133789	1.22066946	5.55E-06
ENSG00000133794	1.34359492	2.25E-05
ENSG00000133805	-1.4286985	0.00026088
ENSG00000133816	1.23827429	1.18E-06
ENSG00000133818	1.20274918	6.19E-06
ENSG00000134107	-4.0261629	5.04E-27
ENSG00000134138	-1.3618931	1.49E-05
ENSG00000134278	1.24709857	8.23E-09
ENSG00000134291	-1.2792496	9.16E-05
ENSG00000134294	1.51441738	0.00043158
ENSG00000134323	1.38360372	0.00184211
ENSG00000134352	1.34313451	0.00031699
ENSG00000134363	1.84264141	1.39E-10
ENSG00000134508	-1.5620668	5.71E-08
ENSG00000134780	-1.2600923	0.00300264
ENSG00000134802	-1.2588026	3.35E-05
ENSG00000134852	1.20881022	0.01944946
ENSG00000134987	1.21543498	0.00566175
ENSG00000135074	-1.4420537	2.10E-07
ENSG00000135083	-1.2718364	0.00117502
ENSG00000135363	-1.3325563	0.00020162
ENSG00000135365	-1.2882517	4.40E-06
ENSG00000135372	1.2013296	6.55E-06
ENSG00000135482	1.21695079	0.00911358
ENSG00000135604	-1.5901956	2.33E-10
ENSG00000135625	-2.0641038	2.23E-13
ENSG00000135924	1.25082968	0.00015589
ENSG00000135966	-1.9186566	3.13E-17
ENSG00000136044	1.24680604	9.12E-05
ENSG00000136114	-1.5259493	7.76E-10
ENSG00000136153	-1.2329973	8.14E-05
ENSG00000136158	-1.5474993	5.26E-08
ENSG00000136160	-1.3284689	0.02631885
ENSG00000136237	-1.5666993	3.23E-09
ENSG00000136244	2.25096124	1.71E-09
ENSG00000136383	1.33022169	1.23E-05
ENSG00000136404	1.21685004	0.00504751
ENSG00000136444	-1.2003077	0.00017391
ENSG00000136451	-1.2957981	1.13E-12
ENSG00000136560	-1.2719172	0.00026351
ENSG00000136603	-1.5029266	1.31E-06
ENSG00000136630	1.45020307	0.00480977
ENSG00000136717	-1.3241391	5.03E-06
ENSG00000136770	1.33524691	7.65E-09
ENSG00000136826	-3.1205993	4.59E-17
ENSG00000136828	-1.2692684	0.02826639
ENSG00000136859	-1.2308771	5.83E-05
ENSG00000136861	-1.2280946	7.52E-05
ENSG00000136869	1.28921625	2.01E-05
ENSG00000136997	-1.3400426	1.49E-06
ENSG00000137177	1.60019211	1.13E-16
ENSG00000137193	-1.8115981	2.57E-06
ENSG00000137203	1.40208464	0.00003275
ENSG00000137216	-1.2082049	0.01168314
ENSG00000137266	-1.395903	8.26E-06
ENSG00000137310	-1.2969721	0.0030347
ENSG00000137393	1.32580896	3.64E-05
ENSG00000137414	1.21842584	0.01778177
ENSG00000137486	-1.5524437	1.33E-11
ENSG00000137494	1.36471352	6.47E-07
ENSG00000137496	1.25064545	0.00855604
ENSG00000137502	1.52004446	2.77E-06
ENSG00000137522	1.24928039	0.00012416
ENSG00000137692	1.26618424	5.53E-07
ENSG00000137693	1.50949224	3.01E-07

Gene ID	FC 1NG - 4NG	adj p-value 1NG - 4NG
ENSG00000137727	1.88221541	7.55E-11
ENSG00000137760	1.41368069	1.14E-06
ENSG00000137802	-1.3261201	1.13E-07
ENSG00000137804	-1.2742302	0.0015945
ENSG00000137807	-1.292485	0.00049816
ENSG00000137825	-1.2413457	0.01455386
ENSG00000137831	1.54781106	9.23E-08
ENSG00000137872	2.85459828	1.80E-15
ENSG00000137878	-1.4132657	0.00071264
ENSG00000137941	-1.2160198	0.03814745
ENSG00000138018	1.41970453	0.00022807
ENSG00000138111	-1.2098574	0.04911292
ENSG00000138134	-1.4432117	3.41E-05
ENSG00000138135	1.23703865	4.85E-05
ENSG00000138162	-1.4884655	5.12E-07
ENSG00000138166	-2.7331472	2.58E-18
ENSG00000138180	-1.2175392	0.02696342
ENSG00000138347	-1.321242	0.02365683
ENSG00000138386	-1.3027803	7.86E-06
ENSG00000138411	1.4758742	1.03E-10
ENSG00000138434	-1.2045261	1.30E-05
ENSG00000138448	1.38698655	9.20E-06
ENSG00000138449	1.38423301	7.37E-06
ENSG00000138496	-1.2778108	0.00544261
ENSG00000138639	1.32571109	2.11E-10
ENSG00000138650	1.34929926	0.00110877
ENSG00000138675	1.25434273	0.03446527
ENSG00000138678	-1.3044013	0.00018364
ENSG00000138685	1.21330342	0.02554664
ENSG00000138835	-1.8811024	1.31E-11
ENSG00000138944	1.22885986	0.00398202
ENSG00000139154	1.20088231	0.00103394

Gene ID	FC 1NG - 4NG	adj p-value 1NG - 4NG
ENSG00000139163	1.22256937	0.01532027
ENSG00000139263	1.25335212	4.13E-05
ENSG00000139354	-1.4122977	1.75E-05
ENSG00000139874	-1.332216	0.01642039
ENSG00000139926	-1.4611987	2.45E-11
ENSG00000140006	1.26999888	0.00525785
ENSG00000140009	-1.269937	0.00022728
ENSG00000140406	-1.2105368	3.0927E-05
ENSG00000140471	1.23494856	0.01389109
ENSG00000140598	-1.2069744	3.09E-05
ENSG00000140691	-1.4077268	4.70E-07
ENSG00000140853	-1.332767	0.00055941
ENSG00000140873	-1.3912227	4.97E-05
ENSG00000140987	1.24159666	0.00205593
ENSG00000141040	1.35626545	8.66E-05
ENSG00000141068	-1.4526687	4.77E-09
ENSG00000141179	1.22075348	2.97E-05
ENSG00000141258	-1.2159635	0.00162819
ENSG00000141431	1.25082551	0.01638881
ENSG00000141441	1.40496164	0.00014269
ENSG00000141480	-1.2133631	0.02199473
ENSG00000141560	-1.2157279	0.00023421
ENSG00000141582	-1.3492886	0.00044952
ENSG00000141655	-1.4387409	5.86E-06
ENSG00000141664	-1.3383241	1.16E-07
ENSG00000141668	1.92051651	2.95E-05
ENSG00000141858	1.2291293	0.00014056
ENSG00000141934	-1.2219161	0.02367083
ENSG00000142065	1.25436689	0.04361555
ENSG00000142178	-2.20219	4.29E-11
ENSG00000142207	1.2045022	1.46E-05
ENSG00000142396	-1.3477543	0.04322337

Gene ID	FC 1NG - 4NG	adj p-value 1NG - 4NG
ENSG00000142627	-1.8196079	6.28E-17
ENSG00000142661	1.65019555	3.98E-06
ENSG00000142871	-1.2679775	1.37E-05
ENSG00000143067	-1.7213315	3.68E-09
ENSG00000143153	1.48590398	9.00E-10
ENSG00000143228	-1.2685099	0.02186631
ENSG00000143341	1.37826175	0.00330049
ENSG00000143344	-1.5807155	1.03E-12
ENSG00000143363	1.22562927	0.01933443
ENSG00000143367	-1.5825651	9.48E-08
ENSG00000143384	-1.3525656	2.78E-10
ENSG00000143409	-1.3020132	0.00127592
ENSG00000143434	-1.2812381	0.00052911
ENSG00000143469	1.41072113	0.00419367
ENSG00000143476	-1.3177607	0.00557568
ENSG00000143603	-1.2543697	0.03537747
ENSG00000143643	-1.4110572	4.38E-07
ENSG00000143669	1.28648549	0.00628203
ENSG00000143772	-2.4573123	1.89E-19
ENSG00000143801	-1.2652366	0.00140758
ENSG00000143816	-3.0218128	2.37E-12
ENSG00000143995	1.23579259	0.00039572
ENSG00000144063	1.65463697	2.87E-12
ENSG00000144120	1.30116589	0.00050437
ENSG00000144161	1.33497827	0.00032165
ENSG00000144320	1.2540752	0.00082942
ENSG00000144366	1.88125978	1.70E-11
ENSG00000144426	1.23158493	0.0328079
ENSG00000144560	-1.5917272	5.18E-15
ENSG00000144644	1.58457511	8.53E-05
ENSG00000144645	-1.3319364	1.03E-09
ENSG00000144655	-4.4941784	2.12E-27

Gene ID	FC 1NG - 4NG	adj p-value 1NG - 4NG
ENSG00000144677	-1.4385473	2.01E-10
ENSG00000144712	-1.2657671	0.00077077
ENSG00000144749	-1.7755462	3.92E-13
ENSG00000144824	-1.5167571	3.12E-09
ENSG00000145014	-1.4655788	3.56E-10
ENSG00000145555	1.27442636	9.04E-08
ENSG00000145632	1.85128039	2.56E-13
ENSG00000145723	1.3256957	0.00297397
ENSG00000145817	1.53937648	8.80E-09
ENSG00000145860	1.26983718	1.18E-07
ENSG00000145911	-2.9627249	7.17E-18
ENSG00000145916	-1.402556	8.78E-07
ENSG00000146021	-1.6851893	2.25E-09
ENSG00000146072	-1.2090766	4.35E-06
ENSG00000146094	-1.4842487	0.00054005
ENSG00000146232	-1.3721238	9.09E-07
ENSG00000146374	1.92167148	5.09E-07
ENSG00000146414	1.26918894	0.00075491
ENSG00000146587	1.29032816	0.00744932
ENSG00000146678	-1.2242175	0.02940889
ENSG00000146834	-1.2895503	8.00E-07
ENSG00000146859	1.34896802	0.00219641
ENSG00000146918	-1.2293045	0.00185869
ENSG00000146950	-1.4522074	1.06E-09
ENSG00000147119	-1.2879354	0.00010607
ENSG00000147130	-1.2444123	7.3987E-05
ENSG00000147180	1.23953494	0.03345934
ENSG00000147454	-1.3168616	1.39E-05
ENSG00000147650	1.33026487	2.19E-05
ENSG00000147852	-1.3488688	0.00389815
ENSG00000148082	-1.2688027	0.00043671
ENSG00000148339	-1.5527395	4.66E-08

Gene ID	FC 1NG - 4NG	adj p-value 1NG - 4NG
ENSG00000148400	-1.2505538	4.14E-06
ENSG00000148429	-1.2537479	2.60E-07
ENSG00000148468	1.32474803	1.19E-13
ENSG00000148660	-1.2317813	7.51E-06
ENSG00000148773	-1.2728493	0.00378813
ENSG00000148841	-1.7272283	9.17E-18
ENSG00000148848	1.45014695	0.00159782
ENSG00000148926	-1.6693698	3.89E-07
ENSG00000149150	-1.246526	0.0159037
ENSG00000149177	1.34809915	8.55E-08
ENSG00000149289	-1.2845147	0.00024124
ENSG00000149313	1.24806636	0.00636955
ENSG00000149503	-1.2928246	0.00052521
ENSG00000149636	-1.2006823	0.00267027
ENSG00000149646	-1.2938371	0.02145264
ENSG00000149798	-1.3672413	2.88E-10
ENSG00000149948	1.54453797	9.41E-08
ENSG00000150347	1.92014898	1.28E-07
ENSG00000150457	-1.4041805	4.19E-11
ENSG00000150594	-1.2155652	0.01288354
ENSG00000150630	2.02273536	1.02E-15
ENSG00000150764	1.58281002	6.62E-09
ENSG00000150907	-1.4226789	1.12E-11
ENSG00000151012	1.69484899	1.19E-07
ENSG00000151023	1.38897973	0.00498652
ENSG00000151136	2.55766941	5.87E-12
ENSG00000151151	1.33267777	0.03045424
ENSG00000151229	1.49739773	6.42E-05
ENSG00000151247	1.23754121	0.00134636
ENSG00000151414	1.37338502	0.00049642
ENSG00000151474	-1.2168724	3.48E-13
ENSG00000151491	-1.2442754	0.00018104

Gene ID	FC 1NG - 4NG	adj p-value 1NG - 4NG
ENSG00000151689	-1.4048891	2.68E-09
ENSG00000151718	1.49580409	4.14E-17
ENSG00000151725	-1.2929459	0.00124983
ENSG00000151967	-1.7187183	1.52E-07
ENSG00000152133	1.21739015	0.000436
ENSG00000152229	1.39505948	4.07E-05
ENSG00000152284	-1.5509324	2.10E-09
ENSG00000152409	-1.3253682	0.00953205
ENSG00000152465	1.41400413	1.07E-09
ENSG00000152518	-1.275402	4.94E-06
ENSG00000152689	1.90406997	2.40E-11
ENSG00000152782	1.28018868	0.0156312
ENSG00000152804	1.21283867	0.00160585
ENSG00000153048	-1.2317547	0.00083226
ENSG00000153071	1.25349749	5.89E-10
ENSG00000153094	-2.3182789	1.08E-10
ENSG00000153162	1.21371057	1.00E-07
ENSG00000153179	-1.2373415	6.45E-06
ENSG00000153201	1.23215058	0.00923252
ENSG00000153208	-1.9445345	6.21E-13
ENSG00000153234	-2.5928418	0.00010162
ENSG00000153250	1.23827399	8.39E-05
ENSG00000153291	-1.2079862	0.03086044
ENSG00000153391	-1.2162754	0.0011127
ENSG00000153443	-1.3143014	0.00038996
ENSG00000153814	1.70765567	7.11E-11
ENSG00000153815	-1.2287326	2.18E-05
ENSG00000153993	1.29525965	0.00912782
ENSG00000154122	1.42053187	1.05E-08
ENSG00000154127	1.47905398	3.97E-12
ENSG00000154217	1.37769867	0.00023522
ENSG00000154310	1.24471658	2.87E-07

Gene ID	FC 1NG - 4NG	adj p-value 1NG - 4NG
ENSG00000154380	1.46595923	1.24E-15
ENSG00000154639	-1.3752606	0.00715932
ENSG00000154678	1.24027879	0.00960167
ENSG00000154734	2.33043445	2.97E-09
ENSG00000154760	-1.3031298	0.00870812
ENSG00000154864	-1.202561	0.00139648
ENSG00000154930	-1.3382033	0.00116086
ENSG00000154957	1.30759791	7.74E-05
ENSG00000155011	1.70228076	6.28E-07
ENSG00000155090	-3.622659	2.72E-19
ENSG00000155111	-1.2804236	0.00593097
ENSG00000155307	1.37146262	0.00172639
ENSG00000155465	-1.2126078	0.00208521
ENSG00000155744	1.23194295	0.00573816
ENSG00000155760	-1.2219593	0.0017403
ENSG00000155966	1.43209412	8.43E-05
ENSG00000155975	1.27780218	4.78E-06
ENSG00000156030	-1.9106432	9.66E-16
ENSG00000156273	-1.4556125	1.12E-07
ENSG00000156381	-1.2304214	0.00027407
ENSG00000156466	-1.7075811	7.41E-06
ENSG00000156675	-1.5722722	2.38E-05
ENSG00000156804	1.66476641	0.03699649
ENSG00000156876	-1.2697653	0.03576242
ENSG00000156970	-1.3769073	8.18E-05
ENSG00000156983	-1.3487007	2.52E-09
ENSG00000157111	-1.2861684	0.00035878
ENSG00000157168	1.41433345	8.61E-09
ENSG00000157181	1.236616	0.02390602
ENSG00000157184	1.25381297	7.72E-05
ENSG00000157214	1.36223882	0.00054665
ENSG00000157216	1.21863437	2.27E-05

Gene ID	FC 1NG - 4NG	adj p-value 1NG - 4NG
ENSG00000157224	1.33076778	0.00627142
ENSG00000157404	-4.5249074	2.93E-16
ENSG00000157483	1.56335141	1.26E-15
ENSG00000157510	-1.469621	2.12E-13
ENSG00000157514	1.30604669	0.00093584
ENSG00000157557	-1.4956841	1.88E-15
ENSG00000157613	-1.7399243	5.63E-05
ENSG00000157617	-1.6730242	2.21E-14
ENSG00000157827	-1.2138879	5.07E-05
ENSG00000158050	-1.5845712	2.11E-06
ENSG00000158106	-1.5341745	0.00038786
ENSG00000158169	-1.2480374	0.00091655
ENSG00000158186	1.34428559	0.00012257
ENSG00000158195	-1.3621182	3.57E-09
ENSG00000158352	1.43456434	1.52E-11
ENSG00000158402	-1.3526809	0.00021715
ENSG00000158458	-1.4773385	6.72E-06
ENSG00000158555	-1.3454002	5.62E-09
ENSG00000158716	1.39826198	1.29E-05
ENSG00000158769	-1.2172317	3.06E-05
ENSG00000158792	-1.2734232	0.00078683
ENSG00000158805	-1.3142062	5.81E-05
ENSG00000158813	-1.2344453	0.04992951
ENSG00000158859	-1.4259256	8.11E-07
ENSG00000158966	1.50908683	3.05E-09
ENSG00000159082	1.21153538	0.01272094
ENSG00000159167	-1.9378417	3.73E-07
ENSG00000159433	-1.2693666	0.00543467
ENSG00000159640	-1.2728993	0.00684473
ENSG00000159733	-1.8334644	1.06E-07
ENSG00000159788	-1.2534848	1.56E-08
ENSG00000159884	-1.3323025	0.00042361

Gene ID	FC 1NG - 4NG	adj p-value 1NG - 4NG
ENSG00000160007	-1.2742875	5.77E-09
ENSG00000160013	-1.7161863	1.97E-06
ENSG00000160124	1.23710932	0.0089281
ENSG00000160179	-1.8394893	2.68E-09
ENSG00000160193	1.2084404	7.97E-05
ENSG00000160223	-1.2077945	0.01234604
ENSG00000160271	-1.410375	6.49E-06
ENSG00000160336	1.28689565	0.00083616
ENSG00000160447	-1.2558391	1.29E-05
ENSG00000160685	-1.2306147	0.00011311
ENSG00000160796	-1.451308	4.12E-09
ENSG00000160813	-1.3287597	1.25E-05
ENSG00000160888	-3.1782002	4.55E-25
ENSG00000160949	-1.2874208	0.00190866
ENSG00000161298	1.26364564	0.02280745
ENSG00000161542	-1.231604	1.09E-08
ENSG00000161653	-1.2466467	0.0480951
ENSG00000161714	-1.3695043	2.07E-06
ENSG00000161813	1.20535736	0.0022144
ENSG00000161835	-1.4453021	4.07E-07
ENSG00000161940	-2.059874	2.30E-21
ENSG00000162032	-1.2037775	0.00738507
ENSG00000162367	-1.2896794	7.12E-10
ENSG00000162390	1.57071371	6.44E-07
ENSG00000162396	1.31749476	0.0039289
ENSG00000162407	1.85371188	1.15E-08
ENSG00000162413	-1.6250927	3.60E-14
ENSG00000162434	1.27336823	3.37E-09
ENSG00000162437	1.2310402	0.00337309
ENSG00000162496	-1.7913511	2.34E-09
ENSG00000162522	-1.8817749	1.54E-16
ENSG00000162551	1.2122328	0.03888052

Gene ID	FC 1NG - 4NG	adj p-value 1NG - 4NG
ENSG00000162614	1.34365765	5.92E-05
ENSG00000162616	1.40268765	0.00030557
ENSG00000162636	1.43580075	3.53E-05
ENSG00000162692	5.51922953	1.79E-19
ENSG00000162695	1.27331754	0.01583177
ENSG00000162757	1.47661263	1.64E-05
ENSG00000162772	-2.7257303	1.11E-05
ENSG00000162775	-1.2498866	3.85E-06
ENSG00000162783	-1.3808086	1.34E-07
ENSG00000162817	-1.3364774	1.46E-06
ENSG00000162944	-1.3589117	0.02610649
ENSG00000163026	1.31118204	1.03E-05
ENSG00000163050	-1.2944063	0.00988095
ENSG00000163083	-1.4492527	0.00028088
ENSG00000163110	1.5293992	2.58E-17
ENSG00000163162	-1.2274829	2.64E-05
ENSG00000163171	-1.9145034	1.65E-16
ENSG00000163219	-1.8184153	6.15E-05
ENSG00000163235	1.80604614	8.49E-08
ENSG00000163251	-1.4984739	3.09E-05
ENSG00000163291	1.20066336	0.025372
ENSG00000163491	-1.4750633	0.00157808
ENSG00000163545	-2.2179039	4.49E-09
ENSG00000163577	1.29687217	0.01174546
ENSG00000163629	1.24499992	0.02956514
ENSG00000163638	-1.4497785	4.47E-07
ENSG00000163659	-1.8629337	1.28E-20
ENSG00000163660	-1.4129692	1.67E-05
ENSG00000163734	1.22775546	0.01287492
ENSG00000163788	-1.3594099	8.82E-09
ENSG00000163840	-1.2747334	0.00142353
ENSG00000163874	-2.2516724	2.86E-11

Gene ID	FC 1NG - 4NG	adj p-value 1NG - 4NG
ENSG00000163918	-1.2482977	0.01885499
ENSG00000163935	1.55467396	2.08E-05
ENSG00000163947	-1.2314827	0.00202232
ENSG00000164056	2.58807981	2.48E-11
ENSG00000164104	-1.2551202	0.00292428
ENSG00000164125	1.21914437	0.02438332
ENSG00000164161	1.26291283	1.18E-05
ENSG00000164283	1.83535292	9.54E-12
ENSG00000164292	1.40434424	3.92E-08
ENSG00000164294	1.22576049	0.00222911
ENSG00000164306	-1.2914937	0.00024247
ENSG00000164338	1.25737965	2.64E-05
ENSG00000164442	1.50647178	1.7959E-05
ENSG00000164484	1.20503654	0.00246298
ENSG00000164506	1.27557678	2.85E-05
ENSG00000164512	2.44605707	7.04E-12
ENSG00000164649	-1.3271212	7.30E-12
ENSG00000164683	-5.1965278	1.51E-18
ENSG00000164684	2.40328475	3.79E-14
ENSG00000164736	1.32397979	1.03E-06
ENSG00000164741	-1.3171328	1.27E-09
ENSG00000164743	1.52470261	8.66E-06
ENSG00000164823	1.48168726	1.99E-07
ENSG00000164849	-1.8848172	4.31E-05
ENSG00000164867	-1.3377862	4.00E-06
ENSG00000164877	-1.3592734	0.00040682
ENSG00000164897	-1.2209647	0.00053007
ENSG00000164970	-1.214124	2.10E-05
ENSG00000164975	1.21211317	0.00056775
ENSG00000165138	-1.2332756	9.69E-07
ENSG00000165233	-1.2477714	8.55E-05
ENSG00000165244	-1.3914845	0.00029358

Gene ID	FC 1NG - 4NG	adj p-value 1NG - 4NG
ENSG00000165259	1.33797751	0.00089343
ENSG00000165271	1.26766291	1.41E-06
ENSG00000165304	-1.2530466	0.00019231
ENSG00000165322	-1.2282378	2.78E-05
ENSG00000165338	1.35036789	0.00941191
ENSG00000165406	-1.2277037	0.03754112
ENSG00000165424	-1.2464685	0.00269158
ENSG00000165434	1.30035568	0.0009525
ENSG00000165507	2.11527651	3.94E-07
ENSG00000165572	1.26034416	1.48E-05
ENSG00000165655	-1.8592288	4.28E-09
ENSG00000165732	1.25450145	2.1065E-05
ENSG00000165757	-1.3571557	3.24E-14
ENSG00000165801	1.42727115	0.00028201
ENSG00000165806	-1.2834354	2.47E-11
ENSG00000165886	-1.5151172	1.12E-11
ENSG00000165891	1.39568036	6.82E-07
ENSG00000165895	1.31718914	0.00303093
ENSG00000165959	-1.6034257	1.68E-10
ENSG00000166073	1.33967767	1.39E-06
ENSG00000166188	1.20470811	0.00142681
ENSG00000166250	1.32645879	0.00133582
ENSG00000166398	1.20765121	0.00075462
ENSG00000166439	-1.3659916	5.17E-10
ENSG00000166446	1.29199655	6.93E-09
ENSG00000166471	1.23095253	0.00794857
ENSG00000166483	-1.6326544	1.26E-08
ENSG00000166503	1.24580818	0.00016903
ENSG00000166510	1.72696706	7.78E-06
ENSG00000166529	1.21032617	0.02348704
ENSG00000166670	1.33918054	0.00092073
ENSG00000166704	1.35955103	0.00022728

Gene ID	FC 1NG - 4NG	adj p-value 1NG - 4NG
ENSG00000166801	-1.3649643	4.50E-05
ENSG00000166851	-1.215738	0.042226
ENSG00000166886	-2.1832795	2.65E-13
ENSG00000166925	-1.4836893	1.15E-09
ENSG00000166938	-1.2306184	8.93E-05
ENSG00000166949	-1.3132529	2.05E-10
ENSG00000166971	-1.2633285	4.89E-05
ENSG00000167081	1.21095561	0.00020409
ENSG00000167106	-2.0760355	1.09E-15
ENSG00000167173	-1.2251873	0.00023477
ENSG00000167191	-1.6942863	6.18E-08
ENSG00000167202	-1.2479063	2.05E-07
ENSG00000167363	-1.286135	0.00593789
ENSG00000167378	1.24087372	3.04E-08
ENSG00000167470	-2.785101	1.74E-24
ENSG00000167487	-1.2699128	0.04093999
ENSG00000167549	-1.3012513	0.04313048
ENSG00000167554	1.42923852	7.30E-05
ENSG00000167565	-1.6885752	3.91E-12
ENSG00000167600	-1.3083849	0.01453518
ENSG00000167625	1.27715102	4.94E-05
ENSG00000167670	-1.2961527	0.0064365
ENSG00000167680	-1.4564981	1.02E-09
ENSG00000167767	-1.2138026	0.00354598
ENSG00000167772	-2.1936069	1.78E-10
ENSG00000167861	-1.3695557	1.66E-05
ENSG00000167874	-1.4493181	6.75E-05
ENSG00000167972	-1.2388054	9.97E-06
ENSG00000168016	-1.3658385	9.06E-06
ENSG00000168209	-2.8269258	3.37E-13
ENSG00000168297	1.20770362	0.00601656
ENSG00000168298	-1.2040632	0.01573435

Gene ID	FC 1NG - 4NG	adj p-value 1NG - 4NG
ENSG00000168309	-1.3831945	2.67E-06
ENSG00000168386	1.29869625	0.01106119
ENSG00000168461	1.50596055	1.25E-12
ENSG00000168497	-1.7369233	3.62E-15
ENSG00000168575	1.27789864	1.11E-06
ENSG00000168675	1.27959782	0.01348116
ENSG00000168685	1.49806693	0.01137645
ENSG00000168758	-1.4378313	0.00035449
ENSG00000168763	-1.2688774	0.00129682
ENSG00000168769	1.22854764	1.52E-06
ENSG00000168792	-1.2528521	0.00074817
ENSG00000169174	1.23804739	0.00936895
ENSG00000169184	-1.3617402	0.00999459
ENSG00000169242	-2.9462777	5.45E-13
ENSG00000169247	-1.3273568	0.01171364
ENSG00000169252	-1.3632891	0.00607205
ENSG00000169255	-1.3210563	0.00017948
ENSG00000169554	1.31933764	4.36E-10
ENSG00000169594	-1.2849814	3.83E-08
ENSG00000169629	1.30485025	0.00534341
ENSG00000169679	-1.2624091	0.00251352
ENSG00000169683	-1.2293732	0.00142631
ENSG00000169756	1.26512681	0.0036858
ENSG00000169851	1.52997211	1.26E-05
ENSG00000169902	1.21356367	0.00020019
ENSG00000169926	-1.3977646	1.59E-07
ENSG00000169946	1.22340944	2.40E-05
ENSG00000169991	-1.4071598	2.23E-09
ENSG00000170011	-1.4354376	0.0001659
ENSG00000170017	1.21332179	2.49E-05
ENSG00000170145	1.22440865	4.06E-06
ENSG00000170264	-1.2531889	0.00571733

Gene ID	FC 1NG - 4NG	adj p-value 1NG - 4NG
ENSG00000170271	-1.5013742	0.00011981
ENSG00000170293	1.59130221	2.09E-07
ENSG00000170345	-3.0267252	5.39E-10
ENSG00000170365	-1.6461616	1.76E-11
ENSG00000170421	-1.2270018	0.02596204
ENSG00000170425	-1.4133253	8.26E-05
ENSG00000170485	-1.3133295	4.50E-09
ENSG00000170525	-1.6576431	1.47E-17
ENSG00000170558	1.39476512	1.70E-12
ENSG00000170775	1.4594821	8.60E-10
ENSG00000170852	-1.3497269	5.04E-08
ENSG00000170873	-1.254594	0.0004977
ENSG00000170921	1.52676881	2.25E-08
ENSG00000171056	-1.3588839	3.67E-06
ENSG00000171206	-1.3282644	4.20E-10
ENSG00000171223	-6.1371901	2.45E-29
ENSG00000171227	-1.4740834	1.85E-05
ENSG00000171246	1.61353832	8.05E-05
ENSG00000171295	1.28567532	0.00053971
ENSG00000171310	1.46670786	1.21E-06
ENSG00000171316	1.21432286	0.00085148
ENSG00000171388	-1.6330696	2.52E-07
ENSG00000171435	-1.2673175	0.00047054
ENSG00000171488	1.3004846	0.00030334
ENSG00000171522	1.21306619	0.00036992
ENSG00000171552	-1.3749846	2.52E-09
ENSG00000171604	-1.33993	0.01225372
ENSG00000171617	-1.2783196	0.00046649
ENSG00000171621	-2.8160564	5.93E-13
ENSG00000171658	1.52084015	5.36E-05
ENSG00000171729	-1.4003014	3.52E-06
ENSG00000171823	-1.2099842	0.00130987

Gene ID	FC 1NG - 4NG	adj p-value 1NG - 4NG
ENSG00000171827	1.33753766	0.00826301
ENSG00000171940	1.33000135	2.83E-07
ENSG00000171988	-1.2237778	0.00111461
ENSG00000172006	1.32805741	0.00374394
ENSG00000172031	-1.5386237	2.82E-05
ENSG00000172059	-1.3899168	8.73E-05
ENSG00000172086	1.24786511	0.00060114
ENSG00000172159	1.38478341	2.65E-06
ENSG00000172216	-1.2753981	0.00045467
ENSG00000172292	1.20872504	0.01898666
ENSG00000172331	1.27003539	0.00696971
ENSG00000172572	1.20533412	0.00141573
ENSG00000172578	1.33995023	2.89E-09
ENSG00000172731	-1.3572373	0.00020426
ENSG00000172765	-1.2333415	1.43E-06
ENSG00000172795	1.2442876	0.04252767
ENSG00000172819	1.20885699	0.03813053
ENSG00000173011	-1.2603925	1.82E-06
ENSG00000173276	-1.38603	7.03E-07
ENSG00000173281	-1.2061133	0.00020793
ENSG00000173334	-2.0059178	5.52E-10
ENSG00000173530	-1.2499842	1.35E-05
ENSG00000173535	-1.4890606	1.35E-08
ENSG00000173548	-1.2621416	2.01E-06
ENSG00000173597	1.47783499	0.00041858
ENSG00000173846	-1.5014035	7.60E-08
ENSG00000173868	1.24325745	0.00044256
ENSG00000174004	-1.8326303	3.33E-08
ENSG00000174059	-1.8230142	7.37E-05
ENSG00000174136	-1.2962834	2.20E-07
ENSG00000174370	-1.3317711	0.00738507
ENSG00000174705	-1.2664856	1.16E-07

Gene ID	FC 1NG - 4NG	adj p-value 1NG - 4NG
ENSG00000174804	1.23294145	2.35E-08
ENSG00000175040	1.22440366	0.00099053
ENSG00000175155	1.30500272	0.00177214
ENSG00000175264	-2.1944355	1.31E-17
ENSG00000175274	-1.2936723	1.63E-05
ENSG00000175352	1.23223868	0.00442598
ENSG00000175426	1.41854141	0.00013756
ENSG00000175505	-1.316943	0.0055832
ENSG00000175592	-1.8886931	1.65E-12
ENSG00000175595	1.21619983	0.00060357
ENSG00000175746	1.3448619	0.00085208
ENSG00000175772	1.27004798	0.01102723
ENSG00000176055	1.35928791	0.00066235
ENSG00000176102	-1.2093514	0.00318315
ENSG00000176209	-1.2763285	0.00602276
ENSG00000176293	1.20004371	0.02621985
ENSG00000176438	-1.2278383	0.00070504
ENSG00000176692	-1.7522212	5.44E-11
ENSG00000176749	-1.646269	9.66E-08
ENSG00000176842	-1.4100454	0.00018655
ENSG00000176907	-1.9356722	2.48E-07
ENSG00000176974	-1.2291352	1.68E-05
ENSG00000177125	-1.3449562	4.97E-06
ENSG00000177191	-1.392165	0.00011162
ENSG00000177283	-1.4750481	2.37E-08
ENSG00000177303	-1.2580937	6.94E-06
ENSG00000177374	-2.6588839	1.53E-14
ENSG00000177606	-1.7603209	1.91E-18
ENSG00000177663	1.20427389	0.00020346
ENSG00000177679	1.34584657	0.00452356
ENSG00000177683	1.31762486	0.01975379
ENSG00000177706	1.22967817	2.90E-07

Gene ID	FC 1NG - 4NG	adj p-value 1NG - 4NG
ENSG00000177732	-1.3455598	1.97E-06
ENSG00000177951	1.23312929	1.79E-05
ENSG00000178031	1.21279612	6.07E-05
ENSG00000178033	1.21686962	0.00093169
ENSG00000178038	-1.2842664	0.00572202
ENSG00000178104	1.22979824	0.00024288
ENSG00000178105	1.28318496	8.34E-08
ENSG00000178343	1.220839	0.02597613
ENSG00000178397	1.26349263	0.00124218
ENSG00000178409	1.35476568	2.28E-07
ENSG00000178445	-1.2833531	0.00156124
ENSG00000178567	1.22581596	0.04801807
ENSG00000178685	-1.200367	0.02314239
ENSG00000178695	-1.2459762	0.00019883
ENSG00000178700	1.27325133	0.00484032
ENSG00000178718	-1.2782299	1.04E-05
ENSG00000178726	-1.6835688	6.06E-11
ENSG00000178764	1.36797979	5.74E-05
ENSG00000178878	-1.6076364	0.0016407
ENSG00000178882	1.47626882	0.00037462
ENSG00000178971	-1.2453538	0.00060301
ENSG00000178996	-1.24765	5.94E-06
ENSG00000178999	-1.2614487	0.01726511
ENSG00000179044	1.24531307	0.0284054
ENSG00000179314	-1.6829915	3.24E-10
ENSG00000179348	-2.2258864	8.87E-14
ENSG00000179361	-1.4649521	5.29E-06
ENSG00000179388	-13.359393	3.41E-18
ENSG00000179403	1.38654386	0.00692579
ENSG00000179428	1.36098504	0.02417121
ENSG00000179431	-1.2566326	0.00653162
ENSG00000179456	-1.3851883	6.35E-09

Gene ID	FC 1NG - 4NG	adj p-value 1NG - 4NG
ENSG00000179546	-1.6109215	3.22E-06
ENSG00000179630	1.2706203	0.03987478
ENSG00000179862	-1.594575	5.13E-06
ENSG00000179941	1.37827144	0.00252495
ENSG00000179981	-1.3143283	3.21E-07
ENSG00000180035	-1.4278573	3.09E-07
ENSG00000180071	-1.246456	0.03107616
ENSG00000180257	1.33364438	0.00172871
ENSG00000180354	1.24053093	0.04271051
ENSG00000180448	-1.4180623	0.0036092
ENSG00000180479	1.30970433	0.00812409
ENSG00000180530	-1.4575857	1.26E-07
ENSG00000180537	-1.2514309	0.00812583
ENSG00000180592	-1.4356555	0.00026728
ENSG00000180626	1.37700203	0.00055528
ENSG00000180730	-1.2295844	0.0073521
ENSG00000180891	-1.2463592	0.00024864
ENSG00000180938	1.25730618	0.00629749
ENSG00000180998	2.51747401	2.85E-11
ENSG00000181004	1.29040422	0.01098909
ENSG00000181007	1.37471863	0.00022126
ENSG00000181274	1.40563511	1.56E-05
ENSG00000181315	1.21250503	0.011468
ENSG00000181444	-1.2789693	0.01439837
ENSG00000181541	2.26841157	5.57E-13
ENSG00000181634	-1.5991371	0.00282723
ENSG00000181690	1.32315036	0.00117501
ENSG00000181773	-1.3502212	0.00451702
ENSG00000181852	1.21736635	3.04E-05
ENSG00000182022	-2.0332201	1.67E-22
ENSG00000182118	-1.9024271	1.90E-07
ENSG00000182185	-1.2665286	0.03470956
ENSG00000182197	1.61972951	3.48E-22
ENSG00000182253	1.38323675	3.36E-05
ENSG00000182489	1.26729986	0.01593561
ENSG00000182541	-1.3216446	3.42E-07
ENSG00000182568	1.39470787	0.00260664
ENSG00000182704	-1.2975497	5.18E-06
ENSG00000182986	1.21494744	0.00271226
ENSG00000183049	1.79680023	3.26E-08
ENSG00000183072	-1.3119396	0.00060207
ENSG00000183090	1.23380458	0.00570803
ENSG00000183161	1.36025321	0.00030003
ENSG00000183307	-1.3571022	0.00310596
ENSG00000183337	-2.9392312	1.81E-15
ENSG00000183340	1.2977604	1.94E-06
ENSG00000183386	-1.3526177	2.11E-06
ENSG00000183496	-1.5425426	2.01E-06
ENSG00000183578	1.23543421	0.0007285
ENSG00000183580	-1.4175551	1.26E-12
ENSG00000183691	-2.0630394	1.73E-08
ENSG00000183775	1.24701479	0.00068144
ENSG00000183779	-1.6028695	1.72E-07
ENSG00000183808	1.20725472	0.00861324
ENSG00000183856	-1.2535422	0.0032279
ENSG00000183873	-1.2227705	0.00674119
ENSG00000184113	-1.9255159	7.26E-13
ENSG00000184226	1.96964701	2.26E-10
ENSG00000184281	-1.2348576	0.00049412
ENSG00000184304	1.35306464	1.74E-07
ENSG00000184384	1.27988475	5.25E-05
ENSG00000184465	-1.3391174	0.0020925
ENSG00000184557	-3.9358684	4.19E-20
ENSG00000184635	-1.3969751	0.01578277
ENSG00000184792	-1.246078	0.00375737
ENSG00000184939	1.22747794	0.00011906
ENSG00000184988	1.27166029	0.0003758
ENSG00000185022	-2.553947	9.93E-22
ENSG00000185033	-1.241304	0.0088684
ENSG00000185112	-1.7348642	1.93E-14
ENSG00000185262	-1.3744458	2.56E-07
ENSG00000185269	1.53745923	4.56E-08
ENSG00000185338	-1.4663087	0.00062624
ENSG00000185361	-1.7211139	2.99E-12
ENSG00000185483	1.22782041	0.01842127
ENSG00000185614	-1.3546017	0.00577415
ENSG00000185630	-1.2257202	0.04755415
ENSG00000185753	1.20410041	0.00018327
ENSG00000185909	-1.2399505	0.0256556
ENSG00000185920	-1.3528159	0.00389422
ENSG00000185972	1.34080903	0.0187489
ENSG00000186019	1.23947206	0.0088834
ENSG00000186026	1.38144744	0.00169983
ENSG00000186185	-1.2784168	0.01489199
ENSG00000186193	-1.3824795	1.94E-06
ENSG00000186300	1.26578551	0.0108786
ENSG00000186479	-1.621005	3.21E-08
ENSG00000186480	-1.2868903	0.01038518
ENSG00000186591	-1.2852966	1.15E-09
ENSG00000186635	-1.2208568	6.57E-05
ENSG00000186642	-1.282348	0.0023926
ENSG00000186665	-1.3380265	0.00062708
ENSG00000186666	1.25235233	0.01589019
ENSG00000186812	1.41304049	0.00083865
ENSG00000186918	-1.75307	9.43E-10
ENSG00000186994	-1.2372538	0.00878991

Gene ID	FC 1NG - 4NG	adj p-value 1NG - 4NG
ENSG00000187189	1.2274269	2.1452E-05
ENSG00000187266	-1.4391924	5.10E-07
ENSG00000187479	-1.860095	2.43E-07
ENSG00000187498	1.23272882	4.78E-05
ENSG00000187513	-1.468507	0.04586242
ENSG00000187566	1.32871025	0.00027726
ENSG00000187605	-1.2385429	3.94E-05
ENSG00000187607	1.27341267	0.00041888
ENSG00000187609	-1.2213701	0.02236754
ENSG00000187624	1.34654002	0.00088564
ENSG00000187626	1.27705112	0.01065232
ENSG00000187678	-2.5030336	5.99E-20
ENSG00000187741	-1.2707617	0.0072668
ENSG00000187764	-1.9067609	3.91E-11
ENSG00000187800	-1.3960996	1.10E-10
ENSG00000187801	1.29525525	0.00910974
ENSG00000187815	1.32239973	0.00615447
ENSG00000188033	1.25090763	0.01743493
ENSG00000188042	-2.6982941	1.02E-13
ENSG00000188171	1.38569443	0.00226532
ENSG00000188215	-1.3449089	2.16E-07
ENSG00000188277	-2.2059699	1.66E-05
ENSG00000188295	-1.2790692	0.00854433
ENSG00000188312	-1.3409674	0.00055316
ENSG00000188428	1.7435315	9.69E-09
ENSG00000188483	-2.8577868	1.12E-21
ENSG00000188522	-1.6463984	4.13E-10
ENSG00000188549	-1.2662791	0.00187702
ENSG00000188732	-1.5339857	0.00146485
ENSG00000188811	-1.3377047	0.00154761
ENSG00000188868	1.38668938	0.00226864
ENSG00000188997	1.40741522	5.58E-06

Gene ID	FC 1NG - 4NG	adj p-value 1NG - 4NG
ENSG00000189164	1.34988163	0.00082557
ENSG00000189298	1.60160568	2.42E-06
ENSG00000189337	-1.29489	0.04510224
ENSG00000196116	-1.2260804	0.00012601
ENSG00000196169	-1.7576654	3.83E-08
ENSG00000196187	-1.227866	0.00493036
ENSG00000196214	1.24819169	0.00149131
ENSG00000196227	1.52318933	8.24E-06
ENSG00000196263	1.26628506	0.0471558
ENSG00000196268	1.24975713	0.01228947
ENSG00000196352	1.25373169	9.35E-05
ENSG00000196371	-1.2780233	1.51E-06
ENSG00000196411	1.20157146	4.26E-05
ENSG00000196422	-1.2241597	0.01147485
ENSG00000196428	-1.2749851	1.89E-07
ENSG00000196437	1.30466239	0.02669477
ENSG00000196449	-1.3032661	1.66E-07
ENSG00000196456	1.2595345	0.00364263
ENSG00000196458	1.35292306	0.00036905
ENSG00000196468	-1.4411201	0.00029158
ENSG00000196526	1.21561608	2.66E-09
ENSG00000196544	1.29032132	0.00172586
ENSG00000196576	-1.2067636	9.6892E-05
ENSG00000196659	1.33935157	0.00158295
ENSG00000196670	1.35258412	3.56E-06
ENSG00000196730	-1.5658144	9.15E-17
ENSG00000196781	-1.4004767	6.75E-12
ENSG00000196782	-1.6035582	5.76E-15
ENSG00000196792	1.24313852	9.08E-05
ENSG00000196843	-1.7809347	2.88E-10
ENSG00000196876	-1.3383106	0.00017628
ENSG00000196890	-1.2350811	0.03298914

Gene ID	FC 1NG - 4NG	adj p-value 1NG - 4NG
ENSG00000196967	1.28952794	0.03662669
ENSG00000196981	1.3454026	0.00273055
ENSG00000197016	1.29945882	0.01727543
ENSG00000197019	-2.1606588	1.44E-17
ENSG00000197050	1.3196307	0.00137965
ENSG00000197063	-1.4657251	8.02E-12
ENSG00000197128	1.28360587	0.00071891
ENSG00000197182	-1.4249777	0.00347167
ENSG00000197183	-1.4020261	4.25E-12
ENSG00000197256	-1.3492918	8.33E-11
ENSG00000197261	-1.3372795	0.00566448
ENSG00000197329	-1.3207851	0.0001623
ENSG00000197363	1.38964676	0.00062601
ENSG00000197385	-1.2309251	0.03464
ENSG00000197461	-1.3875758	1.56E-05
ENSG00000197536	-1.3997362	0.00061155
ENSG00000197557	1.46617008	8.95E-06
ENSG00000197566	1.40309596	8.62E-05
ENSG00000197619	1.41962262	0.00049342
ENSG00000197622	-1.2201213	3.33E-10
ENSG00000197779	1.21659063	0.01045493
ENSG00000197822	-1.2520308	0.04035146
ENSG00000197841	1.37401024	0.00709562
ENSG00000197852	-1.8700806	1.21E-15
ENSG00000197857	1.24639983	0.00863244
ENSG00000197860	1.29649324	0.00018774
ENSG00000197872	-1.2552477	1.53E-07
ENSG00000197928	1.25613509	0.00237648
ENSG00000197951	1.30062015	0.00041314
ENSG00000198018	1.30798342	3.28E-06
ENSG00000198042	1.20325215	0.00012071
ENSG00000198055	-1.2267502	0.00016678

Gene ID	FC 1NG - 4NG	adj p-value 1NG - 4NG
ENSG00000198060	1.22554232	0.00010723
ENSG00000198081	1.34726751	0.00080811
ENSG00000198108	3.12735144	3.16E-15
ENSG00000198113	-1.2344392	6.59E-05
ENSG00000198142	-1.4755382	1.19E-05
ENSG00000198182	1.46947182	5.63E-06
ENSG00000198205	1.21715075	0.00231567
ENSG00000198324	-1.3850697	6.90E-08
ENSG00000198346	1.21451224	0.03515847
ENSG00000198355	-2.2546266	1.50E-18
ENSG00000198369	-1.3723074	1.97E-07
ENSG00000198416	1.2903313	0.00234439
ENSG00000198435	-4.2952001	1.65E-21
ENSG00000198440	1.35705504	0.00236571
ENSG00000198464	1.20048778	0.03396073
ENSG00000198517	-2.0481393	1.32E-17
ENSG00000198538	1.2872948	0.00030484
ENSG00000198551	1.20203047	0.01370767
ENSG00000198576	-2.9354103	2.12E-09
ENSG00000198604	1.28887017	3.32E-06
ENSG00000198722	-1.2316558	8.59E-09
ENSG00000198774	1.81419761	1.56E-08
ENSG00000198799	1.23309031	0.01559871
ENSG00000198846	1.38822913	0.00154615
ENSG00000198855	1.24271315	3.23E-05
ENSG00000198873	-1.4032493	7.47E-10
ENSG00000198901	-1.2122917	0.00397657
ENSG00000198909	-1.3130889	2.52E-11
ENSG00000198933	-1.2085128	0.0036056
ENSG00000203485	-1.2942091	5.12E-07
ENSG00000203709	1.52997813	0.00105332
ENSG00000203805	1.35732539	0.00173302

Gene ID	FC 1NG - 4NG	adj p-value 1NG - 4NG
ENSG00000203883	-4.1694522	4.58E-18
ENSG00000204103	-1.4376381	0.00015568
ENSG00000204131	-1.2007922	0.00027232
ENSG00000204217	1.86601114	4.97E-13
ENSG00000204406	-1.650039	5.05E-11
ENSG00000204442	1.47956157	4.39E-06
ENSG00000204520	1.21441076	0.01162703
ENSG00000204611	1.49041257	2.66E-10
ENSG00000204634	-1.2002248	0.00116799
ENSG00000204767	1.36859325	6.06E-10
ENSG00000205213	-1.398076	2.97E-05
ENSG00000205269	-1.4958522	0.00223857
ENSG00000205356	-1.3403815	2.14E-08
ENSG00000205476	-1.4412346	2.93E-09
ENSG00000205643	1.24190619	0.00150574
ENSG00000205683	-1.3138346	5.76E-05
ENSG00000206538	2.3445676	9.12E-15
ENSG00000206560	-1.3571388	3.21E-09
ENSG00000207808	-1.305209	0.00065593
ENSG00000207980	-1.9315611	5.94E-08
ENSG00000211455	1.53975345	7.30E-06
ENSG00000213047	1.2550997	0.04975165
ENSG00000213064	1.27052103	0.00040682
ENSG00000213085	1.25584776	0.03855531
ENSG00000213160	-1.6018684	1.26E-05
ENSG00000213190	1.26263482	0.00052248
ENSG00000213347	-1.4383722	1.90E-05
ENSG00000213397	-1.2094002	0.01641357
ENSG00000213626	1.55175124	2.23E-05
ENSG00000213694	-1.4473428	3.18E-11
ENSG00000213799	1.22551184	0.04928469
ENSG00000213903	-1.2764678	0.03858037

Gene ID	FC 1NG - 4NG	adj p-value 1NG - 4NG
ENSG00000214189	1.26986757	0.01218427
ENSG00000214357	-1.5847653	0.00049852
ENSG00000214595	1.26552035	0.02195336
ENSG00000214654	1.28293897	0.013605
ENSG00000214944	-1.7626794	2.42E-14
ENSG00000215012	-1.3569946	7.84E-06
ENSG00000215271	1.25239278	0.00405515
ENSG00000215788	-1.3383487	0.0088766
ENSG00000216775	-1.5387986	2.42E-06
ENSG00000216937	1.26320257	0.04140851
ENSG00000220785	-1.4927425	0.0039233
ENSG00000221829	-1.222699	0.00518753
ENSG00000221866	1.29057705	0.03917409
ENSG00000221886	1.49798984	0.00052742
ENSG00000221926	-1.2336176	4.25E-05
ENSG00000222047	-1.3773922	0.00163794
ENSG00000222455	-1.2147269	0.01956876
ENSG00000223764	1.26057733	4.55E-05
ENSG00000224959	-1.2462818	0.03364471
ENSG00000225206	1.30694034	0.0139523
ENSG00000225614	-1.3580655	7.67E-08
ENSG00000225830	1.59058198	1.41E-11
ENSG00000225968	2.97412761	1.15E-13
ENSG00000226348	-1.212155	0.00468774
ENSG00000226562	1.27308171	0.01430701
ENSG00000226808	1.28504603	0.00280068
ENSG00000226823	1.231941	0.03159728
ENSG00000227124	1.2592653	0.02243586
ENSG00000227517	1.43737797	0.00058096
ENSG00000229644	-1.2109204	0.02351517
ENSG00000229729	-1.362784	0.00074852
ENSG00000229953	-1.5792012	2.70E-06

Gene ID	FC 1NG - 4NG	adj p-value 1NG - 4NG
ENSG00000230183	-1.2253671	0.00298409
ENSG00000230266	-1.3490535	0.00158015
ENSG00000230438	-1.4817256	0.00012968
ENSG00000230479	-1.2752117	1.61E-06
ENSG00000230487	-1.2678549	0.01800387
ENSG00000230753	-1.3280451	0.00025719
ENSG00000232098	1.2735333	0.00478402
ENSG00000233237	1.54595541	0.00052021
ENSG00000233370	1.31903181	0.00012024
ENSG00000234284	1.243558	0.02987598
ENSG00000235831	-1.4079949	8.19E-06
ENSG00000236859	1.23159823	0.03248098
ENSG00000237440	1.35894202	0.00043688
ENSG00000237989	-1.2438802	0.00084517
ENSG00000239306	1.26861439	1.80E-08
ENSG00000239521	-1.3002928	0.04148239
ENSG00000239887	-1.5808813	5.91E-08
ENSG00000240225	1.28357385	5.74E-05
ENSG00000240694	-1.5628095	2.21E-12
ENSG00000241015	1.22802985	0.00889012
ENSG00000241839	-1.2624245	2.41E-05
ENSG00000241852	-1.4300766	0.00003502
ENSG00000241978	-1.2946653	0.00084573
ENSG00000242829	-1.3775015	5.71E-05
ENSG00000243547	-1.2186145	0.02419592
ENSG00000244694	-1.241754	0.04559601
ENSG00000245060	1.26002615	0.00894903
ENSG00000245680	1.24475165	0.00031188
ENSG00000247679	-1.2913539	0.03159645
ENSG00000248008	1.30721695	0.00316817
ENSG00000248866	1.25968294	0.01069523
ENSG00000249087	1.24690141	0.01242968

Gene ID	FC 1NG - 4NG	adj p-value 1NG - 4NG
ENSG00000249279	-1.5958101	1.15E-06
ENSG00000249459	1.28154704	0.03766779
ENSG00000249471	1.34049061	0.00103851
ENSG00000250303	1.4858119	1.58E-06
ENSG00000250510	-1.5161041	8.08E-07
ENSG00000250899	-1.2641081	0.00015266
ENSG00000253276	1.34078538	1.18E-06
ENSG00000253368	1.31975523	9.20E-07
ENSG00000253669	-1.509949	7.75E-07
ENSG00000253738	1.22751647	0.02939529
ENSG00000253958	-1.3170072	0.00186856
ENSG00000254004	1.22378375	0.01500892
ENSG00000254087	1.30447163	9.24E-08
ENSG00000254470	-1.269322	4.80E-07
ENSG00000254858	1.24617753	0.00065276
ENSG00000255112	-1.2048443	8.74E-05
ENSG00000255176	-1.8896868	2.5152E-05
ENSG00000256229	1.29805982	0.01138115
ENSG00000256294	1.21053974	0.02379289
ENSG00000256448	-1.2167791	0.00024074
ENSG00000256628	1.2093251	0.02912211
ENSG00000257135	1.23973791	0.01680321
ENSG00000257704	-1.2316795	0.02303593
ENSG00000257923	1.27113687	7.82E-07
ENSG00000258366	-1.2348263	0.04003102
ENSG00000258405	1.22711084	0.03438572
ENSG00000259330	-1.3497032	0.00021845
ENSG00000259642	1.37104465	0.00106316
ENSG00000259726	1.22904702	0.04259792
ENSG00000259768	-1.2830795	0.03447482
ENSG00000259863	-1.3804835	0.00046906
ENSG00000259877	1.29609379	0.00834878

Gene ID	FC 1NG - 4NG	adj p-value 1NG - 4NG
ENSG00000260428	-1.2054316	0.04793275
ENSG00000260992	-1.2448869	0.00241872
ENSG00000260996	-1.3348238	0.00675663
ENSG00000261253	-1.5961403	2.40E-07
ENSG00000261373	1.41292118	2.39E-07
ENSG00000263002	1.29625664	0.0005299
ENSG00000263155	-1.5500114	1.83E-05
ENSG00000263528	1.21038869	0.00104973
ENSG00000264247	1.22989089	0.02449761
ENSG00000264395	-1.2264206	0.00051882
ENSG00000264635	-1.2564041	0.00595886
ENSG00000265185	-1.3773804	0.01223686
ENSG00000265666	-1.3197097	0.0091733
ENSG00000265972	1.96149114	4.22E-13
ENSG00000265982	-1.2303622	0.03041202
ENSG00000266028	-1.2130008	1.02E-09
ENSG00000266533	-1.260386	0.001047
ENSG00000267041	-1.5904091	3.52E-06
ENSG00000267045	1.20752119	0.02285566
ENSG00000267365	1.42002852	0.02647355
ENSG00000267481	1.23148254	0.03403127
ENSG00000267493	1.20136118	0.00327909
ENSG00000267519	-2.7667813	4.48E-10
ENSG00000267583	-1.4629161	2.58E-09
ENSG00000267598	-2.4636354	6.88E-08
ENSG00000267745	1.21716472	0.03429475
ENSG00000269890	1.27640898	0.03309265
ENSG00000270177	1.20184215	0.04895706
ENSG00000270362	1.2656493	0.03130456
ENSG00000271270	1.25724003	0.0196684
ENSG00000271936	-1.2177946	0.03974917
ENSG00000272405	-1.682754	6.08E-05

Gene ID	FC 1NG - 4NG	adj p-value 1NG - 4NG
ENSG00000272476	1.26936626	0.036154
ENSG00000272574	-1.348235	0.00011383
ENSG00000272661	1.22443515	0.00712796
ENSG00000272941	1.26291225	0.01192581
ENSG00000273489	-1.5631307	0.00036924
ENSG00000273729	-1.3403247	0.00092219
ENSG00000273820	-1.383422	0.00086921
ENSG00000274265	1.2095923	0.00185221
ENSG00000274349	1.45901798	0.00011271
ENSG00000274602	-1.2303854	0.02844203
ENSG00000274636	-1.6390335	0.00011394
ENSG00000274853	1.23023012	0.00956565
ENSG00000275031	-1.3356059	0.00250069
ENSG00000275074	1.29364753	0.0142575
ENSG00000275111	1.26445219	0.01038691
ENSG00000275202	-1.3333058	0.00582763
ENSG00000275342	-1.4059974	6.34E-06
ENSG00000275793	-1.2990854	3.6363E-05
ENSG00000275993	-4.7411689	2.51E-21
ENSG00000276043	-1.2617483	0.00032666
ENSG00000276340	1.30450922	0.00018716
ENSG00000276386	-1.265747	6.33E-05
ENSG00000276600	1.24684721	0.02297748
ENSG00000276644	1.58983334	8.47E-06
ENSG00000276836	-1.3509199	0.00693521
ENSG00000276849	-1.3815415	0.02916522
ENSG00000278546	-1.2659539	0.01297836
ENSG00000278921	-1.2722204	0.01395626
ENSG00000278970	1.31892751	5.29E-06
ENSG00000279059	1.33622083	0.00610586
ENSG00000279069	1.40134048	0.00245723
ENSG00000279095	1.26695878	1.49E-05

Gene ID	FC 1NG - 4NG	adj p-value 1NG - 4NG
ENSG00000279117	-1.6265821	1.80E-13
ENSG00000279289	1.67425102	3.74E-05
ENSG00000279365	-1.2567908	0.00072287
ENSG00000279496	-1.3235141	0.00317829
ENSG00000279637	-1.248977	3.08E-06
ENSG00000279822	-1.2132524	0.03259108
ENSG00000279861	-1.2122334	0.01222231
ENSG00000280138	-1.4235385	0.01176777
ENSG00000280187	1.42507539	0.00051304
ENSG00000280303	1.26075915	0.03525917
ENSG00000281614	-1.3326874	0.00071268
ENSG00000281881	-2.3970752	1.12E-08
ENSG00000282386	1.20327193	0.04115537

Supplementary Table 2c. Differentially expressed genes between 4hr and 8hr normal glucose samples

Ensembl gene IDs, Fold change (4NG-8NG) and FDR adjusted p-values of DEGs

Gene ID	FC 4NG-8NG	adj p-value 4NG - 8NG
ENSG00000002587	-1.458479	4.85E-06
ENSG00000004660	1.37466523	0.00041413
ENSG00000006459	-1.6506578	1.28E-08
ENSG00000006468	1.25924447	0.00715181
ENSG00000007908	-7.7890543	3.29E-13
ENSG00000007944	1.35390212	3.27E-05
ENSG00000007968	2.22378338	9.23E-10
ENSG00000008083	1.24259601	0.00445984
ENSG00000008405	-1.3712411	7.67E-07
ENSG00000010818	-1.6107096	1.70E-06
ENSG00000011638	1.2318673	0.00023814
ENSG00000012048	1.26468615	0.00758245
ENSG00000013016	1.23828765	0.03579959
ENSG00000013293	1.63106781	5.74E-07
ENSG00000013588	1.44872353	8.77E-07
ENSG00000015133	1.94372578	2.30E-13
ENSG00000015532	1.22654675	9.38E-06
ENSG00000015568	-1.6915053	9.90E-07
ENSG00000018869	-1.249649	0.02438412
ENSG00000019102	1.31313718	0.03564811
ENSG00000019144	-1.3625205	9.34E-09
ENSG00000020256	1.21485888	6.19E-05
ENSG00000020577	-1.2047772	1.65E-07
ENSG00000021645	1.60663873	3.32E-07
ENSG00000021762	1.29189971	3.38E-06
ENSG00000022567	1.31866256	0.00802956
ENSG00000023445	-2.3923607	4.13E-07
ENSG00000023902	-1.3073244	1.58E-05
ENSG00000027869	-1.2842325	0.04195162
ENSG00000028137	1.41158408	4.28E-06
ENSG00000028277	1.34997378	0.00123303
ENSG00000029993	-1.2074323	0.00011606

Gene ID	FC 4NG-8NG	adj p-value 4NG - 8NG
ENSG00000031081	-1.344087	3.65E-12
ENSG00000034152	-1.3045544	4.94E-05
ENSG00000042286	1.23450768	0.00552375
ENSG00000043143	2.15804167	3.30E-15
ENSG00000046889	1.2375204	0.00048177
ENSG00000047617	1.49519189	1.90E-06
ENSG00000048405	1.24117295	0.0100981
ENSG00000049130	-2.1709687	1.17E-06
ENSG00000049283	1.76384085	6.15E-05
ENSG00000050820	1.26780207	3.32E-07
ENSG00000053254	1.24665328	6.96E-06
ENSG00000056558	-3.2869733	3.96E-09
ENSG00000057294	1.83946555	2.46E-07
ENSG00000057704	1.49334602	3.16E-10
ENSG00000063438	1.26492161	0.00120853
ENSG00000064042	-1.3032556	0.00833804
ENSG00000064225	-1.2600602	0.02752924
ENSG00000064999	1.31098961	3.48E-05
ENSG00000065308	1.29400413	6.20E-08
ENSG00000065328	1.43619505	7.9897E-05
ENSG00000065559	-1.2295472	0.00032145
ENSG00000065809	1.32687892	9.38E-11
ENSG00000066735	1.84542735	1.02E-12
ENSG00000066933	1.21985692	0.0007222
ENSG00000067064	-1.4599263	0.00026483
ENSG00000067177	1.21143542	0.00323218
ENSG00000067715	1.26894803	0.0223388
ENSG00000067798	-1.6536891	1.06E-16
ENSG00000067955	-1.8162473	1.29E-10
ENSG00000068024	1.30663393	4.32E-07
ENSG00000068078	-1.3410526	0.00024389
ENSG00000068137	1.42869338	0.00062611

Gene ID	FC 4NG-8NG	adj p-value 4NG - 8NG
ENSG00000068305	-1.3174982	2.35E-05
ENSG00000069702	1.50094305	2.21E-06
ENSG00000070669	-1.2272195	0.00600691
ENSG00000071073	1.32668902	3.27E-06
ENSG00000072422	-1.4249877	2.19E-10
ENSG00000073111	1.24259288	0.00298813
ENSG00000073417	-1.4350922	4.17E-12
ENSG00000073712	-1.2316039	4.13E-07
ENSG00000073756	-4.2689894	3.06E-09
ENSG00000074047	-2.6286081	8.89E-15
ENSG00000074410	1.26485291	0.01660351
ENSG00000074855	1.39446872	7.15E-05
ENSG00000074935	-1.209992	0.03525913
ENSG00000075651	1.52310905	1.05E-10
ENSG00000076003	1.23819798	2.2302E-05
ENSG00000076248	1.37592718	6.86E-10
ENSG00000076351	1.60053776	2.16E-08
ENSG00000076382	-1.2377463	0.00130076
ENSG00000077044	-1.2429027	0.0005918
ENSG00000077150	-1.3685163	3.77E-06
ENSG00000077514	1.33042336	1.34E-06
ENSG00000078018	1.64069841	1.53E-06
ENSG00000078401	1.27638688	7.8204E-05
ENSG00000078687	1.28497135	1.13E-06
ENSG00000078747	-1.2435781	7.84E-06
ENSG00000079102	-1.4863789	2.76E-05
ENSG00000079308	-1.3281392	8.38E-07
ENSG00000079335	-1.4124341	4.25E-07
ENSG00000080298	1.296587	0.00059496
ENSG00000080608	-1.3109435	3.12E-08
ENSG00000080709	-1.2187994	0.00107356
ENSG00000080839	1.2384117	0.02781114

Gene ID	FC 4NG-8NG	adj p-value 4NG - 8NG
ENSG00000081059	-1.3476433	0.00332197
ENSG00000081177	1.23868442	6.03E-07
ENSG00000081189	-1.2658446	4.54E-05
ENSG00000081803	1.27454082	7.87E-10
ENSG00000081923	1.28866027	0.00713161
ENSG00000082512	1.26413164	0.02343967
ENSG00000084093	-1.5941232	2.88E-10
ENSG00000084710	-1.4533364	0.00037539
ENSG00000085117	-1.2058775	0.00336979
ENSG00000085185	1.67728018	5.00E-09
ENSG00000085840	1.50451259	6.98E-05
ENSG00000087266	1.31537914	4.82E-06
ENSG00000087494	-1.5540186	0.00020217
ENSG00000087903	-1.330075	0.03137803
ENSG00000088387	1.41374886	2.68E-12
ENSG00000089041	1.74676574	4.11E-06
ENSG00000089685	-1.2115956	0.00221115
ENSG00000090376	1.35726896	7.96E-05
ENSG00000090447	1.33664304	0.00039595
ENSG00000090889	-1.2009468	0.02760304
ENSG00000090975	-1.2730996	1.17E-05
ENSG00000091157	1.20393752	6.69E-06
ENSG00000091428	-1.3855646	5.21E-05
ENSG00000091622	1.3686985	0.03188295
ENSG00000091879	1.25007348	7.02E-05
ENSG00000092470	1.55051305	1.06E-07
ENSG00000092621	-1.2535467	0.00221347
ENSG00000094804	1.51052973	5.70E-07
ENSG00000095370	1.42296439	1.89E-08
ENSG00000095383	1.7416767	1.94E-09
ENSG00000095397	-1.2023691	0.03349348
ENSG00000095752	-2.1932659	2.85E-09

Gene ID	FC 4NG-8NG	adj p-value 4NG - 8NG
ENSG00000099260	1.20787496	1.48E-05
ENSG00000099337	1.20189971	0.00292218
ENSG00000099860	1.23761786	0.0485878
ENSG00000099875	1.47723799	2.06E-10
ENSG00000099910	1.35794916	6.52E-05
ENSG00000100036	1.32039203	2.54E-05
ENSG00000100065	1.35991378	1.07E-06
ENSG00000100276	-1.2138214	0.04094199
ENSG00000100292	-1.2199228	0.00824921
ENSG00000100307	1.32234421	0.01584465
ENSG00000100344	-1.3636616	0.00147141
ENSG00000100417	1.23374147	0.02268569
ENSG00000100490	1.44083959	1.9988E-05
ENSG00000100526	-1.2876295	0.00202532
ENSG00000100592	-1.411676	3.79E-11
ENSG00000100890	1.21835228	0.02144211
ENSG00000101019	1.21853266	8.81E-06
ENSG00000101040	1.24906837	5.86E-07
ENSG00000101096	-1.2060995	0.00676984
ENSG00000101187	1.4243817	0.00022897
ENSG00000101265	1.20151566	0.00872634
ENSG00000101298	1.25674569	2.30E-05
ENSG00000101331	1.23335312	0.00282171
ENSG00000101384	-1.5679877	6.16E-08
ENSG00000101412	1.47624116	5.08E-05
ENSG00000101445	1.48232914	3.03E-07
ENSG00000101447	-1.2176976	0.01992631
ENSG00000101577	1.23297053	1.16E-06
ENSG00000101665	-1.7811025	0.0001027
ENSG00000101670	-1.7821762	6.66E-13
ENSG00000102096	1.3174964	0.00326242
ENSG00000102098	1.22638785	0.02359914

Gene ID	FC 4NG-8NG	adj p-value 4NG - 8NG
ENSG00000102178	1.26244864	9.68E-06
ENSG00000102221	-1.2156766	5.66E-05
ENSG00000102531	-1.3166825	5.09E-10
ENSG00000102554	-1.217049	0.01017485
ENSG00000102755	-1.3892332	3.45E-08
ENSG00000102935	-1.3962862	0.00580701
ENSG00000102984	-1.3365297	0.01711469
ENSG00000103034	1.20686659	0.01310962
ENSG00000103257	1.21106066	0.00052207
ENSG00000103319	1.24098195	6.35E-09
ENSG00000103522	-1.2386607	2.14E-05
ENSG00000103647	-1.2186421	0.00075202
ENSG00000103888	-1.2007906	0.01147178
ENSG00000104093	1.27407715	0.00386958
ENSG00000104154	1.26565285	0.01774596
ENSG00000104312	-1.2230074	0.00158274
ENSG00000104419	-1.3475977	2.00E-08
ENSG00000104447	-1.2085118	0.02859654
ENSG00000104450	-1.3038669	0.00224713
ENSG00000104549	-1.3134141	2.17E-06
ENSG00000104738	1.22896195	0.00133557
ENSG00000104856	-1.7656265	1.16E-08
ENSG00000104863	-1.2186003	0.02529802
ENSG00000104967	1.24505059	0.00071294
ENSG00000105173	1.20400163	0.04395097
ENSG00000105287	1.24616029	2.78E-05
ENSG00000105538	1.20927468	4.16E-05
ENSG00000105662	1.38098753	1.85E-05
ENSG00000105810	-1.2255679	0.01181376
ENSG00000105855	-1.2520955	0.01639529
ENSG00000105875	-1.270424	0.00053602
ENSG00000105939	1.44152518	4.72E-13

Gene ID	FC 4NG-8NG	adj p-value 4NG - 8NG
ENSG00000106070	-2.0009933	3.84E-19
ENSG00000106089	-1.2518696	0.03850592
ENSG00000106236	-1.2848386	0.04015577
ENSG00000106351	1.58198821	4.37E-09
ENSG00000106479	1.33468279	0.00244976
ENSG00000106546	-1.2165322	5.37E-05
ENSG00000106571	1.46318806	1.19E-10
ENSG00000106688	1.2772739	3.63E-08
ENSG00000106789	1.37276612	0.00117501
ENSG00000107099	-1.320012	0.00347483
ENSG00000107104	-1.4997218	2.71E-15
ENSG00000107185	1.23276837	2.05E-05
ENSG00000107485	1.30523744	0.00012343
ENSG00000107551	1.27467006	0.04448832
ENSG00000107731	-4.2907545	1.17E-18
ENSG00000107821	-1.5020735	0.00230231
ENSG00000107863	-1.4691567	5.78E-15
ENSG00000107984	-1.3203466	0.00073381
ENSG00000108106	-1.2270825	0.00106665
ENSG00000108262	1.24691294	1.58E-06
ENSG00000108312	-1.6884399	1.82E-16
ENSG00000108389	1.21430962	1.85E-08
ENSG00000108433	-1.2219207	0.00026236
ENSG00000108691	-1.9379172	1.79E-07
ENSG00000108960	-1.6016116	1.81E-06
ENSG00000109265	-1.2492177	0.00805102
ENSG00000109320	-1.4487162	3.41E-17
ENSG00000109321	-1.2758448	0.00017143
ENSG00000109466	-1.227644	1.71E-05
ENSG00000109756	-1.2447589	1.3633E-05
ENSG00000109906	1.87334084	2.19E-08
ENSG00000109929	-1.3416378	0.01956924

Gene ID	FC 4NG-8NG	adj p-value 4NG - 8NG
ENSG00000109944	1.32589175	9.4169E-05
ENSG00000110330	-1.3596806	0.00020921
ENSG00000110675	1.24837507	0.03649497
ENSG00000110876	1.2147107	0.00154351
ENSG00000111077	1.40304216	1.55E-08
ENSG00000111490	-1.258149	0.00185275
ENSG00000111665	-1.2901728	0.01774757
ENSG00000111671	-1.2394953	0.03513322
ENSG00000111725	1.35446282	1.08E-05
ENSG00000111846	1.35781266	4.22E-07
ENSG00000111859	-1.4030541	0.00058871
ENSG00000111860	-1.2402927	0.00657837
ENSG00000111907	-1.2652778	0.01666627
ENSG00000111912	-1.2559282	1.28E-05
ENSG00000111961	1.54357689	2.84E-20
ENSG00000112033	-1.2380634	1.21E-05
ENSG00000112039	1.42774778	1.94E-07
ENSG00000112183	-1.4208714	0.00512809
ENSG00000112208	-1.23532	0.0227467
ENSG00000112210	-1.4653419	2.59E-08
ENSG00000112541	-1.5701925	1.35E-05
ENSG00000112559	-1.3451181	0.00018461
ENSG00000112561	1.25038248	0.00143154
ENSG00000112658	-1.2074245	1.95E-07
ENSG00000112773	-1.4656923	0.02601596
ENSG00000112972	-1.723436	7.68E-07
ENSG00000112984	-1.2775035	0.00018263
ENSG00000113161	-1.5226201	7.29E-07
ENSG00000113369	-1.2290829	0.0137624
ENSG00000113522	1.2618526	1.35E-06
ENSG00000113532	-1.545472	0.00014677
ENSG00000113580	-1.6321214	1.44E-10

Gene ID	FC 4NG-8NG	adj p-value 4NG - 8NG
ENSG00000113739	1.22128624	0.01707849
ENSG00000114315	1.60255716	0.00379046
ENSG00000114423	-1.2900863	0.00042712
ENSG00000114529	-1.4487047	6.72E-05
ENSG00000114698	1.36758933	4.3475E-05
ENSG00000114796	-1.2356638	0.01592893
ENSG00000114841	1.26108295	0.04292192
ENSG00000115163	-1.2695794	0.00819437
ENSG00000115170	-1.2963838	8.67E-10
ENSG00000115183	1.20493571	1.77E-05
ENSG00000115525	-1.3864739	0.00200248
ENSG00000115548	-1.3295872	1.47E-07
ENSG00000115738	-1.4357693	0.03077349
ENSG00000115750	1.27747006	0.00218839
ENSG00000115760	1.22437286	0.00621671
ENSG00000115896	-1.3554636	0.01397262
ENSG00000115946	-1.2443332	1.60E-05
ENSG00000116062	1.38858543	5.28E-16
ENSG00000116106	1.20070085	0.00035209
ENSG00000116128	-1.2815091	2.40E-05
ENSG00000116273	1.24400904	0.00016737
ENSG00000116584	1.34225635	9.24E-10
ENSG00000116679	-1.2049415	0.00035827
ENSG00000116741	-1.216648	0.04773476
ENSG00000116786	-1.2037058	6.6939E-05
ENSG00000117143	-1.2912147	3.09E-07
ENSG00000117228	-1.258398	3.32E-05
ENSG00000117318	-1.2655897	0.00047054
ENSG00000117595	-1.6253407	0.00046244
ENSG00000117602	1.22696315	0.00359916
ENSG00000117650	-1.274908	0.00346104
ENSG00000118058	1.25198924	0.00116989

Gene ID	FC 4NG-8NG	adj p-value 4NG - 8NG
ENSG00000118257	1.24660556	5.42E-05
ENSG00000118402	-1.3225343	0.01180143
ENSG00000118503	-1.7715931	4.46E-07
ENSG00000118655	1.23055612	0.00755694
ENSG00000118689	1.20059846	0.00099278
ENSG00000118922	1.36012678	0.0001215
ENSG00000118985	-1.368139	2.60E-08
ENSG00000119139	-1.2508112	1.32E-06
ENSG00000119771	-1.5363095	1.51E-13
ENSG00000119862	1.23800485	0.01231703
ENSG00000119906	1.23925424	3.61E-05
ENSG00000119965	-1.2184824	0.01556121
ENSG00000119969	1.32085428	0.02835309
ENSG00000120278	1.86038948	9.63E-17
ENSG00000120279	-1.2401638	0.00072485
ENSG00000120693	-2.4431159	1.03E-13
ENSG00000120738	-1.7699139	0.00703856
ENSG00000120875	1.73748259	2.69E-17
ENSG00000120899	1.45249979	7.80E-06
ENSG00000121057	1.20968514	0.0020045
ENSG00000121060	1.28375718	7.22E-09
ENSG00000121390	-1.2444142	2.38E-08
ENSG00000121931	-1.2064755	0.0048237
ENSG00000121966	1.77200311	4.29E-11
ENSG00000122068	-1.2497866	3.02E-06
ENSG00000122257	1.35518473	6.78E-11
ENSG00000122376	1.21668706	0.03314064
ENSG00000122641	-1.2374382	0.02522337
ENSG00000122863	-1.3674392	3.70E-05
ENSG00000122870	-1.2927067	0.00418354
ENSG00000122966	-1.2039839	0.00970337
ENSG00000123066	-1.2200108	1.51E-06

Gene ID	FC 4NG-8NG	adj p-value 4NG - 8NG
ENSG00000123094	-1.2402533	0.00086262
ENSG00000123146	1.23222098	0.01168902
ENSG00000123358	-1.6241169	0.00312429
ENSG00000123700	-1.4894752	0.00981923
ENSG00000123933	1.24269219	0.00043926
ENSG00000123975	-1.2477898	0.00403748
ENSG00000124225	-1.9543621	1.28E-09
ENSG00000124313	1.20758873	0.03982318
ENSG00000124766	-1.2252249	0.00014916
ENSG00000125089	1.25713783	0.00031771
ENSG00000125629	1.31734547	0.01178116
ENSG00000125845	-1.7857164	1.88E-09
ENSG00000125864	1.24022098	0.02212575
ENSG00000125898	-1.4591647	8.95E-06
ENSG00000125962	-1.2024427	0.01962243
ENSG00000125968	-1.5113021	0.00094976
ENSG00000126562	1.2073395	0.01737956
ENSG00000126705	1.46390833	1.99E-06
ENSG00000126882	1.56042501	6.37E-05
ENSG00000127080	-1.3552439	1.24E-07
ENSG00000127124	-1.6168167	1.01E-07
ENSG00000127533	1.25948264	0.02754701
ENSG00000128394	1.22089671	0.04774696
ENSG00000128487	-1.2189135	0.0481651
ENSG00000128594	-5.3467321	1.02E-18
ENSG00000128641	-1.2921124	0.00027236
ENSG00000128805	1.38145908	5.63E-10
ENSG00000128833	-1.4079615	0.00010226
ENSG00000128881	1.25471886	0.00127822
ENSG00000128917	2.2389035	1.53E-10
ENSG00000128923	1.40300941	8.67E-07
ENSG00000128944	-1.2429518	0.00046694

Gene ID	FC 4NG-8NG	adj p-value 4NG - 8NG
ENSG00000129116	-1.2524581	0.00437081
ENSG00000129173	1.48032182	2.68E-06
ENSG00000129195	-1.2722697	0.00316295
ENSG00000129355	1.2928559	0.01017409
ENSG00000129422	1.44073631	2.87E-10
ENSG00000129474	1.62458375	5.83E-13
ENSG00000130021	1.27613193	9.88E-06
ENSG00000130066	-1.4519281	1.37E-05
ENSG00000130164	-1.5005682	1.10E-08
ENSG00000130201	-1.7535216	1.85E-08
ENSG00000130338	-1.361544	5.94E-08
ENSG00000130363	1.23465944	0.01200216
ENSG00000130449	1.26693601	7.58E-12
ENSG00000130517	1.42799159	1.49E-07
ENSG00000130544	-1.2279156	0.00935596
ENSG00000130702	1.3675076	3.75E-07
ENSG00000130956	-1.4140622	7.49E-08
ENSG00000131037	-1.2756742	0.00319463
ENSG00000131149	-1.7557474	5.19E-11
ENSG00000131188	1.22653858	0.04247942
ENSG00000131378	-1.2539569	5.66E-07
ENSG00000131386	1.62919096	6.40E-05
ENSG00000131409	-1.6708927	0.00726424
ENSG00000131634	-1.2238802	0.00251685
ENSG00000131759	1.30847008	0.00156224
ENSG00000131831	-1.554494	2.04E-06
ENSG00000131845	-1.3479944	7.82E-05
ENSG00000131943	1.32442808	1.98E-06
ENSG00000132003	-1.242177	0.00297453
ENSG00000132170	-1.2620298	0.01707147
ENSG00000132196	-1.2697952	0.00018866
ENSG00000132205	1.57409917	6.93E-07

Gene ID	FC 4NG-8NG	adj p-value 4NG - 8NG
ENSG00000132436	1.27712384	0.00607893
ENSG00000132510	-1.284263	0.00033823
ENSG00000132613	1.2930733	1.35E-05
ENSG00000132622	1.22326721	0.01792073
ENSG00000132623	1.24535129	0.02587708
ENSG00000132881	1.28399096	0.00761945
ENSG00000132906	1.22388498	0.00227303
ENSG00000133056	1.38797029	1.90E-05
ENSG00000133101	1.22155781	0.00545154
ENSG00000133401	1.28767512	0.03259487
ENSG00000133424	1.24100077	0.03273259
ENSG00000133561	1.21610687	7.02E-05
ENSG00000133574	1.27793499	0.00340253
ENSG00000133612	1.25736669	3.23E-06
ENSG00000133657	-1.3796042	0.00238125
ENSG00000133773	-1.2063097	0.00441862
ENSG00000133794	-1.2402594	0.00226686
ENSG00000133874	-1.4449769	1.81E-06
ENSG00000134057	-1.2879521	0.00041235
ENSG00000134215	-1.3244662	0.00319853
ENSG00000134363	-1.2901367	0.00300012
ENSG00000134690	-1.2223262	0.03558934
ENSG00000134780	1.40769111	8.96E-06
ENSG00000135083	1.22506809	0.00784649
ENSG00000135272	-1.3403686	3.62E-05
ENSG00000135312	1.26648952	6.43E-05
ENSG00000135362	1.24486606	0.00106211
ENSG00000135537	1.34021065	0.00055531
ENSG00000135547	-2.5348493	9.81E-10
ENSG00000135604	1.40122476	4.83E-07
ENSG00000135709	1.2084408	0.00618434
ENSG00000135723	-1.2306667	5.57E-06

Gene ID	FC 4NG-8NG	adj p-value 4NG - 8NG
ENSG00000135845	-1.2245446	0.01049584
ENSG00000135966	1.56683391	3.50E-12
ENSG00000136044	-1.4213162	6.77E-09
ENSG00000136114	1.2835985	5.91E-05
ENSG00000136237	1.20273371	0.01187625
ENSG00000136244	-1.6233763	8.26E-05
ENSG00000136383	-1.2925434	7.69E-05
ENSG00000136490	-1.3214722	6.31E-06
ENSG00000136560	1.21663874	0.0036034
ENSG00000136630	-1.6694405	6.50E-05
ENSG00000136709	1.25979042	3.80E-10
ENSG00000136770	-1.2155323	2.92E-05
ENSG00000136869	-1.2547822	0.00013123
ENSG00000136935	1.2929285	3.14E-08
ENSG00000137094	-1.4515816	1.11E-08
ENSG00000137145	1.3635718	7.81E-09
ENSG00000137177	-1.2306142	6.95E-07
ENSG00000137193	1.43260069	0.0049646
ENSG00000137203	-1.3224342	0.00060213
ENSG00000137310	1.28503141	0.00452223
ENSG00000137331	1.30290542	0.00030322
ENSG00000137486	1.20953077	0.00066502
ENSG00000137494	-1.3362358	2.73E-06
ENSG00000137502	-1.2261513	0.03448787
ENSG00000137692	-1.2023162	5.96E-05
ENSG00000137693	-1.3152086	0.00043945
ENSG00000137713	-1.2228555	3.10E-06
ENSG00000137727	-1.3039447	0.00214789
ENSG00000137834	-4.8598955	1.48E-14
ENSG00000137872	-1.6029542	2.77E-06
ENSG00000137877	1.34119852	0.01116775
ENSG00000138134	1.48200234	9.79E-06

Gene ID	FC 4NG-8NG	adj p-value 4NG - 8NG
ENSG00000138623	-1.6314017	0.00032165
ENSG00000138650	-1.2566507	0.01884669
ENSG00000138675	-1.4433394	0.00017565
ENSG00000138678	1.5030763	4.98E-08
ENSG00000138764	-1.3025433	0.02432769
ENSG00000138771	1.28084376	0.00342619
ENSG00000138944	-1.2406739	0.0023903
ENSG00000139117	1.22413362	0.00240142
ENSG00000139174	1.28600493	0.00010806
ENSG00000139505	-1.2165142	0.02593114
ENSG00000139517	-1.2089871	0.00158637
ENSG00000139725	-1.2955924	0.00114288
ENSG00000139926	1.25507179	4.66E-06
ENSG00000139946	-1.2736314	0.01824078
ENSG00000140451	-1.2936431	0.04091387
ENSG00000140464	1.20455256	6.15E-06
ENSG00000141068	1.25721413	0.000105
ENSG00000141431	-1.2322235	0.02850973
ENSG00000141441	-1.2221545	0.04299983
ENSG00000141655	1.32713446	0.0003758
ENSG00000141664	1.23231135	7.46E-05
ENSG00000141668	1.44117449	0.03153488
ENSG00000142279	-1.4598018	4.00E-07
ENSG00000142459	1.20190703	0.00498764
ENSG00000142627	1.20821513	0.00020644
ENSG00000142945	-1.2817626	0.00811216
ENSG00000143344	1.28154804	3.93E-06
ENSG00000143375	1.3797075	0.00073959
ENSG00000143409	1.22894046	0.01691741
ENSG00000143458	1.31419596	0.00010629
ENSG00000143476	1.74491809	4.42E-08
ENSG00000143479	-1.4504799	3.38E-06

Gene ID	FC 4NG-8NG	adj p-value 4NG - 8NG
ENSG00000143507	-1.4416309	4.77E-06
ENSG00000143702	-1.2407896	0.02477728
ENSG00000143772	1.5383288	1.05E-09
ENSG00000143801	1.26601163	0.00136179
ENSG00000143816	1.52534194	0.00153453
ENSG00000143850	-1.2900847	0.00086495
ENSG00000143851	-1.4002051	0.00995893
ENSG00000144354	1.43817985	6.29E-07
ENSG00000144366	-1.446113	6.00E-06
ENSG00000144445	-1.2564883	0.04049097
ENSG00000144642	1.36306228	3.51E-06
ENSG00000144712	1.22067501	0.00553887
ENSG00000144802	-1.4744026	0.01078835
ENSG00000144843	1.34051739	0.00108767
ENSG00000144893	-1.2259	0.00184971
ENSG00000145012	1.23310896	0.02758174
ENSG00000145014	1.29598417	2.82E-06
ENSG00000145016	1.27190581	1.26E-07
ENSG00000145147	1.23151962	1.20E-05
ENSG00000145365	-1.3764579	5.82E-05
ENSG00000145386	-1.2365651	0.00344689
ENSG00000145817	-1.3677565	8.79E-06
ENSG00000145911	1.97407543	1.46E-11
ENSG00000146021	1.33740789	0.00028167
ENSG00000146054	1.31506324	2.3835E-05
ENSG00000146232	-1.5487747	4.44E-10
ENSG00000146263	1.45219664	0.00134343
ENSG00000146555	1.42663102	0.02743781
ENSG00000146678	1.59550974	1.49E-07
ENSG00000146834	1.24261618	1.7023E-05
ENSG00000146859	-1.3396619	0.00287702
ENSG00000147082	1.22463975	0.00926411

Gene ID	FC 4NG-8NG	adj p-value 4NG - 8NG
ENSG00000147130	1.28469858	6.79E-06
ENSG00000147180	-1.229755	0.04349283
ENSG00000147202	1.43819585	6.68E-05
ENSG00000147383	-1.2057202	5.89E-05
ENSG00000147408	-1.3469863	0.0008727
ENSG00000147642	1.46490073	0.00030958
ENSG00000147650	-1.3700816	3.54E-06
ENSG00000147789	-1.2140339	0.00346688
ENSG00000147854	1.20229519	2.0671E-05
ENSG00000147862	1.26926506	7.58E-06
ENSG00000148082	1.3653357	5.13E-06
ENSG00000148411	1.28071313	6.09E-12
ENSG00000148634	1.22333271	0.00014072
ENSG00000148926	1.74944331	5.56E-08
ENSG00000149474	1.28395346	5.74E-05
ENSG00000149636	1.32438709	3.58E-06
ENSG00000150347	-1.6652391	1.83E-05
ENSG00000150457	1.24994016	1.08E-06
ENSG00000150510	1.29336745	0.00030551
ENSG00000150630	-1.607803	1.39E-10
ENSG00000151131	-1.2076503	0.02600877
ENSG00000151136	-2.1037566	3.12E-09
ENSG00000151150	1.29079792	4.01E-05
ENSG00000151151	-1.3347539	0.02923276
ENSG00000151229	-1.3188632	0.00841608
ENSG00000151338	1.32547737	0.00031496
ENSG00000151689	1.33981418	9.62E-08
ENSG00000151702	1.21321275	1.38E-07
ENSG00000151718	-1.4226669	3.01E-15
ENSG00000151748	-1.59624	3.76E-11
ENSG00000151914	1.24568536	0.04102215
ENSG00000151929	-1.3008142	2.14E-08

Gene ID	FC 4NG-8NG	adj p-value 4NG - 8NG
ENSG00000152137	1.23284529	0.00147164
ENSG00000152409	1.33265824	0.00790837
ENSG00000152518	1.39625274	4.58E-09
ENSG00000152689	-1.4446377	1.12E-05
ENSG00000152763	1.22630682	0.0470885
ENSG00000152953	-1.3950092	1.18E-06
ENSG00000153208	1.24376228	0.00516514
ENSG00000153551	-1.2422503	5.67E-05
ENSG00000153707	1.29637787	0.03766241
ENSG00000153814	-1.3350446	5.98E-05
ENSG00000153885	-1.3020085	2.54E-06
ENSG00000153993	1.24787468	0.03338213
ENSG00000154016	-1.4182062	1.77E-06
ENSG00000154122	-1.5561553	2.60E-11
ENSG00000154127	-1.2456646	4.85E-06
ENSG00000154217	-1.5473789	9.62E-07
ENSG00000154553	-1.2787891	0.00023448
ENSG00000154556	1.63751275	1.26E-07
ENSG00000154734	-1.7076122	4.62E-05
ENSG00000155011	-1.5503142	2.62E-05
ENSG00000155034	1.24160549	0.00043557
ENSG00000155100	-1.200911	0.00433751
ENSG00000155256	-1.2387693	0.00013712
ENSG00000155846	1.34729628	0.001371
ENSG00000155849	-1.2541404	4.06E-10
ENSG00000156030	1.2569281	0.00013768
ENSG00000156313	1.36864357	1.67E-07
ENSG00000156675	1.48648307	0.00020455
ENSG00000156755	1.45350552	0.01937211
ENSG00000156876	1.30417026	0.01558888
ENSG00000157111	1.35436501	1.79E-05
ENSG00000157184	-1.2081989	0.00102499

Gene ID	FC 4NG-8NG	adj p-value 4NG - 8NG
ENSG00000157216	-1.2017636	7.8204E-05
ENSG00000157240	-1.2885392	0.01036368
ENSG00000157404	2.27981701	1.12E-08
ENSG00000157456	-1.2620641	0.00055161
ENSG00000157483	-1.3555773	1.26E-10
ENSG00000157557	1.33471791	4.31E-11
ENSG00000157570	-1.3010483	2.44E-08
ENSG00000157613	-1.8288134	1.25E-05
ENSG00000157617	1.3152471	4.31E-07
ENSG00000157978	1.26062502	3.90E-06
ENSG00000158122	1.20568161	0.00022087
ENSG00000158402	-1.2322002	0.01589965
ENSG00000158458	1.23769654	0.02025638
ENSG00000158683	1.29455421	0.0210211
ENSG00000158859	-1.3279657	5.44E-05
ENSG00000158966	-1.4322317	7.86E-08
ENSG00000159259	1.32247145	0.00026673
ENSG00000159733	2.28609873	4.42E-11
ENSG00000160113	1.2615966	5.75E-05
ENSG00000160179	1.25647624	0.02434731
ENSG00000160271	1.22730197	0.00933938
ENSG00000160685	1.28064122	5.31E-06
ENSG00000160712	1.46529615	2.60E-05
ENSG00000160949	1.22380613	0.01830347
ENSG00000161653	1.87793217	5.13E-09
ENSG00000161714	1.21072292	0.00418698
ENSG00000161800	-1.2388666	0.00496551
ENSG00000161835	-1.3241216	6.97E-05
ENSG00000162062	1.27336652	0.00927431
ENSG00000162073	1.34041763	3.55E-07
ENSG00000162367	1.20539019	9.61E-07
ENSG00000162390	-1.433967	4.64E-05

Gene ID	FC 4NG-8NG	adj p-value 4NG - 8NG
ENSG00000162407	-2.5991712	6.60E-14
ENSG00000162522	1.70331914	3.70E-14
ENSG00000162542	1.20653676	0.03525819
ENSG00000162636	-1.4750767	9.77E-06
ENSG00000162692	-2.7213424	3.98E-12
ENSG00000162772	-1.7272851	0.02560443
ENSG00000162817	1.3567408	5.07E-07
ENSG00000162869	1.22738627	0.00048054
ENSG00000163050	1.25236161	0.0309605
ENSG00000163138	-1.2174884	0.00785405
ENSG00000163171	1.75976917	1.41E-14
ENSG00000163235	-1.8104511	7.73E-08
ENSG00000163251	1.53204555	1.19E-05
ENSG00000163291	-1.200121	0.02583687
ENSG00000163297	-1.2649582	9.88E-06
ENSG00000163428	1.22113725	0.03662697
ENSG00000163462	1.24860513	0.03752426
ENSG00000163491	1.38185925	0.01126312
ENSG00000163625	1.20094917	0.00070588
ENSG00000163637	-1.2998837	0.00071298
ENSG00000163638	1.37781516	8.60E-06
ENSG00000163661	1.47705753	4.37E-07
ENSG00000163686	1.30017197	0.00097212
ENSG00000163689	1.35078904	0.00205823
ENSG00000163710	1.22748277	0.04755765
ENSG00000163734	-1.2545447	0.0048664
ENSG00000163788	1.2373393	2.01E-05
ENSG00000163840	1.23325182	0.00728247
ENSG00000163918	1.34389092	0.00090171
ENSG00000163935	-1.6232834	3.67E-06
ENSG00000163946	1.21170674	0.02203545
ENSG00000164045	1.25854707	0.00201136

Gene ID	FC 4NG-8NG	adj p-value 4NG - 8NG
ENSG00000164066	-1.2938423	0.01019042
ENSG00000164073	1.26250613	0.01198583
ENSG00000164074	1.30376512	7.68E-05
ENSG00000164161	1.25498194	1.93E-05
ENSG00000164197	-1.249851	0.02791495
ENSG00000164236	1.27739947	0.00370161
ENSG00000164241	1.31949932	0.01659977
ENSG00000164306	1.3054092	0.00013063
ENSG00000164323	1.23582018	0.00457667
ENSG00000164342	-1.2749091	0.03470194
ENSG00000164484	-1.2083187	0.00207697
ENSG00000164512	-1.683612	3.46E-06
ENSG00000164543	1.29998863	0.0002746
ENSG00000164611	-1.2613106	6.95E-06
ENSG00000164649	1.23875112	1.15E-08
ENSG00000164683	-1.5786198	0.00025975
ENSG00000164684	-1.9012486	3.11E-10
ENSG00000164736	-1.2484563	7.8477E-05
ENSG00000164741	1.20219256	9.50E-06
ENSG00000164758	-1.2283706	0.02148249
ENSG00000164823	-1.5952355	3.48E-09
ENSG00000164867	1.28988954	4.54E-05
ENSG00000165030	-1.2090718	0.04427961
ENSG00000165113	1.23931238	0.01211631
ENSG00000165118	1.2375197	0.0089746
ENSG00000165138	1.27341643	4.23E-08
ENSG00000165244	1.73940754	1.31E-08
ENSG00000165259	-1.3297824	0.00117501
ENSG00000165494	1.22410803	6.38E-05
ENSG00000165671	1.2562069	1.02E-06
ENSG00000165757	1.25047351	2.80E-10
ENSG00000165801	-1.5114951	2.59E-05

Gene ID	FC 4NG-8NG	adj p-value 4NG - 8NG
ENSG00000165891	-1.490377	1.17E-08
ENSG00000165895	-1.3839342	0.00037042
ENSG00000165899	1.20794541	0.0466919
ENSG00000165983	1.25159281	0.01747627
ENSG00000166068	-1.2556859	0.00011843
ENSG00000166289	1.41937554	9.14E-06
ENSG00000166398	-1.3639115	9.86E-08
ENSG00000166401	-1.330624	4.47E-10
ENSG00000166436	1.3242695	0.00533386
ENSG00000166446	-1.2928427	6.51E-09
ENSG00000166471	-1.3883168	1.47E-05
ENSG00000166532	-1.209649	0.03701848
ENSG00000166592	-1.2276825	0.02922331
ENSG00000166770	-1.2267847	0.03380683
ENSG00000166823	1.26892771	0.00371521
ENSG00000166831	-1.21265	0.02052032
ENSG00000166833	-1.3471653	2.15E-06
ENSG00000166851	-1.2377283	0.02210333
ENSG00000166886	-1.3767201	0.00022029
ENSG00000166897	1.26753968	0.01881191
ENSG00000166924	1.28317675	0.02597744
ENSG00000166925	1.42114513	1.96E-08
ENSG00000166938	1.3199022	3.74E-07
ENSG00000166963	1.35250185	0.00392934
ENSG00000167081	-1.3080523	4.21E-07
ENSG00000167363	1.33469185	0.00123454
ENSG00000167378	-1.2587729	6.53E-09
ENSG00000167508	-1.3005953	0.0002313
ENSG00000167513	1.48517044	5.38E-05
ENSG00000167536	1.35320103	0.00553618
ENSG00000167554	-1.2754233	0.00964283
ENSG00000167562	-1.4553661	5.12E-06

Gene ID	FC 4NG-8NG	adj p-value 4NG - 8NG
ENSG00000167600	1.34871029	0.00522739
ENSG00000167657	-1.2478952	2.09E-07
ENSG00000167670	1.39307309	0.00032198
ENSG00000167716	1.24348546	0.01951389
ENSG00000167772	1.46576917	0.00047936
ENSG00000167987	-1.2397028	0.00080102
ENSG00000168016	1.43980985	3.93E-07
ENSG00000168209	1.82716397	4.52E-07
ENSG00000168386	-1.5225078	1.98E-05
ENSG00000168461	-1.3844621	7.61E-10
ENSG00000168497	1.73224165	4.25E-15
ENSG00000168575	-1.2183438	6.16E-05
ENSG00000168672	1.60153288	6.28E-09
ENSG00000168763	1.20251808	0.01837985
ENSG00000168792	1.54613057	2.05E-09
ENSG00000168874	-1.6303612	5.19E-06
ENSG00000169184	1.63285766	2.04E-05
ENSG00000169220	1.31968563	0.00341369
ENSG00000169242	1.45603862	0.00259198
ENSG00000169247	1.29099778	0.02757449
ENSG00000169252	1.52490643	0.0001143
ENSG00000169255	1.25934565	0.00224545
ENSG00000169429	-2.5865053	1.13E-06
ENSG00000169554	-1.3062756	1.12E-09
ENSG00000169594	1.25757715	2.92E-07
ENSG00000169629	-1.2728671	0.01367751
ENSG00000169679	-1.2216446	0.01199856
ENSG00000169715	1.23979801	0.00327901
ENSG00000169744	1.24708976	2.89E-05
ENSG00000169760	1.38919526	3.11E-05
ENSG00000169871	1.27431877	1.94E-10
ENSG00000169902	-1.2050499	0.00034309

Gene ID	FC 4NG-8NG	adj p-value 4NG - 8NG
ENSG00000170264	1.37175219	6.60E-05
ENSG00000170293	-1.5584106	5.64E-07
ENSG00000170312	-1.286713	0.0244528
ENSG00000170325	1.23715839	0.00136828
ENSG00000170365	1.21688159	0.00244177
ENSG00000170425	1.22461389	0.03560269
ENSG00000170647	-1.2318143	0.04600426
ENSG00000170684	1.40740911	0.00075539
ENSG00000170775	-1.5764742	5.26E-12
ENSG00000170921	-1.3877787	5.46E-06
ENSG00000171132	-1.2534231	2.87E-05
ENSG00000171223	-1.2857859	0.00014051
ENSG00000171227	1.58358125	6.86E-07
ENSG00000171246	-1.3209706	0.03693715
ENSG00000171310	-1.576303	2.68E-08
ENSG00000171316	-1.3984459	3.96E-08
ENSG00000171345	1.20966122	0.03821564
ENSG00000171435	1.48924942	2.64E-08
ENSG00000171492	1.23931688	0.00093875
ENSG00000171552	1.3772577	2.21E-09
ENSG00000171604	-1.4034555	0.00273342
ENSG00000171617	-1.3448733	2.45E-05
ENSG00000171621	1.35669566	0.01423814
ENSG00000171813	1.284311	0.00511528
ENSG00000171940	-1.3061191	1.13E-06
ENSG00000172081	1.29325648	8.09E-09
ENSG00000172349	1.21930897	0.04552082
ENSG00000172432	-1.2337385	3.96E-05
ENSG00000172493	1.20218347	1.53E-05
ENSG00000172578	-1.2725733	2.72E-07
ENSG00000172602	-1.7614958	1.17E-06
ENSG00000172687	-1.2697198	0.03842477

Gene ID	FC 4NG-8NG	adj p-value 4NG - 8NG
ENSG00000172716	1.25660865	5.00E-10
ENSG00000172731	1.36160645	0.00017433
ENSG00000172738	-1.413268	0.00238189
ENSG00000172765	1.25326384	3.04E-07
ENSG00000172819	-1.2437119	0.01258274
ENSG00000173068	1.30463686	8.31E-07
ENSG00000173530	1.49911616	1.15E-11
ENSG00000173597	1.31421194	0.02097954
ENSG00000173868	-1.2096133	0.00247146
ENSG00000173894	1.24700139	0.00216092
ENSG00000174004	1.57962321	1.20E-05
ENSG00000174137	1.23697877	0.00662847
ENSG00000174151	-1.2005005	0.02065946
ENSG00000174306	1.32946587	2.14E-08
ENSG00000174371	1.33727748	0.02133467
ENSG00000174485	1.2054901	0.00737019
ENSG00000174791	1.35143105	0.00090244
ENSG00000175040	-1.2442211	0.00035233
ENSG00000175048	1.22449145	0.00357054
ENSG00000175063	-1.3045786	0.00293048
ENSG00000175197	-1.2578447	0.04645437
ENSG00000175264	1.32141217	1.33E-05
ENSG00000175274	-1.2747557	4.5683E-05
ENSG00000175305	1.66310352	3.92E-06
ENSG00000175591	1.4672751	0.00016488
ENSG00000175745	1.2145373	0.00014794
ENSG00000175746	-1.2849267	0.00598242
ENSG00000176102	1.60101011	6.75E-11
ENSG00000176170	-1.5769985	4.89E-11
ENSG00000176422	-1.2098933	0.03656182
ENSG00000176438	1.57902815	4.38E-11
ENSG00000176749	1.44241976	4.33E-05

Gene ID	FC 4NG-8NG	adj p-value 4NG - 8NG
ENSG00000176809	1.24418497	0.04014192
ENSG00000176845	-1.229067	0.02579225
ENSG00000176907	-2.482802	9.67E-11
ENSG00000177000	1.22768692	0.0048161
ENSG00000177283	1.2914075	9.83E-05
ENSG00000177303	1.23782829	2.62E-05
ENSG00000177374	1.53663056	1.53E-05
ENSG00000177383	1.351255	5.19E-06
ENSG00000177606	1.20467091	1.75E-05
ENSG00000177679	-1.662968	1.05E-06
ENSG00000177732	1.36872018	6.21E-07
ENSG00000178038	1.51758934	2.84E-06
ENSG00000178209	1.25298149	0.00147982
ENSG00000178401	-1.4138764	0.00131448
ENSG00000178409	-1.3422047	4.48E-07
ENSG00000178695	1.35659835	5.19E-07
ENSG00000178718	1.35459517	1.36E-07
ENSG00000178726	1.65659735	1.43E-10
ENSG00000178764	-1.3531478	0.0001016
ENSG00000179152	1.26346365	2.33E-05
ENSG00000179456	1.478362	5.63E-11
ENSG00000179532	1.56854897	1.10E-06
ENSG00000179546	1.4729368	0.00012523
ENSG00000179588	-1.2723116	0.00044657
ENSG00000179598	1.5206264	2.71E-07
ENSG00000180448	1.95823617	5.21E-08
ENSG00000180730	-1.4794617	3.36E-07
ENSG00000180758	1.2672056	0.00369992
ENSG00000180884	1.31331702	0.00041426
ENSG00000180998	-2.1501081	3.95E-09
ENSG00000181035	1.36076055	0.00399808
ENSG00000181284	1.20139648	0.03722649

Gene ID	FC 4NG-8NG	adj p-value 4NG - 8NG
ENSG00000181444	1.44964155	9.50E-05
ENSG00000181472	-1.5421099	8.07E-07
ENSG00000181541	-1.4635663	4.32E-05
ENSG00000181754	1.27990681	0.02111014
ENSG00000181938	1.23797438	0.01503221
ENSG00000182022	1.48157661	3.88E-14
ENSG00000182197	-1.5185989	4.28E-20
ENSG00000182253	-1.2308521	0.01114256
ENSG00000182378	1.30057768	0.00328974
ENSG00000182568	-1.4060429	0.00196426
ENSG00000182621	1.36376281	0.00069783
ENSG00000182700	1.36365502	0.00080017
ENSG00000182704	-1.2333402	0.00020808
ENSG00000182749	1.22625515	0.00296114
ENSG00000183208	1.30545074	0.00555537
ENSG00000183287	1.37527446	0.00026127
ENSG00000183337	1.43392376	0.00041682
ENSG00000183621	1.25401938	1.48E-05
ENSG00000183691	-1.928012	1.67E-07
ENSG00000183779	1.46729858	1.15E-05
ENSG00000183826	1.24145057	1.22E-06
ENSG00000183856	-1.2472012	0.0041449
ENSG00000184083	1.21228766	0.00305311
ENSG00000184185	1.28284274	0.04797937
ENSG00000184208	1.43000849	0.00234643
ENSG00000184226	-1.4151552	0.00026195
ENSG00000184304	-1.5309056	3.36E-11
ENSG00000184307	-1.6283602	2.39E-05
ENSG00000184371	-1.3539059	0.00013788
ENSG00000184588	-1.2078348	0.00044634
ENSG00000184702	-1.3179852	0.00873685
ENSG00000184792	-1.2187874	0.0109545

Gene ID	FC 4NG-8NG	adj p-value 4NG - 8NG
ENSG00000184992	1.31230122	2.8805E-05
ENSG00000185015	-1.4803065	8.22E-06
ENSG00000185022	1.25417556	7.4233E-05
ENSG00000185033	1.36175157	9.83E-05
ENSG00000185070	1.27032427	0.00104843
ENSG00000185112	1.77222693	5.75E-15
ENSG00000185261	1.25803691	0.02640183
ENSG00000185269	-1.3625215	3.59E-05
ENSG00000185352	1.54091498	1.11E-05
ENSG00000185453	1.35900374	0.0003493
ENSG00000185483	-1.3759003	9.38E-05
ENSG00000185551	1.29508817	2.31E-08
ENSG00000185567	1.29299056	0.00118146
ENSG00000185697	1.36463362	0.01546123
ENSG00000186104	1.22531661	0.03856
ENSG00000186260	1.2370799	0.00015735
ENSG00000186314	-1.3797964	3.06E-05
ENSG00000186354	1.33004856	0.00926349
ENSG00000186376	1.28180153	0.00014645
ENSG00000186480	-1.6171504	6.40E-07
ENSG00000186567	1.21505731	0.01719631
ENSG00000186918	1.57006295	1.76E-07
ENSG00000186951	1.27822529	0.00084685
ENSG00000187605	1.36069317	2.30E-08
ENSG00000187634	-1.4090802	0.00399525
ENSG00000187764	1.58410545	1.80E-07
ENSG00000187800	1.31868159	1.32E-08
ENSG00000188042	1.55304509	3.73E-05
ENSG00000188549	1.33832091	0.00010119
ENSG00000188596	1.4689592	5.09E-05
ENSG00000188732	1.37057292	0.02934262
ENSG00000188827	1.22662077	0.00131366

Gene ID	FC 4NG-8NG	adj p-value 4NG - 8NG
ENSG00000189056	1.24098139	0.02172969
ENSG00000189057	1.97752075	1.07E-07
ENSG00000189067	-1.2764683	0.02169428
ENSG00000189120	1.34387327	0.00030224
ENSG00000189152	-1.310184	0.04186247
ENSG00000189221	1.26768826	0.00161193
ENSG00000189223	1.31914936	0.0044819
ENSG00000196083	-1.2452893	0.00501912
ENSG00000196159	1.33633268	0.0008982
ENSG00000196196	1.29849424	0.01190724
ENSG00000196268	-1.2513133	0.01167986
ENSG00000196387	-1.219729	0.04002827
ENSG00000196449	-1.274575	1.08E-06
ENSG00000196544	-1.2209315	0.02025864
ENSG00000196576	1.20214288	0.00013393
ENSG00000196584	1.5058223	3.84E-05
ENSG00000196659	-1.2233353	0.04860953
ENSG00000196700	1.2192288	1.48E-06
ENSG00000196730	1.23561751	1.41E-07
ENSG00000196739	-1.218797	0.00267679
ENSG00000196781	1.32343417	1.03E-09
ENSG00000196782	1.46687986	3.50E-12
ENSG00000196839	-1.2523386	0.00940979
ENSG00000196843	-1.236611	0.01377168
ENSG00000196876	1.3960312	1.89E-05
ENSG00000196890	1.22528826	0.04315723
ENSG00000196922	1.21375428	0.00813617
ENSG00000197245	1.39116282	0.00824636
ENSG00000197256	1.36142576	3.72E-11
ENSG00000197343	1.23666169	0.03033063
ENSG00000197375	1.26620652	0.01181328
ENSG00000197380	-1.4717901	0.00104953

Gene ID	FC 4NG-8NG	adj p-value 4NG - 8NG
ENSG00000197385	1.2302835	0.03526096
ENSG00000197442	-1.2791314	0.00014063
ENSG00000197461	-1.3172502	0.00026322
ENSG00000197496	1.27906293	0.0011051
ENSG00000197557	-1.2263386	0.02766796
ENSG00000197619	-1.3345185	0.00519471
ENSG00000197822	1.29343939	0.01407288
ENSG00000197852	1.40268732	3.97E-08
ENSG00000197905	1.2354114	7.58E-05
ENSG00000197928	-1.2715112	0.00126033
ENSG00000197971	1.20033383	0.02156574
ENSG00000198018	-1.2885994	9.73E-06
ENSG00000198056	1.34460543	0.00236781
ENSG00000198060	-1.2066165	0.0003531
ENSG00000198108	-2.5703631	1.10E-12
ENSG00000198342	-1.2705721	0.01155815
ENSG00000198355	1.38807828	2.69E-07
ENSG00000198369	1.23774381	0.00027738
ENSG00000198435	1.40025457	0.00019173
ENSG00000198742	-1.2424878	9.18E-08
ENSG00000198774	-1.2877917	0.01578096
ENSG00000198780	1.25416302	0.01335389
ENSG00000198805	-1.252533	1.38E-08
ENSG00000198824	1.21043927	5.22E-08
ENSG00000198901	-1.2605955	0.00039713
ENSG00000198924	1.25734399	0.00956758
ENSG00000198933	1.23690669	0.00089548
ENSG00000198945	-1.2246185	8.20E-08
ENSG00000203485	1.24240877	1.52E-05
ENSG00000203883	1.40535691	0.00286648
ENSG00000204103	1.238823	0.04476756
ENSG00000204131	1.33931517	3.62E-08

Gene ID	FC 4NG-8NG	adj p-value 4NG - 8NG
ENSG00000204217	-1.3209525	8.03E-05
ENSG00000204406	1.81277047	3.45E-13
ENSG00000204604	-1.2035713	0.00120341
ENSG00000204634	1.27973267	1.11E-05
ENSG00000204776	1.33876532	0.0010723
ENSG00000204923	-1.3068134	0.02254762
ENSG00000205250	-1.2145634	8.38E-05
ENSG00000205269	1.54128396	0.00091839
ENSG00000205356	1.2757342	1.28E-06
ENSG00000205683	1.35064063	1.0645E-05
ENSG00000205726	1.22255198	1.79E-08
ENSG00000206538	-1.5867477	1.62E-07
ENSG00000206560	1.27939477	4.65E-07
ENSG00000211455	-1.646435	3.96E-07
ENSG00000212766	1.31094379	1.33E-05
ENSG00000213047	-1.285772	0.0241956
ENSG00000213085	-1.2581548	0.03649149
ENSG00000213160	1.50328968	0.00014424
ENSG00000213203	1.2748093	0.00102759
ENSG00000213626	-1.5062384	7.33E-05
ENSG00000213694	1.52330726	7.43E-13
ENSG00000214944	1.54384934	6.32E-11
ENSG00000215012	1.40462486	9.45E-07
ENSG00000215458	1.31294299	0.01845328
ENSG00000216775	1.25576376	0.01730055
ENSG00000219023	-1.2262211	0.0238868
ENSG00000223764	-1.3372925	6.02E-07
ENSG00000224870	-1.2273028	0.00415496
ENSG00000224982	-1.3164728	0.01087622
ENSG00000225377	1.27130269	0.00359499
ENSG00000225968	-2.4396577	4.12E-11
ENSG00000229809	-1.2926291	0.011303

Gene ID	FC 4NG-8NG	adj p-value 4NG - 8NG
ENSG00000229953	1.26577018	0.02204591
ENSG00000230438	1.59985382	5.97E-06
ENSG00000230487	1.52288488	9.32E-06
ENSG00000231856	-1.2423485	0.02367083
ENSG00000233117	1.31915285	0.01648126
ENSG00000233251	1.30301411	0.00056935
ENSG00000235770	1.50936326	4.30E-10
ENSG00000235954	1.27379017	0.01414994
ENSG00000236859	-1.2993338	0.00418166
ENSG00000237036	-1.3500701	0.00026921
ENSG00000237440	-1.3361778	0.00093331
ENSG00000239305	-1.2640832	0.00037993
ENSG00000241015	-1.3782822	2.21E-05
ENSG00000241764	-1.2894089	0.0010108
ENSG00000242779	1.26005549	0.00257699
ENSG00000244462	-1.2155759	0.00015321
ENSG00000244694	1.31845854	0.00591665
ENSG00000245556	-1.2323917	0.01726497
ENSG00000247809	1.23852637	0.04586732
ENSG00000248008	-1.3154158	0.00243223
ENSG00000249279	1.63986867	3.48E-07
ENSG00000249992	-1.4488441	2.04E-07
ENSG00000250588	-1.2199953	0.03362714
ENSG00000250899	1.62771552	1.48E-11
ENSG00000251247	-1.2957371	0.01110609
ENSG00000253882	1.30276008	5.55E-06
ENSG00000254004	-1.2168207	0.01909892
ENSG00000254087	-1.4407538	3.05E-11
ENSG00000254726	-1.3520967	3.22E-06
ENSG00000255112	-1.2516209	3.17E-06
ENSG00000255624	1.26261219	0.01415196
ENSG00000256628	-1.3002335	0.00116017

Gene ID	FC 4NG-8NG	adj p-value 4NG - 8NG
ENSG00000257219	1.21635033	0.00071601
ENSG00000257923	-1.2118597	5.12E-05
ENSG00000258441	1.38865655	0.00015
ENSG00000258634	1.20819957	0.01057101
ENSG00000259330	1.27615124	0.00322058
ENSG00000259768	1.28648405	0.03189313
ENSG00000259877	-1.2337262	0.0459677
ENSG00000259943	-1.2561659	0.01810023
ENSG00000260231	-1.2093785	0.03736768
ENSG00000260236	-1.2699231	0.00721019
ENSG00000260267	-1.2017265	0.03585121
ENSG00000260274	-1.2893434	0.02119959
ENSG00000260401	1.37370093	0.01721586
ENSG00000260461	1.21487517	0.04379847
ENSG00000260804	1.32999889	3.17E-05
ENSG00000260822	1.21907095	0.01683147
ENSG00000260996	-1.427839	0.00056829
ENSG00000261534	1.30499297	0.04030082
ENSG00000262001	-1.2218646	0.01679784
ENSG00000262601	-1.3515112	0.00346175
ENSG00000263013	-1.314458	0.01355991
ENSG00000265972	-1.4392855	2.29E-06
ENSG00000266680	-1.2996463	0.01071043
ENSG00000267100	-1.2103076	0.02777391
ENSG00000267532	-1.2505465	0.01557724
ENSG00000269743	-1.2381625	0.00459299
ENSG00000269906	-1.2298073	0.01121131
ENSG00000269973	1.29803091	0.01035134
ENSG00000270170	-1.2141544	0.03639966
ENSG00000271430	1.27416736	0.0112975
ENSG00000271590	1.30741398	0.00034395
ENSG00000271976	1.39057322	0.00072639

Gene ID	FC 4NG-8NG	adj p-value 4NG - 8NG
ENSG00000272447	-1.2705203	0.00206939
ENSG00000272768	-1.2221972	0.04506451
ENSG00000272886	-1.2222855	1.02E-05
ENSG00000273270	-1.3081858	0.00401671
ENSG00000273780	1.33462445	5.43E-05
ENSG00000274995	-1.3267796	0.04427049
ENSG00000275031	-1.2708494	0.0164651
ENSG00000275131	-1.2822887	0.03308489
ENSG00000275342	1.24079501	0.00501908
ENSG00000275832	1.3613718	0.00017902
ENSG00000275993	1.31661031	0.00642037
ENSG00000276043	1.49747167	6.54E-09
ENSG00000276644	-1.7261584	3.13E-07
ENSG00000276728	-1.2393398	0.01924915
ENSG00000277775	-1.263529	0.03081442
ENSG00000277778	1.37127276	0.0294364
ENSG00000278191	-1.3325693	0.00863244
ENSG00000279117	1.25599174	1.99E-05
ENSG00000279207	1.27025318	0.00344356
ENSG00000279348	1.21341127	0.03145371
ENSG00000279822	1.24678792	0.01104981
ENSG00000281614	1.23395617	0.01993776
ENSG00000281840	1.317354	0.0042272

Supplementary Table 2d. Differentially expressed genes between 8hr and 24hr normal glucose samples

Ensembl gene IDs, Fold change (8NG-24NG) and FDR adjusted p-values of DEGs

Gene ID	FC 8NG-24NG	adj p-value 8NG-24NG
ENSG00000000971	-1.2083779	0.01093665
ENSG00000003989	1.26507674	0.00310913
ENSG00000004799	-1.5711013	4.97E-05
ENSG00000005882	-1.2207379	0.03020625
ENSG00000006042	-1.2359309	0.00045398
ENSG00000006831	1.24156491	9.65E-06
ENSG00000008294	1.26157348	1.03E-11
ENSG00000008441	-1.2409271	2.90E-09
ENSG00000010818	-1.4252757	4.87E-05
ENSG00000011007	1.20768806	1.07E-06
ENSG00000011523	-1.2175082	0.00039211
ENSG00000013588	-1.6032278	1.05E-06
ENSG00000019144	1.36094471	2.83E-08
ENSG00000021355	-1.2533469	0.004189
ENSG00000021645	1.24753759	0.00151408
ENSG00000033327	-1.2038462	4.50E-05
ENSG00000033867	1.29159066	0.00067343
ENSG00000048052	1.88619246	6.70E-06
ENSG00000049759	1.37711995	4.18E-08
ENSG00000051128	-1.2868833	2.20E-07
ENSG00000058091	1.23412171	0.00215131
ENSG00000059378	-1.3397809	1.92E-07
ENSG00000064042	-1.8935998	2.80E-10
ENSG00000064651	1.29117741	0.00138632
ENSG00000064692	-1.4438956	0.01731712
ENSG00000064989	1.32587861	0.00104589
ENSG00000065054	1.25266759	0.00343235
ENSG00000065057	-1.2210482	0.0011216
ENSG00000065618	-1.2359256	0.01892305
ENSG00000065809	1.24400019	1.78E-07
ENSG00000067221	1.29483395	0.00172378
ENSG00000067798	1.20181514	0.00023901

Gene ID	FC 8NG-24NG	adj p-value 8NG-24NG
ENSG00000067992	-1.2012136	0.01432507
ENSG00000068078	1.34436485	0.004443
ENSG00000068366	1.20825222	1.7167E-05
ENSG00000069122	-1.6076718	2.00E-05
ENSG00000070495	1.22124937	0.00262516
ENSG00000071073	1.21781653	4.73E-05
ENSG00000071246	-1.2971016	0.02680555
ENSG00000072786	1.2072052	6.29E-05
ENSG00000073849	-1.3523144	2.23E-09
ENSG00000073921	1.28449937	6.54E-05
ENSG00000074527	-1.6254671	2.83E-05
ENSG00000074590	1.2695258	0.01845328
ENSG00000075711	-1.289306	7.68E-05
ENSG00000076356	1.22576775	7.36E-08
ENSG00000076928	1.24589541	0.00335384
ENSG00000077092	-1.8141713	2.16E-05
ENSG00000078018	1.79114308	2.92E-08
ENSG00000078081	-1.281147	0.00728048
ENSG00000079102	-1.2330855	0.00308766
ENSG00000079335	1.28059488	0.03060072
ENSG00000079385	-1.5392226	2.48E-05
ENSG00000081059	1.22397731	0.00350058
ENSG00000084444	-1.2308889	0.01349183
ENSG00000084731	-1.2558319	1.23E-05
ENSG00000086696	-1.4763169	0.00697891
ENSG00000087303	1.24035366	0.00060986
ENSG00000088280	-1.3246424	0.00028812
ENSG00000088367	-1.3785806	0.00157076
ENSG00000088448	-1.2385485	0.0037286
ENSG00000088986	1.25575477	2.53E-10
ENSG00000089127	-1.752559	0.00052565
ENSG00000089351	-1.2364216	0.00026417

Gene ID	FC 8NG-24NG	adj p-value 8NG-24NG
ENSG00000090238	-1.6135943	1.42E-08
ENSG00000091129	1.34504038	1.83E-08
ENSG00000091409	1.33180047	8.37E-09
ENSG00000091656	1.31430083	0.0010801
ENSG00000091879	1.46309576	1.70E-14
ENSG00000091972	-1.4310951	0.00680623
ENSG00000092871	-1.2197633	0.02909223
ENSG00000099204	-1.4621635	3.82E-07
ENSG00000099256	-1.224403	0.00581011
ENSG00000099889	-1.3061414	0.00013018
ENSG00000099937	-1.6770086	1.48E-06
ENSG00000100027	-1.37573	7.72E-05
ENSG00000100100	-2.3105885	4.49E-10
ENSG00000100292	-1.3213371	0.02091427
ENSG00000100311	1.26013378	0.00830592
ENSG00000100342	-1.2919063	0.00022977
ENSG00000100350	-1.2686026	0.00052247
ENSG00000100439	-1.2821752	4.0922E-05
ENSG00000100697	1.26312739	1.01E-06
ENSG00000100784	-1.5029158	0.00030718
ENSG00000100906	-1.2281924	0.01660582
ENSG00000100918	-1.2273297	3.47E-07
ENSG00000101096	-1.5079714	1.17E-05
ENSG00000101188	-1.2997755	0.00217887
ENSG00000101445	-1.4237153	0.00014737
ENSG00000101670	1.26940421	0.00024731
ENSG00000101871	-1.4192289	1.29E-07
ENSG00000102010	-1.3898661	2.29E-08
ENSG00000102024	1.21210529	2.07E-07
ENSG00000102302	-1.2487067	0.00648529
ENSG00000102452	-1.297666	0.01112693
ENSG00000102755	1.41852752	1.41E-10

Gene ID	FC 8NG-24NG	adj p-value 8NG-24NG
ENSG00000102802	-1.8046753	2.60E-06
ENSG00000102908	-1.2613782	0.02198206
ENSG00000103024	-1.2607562	2.68E-06
ENSG00000103034	-1.4388223	1.05E-08
ENSG00000103647	-1.3060711	8.68E-06
ENSG00000104081	-1.5205842	0.00293252
ENSG00000104447	-1.2188022	0.00110942
ENSG00000105088	2.00420423	5.59E-11
ENSG00000105270	-1.5258218	0.00051791
ENSG00000105327	-1.3251736	0.00270172
ENSG00000105810	1.37496563	1.49E-07
ENSG00000105825	1.50302308	1.36E-16
ENSG00000105996	-1.2291594	0.00594229
ENSG00000106537	1.36103516	5.15E-10
ENSG00000106560	-1.2478877	3.37E-05
ENSG00000106780	1.21514819	0.02534425
ENSG00000106852	-1.4721814	1.91E-07
ENSG00000106853	-1.2006526	0.02429414
ENSG00000107438	-1.2173406	4.33E-06
ENSG00000107562	3.94669166	7.83E-22
ENSG00000108001	-1.2636836	0.0081075
ENSG00000108448	-1.3444761	0.04070462
ENSG00000108551	1.55640985	1.18E-06
ENSG00000108622	-1.2775429	5.22E-11
ENSG00000108691	-2.2304769	6.72E-08
ENSG00000108984	-2.1882293	8.95E-06
ENSG00000109046	-1.2604176	0.00087535
ENSG00000109906	1.83003932	1.49E-08
ENSG00000110002	-1.2109245	0.00693526
ENSG00000110042	-1.3311388	0.00801346
ENSG00000110090	-1.2928402	4.59E-06
ENSG00000110092	1.37867653	5.34E-09

Gene ID	FC 8NG-24NG	adj p-value 8NG-24NG
ENSG00000110455	-1.3078074	0.0041616
ENSG00000110799	-1.2499122	0.00749404
ENSG00000110900	-1.2852508	0.04477
ENSG00000110987	-1.206004	0.01855212
ENSG00000111145	-1.2763971	1.42E-08
ENSG00000111335	-1.5141226	0.00324178
ENSG00000111341	1.32561917	1.21E-09
ENSG00000111653	-1.2926736	0.04933964
ENSG00000111801	-1.4052984	0.00090295
ENSG00000111880	1.2388952	0.01376197
ENSG00000112414	-1.344888	0.00035732
ENSG00000112972	1.25657782	0.01850902
ENSG00000113319	1.29467847	6.83E-08
ENSG00000113552	-1.2473018	1.39E-09
ENSG00000114126	-1.3964217	6.54E-08
ENSG00000114480	1.3807202	3.71E-12
ENSG00000114698	-1.6128526	0.0002424
ENSG00000114796	-1.6033159	2.4676E-05
ENSG00000115091	1.20976554	7.81E-07
ENSG00000115109	-1.2620283	0.00901711
ENSG00000115415	-1.3090169	1.91E-05
ENSG00000115525	-1.4508306	3.41E-06
ENSG00000116133	1.25925642	3.52E-07
ENSG00000116473	1.24527051	5.60E-09
ENSG00000116711	2.13715119	1.17E-06
ENSG00000116990	-1.2900046	0.01074208
ENSG00000117226	-1.2059557	5.71E-05
ENSG00000117394	1.25340865	0.00334836
ENSG00000117399	-1.2089721	0.00054798
ENSG00000117595	1.52183938	0.04517522
ENSG00000117707	-1.2583634	0.01956999
ENSG00000118407	-1.6755655	6.04E-06

Gene ID	FC 8NG-24NG	adj p-value 8NG-24NG
ENSG00000118503	-1.6296726	0.00097212
ENSG00000118777	-1.3268914	0.03501176
ENSG00000118946	2.38487737	1.22E-06
ENSG00000119326	-1.2414606	0.00827988
ENSG00000119686	-1.3557496	8.25E-06
ENSG00000119917	-1.3628635	0.02672802
ENSG00000119922	-1.3177047	1.03E-05
ENSG00000119950	-1.4858276	7.04E-05
ENSG00000120093	-1.2449938	0.0028711
ENSG00000120217	1.3651889	7.37E-06
ENSG00000120437	1.380053	1.04E-08
ENSG00000120549	-1.5914981	0.00427187
ENSG00000120756	-1.2262851	0.04377328
ENSG00000120910	1.22712903	0.00109078
ENSG00000121210	-1.2690889	0.00256002
ENSG00000121858	-2.6806031	3.29E-11
ENSG00000122378	-1.2642608	2.16E-08
ENSG00000122861	-1.512091	4.67E-05
ENSG00000123689	-1.3785325	0.00033922
ENSG00000123908	1.2395695	1.11E-08
ENSG00000124145	-1.2502288	0.00879502
ENSG00000124225	-1.3280383	0.01374859
ENSG00000124508	-1.3882868	0.00040541
ENSG00000124575	-1.3325949	0.00020984
ENSG00000124610	-1.5235564	1.16E-08
ENSG00000124635	-1.2962581	0.01048002
ENSG00000124766	-1.3592369	6.80E-06
ENSG00000124875	-1.5275572	1.22E-05
ENSG00000125347	-1.2808851	0.01544053
ENSG00000125772	1.23302637	0.00226688
ENSG00000125864	-1.2730141	0.01802452
ENSG00000125966	1.49798885	0.00016092

Gene ID	FC 8NG-24NG	adj p-value 8NG-24NG
ENSG00000126351	-1.316291	1.29E-08
ENSG00000126785	-1.3062598	5.64E-09
ENSG00000127220	-1.3310054	0.00812342
ENSG00000127528	1.40648149	0.00486267
ENSG00000128284	-1.2219622	0.03705718
ENSG00000128294	-1.2575816	7.11E-10
ENSG00000128567	1.81914712	6.23E-17
ENSG00000128602	-1.3196695	0.00054187
ENSG00000128656	-1.370964	3.73E-06
ENSG00000128815	-1.2994849	0.0161945
ENSG00000128849	-1.3996379	5.05E-09
ENSG00000129116	-1.2981068	0.00887351
ENSG00000129538	-1.2765724	0.00031484
ENSG00000130052	1.27991994	0.00041062
ENSG00000130270	-1.2228502	0.00055539
ENSG00000130300	1.58438944	3.37E-06
ENSG00000130513	-1.5316685	8.73E-10
ENSG00000130830	-1.229985	0.00022355
ENSG00000130956	1.29495858	0.00435701
ENSG00000131016	1.88238346	4.67E-19
ENSG00000131069	1.20698815	0.04268702
ENSG00000131389	-1.3235484	2.97E-09
ENSG00000131409	1.9101716	0.01034834
ENSG00000131634	1.26367332	1.93E-07
ENSG00000131669	-1.3201251	3.49E-06
ENSG00000132003	-1.2798893	0.00015075
ENSG00000132510	-1.4185775	1.31E-06
ENSG00000132530	-1.436719	0.00316758
ENSG00000132622	-1.2513387	0.0322051
ENSG00000132846	-1.2354743	8.07E-07
ENSG00000132970	-1.2757107	0.00085706
ENSG00000133056	-1.4478943	8.19E-05

Gene ID	FC 8NG-24NG	adj p-value 8NG-24NG
ENSG00000133216	-1.3274886	1.59E-07
ENSG00000133424	1.55303183	3.53E-05
ENSG00000133639	-1.4324501	1.06E-05
ENSG00000133657	1.34403729	0.00354678
ENSG00000133800	-1.5895564	1.65E-05
ENSG00000133863	-1.2634915	0.01913831
ENSG00000134138	-1.2179552	0.0054859
ENSG00000134291	-1.2046823	0.00350058
ENSG00000134363	1.36120722	0.00015802
ENSG00000134508	-1.8675515	8.63E-10
ENSG00000134531	1.3309733	1.61E-16
ENSG00000134574	-1.2602237	1.55E-08
ENSG00000134986	-1.2873114	7.85E-06
ENSG00000135074	-1.3348804	1.87E-06
ENSG00000135363	-1.245708	0.00025328
ENSG00000135365	-1.2256846	9.11E-06
ENSG00000135643	-1.351062	0.02313226
ENSG00000135842	-1.2097129	0.00023777
ENSG00000135914	-1.6957823	0.00055081
ENSG00000135919	1.21801403	1.57E-07
ENSG00000135929	-1.2604697	0.00194809
ENSG00000136111	1.24242083	9.64E-07
ENSG00000136205	1.28044733	7.95E-06
ENSG00000136231	1.24649449	0.00097128
ENSG00000136305	-1.3448452	0.00071429
ENSG00000136383	-1.2484201	0.007688
ENSG00000136717	-1.2922682	0.00032831
ENSG00000136859	-1.2585612	0.0004048
ENSG00000137094	-1.3131883	1.71E-06
ENSG00000137198	-1.2180854	0.00117398
ENSG00000137463	-1.2597414	0.00108966
ENSG00000137486	-1.245406	0.00087843

Gene ID	FC 8NG-24NG	adj p-value 8NG-24NG
ENSG00000137507	-1.3262311	0.00015208
ENSG00000137801	1.21815849	1.75E-08
ENSG00000137831	1.35099031	3.67E-06
ENSG00000138356	-1.274352	7.6322E-05
ENSG00000138411	1.2311639	1.07E-07
ENSG00000138448	1.24860953	9.23E-06
ENSG00000138449	-1.2875107	0.0024642
ENSG00000138496	-1.4290792	3.59E-06
ENSG00000138764	-1.6100469	0.00186837
ENSG00000138767	1.26108901	0.00025581
ENSG00000138771	1.618888	3.82E-06
ENSG00000138794	-1.2390872	0.00059025
ENSG00000139112	-1.2806866	3.36E-05
ENSG00000139211	-1.5651158	0.01044602
ENSG00000139428	1.20487793	4.97E-05
ENSG00000139508	-1.2943039	0.00193994
ENSG00000139899	-1.2974025	0.01689749
ENSG00000139946	-1.4621743	0.00156913
ENSG00000140092	1.28850895	1.06E-05
ENSG00000140263	-1.2549585	5.21E-07
ENSG00000140416	1.23992887	3.90E-07
ENSG00000140564	1.26505117	5.42E-06
ENSG00000140873	-1.967966	1.58E-08
ENSG00000141068	-1.2441042	4.57E-05
ENSG00000141447	-1.2126781	0.01435032
ENSG00000141574	-1.4284557	0.00088319
ENSG00000141668	2.1205446	3.19E-06
ENSG00000141858	1.23293663	0.01200801
ENSG00000142661	1.40687368	0.00889319
ENSG00000143127	1.20362071	0.00013945
ENSG00000143153	1.23196635	7.30E-07
ENSG00000143344	1.34344613	7.38E-08

Gene ID	FC 8NG-24NG	adj p-value 8NG-24NG
ENSG00000143387	-1.3605097	0.01902188
ENSG00000143434	-1.4154592	1.02E-07
ENSG00000143819	-1.5014993	9.18E-06
ENSG00000143850	-1.2036505	0.00088943
ENSG00000143891	-1.2884711	0.01645147
ENSG00000144063	2.02290993	2.85E-16
ENSG00000144366	1.37826822	0.00012347
ENSG00000144476	-1.3949816	0.00012179
ENSG00000144560	-1.3201853	1.64E-07
ENSG00000144642	1.29262519	5.73E-06
ENSG00000144644	1.35361704	0.00237497
ENSG00000144824	-1.2198846	0.01472119
ENSG00000145365	-1.3092073	0.00015547
ENSG00000145990	1.24480478	0.00258025
ENSG00000146021	-1.2848434	0.03476182
ENSG00000146083	-1.2860817	4.58E-05
ENSG00000146278	-1.3260607	0.00013296
ENSG00000146386	1.27605126	7.64E-05
ENSG00000146592	-1.2330539	0.00745689
ENSG00000146859	-1.4748226	1.36E-05
ENSG00000147027	1.23735411	0.00523634
ENSG00000147113	-1.58166	7.15E-06
ENSG00000147202	1.39917434	1.96E-07
ENSG00000147408	-1.5120169	0.00010972
ENSG00000148803	1.21766688	0.01894093
ENSG00000148841	-1.2320129	0.00350778
ENSG00000148842	-1.3888417	0.01039656
ENSG00000149212	-1.59749	2.18E-07
ENSG00000149269	-1.522068	0.00026525
ENSG00000149573	-1.3150193	0.00856735
ENSG00000149582	-1.3124392	0.00481319
ENSG00000149591	1.24655842	0.0019984

Gene ID	FC 8NG-24NG	adj p-value 8NG-24NG
ENSG00000149948	1.34165896	8.52E-05
ENSG00000150593	-1.2314606	0.00411157
ENSG00000151012	1.27179137	0.00810228
ENSG00000151023	1.62559873	0.00010303
ENSG00000151322	1.25602372	0.00336587
ENSG00000151414	1.25266939	0.0014608
ENSG00000151692	-1.4540877	0.00429136
ENSG00000151835	1.24135614	0.04561359
ENSG00000151967	-1.2044366	0.04650122
ENSG00000152284	-1.3384578	1.27E-06
ENSG00000152402	1.32520488	0.00954671
ENSG00000152601	1.37592287	3.35E-09
ENSG00000152661	1.23555656	1.06E-07
ENSG00000152689	1.24870514	0.00018111
ENSG00000152953	-1.2525976	7.71E-05
ENSG00000153094	-1.6727941	1.0207E-05
ENSG00000153208	-1.2562019	0.02477179
ENSG00000153707	1.67818772	0.00103043
ENSG00000153956	1.2478294	0.00011021
ENSG00000153993	1.30040787	0.01997565
ENSG00000154556	1.40534894	1.80E-06
ENSG00000154930	-1.7043716	0.00092209
ENSG00000155111	-1.2488975	0.01498034
ENSG00000155657	1.4299182	0.01162369
ENSG00000155755	-1.2361941	0.00037299
ENSG00000155849	-1.2252808	0.00311758
ENSG00000155966	1.33272264	0.01532656
ENSG00000156049	-1.4824478	0.00802002
ENSG00000156265	1.50720395	2.38E-08
ENSG00000156313	1.35202381	2.61E-05
ENSG00000156463	-1.4750785	0.00054612
ENSG00000156587	-1.2859064	0.00435527

Gene ID	FC 8NG-24NG	adj p-value 8NG-24NG
ENSG00000156711	-1.354524	0.00386986
ENSG00000157483	1.25745003	2.96E-07
ENSG00000157570	-1.2322983	5.72E-06
ENSG00000157613	-1.2966325	0.03096577
ENSG00000157617	-1.2035513	0.00462853
ENSG00000157827	-1.2436863	0.00048102
ENSG00000158286	-1.3709271	0.04773437
ENSG00000158373	-1.3126574	0.00066291
ENSG00000158406	-1.3051489	0.00138509
ENSG00000158555	-1.4655767	2.09E-13
ENSG00000158715	-1.2353347	0.00834135
ENSG00000159388	-1.2312169	0.00741869
ENSG00000160013	-1.3176263	0.00741302
ENSG00000160145	-1.3480095	0.01864283
ENSG00000160179	-1.7505765	2.25E-07
ENSG00000160219	-1.2407607	0.03907143
ENSG00000160285	1.22006298	0.00834878
ENSG00000160752	1.22674059	3.05E-06
ENSG00000161011	-1.3181551	2.38E-07
ENSG00000161542	-1.2343662	1.48E-09
ENSG00000161682	-1.4701601	8.69E-05
ENSG00000161714	-1.2178201	0.00140545
ENSG00000162174	-1.3204953	0.04593404
ENSG00000162337	-1.2355392	0.00012234
ENSG00000162407	-1.3541065	0.01057187
ENSG00000162434	1.51047731	3.61E-16
ENSG00000162496	-2.2031077	2.38E-12
ENSG00000162551	1.32385266	0.00029113
ENSG00000162576	-1.2605254	0.00472183
ENSG00000162599	-1.320981	0.00224091
ENSG00000162618	1.21283207	0.02576311
ENSG00000162692	1.24042579	0.00170423

Gene ID	FC 8NG-24NG	adj p-value 8NG-24NG
ENSG00000162733	-1.2826649	2.78E-08
ENSG00000163110	1.35985487	4.15E-15
ENSG00000163171	1.26197022	0.00063216
ENSG00000163346	-1.3359356	0.00010989
ENSG00000163554	-1.4604225	0.00584169
ENSG00000163565	-1.2178408	0.00070841
ENSG00000163596	1.21749407	0.01695827
ENSG00000163637	-1.6440083	3.70E-07
ENSG00000163739	-1.2896848	0.02168132
ENSG00000163840	-1.2303259	0.02308506
ENSG00000164038	1.21416867	0.04358563
ENSG00000164056	1.36255597	0.00182292
ENSG00000164161	1.26678507	4.26E-08
ENSG00000164237	-1.3108638	0.00016565
ENSG00000164283	1.36173993	8.53E-06
ENSG00000164284	1.37499135	0.00303891
ENSG00000164342	-1.3294501	0.00317467
ENSG00000164463	-1.2891747	0.00193825
ENSG00000164615	-1.2040329	0.01346254
ENSG00000164649	-1.2044414	1.48E-07
ENSG00000164687	-1.4480933	0.00111376
ENSG00000164736	1.32274591	2.49E-05
ENSG00000164849	-2.0752104	0.0009343
ENSG00000164932	-1.2037491	0.00056962
ENSG00000164985	-1.2054298	0.00529477
ENSG00000165029	-3.1471554	2.54E-11
ENSG00000165092	-2.3662458	1.06E-09
ENSG00000165244	1.29171201	0.03695007
ENSG00000165312	-1.3570996	0.0319303
ENSG00000165494	1.22460778	0.0050191
ENSG00000165804	-1.2807426	7.34E-05
ENSG00000165821	-1.5134723	2.82E-06

Gene ID	FC 8NG-24NG	adj p-value 8NG-24NG
ENSG00000165861	-1.201864	0.00044529
ENSG00000165912	-1.3914683	8.37E-10
ENSG00000165959	-1.4964042	1.98E-09
ENSG00000166046	-1.5653607	0.00555247
ENSG00000166073	1.26061852	7.34E-05
ENSG00000166265	-1.3225463	0.00022461
ENSG00000166313	-1.2515475	1.41E-06
ENSG00000166439	-1.3809092	2.58E-07
ENSG00000166503	1.20490035	6.32E-05
ENSG00000166670	1.61151711	4.31E-09
ENSG00000166741	-1.2634142	0.04319183
ENSG00000167106	-1.3094115	0.00037559
ENSG00000167107	-1.3807062	1.42E-05
ENSG00000167508	1.31539311	0.00043001
ENSG00000167552	1.26815433	1.75E-06
ENSG00000167680	-1.2149468	1.13E-05
ENSG00000167693	-1.3200234	8.2646E-05
ENSG00000167874	-1.4168176	3.86E-05
ENSG00000168298	-1.3583574	5.13E-06
ENSG00000168300	-1.3062881	0.0001402
ENSG00000168309	-1.3742459	8.07E-08
ENSG00000168374	1.21617871	5.11E-07
ENSG00000168575	1.37401624	6.21E-07
ENSG00000168646	-1.21135	0.01098667
ENSG00000168672	-1.2822749	0.01644966
ENSG00000168906	1.33584907	1.69E-09
ENSG00000168916	-1.327141	0.00018565
ENSG00000168961	-1.4339189	0.00018717
ENSG00000169083	-1.4481398	7.54E-09
ENSG00000169169	-1.3355077	0.01415968
ENSG00000169242	-1.3526539	0.01460145
ENSG00000169504	1.28624516	0.00122149

Gene ID	FC 8NG-24NG	adj p-value 8NG-24NG
ENSG00000169710	1.35393949	2.86E-06
ENSG00000169740	-1.2676233	0.00125188
ENSG00000169908	1.21969294	2.22E-05
ENSG00000170011	-1.344729	0.00142088
ENSG00000170271	-1.7875764	3.18E-10
ENSG00000170425	-1.3326931	0.03457957
ENSG00000170456	-1.3611008	1.06E-05
ENSG00000170485	-1.2201465	1.27E-05
ENSG00000170522	1.29443504	1.54E-07
ENSG00000170558	1.24512739	3.46E-07
ENSG00000170915	1.2129129	0.03820388
ENSG00000171132	-1.3470507	3.08E-06
ENSG00000171246	3.66143102	3.26E-14
ENSG00000171345	-1.2045284	0.00225294
ENSG00000171425	-1.2020233	0.00079363
ENSG00000171444	-1.2540547	5.66E-05
ENSG00000171488	1.2227468	0.00012205
ENSG00000171617	-1.2733907	0.00295245
ENSG00000171843	-1.3197056	0.00055247
ENSG00000172031	-1.4325768	0.00732429
ENSG00000172086	1.28518802	0.00749951
ENSG00000172572	1.350335	5.72E-05
ENSG00000173166	1.20420718	0.01124859
ENSG00000173193	-1.3806804	0.00690258
ENSG00000173511	-1.2582417	9.22E-07
ENSG00000173535	-1.4796486	0.00011867
ENSG00000173597	1.33919353	0.00417445
ENSG00000173599	-1.2307607	0.00812736
ENSG00000174059	-1.8256611	0.00053458
ENSG00000174456	-1.2856022	0.02208598
ENSG00000174501	1.63837203	9.23E-05
ENSG00000174516	-1.289314	0.04115278

Gene ID	FC 8NG-24NG	adj p-value 8NG-24NG
ENSG00000174684	-1.2323344	0.00479239
ENSG00000174738	-1.2022915	0.03968118
ENSG00000175274	-1.2185889	0.00012327
ENSG00000175348	1.21033701	1.75E-05
ENSG00000175471	-1.2161161	0.0108976
ENSG00000175556	-1.3348542	0.00595423
ENSG00000176046	-1.4818612	0.00465559
ENSG00000176102	1.25397366	0.01584135
ENSG00000176170	-1.2349174	0.00027804
ENSG00000176371	-1.3212683	0.0001425
ENSG00000176438	1.4667385	3.25E-08
ENSG00000176658	1.25029048	2.41E-07
ENSG00000176907	-1.3375934	0.0073548
ENSG00000177119	1.25066673	2.63E-08
ENSG00000177169	-1.2844969	9.38E-06
ENSG00000177303	1.25739302	0.00655363
ENSG00000177663	1.21353499	0.00097421
ENSG00000177675	-1.4391906	0.00136057
ENSG00000178026	-1.2245167	0.00063514
ENSG00000178409	-1.2352682	0.02497416
ENSG00000178445	-1.2133451	0.01446249
ENSG00000178685	-1.2458994	0.01088893
ENSG00000178922	-1.380859	1.14E-07
ENSG00000179044	1.73214346	3.53E-06
ENSG00000179242	-1.3040697	0.01485976
ENSG00000179314	-1.3181582	1.42E-06
ENSG00000179403	1.33682062	0.00108238
ENSG00000179532	1.21994971	0.00082155
ENSG00000179598	1.23620637	0.00519246
ENSG00000179820	1.36622686	2.65E-10
ENSG00000180304	-1.2395257	8.63E-06
ENSG00000180448	1.31862074	0.02798362

Gene ID	FC 8NG-24NG	adj p-value 8NG-24NG
ENSG00000180573	-1.2373466	0.0031937
ENSG00000180596	-1.2113419	0.00981631
ENSG00000180758	1.21108975	9.60E-05
ENSG00000181634	-2.1685963	4.26E-05
ENSG00000181826	-1.2132312	1.61E-08
ENSG00000182013	-1.3840918	0.00520912
ENSG00000182568	-1.7057874	0.00125432
ENSG00000182621	1.49743037	5.8658E-05
ENSG00000182809	1.38937926	6.55E-09
ENSG00000182916	-1.3729921	0.0152969
ENSG00000183049	1.45855559	0.02185917
ENSG00000183496	-1.626927	9.40E-07
ENSG00000183578	1.22217648	0.00388132
ENSG00000183580	-1.3442679	4.03E-12
ENSG00000183598	-1.3617131	0.00022055
ENSG00000184113	-1.5784021	2.11E-08
ENSG00000184260	-1.2269558	0.00299288
ENSG00000184270	-1.2729042	0.00035393
ENSG00000184292	-1.3050028	0.04135375
ENSG00000184349	1.24546723	0.03725664
ENSG00000184357	-1.3584299	0.00063759
ENSG00000184371	-1.4816454	8.15E-07
ENSG00000184384	1.21028576	0.00014032
ENSG00000184584	1.34662242	0.00172661
ENSG00000184719	-1.2144842	0.00010629
ENSG00000184897	-1.2737315	1.87E-07
ENSG00000184916	1.31339545	3.71E-06
ENSG00000184988	1.20852616	0.01810529
ENSG00000185070	-1.3903388	0.0003359
ENSG00000185130	-1.3844751	0.00023069
ENSG00000185352	1.76288129	1.26E-07
ENSG00000185432	-1.4244741	3.67E-09

Gene ID	FC 8NG-24NG	adj p-value 8NG-24NG
ENSG00000185477	-1.4343023	0.00015167
ENSG00000185630	-1.5468543	1.35E-05
ENSG00000185753	1.25156902	0.03046232
ENSG00000185813	1.26765681	0.00387168
ENSG00000185909	-1.2520418	0.00044162
ENSG00000186470	-1.26458	0.00017493
ENSG00000186479	-1.3851189	7.70E-05
ENSG00000186567	1.51515867	7.04E-05
ENSG00000186642	-1.3075228	0.00386431
ENSG00000187239	-1.2207076	0.00046735
ENSG00000187479	-1.3327408	0.00716179
ENSG00000187678	1.29546121	0.00043146
ENSG00000187837	-1.334618	0.00150823
ENSG00000188191	-1.2104683	0.00743847
ENSG00000188428	1.20604134	0.00069766
ENSG00000188596	1.41052129	0.00011528
ENSG00000189056	1.3386962	1.50E-06
ENSG00000189060	-1.3386672	0.00025602
ENSG00000196169	-1.5835922	1.21E-07
ENSG00000196209	-1.3600111	3.48E-05
ENSG00000196352	1.27615755	1.8958E-05
ENSG00000196476	-1.2384617	0.00026428
ENSG00000196611	1.74065124	5.35E-06
ENSG00000196730	-1.2126358	2.46E-06
ENSG00000196747	-1.2429183	0.00022369
ENSG00000196787	-1.2614171	0.00257446
ENSG00000196866	-1.3119365	0.00079851
ENSG00000196878	-1.384302	0.00031568
ENSG00000197061	-1.2542094	0.00217784
ENSG00000197093	-1.2797822	0.00255877
ENSG00000197147	1.2960925	0.00136825
ENSG00000197153	-1.3631456	0.01316232

Gene ID	FC 8NG-24NG	adj p-value 8NG-24NG
ENSG00000197238	-1.2406383	0.0005684
ENSG00000197261	-1.3812093	0.00039396
ENSG00000197409	-1.3451359	0.00137582
ENSG00000197461	1.35825128	0.00063882
ENSG00000197555	-1.310976	8.17E-07
ENSG00000197780	1.21034251	0.01551086
ENSG00000197837	-1.3137802	1.21E-06
ENSG00000198108	1.42584182	0.00727093
ENSG00000198720	-1.2896531	1.32E-07
ENSG00000198721	-1.2502724	2.27E-08
ENSG00000198794	-1.3780748	1.54E-06
ENSG00000198795	-1.2339325	2.9126E-05
ENSG00000198816	-1.2224837	0.00329181
ENSG00000198844	1.3112094	0.00066681
ENSG00000198947	-1.4186928	0.00070013
ENSG00000198959	-1.5776561	1.34E-13
ENSG00000203814	-1.347438	1.64E-06
ENSG00000204217	1.25368851	0.00010244
ENSG00000204219	-1.2375255	0.03584436
ENSG00000204262	-1.2448093	0.00151069
ENSG00000204403	-1.2196413	0.02142904
ENSG00000204442	1.70894391	2.19E-06
ENSG00000204634	1.24374501	0.00811673
ENSG00000204776	1.38488247	0.00187536
ENSG00000205213	-1.2299409	0.01914984
ENSG00000205476	-1.2321583	0.00031524
ENSG00000205978	-1.3736965	0.00035041
ENSG00000220785	-1.2350092	0.00448983
ENSG00000221926	-1.2575239	0.00341931
ENSG00000224982	-1.3654069	0.03763625
ENSG00000225177	-1.3059127	0.00155077
ENSG00000225830	1.24645791	0.00021215

Gene ID	FC 8NG-24NG	adj p-value 8NG-24NG
ENSG00000226808	1.34366363	0.00071268
ENSG00000230266	-1.3580644	0.00537024
ENSG00000232931	1.94452128	6.71E-06
ENSG00000233117	1.88704303	2.78E-10
ENSG00000233251	1.23486618	0.02354768
ENSG00000233369	-1.2629181	0.00044162
ENSG00000233695	-1.2166277	0.02937725
ENSG00000233822	-1.2377087	0.03073122
ENSG00000235109	-1.3442886	0.00020773
ENSG00000235750	1.31600375	0.00024074
ENSG00000239521	-1.3590149	0.00934219
ENSG00000239887	-1.2744004	1.20E-06
ENSG00000242265	1.37000979	6.73E-09
ENSG00000243364	-1.2009337	0.02791495
ENSG00000249464	1.35510097	0.00209128
ENSG00000253368	1.70757355	3.66E-12
ENSG00000253522	-1.2523883	0.01268575
ENSG00000253552	-1.3779385	0.01263766
ENSG00000253661	1.24701358	0.00760522
ENSG00000254726	-1.3949372	4.29E-08
ENSG00000254858	1.22839727	0.01262691
ENSG00000255690	-1.3003114	0.04614297
ENSG00000257219	1.20236219	0.01225222
ENSG00000258441	1.46539544	5.5473E-05
ENSG00000260804	1.24131386	7.61E-05
ENSG00000262001	-1.3342428	0.04482034
ENSG00000267583	-1.2506948	0.04384988
ENSG00000268205	1.21889501	0.0055832
ENSG00000269821	1.26005014	0.02184373
ENSG00000270547	-1.7470708	0.00025775
ENSG00000272168	-1.3438405	0.03295007
ENSG00000273703	-1.4609275	0.00036902

Gene ID	FC 8NG-24NG	adj p-value 8NG-24NG
ENSG00000273802	-1.3628146	0.00124187
ENSG00000273983	-1.2739463	0.00045727
ENSG00000274180	-1.439139	5.76E-08
ENSG00000274267	-1.289086	0.00638869
ENSG00000274290	-1.4133097	4.58E-06
ENSG00000274618	-1.2727113	0.00644986
ENSG00000274641	-1.315533	0.0122545
ENSG00000274750	-1.3588837	0.00265633
ENSG00000274997	-1.3516139	0.00094388
ENSG00000275126	-1.24646	0.01187283
ENSG00000275379	-1.4434341	0.00103748
ENSG00000275714	-1.3386487	0.00027318
ENSG00000276180	-1.3658871	0.01188113
ENSG00000276368	-1.2888198	0.00289616
ENSG00000276410	-1.3907889	0.00334836
ENSG00000276903	-1.3270751	0.00205411
ENSG00000276966	-1.2032719	0.00237902
ENSG00000277075	-1.3530701	0.00085441
ENSG00000277157	-1.3387929	0.00492149
ENSG00000277224	-1.4250356	0.00064959
ENSG00000277775	-1.2828898	0.0006564
ENSG00000278272	-1.2743109	0.00346367
ENSG00000278463	-1.3292602	0.00415713
ENSG00000278588	-1.4348278	0.00018932
ENSG00000278637	-1.2443791	0.00015782
ENSG00000278705	-1.2920539	0.00069652
ENSG00000278828	-1.3000995	0.00085521
ENSG00000279095	1.22005184	0.00012778
ENSG00000279117	-1.2837723	0.00016737
ENSG00000280347	1.28712345	0.04084241
ENSG00000282057	1.2278835	0.00216206

Supplementary Table 3a. Differentially expressed genes between 0hr normal glucose and 0.5hr high glucose samples

Ensembl gene IDs, Fold change (baseline-0.5HG) and FDR adjusted p-values of DEGs

Gene ID	FC 0NG - 0.5HG	adj p-value 0NG-0.5HG
ENSG00000062716	1.30033064	0.02374954
ENSG00000067191	-1.2697759	0.02937725
ENSG00000073756	4.22680802	2.34E-08
ENSG00000078401	1.47208593	3.90E-08
ENSG00000087074	1.29784451	7.16E-07
ENSG00000099860	1.91657981	7.09E-09
ENSG00000100906	1.30421767	0.00035118
ENSG00000108551	1.34252794	0.00055223
ENSG00000113070	1.48214049	4.05E-05
ENSG00000113240	-1.4080374	0.00129352
ENSG00000114315	3.48272909	1.40E-10
ENSG00000114670	1.40876205	0.00216221
ENSG00000115738	2.02891885	2.17E-05
ENSG00000115963	1.2204263	0.02764555
ENSG00000116285	1.31384995	0.00053011
ENSG00000117318	1.96787192	9.76E-14
ENSG00000117525	1.59680244	0.00016331
ENSG00000118515	1.70088993	2.34E-18
ENSG00000119669	1.2173636	0.01707697
ENSG00000120129	2.10030013	1.65E-12
ENSG00000120738	16.3119895	6.05E-18
ENSG00000122877	3.14593508	1.67E-09
ENSG00000123358	3.66742084	6.14E-11
ENSG00000124216	2.81480682	8.17E-16
ENSG00000124575	-1.2365918	0.02554629
ENSG00000124635	-1.3073154	0.02297748
ENSG00000125740	3.31249732	4.91E-06
ENSG00000125968	3.4319867	1.51E-13
ENSG00000127528	1.29888913	0.016211
ENSG00000128016	3.70065348	1.99E-15
ENSG00000128594	1.88548032	3.93E-06
ENSG00000130513	1.264649	4.0208E-05

Gene ID	FC 0NG - 0.5HG	adj p-value 0NG-0.5HG
ENSG00000132510	1.29896825	0.0005348
ENSG00000134107	1.29469349	6.16E-05
ENSG00000136158	1.6113021	4.02E-08
ENSG00000136826	2.19656787	4.10E-11
ENSG00000137700	-1.2548241	0.03428735
ENSG00000137834	2.05588565	3.06E-05
ENSG00000138166	1.68887213	6.18E-09
ENSG00000139318	1.26295705	2.25E-05
ENSG00000142871	1.3728119	1.94E-07
ENSG00000143384	1.2304046	8.20E-06
ENSG00000144655	1.70611904	1.29E-11
ENSG00000144802	1.74839689	0.00038122
ENSG00000155090	1.82812094	1.60E-08
ENSG00000158050	1.48425585	0.00012754
ENSG00000160888	2.15765749	5.57E-18
ENSG00000162772	2.96671004	1.0313E-05
ENSG00000163660	1.23772772	0.02117355
ENSG00000163739	1.47822349	0.00143832
ENSG00000164683	2.15021442	6.46E-08
ENSG00000167470	1.33956938	4.00E-07
ENSG00000167565	1.30345266	0.00011319
ENSG00000167625	-1.2059088	0.00515502
ENSG00000168298	-1.2069405	0.02644731
ENSG00000169429	1.75304271	0.00911922
ENSG00000169914	1.21160032	0.02790906
ENSG00000170345	8.8320477	7.38E-18
ENSG00000171223	3.44250372	1.67E-22
ENSG00000173334	1.53931986	7.72E-05
ENSG00000176907	2.30395096	6.44E-09
ENSG00000177606	1.31479934	2.92E-08
ENSG00000177842	1.26038873	0.03177645
ENSG00000179388	5.6881191	9.77E-12

Gene ID	FC 0NG - 0.5HG	adj p-value 0NG-0.5HG
ENSG00000183598	-1.2667436	0.00730875
ENSG00000183691	1.50357803	0.00203401
ENSG00000184557	1.99144938	6.94E-10
ENSG00000184678	-1.2351195	0.01575509
ENSG00000185022	1.22868966	0.00090814
ENSG00000187479	1.36119686	0.02192028
ENSG00000187678	1.31656658	5.58E-05
ENSG00000187837	-1.2102891	0.04097856
ENSG00000196449	1.24898956	2.49E-05
ENSG00000196866	-1.2936379	0.00307213
ENSG00000196890	-1.2463068	0.04218923
ENSG00000197019	1.55554014	2.15E-09
ENSG00000198185	-1.266913	0.04796462
ENSG00000198189	1.22591764	0.02019484
ENSG00000198435	1.37409085	0.00122418
ENSG00000198517	1.21774753	0.0019382
ENSG00000198576	2.05114328	5.46E-05
ENSG00000203804	1.46119874	0.0008254
ENSG00000207808	1.25996988	0.00822851
ENSG00000207980	1.42363399	0.00618469
ENSG00000218792	1.27056547	0.00492004
ENSG00000228742	1.35498819	0.00684392
ENSG00000231856	1.3895325	0.00066254
ENSG00000237188	1.22435698	0.01260007
ENSG00000237742	1.20759383	0.00940587
ENSG00000241990	-1.282062	0.01127297
ENSG00000250539	-1.2305935	0.02480238
ENSG00000253669	1.25705215	0.01361225
ENSG00000254991	-1.2100323	0.01242897
ENSG00000255112	1.21545198	0.0001426
ENSG00000257298	1.20520844	0.01881927
ENSG00000260996	1.3921402	0.00364147

Gene ID	FC 0NG - 0.5HG	adj p-value 0NG-0.5HG
ENSG00000262074	1.29475617	0.0124195
ENSG00000264395	1.26432237	0.00021353
ENSG00000267519	2.16507687	1.65E-06
ENSG00000267598	1.84085337	0.0004022
ENSG00000269609	-1.2614619	0.02294495
ENSG00000272463	-1.21734	0.00604265
ENSG00000273802	-1.2439866	0.0244524
ENSG00000273983	-1.2852092	0.02085395
ENSG00000274026	-1.3089515	0.0072983
ENSG00000275202	1.2942687	0.02992529
ENSG00000275221	-1.2888885	0.04406039
ENSG00000275379	-1.2785433	0.03125426
ENSG00000275993	1.46133363	0.00032509
ENSG00000276180	-1.3070832	0.03324367
ENSG00000276603	-1.2669635	0.03709508
ENSG00000276849	-1.3966242	0.0400342
ENSG00000277224	-1.2926375	0.01635922
ENSG00000277639	-1.2533234	0.02784903
ENSG00000278272	-1.3120047	0.01165235
ENSG00000278463	-1.3465699	0.00323913
ENSG00000278828	-1.2408593	0.04534968
ENSG00000279672	-1.2973946	0.02934262
ENSG00000280152	-1.3956226	0.00253797
ENSG00000281614	1.25140042	0.02203304
ENSG00000281881	1.45384	0.02480742

Supplementary Table 3b. Differentially expressed genes between 0hr normal glucose and 1hr high glucose samples

Ensembl gene IDs, Fold change (baseline-1HG) and FDR adjusted p-values of DEGs

Gene ID	FC 0NG - 1HG	adj p-value 0NG-1HG
ENSG00000002016	-1.2776588	0.02460043
ENSG00000005238	1.26910297	6.11E-07
ENSG00000005513	3.0222181	4.36E-14
ENSG00000006327	1.26679022	2.77E-06
ENSG00000006459	1.70325027	2.92E-09
ENSG00000011422	1.21127329	0.00278748
ENSG00000015568	1.36414525	0.00386415
ENSG00000019144	1.30724941	2.58E-07
ENSG00000019549	1.72231925	2.01E-07
ENSG00000023445	1.83590694	0.00028217
ENSG00000023902	1.31345763	1.16E-05
ENSG00000041988	-1.2165628	0.00614946
ENSG00000047346	1.2964436	0.01626176
ENSG00000049130	1.43869071	0.03310766
ENSG00000053524	1.29174214	0.02315752
ENSG00000054598	1.78633086	9.14E-11
ENSG00000054967	1.7638298	3.61E-09
ENSG00000059728	1.48846888	8.88E-08
ENSG00000066923	-1.2072958	0.01079321
ENSG00000069399	1.36561994	7.23E-05
ENSG00000069667	1.38983103	0.00539096
ENSG00000070404	1.27366617	0.00094091
ENSG00000070610	1.41591889	0.00023339
ENSG00000073756	9.93733459	5.85E-15
ENSG00000075426	1.66392652	2.65E-13
ENSG00000078401	1.35089114	1.74E-06
ENSG00000079337	1.38450251	0.00020624
ENSG00000080947	-1.2624166	0.04729036
ENSG00000081386	-1.3032054	7.51E-06
ENSG00000084093	1.50666938	7.55E-09
ENSG00000085274	1.25255842	0.00386496
ENSG00000087074	1.28877355	2.46E-07

Gene ID	FC 0NG - 1HG	adj p-value 0NG-1HG
ENSG00000087448	1.23742952	0.00019129
ENSG00000087494	1.75295026	3.08E-06
ENSG00000088826	1.36289968	1.30E-06
ENSG00000088876	-1.2317023	0.00634889
ENSG00000089335	-1.2151769	0.02952021
ENSG00000090339	1.36943206	0.03118624
ENSG00000095739	1.33820636	0.00110198
ENSG00000095752	1.96918901	9.57E-08
ENSG00000096717	1.25805491	0.00443624
ENSG00000097096	1.37509976	0.00214384
ENSG00000099860	3.19572393	3.11E-17
ENSG00000100276	1.27987202	0.00517288
ENSG00000100292	1.25340702	0.00209161
ENSG00000100311	1.41340111	0.00069311
ENSG00000100906	2.11446649	7.10E-16
ENSG00000101384	1.49444108	7.35E-07
ENSG00000101665	10.8010839	2.19E-21
ENSG00000102034	1.2151635	0.00014345
ENSG00000102890	1.65809767	7.63E-05
ENSG00000103241	1.29522478	0.03834984
ENSG00000103257	1.37438731	4.20E-08
ENSG00000104081	1.48861031	0.03576979
ENSG00000104419	1.2543182	7.16E-06
ENSG00000104903	1.21098714	0.02147477
ENSG00000105072	-1.2510307	0.0078977
ENSG00000105443	1.20915889	0.00661989
ENSG00000105656	1.23114417	0.00026069
ENSG00000105722	1.60332879	5.81E-11
ENSG00000105991	-1.2104933	0.04622144
ENSG00000106003	2.45456276	1.43E-10
ENSG00000106089	1.85246743	6.23E-09
ENSG00000106133	-1.3393052	0.00660177

Gene ID	FC 0NG - 1HG	adj p-value 0NG-1HG
ENSG00000106799	1.22785102	0.01323065
ENSG00000107562	1.23407563	0.02191463
ENSG00000107731	1.33084145	0.01337258
ENSG00000107807	1.5032104	0.00012782
ENSG00000107821	1.93387344	9.37E-07
ENSG00000107968	1.54241068	0.00097329
ENSG00000108551	1.69295274	1.20E-09
ENSG00000109787	1.397137	8.48E-11
ENSG00000110330	1.44018017	1.21E-05
ENSG00000110888	1.26933222	0.00278197
ENSG00000111224	1.22486355	0.0112354
ENSG00000111276	-1.393445	9.42E-07
ENSG00000111859	1.76341152	4.23E-08
ENSG00000111860	1.2712682	0.00200321
ENSG00000112406	1.24372782	0.00550368
ENSG00000112658	1.23632402	1.26E-08
ENSG00000112715	2.35471941	2.32E-12
ENSG00000113070	1.99377474	2.01E-11
ENSG00000113369	-1.6805316	2.09E-09
ENSG00000113742	1.44703095	1.49E-08
ENSG00000114019	1.65296316	8.14E-13
ENSG00000114315	7.70751206	3.73E-18
ENSG00000114529	1.4893161	1.97E-05
ENSG00000114739	-1.2782336	0.00652702
ENSG00000115255	-1.2094587	0.04679523
ENSG00000115325	-1.2170122	0.01664271
ENSG00000115520	1.34132518	0.00041046
ENSG00000115548	1.38631949	5.89E-09
ENSG00000115738	5.92107644	1.02E-16
ENSG00000115844	2.76176833	6.47E-15
ENSG00000115963	1.27645172	0.00181183
ENSG00000116017	1.2788837	0.02059827

Gene ID	FC 0NG - 1HG	adj p-value 0NG-1HG
ENSG00000116205	-1.2037112	0.01306962
ENSG00000116285	2.14711211	2.25E-15
ENSG00000116604	1.31012741	1.15E-05
ENSG00000116678	1.26091238	0.04276259
ENSG00000116717	-1.482501	4.63E-10
ENSG00000116731	1.27359088	1.11E-07
ENSG00000116741	-1.2912329	0.00514312
ENSG00000117036	1.23494994	1.07E-06
ENSG00000117143	1.2482612	5.61E-06
ENSG00000117318	3.20620087	1.67E-22
ENSG00000117479	1.38039555	0.00020733
ENSG00000117525	2.02640194	9.76E-09
ENSG00000118263	1.45999511	1.60E-09
ENSG00000118515	2.47452115	2.12E-27
ENSG00000118523	1.31945285	1.51E-10
ENSG00000119508	3.41449526	1.57E-11
ENSG00000119669	2.00976218	2.71E-14
ENSG00000120129	2.44467601	4.55E-16
ENSG00000120279	1.22758613	0.00135186
ENSG00000120616	1.29442932	4.02E-06
ENSG00000120696	-1.3290733	0.01963479
ENSG00000120738	12.4575292	1.35E-17
ENSG00000120784	-1.3428765	0.00101332
ENSG00000121417	-1.2617669	0.00729111
ENSG00000121966	-1.3818904	1.9748E-05
ENSG00000122386	-1.2761988	0.00329041
ENSG00000122644	-1.2172563	0.02768971
ENSG00000122863	1.71996721	1.99E-10
ENSG00000122877	4.59154911	5.84E-14
ENSG00000122971	-1.2056916	0.01658671
ENSG00000123095	1.50724202	0.0003531
ENSG00000123358	9.25706336	3.02E-19

Gene ID	FC 0NG - 1HG	adj p-value 0NG-1HG
ENSG00000123395	1.21169464	1.14E-05
ENSG00000124019	-1.3482741	2.39E-05
ENSG00000124193	1.20248816	2.84E-06
ENSG00000124216	6.79133115	6.88E-26
ENSG00000124575	-1.3720086	0.00010467
ENSG00000124610	-1.2039948	0.04440501
ENSG00000124762	1.36027917	4.83E-14
ENSG00000125266	1.34244005	0.00032653
ENSG00000125378	-1.334411	0.0016233
ENSG00000125740	6.53793221	1.75E-11
ENSG00000125812	1.41915457	6.80E-09
ENSG00000125845	1.26161033	0.01229972
ENSG00000125898	1.20642724	0.04587694
ENSG00000125945	-1.3310823	6.02E-06
ENSG00000125968	6.84800219	6.92E-21
ENSG00000126368	-1.4672031	5.22E-05
ENSG00000126603	1.3571133	7.99E-05
ENSG00000126778	1.31694562	0.00959733
ENSG00000127080	1.54749506	1.26E-11
ENSG00000127528	1.4531538	8.26E-05
ENSG00000128016	2.59833958	3.06E-12
ENSG00000128283	1.2775576	5.83E-07
ENSG00000128342	1.36540866	0.0029862
ENSG00000128594	6.95668814	7.73E-21
ENSG00000128917	1.3704476	0.00618923
ENSG00000129355	-1.2470773	0.03515754
ENSG00000129467	1.24220388	0.0063348
ENSG00000129514	1.2021233	0.01800495
ENSG00000130164	1.24258911	0.00116799
ENSG00000130222	1.61580837	1.67E-06
ENSG00000130513	1.61688741	2.12E-13
ENSG00000131016	1.20257327	8.3619E-05

Gene ID	FC 0NG - 1HG	adj p-value 0NG-1HG
ENSG00000131759	1.44031582	1.57E-05
ENSG00000132326	1.4501131	0.00010522
ENSG00000132510	2.7306988	7.85E-20
ENSG00000132825	-1.2100288	0.01076428
ENSG00000133561	-1.3105737	1.31E-07
ENSG00000133808	1.31101766	0.02547698
ENSG00000133985	1.312725	0.00091265
ENSG00000134107	2.54229662	4.84E-21
ENSG00000134222	-1.3056155	0.00825474
ENSG00000134461	-1.2521645	0.00467922
ENSG00000134531	1.25763671	5.02E-13
ENSG00000134668	1.36677234	9.74E-05
ENSG00000135547	2.75687166	9.85E-11
ENSG00000135625	1.49843889	7.74E-07
ENSG00000136059	-1.2379201	0.02570757
ENSG00000136158	1.92857606	1.10E-12
ENSG00000136603	1.98991245	2.81E-12
ENSG00000136826	4.15200555	2.94E-20
ENSG00000137449	1.22157414	0.01050241
ENSG00000137700	-1.2240135	0.04490516
ENSG00000137834	5.75064312	5.93E-16
ENSG00000138134	1.22724868	0.0343168
ENSG00000138166	2.78847404	1.37E-18
ENSG00000138623	1.90304623	3.07E-06
ENSG00000138675	1.47457397	7.22E-05
ENSG00000138835	1.46837026	2.26E-06
ENSG00000139263	-1.2138299	0.0004376
ENSG00000139278	1.26390171	0.00011744
ENSG00000139517	1.21926514	0.00091265
ENSG00000140416	1.26486964	8.59E-09
ENSG00000140534	-1.2620437	0.01732389
ENSG00000140691	1.22944624	0.00200701

Gene ID	FC 0NG - 1HG	adj p-value 0NG-1HG
ENSG00000141034	-1.2012803	9.95E-05
ENSG00000141526	1.30852353	7.05E-08
ENSG00000141582	1.25297736	0.01169044
ENSG00000141858	1.23282056	0.00011229
ENSG00000142178	1.65316169	2.09E-06
ENSG00000142279	1.45720639	4.43E-07
ENSG00000142396	1.34287816	0.04692777
ENSG00000142408	1.42146952	2.22E-05
ENSG00000142627	1.32983229	8.89E-08
ENSG00000142871	1.4878183	1.12E-10
ENSG00000143067	1.71133067	4.80E-09
ENSG00000143322	1.29893036	1.70E-08
ENSG00000143367	1.44622637	8.67E-06
ENSG00000143384	1.50687572	3.88E-14
ENSG00000143479	1.30150075	0.00090724
ENSG00000143622	1.239355	1.39E-05
ENSG00000143751	1.25473922	1.76E-05
ENSG00000143878	1.53829451	7.75E-07
ENSG00000144026	-1.2226672	0.01276475
ENSG00000144120	-1.203228	0.02249501
ENSG00000144136	1.20593978	3.91E-05
ENSG00000144560	1.22439714	9.63E-06
ENSG00000144566	1.21953794	5.7264E-05
ENSG00000144655	3.67572748	3.16E-25
ENSG00000145632	-2.1839404	1.38E-16
ENSG00000145675	1.20522801	0.0169184
ENSG00000146232	1.97121173	9.95E-16
ENSG00000146426	1.21159971	0.00162342
ENSG00000146676	1.35058309	0.00049113
ENSG00000148339	1.49001201	4.19E-07
ENSG00000148677	1.56766079	2.30E-11
ENSG00000148841	1.4387436	3.12E-12

Gene ID	FC 0NG - 1HG	adj p-value 0NG-1HG
ENSG00000148926	-1.6254343	1.20E-06
ENSG00000149289	1.47221947	6.46E-08
ENSG00000149591	1.2158668	0.02146619
ENSG00000149646	1.32263826	0.01056523
ENSG00000150907	1.20375069	3.03E-05
ENSG00000151014	1.39804912	1.34E-10
ENSG00000151458	1.2140273	0.00818423
ENSG00000151748	1.36343947	8.30E-07
ENSG00000151967	1.28905534	0.0157049
ENSG00000152433	-1.2303356	0.03792699
ENSG00000153094	1.31357709	0.02860786
ENSG00000153234	1.99912421	0.00623797
ENSG00000153879	1.21525101	0.00255385
ENSG00000153885	1.47295357	3.49E-10
ENSG00000155090	3.46660327	8.85E-19
ENSG00000155760	1.41137524	1.61E-07
ENSG00000156030	1.33303584	2.40E-06
ENSG00000156273	1.74420972	4.47E-12
ENSG00000156521	-1.2344193	0.01456421
ENSG00000156966	1.25030244	0.03876943
ENSG00000156983	1.27103504	4.31E-07
ENSG00000157240	1.50432994	1.69E-05
ENSG00000157514	-1.3149667	0.00067343
ENSG00000158050	1.38663816	0.00064604
ENSG00000158615	1.26238252	1.07E-09
ENSG00000158859	1.39491243	2.98E-06
ENSG00000159216	1.24010226	0.00103313
ENSG00000159261	1.31281935	0.00955653
ENSG00000159388	-1.2719551	7.10E-06
ENSG00000160201	1.35902685	0.00291321
ENSG00000160888	2.74601301	5.38E-23
ENSG00000161642	1.22607062	0.00212054

Gene ID	FC 0NG - 1HG	adj p-value 0NG-1HG
ENSG00000161653	-1.3031241	0.01156381
ENSG00000161835	2.00855448	1.56E-14
ENSG00000161940	1.34268513	2.28E-09
ENSG00000162063	-1.2162361	0.03094216
ENSG00000162407	1.36432031	0.00252728
ENSG00000162413	1.49939747	8.25E-12
ENSG00000162702	1.3323412	2.08E-06
ENSG00000162757	-1.3313842	0.00168068
ENSG00000162772	3.44830642	1.53E-07
ENSG00000162852	1.24066101	0.00237665
ENSG00000163026	-1.2001418	0.00342048
ENSG00000163155	-1.3453174	0.00502012
ENSG00000163293	1.35034577	0.02645902
ENSG00000163376	1.3652602	0.01195523
ENSG00000163462	-1.2552762	0.03161445
ENSG00000163545	1.92750988	3.99E-07
ENSG00000163596	-1.2485023	0.0422484
ENSG00000163635	1.22114521	5.81E-07
ENSG00000163659	1.51099429	8.09E-15
ENSG00000163660	1.38927621	4.02E-05
ENSG00000163739	1.2865429	0.04338694
ENSG00000163874	2.24704497	3.08E-11
ENSG00000164048	-1.2286548	0.00081121
ENSG00000164056	-1.8799176	5.87E-07
ENSG00000164683	8.40714956	2.97E-22
ENSG00000164736	1.21170181	0.00066255
ENSG00000164741	1.24774011	2.28E-07
ENSG00000164855	1.24201096	0.0329814
ENSG00000165030	1.22376081	0.02853731
ENSG00000165244	1.24342621	0.027387
ENSG00000165507	-3.0941987	1.42E-11
ENSG00000165572	-1.2957765	1.71E-06

Gene ID	FC 0NG - 1HG	adj p-value 0NG-1HG
ENSG00000165655	1.58439809	3.30E-06
ENSG00000166289	-1.5567719	7.10E-08
ENSG00000166401	1.34809339	1.36E-10
ENSG00000166471	1.23959378	0.00571931
ENSG00000166483	1.43105831	1.02E-05
ENSG00000166592	1.23023245	0.027062
ENSG00000166704	-1.2730625	0.00480882
ENSG00000166860	1.22819905	0.0002102
ENSG00000166886	2.81376362	3.53E-17
ENSG00000166900	1.21447349	8.25E-06
ENSG00000167077	-1.2420469	0.01606985
ENSG00000167470	2.35473481	7.67E-22
ENSG00000167550	-1.2131075	0.0489937
ENSG00000167565	1.73277713	9.54E-13
ENSG00000167625	-1.379415	2.47E-07
ENSG00000167657	1.22418431	1.42E-06
ENSG00000167772	1.78230548	3.05E-07
ENSG00000167874	1.2331303	0.04206253
ENSG00000168209	1.46636	0.00105501
ENSG00000168298	-1.2024596	0.01672738
ENSG00000168386	1.51822315	2.23E-05
ENSG00000168672	-1.2020876	0.02292807
ENSG00000168795	1.28060651	0.00246731
ENSG00000168874	2.17732059	1.05E-10
ENSG00000168994	1.32741599	2.62E-08
ENSG00000169016	1.30900912	7.2858E-05
ENSG00000169155	1.21748047	0.00226864
ENSG00000169242	1.50504655	0.00091643
ENSG00000169429	1.67707128	0.00957216
ENSG00000169641	1.2138276	3.56E-10
ENSG00000169914	1.26920724	0.00156047
ENSG00000169926	1.26202198	0.00015167

Gene ID	FC 0NG - 1HG	adj p-value 0NG-1HG
ENSG00000169991	1.28611248	2.26E-06
ENSG00000170345	3.29541795	7.36E-11
ENSG00000170385	1.29154823	0.0272459
ENSG00000170525	1.3724543	2.72E-11
ENSG00000170775	1.21502362	0.00049816
ENSG00000170852	1.51717104	1.04E-11
ENSG00000171115	-1.2004156	0.00039869
ENSG00000171223	6.56027774	8.40E-30
ENSG00000171246	1.97184814	8.78E-08
ENSG00000171604	1.74223033	1.02E-06
ENSG00000171612	1.21168094	0.00456064
ENSG00000171617	1.51779738	2.02E-08
ENSG00000171621	1.53371791	0.0002849
ENSG00000171889	1.28033051	0.00809869
ENSG00000171988	1.21379547	0.00186591
ENSG00000172059	1.51856575	1.10E-06
ENSG00000172216	1.27080251	0.00055791
ENSG00000172602	1.293028	0.04181548
ENSG00000173013	1.41782226	0.00088883
ENSG00000173276	1.21768289	0.00254443
ENSG00000173281	1.46922539	3.74E-11
ENSG00000173334	1.36511572	0.00242891
ENSG00000173530	1.20329503	0.00028665
ENSG00000173846	1.33578988	6.08E-05
ENSG00000173894	-1.2197621	0.00680301
ENSG00000174738	1.20099057	0.01798388
ENSG00000175087	-1.2515505	0.02043226
ENSG00000175105	1.67538395	6.57E-05
ENSG00000175505	1.29582783	0.01011111
ENSG00000175592	1.66850865	6.89E-10
ENSG00000175606	1.26221876	0.00037173
ENSG00000175643	-1.2125039	0.04100438

Gene ID	FC 0NG - 1HG	adj p-value 0NG-1HG
ENSG00000175906	-1.6062854	9.37E-08
ENSG00000176170	1.39337024	1.35E-07
ENSG00000176371	-1.2310036	0.03537313
ENSG00000176692	1.97829514	1.81E-13
ENSG00000176714	-1.246358	0.0397037
ENSG00000176749	1.39234254	0.00021581
ENSG00000176842	1.31880963	0.00318699
ENSG00000176845	1.41747585	4.73E-05
ENSG00000176907	3.95870205	3.56E-16
ENSG00000177125	1.48198871	1.04E-08
ENSG00000177173	1.25507483	0.01768093
ENSG00000177283	1.33205622	1.40E-05
ENSG00000177337	-1.2474207	0.02152699
ENSG00000177426	-1.3704978	3.19E-08
ENSG00000177606	1.20242307	2.10E-05
ENSG00000177679	1.43780798	0.00037299
ENSG00000178996	1.21620774	5.28E-05
ENSG00000179094	1.22362758	0.00315073
ENSG00000179144	-1.4453657	5.35E-09
ENSG00000179348	2.07550138	1.39E-12
ENSG00000179361	1.32169758	0.00082459
ENSG00000179388	12.3392681	9.36E-18
ENSG00000179431	1.2344862	0.01403624
ENSG00000179604	1.23406677	0.0006696
ENSG00000179833	1.29557758	5.30E-07
ENSG00000179922	1.25395933	0.00766456
ENSG00000180257	-1.243655	0.02637974
ENSG00000180376	-1.2629593	0.01847551
ENSG00000180530	1.2537352	0.00102861
ENSG00000180592	1.52183978	2.4239E-05
ENSG00000180730	1.21066293	0.01516168
ENSG00000180787	-1.3191451	0.00310246

Gene ID	FC 0NG - 1HG	adj p-value 0NG-1HG
ENSG00000181007	-1.2586386	0.01081147
ENSG00000181274	-1.313756	0.00052372
ENSG00000181472	1.7813999	9.94E-10
ENSG00000181541	-1.3878825	0.00043628
ENSG00000181773	1.27131277	0.03299912
ENSG00000182310	1.25114248	0.04732504
ENSG00000183072	1.24457327	0.00748947
ENSG00000183161	-1.3133502	0.00151371
ENSG00000183307	1.37301646	0.00201013
ENSG00000183337	2.18156983	3.28E-11
ENSG00000183598	-1.2680751	0.00309522
ENSG00000183691	3.59636898	1.66E-15
ENSG00000183779	1.23779011	0.02215052
ENSG00000183935	1.20778104	0.00629612
ENSG00000184058	1.24944385	0.00454212
ENSG00000184270	-1.2701196	0.04071702
ENSG00000184307	1.78410287	8.86E-07
ENSG00000184357	-1.2356982	0.03168491
ENSG00000184402	1.29742434	3.48E-05
ENSG00000184557	3.31635212	3.55E-18
ENSG00000184588	-1.2034129	0.00058419
ENSG00000184898	-1.2564503	0.01041822
ENSG00000184916	1.25699897	9.90E-05
ENSG00000185015	1.32467687	0.00141517
ENSG00000185022	1.59558398	4.93E-12
ENSG00000185130	-1.2390954	0.04079461
ENSG00000185252	-1.2818092	0.0006354
ENSG00000185950	1.38013703	0.03964505
ENSG00000186480	1.41860862	0.00020161
ENSG00000187479	1.79380723	8.80E-07
ENSG00000187566	-1.2377762	0.00875472
ENSG00000187626	-1.5024359	9.87E-06

Gene ID	FC 0NG - 1HG	adj p-value 0NG-1HG
ENSG00000187678	2.43186755	1.72E-19
ENSG00000187837	-1.2076155	0.02546824
ENSG00000188290	1.2949678	0.00368324
ENSG00000188295	1.52366549	3.77E-06
ENSG00000188483	2.75705652	3.58E-21
ENSG00000188522	1.61468336	1.16E-09
ENSG00000188997	-1.3793488	1.71E-05
ENSG00000189298	-1.3163005	0.00694876
ENSG00000196150	-1.2867508	0.00089686
ENSG00000196152	-1.204167	0.00636974
ENSG00000196371	1.29660857	4.59E-07
ENSG00000196418	1.24609646	0.00318517
ENSG00000196428	1.488369	4.76E-13
ENSG00000196449	1.54365844	3.82E-13
ENSG00000196659	-1.222633	0.04950253
ENSG00000196670	-1.2981384	5.0365E-05
ENSG00000196843	1.76436041	4.44E-10
ENSG00000196866	-1.2519152	0.00580686
ENSG00000196890	-1.2662915	0.01346428
ENSG00000197019	2.75784021	1.46E-21
ENSG00000197050	-1.3439127	0.00060404
ENSG00000197063	1.31113117	5.85E-08
ENSG00000197153	-1.2817304	0.03604834
ENSG00000197183	1.30621034	2.50E-09
ENSG00000197238	-1.2325859	0.0054889
ENSG00000197329	1.32007204	0.00016727
ENSG00000197362	-1.2827458	0.03842975
ENSG00000197380	1.29499674	0.04807263
ENSG00000197409	-1.254441	0.0221443
ENSG00000197461	2.00103174	1.52E-13
ENSG00000197837	-1.2035556	0.00745938
ENSG00000198108	1.3237804	0.01585018

Gene ID	FC 0NG - 1HG	adj p-value 0NG-1HG
ENSG00000198142	1.44725822	2.96E-05
ENSG00000198182	-1.4649019	6.57E-06
ENSG00000198185	-1.2919783	0.01459207
ENSG00000198355	1.33264173	4.15E-06
ENSG00000198435	2.49696888	6.93E-15
ENSG00000198517	2.14558267	1.68E-18
ENSG00000198576	2.85589311	4.01E-09
ENSG00000198774	-1.3802131	0.00122316
ENSG00000198938	1.21708789	0.03338612
ENSG00000203709	-2.2010513	1.07E-08
ENSG00000203814	-1.2295851	0.00619607
ENSG00000203883	1.55766282	7.65E-05
ENSG00000204103	1.37845165	0.00088598
ENSG00000204442	1.22613325	0.02502588
ENSG00000204611	-1.286499	9.03E-06
ENSG00000205138	-1.2286579	0.00524624
ENSG00000205730	1.20046068	6.83E-08
ENSG00000207808	1.25006588	0.00539375
ENSG00000207980	1.5196382	0.00029849
ENSG00000213203	-1.4157793	3.07E-06
ENSG00000213928	-1.3039424	9.54E-05
ENSG00000215271	-1.2076684	0.02126645
ENSG00000215417	1.32643367	0.04386966
ENSG00000215458	-1.2716601	0.04665163
ENSG00000218891	1.22434588	0.01430695
ENSG00000222455	1.2775398	0.00194403
ENSG00000223749	-1.2830601	0.03399573
ENSG00000224531	1.21345882	0.00609946
ENSG00000224959	1.33504943	0.00273322
ENSG00000225614	1.26082866	2.15E-05
ENSG00000225630	1.26909383	0.02385712
ENSG00000227124	-1.2787348	0.0130604

Gene ID	FC 0NG - 1HG	adj p-value 0NG-1HG
ENSG00000228794	1.22545758	0.00871622
ENSG00000229729	1.51791204	6.14E-06
ENSG00000230183	1.29511897	0.00011721
ENSG00000230701	1.36575114	0.01240587
ENSG00000231856	1.21662096	0.04900493
ENSG00000232759	-1.2959546	0.0237492
ENSG00000233822	-1.2459265	0.02548885
ENSG00000235316	1.36961324	0.00976267
ENSG00000235831	1.21184296	0.01765958
ENSG00000236515	-1.2549297	0.01792834
ENSG00000237181	1.24977208	0.00966701
ENSG00000237892	1.22450944	0.04495065
ENSG00000241852	1.23751782	0.02115029
ENSG00000242829	1.30757673	0.00079201
ENSG00000243811	-1.2031616	0.04754298
ENSG00000250714	1.26265444	0.01530004
ENSG00000251493	1.56633244	0.00004907
ENSG00000253276	-1.2053986	0.00178999
ENSG00000253368	1.35030587	1.68E-07
ENSG00000253669	1.5325579	3.65E-07
ENSG00000253958	1.60022899	2.12E-07
ENSG00000254165	1.23584839	0.0006712
ENSG00000254470	1.30557064	3.96E-08
ENSG00000255112	1.43731774	3.45E-11
ENSG00000255176	2.05871056	2.38E-06
ENSG00000255517	-1.245996	0.02512179
ENSG00000256448	1.20930742	0.00038178
ENSG00000259330	1.30858405	0.0009862
ENSG00000260231	1.26532541	0.00602351
ENSG00000260996	1.57796984	1.09E-05
ENSG00000261556	-1.314659	0.03038221
ENSG00000262292	1.30358113	0.04393621

Gene ID	FC 0NG - 1HG	adj p-value 0NG-1HG
ENSG00000264395	1.2699159	4.71E-05
ENSG00000264635	1.25876803	0.00548713
ENSG00000265666	1.29903463	0.01560806
ENSG00000265972	-1.3654228	4.08E-05
ENSG00000265982	1.26147622	0.01197054
ENSG00000266236	1.20802019	0.0001886
ENSG00000267041	1.30838643	0.00898297
ENSG00000267519	2.70225115	8.29E-10
ENSG00000267598	2.24797478	7.20E-07
ENSG00000269318	1.27174314	0.0308841
ENSG00000269609	-1.2216376	0.03902451
ENSG00000269906	1.25482131	0.00446439
ENSG00000272086	1.25319148	0.02290383
ENSG00000272398	1.25593095	0.00432951
ENSG00000272574	1.33520281	0.00018921
ENSG00000272768	1.352707	0.00084517
ENSG00000273489	1.93582344	3.29E-07
ENSG00000273729	1.24992055	0.01690069
ENSG00000273802	-1.201083	0.04933109
ENSG00000273820	1.32085566	0.00530899
ENSG00000273888	-1.2556183	0.04294324
ENSG00000273983	-1.2905796	0.00916872
ENSG00000274290	-1.2450391	0.01068393
ENSG00000274349	-1.3533291	0.00233039
ENSG00000274618	-1.2561777	0.04987994
ENSG00000274636	1.64517799	0.00010119
ENSG00000274995	1.82719204	2.71E-06
ENSG00000275031	1.37792065	0.00069621
ENSG00000275111	-1.3479958	0.00067666
ENSG00000275126	-1.2490086	0.02128662
ENSG00000275202	1.48148864	0.00010142
ENSG00000275379	-1.2483399	0.03752958

Gene ID	FC 0NG - 1HG	adj p-value 0NG-1HG
ENSG00000275713	-1.3470035	0.00773188
ENSG00000275714	-1.2174714	0.00867911
ENSG00000275793	1.28764842	6.46E-05
ENSG00000275993	2.83221924	1.39E-15
ENSG00000276180	-1.3041562	0.01958678
ENSG00000276649	1.28184393	0.00887318
ENSG00000277013	1.24964843	0.01346356
ENSG00000277157	-1.264721	0.02150397
ENSG00000277224	-1.3276994	0.00285152
ENSG00000277462	1.31118142	0.00714266
ENSG00000277775	-1.3086986	0.00954504
ENSG00000278272	-1.2642125	0.02118196
ENSG00000278463	-1.2771362	0.01087708
ENSG00000278588	-1.2462717	0.02928107
ENSG00000278828	-1.2585008	0.01561736
ENSG00000279227	1.24912584	0.0284725
ENSG00000279236	1.22588881	0.01978419
ENSG00000280138	1.72860329	4.24E-05
ENSG00000280143	1.30247704	0.00918875
ENSG00000280832	-1.2989863	0.01756393
ENSG00000281766	1.26860203	0.01390691
ENSG00000281881	1.89672302	9.03E-06

Supplementary Table 3c. Differentially expressed genes between 0hr normal glucose and 4hr high glucose samples

Ensembl gene IDs, Fold change (baseline-4HG) and FDR adjusted p-values of DEGs

Gene ID	FC 0NG - 4HG	adj p-value 0NG-4HG
ENSG00000001167	1.50388493	7.02E-12
ENSG00000001460	-1.2343528	0.04074234
ENSG00000002587	1.65169827	9.65E-09
ENSG00000003096	1.20677007	0.04548534
ENSG00000003400	-1.3799249	4.51E-09
ENSG00000003989	1.91226835	3.81E-08
ENSG00000004660	-1.4038136	0.00016266
ENSG00000004777	-1.3400751	0.01337323
ENSG00000004799	-1.3499162	0.00174164
ENSG00000005238	1.51719793	3.32E-13
ENSG00000005513	1.32192336	0.02532309
ENSG00000005700	1.2893227	0.00428386
ENSG00000006327	1.25223725	7.27E-06
ENSG00000006459	1.69786808	3.38E-09
ENSG00000006468	-1.4068468	3.78E-05
ENSG00000006576	1.2912974	0.00534934
ENSG00000006607	1.20016245	1.15E-05
ENSG00000006652	1.4531591	1.0904E-05
ENSG00000007384	-1.5020046	7.79E-07
ENSG00000007908	10.8090666	3.20E-15
ENSG00000007944	-1.3597765	2.55E-05
ENSG00000007968	-1.7354424	4.14E-06
ENSG00000008083	-1.4440425	1.31E-06
ENSG00000008294	1.5287487	1.67E-18
ENSG00000008405	1.20109661	0.00410575
ENSG00000008441	-1.3051782	1.41E-10
ENSG00000010017	1.23614406	2.84E-07
ENSG00000010292	-1.2613352	2.62E-07
ENSG00000010318	-1.3617126	0.00124369
ENSG00000010810	-1.3100985	3.63E-09
ENSG00000010818	1.24574404	0.04261984
ENSG00000011143	-1.2346701	0.02701855

Gene ID	FC 0NG - 4HG	adj p-value 0NG-4HG
ENSG00000011405	1.2550989	0.00194731
ENSG00000011426	-1.240487	0.00332695
ENSG00000011523	-1.4011761	6.48E-11
ENSG00000011638	-1.2159387	0.00059356
ENSG00000012048	-1.3367861	0.00064623
ENSG00000012174	1.21147081	0.00848772
ENSG00000012660	1.2458691	6.82E-06
ENSG00000012983	1.24398396	0.0010459
ENSG00000013573	-1.3175573	0.00039073
ENSG00000013588	-1.3505546	4.58E-05
ENSG00000013810	-1.4161626	5.21E-05
ENSG00000014123	1.25705171	9.98E-05
ENSG00000014138	-1.3081918	6.41E-06
ENSG00000015133	-2.3224583	1.25E-16
ENSG00000015171	1.23470835	6.32E-06
ENSG00000015532	-1.2885618	1.10E-07
ENSG00000015568	1.9361652	5.47E-09
ENSG00000015676	-1.2102721	1.08E-06
ENSG00000018510	1.20517199	0.00120668
ENSG00000019144	1.67862261	4.15E-15
ENSG00000020256	-1.2583155	3.09E-06
ENSG00000020426	1.21012736	0.00410661
ENSG00000020577	1.45118161	7.52E-16
ENSG00000021355	-1.2069311	0.00679192
ENSG00000021645	1.28122218	0.00802422
ENSG00000021762	-1.4043313	6.58E-09
ENSG00000023445	2.24617434	1.97E-06
ENSG00000023572	1.22624792	0.02645677
ENSG00000023909	1.20029096	0.00028828
ENSG00000026559	1.28151337	0.00944772
ENSG00000026652	-1.2106295	0.00389703
ENSG00000026950	-1.3506143	1.60E-06

Gene ID	FC 0NG - 4HG	adj p-value 0NG-4HG
ENSG00000028137	-1.4101878	4.52E-06
ENSG00000028277	-1.4571455	4.4686E-05
ENSG00000031081	1.45147221	3.04E-15
ENSG00000033327	-1.2742695	1.05E-08
ENSG00000034533	1.20626317	0.04248179
ENSG00000035499	-1.3703587	8.61E-05
ENSG00000036257	1.20871214	0.00317784
ENSG00000037280	1.32036081	0.00407695
ENSG00000038295	1.60622055	8.25E-07
ENSG00000038382	1.2043905	1.02E-06
ENSG00000039319	1.35584118	0.00123296
ENSG00000040199	1.25081215	8.56E-05
ENSG00000042088	-1.2013594	2.36E-05
ENSG00000042286	-1.2265462	0.00753861
ENSG00000043093	1.28893004	0.03211289
ENSG00000043143	-2.1058692	9.12E-15
ENSG00000047188	1.22406859	0.00019721
ENSG00000047230	-1.2249269	0.00022286
ENSG00000047579	-1.2648997	3.32E-05
ENSG00000047617	-1.266639	0.00574713
ENSG00000047621	1.29395439	0.00063572
ENSG00000047648	-1.3318601	0.00434621
ENSG00000047932	1.41332464	5.85E-05
ENSG00000048052	1.67771405	0.00007082
ENSG00000048162	1.25580962	1.45E-05
ENSG00000048544	1.21086478	0.00743561
ENSG00000049130	2.67556532	5.24E-09
ENSG00000049192	1.6011829	2.92E-12
ENSG00000049283	-2.05597	6.90E-07
ENSG00000049759	1.80353087	1.13E-13
ENSG00000050130	1.24370605	0.02527495
ENSG00000050820	-1.4054739	4.57E-11

Gene ID	FC 0NG - 4HG	adj p-value 0NG-4HG
ENSG00000051009	-1.210724	0.0099421
ENSG00000051180	-1.4332813	0.00021361
ENSG00000051341	-1.3106482	0.00265638
ENSG00000052749	1.31952693	3.48E-08
ENSG00000052795	1.2524447	0.00025871
ENSG00000053747	-1.2924071	0.00011049
ENSG00000055813	1.28680203	0.00587939
ENSG00000056097	1.21910099	1.37E-07
ENSG00000056277	1.24567955	0.01327858
ENSG00000056558	2.49303817	1.57E-06
ENSG00000056972	-1.2662354	0.00501666
ENSG00000057019	1.40181576	1.89E-06
ENSG00000057252	1.30242546	0.00041819
ENSG00000057294	-1.6385139	1.64E-05
ENSG00000057704	-1.9751741	3.60E-17
ENSG00000058085	-1.2200377	0.03891326
ENSG00000058091	1.36700004	5.85E-07
ENSG00000058453	-1.3592914	3.99E-05
ENSG00000059145	-1.208427	0.00117504
ENSG00000059378	-1.2861995	9.76E-06
ENSG00000060491	-1.2664216	1.13E-05
ENSG00000061676	1.23712126	0.01452298
ENSG00000061938	-1.2207288	2.97E-05
ENSG00000061987	1.22678861	0.00149125
ENSG00000062716	1.28512182	0.01846234
ENSG00000062822	-1.2624422	0.00024319
ENSG00000063438	-1.2072284	0.01305883
ENSG00000064225	1.48466852	4.57E-05
ENSG00000064692	-1.9112512	8.36E-06
ENSG00000064703	1.2510137	0.00012771
ENSG00000064989	1.64758337	7.41E-06
ENSG00000064999	-1.4107622	3.30E-07

Gene ID	FC 0NG - 4HG	adj p-value 0NG-4HG
ENSG00000065054	1.32858935	2.33E-05
ENSG00000065057	-1.2343339	0.00075137
ENSG00000065183	1.2225067	5.23E-08
ENSG00000065308	-1.5925659	4.77E-15
ENSG00000065328	-1.2939707	0.00661781
ENSG00000065559	1.27721755	2.15E-05
ENSG00000066027	-1.2472352	0.00269012
ENSG00000066084	1.34230815	2.10E-07
ENSG00000066629	1.2042016	2.76E-05
ENSG00000066735	-1.7349561	2.21E-11
ENSG00000066777	1.20177398	0.00065416
ENSG00000067064	1.29873163	0.01792903
ENSG00000067182	-1.2407109	1.66E-07
ENSG00000067208	1.23511728	0.00012187
ENSG00000067334	1.220133	3.75E-05
ENSG00000067533	1.2176351	0.00047677
ENSG00000067798	1.72779264	7.38E-18
ENSG00000067955	2.2358486	1.97E-14
ENSG00000068024	-1.30325	5.32E-07
ENSG00000068078	1.74932633	3.77E-10
ENSG00000068137	-1.6726267	1.25E-06
ENSG00000068305	1.48104144	1.46E-08
ENSG00000068366	1.36732246	3.92E-08
ENSG00000068724	-1.2142812	0.00156846
ENSG00000069702	-1.4596737	8.70E-06
ENSG00000069812	-1.3259338	1.08E-06
ENSG00000069966	-1.2306969	0.00025021
ENSG00000070404	1.48177098	1.88E-07
ENSG00000070444	-1.2066068	0.00737156
ENSG00000070610	-1.3704329	0.0009149
ENSG00000070614	-1.3334407	1.94E-10
ENSG00000070961	1.69571795	2.37E-09

Gene ID	FC 0NG - 4HG	adj p-value 0NG-4HG
ENSG00000071205	1.22735225	0.00013973
ENSG00000071246	-1.4373812	0.00033993
ENSG00000071282	-1.3264199	0.00717188
ENSG00000072201	-1.4059406	6.22E-05
ENSG00000072364	1.24883818	6.59E-07
ENSG00000072422	1.22272757	5.17E-05
ENSG00000072756	1.20353327	0.00621671
ENSG00000072840	-1.2715492	4.24E-06
ENSG00000072952	-1.229274	0.02265476
ENSG00000073008	1.53652094	7.16E-15
ENSG00000073111	-1.3105528	0.00016402
ENSG00000073331	-1.4580507	3.02E-07
ENSG00000073417	1.81360645	6.10E-19
ENSG00000073711	-1.2442692	0.00615025
ENSG00000073712	1.47234026	3.01E-14
ENSG00000073756	6.08858848	8.76E-12
ENSG00000073921	1.55937559	2.67E-09
ENSG00000074047	2.65352694	6.49E-15
ENSG00000074855	-1.2264185	0.02343305
ENSG00000075218	-1.3835718	0.00015029
ENSG00000075420	1.22481983	1.15E-05
ENSG00000075539	1.32873797	2.85E-05
ENSG00000075643	-1.3450236	0.00131813
ENSG00000075651	-1.6520334	6.79E-13
ENSG00000075702	-1.482954	1.48E-05
ENSG00000075711	-1.2533619	8.53E-05
ENSG00000075790	1.29636711	0.01715185
ENSG00000076003	-1.2135345	0.00011592
ENSG00000076351	-1.563685	7.14E-08
ENSG00000076356	1.2238982	5.20E-06
ENSG00000076382	-1.2328386	0.00164486
ENSG00000077147	1.42122435	0.00115377

Gene ID	FC 0NG - 4HG	adj p-value 0NG-4HG
ENSG00000077254	1.2132641	0.01329084
ENSG00000077514	-1.2696576	3.84E-05
ENSG00000078043	1.22670436	0.00203747
ENSG00000078140	1.21582679	0.00895178
ENSG00000078177	1.23651412	0.03636689
ENSG00000078687	-1.2034917	0.00024082
ENSG00000078747	1.2985722	1.94E-07
ENSG00000078804	1.21197953	0.0093403
ENSG00000079102	1.56582293	2.80E-06
ENSG00000079335	1.33654395	1.34E-05
ENSG00000079432	-1.2405776	4.36E-06
ENSG00000079435	-1.2699011	0.01301623
ENSG00000079616	-1.4391227	3.52E-07
ENSG00000080371	1.21173928	0.00115951
ENSG00000080561	-1.3838239	4.75E-09
ENSG00000080608	1.36261955	1.20E-09
ENSG00000080839	-1.2870898	0.00649477
ENSG00000080845	-1.2052721	7.88E-06
ENSG00000080986	-1.3359797	0.00113986
ENSG00000081059	1.80066134	2.46E-08
ENSG00000081177	-1.229443	1.27E-06
ENSG00000081320	-1.2420546	0.02097213
ENSG00000081665	1.23042938	0.03413286
ENSG00000081913	1.34930063	0.00245549
ENSG00000082014	1.33282512	0.00865052
ENSG00000082126	-1.2316698	0.00085738
ENSG00000082438	1.34333254	5.40E-06
ENSG00000082458	-1.314951	0.00786171
ENSG00000082512	-1.3853518	0.00073627
ENSG00000083097	1.22419742	0.00145005
ENSG00000083520	1.2110856	0.01154327
ENSG00000083937	1.22972188	0.03549503

Gene ID	FC 0NG - 4HG	adj p-value 0NG-4HG
ENSG00000084073	1.20426065	0.02502775
ENSG00000084093	1.84879277	1.03E-13
ENSG00000084676	1.26866128	2.05E-05
ENSG00000084710	1.53594341	4.45E-05
ENSG00000084734	-1.3662206	0.00010809
ENSG00000085117	-1.2125046	0.00242687
ENSG00000085185	-1.3965896	4.62E-05
ENSG00000085840	-1.3998234	0.00115027
ENSG00000085999	-1.2969149	0.02780176
ENSG00000086015	-1.2736911	1.67E-06
ENSG00000086544	-1.2089932	0.0004412
ENSG00000086696	-1.2859681	0.02529802
ENSG00000086991	1.34680271	6.24E-05
ENSG00000087095	1.29995103	2.66E-06
ENSG00000087157	-1.2793201	0.00010913
ENSG00000087266	-1.452689	5.31E-09
ENSG00000087494	1.6060898	6.52E-05
ENSG00000087586	-1.2293085	0.00235904
ENSG00000087842	-1.205445	0.00020504
ENSG00000087903	1.55128511	0.00030673
ENSG00000088179	-1.2486287	0.00169573
ENSG00000088305	-1.3716692	0.00090945
ENSG00000088325	-1.3266758	4.54E-05
ENSG00000088387	-1.3195403	1.20E-09
ENSG00000088836	-1.2549632	0.00151603
ENSG00000089041	-2.0325236	2.29E-08
ENSG00000089048	1.31975615	7.18E-06
ENSG00000089060	-1.2789954	5.00E-06
ENSG00000089127	-1.4464473	0.02292807
ENSG00000089505	-1.2507454	0.01069571
ENSG00000089685	-1.2289764	0.00090708
ENSG00000089818	1.24568859	2.09E-06

Gene ID	FC 0NG - 4HG	adj p-value 0NG-4HG
ENSG00000089820	-1.3039488	0.00341642
ENSG00000089902	1.21771659	2.68E-08
ENSG00000090238	-1.3531998	3.72E-05
ENSG00000090447	-1.2212368	0.02299541
ENSG00000090530	1.42995256	0.00034613
ENSG00000090612	1.22881385	0.005616
ENSG00000090776	-1.3763488	1.95E-08
ENSG00000090889	-1.3403037	0.00013436
ENSG00000091039	1.22325997	0.00301446
ENSG00000091129	1.4856954	5.86E-10
ENSG00000091137	-1.3005962	0.00488947
ENSG00000091428	1.4858983	1.42E-06
ENSG00000091483	1.2555036	2.79E-06
ENSG00000091656	1.59973973	5.51E-09
ENSG00000091844	-1.4699203	4.61E-05
ENSG00000091879	1.47014496	6.10E-10
ENSG00000091972	-1.5137106	5.18E-06
ENSG00000092036	-1.2283213	0.0170093
ENSG00000092445	1.28219593	2.38E-05
ENSG00000092470	-1.3088617	0.0006933
ENSG00000092621	-1.2197132	0.00875246
ENSG00000092820	-1.204609	1.30E-07
ENSG00000092853	-1.3032006	0.00944564
ENSG00000092969	1.60801766	0.00018154
ENSG00000093009	-1.3535577	0.00453123
ENSG00000093217	-1.2423198	0.00438993
ENSG00000094804	-1.3398013	0.00026981
ENSG00000095015	1.34880791	8.16E-07
ENSG00000095303	-1.2199733	0.00012523
ENSG00000095370	-1.6410646	2.31E-12
ENSG00000095383	-1.4448572	1.49E-05
ENSG00000095397	1.28997326	0.00146793

Gene ID	FC 0NG - 4HG	adj p-value 0NG-4HG
ENSG00000095637	-1.3229158	0.01205808
ENSG00000095713	-1.2227403	0.01896799
ENSG00000095739	1.3234148	0.00178702
ENSG00000095752	2.96558687	3.48E-13
ENSG00000096070	1.24178627	1.39E-07
ENSG00000096433	-1.2676281	3.97E-08
ENSG00000096717	1.23600745	0.00990263
ENSG00000097046	-1.2995325	0.00222951
ENSG00000099204	-1.3362908	9.93E-07
ENSG00000099246	1.29489093	0.00535214
ENSG00000099282	-1.2729123	0.00220301
ENSG00000099822	1.52644996	0.00042001
ENSG00000099875	-1.5589695	5.64E-12
ENSG00000099889	-1.7252877	5.04E-11
ENSG00000099910	-1.3417988	0.00012338
ENSG00000099998	-1.2743189	0.0014604
ENSG00000100027	-1.3047234	0.03269074
ENSG00000100034	-1.3467769	1.19E-10
ENSG00000100036	-1.5852156	3.66E-10
ENSG00000100060	-1.2688079	1.03E-06
ENSG00000100065	-1.5021585	1.66E-09
ENSG00000100147	-1.2818749	0.00482468
ENSG00000100271	-1.2519735	0.00533181
ENSG00000100276	1.23842622	0.01955461
ENSG00000100292	-1.2595203	0.0016165
ENSG00000100294	-1.2056236	0.01564121
ENSG00000100297	-1.2653185	4.37E-05
ENSG00000100304	-1.2068496	1.05E-06
ENSG00000100311	1.53883093	2.33E-05
ENSG00000100342	-1.2206009	0.0153514
ENSG00000100350	-1.2049713	0.00343766
ENSG00000100439	-1.380828	7.72E-05

Gene ID	FC 0NG - 4HG	adj p-value 0NG-4HG
ENSG00000100485	1.23275978	0.00651462
ENSG00000100490	-1.412484	5.25E-05
ENSG00000100522	1.29484528	0.00064646
ENSG00000100523	1.30382762	0.00023182
ENSG00000100526	-1.2176588	0.02348111
ENSG00000100554	1.3096073	6.66E-10
ENSG00000100580	1.2244075	0.00016105
ENSG00000100592	1.43484025	1.04E-11
ENSG00000100629	-1.4523611	9.27E-05
ENSG00000100647	-1.2082336	3.16E-07
ENSG00000100889	-1.3081812	0.00011094
ENSG00000101003	-1.3957551	0.00012286
ENSG00000101040	-1.2895283	2.93E-08
ENSG00000101057	-1.2398964	0.00888125
ENSG00000101096	-1.474024	3.79E-08
ENSG00000101132	1.38377901	0.00961798
ENSG00000101188	-1.273047	0.00020895
ENSG00000101190	-1.2232744	0.00354283
ENSG00000101224	-1.524572	1.52E-10
ENSG00000101236	1.27456898	2.68E-06
ENSG00000101255	-1.557562	4.84E-11
ENSG00000101265	-1.6230342	1.70E-10
ENSG00000101331	-1.3690171	6.42E-06
ENSG00000101384	1.72458175	5.62E-10
ENSG00000101412	-1.3725911	0.00105715
ENSG00000101417	-1.2526728	0.01254159
ENSG00000101445	-2.0319798	4.89E-14
ENSG00000101447	-1.4391507	5.35E-06
ENSG00000101558	1.24079863	0.00011782
ENSG00000101577	-1.4239788	2.40E-12
ENSG00000101665	2.87964251	2.37E-10
ENSG00000101670	2.45138876	5.61E-19

Gene ID	FC 0NG - 4HG	adj p-value 0NG-4HG
ENSG00000101745	1.28504287	0.04717086
ENSG00000101871	-1.693117	1.11E-14
ENSG00000101945	-1.3235074	0.00088832
ENSG00000101974	1.22004749	0.00719907
ENSG00000101986	1.26801392	0.0001822
ENSG00000102010	-1.5824114	8.39E-11
ENSG00000102024	1.25333684	4.96E-07
ENSG00000102098	-1.2094192	0.03974895
ENSG00000102125	-1.2082116	0.00675661
ENSG00000102178	-1.2814661	2.96E-06
ENSG00000102312	1.31720785	0.00027035
ENSG00000102384	-1.280724	0.00526409
ENSG00000102401	1.2045749	0.00055147
ENSG00000102531	1.58640664	9.51E-17
ENSG00000102554	1.29488168	0.00041682
ENSG00000102699	-1.2206685	4.91E-09
ENSG00000102755	2.19147819	2.56E-19
ENSG00000102934	-1.4141829	0.00032511
ENSG00000102935	2.36448236	1.04E-10
ENSG00000102984	1.35945378	0.01032947
ENSG00000103064	1.32364684	1.33E-11
ENSG00000103111	-1.2851443	6.14E-10
ENSG00000103152	-1.2330749	7.66E-06
ENSG00000103254	-1.2520726	0.0086703
ENSG00000103319	-1.3183419	9.17E-12
ENSG00000103365	-1.2015085	2.44E-05
ENSG00000103404	-1.2193109	1.52E-05
ENSG00000103528	1.24073052	0.00950966
ENSG00000103888	1.31753755	6.44E-05
ENSG00000104081	-1.6504078	0.00473446
ENSG00000104131	1.30979645	0.00563058
ENSG00000104147	-1.2382157	0.04800833

Gene ID	FC 0NG - 4HG	adj p-value 0NG-4HG
ENSG00000104312	1.34958493	2.79E-06
ENSG00000104365	-1.235681	2.68E-06
ENSG00000104368	-1.2194978	0.02130386
ENSG00000104388	1.26537894	9.70E-05
ENSG00000104419	1.31450011	1.50E-07
ENSG00000104643	1.27174268	0.00029706
ENSG00000104689	-1.2362829	2.40E-06
ENSG00000104695	1.2772134	1.86E-07
ENSG00000104738	-1.2399751	0.00077634
ENSG00000104856	1.46952247	3.73E-05
ENSG00000104863	1.29030979	0.00215894
ENSG00000104889	-1.280783	0.0042381
ENSG00000104903	1.27032247	0.00243138
ENSG00000104951	1.3073187	0.01299799
ENSG00000105011	-1.3378228	0.00015282
ENSG00000105088	2.52209063	1.14E-16
ENSG00000105281	1.30096377	1.81E-06
ENSG00000105287	-1.4806921	5.83E-11
ENSG00000105329	1.24727383	6.69E-06
ENSG00000105486	-1.2762108	5.61E-07
ENSG00000105514	-1.2813218	1.37E-06
ENSG00000105538	-1.2186602	2.08E-05
ENSG00000105662	-1.3694468	2.95E-05
ENSG00000105722	1.3150582	1.51E-05
ENSG00000105738	-1.2311393	8.91E-06
ENSG00000105793	1.34414899	0.00193006
ENSG00000105810	1.82177789	1.93E-11
ENSG00000105821	1.21724173	6.57E-06
ENSG00000105825	1.46353459	3.43E-13
ENSG00000105849	1.26647638	0.00675073
ENSG00000105851	-1.5229351	9.99E-10
ENSG00000105855	2.34974259	3.06E-14
ENSG00000105887	1.2164543	0.00014935
ENSG00000105926	1.22372536	0.0008507
ENSG00000105967	1.28702905	0.01808015
ENSG00000105976	-1.2126198	3.38E-06
ENSG00000106070	2.48435432	5.25E-23
ENSG00000106100	-1.5275484	1.53E-10
ENSG00000106351	-1.7654722	1.49E-11
ENSG00000106366	1.29657254	4.48E-09
ENSG00000106479	-1.3317521	0.00267766
ENSG00000106537	1.55594716	4.72E-10
ENSG00000106546	1.34469515	1.12E-08
ENSG00000106560	-1.308778	0.00056762
ENSG00000106571	-1.3881599	5.53E-09
ENSG00000106615	1.24017791	5.93E-05
ENSG00000106688	-1.2460753	3.96E-07
ENSG00000106723	1.30253142	0.00131198
ENSG00000106799	1.34916426	0.00013887
ENSG00000106804	-1.3090224	0.002547
ENSG00000106852	-1.6279773	2.26E-07
ENSG00000106948	-1.4984536	4.44E-08
ENSG00000106991	1.21544965	0.00020905
ENSG00000106993	1.27220501	0.00091292
ENSG00000107077	-1.3163644	0.00061144
ENSG00000107099	1.30730392	0.005105
ENSG00000107104	1.42738405	1.62E-13
ENSG00000107130	1.22210423	1.56E-07
ENSG00000107175	1.22804833	0.00036274
ENSG00000107185	-1.2848435	6.14E-07
ENSG00000107485	-1.3737542	5.90E-06
ENSG00000107551	-1.5998009	1.68E-05
ENSG00000107560	1.23231112	0.00512641
ENSG00000107562	4.82022578	2.43E-23
ENSG00000107643	1.21915152	0.00035923
ENSG00000107672	-1.2121014	3.03E-05
ENSG00000107679	1.39612524	4.07E-10
ENSG00000107719	-1.7172668	3.42E-09
ENSG00000107731	3.30270206	6.99E-16
ENSG00000107771	1.42485561	4.91E-07
ENSG00000107779	1.21463899	0.00194352
ENSG00000107789	1.25483914	0.00121224
ENSG00000107807	1.48012344	0.00022978
ENSG00000107821	1.56407247	0.00070083
ENSG00000107863	1.63492079	2.25E-18
ENSG00000107872	-1.2503262	0.0065153
ENSG00000107937	1.22378706	8.19E-08
ENSG00000107949	1.214601	6.39E-05
ENSG00000107957	-1.2115788	6.74E-08
ENSG00000107984	2.01084806	1.45E-12
ENSG00000108021	1.21213066	3.41E-05
ENSG00000108061	1.26446247	0.00807201
ENSG00000108219	-1.2975713	1.43E-10
ENSG00000108262	-1.358402	8.16E-10
ENSG00000108312	1.56495045	2.75E-14
ENSG00000108433	1.36983103	5.04E-08
ENSG00000108556	1.25045082	0.02536959
ENSG00000108587	1.21524324	2.66E-07
ENSG00000108622	-1.3086162	1.14E-10
ENSG00000108799	-1.2486993	0.0010913
ENSG00000108854	1.26377757	1.02E-06
ENSG00000108960	1.73472604	6.21E-08
ENSG00000109046	-1.2833463	0.00030205
ENSG00000109133	1.23403884	0.01236972
ENSG00000109189	1.35671082	1.44E-05
ENSG00000109320	1.39593003	1.01E-15

Gene ID	FC 0NG - 4HG	adj p-value 0NG-4HG
ENSG00000109321	1.32548865	1.52E-05
ENSG00000109332	1.2315877	0.02016754
ENSG00000109458	-1.4436441	1.13E-05
ENSG00000109466	1.29639425	1.49E-07
ENSG00000109501	-1.4620827	9.67E-08
ENSG00000109534	1.22705149	0.04537548
ENSG00000109674	-1.3530366	0.00299374
ENSG00000109685	-1.2765366	5.90E-09
ENSG00000109689	1.29275607	7.94E-06
ENSG00000109756	1.33818899	3.56E-08
ENSG00000109805	-1.2692792	0.02732006
ENSG00000109814	1.20174056	3.87E-07
ENSG00000109929	1.43073599	0.00284759
ENSG00000110047	-1.2964839	3.16E-07
ENSG00000110080	-1.4050823	2.87E-09
ENSG00000110172	1.24644432	8.51E-05
ENSG00000110318	1.29270628	0.02793177
ENSG00000110330	1.92270021	1.38E-11
ENSG00000110422	1.21217005	0.00146792
ENSG00000110675	1.236875	0.04872544
ENSG00000110852	1.36674383	0.01109669
ENSG00000110888	1.32129441	0.00038076
ENSG00000110931	-1.2740145	2.25E-10
ENSG00000111057	-1.2152643	0.00056648
ENSG00000111077	-1.5471121	2.15E-11
ENSG00000111110	-1.630107	0.0006694
ENSG00000111145	-1.2820283	7.09E-10
ENSG00000111199	1.26930083	0.01453327
ENSG00000111206	-1.2386542	1.9436E-05
ENSG00000111247	-1.2816368	0.00209578
ENSG00000111300	1.21358748	0.00189314
ENSG00000111331	-1.2791338	3.62E-05

Gene ID	FC 0NG - 4HG	adj p-value 0NG-4HG
ENSG00000111335	-1.4773378	0.00047551
ENSG00000111339	1.56669496	2.57E-08
ENSG00000111445	-1.230826	0.0031866
ENSG00000111490	1.47070569	2.99E-07
ENSG00000111530	1.21935054	0.00416049
ENSG00000111602	-1.2247471	0.00052217
ENSG00000111647	1.59292581	1.22E-05
ENSG00000111665	-1.4151285	0.00060897
ENSG00000111674	-1.2894493	0.0104152
ENSG00000111725	-1.5757664	1.44E-09
ENSG00000111801	-1.2380648	0.04754517
ENSG00000111817	1.36413965	1.46E-07
ENSG00000111845	1.22734473	0.00113597
ENSG00000111846	-1.550808	6.22E-11
ENSG00000111859	1.89372702	2.44E-09
ENSG00000111907	1.27906503	0.01120955
ENSG00000111912	1.26796469	6.00E-06
ENSG00000111961	-1.7458241	6.71E-24
ENSG00000112033	1.36440212	3.54E-09
ENSG00000112039	-1.2290514	0.00203179
ENSG00000112096	-1.2689008	6.64E-05
ENSG00000112137	-1.3176572	2.09E-05
ENSG00000112183	1.40585325	0.00699927
ENSG00000112208	1.23969305	0.01992315
ENSG00000112210	1.57329395	3.75E-10
ENSG00000112245	1.3601305	6.02E-07
ENSG00000112297	-1.7262152	1.44E-08
ENSG00000112305	1.40449067	6.79E-09
ENSG00000112312	-1.2911117	1.13E-05
ENSG00000112414	-1.239467	0.00988447
ENSG00000112531	1.21345782	0.00027546
ENSG00000112541	1.9638766	2.07E-09

Gene ID	FC 0NG - 4HG	adj p-value 0NG-4HG
ENSG00000112559	1.68756952	1.67E-09
ENSG00000112679	-1.2545894	1.49E-06
ENSG00000112697	1.25839806	0.02064866
ENSG00000112701	1.27341801	0.00575765
ENSG00000112742	-1.3425267	0.04251383
ENSG00000112773	2.51626648	3.75E-08
ENSG00000112837	1.31206965	5.46E-05
ENSG00000112851	1.38613354	5.04E-05
ENSG00000112877	-1.2120696	0.02875529
ENSG00000112983	-1.2260306	7.65E-05
ENSG00000112984	-1.3271624	1.67E-05
ENSG00000113161	1.20037708	0.04817365
ENSG00000113368	-1.3243211	6.07E-08
ENSG00000113369	1.39327403	3.0454E-05
ENSG00000113384	1.24135175	2.74E-06
ENSG00000113387	1.20573708	0.00445749
ENSG00000113460	1.22921625	0.00897558
ENSG00000113532	1.91190303	7.05E-08
ENSG00000113552	-1.2737511	1.08E-09
ENSG00000113555	-1.8225885	1.37E-07
ENSG00000113575	1.27605504	0.00028262
ENSG00000113580	1.97424033	8.75E-15
ENSG00000113594	1.29107046	0.0005747
ENSG00000113595	1.25098237	0.03826164
ENSG00000113742	1.42592064	3.89E-08
ENSG00000113749	-1.3835669	0.0103864
ENSG00000113916	1.36788434	1.7112E-05
ENSG00000114062	1.27938257	0.00016185
ENSG00000114166	-1.2559777	0.00780973
ENSG00000114302	1.26220796	7.73E-09
ENSG00000114315	-1.9416646	3.20E-05
ENSG00000114346	-1.2297431	0.00956901

Gene ID	FC 0NG - 4HG	adj p-value 0NG-4HG
ENSG00000114423	1.48054268	1.67E-07
ENSG00000114450	1.28168187	0.00054046
ENSG00000114480	1.20905919	1.85E-05
ENSG00000114529	1.64139028	2.59E-07
ENSG00000114626	-1.4591605	0.00064827
ENSG00000114698	-1.2337728	0.00823648
ENSG00000114812	-1.780312	1.63E-05
ENSG00000114841	-1.2724594	0.03290416
ENSG00000114978	1.21501048	0.0239226
ENSG00000114999	1.30640174	8.71E-15
ENSG00000115084	1.27833476	0.00102066
ENSG00000115091	1.25638385	1.73E-08
ENSG00000115163	-1.3042842	0.00264341
ENSG00000115170	1.33450892	5.08E-11
ENSG00000115183	-1.220022	5.40E-06
ENSG00000115233	1.25086317	1.85E-06
ENSG00000115255	-1.2414357	0.01796661
ENSG00000115290	1.20469646	0.02975803
ENSG00000115368	1.24659449	0.00024548
ENSG00000115520	1.28593327	0.00293525
ENSG00000115525	-1.4375156	0.00052135
ENSG00000115540	1.31338493	0.00170423
ENSG00000115548	1.39890643	3.02E-09
ENSG00000115602	2.56175012	4.35E-09
ENSG00000115604	1.52543443	4.75E-06
ENSG00000115641	-1.4459915	5.06E-11
ENSG00000115649	-1.23416	0.00061774
ENSG00000115687	-1.3520554	0.00016348
ENSG00000115738	2.06289284	3.37E-06
ENSG00000115825	1.21383296	0.00125218
ENSG00000115884	-1.248401	0.02077618
ENSG00000115896	1.68880876	9.10E-06

Gene ID	FC 0NG - 4HG	adj p-value 0NG-4HG
ENSG00000115902	-1.2854308	1.02E-05
ENSG00000115946	1.37084995	6.33E-09
ENSG00000115963	1.31013442	0.00048947
ENSG00000116017	-1.2895203	0.01563659
ENSG00000116095	1.32272789	0.00013754
ENSG00000116106	-1.2313402	4.79E-05
ENSG00000116273	-1.2899181	1.2786E-05
ENSG00000116473	1.26357071	2.36E-08
ENSG00000116514	-1.4148993	1.26E-09
ENSG00000116560	1.20942771	2.47E-09
ENSG00000116584	-1.4767853	3.43E-13
ENSG00000116641	1.49240241	8.90E-08
ENSG00000116704	1.32539953	1.15E-05
ENSG00000116711	2.43855994	8.97E-11
ENSG00000116717	-1.235735	0.00019165
ENSG00000116815	1.4891005	0.0002538
ENSG00000116991	-1.2831146	0.00014205
ENSG00000117114	1.2800394	6.84E-08
ENSG00000117133	1.30124622	9.31E-05
ENSG00000117143	1.65603636	2.79E-15
ENSG00000117152	1.5173385	1.88E-08
ENSG00000117174	1.33711725	6.99E-06
ENSG00000117226	-1.2852589	2.95E-08
ENSG00000117318	1.59403654	4.63E-10
ENSG00000117399	-1.4228675	4.57E-06
ENSG00000117479	1.26916023	0.008354
ENSG00000117595	2.98998592	1.27E-11
ENSG00000117597	1.35927864	1.24E-09
ENSG00000117650	-1.4282899	1.30E-05
ENSG00000117724	-1.3671799	4.83E-05
ENSG00000117748	-1.2202502	1.57E-05
ENSG00000117758	1.23845091	3.45E-06

Gene ID	FC 0NG - 4HG	adj p-value 0NG-4HG
ENSG00000118058	-1.2236685	0.00413764
ENSG00000118193	-1.3502474	0.00046427
ENSG00000118200	1.27275802	2.33E-05
ENSG00000118246	1.27827232	0.007725
ENSG00000118263	1.29281937	8.54E-06
ENSG00000118402	1.70604241	8.10E-07
ENSG00000118407	-1.3113413	0.00775345
ENSG00000118454	1.2279313	4.29E-05
ENSG00000118496	1.32599165	0.00014723
ENSG00000118503	1.4293873	0.00128689
ENSG00000118508	1.24250502	2.40E-06
ENSG00000118515	1.78922926	8.31E-21
ENSG00000118523	1.32704833	8.68E-11
ENSG00000118655	-1.2355792	0.00623548
ENSG00000118689	-1.2918086	4.49E-06
ENSG00000118922	-1.3026795	0.00103487
ENSG00000118939	1.27771752	0.03212035
ENSG00000118946	4.33083283	4.10E-11
ENSG00000118985	1.76013281	1.52E-15
ENSG00000119138	1.20182847	0.00192709
ENSG00000119139	1.35537529	1.12E-09
ENSG00000119231	1.25504011	8.37E-10
ENSG00000119314	1.24069293	0.00666642
ENSG00000119333	-1.2192262	2.64E-05
ENSG00000119397	-1.3345296	2.65E-06
ENSG00000119403	-1.2465598	1.79E-05
ENSG00000119522	-1.3523574	8.34E-08
ENSG00000119630	-1.4732124	1.16E-07
ENSG00000119661	1.3320861	0.0001686
ENSG00000119673	-1.2704056	0.01712574
ENSG00000119686	-1.2555364	0.00767002
ENSG00000119771	1.33950857	6.24E-09

Gene ID	FC 0NG - 4HG	adj p-value 0NG-4HG
ENSG00000119778	1.30625314	8.0684E-05
ENSG00000119801	1.28712563	0.00188225
ENSG00000119862	-1.2712982	0.00382999
ENSG00000119917	-1.3329804	0.01348154
ENSG00000119938	1.97927751	4.73E-10
ENSG00000119965	1.34805474	0.00010693
ENSG00000120137	1.23952016	0.0086976
ENSG00000120217	1.26138878	0.00090546
ENSG00000120278	-2.2841747	7.99E-21
ENSG00000120279	1.22995591	0.00120201
ENSG00000120457	-1.2745219	0.00138193
ENSG00000120539	-1.2471663	0.00497767
ENSG00000120549	-1.3718982	0.00097284
ENSG00000120686	1.23819242	0.00145629
ENSG00000120693	3.79180687	2.62E-19
ENSG00000120694	1.20661129	7.89E-05
ENSG00000120705	1.24323358	1.01E-07
ENSG00000120742	1.23246559	0.01681385
ENSG00000120800	1.27416686	1.95E-05
ENSG00000120802	-1.2277557	0.00019447
ENSG00000120875	-1.7599975	1.33E-17
ENSG00000120899	-1.3498056	0.00029401
ENSG00000121060	-1.3171004	6.29E-10
ENSG00000121152	-1.3880513	0.00017436
ENSG00000121236	-1.3033171	0.0226981
ENSG00000121350	1.32109286	0.00772866
ENSG00000121390	1.22975954	8.72E-08
ENSG00000121481	1.20543785	0.00043195
ENSG00000121644	1.252194	0.00611697
ENSG00000121741	1.23861389	0.02645337
ENSG00000121797	-1.4947202	2.55E-07
ENSG00000121858	-2.3033704	1.38E-09

Gene ID	FC 0NG - 4HG	adj p-value 0NG-4HG
ENSG00000121879	1.28130808	0.01135474
ENSG00000121957	-1.5863924	4.28E-09
ENSG00000121964	-1.2130488	0.00913838
ENSG00000121966	-2.5765591	7.89E-18
ENSG00000121988	-1.3792292	0.00011239
ENSG00000122068	1.41654599	8.25E-11
ENSG00000122378	-1.2187644	4.09E-07
ENSG00000122386	-1.484582	1.80E-06
ENSG00000122420	1.24142854	0.0262701
ENSG00000122515	-1.2023789	3.64E-05
ENSG00000122641	2.1106404	1.77E-12
ENSG00000122644	-1.2312716	0.01768498
ENSG00000122786	1.30205486	1.43E-10
ENSG00000122861	-1.9003366	8.73E-12
ENSG00000122863	1.68600594	5.39E-10
ENSG00000122870	1.66705481	3.21E-08
ENSG00000122912	1.26948854	7.74E-05
ENSG00000122952	-1.2172008	0.00051464
ENSG00000122966	-1.3674525	6.48E-06
ENSG00000123066	1.38769314	7.04E-12
ENSG00000123080	-1.5063459	5.84E-08
ENSG00000123091	1.2454001	0.00027746
ENSG00000123094	1.40165717	3.55E-07
ENSG00000123095	1.45659377	0.00113751
ENSG00000123178	1.20399469	0.00150147
ENSG00000123358	1.54580843	0.00948548
ENSG00000123384	-1.2551988	0.01435627
ENSG00000123473	-1.2912189	0.00038029
ENSG00000123485	-1.5849384	2.4485E-05
ENSG00000123545	1.37059738	0.00108585
ENSG00000123552	1.22855753	0.03793336
ENSG00000123700	2.06390533	1.49E-06

Gene ID	FC 0NG - 4HG	adj p-value 0NG-4HG
ENSG00000123815	-1.2172319	0.03382221
ENSG00000124006	-1.2849464	0.00937057
ENSG00000124019	-1.2364581	0.00317784
ENSG00000124074	-1.2009543	0.01514764
ENSG00000124216	1.32235401	0.00177647
ENSG00000124225	1.84662571	1.14E-08
ENSG00000124275	1.20642227	5.55E-06
ENSG00000124279	1.24343508	0.03861074
ENSG00000124383	1.20057394	0.00096069
ENSG00000124575	-1.5069364	8.93E-07
ENSG00000124635	-1.3384666	0.00527778
ENSG00000124772	1.57051193	6.19E-06
ENSG00000124802	1.44812464	0.02421295
ENSG00000125089	-1.3323283	7.51E-06
ENSG00000125257	1.256674	4.35E-05
ENSG00000125266	1.96793858	3.58E-12
ENSG00000125347	-1.5699485	1.68E-05
ENSG00000125378	1.43107515	7.54E-05
ENSG00000125457	-1.2116644	0.03099173
ENSG00000125520	-1.2187851	0.00016201
ENSG00000125845	1.87067739	2.57E-10
ENSG00000125871	-1.3745127	4.19E-08
ENSG00000125898	1.39009303	9.58E-05
ENSG00000125965	-1.3024404	0.00397287
ENSG00000125966	1.41892108	0.00086085
ENSG00000125968	2.7041817	8.98E-12
ENSG00000126001	-1.2741443	2.07E-05
ENSG00000126070	1.24310076	0.00563372
ENSG00000126215	-1.2720132	0.0005475
ENSG00000126351	-1.5004519	3.75E-11
ENSG00000126368	-1.2736623	0.0159641
ENSG00000126391	1.20627356	1.97E-05

Gene ID	FC 0NG - 4HG	adj p-value 0NG-4HG
ENSG00000126456	-1.2082158	0.00021466
ENSG00000126562	1.2632146	0.00196557
ENSG00000126705	-1.5925553	2.50E-08
ENSG00000126778	1.3130397	0.01061897
ENSG00000126790	1.23220049	0.00030344
ENSG00000126822	-1.3321022	0.00084573
ENSG00000126878	-1.2486108	0.00102205
ENSG00000126882	-1.9129609	3.37E-08
ENSG00000127080	1.46074659	6.01E-10
ENSG00000127124	1.33726743	0.0008175
ENSG00000127220	-1.3307518	0.00239082
ENSG00000127314	1.37127249	0.00067466
ENSG00000127418	1.2772962	0.00013449
ENSG00000127419	-1.2030823	0.02365418
ENSG00000127511	-1.2165651	1.15E-06
ENSG00000127533	-2.6376929	9.53E-15
ENSG00000127564	-1.3494819	0.00221036
ENSG00000127824	-1.4515161	4.20E-06
ENSG00000128052	-1.2483957	1.27E-07
ENSG00000128266	-1.2166465	0.03615561
ENSG00000128284	-1.6739949	5.46E-09
ENSG00000128335	-1.2382067	2.98E-07
ENSG00000128394	-1.3223758	0.00232573
ENSG00000128567	1.5261509	9.72E-13
ENSG00000128590	1.28190935	0.00632243
ENSG00000128594	8.21461216	4.78E-22
ENSG00000128606	1.2445748	0.00068834
ENSG00000128805	-1.5593224	6.07E-14
ENSG00000128815	-1.526233	1.48E-06
ENSG00000128833	-1.2248634	0.03594826
ENSG00000128849	-1.4056736	1.58E-08
ENSG00000128881	-1.2430404	0.00214406

Gene ID	FC 0NG - 4HG	adj p-value 0NG-4HG
ENSG00000128917	-3.7669768	4.07E-17
ENSG00000128923	-1.3873788	1.73E-06
ENSG00000128965	-1.3101883	0.03697184
ENSG00000129083	1.23307256	0.00023608
ENSG00000129158	-1.2496341	0.01651918
ENSG00000129173	-1.6342699	1.95E-08
ENSG00000129195	-1.3327826	0.00033167
ENSG00000129292	1.25382843	2.48E-06
ENSG00000129355	-1.3397978	0.00263037
ENSG00000129422	-1.6681186	1.48E-14
ENSG00000129474	-1.3932301	1.66E-08
ENSG00000129595	1.24905474	0.00022813
ENSG00000129667	-1.2587514	4.19E-05
ENSG00000129810	-1.3106261	0.00229268
ENSG00000130066	1.82466333	3.03E-10
ENSG00000130201	2.01087612	7.02E-11
ENSG00000130270	-1.300934	0.00102577
ENSG00000130300	1.4666574	0.00604429
ENSG00000130338	1.33891452	2.08E-07
ENSG00000130449	-1.3017345	3.11E-13
ENSG00000130517	-1.3872847	9.20E-07
ENSG00000130590	-1.5359119	4.62E-06
ENSG00000130702	-1.4908754	1.20E-09
ENSG00000130706	1.21679698	1.01E-05
ENSG00000130751	1.28715011	6.26E-05
ENSG00000130775	-1.2324537	0.01208329
ENSG00000130956	1.46309238	8.14E-09
ENSG00000131016	1.37012446	1.07E-09
ENSG00000131149	1.87743384	2.10E-12
ENSG00000131153	-1.3210811	0.00060104
ENSG00000131370	-1.8442254	2.63E-18
ENSG00000131378	1.40303296	2.83E-11

Gene ID	FC 0NG - 4HG	adj p-value 0NG-4HG
ENSG00000131386	-1.5229599	0.00059175
ENSG00000131409	3.56928183	5.42E-10
ENSG00000131435	1.21106725	0.00045145
ENSG00000131437	1.21723288	0.00092229
ENSG00000131459	1.3215601	0.00098184
ENSG00000131634	1.39064221	9.51E-07
ENSG00000131652	-1.2262389	0.00526314
ENSG00000131711	1.20261007	0.01551086
ENSG00000131724	1.43780937	9.07E-08
ENSG00000131725	1.22983473	3.37E-05
ENSG00000131747	-1.3383636	0.00123171
ENSG00000131759	-1.3151848	0.00123341
ENSG00000131831	1.9119242	2.55E-10
ENSG00000131979	1.50850822	7.92E-05
ENSG00000132003	-1.2980222	0.00027245
ENSG00000132109	-1.2102896	0.00086152
ENSG00000132205	-1.4283342	6.36E-05
ENSG00000132286	1.21125616	0.00034888
ENSG00000132294	1.40483627	2.45E-07
ENSG00000132326	1.45476195	9.18E-05
ENSG00000132388	1.27926657	0.00051799
ENSG00000132432	1.23499264	0.01848428
ENSG00000132436	-1.3754528	0.00022294
ENSG00000132470	-1.5573546	1.19E-06
ENSG00000132563	-1.3724703	0.00137776
ENSG00000132570	-1.3013994	0.0036833
ENSG00000132589	-1.3007431	8.26E-08
ENSG00000132603	1.25180047	1.75E-06
ENSG00000132613	-1.3369592	1.27E-06
ENSG00000132622	-1.5356625	2.24E-07
ENSG00000132623	-1.2357333	0.03387797
ENSG00000132640	1.31468359	5.66E-08

Gene ID	FC 0NG - 4HG	adj p-value 0NG-4HG
ENSG00000132646	-1.2587985	1.19E-05
ENSG00000132694	-1.2333928	3.25E-06
ENSG00000132906	-1.3162897	2.62E-05
ENSG00000132963	1.21767395	0.0198427
ENSG00000133056	-1.7077774	3.56E-10
ENSG00000133065	1.20692737	2.97E-06
ENSG00000133401	-1.5498245	5.3113E-05
ENSG00000133466	-1.2767846	0.03560269
ENSG00000133561	-1.3825992	1.76E-09
ENSG00000133574	-1.2419413	0.01186703
ENSG00000133612	-1.3277409	3.21E-08
ENSG00000133641	1.2858835	0.00493456
ENSG00000133657	2.47045746	8.69E-13
ENSG00000133773	1.29552461	5.60E-05
ENSG00000133789	1.32724986	2.29E-09
ENSG00000133794	1.4217712	7.68E-07
ENSG00000133805	-1.4767468	6.61E-05
ENSG00000133818	1.3624855	3.33E-11
ENSG00000133858	1.25675827	0.00755199
ENSG00000133874	1.25521035	0.00328996
ENSG00000134107	-1.5389515	1.55E-10
ENSG00000134108	1.2134594	0.02778851
ENSG00000134138	-1.3182224	9.96E-05
ENSG00000134222	-1.7178352	9.66E-08
ENSG00000134278	1.35469328	1.56E-12
ENSG00000134294	2.0811269	7.00E-09
ENSG00000134324	-1.2115125	9.22E-06
ENSG00000134352	1.43774869	1.05E-05
ENSG00000134363	1.87743472	6.09E-11
ENSG00000134371	1.2882256	2.28E-07
ENSG00000134375	1.27296977	8.95E-05
ENSG00000134480	1.29593886	5.27E-05

Gene ID	FC 0NG - 4HG	adj p-value 0NG-4HG
ENSG00000134508	-1.2375203	0.00891703
ENSG00000134531	1.30030091	7.30E-15
ENSG00000134574	-1.2787534	1.62E-07
ENSG00000134668	1.24258011	0.00944266
ENSG00000134690	-1.4765285	8.89E-06
ENSG00000134698	1.21347282	9.33E-05
ENSG00000134769	1.32012384	2.78E-05
ENSG00000134780	-1.2243117	0.01165235
ENSG00000134802	-1.3552875	1.35E-07
ENSG00000134852	1.23609734	0.00709312
ENSG00000134987	1.20096465	0.0105129
ENSG00000135063	-1.2599558	0.01395955
ENSG00000135069	-1.267379	1.80E-07
ENSG00000135074	-1.3081004	7.91E-05
ENSG00000135083	-1.345761	5.45E-05
ENSG00000135119	-1.3500722	0.0215124
ENSG00000135269	1.30528112	4.05E-07
ENSG00000135338	1.32097372	0.01533389
ENSG00000135362	-1.3154854	3.79E-05
ENSG00000135365	-1.3450867	1.67E-07
ENSG00000135451	-1.3510759	0.00571759
ENSG00000135473	-1.200641	0.00941202
ENSG00000135476	-1.5712631	2.46E-05
ENSG00000135480	1.23783605	0.00549131
ENSG00000135547	4.07512725	6.02E-15
ENSG00000135604	-1.5245481	2.73E-09
ENSG00000135723	1.28437772	1.13E-07
ENSG00000135842	-1.2810846	4.06E-05
ENSG00000135919	1.26929733	1.29E-06
ENSG00000135966	-1.8888621	6.79E-17
ENSG00000135968	1.26519659	0.00593717
ENSG00000136021	1.24815245	0.01120476

Gene ID	FC 0NG - 4HG	adj p-value 0NG-4HG
ENSG00000136044	1.41374705	9.85E-09
ENSG00000136048	1.24678364	3.00E-05
ENSG00000136052	1.36057325	0.00922863
ENSG00000136114	-1.4320668	4.13E-08
ENSG00000136153	-1.204529	0.00047776
ENSG00000136158	1.21208535	0.01792879
ENSG00000136237	-1.7253758	1.85E-11
ENSG00000136244	2.30837779	7.94E-10
ENSG00000136305	-1.2537656	0.0410207
ENSG00000136381	1.26855409	0.00125733
ENSG00000136383	1.50584063	5.12E-09
ENSG00000136404	1.49100523	2.88E-08
ENSG00000136449	-1.4722028	0.00156952
ENSG00000136490	1.5272401	4.84E-10
ENSG00000136603	1.38774439	7.22E-05
ENSG00000136717	-1.3639258	6.66E-07
ENSG00000136770	1.25774704	1.39E-06
ENSG00000136826	1.25128238	0.03249044
ENSG00000136856	1.20934589	0.0408744
ENSG00000136859	-1.2947326	1.00E-06
ENSG00000136861	-1.270468	5.03E-06
ENSG00000136869	1.57215335	3.66E-11
ENSG00000136870	1.28743741	3.24E-05
ENSG00000136888	1.23508461	7.35E-07
ENSG00000136897	1.245642	0.00028607
ENSG00000136935	-1.2455264	1.01E-06
ENSG00000136940	-1.2147511	8.36E-06
ENSG00000137094	1.23190203	0.00065686
ENSG00000137135	-1.3671388	0.0003902
ENSG00000137177	1.55783711	7.55E-16
ENSG00000137193	-1.5008983	0.00120652
ENSG00000137203	1.85819928	3.52E-11

Gene ID	FC 0NG - 4HG	adj p-value 0NG-4HG
ENSG00000137310	-1.2251459	0.03007465
ENSG00000137331	-1.3044585	0.00028395
ENSG00000137393	1.30380396	9.97E-05
ENSG00000137414	1.39223587	2.51E-05
ENSG00000137486	-1.5914982	2.71E-12
ENSG00000137494	1.33551262	2.84E-06
ENSG00000137497	-1.2060181	2.90E-07
ENSG00000137502	1.4870255	7.81E-06
ENSG00000137522	1.32780482	1.61E-06
ENSG00000137692	1.37168906	5.55E-10
ENSG00000137693	1.55880877	5.61E-08
ENSG00000137710	1.21850823	2.12E-05
ENSG00000137713	1.28014907	3.72E-08
ENSG00000137727	1.76422696	1.31E-09
ENSG00000137760	1.3502001	1.77E-05
ENSG00000137764	-1.3429236	2.17E-06
ENSG00000137767	-1.3214875	0.0010221
ENSG00000137801	1.2859973	8.33E-08
ENSG00000137802	-1.2896228	1.06E-06
ENSG00000137804	-1.3426531	0.00010405
ENSG00000137807	-1.4537023	6.96E-07
ENSG00000137831	1.63846126	5.08E-09
ENSG00000137834	10.9455027	2.10E-20
ENSG00000137872	2.7837046	3.93E-15
ENSG00000137878	-1.3320784	0.00641209
ENSG00000137955	1.22160189	0.0081262
ENSG00000138002	-1.2159024	0.00080733
ENSG00000138018	1.37076058	0.00098045
ENSG00000138081	1.2492722	0.00612336
ENSG00000138092	-1.2244621	0.0006694
ENSG00000138101	-1.2198216	0.01557945
ENSG00000138134	-1.3202737	0.00190866

Gene ID	FC 0NG - 4HG	adj p-value 0NG-4HG
ENSG00000138135	1.51033286	1.41E-11
ENSG00000138162	-1.6176143	6.59E-09
ENSG00000138180	-1.2763613	0.00370837
ENSG00000138347	-1.5092411	0.00032356
ENSG00000138376	-1.3531428	0.00013013
ENSG00000138411	1.49035747	5.27E-11
ENSG00000138448	1.48540783	1.91E-07
ENSG00000138449	1.52846355	2.56E-08
ENSG00000138496	-1.3451502	0.0005531
ENSG00000138600	1.26771561	0.00301946
ENSG00000138623	1.66104072	0.00018963
ENSG00000138639	1.4024047	1.24E-12
ENSG00000138650	1.68590219	5.14E-08
ENSG00000138675	2.02115029	1.76E-10
ENSG00000138678	-1.3747056	8.79E-06
ENSG00000138785	1.2057726	0.00132743
ENSG00000138802	1.2024924	5.95E-05
ENSG00000138835	-1.222965	0.01806923
ENSG00000139112	1.21509949	0.00054375
ENSG00000139154	1.34289294	2.69E-07
ENSG00000139163	1.34581214	0.00014722
ENSG00000139218	1.28939953	6.29E-05
ENSG00000139266	-1.3429342	0.04823473
ENSG00000139278	1.48441978	2.51E-09
ENSG00000139289	-1.3012373	0.00020879
ENSG00000139354	-1.4922617	1.00E-06
ENSG00000139372	1.38234106	2.10E-07
ENSG00000139505	1.2714221	0.00398454
ENSG00000139508	-1.3478983	0.00894423
ENSG00000139517	1.25617536	0.00012394
ENSG00000139687	1.37972732	5.12E-05
ENSG00000139880	1.22421858	0.00487216

Gene ID	FC 0NG - 4HG	adj p-value 0NG-4HG
ENSG00000139926	-1.4078842	4.02E-10
ENSG00000140006	1.20321455	0.04568611
ENSG00000140009	-1.212099	0.00371027
ENSG00000140280	1.36622517	0.00106286
ENSG00000140382	-1.2391951	8.32E-07
ENSG00000140403	1.24392507	5.04E-05
ENSG00000140416	1.61039022	8.11E-18
ENSG00000140450	1.52202539	1.33E-05
ENSG00000140451	-1.7102415	3.36E-06
ENSG00000140464	-1.2771497	1.72E-08
ENSG00000140534	-1.5589568	2.06E-06
ENSG00000140564	1.4262472	1.99E-10
ENSG00000140691	-1.2757478	0.00022241
ENSG00000140853	-1.372046	0.00013823
ENSG00000140873	-1.3499936	0.00022322
ENSG00000141068	-1.5357276	1.34E-10
ENSG00000141298	-1.2321527	1.12E-05
ENSG00000141401	-1.3842589	0.00300048
ENSG00000141441	1.38468805	0.00027385
ENSG00000141448	1.23328694	0.00090196
ENSG00000141480	-1.3096671	0.00063516
ENSG00000141503	-1.2240819	3.94E-06
ENSG00000141526	1.42165602	8.39E-11
ENSG00000141560	-1.2490227	2.94E-05
ENSG00000141577	-1.2221145	0.00083616
ENSG00000141655	-1.4009623	2.35E-05
ENSG00000141664	-1.2636503	0.0000104
ENSG00000141668	1.86601992	6.32E-05
ENSG00000141858	1.56890144	3.08E-12
ENSG00000142279	1.62525634	1.11E-09
ENSG00000142408	1.29090399	0.00235629
ENSG00000142556	1.30017503	1.42E-05

Gene ID	FC 0NG - 4HG	adj p-value 0NG-4HG
ENSG00000142599	-1.2018627	1.04E-05
ENSG00000142627	-1.3446552	3.67E-08
ENSG00000142661	1.51781117	0.00010001
ENSG00000142871	1.2095668	0.00047398
ENSG00000142875	1.24199383	0.00266966
ENSG00000142945	-1.4202665	0.00010052
ENSG00000143153	1.53175887	1.26E-10
ENSG00000143319	1.25323962	1.79E-06
ENSG00000143322	1.29301106	2.57E-08
ENSG00000143341	1.53187967	6.72E-05
ENSG00000143344	-1.5041823	2.84E-11
ENSG00000143369	-1.3024653	0.00325795
ENSG00000143375	-1.3266259	0.0035939
ENSG00000143409	-1.4014889	3.37E-05
ENSG00000143458	-1.2395372	0.00278031
ENSG00000143469	1.34100773	0.01900241
ENSG00000143476	-1.3483283	0.00231105
ENSG00000143479	1.43948534	5.05E-06
ENSG00000143578	-1.3325605	0.00177669
ENSG00000143622	1.38760682	1.28E-09
ENSG00000143643	-1.2226319	0.00287703
ENSG00000143772	-2.4764431	1.42E-19
ENSG00000143801	-1.2600781	0.00174486
ENSG00000143816	-2.4976432	4.58E-10
ENSG00000143851	1.53351532	0.0006551
ENSG00000143878	1.46192374	9.32E-06
ENSG00000144040	-1.2190001	0.00248448
ENSG00000144063	1.61739131	1.10E-11
ENSG00000144136	1.32124881	1.04E-08
ENSG00000144161	1.28026171	0.00245689
ENSG00000144199	-1.2145091	0.03965391
ENSG00000144320	1.2302223	0.00248679

Gene ID	FC 0NG - 4HG	adj p-value 0NG-4HG
ENSG00000144354	-1.3422531	3.55E-05
ENSG00000144366	2.10852797	1.27E-13
ENSG00000144554	-1.2349014	0.00610766
ENSG00000144560	-1.2591552	7.47E-07
ENSG00000144566	1.22091996	5.21E-05
ENSG00000144644	1.54856339	0.00018921
ENSG00000144645	-1.265878	1.13E-07
ENSG00000144647	-1.2099976	0.01515691
ENSG00000144655	-1.2497671	0.00036902
ENSG00000144677	-1.4417481	1.71E-10
ENSG00000144712	-1.3670809	8.65E-06
ENSG00000144749	-1.9006859	1.20E-14
ENSG00000144802	1.46846382	0.01187869
ENSG00000144824	-1.5498857	8.79E-10
ENSG00000144893	1.46520884	2.43E-08
ENSG00000144935	1.21744495	0.04078986
ENSG00000145014	-1.5003197	7.12E-11
ENSG00000145386	-1.3494011	2.61E-05
ENSG00000145391	1.38499308	1.68E-11
ENSG00000145414	1.30059411	5.68E-06
ENSG00000145555	1.25638254	3.45E-07
ENSG00000145632	-1.2232483	0.00387613
ENSG00000145675	1.29079717	0.00053934
ENSG00000145723	1.32827097	0.00275336
ENSG00000145740	1.31318818	0.00020686
ENSG00000145794	-1.2883157	0.01137189
ENSG00000145817	1.77734861	4.12E-12
ENSG00000145860	1.28798432	3.12E-08
ENSG00000145911	-3.2050462	7.11E-19
ENSG00000145916	-1.584259	6.59E-10
ENSG00000145982	-1.2190498	0.00028968
ENSG00000146021	-1.8230196	5.59E-11

Gene ID	FC 0NG - 4HG	adj p-value 0NG-4HG
ENSG00000146054	-1.297376	0.00005676
ENSG00000146094	-1.6029169	3.51E-05
ENSG00000146143	-1.2406603	0.00209927
ENSG00000146232	1.57803405	1.45E-10
ENSG00000146242	1.32836584	7.76E-08
ENSG00000146373	1.20018639	0.03267575
ENSG00000146374	2.01947214	1.03E-07
ENSG00000146386	1.33777998	2.23E-08
ENSG00000146414	1.25658771	0.00130679
ENSG00000146587	1.28353176	0.0091388
ENSG00000146670	-1.4328749	6.8381E-05
ENSG00000146676	1.34542092	0.00058466
ENSG00000146776	-1.3491734	1.84E-05
ENSG00000146830	-1.24319	0.00267872
ENSG00000146834	-1.4641068	4.24E-11
ENSG00000146918	-1.2155541	0.00354618
ENSG00000146950	-1.3987227	1.45E-08
ENSG00000147119	-1.4655592	3.07E-08
ENSG00000147130	-1.3261763	6.19E-07
ENSG00000147180	1.24239696	0.03093612
ENSG00000147408	-1.2390991	0.02573279
ENSG00000147454	-1.2190729	0.00191734
ENSG00000147536	-1.2400512	0.00663511
ENSG00000147642	-1.2728601	0.03842378
ENSG00000147650	1.47372257	4.04E-08
ENSG00000147852	-1.4133035	0.0006696
ENSG00000147854	-1.2183888	5.76E-06
ENSG00000148143	1.21160534	0.00449219
ENSG00000148219	-1.2881727	0.00774594
ENSG00000148400	-1.20883	7.73E-05
ENSG00000148429	-1.2312792	1.50E-06
ENSG00000148468	1.35022397	1.53E-14

Gene ID	FC 0NG - 4HG	adj p-value 0NG-4HG
ENSG00000148660	-1.3150412	2.23E-08
ENSG00000148677	1.79829518	7.44E-15
ENSG00000148737	1.23297258	3.50E-05
ENSG00000148773	-1.456701	4.93E-06
ENSG00000148848	1.37723087	0.00820978
ENSG00000148926	-2.7084881	6.24E-15
ENSG00000148943	1.28315448	0.00020963
ENSG00000149150	-1.3233524	0.00126983
ENSG00000149177	1.30316754	1.15E-06
ENSG00000149313	1.25745881	0.00451699
ENSG00000149503	-1.4726332	3.75E-07
ENSG00000149591	1.45824784	2.77E-06
ENSG00000149636	-1.2947721	1.7133E-05
ENSG00000149679	-1.2125134	0.00180139
ENSG00000149798	-1.2658942	2.65E-07
ENSG00000149929	-1.254966	0.00581955
ENSG00000149948	1.47878498	9.27E-07
ENSG00000150347	2.0388832	1.66E-08
ENSG00000150457	-1.2494086	1.12E-06
ENSG00000150630	2.18858848	3.41E-17
ENSG00000150764	1.63912907	1.07E-09
ENSG00000150787	1.30999858	3.46E-05
ENSG00000151012	1.96938213	2.58E-10
ENSG00000151014	1.30363512	4.67E-08
ENSG00000151023	1.52192893	0.00022317
ENSG00000151093	1.22840793	0.03178254
ENSG00000151135	1.22137139	0.00785169
ENSG00000151136	2.32630785	1.16E-10
ENSG00000151151	1.51227801	0.00072928
ENSG00000151229	1.46031811	0.00017637
ENSG00000151239	1.25038137	0.01165109
ENSG00000151247	1.49851969	9.93E-09

Gene ID	FC 0NG - 4HG	adj p-value 0NG-4HG
ENSG00000151304	1.24861135	0.00226606
ENSG00000151414	1.57705892	9.57E-07
ENSG00000151458	1.54917026	7.69E-09
ENSG00000151474	-1.2091976	9.30E-13
ENSG00000151503	-1.2274818	0.00018665
ENSG00000151576	1.22252888	1.04E-05
ENSG00000151689	-1.302843	8.53E-07
ENSG00000151692	-1.3438215	0.00960012
ENSG00000151702	-1.2104578	1.79E-07
ENSG00000151718	1.66326275	1.95E-20
ENSG00000151725	-1.3291748	0.00031622
ENSG00000151748	1.48502634	3.10E-09
ENSG00000151835	1.45176998	0.00176959
ENSG00000151929	1.21187426	1.54E-05
ENSG00000151967	-1.5489555	1.16E-05
ENSG00000152213	-1.2697556	0.0003494
ENSG00000152219	1.22483613	0.027552
ENSG00000152229	1.26547544	0.00466855
ENSG00000152256	1.34535814	1.09E-06
ENSG00000152284	-1.4829811	2.85E-08
ENSG00000152413	1.31981327	0.00090179
ENSG00000152465	1.4195601	8.06E-10
ENSG00000152518	-1.3462664	7.22E-08
ENSG00000152558	1.34193154	3.5368E-05
ENSG00000152601	1.23387605	0.00040768
ENSG00000152689	1.89139703	3.20E-11
ENSG00000153006	1.22359685	0.02909997
ENSG00000153037	1.34254579	0.00902914
ENSG00000153048	-1.2327977	0.00078913
ENSG00000153071	1.26832068	1.52E-10
ENSG00000153094	-1.7623544	1.32E-06
ENSG00000153162	1.27817393	3.03E-10

Gene ID	FC 0NG - 4HG	adj p-value 0NG-4HG
ENSG00000153165	1.35777782	0.00300426
ENSG00000153201	1.44043502	2.90E-06
ENSG00000153208	-1.6485763	1.69E-09
ENSG00000153250	1.23389855	0.00010947
ENSG00000153391	-1.2238163	0.00074387
ENSG00000153721	1.25619856	0.00860304
ENSG00000153814	1.7267632	4.07E-11
ENSG00000153885	1.48749004	1.78E-10
ENSG00000153989	1.28254813	0.00266194
ENSG00000154016	1.33641315	5.49E-05
ENSG00000154102	-1.2343061	0.0016821
ENSG00000154122	1.63235667	1.33E-12
ENSG00000154127	1.57698563	4.10E-14
ENSG00000154174	1.2129268	0.00252048
ENSG00000154217	1.41384242	7.06E-05
ENSG00000154309	-1.2615661	0.02795
ENSG00000154380	1.55171601	1.48E-17
ENSG00000154553	1.29692582	0.00010015
ENSG00000154608	1.28057954	0.0014523
ENSG00000154639	-1.3767571	0.0069221
ENSG00000154642	1.2940489	0.03788652
ENSG00000154734	3.38549248	1.07E-13
ENSG00000154864	-1.22463	0.00040296
ENSG00000154917	-1.2461431	0.02841704
ENSG00000154920	-1.3622375	0.00086962
ENSG00000154930	-1.3886871	0.00022464
ENSG00000155011	1.73846279	2.74E-07
ENSG00000155034	-1.272632	8.62E-05
ENSG00000155096	1.24386295	0.01084566
ENSG00000155097	1.26482884	0.00094889
ENSG00000155307	1.38258448	0.00126097
ENSG00000155330	1.25898817	0.00402902

Gene ID	FC 0NG - 4HG	adj p-value 0NG-4HG
ENSG00000155438	1.25562507	0.00026644
ENSG00000155465	-1.2828685	5.56E-05
ENSG00000155850	1.21774096	0.00179811
ENSG00000155868	1.25825636	0.00186074
ENSG00000155966	1.31069628	0.0037881
ENSG00000155970	1.33687736	0.0025293
ENSG00000155975	1.35090892	6.36E-08
ENSG00000156011	1.22392467	0.00336585
ENSG00000156017	1.25833163	0.04374268
ENSG00000156030	-1.4204301	3.13E-08
ENSG00000156049	1.4082839	0.00131118
ENSG00000156381	-1.2893293	9.47E-06
ENSG00000156463	-1.5633815	2.07E-05
ENSG00000156466	-1.2964521	0.04999761
ENSG00000156502	1.22770491	0.0022941
ENSG00000156509	-1.2515133	0.01656352
ENSG00000156521	-1.2228716	0.0213242
ENSG00000156671	1.37164585	8.53E-06
ENSG00000156675	-1.5136943	0.00010274
ENSG00000156876	-1.2730562	0.0330805
ENSG00000156970	-1.4504829	5.77E-06
ENSG00000157111	-1.4702781	1.45E-07
ENSG00000157168	1.48842842	2.66E-10
ENSG00000157181	1.24663363	0.01768597
ENSG00000157214	1.62635043	1.56E-07
ENSG00000157240	1.98568644	1.40E-10
ENSG00000157404	-5.1371282	2.23E-17
ENSG00000157456	-1.2703435	0.00037489
ENSG00000157483	1.62944487	7.39E-17
ENSG00000157510	-1.4824997	1.08E-13
ENSG00000157554	-1.2235358	3.22E-09
ENSG00000157557	-1.6158659	6.91E-18

Gene ID	FC 0NG - 4HG	adj p-value 0NG-4HG
ENSG00000157617	-1.6489085	5.44E-14
ENSG00000157933	1.3520582	1.36E-12
ENSG00000158106	-1.3653199	0.01443395
ENSG00000158169	-1.2379259	0.00147533
ENSG00000158186	1.26663188	0.00255867
ENSG00000158195	-1.4515791	2.57E-11
ENSG00000158352	1.33811745	4.42E-09
ENSG00000158373	-1.3485821	0.0006382
ENSG00000158402	-1.392747	5.08E-05
ENSG00000158406	-1.391695	3.09E-05
ENSG00000158458	-1.3303712	0.00095363
ENSG00000158555	-1.3898025	4.06E-10
ENSG00000158716	1.46582143	9.93E-07
ENSG00000158769	-1.2071618	6.33E-05
ENSG00000158966	1.5595502	4.31E-10
ENSG00000159167	-1.9214901	4.91E-07
ENSG00000159231	-1.3581308	6.86E-07
ENSG00000159259	-1.2580299	0.00335615
ENSG00000159388	-1.3155897	5.07E-07
ENSG00000159399	1.23080784	8.15E-06
ENSG00000159433	-1.4213299	2.55E-05
ENSG00000159459	1.21482337	0.00143131
ENSG00000159733	-1.6665628	3.89E-06
ENSG00000159784	1.3826477	0.01152169
ENSG00000159788	-1.2535791	1.55E-08
ENSG00000159840	1.26690249	3.85E-07
ENSG00000159884	-1.2526988	0.0075127
ENSG00000160007	-1.2788795	4.02E-09
ENSG00000160013	-2.0471156	3.35E-09
ENSG00000160062	1.27591568	0.0114119
ENSG00000160179	-1.8285382	3.41E-09
ENSG00000160193	1.24025796	8.49E-06

Gene ID	FC 0NG - 4HG	adj p-value 0NG-4HG
ENSG00000160223	-1.2636325	0.00121577
ENSG00000160271	-1.4839136	3.98E-07
ENSG00000160293	-1.2010676	1.9748E-05
ENSG00000160298	-1.4060988	0.00592241
ENSG00000160299	-1.2698838	5.69E-06
ENSG00000160336	1.21496983	0.01409019
ENSG00000160447	-1.3371211	8.89E-08
ENSG00000160539	-1.2966997	0.02991439
ENSG00000160570	-1.2555519	0.00102946
ENSG00000160703	-1.2721726	0.00023563
ENSG00000160712	-1.6284403	2.09E-07
ENSG00000160796	-1.5084226	3.34E-10
ENSG00000160799	-1.2566097	9.43E-05
ENSG00000160813	-1.2595634	0.0003664
ENSG00000160949	-1.4803346	1.64E-06
ENSG00000160957	-1.376944	0.00801211
ENSG00000161277	-1.2525195	0.02222099
ENSG00000161558	-1.2413457	0.01960116
ENSG00000161653	-1.5088261	3.93E-05
ENSG00000161714	-1.3051947	4.44E-05
ENSG00000161800	-1.3570746	3.92E-05
ENSG00000161813	1.23481566	0.00046561
ENSG00000161835	1.58032187	2.60E-09
ENSG00000161888	-1.256745	0.00150235
ENSG00000161940	-1.4761416	1.07E-12
ENSG00000162032	-1.252346	0.00079732
ENSG00000162063	-1.4945921	2.08E-06
ENSG00000162066	-1.2815611	0.00053375
ENSG00000162073	-1.2957726	4.39E-06
ENSG00000162367	-1.2275697	1.29E-07
ENSG00000162368	1.22254764	0.02370739
ENSG00000162390	1.44726166	3.01E-05

Gene ID	FC 0NG - 4HG	adj p-value 0NG-4HG
ENSG00000162407	2.52799911	1.63E-13
ENSG00000162434	1.3119724	1.67E-10
ENSG00000162437	1.40687613	1.42E-06
ENSG00000162496	-1.6054707	3.08E-07
ENSG00000162522	-1.8695173	2.14E-16
ENSG00000162542	-1.2942864	0.0017144
ENSG00000162551	1.26815429	0.0065724
ENSG00000162614	1.52746665	4.44E-08
ENSG00000162616	1.31206671	0.00477539
ENSG00000162618	1.29411617	0.01745563
ENSG00000162623	1.26605622	0.00034091
ENSG00000162636	1.39445354	0.00013887
ENSG00000162692	5.11414986	8.21E-19
ENSG00000162695	1.44742029	9.07E-05
ENSG00000162702	1.21810446	0.00097531
ENSG00000162733	-1.2019166	4.79E-07
ENSG00000162746	1.36915432	0.00079926
ENSG00000162757	1.2226177	0.04462075
ENSG00000162783	-1.3773685	1.59E-07
ENSG00000162817	-1.4829176	1.27E-09
ENSG00000162878	1.27750408	0.03796981
ENSG00000162980	1.31403604	0.01275461
ENSG00000163050	-1.4142575	0.00029641
ENSG00000163083	-1.2683152	0.03325228
ENSG00000163110	1.59960739	9.98E-19
ENSG00000163162	-1.253167	4.52E-06
ENSG00000163171	-2.1685966	5.62E-19
ENSG00000163219	-1.5894019	0.00220968
ENSG00000163235	2.12733687	2.02E-10
ENSG00000163251	-1.4610667	9.05E-05
ENSG00000163291	1.27632211	0.00142842
ENSG00000163293	1.35503608	0.02424779

Gene ID	FC 0NG - 4HG	adj p-value 0NG-4HG
ENSG00000163297	1.29928325	1.20E-06
ENSG00000163491	-1.3325785	0.03012646
ENSG00000163516	-1.244108	0.00277164
ENSG00000163527	1.24949455	0.00117499
ENSG00000163558	1.20975294	0.00597353
ENSG00000163577	1.3495914	0.00272343
ENSG00000163590	-1.2390965	0.04728484
ENSG00000163637	1.44644273	2.49E-06
ENSG00000163638	-1.4964	7.37E-08
ENSG00000163644	-1.2192511	0.00964283
ENSG00000163659	-1.2563101	9.48E-08
ENSG00000163734	1.22164824	0.01594143
ENSG00000163820	-1.2283833	8.26E-07
ENSG00000163840	-1.3758769	2.43E-05
ENSG00000163918	-1.3138307	0.00241347
ENSG00000163935	1.48848106	0.00011771
ENSG00000163947	-1.3001545	7.99E-05
ENSG00000164022	1.26803612	1.84E-06
ENSG00000164023	1.21456393	0.01035962
ENSG00000164024	1.25803989	8.79E-07
ENSG00000164035	1.33304947	0.00616795
ENSG00000164038	1.28550081	7.39E-07
ENSG00000164056	1.32290678	0.03920756
ENSG00000164080	1.23005835	3.15E-08
ENSG00000164081	-1.2046971	0.00057615
ENSG00000164087	-1.3798085	0.00013787
ENSG00000164088	-1.2231675	0.00626125
ENSG00000164104	-1.5957987	6.77E-09
ENSG00000164125	1.3385629	0.00036292
ENSG00000164134	1.30954155	0.00093749
ENSG00000164161	1.29858887	1.32E-06
ENSG00000164197	1.23438593	0.04218741

Gene ID	FC 0NG - 4HG	adj p-value 0NG-4HG
ENSG00000164211	1.40402086	0.00758079
ENSG00000164220	1.31847475	0.03328375
ENSG00000164283	1.93125531	9.04E-13
ENSG00000164292	1.56281455	3.70E-11
ENSG00000164294	1.21905213	0.00305311
ENSG00000164300	1.21768325	0.00064743
ENSG00000164338	1.30507391	1.54E-06
ENSG00000164442	1.27352778	0.01674114
ENSG00000164484	1.28582108	3.47E-05
ENSG00000164506	1.38125195	9.87E-08
ENSG00000164512	1.82558996	1.69E-07
ENSG00000164532	1.28256905	0.00252476
ENSG00000164604	1.24386194	0.02032963
ENSG00000164649	-1.4294438	6.02E-15
ENSG00000164683	1.89032469	6.79E-07
ENSG00000164684	2.75935298	3.56E-16
ENSG00000164736	1.6560197	3.11E-13
ENSG00000164823	1.94915123	1.69E-13
ENSG00000164849	-1.7050507	0.00058871
ENSG00000164867	-1.462353	1.17E-08
ENSG00000164933	1.32720104	3.38E-05
ENSG00000164976	-1.2028518	0.00130322
ENSG00000164985	-1.2186705	0.00012085
ENSG00000165030	1.38805957	0.00011357
ENSG00000165138	-1.2172145	3.56E-06
ENSG00000165156	1.22698128	0.00156414
ENSG00000165233	-1.2697017	2.39E-05
ENSG00000165240	1.22465778	0.00622281
ENSG00000165244	-1.260079	0.01707202
ENSG00000165259	1.24236585	0.01980416
ENSG00000165271	1.30694941	1.05E-07
ENSG00000165272	-1.2659645	0.01980284

Gene ID	FC 0NG - 4HG	adj p-value 0NG-4HG
ENSG00000165304	-1.3303951	3.25E-06
ENSG00000165338	1.64072047	9.26E-06
ENSG00000165417	1.21170878	0.01267481
ENSG00000165424	-1.2460828	0.00273718
ENSG00000165434	1.36330466	8.75E-05
ENSG00000165480	-1.356732	0.00225781
ENSG00000165494	-1.2150458	0.00011641
ENSG00000165507	-1.8095505	3.6101E-05
ENSG00000165644	-1.2195916	0.01861864
ENSG00000165650	1.21937745	0.00061336
ENSG00000165732	1.47511376	1.11E-10
ENSG00000165757	-1.4102788	7.52E-16
ENSG00000165801	1.42483125	0.00030255
ENSG00000165804	-1.2405433	0.04176228
ENSG00000165806	-1.3627821	4.07E-14
ENSG00000165832	1.21311667	0.00249093
ENSG00000165886	-1.3560141	3.46E-08
ENSG00000165891	1.32602288	1.75E-05
ENSG00000165895	1.4886951	1.40E-05
ENSG00000165898	-1.2736985	0.00360768
ENSG00000165899	1.3486752	0.00046186
ENSG00000165959	-1.4474222	7.23E-08
ENSG00000165997	1.32719729	0.03179644
ENSG00000166068	1.32210107	3.24E-06
ENSG00000166073	1.33547359	1.74E-06
ENSG00000166140	-1.2840089	0.00051582
ENSG00000166169	-1.3331211	0.00010935
ENSG00000166200	1.3715213	0.00642489
ENSG00000166225	1.2166342	0.02012593
ENSG00000166289	-1.710069	6.57E-10
ENSG00000166292	1.32359624	0.01995195
ENSG00000166387	-1.2582016	0.04711363

Gene ID	FC 0NG - 4HG	adj p-value 0NG-4HG
ENSG00000166398	1.34550365	2.66E-07
ENSG00000166401	1.60581041	1.17E-16
ENSG00000166439	-1.380408	2.14E-10
ENSG00000166446	1.42815444	8.94E-13
ENSG00000166471	1.52399739	8.97E-08
ENSG00000166503	1.41498497	2.19E-08
ENSG00000166508	-1.2338231	0.00020474
ENSG00000166510	1.95406133	1.15E-07
ENSG00000166741	1.20468955	9.55E-05
ENSG00000166801	-1.44794	1.80E-06
ENSG00000166831	1.30585838	0.00062757
ENSG00000166833	1.20693209	0.00283547
ENSG00000166851	-1.3966092	0.00011912
ENSG00000166881	-1.2229061	0.01032491
ENSG00000166886	1.49039592	5.08E-06
ENSG00000166900	1.35148021	4.13E-10
ENSG00000166924	-1.4350996	0.00050823
ENSG00000166925	-1.7551582	4.59E-14
ENSG00000166938	-1.2961563	1.54E-06
ENSG00000166949	-1.3692468	3.79E-12
ENSG00000166965	-1.3034036	0.00023185
ENSG00000166971	-1.3744965	1.14E-07
ENSG00000167081	1.35745369	2.27E-08
ENSG00000167106	-1.7056399	1.56E-11
ENSG00000167191	-1.7710846	9.11E-09
ENSG00000167202	-1.270584	3.45E-08
ENSG00000167363	-1.4800924	1.1039E-05
ENSG00000167378	1.29650003	3.06E-10
ENSG00000167513	-1.4729384	7.60E-05
ENSG00000167536	-1.3938741	0.00195208
ENSG00000167550	-1.4117643	8.5695E-05
ENSG00000167562	1.27165223	0.00393214

Gene ID	FC 0NG - 4HG	adj p-value 0NG-4HG
ENSG00000167600	-1.5176978	6.15E-05
ENSG00000167635	1.27434873	5.04E-05
ENSG00000167637	1.29091388	0.0099572
ENSG00000167670	-1.4251077	0.0001194
ENSG00000167680	-1.4524187	1.23E-09
ENSG00000167716	-1.2637339	0.01044695
ENSG00000167720	-1.2310846	0.00320312
ENSG00000167779	-1.2339519	0.00693241
ENSG00000167799	-1.2629278	0.00110528
ENSG00000167861	-1.431376	1.33E-06
ENSG00000167895	-1.3429924	0.00701186
ENSG00000167904	1.22109508	0.04780855
ENSG00000167972	-1.3081566	9.37E-08
ENSG00000167977	1.21165469	0.00015489
ENSG00000168010	-1.338319	0.0252271
ENSG00000168016	-1.5037071	3.12E-08
ENSG00000168061	-1.2530851	0.00538406
ENSG00000168067	-1.2118351	0.00081121
ENSG00000168172	1.22159759	0.00015735
ENSG00000168175	1.21394833	5.59E-06
ENSG00000168209	-1.6644769	1.27E-05
ENSG00000168298	-1.4049818	3.72E-06
ENSG00000168309	-1.4832694	3.93E-08
ENSG00000168310	-1.3742608	5.95E-09
ENSG00000168374	1.24291117	2.16E-07
ENSG00000168386	1.96494101	5.71E-10
ENSG00000168461	1.62193529	7.30E-15
ENSG00000168476	-1.2877818	6.93E-06
ENSG00000168497	-2.079981	3.91E-19
ENSG00000168502	1.26639572	7.96E-06
ENSG00000168575	1.39426246	9.60E-10
ENSG00000168672	-1.3632501	3.95E-05

Gene ID	FC 0NG - 4HG	adj p-value 0NG-4HG
ENSG00000168675	1.32970542	0.00315903
ENSG00000168685	1.54101777	0.00590945
ENSG00000168763	-1.3154392	0.00018702
ENSG00000168769	1.36842666	6.37E-11
ENSG00000168792	-1.3272773	2.24E-05
ENSG00000168874	2.3949826	4.05E-12
ENSG00000168917	1.26969142	0.01034176
ENSG00000168994	1.27749947	6.78E-07
ENSG00000169184	-1.6916285	5.70E-06
ENSG00000169220	-1.3051321	0.00530275
ENSG00000169242	-2.3274471	5.56E-10
ENSG00000169247	-1.4030723	0.00176357
ENSG00000169255	-1.4243057	2.76E-06
ENSG00000169371	-1.2117636	0.0064078
ENSG00000169379	1.2495057	0.02121563
ENSG00000169429	2.85947138	1.32E-07
ENSG00000169439	1.32551339	0.00013829
ENSG00000169504	1.28066622	0.00612043
ENSG00000169554	1.56423824	2.67E-16
ENSG00000169583	1.36240413	0.02174863
ENSG00000169594	-1.2156702	7.51E-06
ENSG00000169607	-1.367544	0.00105836
ENSG00000169629	1.34135403	0.00175516
ENSG00000169641	1.21439133	3.34E-10
ENSG00000169679	-1.3220364	0.00023272
ENSG00000169683	-1.2594855	0.00032442
ENSG00000169756	1.32694444	0.00036002
ENSG00000169762	1.23720378	0.0082167
ENSG00000169826	1.24006976	0.00061598
ENSG00000169851	1.59075581	2.37E-06
ENSG00000169902	1.23352066	5.59E-05
ENSG00000169946	1.23710022	9.13E-06

Gene ID	FC 0NG - 4HG	adj p-value 0NG-4HG
ENSG00000169967	1.36627503	2.80E-05
ENSG00000170011	-1.3609982	0.00148096
ENSG00000170017	1.2565831	1.03E-06
ENSG00000170037	-1.2072406	0.00047054
ENSG00000170214	1.36972162	0.00227079
ENSG00000170271	-1.5519286	3.32E-05
ENSG00000170293	1.92492408	4.11E-11
ENSG00000170323	1.41024619	0.0167569
ENSG00000170364	1.26586303	0.00273342
ENSG00000170365	-1.6964548	3.25E-12
ENSG00000170485	-1.3368173	9.33E-10
ENSG00000170537	1.2554331	0.00631412
ENSG00000170558	1.41845938	3.89E-13
ENSG00000170775	2.23695648	7.40E-20
ENSG00000170802	1.26419025	0.02627229
ENSG00000170873	-1.2982228	5.96E-05
ENSG00000170915	-1.2144305	0.00788578
ENSG00000170921	1.47716709	1.47E-07
ENSG00000170962	1.26703507	0.03879777
ENSG00000171056	-1.3692771	2.26E-06
ENSG00000171105	-1.2494729	0.01543264
ENSG00000171130	-1.2240802	0.00351491
ENSG00000171227	-1.4815089	1.47E-05
ENSG00000171246	3.79882576	5.20E-16
ENSG00000171295	1.21720113	0.00925738
ENSG00000171310	1.71820027	3.61E-10
ENSG00000171316	1.38373691	8.35E-08
ENSG00000171345	-1.3085246	0.00140036
ENSG00000171368	-1.226286	0.0034822
ENSG00000171388	-1.6665713	1.02E-07
ENSG00000171435	-1.4436549	1.71E-07
ENSG00000171488	1.46625686	3.38E-07

Gene ID	FC 0NG - 4HG	adj p-value 0NG-4HG
ENSG00000171552	-1.396508	7.58E-10
ENSG00000171604	1.66636037	5.42E-06
ENSG00000171617	1.47298923	1.14E-07
ENSG00000171621	-1.9181069	1.15E-07
ENSG00000171658	1.39598329	0.0014465
ENSG00000171729	-1.442688	6.24E-07
ENSG00000171792	-1.2075434	1.85E-06
ENSG00000171827	1.41552366	0.00112632
ENSG00000171848	-1.2315965	0.00507464
ENSG00000171889	1.31990074	0.00234367
ENSG00000171940	1.31547942	6.55E-07
ENSG00000172031	-1.3778394	0.00207709
ENSG00000172081	-1.2526997	1.76E-07
ENSG00000172115	1.22166636	0.00163279
ENSG00000172159	1.42941604	3.85E-07
ENSG00000172331	1.36708108	0.00025822
ENSG00000172366	-1.2024576	0.01715889
ENSG00000172466	1.2140785	0.00010281
ENSG00000172572	1.26786725	4.32E-05
ENSG00000172578	1.35706896	9.94E-10
ENSG00000172602	1.71153264	3.35E-06
ENSG00000172613	-1.2074038	0.01719631
ENSG00000172716	-1.240708	2.17E-09
ENSG00000172731	-1.3166511	0.00088008
ENSG00000172765	-1.2882332	2.22E-08
ENSG00000172819	1.33400214	0.0005408
ENSG00000173068	-1.309071	6.37E-07
ENSG00000173207	-1.2757984	7.61E-05
ENSG00000173210	-1.278827	1.08E-11
ENSG00000173334	-1.5792733	7.30E-06
ENSG00000173530	-1.2050887	0.00025513
ENSG00000173535	-1.4869452	1.47E-08

Gene ID	FC 0NG - 4HG	adj p-value 0NG-4HG
ENSG00000173542	1.29987642	0.01743956
ENSG00000173548	-1.2883974	3.44E-07
ENSG00000173559	1.39128117	0.0035413
ENSG00000173597	1.57423295	4.13E-05
ENSG00000173599	-1.2283297	0.00172062
ENSG00000173638	-1.2061487	0.00440602
ENSG00000173681	-1.2352088	0.03740257
ENSG00000173868	1.37737667	4.85E-07
ENSG00000173875	1.20854313	0.00102007
ENSG00000173894	-1.2219799	0.00621183
ENSG00000174004	-1.64259	2.53E-06
ENSG00000174010	1.22041577	0.03336269
ENSG00000174059	-1.986666	7.04E-06
ENSG00000174130	-1.2718717	0.01605216
ENSG00000174348	-1.2747509	0.01608408
ENSG00000174370	-1.6136518	4.52E-06
ENSG00000174405	1.2734149	0.04502474
ENSG00000174574	1.22763766	0.00015798
ENSG00000174695	1.27193566	0.02520529
ENSG00000174705	-1.2119112	8.19E-06
ENSG00000174738	1.20812466	0.0136855
ENSG00000174791	-1.3381998	0.00137836
ENSG00000174796	1.29152934	0.01917716
ENSG00000174804	1.336477	4.48E-12
ENSG00000174951	-1.2258214	0.00922205
ENSG00000174953	1.21605054	0.01853099
ENSG00000175040	1.48967664	2.23E-09
ENSG00000175063	-1.3306614	0.00123558
ENSG00000175105	1.5105299	0.0015949
ENSG00000175264	-2.0436664	2.65E-16
ENSG00000175274	-1.3168116	4.75E-06
ENSG00000175348	1.2494354	6.71E-07

Gene ID	FC 0NG - 4HG	adj p-value 0NG-4HG
ENSG00000175352	1.27430665	0.00073857
ENSG00000175426	1.27279138	0.0124124
ENSG00000175471	1.28462684	0.00078597
ENSG00000175567	-1.2369792	0.02229266
ENSG00000175591	-1.2913494	0.01841732
ENSG00000175643	-1.4366541	2.29E-05
ENSG00000175746	1.43352178	4.69E-05
ENSG00000175832	-1.3475884	1.12E-06
ENSG00000175938	-1.423583	0.00024997
ENSG00000176155	-1.4201151	0.00061761
ENSG00000176170	1.38942794	1.63E-07
ENSG00000176244	-1.2591272	0.01767501
ENSG00000176273	1.32156547	0.03231504
ENSG00000176407	1.20707441	5.95E-05
ENSG00000176438	-1.3927242	1.35E-07
ENSG00000176476	-1.2432164	0.02704146
ENSG00000176542	1.20975276	0.00329356
ENSG00000176641	1.41201713	7.70E-06
ENSG00000176692	1.30993454	0.00030242
ENSG00000176697	1.46199911	0.01189132
ENSG00000176749	-1.3475907	0.00091485
ENSG00000176834	1.25878632	1.67E-07
ENSG00000176845	1.37199282	0.00022216
ENSG00000176853	1.26416467	0.00031206
ENSG00000176890	-1.2115609	0.00034375
ENSG00000176907	2.43924834	1.66E-10
ENSG00000176974	-1.3533468	4.55E-09
ENSG00000177000	-1.394759	3.52E-06
ENSG00000177076	-1.2814985	0.00627191
ENSG00000177084	-1.2248773	0.00161581
ENSG00000177119	1.21302386	0.00015704
ENSG00000177191	-1.2603964	0.00965656

Gene ID	FC 0NG - 4HG	adj p-value 0NG-4HG
ENSG00000177337	-1.2201828	0.04646095
ENSG00000177374	-2.2078499	8.99E-12
ENSG00000177383	-1.2020319	0.00645529
ENSG00000177426	-1.4935364	7.04E-11
ENSG00000177464	1.24818489	6.03E-05
ENSG00000177602	-1.5070783	1.44E-05
ENSG00000177606	-1.4979617	8.78E-14
ENSG00000177674	-1.2383143	0.00068389
ENSG00000177679	2.23685469	1.33E-11
ENSG00000177706	1.35824344	1.57E-11
ENSG00000177707	1.20556844	0.00978643
ENSG00000177732	-1.3838899	2.97E-07
ENSG00000177888	1.3760266	0.03984175
ENSG00000178038	-1.4230978	5.9127E-05
ENSG00000178105	1.32068178	6.26E-09
ENSG00000178127	1.26708862	0.03586638
ENSG00000178150	1.37052138	0.00669047
ENSG00000178175	-1.4265307	0.00137468
ENSG00000178209	-1.2606604	0.00105585
ENSG00000178338	1.22083367	0.04032245
ENSG00000178343	1.38339273	9.11E-05
ENSG00000178381	1.25413545	0.01128491
ENSG00000178401	1.33169339	0.01040195
ENSG00000178409	1.37059362	9.93E-08
ENSG00000178445	-1.2944806	0.00101773
ENSG00000178567	1.32118717	0.0030656
ENSG00000178685	-1.3184362	0.00021863
ENSG00000178695	-1.2442596	0.00021801
ENSG00000178718	-1.3791307	3.63E-08
ENSG00000178726	-1.3562396	1.34E-05
ENSG00000178773	-1.2964537	0.01091243
ENSG00000178882	1.64259589	6.89E-06

Gene ID	FC 0NG - 4HG	adj p-value 0NG-4HG
ENSG00000178971	-1.3555083	2.62E-06
ENSG00000178999	-1.4099043	0.0001805
ENSG00000179044	1.32459748	0.00277044
ENSG00000179094	1.27872667	0.0002434
ENSG00000179144	-1.2626836	6.73E-05
ENSG00000179219	1.25888677	3.50E-05
ENSG00000179314	-1.7197661	1.08E-10
ENSG00000179403	2.00492748	2.09E-08
ENSG00000179428	1.40941598	0.00958403
ENSG00000179456	-1.3431886	6.74E-08
ENSG00000179532	-1.2692672	0.01211631
ENSG00000179546	-1.3423356	0.00451352
ENSG00000179630	1.45352361	0.00040496
ENSG00000179750	-1.2477077	0.01678103
ENSG00000179820	1.36166843	3.35E-12
ENSG00000179833	1.24055749	1.91E-05
ENSG00000179862	-1.3403789	0.00488061
ENSG00000179941	1.31605465	0.0124465
ENSG00000179981	-1.522364	6.09E-12
ENSG00000180008	1.21756355	0.04837973
ENSG00000180257	1.25167622	0.0210211
ENSG00000180354	1.35402349	0.00181095
ENSG00000180448	-1.4968783	0.00061685
ENSG00000180488	1.20573133	0.03431799
ENSG00000180573	-1.2722206	0.00054524
ENSG00000180596	-1.3086149	0.0008801
ENSG00000180694	1.36785626	2.25E-05
ENSG00000180776	1.25696041	0.00270785
ENSG00000180884	-1.3806393	2.97E-05
ENSG00000180891	-1.2005831	0.00282086
ENSG00000180938	1.27925797	0.00287949
ENSG00000180957	1.25042904	1.80E-08

Gene ID	FC 0NG - 4HG	adj p-value 0NG-4HG
ENSG00000180998	2.8416166	8.02E-13
ENSG00000181035	-1.3633852	0.00373347
ENSG00000181218	-1.3766299	0.00152088
ENSG00000181472	1.76264742	1.58E-09
ENSG00000181541	2.0116855	5.38E-11
ENSG00000181638	1.2033998	0.03738603
ENSG00000181704	1.24808714	5.13E-05
ENSG00000181744	1.32893755	0.00011748
ENSG00000181751	1.28589977	0.00089847
ENSG00000181754	-1.3342136	0.00501314
ENSG00000181982	-1.2756001	5.65E-07
ENSG00000182022	-1.9421488	1.45E-21
ENSG00000182118	-1.6918899	1.12E-05
ENSG00000182134	-1.2500939	0.00170478
ENSG00000182141	1.29276391	0.01747616
ENSG00000182197	1.71759732	7.44E-24
ENSG00000182253	1.62072639	8.25E-09
ENSG00000182405	1.29822392	0.01501181
ENSG00000182481	-1.2723537	5.04E-08
ENSG00000182541	-1.2818815	3.72E-06
ENSG00000182568	1.41388157	0.00161343
ENSG00000182575	-1.3416439	0.00465559
ENSG00000182796	-1.3459989	0.00328367
ENSG00000182836	1.34290442	0.01861628
ENSG00000183049	1.97796664	7.93E-10
ENSG00000183054	1.44635224	0.00089887
ENSG00000183077	-1.2638623	0.00200578
ENSG00000183090	1.37552468	1.66E-05
ENSG00000183208	-1.4612519	4.86E-05
ENSG00000183340	1.21963431	0.00022508
ENSG00000183354	1.24482047	0.00439184
ENSG00000183386	-1.2364364	0.00074315

Gene ID	FC 0NG - 4HG	adj p-value 0NG-4HG
ENSG00000183496	-1.5102255	5.39E-06
ENSG00000183578	1.48893475	3.74E-09
ENSG00000183580	-1.3245764	5.34E-10
ENSG00000183598	-1.3087594	0.00066167
ENSG00000183624	-1.2242634	0.00013537
ENSG00000183638	-1.3900935	0.00112352
ENSG00000183688	1.26248983	0.00022029
ENSG00000183691	2.3748647	1.96E-10
ENSG00000183763	-1.3384555	0.00799773
ENSG00000183779	-1.2977863	0.00329005
ENSG00000183808	1.28388537	0.00028741
ENSG00000183856	-1.2997921	0.00050453
ENSG00000183873	-1.2487409	0.00229268
ENSG00000184009	1.23580136	1.70E-11
ENSG00000184014	1.22732407	1.21E-10
ENSG00000184113	-2.0004154	1.31E-13
ENSG00000184182	1.22405427	0.03099173
ENSG00000184208	-1.347597	0.0148096
ENSG00000184226	2.33312736	3.91E-13
ENSG00000184260	-1.2690755	0.00257876
ENSG00000184270	-1.5291202	5.95E-05
ENSG00000184304	1.42046053	5.28E-09
ENSG00000184307	2.4652585	1.63E-11
ENSG00000184357	-1.4343147	6.55E-05
ENSG00000184384	1.24482833	0.00033198
ENSG00000184465	-1.3733307	0.00073736
ENSG00000184508	-1.2446758	7.8477E-05
ENSG00000184640	-1.2201086	8.01E-07
ENSG00000184661	-1.4696973	2.55E-05
ENSG00000184678	-1.3387606	0.00013358
ENSG00000184702	1.2708178	0.02910875
ENSG00000184792	-1.2710622	0.00136421

Gene ID	FC 0NG - 4HG	adj p-value 0NG-4HG
ENSG00000184897	-1.2328137	1.16E-05
ENSG00000184916	1.55990834	4.45E-11
ENSG00000185015	1.42459095	5.08E-05
ENSG00000185022	-1.663006	4.02E-13
ENSG00000185033	-1.4674176	1.99E-06
ENSG00000185112	-1.9532793	3.74E-17
ENSG00000185130	-1.4867446	3.00E-05
ENSG00000185187	-1.3057223	3.03E-05
ENSG00000185222	1.21990403	0.00029304
ENSG00000185262	-1.482308	1.66E-09
ENSG00000185269	1.80634415	1.27E-11
ENSG00000185338	-1.7781115	5.17E-07
ENSG00000185361	-1.7581573	9.66E-13
ENSG00000185432	-1.355218	2.18E-07
ENSG00000185453	-1.2102889	0.04524814
ENSG00000185483	1.23364684	0.01519656
ENSG00000185567	-1.2820064	0.00179509
ENSG00000185630	-1.2495307	0.02489993
ENSG00000185697	-1.310448	0.04476781
ENSG00000185716	1.28882796	0.0015945
ENSG00000185728	1.35999258	2.64E-08
ENSG00000185909	-1.2634398	0.01283047
ENSG00000185963	1.24527538	6.51E-08
ENSG00000186020	1.2393025	0.01725193
ENSG00000186130	1.33515856	0.02101207
ENSG00000186185	-1.4840848	3.58E-05
ENSG00000186193	-1.551895	1.87E-09
ENSG00000186340	1.40953978	0.00498702
ENSG00000186432	1.32851531	1.38E-06
ENSG00000186479	-1.4278857	1.77E-05
ENSG00000186577	-1.3665244	6.33E-05
ENSG00000186591	-1.2738588	2.87E-09

Gene ID	FC 0NG - 4HG	adj p-value 0NG-4HG
ENSG00000186635	-1.3155978	1.36E-07
ENSG00000186638	-1.3438238	0.00694368
ENSG00000186642	-1.4641663	3.10E-06
ENSG00000186665	-1.3976823	7.97E-05
ENSG00000186812	1.40008196	0.00118647
ENSG00000186827	-1.2367142	0.01273157
ENSG00000186871	-1.2829842	0.01026814
ENSG00000186918	-1.886742	3.48E-11
ENSG00000186951	-1.282618	0.00070557
ENSG00000186994	-1.2659775	0.00302472
ENSG00000187189	1.27333115	9.00E-07
ENSG00000187266	-1.5137409	2.76E-08
ENSG00000187498	1.21111468	0.00019398
ENSG00000187555	1.3249308	2.14E-12
ENSG00000187605	-1.3135321	3.62E-07
ENSG00000187609	-1.2167532	0.02595144
ENSG00000187634	1.74054537	2.70E-06
ENSG00000187676	1.2011996	0.00340745
ENSG00000187741	-1.2869025	0.00428495
ENSG00000187742	1.21122786	9.96E-06
ENSG00000187764	-2.0717713	1.20E-12
ENSG00000187800	-1.4853921	8.57E-13
ENSG00000187801	1.27301887	0.01697425
ENSG00000187815	1.28726186	0.0158874
ENSG00000187837	-1.3570196	9.45E-05
ENSG00000187840	-1.25078	8.70E-05
ENSG00000187866	1.21571503	0.00509307
ENSG00000187951	-1.4101782	0.00332047
ENSG00000188042	-2.3630154	7.25E-12
ENSG00000188277	-2.0626867	0.00007608
ENSG00000188312	-1.2436013	0.01501146
ENSG00000188315	-1.299959	0.00282394

Gene ID	FC 0NG - 4HG	adj p-value 0NG-4HG
ENSG00000188428	1.61551826	2.90E-07
ENSG00000188486	-1.3005854	0.00011395
ENSG00000188549	-1.3155075	0.00025535
ENSG00000188647	1.20982489	0.03600389
ENSG00000188732	-1.4393916	0.00858688
ENSG00000188811	-1.379113	0.0004182
ENSG00000189067	1.38200269	0.00120742
ENSG00000189079	1.21354484	6.61E-05
ENSG00000189120	-1.240349	0.01219223
ENSG00000189241	1.21227758	1.24E-09
ENSG00000189337	-1.2937351	0.04614519
ENSG00000196072	1.32489818	2.60E-05
ENSG00000196083	1.34416786	9.95E-05
ENSG00000196110	1.35629187	0.00159413
ENSG00000196169	-2.0219064	1.48E-10
ENSG00000196187	-1.3099115	0.00014316
ENSG00000196209	-1.3447016	0.00290597
ENSG00000196233	1.25805096	0.03083128
ENSG00000196352	1.30280028	6.04E-06
ENSG00000196411	1.29902192	3.27E-08
ENSG00000196417	1.29710765	0.00728828
ENSG00000196418	1.27837512	0.0008332
ENSG00000196421	-1.4725568	4.59E-06
ENSG00000196422	-1.2672076	0.00221632
ENSG00000196428	1.24755445	1.39E-06
ENSG00000196449	1.38339955	1.29E-09
ENSG00000196476	-1.2096206	0.01744077
ENSG00000196526	1.27753723	5.85E-12
ENSG00000196576	-1.2212992	3.50E-05
ENSG00000196584	-1.2476805	0.04498121
ENSG00000196591	1.20161462	1.16E-05
ENSG00000196646	1.24987532	0.01007454

Gene ID	FC 0NG - 4HG	adj p-value 0NG-4HG
ENSG00000196730	-1.5654512	9.28E-17
ENSG00000196747	-1.4181387	0.00021497
ENSG00000196781	-1.4641801	1.70E-13
ENSG00000196782	-1.6809809	2.76E-16
ENSG00000196787	-1.3688422	0.0007363
ENSG00000196792	1.40816852	9.30E-09
ENSG00000196812	-1.4344421	0.00435302
ENSG00000196866	-1.3931116	2.8028E-05
ENSG00000196876	-1.4825359	7.64E-07
ENSG00000196890	-1.5730472	1.01E-06
ENSG00000196981	1.25023924	0.03668438
ENSG00000197016	1.34182335	0.00588998
ENSG00000197019	1.32958835	6.54E-06
ENSG00000197061	-1.4124019	4.2308E-05
ENSG00000197119	-1.2030558	0.0221443
ENSG00000197150	-1.2107023	0.00110094
ENSG00000197153	-1.3253598	0.01316601
ENSG00000197170	1.22706399	0.01608824
ENSG00000197238	-1.2927489	0.00045427
ENSG00000197256	-1.4030438	2.59E-12
ENSG00000197261	-1.5874139	6.96E-06
ENSG00000197312	1.20778122	0.00098296
ENSG00000197380	1.74233795	2.98E-06
ENSG00000197409	-1.4321581	9.96E-05
ENSG00000197442	1.27627384	0.00016175
ENSG00000197451	1.21426549	8.02E-11
ENSG00000197461	1.72922302	1.41E-10
ENSG00000197465	1.28600485	0.02382467
ENSG00000197536	-1.3257744	0.00507616
ENSG00000197566	1.25086294	0.01396916
ENSG00000197632	1.49800494	0.00044795
ENSG00000197714	1.23034577	0.0072166

Gene ID	FC 0NG - 4HG	adj p-value 0NG-4HG
ENSG00000197771	1.23338377	3.16E-06
ENSG00000197780	1.44550717	0.00069275
ENSG00000197837	-1.2281054	0.00246634
ENSG00000197852	-1.6767016	4.88E-13
ENSG00000197857	1.43321957	9.21E-06
ENSG00000197860	1.31879268	6.93E-05
ENSG00000197872	-1.2932866	8.57E-09
ENSG00000197903	-1.4068479	0.00013302
ENSG00000197927	1.230838	0.04835413
ENSG00000197928	1.35078472	4.78E-05
ENSG00000197930	1.21178015	0.03004205
ENSG00000197961	1.20523554	0.02575994
ENSG00000198018	1.43131124	5.30E-09
ENSG00000198042	1.42852846	7.92E-11
ENSG00000198060	1.45610601	2.03E-10
ENSG00000198108	4.67400853	1.78E-19
ENSG00000198205	1.33008444	8.37E-06
ENSG00000198208	-1.3626341	0.00210308
ENSG00000198252	1.23527809	0.04949732
ENSG00000198298	1.26000326	0.03063
ENSG00000198342	1.39844994	0.00020581
ENSG00000198346	1.37889937	0.00013351
ENSG00000198355	-1.7251569	4.81E-13
ENSG00000198369	-1.4834877	1.04E-09
ENSG00000198431	1.26581799	9.1637E-05
ENSG00000198435	-1.4469734	4.35E-05
ENSG00000198440	1.28552552	0.016753
ENSG00000198464	1.21078414	0.02408988
ENSG00000198498	1.23553564	0.0047314
ENSG00000198538	1.36707865	8.97E-06
ENSG00000198604	1.45178344	4.88E-10
ENSG00000198624	-1.2022558	0.00459118

Gene ID	FC 0NG - 4HG	adj p-value 0NG-4HG
ENSG00000198642	1.22606306	8.00E-05
ENSG00000198648	1.21818945	0.0026716
ENSG00000198722	-1.2825833	8.90E-11
ENSG00000198743	1.3512372	0.00511327
ENSG00000198753	-1.2364176	0.02355315
ENSG00000198774	1.45669105	0.00014118
ENSG00000198791	1.20442071	0.02437875
ENSG00000198805	1.36423182	2.65E-12
ENSG00000198836	1.20681966	0.00104654
ENSG00000198846	1.42288438	0.00060724
ENSG00000198862	1.26816847	0.0300809
ENSG00000198873	-1.3679854	5.26E-09
ENSG00000198901	-1.2136261	0.00373596
ENSG00000198909	-1.244055	8.02E-09
ENSG00000198929	-1.4424911	0.00026214
ENSG00000198933	-1.2013857	0.00508714
ENSG00000198945	1.24267602	1.60E-08
ENSG00000198959	-1.2122998	3.19E-05
ENSG00000201558	-1.2143971	0.01809638
ENSG00000203326	1.25985892	0.01887008
ENSG00000203485	-1.3539183	1.32E-08
ENSG00000203805	1.64328621	5.35E-07
ENSG00000203814	-1.4195986	2.39E-06
ENSG00000203883	-2.300109	7.16E-11
ENSG00000204131	-1.2704694	2.70E-06
ENSG00000204152	1.2830667	0.00029039
ENSG00000204217	2.06308749	4.76E-15
ENSG00000204403	1.24714939	0.00225734
ENSG00000204406	-1.2592159	0.00054704
ENSG00000204442	1.67815654	9.92E-09
ENSG00000204604	1.37927396	6.66E-08
ENSG00000204611	1.26034315	4.09E-05

Gene ID	FC 0NG - 4HG	adj p-value 0NG-4HG
ENSG00000204634	-1.2548997	4.78E-05
ENSG00000204682	1.45452488	0.00110482
ENSG00000204767	1.30213914	4.17E-08
ENSG00000204856	1.25250946	0.00507114
ENSG00000204859	-1.2482384	0.00498363
ENSG00000204954	1.25405904	0.01092683
ENSG00000204977	1.29538159	0.00280511
ENSG00000204991	-1.2841251	0.00115696
ENSG00000205177	-1.2433314	0.04875791
ENSG00000205213	-1.2676969	0.00377929
ENSG00000205250	1.20174657	0.00020052
ENSG00000205268	1.24060687	0.04053701
ENSG00000205269	-1.5012392	0.00201321
ENSG00000205339	1.27132028	0.00066085
ENSG00000205356	-1.3528181	1.02E-08
ENSG00000205476	-1.4714858	7.26E-10
ENSG00000205683	-1.5201154	8.60E-09
ENSG00000205808	1.24749243	0.01219511
ENSG00000205978	-1.2129406	0.01039927
ENSG00000206417	-1.2438199	0.02527298
ENSG00000206532	1.24768032	0.0058721
ENSG00000206538	2.90729745	6.93E-18
ENSG00000206560	-1.3360447	1.16E-08
ENSG00000211455	1.84001214	3.71E-09
ENSG00000213047	1.36930374	0.00287482
ENSG00000213064	1.36833623	4.53E-06
ENSG00000213160	-1.3655273	0.00470308
ENSG00000213190	1.27577211	0.00028224
ENSG00000213203	-1.446778	9.00E-07
ENSG00000213347	-1.7658998	1.07E-09
ENSG00000213445	-1.2188384	2.36E-05
ENSG00000213516	1.21475615	0.0188665

Gene ID	FC 0NG - 4HG	adj p-value 0NG-4HG
ENSG00000213626	1.65493414	1.67E-06
ENSG00000213672	-1.2784271	0.00093584
ENSG00000213694	-1.4978797	2.48E-12
ENSG00000213853	-1.3062167	7.8369E-05
ENSG00000214063	-1.2436122	2.17E-05
ENSG00000214106	-1.2091517	0.02851627
ENSG00000214357	-1.9827275	4.62E-07
ENSG00000214595	1.34835489	0.00209475
ENSG00000214826	-1.3828203	0.01224799
ENSG00000214944	-1.6943577	2.26E-13
ENSG00000214960	1.30684105	0.01522729
ENSG00000215012	-1.3416511	1.57E-05
ENSG00000215458	-1.2840424	0.03557691
ENSG00000215883	-1.4532641	0.00034464
ENSG00000216775	-1.781698	3.34E-09
ENSG00000220785	-1.4196046	0.01457245
ENSG00000221829	-1.3683674	8.49E-06
ENSG00000221866	1.29727365	0.03400912
ENSG00000221926	-1.4390795	2.45E-10
ENSG00000221963	-1.3772155	9.14E-05
ENSG00000222047	-1.257556	0.03862746
ENSG00000223749	-1.3191936	0.01471599
ENSG00000223764	1.24824043	9.24E-05
ENSG00000224051	-1.2922729	4.21E-05
ENSG00000224383	-1.7089099	0.02743745
ENSG00000224531	1.46014902	1.10E-07
ENSG00000225206	1.34859705	0.00478138
ENSG00000225264	1.31484993	0.00128153
ENSG00000225484	1.27141428	0.00061786
ENSG00000225830	1.50916322	3.77E-10
ENSG00000225968	2.87689474	2.97E-13
ENSG00000226137	-1.2115101	0.00946603

Gene ID	FC 0NG - 4HG	adj p-value 0NG-4HG
ENSG00000226808	1.41020137	3.15E-05
ENSG00000226823	1.26996164	0.01022783
ENSG00000227051	1.23826026	0.00152668
ENSG00000227517	1.34441632	0.00646982
ENSG00000228495	1.29933546	5.32E-06
ENSG00000229729	1.3628236	0.00074778
ENSG00000229953	-1.4572764	8.63E-05
ENSG00000230266	-1.596449	1.05E-06
ENSG00000230438	-1.6995343	5.18E-07
ENSG00000230453	1.39378639	0.01819187
ENSG00000230479	-1.3818386	2.44E-09
ENSG00000230487	-1.3246539	0.00348848
ENSG00000230753	-1.2249294	0.0130733
ENSG00000230896	-1.2091517	0.0207247
ENSG00000231312	-1.2330249	0.02760641
ENSG00000231856	1.26093712	0.01365181
ENSG00000232098	1.22274768	0.02554175
ENSG00000232533	1.27784313	0.00015029
ENSG00000233237	1.39152056	0.01202981
ENSG00000233370	1.40013711	3.92E-06
ENSG00000233450	-1.2449767	0.00122078
ENSG00000233766	-1.3825793	0.02585911
ENSG00000233818	-1.2227985	0.00152168
ENSG00000233822	-1.4557268	3.9056E-05
ENSG00000234072	1.31984926	0.00892741
ENSG00000234373	1.28050555	0.01597764
ENSG00000234912	1.24050724	0.03554676
ENSG00000235257	-1.3051951	0.00052737
ENSG00000235954	-1.2543194	0.02436363
ENSG00000236901	-1.227829	0.04450174
ENSG00000237172	1.28840447	0.0009543
ENSG00000237181	1.30223469	0.00154199

Gene ID	FC 0NG - 4HG	adj p-value 0NG-4HG
ENSG00000237440	1.23786986	0.02243709
ENSG00000239305	1.22882844	0.00202783
ENSG00000239306	1.31519396	5.44E-10
ENSG00000239887	-1.5551211	1.35E-07
ENSG00000240225	1.23129971	0.00084416
ENSG00000240694	-1.6639318	4.10E-14
ENSG00000240891	-1.5287479	5.68E-06
ENSG00000241839	-1.2111008	0.0005175
ENSG00000241978	-1.2771162	0.00168153
ENSG00000244300	1.29027548	0.00026206
ENSG00000244462	1.25444195	1.24E-05
ENSG00000244625	-1.2464209	0.01158193
ENSG00000245213	-1.3309726	0.00156071
ENSG00000245910	1.32803551	0.03344531
ENSG00000248441	-1.3195321	4.69E-05
ENSG00000248610	1.22277515	3.46E-07
ENSG00000249115	-1.2226344	0.00453457
ENSG00000249279	-1.6312431	4.37E-07
ENSG00000249471	1.25631189	0.01512957
ENSG00000249741	1.23501126	0.04401985
ENSG00000250303	1.37942217	7.11E-05
ENSG00000250510	-1.4470226	8.50E-06
ENSG00000250899	-1.3236641	7.14E-06
ENSG00000250903	-1.318558	0.00292011
ENSG00000251348	1.26924514	0.00694111
ENSG00000251493	1.45033596	0.00080628
ENSG00000253276	1.20572466	0.00175943
ENSG00000253368	2.06435245	1.78E-18
ENSG00000253661	1.38687732	0.00036453
ENSG00000253710	1.27103979	0.03011593
ENSG00000253738	1.25459432	0.01288317
ENSG00000253958	1.29883147	0.00340256

Gene ID	FC 0NG - 4HG	adj p-value 0NG-4HG
ENSG00000254004	1.3383939	0.00020364
ENSG00000254087	1.38941358	5.18E-10
ENSG00000254429	-1.2084667	0.02178322
ENSG00000255248	1.5583187	6.81E-06
ENSG00000255583	1.26671155	0.00763617
ENSG00000255690	-1.3181182	0.00657794
ENSG00000256628	1.22790121	0.01571011
ENSG00000257167	-1.5005237	0.00019812
ENSG00000257337	-1.386983	0.04185397
ENSG00000257365	-1.2130453	0.00982052
ENSG00000257923	1.34364429	6.89E-09
ENSG00000258376	1.23129144	0.01567667
ENSG00000259943	1.27137377	0.01156321
ENSG00000260231	1.31197665	0.00117005
ENSG00000260628	-1.3986719	0.00094882
ENSG00000260920	1.45834111	1.51E-05
ENSG00000261253	-1.5705377	5.10E-07
ENSG00000261373	1.34227673	6.41E-06
ENSG00000261572	1.30195622	0.0066339
ENSG00000261737	1.2667828	0.01874644
ENSG00000262001	-1.2352891	0.01048694
ENSG00000262601	1.38645461	0.00132263
ENSG00000263155	-1.5811775	8.17E-06
ENSG00000263528	1.28867552	1.31E-05
ENSG00000265808	1.2430748	2.17E-06
ENSG00000265972	2.11255989	1.65E-14
ENSG00000267002	1.24474135	0.00131937
ENSG00000267045	1.48380519	6.17E-07
ENSG00000267316	1.22356223	0.0108824
ENSG00000267325	1.22712427	4.15E-06
ENSG00000267365	1.56771891	0.00247131
ENSG00000267583	-1.5511804	5.95E-11

Gene ID	FC 0NG - 4HG	adj p-value 0NG-4HG
ENSG00000269343	1.23524845	0.00226087
ENSG00000269906	1.2444022	0.00656546
ENSG00000269973	-1.280982	0.01643695
ENSG00000270157	1.35397855	0.00145564
ENSG00000270504	-1.2551026	0.00098291
ENSG00000272269	1.26364954	0.00241127
ENSG00000272398	1.53351765	1.35E-07
ENSG00000272405	-1.7123833	3.50E-05
ENSG00000272463	-1.2020883	0.00541507
ENSG00000272886	1.32086653	9.12E-09
ENSG00000273542	-1.3305236	0.0434722
ENSG00000273703	-1.3341271	0.0047314
ENSG00000273780	-1.3333173	5.7386E-05
ENSG00000273802	-1.2569136	0.00835097
ENSG00000273850	-1.2527201	0.02334783
ENSG00000273983	-1.4640851	4.98E-05
ENSG00000274016	1.33403649	0.00441564
ENSG00000274180	-1.3499411	0.00235178
ENSG00000274267	-1.409848	0.00059931
ENSG00000274290	-1.3847301	7.22E-05
ENSG00000274618	-1.3425548	0.00604656
ENSG00000274641	-1.4241296	0.00038065
ENSG00000274995	1.47792024	0.00237925
ENSG00000274997	-1.413467	0.00069316
ENSG00000275126	-1.4416081	5.00E-05
ENSG00000275202	1.29605548	0.01549485
ENSG00000275221	-1.4155803	0.00101894
ENSG00000275342	-1.4026363	7.23E-06
ENSG00000275379	-1.3986276	0.0005565
ENSG00000275713	-1.6074084	1.19E-05
ENSG00000275714	-1.2229022	0.00697008
ENSG00000275993	-1.4277184	0.00026635

Gene ID	FC 0NG - 4HG	adj p-value 0NG-4HG
ENSG00000276043	-1.3550852	3.52E-06
ENSG00000276045	-1.2036244	0.00144625
ENSG00000276180	-1.3670299	0.00418166
ENSG00000276368	-1.4550347	0.00012045
ENSG00000276410	-1.411695	0.00024497
ENSG00000276600	1.3095166	0.00344854
ENSG00000276644	1.30341823	0.01544771
ENSG00000276836	-1.3764555	0.00368417
ENSG00000276849	-1.4817131	0.00508323
ENSG00000276903	-1.4786063	0.00015472
ENSG00000276931	1.27774442	0.03512976
ENSG00000277075	-1.4036132	8.0019E-05
ENSG00000277150	-1.3857035	0.00127874
ENSG00000277157	-1.380482	0.00075664
ENSG00000277224	-1.4955876	1.73E-05
ENSG00000277775	-1.6830864	3.82E-07
ENSG00000278272	-1.4577316	7.23E-05
ENSG00000278384	1.26414283	0.01964506
ENSG00000278463	-1.3410924	0.00153724
ENSG00000278588	-1.4203961	0.00015954
ENSG00000278637	-1.2895025	0.00122609
ENSG00000278705	-1.308079	0.00047375
ENSG00000278828	-1.4272256	7.40E-05
ENSG00000279095	1.38653039	1.66E-08
ENSG00000279117	-1.6314754	1.49E-13
ENSG00000279289	1.36786864	0.01854277
ENSG00000279365	-1.2269474	0.00285575
ENSG00000279369	1.2702071	0.01131086
ENSG00000279419	1.27152963	0.00010772
ENSG00000279457	-1.2663624	0.00820183
ENSG00000279496	-1.2403457	0.034858
ENSG00000279822	-1.2390671	0.01427584

Gene ID	FC 0NG - 4HG	adj p-value 0NG-4HG
ENSG00000280088	1.34517044	0.02107514
ENSG00000280152	-1.3516378	0.00332738
ENSG00000280304	-1.2539407	0.0319574
ENSG00000280696	-1.3134129	0.01739653
ENSG00000280832	-1.4212011	0.00072415
ENSG00000280987	1.20138925	0.01181138
ENSG00000281005	1.25551493	0.00816657
ENSG00000281614	-1.2101501	0.04124317
ENSG00000281823	-1.3249776	0.01314324
ENSG00000282057	1.49835428	0.00017456

Supplementary Table 3d. Differentially expressed genes between 0hr normal glucose and 8hr high glucose samples

Ensembl gene IDs, Fold change (baseline-8HG) and FDR adjusted p-values of DEGs

Gene ID	FC 0NG - 8HG	adj p-value 0NG-8HG
ENSG00000000460	-1.3422989	0.00356543
ENSG00000002079	-1.4718249	0.00055918
ENSG00000002822	-1.2145118	0.00055687
ENSG00000003096	1.23726306	0.01790675
ENSG00000003989	1.66685097	5.99E-06
ENSG00000004777	-1.3125158	0.02463045
ENSG00000005238	1.34712609	3.54E-09
ENSG00000006638	1.28611171	0.00419904
ENSG00000006831	1.21739659	1.00E-06
ENSG00000007908	1.67438707	0.04217802
ENSG00000007968	1.40955137	0.00473938
ENSG00000008405	-1.254295	0.00027844
ENSG00000008441	-1.2515386	1.11E-08
ENSG00000010292	-1.5025559	9.25E-14
ENSG00000010318	-1.2924343	0.00956355
ENSG00000010818	-1.3215928	0.00569754
ENSG00000011405	1.38694423	7.43E-06
ENSG00000011422	-1.29831	3.48E-05
ENSG00000011426	-1.5508808	1.14E-08
ENSG00000012232	-1.2073985	2.84E-07
ENSG00000013016	1.29100903	0.00804391
ENSG00000013561	1.20834715	0.00031446
ENSG00000013810	-1.6277019	6.51E-08
ENSG00000014123	1.21300312	0.00111461
ENSG00000019144	1.29655509	5.05E-07
ENSG00000021645	1.82363949	1.24E-09
ENSG00000022567	1.36162359	0.00250323
ENSG00000026950	-1.291131	3.37E-05
ENSG00000029993	-1.317773	7.33E-08
ENSG00000035499	-1.6127273	2.05E-08
ENSG00000035664	-1.2789461	0.04228772
ENSG00000038295	1.86953698	1.29E-09
ENSG00000042088	-1.282664	4.59E-08
ENSG00000046889	1.35665157	9.77E-07
ENSG00000047621	1.23180556	0.00749758
ENSG00000048052	1.71440457	3.57E-05
ENSG00000049192	1.47320851	6.48E-10
ENSG00000049283	-1.371225	0.04481562
ENSG00000049759	1.43940361	5.07E-08
ENSG00000051341	-1.2760732	0.00798864
ENSG00000052749	1.2794298	4.88E-07
ENSG00000053747	-1.2696682	0.000324
ENSG00000054654	-1.3083112	0.01418204
ENSG00000055813	1.26840217	0.01043876
ENSG00000056097	1.22928749	5.39E-08
ENSG00000057019	1.37929546	4.98E-06
ENSG00000057704	-1.2662833	3.53E-05
ENSG00000058085	-1.3388924	0.0008929
ENSG00000058091	1.44349588	1.56E-08
ENSG00000058404	-1.279046	0.01120052
ENSG00000058668	-1.2072679	7.25E-07
ENSG00000058804	-1.2231335	0.02407296
ENSG00000059378	-1.3169239	1.72E-06
ENSG00000060140	1.23979016	4.97E-05
ENSG00000060656	-1.2352338	0.02224249
ENSG00000062725	1.20497011	0.01821159
ENSG00000064692	-1.6162585	0.00093576
ENSG00000064989	1.39865931	0.00286461
ENSG00000065054	1.33145693	2.0367E-05
ENSG00000065135	1.20838673	8.56E-05
ENSG00000065308	-1.3165607	1.34E-08
ENSG00000065618	-1.2689635	0.01167277
ENSG00000065989	1.31159219	0.0004911
ENSG00000066279	-1.5684452	0.00191551
ENSG00000066735	1.29004524	0.00033922
ENSG00000066923	-1.2401164	0.0027338
ENSG00000067208	1.27475174	1.14E-05
ENSG00000067221	1.27703882	0.00053482
ENSG00000067533	1.21915598	0.00043683
ENSG00000067715	1.38080306	0.00095748
ENSG00000068078	1.30379164	0.00098092
ENSG00000068079	-1.2070537	0.02776699
ENSG00000068489	-1.4891171	3.69E-11
ENSG00000069122	-1.2716467	0.02036148
ENSG00000070404	1.28689659	0.0005408
ENSG00000070614	-1.4427637	1.84E-13
ENSG00000070669	-1.4005009	4.05E-06
ENSG00000070759	-1.2113428	0.03820462
ENSG00000070961	1.3271891	0.00050176
ENSG00000071246	-1.5176413	3.84E-05
ENSG00000071539	-1.4229748	4.40E-08
ENSG00000072071	1.34528373	1.7887E-05
ENSG00000072201	-1.3189367	0.00124516
ENSG00000072571	-1.4201112	0.00103969
ENSG00000073008	1.30792932	4.72E-09
ENSG00000073417	1.20491781	2.34E-05
ENSG00000073464	1.27752733	5.57E-05
ENSG00000073711	-1.322461	0.00029902
ENSG00000073921	1.37900129	3.72E-06
ENSG00000074590	1.27503258	0.00822012
ENSG00000074855	1.29478477	0.00238313
ENSG00000075218	-1.5521112	5.85E-07
ENSG00000075702	-1.4203964	0.0001059
ENSG00000075711	-1.3241836	1.62E-06
ENSG00000075790	1.28644279	0.02194241
ENSG00000076382	-1.5294197	2.85E-09

Gene ID	FC 0NG - 8HG	adj p-value 0NG-8HG
ENSG00000077147	1.32418215	0.0130031
ENSG00000077152	-1.234268	0.00139412
ENSG00000078018	1.87029452	7.95E-09
ENSG00000078114	1.33208966	0.01530081
ENSG00000078401	1.40059461	1.57E-07
ENSG00000079257	-1.3132558	0.00023057
ENSG00000079308	-1.2173596	0.0004866
ENSG00000079435	-1.355688	0.00092629
ENSG00000079459	-1.224979	1.10E-05
ENSG00000079616	-1.5821429	1.52E-09
ENSG00000080986	-1.562546	8.16E-07
ENSG00000081059	1.30291756	0.01123409
ENSG00000081189	-1.2309617	0.0003227
ENSG00000081320	-1.3588333	0.00048459
ENSG00000081803	1.20105037	5.77E-07
ENSG00000082126	-1.4127972	1.02E-07
ENSG00000082512	-1.3166196	0.00549072
ENSG00000084073	1.21333089	0.01822536
ENSG00000085117	-1.4146036	1.07E-07
ENSG00000085185	1.26842662	0.00471358
ENSG00000085563	1.31447947	0.00674716
ENSG00000085871	-1.230484	9.35E-06
ENSG00000085999	-1.2889999	0.0332187
ENSG00000087586	-1.5419218	2.66E-09
ENSG00000087842	-1.3836309	2.94E-09
ENSG00000088305	-1.30569	0.00645507
ENSG00000088325	-1.5822774	1.49E-09
ENSG00000088986	1.2502747	6.73E-10
ENSG00000089127	-1.4080082	0.04014506
ENSG00000089280	1.20114399	5.45E-07
ENSG00000089685	-1.5129215	1.14E-09
ENSG00000089820	-1.292074	0.00499979

Gene ID	FC 0NG - 8HG	adj p-value 0NG-8HG
ENSG00000090238	-1.3863625	9.44E-06
ENSG00000090530	1.33886792	0.00439198
ENSG00000090889	-1.6807407	8.88E-10
ENSG00000091129	1.79995877	5.55E-15
ENSG00000091137	-1.333972	0.00171684
ENSG00000091483	1.21732559	4.05E-05
ENSG00000091622	1.62047291	0.00032057
ENSG00000091656	1.54017159	4.12E-08
ENSG00000091879	1.85136556	5.62E-16
ENSG00000091972	-1.294411	0.00536402
ENSG00000092108	1.26674741	0.00143664
ENSG00000092531	1.22368381	1.25E-07
ENSG00000092621	-1.5815854	5.26E-09
ENSG00000094631	-1.2528174	5.49E-06
ENSG00000094914	-1.2116431	6.92E-05
ENSG00000095303	-1.2904241	1.42E-06
ENSG00000096433	-1.2608905	6.76E-08
ENSG00000099204	-1.4334375	7.36E-09
ENSG00000099251	-1.2922246	0.02754934
ENSG00000099260	1.21782654	6.81E-06
ENSG00000099282	-1.3462119	0.0001284
ENSG00000099822	1.33877322	0.02350643
ENSG00000099860	1.27539774	0.01830149
ENSG00000099889	-1.3520719	3.22E-05
ENSG00000100027	-1.3238848	0.02176056
ENSG00000100034	-1.2976917	3.74E-09
ENSG00000100100	-1.3680403	0.00212756
ENSG00000100162	-1.3165654	0.00137107
ENSG00000100292	-1.5462514	1.58E-08
ENSG00000100342	-1.3683834	5.45E-05
ENSG00000100368	1.31841972	3.85E-05
ENSG00000100439	-1.2302144	0.0168808

Gene ID	FC 0NG - 8HG	adj p-value 0NG-8HG
ENSG00000100526	-1.4396131	7.73E-06
ENSG00000100629	-1.5591137	4.29E-06
ENSG00000100889	-1.2912164	0.00023656
ENSG00000101003	-1.2867068	0.00472686
ENSG00000101057	-1.3341008	0.00025923
ENSG00000101096	-1.8391564	1.96E-13
ENSG00000101188	-1.4058425	3.87E-07
ENSG00000101224	-1.3593044	3.38E-07
ENSG00000101255	-1.2459455	0.00018127
ENSG00000101265	-1.2264886	0.00287124
ENSG00000101445	-1.3220748	0.0001736
ENSG00000101447	-1.672248	2.50E-09
ENSG00000101665	1.46797243	0.01455767
ENSG00000101670	1.3246965	2.18E-05
ENSG00000101844	1.30405387	3.77E-10
ENSG00000101871	-1.3762743	1.36E-08
ENSG00000101945	-1.2756503	0.00471698
ENSG00000102010	-1.5379247	4.71E-10
ENSG00000102024	1.20413529	2.2302E-05
ENSG00000102384	-1.2616063	0.00978617
ENSG00000102755	1.47358782	6.00E-10
ENSG00000102935	1.31749352	0.03097304
ENSG00000102981	-1.3096653	0.02083364
ENSG00000103018	-1.2577145	9.87E-10
ENSG00000103034	1.21118705	0.01105327
ENSG00000103152	-1.2020048	7.43E-05
ENSG00000103326	1.21424636	6.02E-06
ENSG00000103642	1.20832998	0.01956407
ENSG00000103647	-1.4063743	3.28E-08
ENSG00000104093	1.20357875	0.0407337
ENSG00000104147	-1.3687775	0.00130575
ENSG00000104213	-1.2955877	0.0004932

Gene ID	FC 0NG - 8HG	adj p-value 0NG-8HG
ENSG00000104219	1.23394927	0.00012779
ENSG00000104368	-1.3033967	0.00106738
ENSG00000104369	-1.2099418	0.00271609
ENSG00000104450	-1.2320479	0.02332511
ENSG00000104549	-1.224297	0.00035679
ENSG00000104812	1.27431547	4.08E-06
ENSG00000104863	1.24545348	0.01040832
ENSG00000104889	-1.3448649	0.00046026
ENSG00000104951	1.4311375	0.00048011
ENSG00000105011	-1.291035	0.00095985
ENSG00000105088	1.99617761	1.08E-12
ENSG00000105173	1.46823418	4.76E-06
ENSG00000105327	-1.2069353	0.00535843
ENSG00000105486	-1.3301097	1.63E-08
ENSG00000105497	1.223037	0.00081072
ENSG00000105499	1.35309292	0.00357557
ENSG00000105738	-1.2051186	5.98E-05
ENSG00000105810	1.41165897	8.70E-06
ENSG00000105825	1.49818491	5.48E-14
ENSG00000105851	-1.5496958	3.46E-10
ENSG00000105855	2.1509189	8.62E-13
ENSG00000105887	1.21828288	0.00013288
ENSG00000106070	1.23129147	3.90E-05
ENSG00000106100	-1.2554684	0.00010284
ENSG00000106366	1.24490865	2.39E-07
ENSG00000106404	1.20104226	0.03964119
ENSG00000106537	1.52682783	1.45E-09
ENSG00000106560	-1.234146	0.00963714
ENSG00000106829	-1.2543552	2.18E-06
ENSG00000106852	-1.5985698	5.13E-07
ENSG00000107249	-1.38385	0.00121345
ENSG00000107282	1.27049183	0.02647488

Gene ID	FC 0NG - 8HG	adj p-value 0NG-8HG
ENSG00000107438	-1.2666592	1.30E-07
ENSG00000107560	1.23016521	0.00559157
ENSG00000107562	3.44542457	6.90E-20
ENSG00000107719	-1.5652642	2.77E-07
ENSG00000107984	1.37170533	0.00011422
ENSG00000108106	-1.2325183	0.00080971
ENSG00000108352	1.32665362	0.00170276
ENSG00000108551	1.28083048	0.00191488
ENSG00000108556	1.39167456	0.00036419
ENSG00000108622	-1.2538494	9.57E-09
ENSG00000108691	-1.7350951	7.58E-06
ENSG00000108984	-1.3630884	0.00400421
ENSG00000109062	-1.2657062	8.60E-07
ENSG00000109084	-1.3220913	5.64E-07
ENSG00000109452	-1.2987231	0.00317993
ENSG00000109501	-1.549662	3.25E-09
ENSG00000109674	-1.2561542	0.03828392
ENSG00000109685	-1.2838733	3.34E-09
ENSG00000109805	-1.5077508	3.96E-05
ENSG00000109881	-1.2494638	0.00039202
ENSG00000109906	1.96090145	4.02E-09
ENSG00000110092	1.23137041	6.99E-06
ENSG00000110330	1.30255159	0.0016056
ENSG00000110675	1.57114963	3.81E-06
ENSG00000110888	1.23581303	0.00960109
ENSG00000110921	-1.2406374	0.00078425
ENSG00000110931	-1.2272259	1.55E-08
ENSG00000111057	-1.4182916	7.90E-09
ENSG00000111110	-1.4025542	0.02964324
ENSG00000111145	-1.2831581	6.48E-10
ENSG00000111206	-1.4538361	5.75E-11
ENSG00000111247	-1.293855	0.00132325

Gene ID	FC 0NG - 8HG	adj p-value 0NG-8HG
ENSG00000111331	-1.3983983	7.48E-08
ENSG00000111335	-1.6652465	5.87E-06
ENSG00000111339	1.7240323	2.02E-10
ENSG00000111445	-1.2014886	0.01120119
ENSG00000111581	-1.2102522	0.0003666
ENSG00000111602	-1.2985	8.70E-06
ENSG00000111653	-1.2125112	0.01129708
ENSG00000111665	-1.7352687	1.53E-07
ENSG00000111801	-1.339954	0.00295558
ENSG00000111859	1.36209537	0.00189815
ENSG00000112096	-1.2444806	0.00024871
ENSG00000112137	-1.3395896	7.26E-06
ENSG00000112297	-1.6343283	1.66E-07
ENSG00000112742	-1.4437836	0.00677374
ENSG00000112773	1.71879099	0.0006897
ENSG00000112972	-1.376222	0.00364943
ENSG00000112983	-1.2257756	7.78E-05
ENSG00000112984	-1.7128143	4.19E-12
ENSG00000113161	-1.2669155	0.00555775
ENSG00000113368	-1.4171168	2.93E-10
ENSG00000113522	1.21752251	3.16E-05
ENSG00000113552	-1.2342297	3.26E-08
ENSG00000113555	-2.1724138	2.59E-10
ENSG00000113739	1.25065667	0.00600233
ENSG00000113810	-1.2993008	0.00744159
ENSG00000114166	-1.212933	0.03205702
ENSG00000114480	1.35737294	4.98E-10
ENSG00000114573	1.24616661	1.02E-05
ENSG00000114631	-1.2523773	0.033199
ENSG00000114739	-1.2144964	0.04532145
ENSG00000114796	-1.3330587	0.00053549
ENSG00000114812	-1.5416743	0.00127204

Gene ID	FC 0NG - 8HG	adj p-value 0NG-8HG
ENSG00000115084	1.20067203	0.0216457
ENSG00000115091	1.23031146	1.61E-07
ENSG00000115163	-1.6477155	4.67E-08
ENSG00000115290	1.22451366	0.01518971
ENSG00000115415	-1.2175022	0.00035017
ENSG00000115525	-1.9346459	4.78E-09
ENSG00000115602	2.9122845	1.62E-10
ENSG00000115604	1.30170309	0.00495844
ENSG00000115641	-1.2526277	5.33E-06
ENSG00000115902	-1.2860222	9.82E-06
ENSG00000115935	-1.2068978	3.58E-05
ENSG00000115963	1.20983997	0.0215092
ENSG00000115977	1.21556164	9.20E-05
ENSG00000116062	1.20245147	8.62E-09
ENSG00000116473	1.23783319	1.95E-07
ENSG00000116514	-1.236802	4.49E-05
ENSG00000116574	1.29925974	0.01620539
ENSG00000116711	2.47161872	5.97E-11
ENSG00000116741	-1.2669765	0.01095988
ENSG00000116991	-1.3536235	4.90E-06
ENSG00000117114	1.22864398	3.25E-06
ENSG00000117143	1.2015703	0.00014505
ENSG00000117152	1.34734763	2.05E-05
ENSG00000117226	-1.2173201	5.43E-06
ENSG00000117318	1.380241	2.37E-06
ENSG00000117399	-1.6533126	1.60E-09
ENSG00000117595	1.94123489	2.40E-06
ENSG00000117632	-1.2955578	3.03E-10
ENSG00000117650	-1.7032756	2.04E-09
ENSG00000117724	-1.8014115	3.21E-11
ENSG00000118193	-1.55479	5.28E-07
ENSG00000118242	-1.2570259	0.03012312

Gene ID	FC 0NG - 8HG	adj p-value 0NG-8HG
ENSG00000118263	1.21313079	0.00078414
ENSG00000118402	1.31804002	0.0131751
ENSG00000118503	-1.3403979	0.01109011
ENSG00000118515	1.43309222	5.09E-14
ENSG00000118523	1.37769684	2.57E-12
ENSG00000118596	1.29918234	0.01754432
ENSG00000118777	-1.3036235	0.00455608
ENSG00000118946	2.96561273	7.70E-08
ENSG00000118965	1.20998881	0.00126492
ENSG00000118971	-1.3423818	0.00883778
ENSG00000118985	1.26424599	1.17E-05
ENSG00000119138	1.35166766	5.40E-07
ENSG00000119333	-1.2289768	1.3053E-05
ENSG00000119397	-1.5552504	1.20E-10
ENSG00000119403	-1.3813227	5.05E-09
ENSG00000119522	-1.2666481	1.26E-05
ENSG00000119630	-1.5405754	8.96E-09
ENSG00000119718	-1.2397848	2.35E-06
ENSG00000119729	1.28582266	0.02150841
ENSG00000119900	-1.2221791	0.02222875
ENSG00000119917	-1.3498936	0.0090761
ENSG00000120053	-1.2224286	9.52E-06
ENSG00000120217	1.47306488	9.87E-08
ENSG00000120334	-1.2454224	0.01614224
ENSG00000120457	-1.3086705	0.0003478
ENSG00000120539	-1.2126137	0.01789815
ENSG00000120549	-1.3246949	0.00393911
ENSG00000120647	-1.2706134	4.77E-05
ENSG00000120693	1.50271975	2.32E-05
ENSG00000120699	-1.2240962	0.00111451
ENSG00000120742	1.20131273	0.04505096
ENSG00000120802	-1.2662758	1.95E-05

Gene ID	FC 0NG - 8HG	adj p-value 0NG-8HG
ENSG00000121152	-1.5172709	2.62E-06
ENSG00000121210	-1.209533	6.52E-05
ENSG00000121211	-1.2348762	0.02854714
ENSG00000121621	-1.4183889	0.00393722
ENSG00000121797	-1.2030818	0.02229473
ENSG00000121858	-2.1710695	8.55E-09
ENSG00000121957	-1.6316255	9.78E-10
ENSG00000121966	-1.2796864	0.00119491
ENSG00000122376	1.21977981	0.0301617
ENSG00000122378	-1.2903484	1.05E-09
ENSG00000122483	-1.2720215	0.00991727
ENSG00000122641	1.71528574	1.08E-08
ENSG00000122786	1.27195065	1.61E-09
ENSG00000122861	-1.7520463	3.61E-10
ENSG00000122952	-1.3022161	3.84E-06
ENSG00000122966	-1.7565232	4.58E-12
ENSG00000123080	-1.6095934	1.55E-09
ENSG00000123095	1.33894429	0.016534
ENSG00000123384	-1.233579	0.02739835
ENSG00000123473	-1.2814732	0.00057751
ENSG00000123485	-1.7712396	3.56E-07
ENSG00000123975	-1.4267598	3.13E-06
ENSG00000124191	1.21210689	0.00750122
ENSG00000124216	1.2601202	0.01295381
ENSG00000124444	-1.2296128	0.01020522
ENSG00000124575	-1.5513487	2.06E-07
ENSG00000124610	-1.2470318	0.01134295
ENSG00000124635	-1.4839661	0.00010052
ENSG00000124772	1.3686386	0.0017144
ENSG00000125266	1.49169065	1.63E-06
ENSG00000125347	-1.4176428	0.00088372
ENSG00000125378	1.72844421	1.44E-08

Gene ID	FC 0NG - 8HG	adj p-value 0NG-8HG
ENSG00000125871	-1.2686155	1.69E-05
ENSG00000125965	-1.2357675	0.02979665
ENSG00000125966	1.27268172	0.03527554
ENSG00000125968	1.7594648	6.58E-06
ENSG00000126453	-1.2832389	0.00318372
ENSG00000126562	1.44643165	1.21E-06
ENSG00000126787	-1.396579	0.00086508
ENSG00000126878	-1.4017986	8.92E-07
ENSG00000126882	-1.2806315	0.04569553
ENSG00000127314	1.29265493	0.00745938
ENSG00000127528	1.46112023	6.52E-05
ENSG00000127533	-1.8407942	5.01E-09
ENSG00000127564	-1.2508705	0.03408341
ENSG00000127955	1.21212929	0.03655952
ENSG00000128284	-1.3467892	0.00027886
ENSG00000128294	-1.3018665	1.55E-11
ENSG00000128567	1.33859757	2.01E-08
ENSG00000128590	1.21731956	0.04428326
ENSG00000128641	-1.5751112	2.91E-09
ENSG00000128656	-1.2708026	1.12E-06
ENSG00000128815	-1.2738318	0.00661867
ENSG00000128833	-1.6390185	8.31E-08
ENSG00000128849	-1.5140907	1.03E-10
ENSG00000128917	-1.5610913	6.47E-05
ENSG00000128944	-1.4237092	5.86E-08
ENSG00000129158	-1.229705	0.02994887
ENSG00000129195	-1.5598374	1.02E-07
ENSG00000129465	1.28438339	0.0190521
ENSG00000129467	1.20343698	0.02574683
ENSG00000129480	1.20198845	0.01122114
ENSG00000129484	-1.2487172	0.00033845
ENSG00000129810	-1.4693353	1.19E-05

Gene ID	FC 0NG - 8HG	adj p-value 0NG-8HG
ENSG00000130024	-1.4080795	6.24E-07
ENSG00000130066	1.21302317	0.04046629
ENSG00000130164	-1.3435597	9.95E-06
ENSG00000130201	1.26147244	0.0208697
ENSG00000130270	-1.4262999	9.44E-06
ENSG00000130300	1.35346736	0.04351957
ENSG00000130433	1.39894296	0.00382131
ENSG00000130775	-1.3086523	0.00072526
ENSG00000130827	1.21653615	0.00017194
ENSG00000130830	-1.2936607	4.35E-07
ENSG00000131016	1.37611582	7.60E-10
ENSG00000131370	-1.619147	5.60E-15
ENSG00000131398	1.21544258	0.00094581
ENSG00000131409	2.52013301	9.68E-07
ENSG00000131652	-1.3510926	2.3796E-05
ENSG00000131669	-1.51519	3.42E-11
ENSG00000131724	1.33750852	8.94E-06
ENSG00000131747	-1.7307627	1.02E-08
ENSG00000131831	1.45392045	4.27E-05
ENSG00000131943	1.2108133	0.00107223
ENSG00000132003	-1.3466789	3.37E-05
ENSG00000132470	-1.4027268	0.00015083
ENSG00000132510	-1.2392934	0.00247657
ENSG00000132563	-1.253322	0.03593259
ENSG00000132570	-1.2156646	0.04883921
ENSG00000132622	-1.2597447	0.00498316
ENSG00000132780	-1.2050275	3.64E-05
ENSG00000133056	-1.2741246	0.00171953
ENSG00000133110	1.3655942	8.39E-07
ENSG00000133216	-1.2002248	3.43E-05
ENSG00000133424	1.57774457	1.37E-06
ENSG00000133639	-1.2753344	8.52E-06

Gene ID	FC 0NG - 8HG	adj p-value 0NG-8HG
ENSG00000133657	1.64654753	2.49E-06
ENSG00000133789	1.26134227	2.44E-07
ENSG00000133800	-1.4329589	0.00142695
ENSG00000134057	-1.5667468	5.76E-09
ENSG00000134107	-1.4887135	1.27E-09
ENSG00000134222	-1.8734759	2.81E-09
ENSG00000134245	1.2184884	0.03961259
ENSG00000134291	-1.3209914	1.12E-05
ENSG00000134294	1.46100111	0.0014194
ENSG00000134324	-1.3194243	2.92E-09
ENSG00000134352	1.45355241	6.07E-06
ENSG00000134363	1.45050794	1.14E-05
ENSG00000134508	-1.3924091	2.44E-05
ENSG00000134574	-1.2191565	1.28E-05
ENSG00000134690	-1.6243746	9.65E-08
ENSG00000134769	1.23315446	0.00173178
ENSG00000134802	-1.2163204	0.00041413
ENSG00000134909	-1.2251525	0.0058766
ENSG00000135069	-1.4186372	9.37E-12
ENSG00000135074	-1.5412675	4.21E-09
ENSG00000135119	-1.3825296	0.01119841
ENSG00000135272	-1.247161	0.0021009
ENSG00000135451	-1.5120428	9.00E-05
ENSG00000135476	-1.63109	5.78E-06
ENSG00000135622	-1.2050463	0.01382933
ENSG00000135643	1.23407354	0.04817151
ENSG00000135709	1.32714083	2.49E-05
ENSG00000135842	-1.4627307	5.10E-09
ENSG00000135913	1.22195767	0.00402195
ENSG00000135966	-1.2536889	3.23E-05
ENSG00000136010	-1.356147	4.92E-05
ENSG00000136108	-1.4749813	2.22E-05

Gene ID	FC 0NG - 8HG	adj p-value 0NG-8HG
ENSG00000136122	-1.2753685	0.00415099
ENSG00000136153	-1.4108772	2.69E-09
ENSG00000136205	-1.3164907	2.77E-06
ENSG00000136231	1.20925641	3.8224E-05
ENSG00000136237	-1.2758892	0.00050342
ENSG00000136244	1.4858235	0.00144407
ENSG00000136274	1.33116705	0.01893382
ENSG00000136379	-1.3709404	1.61E-05
ENSG00000136490	1.2049587	0.00274212
ENSG00000136603	1.23769925	0.01451176
ENSG00000136717	-1.4326734	2.47E-08
ENSG00000136732	-1.3540484	0.00029276
ENSG00000136824	-1.2501195	0.00083734
ENSG00000136826	1.30888784	0.00673102
ENSG00000136859	-1.4020778	2.07E-09
ENSG00000136861	-1.4335306	4.57E-10
ENSG00000136869	1.26040705	9.66E-05
ENSG00000137135	-1.4871709	7.81E-06
ENSG00000137177	1.29318784	5.16E-09
ENSG00000137266	-1.2024902	0.02016173
ENSG00000137393	1.25610267	0.00088251
ENSG00000137486	-1.3231852	8.92E-07
ENSG00000137497	-1.2011165	4.64E-07
ENSG00000137522	1.299511	7.55E-06
ENSG00000137727	1.26423052	0.00812295
ENSG00000137767	-1.2687179	0.00628984
ENSG00000137801	1.25656361	6.86E-07
ENSG00000137804	-1.7266372	1.76E-10
ENSG00000137807	-1.6759409	3.33E-10
ENSG00000137812	-1.5771495	1.99E-05
ENSG00000137825	-1.2215179	0.02728186
ENSG00000137831	1.55623725	6.99E-08

Gene ID	FC 0NG - 8HG	adj p-value 0NG-8HG
ENSG00000137834	2.02855711	1.08E-05
ENSG00000137872	1.75609534	6.31E-08
ENSG00000137877	1.37386757	0.00516248
ENSG00000137880	-1.3286963	0.02283392
ENSG00000137941	-1.2176146	0.03643916
ENSG00000138160	-1.421899	0.00035654
ENSG00000138162	-1.4389181	3.19E-06
ENSG00000138180	-1.5014743	1.27E-06
ENSG00000138182	-1.397608	0.02229218
ENSG00000138386	-1.2739993	3.81E-05
ENSG00000138411	1.30858564	7.60E-07
ENSG00000138448	1.43315936	1.43E-06
ENSG00000138449	1.53059769	2.37E-08
ENSG00000138496	-1.298548	0.00271827
ENSG00000138600	1.24773143	0.00633714
ENSG00000138639	1.22356756	6.31E-07
ENSG00000138650	1.37431086	0.00050579
ENSG00000138675	1.33809169	0.00358236
ENSG00000138685	1.244969	0.00867601
ENSG00000138764	-1.5008371	0.0001933
ENSG00000138771	1.2305514	0.01881928
ENSG00000138778	-1.3671238	0.01224779
ENSG00000138796	-1.2917436	0.00053494
ENSG00000139132	1.24217901	0.00940439
ENSG00000139173	1.34948873	0.00129574
ENSG00000139278	1.43776867	1.94E-08
ENSG00000139354	-1.6381815	8.92E-09
ENSG00000139508	-1.3424102	0.01019813
ENSG00000139734	-1.3907948	7.37E-06
ENSG00000139998	-1.2658498	0.00179304
ENSG00000140104	1.26030539	0.0004909
ENSG00000140416	1.56195925	6.72E-17

Gene ID	FC 0NG - 8HG	adj p-value 0NG-8HG
ENSG00000140451	-1.7390864	1.82E-06
ENSG00000140525	-1.2830193	0.00059868
ENSG00000140534	-1.4378611	7.87E-05
ENSG00000140564	1.35785202	8.81E-09
ENSG00000140853	-1.2852464	0.00302178
ENSG00000140873	-1.5428892	2.55E-07
ENSG00000140961	1.21466011	0.00446819
ENSG00000141337	-1.2760774	0.04342549
ENSG00000141441	1.21692084	0.04966919
ENSG00000141458	-1.2068603	0.00012764
ENSG00000141505	-1.2513156	0.0289051
ENSG00000141526	1.34999493	5.16E-09
ENSG00000141542	1.26465127	0.00249286
ENSG00000141560	-1.2412764	4.78E-05
ENSG00000141668	2.65850257	5.86E-09
ENSG00000141858	1.34508413	1.47E-07
ENSG00000142408	1.30060069	0.0016672
ENSG00000142494	-1.2087995	0.0375926
ENSG00000142661	1.40475637	0.00174124
ENSG00000142731	-1.319237	0.00669047
ENSG00000142875	1.26088477	0.00117884
ENSG00000142945	-1.6863696	3.95E-08
ENSG00000143153	1.56034757	3.96E-11
ENSG00000143228	-1.5359371	1.04E-05
ENSG00000143369	-1.2730373	0.00834698
ENSG00000143476	1.27217952	0.01964092
ENSG00000143507	-1.308896	0.00069651
ENSG00000143669	1.27047261	0.01032585
ENSG00000143772	-1.5071711	3.56E-09
ENSG00000143816	-1.6847307	7.68E-05
ENSG00000143819	-1.241983	0.00206141
ENSG00000143850	-1.3060936	0.00045842

Gene ID	FC 0NG - 8HG	adj p-value 0NG-8HG
ENSG00000143878	1.27474312	0.00563622
ENSG00000144063	1.64997139	3.37E-12
ENSG00000144320	1.26146844	0.00058971
ENSG00000144366	1.60763914	2.73E-08
ENSG00000144426	1.26638796	0.01181317
ENSG00000144554	-1.4297433	2.35E-06
ENSG00000144560	-1.2741602	2.55E-07
ENSG00000144642	1.28860879	0.00011861
ENSG00000144644	1.59150491	7.33E-05
ENSG00000144645	-1.3339178	9.04E-10
ENSG00000144677	-1.2182243	0.00010176
ENSG00000144730	-1.2652546	0.02396339
ENSG00000144749	-1.4790768	1.29E-08
ENSG00000144824	-1.3580493	3.12E-06
ENSG00000145147	1.22561839	1.82E-05
ENSG00000145365	-1.2048146	0.0314036
ENSG00000145386	-1.5964491	1.79E-09
ENSG00000145740	1.26035562	0.00186111
ENSG00000145794	-1.2709495	0.01837621
ENSG00000145817	1.28175017	0.00039249
ENSG00000145911	-1.3713779	0.00025691
ENSG00000146021	-1.3682354	8.93E-05
ENSG00000146072	-1.2440952	2.41E-07
ENSG00000146083	-1.2386228	7.09E-05
ENSG00000146143	-1.2125593	0.00711918
ENSG00000146374	1.4763514	0.00243634
ENSG00000146386	1.29384766	3.55E-07
ENSG00000146410	-1.2664656	0.03299717
ENSG00000146587	1.31431735	0.00359902
ENSG00000146670	-1.369881	0.00050855
ENSG00000146678	1.42154637	3.97E-05
ENSG00000146918	-1.4393006	1.02E-07

Gene ID	FC 0NG - 8HG	adj p-value 0NG-8HG
ENSG00000146950	-1.2970391	3.75E-06
ENSG00000147082	1.2635459	0.00199892
ENSG00000147155	-1.2008919	6.80E-06
ENSG00000147202	1.2659637	0.0142685
ENSG00000147408	-1.736394	7.81E-09
ENSG00000147536	-1.2787436	0.00149092
ENSG00000147852	-1.6009688	4.39E-06
ENSG00000148677	1.70559809	1.46E-13
ENSG00000148700	-1.3021886	1.24E-05
ENSG00000148773	-1.7756812	3.03E-10
ENSG00000148926	-1.446019	0.00016657
ENSG00000149503	-1.6348229	1.35E-09
ENSG00000149548	-1.2417953	0.02648219
ENSG00000149564	-1.2096805	5.00E-06
ENSG00000149591	1.34729354	0.00016969
ENSG00000149929	-1.3286792	0.00037764
ENSG00000149948	1.44265422	3.47E-06
ENSG00000150551	1.34365696	5.52E-05
ENSG00000150630	1.32384544	1.94E-05
ENSG00000150764	1.3865619	1.02E-05
ENSG00000150787	1.2169025	0.00310009
ENSG00000151012	1.51575853	1.48E-05
ENSG00000151023	1.44226976	0.00143549
ENSG00000151093	1.23133506	0.02918425
ENSG00000151338	1.25150581	0.00530947
ENSG00000151414	1.60907041	3.84E-07
ENSG00000151458	1.33643756	4.05E-05
ENSG00000151500	-1.2314856	2.14E-06
ENSG00000151503	-1.2050712	0.00070914
ENSG00000151623	1.24892158	0.03141635
ENSG00000151690	-1.4142369	0.00025248
ENSG00000151692	-1.2827223	0.03877238

Gene ID	FC 0NG - 8HG	adj p-value 0NG-8HG
ENSG00000151725	-1.3771255	5.13E-05
ENSG00000151835	1.39115465	0.00677663
ENSG00000151967	-1.4519832	0.00016939
ENSG00000152127	-1.313196	3.66E-11
ENSG00000152213	-1.2208038	0.00341755
ENSG00000152217	1.27022932	0.01007454
ENSG00000152284	-1.2882589	0.00015963
ENSG00000152413	1.22069875	0.02651709
ENSG00000152422	-1.2910017	0.0151926
ENSG00000152465	1.30078182	6.70E-07
ENSG00000152558	1.2636635	0.00106029
ENSG00000152601	1.3980154	7.21E-08
ENSG00000152689	1.31842581	0.00096246
ENSG00000152953	-1.5236693	5.78E-09
ENSG00000153048	-1.2659602	0.00014391
ENSG00000153094	-1.7014114	4.72E-06
ENSG00000153208	-1.4359566	2.59E-06
ENSG00000153391	-1.2233605	0.00076228
ENSG00000153551	-1.353599	7.86E-08
ENSG00000153707	1.48253058	0.00053934
ENSG00000153721	1.2172085	0.03036584
ENSG00000153814	1.26021591	0.00146058
ENSG00000153956	1.26639627	0.00034248
ENSG00000153993	1.60182743	1.24E-06
ENSG00000154102	-1.2362094	0.00153692
ENSG00000154127	1.20535939	8.48E-05
ENSG00000154217	-1.2998753	0.0031682
ENSG00000154310	1.36013561	6.68E-11
ENSG00000154429	-1.3048464	0.02437875
ENSG00000154556	1.53333156	2.60E-06
ENSG00000154639	-1.4253915	0.00226619
ENSG00000154734	1.71417233	4.08E-05

Gene ID	FC 0NG - 8HG	adj p-value 0NG-8HG
ENSG00000154920	-1.2731737	0.01302815
ENSG00000154930	-1.4160626	9.26E-05
ENSG00000155011	1.41254647	0.0009669
ENSG00000155111	-1.3336828	0.00101706
ENSG00000155158	-1.2646351	0.00831552
ENSG00000155307	1.34556662	0.00355391
ENSG00000155324	-1.3006378	0.00074068
ENSG00000155465	-1.2040206	0.00321546
ENSG00000155657	1.40231251	0.00613419
ENSG00000155849	-1.3770408	1.73E-14
ENSG00000155966	1.30366566	0.00471672
ENSG00000156313	1.40943199	2.12E-08
ENSG00000156463	-1.500341	0.0001048
ENSG00000156509	-1.2917395	0.0047247
ENSG00000156587	-1.3319767	3.32E-06
ENSG00000156970	-1.6549305	7.68E-09
ENSG00000157168	1.63068992	7.66E-13
ENSG00000157214	1.39168273	0.00021012
ENSG00000157227	-1.2810159	1.12E-06
ENSG00000157240	1.32853692	0.00322957
ENSG00000157404	-1.7947113	1.59E-05
ENSG00000157456	-1.6654416	4.06E-11
ENSG00000157483	1.2492372	2.62E-07
ENSG00000157510	-1.3310119	9.45E-10
ENSG00000157570	-1.4212993	1.34E-11
ENSG00000157613	-2.5286578	9.41E-10
ENSG00000158186	1.21311374	0.01871475
ENSG00000158195	-1.2647446	1.77E-06
ENSG00000158270	-1.2741765	0.00784122
ENSG00000158352	1.21541776	2.2633E-05
ENSG00000158373	-1.4828355	7.58E-06
ENSG00000158402	-1.743822	7.61E-10

Gene ID	FC 0NG - 8HG	adj p-value 0NG-8HG
ENSG00000158406	-1.5618405	7.54E-08
ENSG00000158555	-1.4532652	1.25E-11
ENSG00000158716	1.3819371	2.44E-05
ENSG00000158856	-1.4305917	4.76E-08
ENSG00000158859	-1.326341	5.84E-05
ENSG00000159167	-1.9484707	3.13E-07
ENSG00000159231	-1.3052463	1.07E-05
ENSG00000159388	-1.2812764	4.00E-06
ENSG00000159399	1.22601208	1.15E-05
ENSG00000159433	-1.2333946	0.01802035
ENSG00000159733	1.35501113	0.0073069
ENSG00000160013	-1.8259402	2.00E-07
ENSG00000160117	1.2561256	0.02553651
ENSG00000160145	-1.2219215	0.04041879
ENSG00000160179	-1.4554025	6.6939E-05
ENSG00000160298	-1.3374129	0.02463174
ENSG00000160685	1.28410856	4.30E-06
ENSG00000160796	-1.2315221	0.00038001
ENSG00000161542	-1.2432226	3.70E-09
ENSG00000161692	-1.4099667	8.12E-05
ENSG00000161800	-1.6277524	1.86E-09
ENSG00000161813	1.26264125	0.00010508
ENSG00000161888	-1.5144685	3.46E-08
ENSG00000161940	-1.3130643	1.56E-08
ENSG00000162063	-1.5157502	1.03E-06
ENSG00000162139	-1.2060267	0.00055464
ENSG00000162337	-1.2813992	5.13E-06
ENSG00000162434	1.33724611	2.60E-11
ENSG00000162437	1.22154943	0.00509307
ENSG00000162496	-1.6294854	1.56E-07
ENSG00000162614	1.68674208	2.40E-10
ENSG00000162616	1.42281782	0.00016621

Gene ID	FC 0NG - 8HG	adj p-value 0NG-8HG
ENSG00000162692	1.83373294	1.06E-06
ENSG00000162695	1.27565011	0.01484305
ENSG00000162733	-1.2268809	4.52E-08
ENSG00000162928	1.21342533	0.00115232
ENSG00000163050	-1.265417	0.02192264
ENSG00000163083	-1.4146786	0.00071711
ENSG00000163110	1.54043193	1.52E-17
ENSG00000163257	-1.2174516	0.00081573
ENSG00000163346	-1.2635777	0.00071037
ENSG00000163428	1.2186248	0.03933603
ENSG00000163527	1.27597568	0.00035081
ENSG00000163535	-1.4872129	0.01881171
ENSG00000163554	-1.3111306	0.01994127
ENSG00000163590	-1.2610718	0.02698121
ENSG00000163633	1.21142867	0.02582614
ENSG00000163661	1.39396972	0.00001076
ENSG00000163814	-1.3985694	9.32E-09
ENSG00000163840	-1.2165741	0.01367779
ENSG00000163884	1.20623978	0.03767794
ENSG00000163923	-1.3068777	0.00482418
ENSG00000163947	-1.2108564	0.00521058
ENSG00000164023	1.31300461	0.00017133
ENSG00000164035	1.34607715	0.00436045
ENSG00000164038	1.23038044	2.92E-05
ENSG00000164045	1.21155466	0.01314324
ENSG00000164056	1.371412	0.01535312
ENSG00000164086	1.22049521	3.38E-05
ENSG00000164087	-1.4868982	3.57E-06
ENSG00000164104	-1.7795797	2.84E-11
ENSG00000164105	-1.249492	0.00182963
ENSG00000164109	-1.4092818	0.00016087
ENSG00000164125	1.22534998	0.01993972

Gene ID	FC 0NG - 8HG	adj p-value 0NG-8HG
ENSG00000164134	1.20397823	0.03729949
ENSG00000164161	1.52287121	1.14E-11
ENSG00000164236	1.24660993	0.01070529
ENSG00000164283	1.74593942	1.04E-10
ENSG00000164292	1.4518056	4.18E-09
ENSG00000164442	1.32910683	0.00342138
ENSG00000164611	-1.3286671	1.05E-07
ENSG00000164663	1.21765874	0.03158654
ENSG00000164736	1.36520268	1.10E-07
ENSG00000164754	-1.2433856	3.29E-08
ENSG00000164823	1.23185956	0.0054963
ENSG00000164951	1.33411279	3.03E-06
ENSG00000164976	-1.222205	0.00043678
ENSG00000164985	-1.297277	7.92E-07
ENSG00000165118	1.25568422	0.00461382
ENSG00000165240	1.22008983	0.00748422
ENSG00000165244	1.38712825	0.00033667
ENSG00000165271	1.24019928	9.33E-06
ENSG00000165272	-1.2545409	0.02692684
ENSG00000165304	-1.3776585	2.98E-07
ENSG00000165480	-1.3512249	0.00264024
ENSG00000165494	1.22541811	5.87E-05
ENSG00000165507	-1.4339091	0.01825777
ENSG00000165806	-1.2332714	2.50E-09
ENSG00000165886	-1.3883329	5.82E-09
ENSG00000165899	1.55201602	5.25E-07
ENSG00000165959	-1.6507032	3.29E-11
ENSG00000166073	1.25733486	0.00011561
ENSG00000166250	1.3518862	0.00057307
ENSG00000166439	-1.3124864	1.57E-08
ENSG00000166503	1.27250592	3.84E-05
ENSG00000166510	1.74672327	5.27E-06

Gene ID	FC 0NG - 8HG	adj p-value 0NG-8HG
ENSG00000166801	-1.3306607	0.00017561
ENSG00000166803	-1.286887	0.00023282
ENSG00000166833	-1.3699881	6.98E-07
ENSG00000166851	-1.7629924	2.18E-09
ENSG00000166912	1.3492032	0.0131958
ENSG00000166974	1.21713765	5.75E-09
ENSG00000167106	-1.3425994	1.60E-05
ENSG00000167191	-1.6332865	3.08E-07
ENSG00000167325	-1.2242006	0.0001008
ENSG00000167363	-1.2893057	0.00537825
ENSG00000167550	-1.4027584	0.00011533
ENSG00000167637	1.27478304	0.01567927
ENSG00000167680	-1.3292467	6.86E-07
ENSG00000167772	1.42644473	0.00126349
ENSG00000167900	-1.2660965	0.00023038
ENSG00000168061	-1.26328	0.00368417
ENSG00000168077	-1.2954827	1.77E-07
ENSG00000168078	-1.4700097	7.96E-05
ENSG00000168298	-1.5651248	8.65E-09
ENSG00000168309	-1.464999	8.27E-08
ENSG00000168374	1.25095885	1.13E-07
ENSG00000168386	1.39251892	0.00078627
ENSG00000168476	-1.3840992	3.38E-08
ENSG00000168874	1.38886882	0.00230045
ENSG00000168906	1.3105627	1.28E-12
ENSG00000169083	-1.2931657	5.69E-06
ENSG00000169242	-1.3112697	0.04759422
ENSG00000169258	-1.2055506	0.0020562
ENSG00000169288	1.25818245	0.0187727
ENSG00000169330	1.27246774	0.00464229
ENSG00000169504	1.23891529	0.0224477
ENSG00000169583	1.34389643	0.03079119

Gene ID	FC 0NG - 8HG	adj p-value 0NG-8HG
ENSG00000169604	-1.2541355	0.00020773
ENSG00000169607	-1.6135614	8.55E-07
ENSG00000169679	-1.6888138	5.15E-10
ENSG00000169744	1.3388444	1.10E-07
ENSG00000169760	1.50734183	4.20E-07
ENSG00000169851	1.32933655	0.00410537
ENSG00000169891	-1.3420792	0.00745938
ENSG00000169914	1.3021772	0.00040769
ENSG00000170011	-1.3926216	0.00058388
ENSG00000170017	1.336152	4.59E-09
ENSG00000170088	1.2258067	0.00237902
ENSG00000170175	-1.3061403	0.0005074
ENSG00000170214	1.34849787	0.00403178
ENSG00000170260	-1.2159037	0.01861121
ENSG00000170271	-1.3333947	0.00897763
ENSG00000170293	1.22220044	0.03354843
ENSG00000170312	-1.6353028	3.09E-06
ENSG00000170323	1.65376917	0.00020112
ENSG00000170325	1.20995193	0.00488705
ENSG00000170365	-1.4519191	3.12E-08
ENSG00000170385	1.30609563	0.01945983
ENSG00000170485	-1.299627	1.17E-08
ENSG00000170525	-1.2753405	2.39E-08
ENSG00000170540	-1.3221351	0.01059389
ENSG00000170558	1.39090821	2.17E-12
ENSG00000170775	1.49377158	1.77E-10
ENSG00000170801	-1.2967125	0.00030205
ENSG00000170873	-1.280507	0.00014112
ENSG00000170962	1.29694695	0.01900902
ENSG00000171033	1.20153103	0.0036903
ENSG00000171056	-1.2669904	0.00029825
ENSG00000171241	-1.3650725	0.00025658

Gene ID	FC 0NG - 8HG	adj p-value 0NG-8HG
ENSG00000171246	2.98585632	2.72E-13
ENSG00000171320	-1.3359905	0.03983588
ENSG00000171388	-1.40945	0.0001878
ENSG00000171488	1.21747284	0.00948548
ENSG00000171608	-1.2030063	0.00163457
ENSG00000171621	-1.3454122	0.01805176
ENSG00000171729	-1.2473201	0.00239744
ENSG00000171791	-1.2612177	0.0483888
ENSG00000171792	-1.2291458	2.82E-07
ENSG00000171848	-1.3982228	4.52E-06
ENSG00000171889	1.4264338	7.67E-05
ENSG00000172031	-1.6161169	3.87E-06
ENSG00000172086	1.31524607	2.18E-05
ENSG00000172159	1.20040002	0.0105796
ENSG00000172331	1.24860114	0.01395035
ENSG00000172456	-1.2048345	0.02518719
ENSG00000172572	1.24540544	0.00015214
ENSG00000172819	1.20645079	0.04097856
ENSG00000172985	-1.412554	2.09E-07
ENSG00000173083	1.22661865	0.00177696
ENSG00000173166	1.21963783	0.01266549
ENSG00000173193	-1.2389197	0.00170334
ENSG00000173207	-1.5020442	1.61E-09
ENSG00000173214	1.22217636	0.02597654
ENSG00000173334	-1.3453423	0.00416132
ENSG00000173456	-1.2672252	6.63E-05
ENSG00000173530	1.27417494	2.84E-06
ENSG00000173535	-1.4265474	1.88E-07
ENSG00000173548	-1.215251	5.25E-05
ENSG00000173559	1.31495465	0.02106641
ENSG00000173597	1.93671033	1.85E-08
ENSG00000173846	1.22276808	0.00715191

Gene ID	FC 0NG - 8HG	adj p-value 0NG-8HG
ENSG00000173852	1.20063597	0.0301775
ENSG00000174059	-1.5862653	0.0027739
ENSG00000174130	-1.2492573	0.02978573
ENSG00000174306	1.25106474	3.88E-06
ENSG00000174348	-1.2565626	0.02633095
ENSG00000174370	-1.5992166	6.49E-06
ENSG00000174705	-1.2431216	6.91E-07
ENSG00000175048	1.33494846	2.29E-05
ENSG00000175063	-1.6530353	5.08E-08
ENSG00000175216	-1.3121292	3.69E-09
ENSG00000175264	-1.5616327	3.61E-10
ENSG00000175274	-1.6172147	6.88E-12
ENSG00000175305	1.38584278	0.00331794
ENSG00000175348	1.25328423	5.01E-07
ENSG00000175471	1.29700484	0.00047349
ENSG00000175544	1.25312312	0.01012563
ENSG00000175567	-1.3046935	0.00255115
ENSG00000175745	1.38109897	5.25E-09
ENSG00000175832	-1.3389263	1.75E-06
ENSG00000175899	1.39692542	0.01580798
ENSG00000175938	-1.3773487	0.00096655
ENSG00000176102	1.32945839	7.79E-06
ENSG00000176182	1.28074355	0.00299316
ENSG00000176194	-1.2261312	0.03034431
ENSG00000176619	-1.203496	1.79E-05
ENSG00000176641	1.2104989	0.01930938
ENSG00000176697	1.44203888	0.01642298
ENSG00000176749	1.29552468	0.00487433
ENSG00000176871	1.20305931	4.08E-07
ENSG00000176890	-1.3555401	6.26E-08
ENSG00000176974	-1.2922087	2.20E-07
ENSG00000177303	1.24666759	1.46E-05

Gene ID	FC 0NG - 8HG	adj p-value 0NG-8HG
ENSG00000177337	-1.3850063	0.00028462
ENSG00000177374	-1.3441842	0.00340446
ENSG00000177426	-1.3959674	8.24E-09
ENSG00000177464	1.20360593	0.00084386
ENSG00000177602	-1.2909356	0.00927513
ENSG00000177606	-1.2117208	1.00E-05
ENSG00000177663	1.20568281	0.00018495
ENSG00000177707	1.20123035	0.01172952
ENSG00000178015	1.27942829	0.00852514
ENSG00000178031	1.24161411	8.04E-06
ENSG00000178163	1.21558207	0.00069547
ENSG00000178343	1.21583567	0.03042209
ENSG00000178445	-1.4122877	1.11E-05
ENSG00000178752	-1.2538001	0.0210829
ENSG00000178882	1.71283212	1.40E-06
ENSG00000178951	1.20311351	4.88E-06
ENSG00000178999	-1.5808796	1.04E-06
ENSG00000179044	1.49464383	1.46E-05
ENSG00000179094	1.52495247	5.33E-09
ENSG00000179241	-1.2143547	4.54E-05
ENSG00000179314	-1.4403504	1.35E-06
ENSG00000179403	1.74473393	2.72E-06
ENSG00000179532	1.30117746	0.00457667
ENSG00000179598	1.26962811	0.00295068
ENSG00000179750	-1.3918418	0.00014718
ENSG00000179820	1.28998952	6.91E-10
ENSG00000179979	1.25313499	0.03420274
ENSG00000179981	-1.293342	1.15E-06
ENSG00000180448	1.37193422	0.00995161
ENSG00000180530	-1.3146395	6.43E-05
ENSG00000180573	-1.3475975	1.8728E-05
ENSG00000180596	-1.465836	2.79E-06

Gene ID	FC 0NG - 8HG	adj p-value 0NG-8HG
ENSG00000180730	-1.2268427	0.00817701
ENSG00000180776	1.21647291	0.01301941
ENSG00000180902	1.24333261	0.006556
ENSG00000180998	1.37582265	0.01129251
ENSG00000181444	1.37905675	0.00077421
ENSG00000181744	1.21529368	0.01197888
ENSG00000181826	-1.2565388	8.63E-11
ENSG00000182022	-1.399021	4.46E-12
ENSG00000182118	-1.386212	0.00828918
ENSG00000182134	-1.2038306	0.01206913
ENSG00000182158	-1.3068998	2.36E-18
ENSG00000182165	-1.2261334	0.0109006
ENSG00000182185	-1.2931374	0.01807742
ENSG00000182481	-1.4687696	1.73E-13
ENSG00000182489	1.34231041	0.00169347
ENSG00000182541	-1.2121459	0.00028475
ENSG00000182575	-1.3475889	0.00395442
ENSG00000182704	-1.2455497	0.00010275
ENSG00000182901	1.23376065	0.03139447
ENSG00000183044	1.35332832	0.00199907
ENSG00000183049	1.74271659	1.12E-07
ENSG00000183496	-1.5509936	1.56E-06
ENSG00000183578	1.21429679	0.00212739
ENSG00000183580	-1.2839243	1.01E-08
ENSG00000183598	-1.3078456	0.00068497
ENSG00000183688	1.25873253	0.00026595
ENSG00000183691	-1.3034039	0.04975165
ENSG00000183763	-1.3768354	0.00302931
ENSG00000183856	-1.654391	1.16E-09
ENSG00000183873	-1.2552748	0.00173413
ENSG00000183971	1.29736061	0.0227979
ENSG00000184009	1.22302661	6.84E-11

Gene ID	FC 0NG - 8HG	adj p-value 0NG-8HG
ENSG00000184083	1.22474339	0.00166104
ENSG00000184113	-1.9653484	2.88E-13
ENSG00000184220	-1.2329916	0.00026798
ENSG00000184226	1.3871752	0.00060351
ENSG00000184254	-1.299944	1.03E-07
ENSG00000184260	-1.4142197	9.56E-06
ENSG00000184270	-1.5637357	2.48E-05
ENSG00000184307	1.33713029	0.01765758
ENSG00000184357	-1.6621828	7.78E-08
ENSG00000184384	1.26677327	0.00010484
ENSG00000184445	-1.2362553	0.03490226
ENSG00000184588	-1.2076456	0.00045143
ENSG00000184640	-1.2098372	1.95E-06
ENSG00000184661	-1.6109294	3.94E-07
ENSG00000184678	-1.4191276	5.73E-06
ENSG00000184792	-1.522168	6.76E-08
ENSG00000184897	-1.3806471	7.84E-10
ENSG00000184916	1.42502725	1.55E-08
ENSG00000184988	1.20861	0.00664536
ENSG00000185130	-1.5352518	7.38E-06
ENSG00000185215	1.27391261	0.00086863
ENSG00000185246	1.23017319	0.04469994
ENSG00000185262	-1.4107783	4.30E-08
ENSG00000185338	-1.4952784	0.00031212
ENSG00000185352	1.69597201	1.92E-07
ENSG00000185361	-1.4056063	4.13E-07
ENSG00000185432	-1.3725371	8.79E-08
ENSG00000185551	1.23910264	1.44E-06
ENSG00000185630	-1.3427214	0.00153902
ENSG00000185670	-1.2326106	0.02759189
ENSG00000185753	1.23473241	2.30E-05
ENSG00000186130	1.31796403	0.02995928

Gene ID	FC 0NG - 8HG	adj p-value 0NG-8HG
ENSG00000186185	-1.5737779	2.79E-06
ENSG00000186193	-1.7033549	1.06E-11
ENSG00000186470	-1.2733772	2.31E-05
ENSG00000186479	-1.3175065	0.00091151
ENSG00000186480	-1.5130923	1.20E-05
ENSG00000186567	1.40564847	1.09E-05
ENSG00000186603	1.23642854	0.02160819
ENSG00000186638	-1.5288031	6.00E-05
ENSG00000186642	-1.3569603	0.00015199
ENSG00000186871	-1.3047186	0.00540712
ENSG00000187266	-1.369174	9.62E-06
ENSG00000187513	-1.5474117	0.017646
ENSG00000187741	-1.219984	0.03527406
ENSG00000187764	-1.3612115	0.00028224
ENSG00000187824	1.21729705	0.01056009
ENSG00000187837	-1.5988474	1.72E-08
ENSG00000187840	-1.2960034	6.64E-06
ENSG00000187951	-1.400891	0.00411056
ENSG00000188211	-1.2100175	6.24E-05
ENSG00000188277	-1.8149532	0.00123215
ENSG00000188428	1.29423044	0.00569169
ENSG00000188486	-1.4014511	1.22E-06
ENSG00000188596	1.34808287	0.00187186
ENSG00000189056	1.28859121	0.00493456
ENSG00000189057	1.36886265	0.01601081
ENSG00000189221	1.36121099	3.51E-05
ENSG00000196159	1.26287442	0.01013763
ENSG00000196169	-1.9451735	6.70E-10
ENSG00000196187	-1.2063499	0.01189667
ENSG00000196209	-1.4121488	0.00041734
ENSG00000196378	-1.2571823	0.02420818
ENSG00000196421	-1.3270762	0.00073902

Gene ID	FC 0NG - 8HG	adj p-value 0NG-8HG
ENSG00000196468	-1.4425751	0.00028004
ENSG00000196730	-1.2419009	8.30E-08
ENSG00000196747	-1.5466057	4.91E-06
ENSG00000196787	-1.4151032	0.00017672
ENSG00000196814	-1.2126556	0.01381396
ENSG00000196839	-1.3190862	0.00091402
ENSG00000196866	-1.501237	5.52E-07
ENSG00000196890	-1.4226421	9.9627E-05
ENSG00000197061	-1.5364506	6.67E-07
ENSG00000197153	-1.4266064	0.0010666
ENSG00000197238	-1.3954466	6.30E-06
ENSG00000197261	-1.4475653	0.00028475
ENSG00000197343	1.2342531	0.03243904
ENSG00000197409	-1.6351083	2.63E-07
ENSG00000197461	1.32925209	0.00016265
ENSG00000197632	1.3138179	0.02862107
ENSG00000197837	-1.3035912	7.20E-05
ENSG00000197852	-1.2431734	0.00019564
ENSG00000197860	1.2153555	0.00670304
ENSG00000197863	1.26823509	0.00276124
ENSG00000197903	-1.4406408	4.50E-05
ENSG00000198108	1.46089474	0.00056999
ENSG00000198431	1.28337499	3.58E-05
ENSG00000198483	1.29940966	0.04228603
ENSG00000198695	1.28793868	0.02483675
ENSG00000198720	-1.2195267	0.00017676
ENSG00000198804	1.22415349	0.03124278
ENSG00000198840	1.29666458	0.02033916
ENSG00000198846	1.88381203	8.57E-09
ENSG00000198873	-1.4360267	1.29E-10
ENSG00000198901	-1.57769	3.87E-10
ENSG00000198909	-1.2477382	5.77E-09

Gene ID	FC 0NG - 8HG	adj p-value 0NG-8HG
ENSG00000198929	-1.3241398	0.00687641
ENSG00000198938	1.25102714	0.01148178
ENSG00000198959	-1.3245944	1.22E-08
ENSG00000203805	1.4210245	0.00026829
ENSG00000203814	-1.6251648	1.41E-09
ENSG00000203883	-1.6046051	2.56E-05
ENSG00000203896	1.29446272	0.02578887
ENSG00000204217	1.379051	6.46E-06
ENSG00000204442	2.11234765	4.50E-13
ENSG00000204767	1.21594591	2.01E-05
ENSG00000204776	1.43738465	4.30E-05
ENSG00000204977	1.20991078	0.04342599
ENSG00000205177	1.25212889	0.03932532
ENSG00000205476	-1.3201681	1.50E-06
ENSG00000206417	-1.3260359	0.00207317
ENSG00000206538	1.70740331	5.09E-09
ENSG00000210196	1.28875557	0.0461671
ENSG00000211584	-1.2100757	0.00690912
ENSG00000213139	1.24230542	0.02883135
ENSG00000213186	-1.2574907	0.01894974
ENSG00000213190	1.35764565	6.27E-06
ENSG00000213347	-1.6530337	2.22E-08
ENSG00000213390	-1.2380282	0.00016943
ENSG00000213853	-1.411241	6.85E-07
ENSG00000214357	-1.9218573	1.23E-06
ENSG00000214655	-1.2425285	9.3945E-05
ENSG00000215790	1.21034529	0.03837988
ENSG00000216775	-1.6805906	4.32E-08
ENSG00000221829	-1.309432	0.00011406
ENSG00000221866	1.31972079	0.02088781
ENSG00000221926	-1.2491069	1.54E-05
ENSG00000221963	-1.3964256	4.57E-05

Gene ID	FC 0NG - 8HG	adj p-value 0NG-8HG
ENSG00000222047	-1.2880225	0.01810846
ENSG00000224080	1.24988489	0.02714388
ENSG00000224635	1.28368852	0.00979012
ENSG00000224914	1.22923136	0.01615584
ENSG00000224982	-1.2864657	0.02297748
ENSG00000225630	1.29733931	0.01127665
ENSG00000225830	1.63332841	2.82E-12
ENSG00000226328	1.26068996	0.01765958
ENSG00000226702	1.29162311	0.0028526
ENSG00000226808	1.2738446	0.00415413
ENSG00000226823	1.27336213	0.00920927
ENSG00000227082	1.36801337	0.01154603
ENSG00000227517	1.26299842	0.04606926
ENSG00000228495	1.40004987	2.44E-08
ENSG00000228794	1.30658151	0.00033163
ENSG00000230266	-1.2341814	0.04289717
ENSG00000230438	1.2626163	0.0398362
ENSG00000230479	-1.2235484	5.07E-05
ENSG00000230753	-1.2580845	0.003839
ENSG00000232034	-1.2233195	0.03086044
ENSG00000232434	1.23864428	0.03656779
ENSG00000232527	1.20496836	0.01577345
ENSG00000232949	1.20428667	0.04387446
ENSG00000233117	1.28995131	0.03201987
ENSG00000233251	1.43247308	3.62E-06
ENSG00000233369	-1.2334029	0.00013418
ENSG00000233370	1.23942591	0.00354058
ENSG00000233818	-1.2340582	0.00086388
ENSG00000233822	-1.5980332	5.60E-07
ENSG00000234608	-1.2146193	0.02270725
ENSG00000235217	1.34460525	0.00190609
ENSG00000235770	1.37199236	2.44E-07

Gene ID	FC 0NG - 8HG	adj p-value 0NG-8HG
ENSG00000235823	-1.4384497	0.00012106
ENSG00000237181	1.24866825	0.01003494
ENSG00000237870	-1.2740527	0.02164764
ENSG00000239306	1.21124229	2.10E-06
ENSG00000239887	-1.2785864	0.00268142
ENSG00000240694	-1.8293026	1.77E-16
ENSG00000240891	-1.2502693	0.02545246
ENSG00000241749	1.2892282	0.01316322
ENSG00000243927	-1.2096793	0.00199894
ENSG00000244300	1.32318501	6.08E-05
ENSG00000244405	-1.2058809	4.29E-10
ENSG00000247626	1.221057	0.00275026
ENSG00000249242	1.33500601	0.00059336
ENSG00000249279	-1.2487932	0.02989733
ENSG00000249471	1.22505177	0.03770072
ENSG00000249992	-1.2716197	0.00049465
ENSG00000250303	1.44370931	6.94E-06
ENSG00000250510	-1.2488896	0.00976123
ENSG00000253368	1.89340027	1.06E-16
ENSG00000253661	1.48098113	1.96E-05
ENSG00000255248	1.42362516	0.00030245
ENSG00000255690	-1.3018493	0.01028955
ENSG00000257167	-1.4326836	0.00105802
ENSG00000258376	1.23050299	0.0160787
ENSG00000258441	1.53168182	1.39E-06
ENSG00000259518	-1.2969082	0.04912411
ENSG00000260766	1.2371023	0.03608557
ENSG00000260920	1.22733297	0.02999188
ENSG00000261253	-1.2801119	0.00624504
ENSG00000261373	1.28939703	8.37E-05
ENSG00000261468	1.52732033	0.00037397
ENSG00000261534	1.31143825	0.03546898

Gene ID	FC 0NG - 8HG	adj p-value 0NG-8HG
ENSG00000262001	-1.4613547	2.24E-06
ENSG00000262580	1.29622529	0.00599998
ENSG00000263155	-1.278538	0.02550766
ENSG00000263528	1.24510249	0.0001513
ENSG00000265972	1.29662401	0.00064041
ENSG00000267317	1.23695613	0.04831108
ENSG00000267583	-1.4657619	2.26E-09
ENSG00000267710	1.20697839	0.00699353
ENSG00000270276	-1.2307967	0.04185428
ENSG00000270607	-1.3139476	0.0010107
ENSG00000271425	-1.2590564	0.02377837
ENSG00000272324	-1.2361508	0.0330454
ENSG00000272398	1.20548242	0.0264699
ENSG00000272831	-1.2793337	0.00595501
ENSG00000272914	1.30216025	0.00382808
ENSG00000273466	1.24716423	0.00820978
ENSG00000273542	-1.5498275	0.00061358
ENSG00000273703	-1.4753868	9.02E-05
ENSG00000273802	-1.4856963	2.86E-06
ENSG00000273850	-1.2353529	0.03760358
ENSG00000273983	-1.4577039	6.03E-05
ENSG00000274016	1.39180594	0.00086011
ENSG00000274267	-1.593974	3.92E-06
ENSG00000274290	-1.5365424	3.77E-07
ENSG00000274618	-1.3826506	0.00212061
ENSG00000274641	-1.558089	9.40E-06
ENSG00000274897	1.20467892	0.04756385
ENSG00000274997	-1.4666645	0.00016293
ENSG00000275126	-1.5432548	2.17E-06
ENSG00000275221	-1.3656872	0.00373688
ENSG00000275322	1.25316067	0.02734653
ENSG00000275342	-1.2807622	0.00100686

Gene ID	FC 0NG - 8HG	adj p-value 0NG-8HG
ENSG00000275379	-1.580686	3.18E-06
ENSG00000275713	-1.7739858	2.69E-07
ENSG00000275714	-1.3697638	1.3817E-05
ENSG00000276180	-1.2738346	0.03942266
ENSG00000276368	-1.5735595	4.37E-06
ENSG00000276410	-1.5744144	2.06E-06
ENSG00000276903	-1.5531725	2.19E-05
ENSG00000276966	-1.3292702	2.01E-06
ENSG00000277075	-1.5611781	4.74E-07
ENSG00000277157	-1.6398451	4.58E-07
ENSG00000277224	-1.6558376	1.90E-07
ENSG00000277775	-1.9022167	2.80E-09
ENSG00000278272	-1.5487422	5.17E-06
ENSG00000278384	1.24156863	0.03617469
ENSG00000278463	-1.4402691	6.96E-05
ENSG00000278588	-1.5016109	1.36E-05
ENSG00000278637	-1.3552028	9.76E-05
ENSG00000278705	-1.394313	1.59E-05
ENSG00000278828	-1.5340144	2.66E-06
ENSG00000279010	1.2058892	0.04228279
ENSG00000279088	-1.2996296	0.04637529
ENSG00000279095	1.29636769	2.61E-06
ENSG00000279117	-1.3345589	1.78E-07
ENSG00000279207	1.30591693	0.00091775
ENSG00000280696	-1.3295458	0.01186987
ENSG00000280826	-1.353277	0.04176046

Supplementary Table 3e. Differentially expressed genes between 0hr normal glucose and 24hr high glucose samples

Ensembl gene IDs, Fold change (baseline-24HG) and FDR adjusted p-values of DEGs

Gene ID	FC 0NG - 24HG	adj p-value 0NG-24HG
ENSG00000000003	1.27758467	0.00010018
ENSG00000000460	-1.279817	0.01941291
ENSG00000000971	1.22246836	0.01368394
ENSG00000001167	1.20577027	0.00021222
ENSG00000001630	-1.3527586	0.00015339
ENSG00000002079	-1.362029	0.00768292
ENSG00000003096	1.27764777	0.00475129
ENSG00000003989	1.31758882	0.02128138
ENSG00000004777	-1.3890294	0.00431079
ENSG00000004799	1.45668732	7.15E-05
ENSG00000005187	1.24354037	0.01021081
ENSG00000005448	-1.2195993	0.0231079
ENSG00000006638	1.26499207	0.00840918
ENSG00000007384	-1.2917834	0.00162896
ENSG00000009413	1.20041808	0.04965974
ENSG00000010292	-1.3521489	4.92E-10
ENSG00000010318	-1.2460943	0.03410449
ENSG00000010319	1.40853312	0.00309327
ENSG00000011405	1.20653777	0.01399343
ENSG00000011426	-1.3484849	3.33E-05
ENSG00000013016	1.23197338	0.04231028
ENSG00000013293	1.26207448	0.02303216
ENSG00000013588	1.58072801	7.28E-09
ENSG00000013810	-1.4110839	6.18E-05
ENSG00000020577	1.21728369	4.83E-08
ENSG00000021645	1.46179121	2.57E-05
ENSG00000022267	1.22120793	4.25E-07
ENSG00000025156	1.24306148	0.01536807
ENSG00000026559	1.24782943	0.02504787
ENSG00000029993	-1.2832768	6.70E-07
ENSG00000033867	-1.2413852	0.00794035
ENSG00000035499	-1.4768739	1.81E-06

Gene ID	FC 0NG - 24HG	adj p-value 0NG-24HG
ENSG00000037280	1.40433596	0.00032755
ENSG00000038295	1.42137746	0.00017931
ENSG00000046889	1.23621906	0.00051589
ENSG00000047346	1.36799223	0.0024709
ENSG00000049192	1.22872727	0.00027155
ENSG00000050344	1.20833776	0.00017676
ENSG00000050438	-1.2119352	0.00109687
ENSG00000051341	-1.2196048	0.04295807
ENSG00000053254	1.20960516	9.05E-05
ENSG00000054654	-1.2572545	0.04811202
ENSG00000057019	1.44775556	2.79E-07
ENSG00000057704	-1.2092929	0.00091215
ENSG00000058085	-1.3521243	0.00057532
ENSG00000058404	-1.4466845	6.63E-05
ENSG00000063660	-1.2336459	1.5844E-05
ENSG00000064042	1.71458511	9.17E-08
ENSG00000064199	-1.2079588	0.02768284
ENSG00000064309	1.24836688	0.01574809
ENSG00000065308	-1.2264559	8.05E-06
ENSG00000066279	-1.449408	0.01407822
ENSG00000066923	-1.2475816	0.00197949
ENSG00000067064	-1.3655615	0.00324368
ENSG00000067191	-1.2460025	0.0305932
ENSG00000068489	-1.2797914	3.23E-06
ENSG00000069122	1.26424408	0.024742
ENSG00000069509	1.22216264	0.00284911
ENSG00000069702	1.32224866	0.00100823
ENSG00000070404	1.28933844	0.00048831
ENSG00000070614	-1.2895597	4.62E-09
ENSG00000070669	-1.5740631	5.63E-09
ENSG00000070961	1.2192594	0.02305257
ENSG00000071073	-1.2136876	0.00136199

Gene ID	FC 0NG - 24HG	adj p-value 0NG-24HG
ENSG00000071539	-1.3301871	4.09E-06
ENSG00000072071	1.53520817	7.09E-09
ENSG00000072201	-1.2905368	0.0033041
ENSG00000072571	-1.3840058	0.00264089
ENSG00000073464	1.28583609	3.59E-05
ENSG00000073849	1.27545501	4.83E-07
ENSG00000074410	1.2380842	0.03497736
ENSG00000074527	1.47592419	1.35E-05
ENSG00000074855	1.29390261	0.0024584
ENSG00000075218	-1.4623994	1.05E-05
ENSG00000075702	-1.3180464	0.00273549
ENSG00000076043	1.21119241	4.62E-05
ENSG00000076248	1.25005862	2.51E-06
ENSG00000076382	-1.3335027	1.34E-05
ENSG00000077092	2.12343368	1.35E-12
ENSG00000078401	1.21948638	0.00147245
ENSG00000078596	1.2191475	3.15E-05
ENSG00000079102	1.28840803	0.01018006
ENSG00000079385	1.43676067	0.00011334
ENSG00000079435	-1.2468091	0.02526048
ENSG00000079459	-1.4289853	2.01E-11
ENSG00000079616	-1.4511838	2.15E-07
ENSG00000080986	-1.4321093	4.90E-05
ENSG00000081320	-1.2644551	0.01055785
ENSG00000081760	-1.2080091	3.39E-06
ENSG00000081913	1.26943611	0.02243709
ENSG00000082126	-1.4916054	3.17E-09
ENSG00000085871	-1.2362104	6.19E-06
ENSG00000087253	1.29294519	2.16E-05
ENSG00000087303	-1.3655905	2.33E-07
ENSG00000087494	1.32847272	0.02691506
ENSG00000087586	-1.3589041	5.58E-06

Gene ID	FC 0NG - 24HG	adj p-value 0NG-24HG
ENSG00000088038	-1.2065664	0.0486885
ENSG00000088280	1.34854071	1.80E-05
ENSG00000088305	-1.2596315	0.02355445
ENSG00000088325	-1.4255993	6.07E-07
ENSG00000088899	1.24946089	0.03912359
ENSG00000089685	-1.3030503	1.97E-05
ENSG00000090339	1.62582341	0.00028517
ENSG00000090530	1.403438	0.00072639
ENSG00000090776	-1.2515418	2.88E-05
ENSG00000090889	-1.5420748	7.21E-08
ENSG00000091129	1.33821913	7.86E-07
ENSG00000091137	-1.344924	0.00121173
ENSG00000091409	-1.3034984	4.64E-07
ENSG00000091622	1.39930606	0.01879186
ENSG00000091879	1.26537553	2.87E-05
ENSG00000092621	-1.6228228	1.35E-09
ENSG00000092964	-1.2345536	6.67E-13
ENSG00000092969	1.60801076	0.00018155
ENSG00000094914	-1.2177273	4.5249E-05
ENSG00000094916	-1.2564478	1.03E-05
ENSG00000095370	-1.2187982	0.00085455
ENSG00000095397	1.24749726	0.00706543
ENSG00000095539	1.384217	7.12E-05
ENSG00000096433	-1.3651967	3.93E-11
ENSG00000099194	-1.2644111	4.09E-08
ENSG00000099282	-1.2341687	0.00941502
ENSG00000099864	1.24136454	0.02577669
ENSG00000099937	1.68793487	5.66E-06
ENSG00000099998	-1.3393106	0.00010683
ENSG00000100034	-1.289078	7.06E-09
ENSG00000100036	-1.2446775	0.0009481
ENSG00000100075	-1.2431948	4.65E-06

Gene ID	FC 0NG - 24HG	adj p-value 0NG-24HG
ENSG00000100092	1.28716134	0.02459512
ENSG00000100100	1.68897693	4.19E-07
ENSG00000100321	1.42254731	0.00225395
ENSG00000100368	1.28286295	0.0002035
ENSG00000100490	-1.2195803	0.03338213
ENSG00000100526	-1.3308559	0.00041258
ENSG00000100577	-1.2800479	0.00016731
ENSG00000100629	-1.4906858	3.02E-05
ENSG00000100784	1.28309133	0.0113245
ENSG00000100906	1.22855524	0.00335567
ENSG00000100918	1.29123613	0.00042216
ENSG00000101057	-1.218633	0.01876564
ENSG00000101096	-1.2196229	0.0036903
ENSG00000101187	1.27783141	0.01601998
ENSG00000101224	-1.3322232	1.40E-06
ENSG00000101255	-1.2117574	0.00117754
ENSG00000101311	1.28281247	0.00531513
ENSG00000101333	1.26057948	0.03605423
ENSG00000101335	1.26435484	0.00868106
ENSG00000101447	-1.522137	2.84E-07
ENSG00000101665	1.49944776	0.00889079
ENSG00000101945	-1.2707259	0.00557534
ENSG00000102007	-1.2007338	1.93E-06
ENSG00000102265	-1.230325	6.74E-05
ENSG00000102287	1.36720232	0.01758888
ENSG00000102384	-1.299655	0.00280556
ENSG00000102452	1.23985957	0.01790159
ENSG00000102802	1.8480947	4.02E-09
ENSG00000102908	1.22010837	0.00982052
ENSG00000103018	-1.2769738	1.81E-10
ENSG00000103034	1.74268289	2.55E-11
ENSG00000103044	1.32236096	0.00987291

Gene ID	FC 0NG - 24HG	adj p-value 0NG-24HG
ENSG00000103152	-1.2064961	5.35E-05
ENSG00000103642	1.22189768	0.01194438
ENSG00000104147	-1.3711234	0.00121926
ENSG00000104290	1.23462083	0.04255
ENSG00000104361	1.37213137	5.81E-05
ENSG00000104369	-1.2648864	0.00016739
ENSG00000104497	1.22823591	0.01111958
ENSG00000104549	-1.4055873	1.64E-08
ENSG00000104884	1.23430957	3.68E-06
ENSG00000104889	-1.3775574	0.00014712
ENSG00000104951	1.34760564	0.00455604
ENSG00000105011	-1.2686556	0.00230724
ENSG00000105173	1.33041946	0.00060818
ENSG00000105270	1.48589666	0.0001362
ENSG00000105325	-1.2168116	3.48E-05
ENSG00000105497	1.20300527	0.00235178
ENSG00000105499	1.46796784	0.00015626
ENSG00000105851	-1.4273713	5.82E-08
ENSG00000105855	2.1109096	1.80E-12
ENSG00000105967	1.42727907	0.0003932
ENSG00000106004	1.31640072	0.00112868
ENSG00000106236	-1.2863991	0.0388451
ENSG00000106392	1.22501985	0.03745365
ENSG00000106723	1.26870239	0.0044489
ENSG00000106733	1.24147964	0.01921456
ENSG00000106772	1.22096771	0.00108065
ENSG00000107719	-1.6013395	9.21E-08
ENSG00000107807	1.31437002	0.01529462
ENSG00000107984	1.29943252	0.00156227
ENSG00000108106	-1.283733	5.84E-05
ENSG00000108306	1.2647255	0.00267386
ENSG00000108448	1.23713434	0.00713173

Gene ID	FC 0NG - 24HG	adj p-value 0NG-24HG
ENSG00000108551	-1.2151568	0.02078051
ENSG00000108861	1.24581052	4.69E-12
ENSG00000108984	1.60534664	7.95E-06
ENSG00000109084	-1.32262	5.46E-07
ENSG00000109501	-1.3431088	1.65E-05
ENSG00000109685	-1.218284	7.51E-07
ENSG00000109738	1.39054277	0.00064142
ENSG00000109805	-1.3554278	0.00275512
ENSG00000109854	1.22291009	2.77E-08
ENSG00000110042	1.26417881	0.00022959
ENSG00000110330	1.28396654	0.00310246
ENSG00000110675	1.32714491	0.00423775
ENSG00000110799	1.24921705	9.03E-05
ENSG00000110900	1.36756062	0.00018826
ENSG00000110921	-1.3835576	7.22E-07
ENSG00000111057	-1.2427165	0.00011592
ENSG00000111206	-1.321019	9.48E-08
ENSG00000111247	-1.3156722	0.00058421
ENSG00000111339	1.58704427	1.31E-08
ENSG00000111341	-1.2618855	0.00013763
ENSG00000111665	-1.4079338	0.00074207
ENSG00000111799	1.41089213	0.00149945
ENSG00000111912	1.22687386	8.25E-05
ENSG00000111962	1.22631256	0.03325786
ENSG00000112137	-1.2442708	0.00075971
ENSG00000112297	-1.4883492	1.20E-05
ENSG00000112697	1.25485689	0.02277075
ENSG00000112742	-1.4566889	0.00531582
ENSG00000112773	1.42501886	0.04509158
ENSG00000112972	-1.72933	6.73E-07
ENSG00000112984	-1.4620609	3.78E-08
ENSG00000113161	-1.4745379	3.64E-06

Gene ID	FC 0NG - 24HG	adj p-value 0NG-24HG
ENSG00000113368	-1.2292469	3.05E-05
ENSG00000113555	-1.9048144	2.66E-08
ENSG00000114126	1.24496301	0.00033905
ENSG00000114698	1.64739536	2.20E-09
ENSG00000114796	1.2027346	0.04491989
ENSG00000114812	-1.9702892	6.92E-07
ENSG00000115042	-1.2038854	0.00376007
ENSG00000115163	-1.3608398	0.00039648
ENSG00000115290	1.26454167	0.00356722
ENSG00000115525	-1.3334748	0.00790115
ENSG00000115594	1.21335113	0.00257712
ENSG00000115602	2.3001156	7.87E-08
ENSG00000115604	1.27912123	0.00980425
ENSG00000115738	1.49545316	0.01294177
ENSG00000115902	-1.2364737	0.00017178
ENSG00000115963	1.3271511	0.00025113
ENSG00000116133	-1.2929943	9.92E-09
ENSG00000116771	1.3014852	0.01287461
ENSG00000116830	-1.2033956	0.00343846
ENSG00000116991	-1.2946957	8.12E-05
ENSG00000117152	1.52065917	1.66E-08
ENSG00000117298	-1.2435506	4.62E-07
ENSG00000117318	1.43679178	2.01E-07
ENSG00000117399	-1.3675357	3.96E-05
ENSG00000117650	-1.603554	3.72E-08
ENSG00000117724	-1.635663	3.57E-09
ENSG00000118193	-1.4687585	8.37E-06
ENSG00000118402	1.40185496	0.00157079
ENSG00000118407	1.39820853	0.00066171
ENSG00000118515	1.32340279	7.76E-11
ENSG00000118564	1.21329871	0.01197962
ENSG00000118596	1.29635634	0.01880647

Gene ID	FC 0NG - 24HG	adj p-value 0NG-24HG
ENSG00000119326	1.22462156	0.00150091
ENSG00000119333	-1.2273583	1.47E-05
ENSG00000119397	-1.3568571	8.46E-07
ENSG00000119403	-1.3445916	4.05E-08
ENSG00000119630	-1.6831294	7.36E-11
ENSG00000119714	1.24531822	0.02004941
ENSG00000119718	-1.4446175	4.36E-12
ENSG00000119801	1.26800603	0.00379354
ENSG00000119900	-1.3093461	0.00105841
ENSG00000119938	1.52762818	1.83E-05
ENSG00000119950	1.30840714	0.01204674
ENSG00000120093	1.24451007	0.00220711
ENSG00000120256	-1.2248335	9.53E-05
ENSG00000120278	-1.2287991	0.0001193
ENSG00000120437	-1.5549196	2.14E-10
ENSG00000120457	-1.2893644	0.00075999
ENSG00000120693	1.31965095	0.00493649
ENSG00000121152	-1.3868242	0.00018166
ENSG00000121621	-1.3043462	0.04298931
ENSG00000121741	1.27143316	0.0099549
ENSG00000121797	-1.3139647	0.00029289
ENSG00000121957	-1.4650694	3.47E-07
ENSG00000121966	-1.2102245	0.01754432
ENSG00000122483	-1.3479708	0.00088319
ENSG00000122641	1.64699599	6.87E-08
ENSG00000122952	-1.2661181	3.03E-05
ENSG00000122966	-1.6087526	5.01E-10
ENSG00000123080	-1.6346811	6.90E-10
ENSG00000123095	1.31635368	0.0267784
ENSG00000123384	-1.3595557	0.00048803
ENSG00000123473	-1.2538537	0.00187513
ENSG00000123485	-1.7525593	5.29E-07

Gene ID	FC 0NG - 24HG	adj p-value 0NG-24HG
ENSG00000123700	1.58670437	0.00205552
ENSG00000123975	-1.224328	0.00998472
ENSG00000124216	1.23046475	0.03123643
ENSG00000124406	1.24989324	0.029517
ENSG00000124508	1.22788228	0.0074841
ENSG00000124610	1.22174621	0.02569213
ENSG00000124875	1.87027465	1.98E-07
ENSG00000125266	1.48366655	2.15E-06
ENSG00000125378	1.97110164	5.74E-11
ENSG00000125848	1.59552603	0.00054298
ENSG00000125864	1.28142507	0.0061639
ENSG00000125945	1.22200802	0.00151822
ENSG00000125968	1.66012058	4.58E-05
ENSG00000126016	1.25225608	0.04440983
ENSG00000126453	-1.2324003	0.01786712
ENSG00000126562	1.38301813	1.47E-05
ENSG00000126785	1.26631525	2.12E-09
ENSG00000126950	1.24166842	0.0445014
ENSG00000127418	1.26633152	0.00023089
ENSG00000127533	-2.1894596	6.22E-12
ENSG00000127955	1.21770698	0.03079285
ENSG00000128567	-1.3589948	5.93E-09
ENSG00000128590	1.24780063	0.01823882
ENSG00000128602	1.23810418	0.03839981
ENSG00000128606	1.42504752	1.21E-07
ENSG00000128641	-1.3318092	4.77E-05
ENSG00000128833	-1.3980728	0.00014159
ENSG00000128917	-1.6813829	4.10E-06
ENSG00000128944	-1.2578638	0.00021653
ENSG00000129116	1.27836425	0.00160641
ENSG00000129195	-1.3658329	9.66E-05
ENSG00000129465	1.25921999	0.03582999

Gene ID	FC 0NG - 24HG	adj p-value 0NG-24HG
ENSG00000129646	-1.2546675	0.03139878
ENSG00000129810	-1.4060596	9.55E-05
ENSG00000129946	1.26169708	0.0316333
ENSG00000130024	-1.2253332	0.00286245
ENSG00000130052	-1.2549303	0.00028959
ENSG00000130164	-1.3574536	5.25E-06
ENSG00000130513	1.31228698	5.63E-07
ENSG00000130520	-1.2127568	2.82E-06
ENSG00000130755	-1.2136157	0.00146457
ENSG00000130956	-1.2013119	0.00350468
ENSG00000130988	1.27877742	0.01530098
ENSG00000131016	-1.3678961	1.22E-09
ENSG00000131069	-1.2581875	0.00412827
ENSG00000131370	-1.4317259	3.93E-11
ENSG00000131389	1.31644644	1.89E-11
ENSG00000131459	1.35248495	0.00033229
ENSG00000131652	-1.2386234	0.00312433
ENSG00000131724	1.31910464	2.16E-05
ENSG00000131747	-1.5195243	3.86E-06
ENSG00000131773	-1.3407933	0.00109409
ENSG00000132182	-1.3706069	0.00118116
ENSG00000132196	-1.3246566	1.24E-05
ENSG00000132470	-1.3090752	0.0031159
ENSG00000132530	1.35533308	0.02810638
ENSG00000132563	-1.3396536	0.00351431
ENSG00000132570	-1.2300456	0.03260664
ENSG00000132623	1.23209883	0.03744902
ENSG00000133110	1.34261765	2.64E-06
ENSG00000133863	1.57841368	0.00026815
ENSG00000133935	-1.2628842	1.18E-05
ENSG00000134057	-1.3477435	3.1375E-05
ENSG00000134107	-1.3612886	4.97E-07

Gene ID	FC 0NG - 24HG	adj p-value 0NG-24HG
ENSG00000134109	-1.2024574	5.13E-09
ENSG00000134222	-1.642795	6.33E-07
ENSG00000134294	1.48037672	0.00091876
ENSG00000134324	-1.3828868	4.76E-11
ENSG00000134352	1.33120408	0.00049016
ENSG00000134504	1.22778021	0.00077633
ENSG00000134508	1.34123767	0.00017388
ENSG00000134690	-1.5793171	3.60E-07
ENSG00000134775	1.4454484	0.0309928
ENSG00000134824	-1.2470816	1.15E-05
ENSG00000135069	-1.30395	1.31E-08
ENSG00000135119	-1.310013	0.04659959
ENSG00000135451	-1.4780148	0.00021524
ENSG00000135476	-1.4677683	0.00033164
ENSG00000135643	1.66730986	2.26E-07
ENSG00000135842	-1.2091552	0.00186403
ENSG00000135914	1.35126328	0.02107005
ENSG00000135929	1.25586875	0.00486322
ENSG00000135966	-1.2239453	0.00019706
ENSG00000136010	-1.3615766	3.94E-05
ENSG00000136108	-1.3391002	0.0015598
ENSG00000136122	-1.2421877	0.01286856
ENSG00000136153	-1.558251	2.72E-12
ENSG00000136161	1.20353474	0.03042267
ENSG00000136205	-1.6856969	2.56E-13
ENSG00000136237	-1.2581914	0.00109746
ENSG00000136367	1.23801328	0.04340901
ENSG00000136383	1.42772833	1.37E-07
ENSG00000136490	1.33493691	3.17E-06
ENSG00000136603	1.2153902	0.02974005
ENSG00000136630	-1.3408494	0.03778554
ENSG00000136811	-1.272625	1.74E-06

Gene ID	FC 0NG - 24HG	adj p-value 0NG-24HG
ENSG00000136824	-1.2060639	0.00640352
ENSG00000136861	-1.2539454	1.43E-05
ENSG00000136869	1.4116279	3.72E-08
ENSG00000136928	-1.266315	6.96E-05
ENSG00000137033	1.22237995	0.00012813
ENSG00000137133	-1.2265172	0.00328657
ENSG00000137135	-1.3548357	0.00058552
ENSG00000137502	1.21635722	0.04550943
ENSG00000137507	1.39392338	6.06E-06
ENSG00000137522	1.32580916	1.79E-06
ENSG00000137573	1.2427491	0.01167436
ENSG00000137727	1.45355277	1.2761E-05
ENSG00000137752	1.24779697	0.02258244
ENSG00000137804	-1.6366604	2.57E-09
ENSG00000137807	-1.5422107	2.62E-08
ENSG00000137812	-1.5130006	9.94E-05
ENSG00000137825	-1.2808358	0.00386276
ENSG00000137834	1.73076913	0.00061774
ENSG00000137872	1.46501399	0.00011753
ENSG00000137878	-1.291819	0.01818419
ENSG00000137965	1.2550621	0.04141259
ENSG00000137968	1.36373509	0.01728497
ENSG00000138160	-1.4062177	0.00055695
ENSG00000138162	-1.3188432	0.00033115
ENSG00000138180	-1.4274774	1.63E-05
ENSG00000138185	1.36479023	0.00278647
ENSG00000138347	-1.3697883	0.00813042
ENSG00000138356	1.34183224	0.00025113
ENSG00000138434	-1.2065459	1.11E-05
ENSG00000138449	1.97066084	6.80E-14
ENSG00000138640	1.22647078	0.03263881
ENSG00000138675	1.319433	0.00605856

Gene ID	FC 0NG - 24HG	adj p-value 0NG-24HG
ENSG00000138685	1.28335665	0.00216947
ENSG00000138722	1.20003	0.01500803
ENSG00000138771	-1.3155793	0.00099755
ENSG00000139112	1.29945318	4.10E-06
ENSG00000139187	1.47403096	0.00014588
ENSG00000139211	1.32601447	0.0025745
ENSG00000139278	1.3266605	4.37E-06
ENSG00000139354	-1.3354219	0.00030467
ENSG00000139410	-1.2503758	0.00576946
ENSG00000139428	-1.3979872	1.02E-10
ENSG00000139517	1.21316374	0.00126545
ENSG00000139734	-1.2508865	0.00255097
ENSG00000139793	1.21812468	0.00162164
ENSG00000139880	1.26782022	0.00072415
ENSG00000140092	-1.3713219	1.35E-06
ENSG00000140105	1.28723719	9.05E-05
ENSG00000140365	-1.2374938	4.93E-06
ENSG00000140416	1.25971682	1.31E-08
ENSG00000140451	-1.6023861	3.6004E-05
ENSG00000140465	1.28050701	0.03153216
ENSG00000140525	-1.2458386	0.00286749
ENSG00000140534	-1.4510012	5.25E-05
ENSG00000140543	-1.2046452	0.00505017
ENSG00000140678	-1.317883	0.00107113
ENSG00000140848	-1.2114073	5.55E-06
ENSG00000140873	1.27550698	0.00337927
ENSG00000140939	1.21565785	0.03849946
ENSG00000141338	1.27461086	0.01751442
ENSG00000141519	1.25364637	0.00880715
ENSG00000141526	1.22263605	2.53E-05
ENSG00000141542	1.21689893	0.01516836
ENSG00000141934	1.29530264	0.00191655

Gene ID	FC 0NG - 24HG	adj p-value 0NG-24HG
ENSG00000142082	1.20638971	0.04318787
ENSG00000142494	-1.2025395	0.04544241
ENSG00000142945	-1.5945491	4.96E-07
ENSG00000143153	1.26655047	5.51E-05
ENSG00000143156	1.22025678	0.02400904
ENSG00000143228	-1.33043	0.00392416
ENSG00000143344	-1.355451	5.11E-08
ENSG00000143387	1.32673018	0.00094049
ENSG00000143434	1.47433437	1.63E-07
ENSG00000143469	1.43928706	0.00221632
ENSG00000143772	-1.427533	1.04E-07
ENSG00000143816	-1.4425284	0.00737939
ENSG00000143819	1.20895319	0.00857303
ENSG00000143878	1.31618559	0.00137306
ENSG00000144063	-1.2260273	0.00079546
ENSG00000144199	-1.2172482	0.03662697
ENSG00000144554	-1.3292489	0.00013345
ENSG00000144645	-1.3046383	6.63E-09
ENSG00000144677	-1.223927	7.00E-05
ENSG00000144749	-1.3366826	7.97E-06
ENSG00000144843	1.27126529	0.00991113
ENSG00000145147	1.32364603	2.27E-08
ENSG00000145386	-1.488044	8.90E-08
ENSG00000145882	-1.2691931	4.41E-06
ENSG00000145911	-1.4877768	5.34E-06
ENSG00000146278	1.20425063	0.01383075
ENSG00000146409	1.21226361	0.01836165
ENSG00000146859	1.30814281	0.00710113
ENSG00000146918	-1.2729639	0.00022946
ENSG00000147113	1.32372962	0.00096949
ENSG00000147133	-1.2277004	2.65E-08
ENSG00000147155	-1.2840923	7.72E-09

Gene ID	FC 0NG - 24HG	adj p-value 0NG-24HG
ENSG00000147180	1.40909868	0.00020372
ENSG00000147383	-1.312417	3.23E-08
ENSG00000147536	-1.2163042	0.01593243
ENSG00000147852	-1.4280485	0.00044757
ENSG00000147853	1.20253924	0.01018611
ENSG00000147872	1.34790923	1.17E-06
ENSG00000148143	1.22714375	0.0021807
ENSG00000148671	-1.2912857	0.00027887
ENSG00000148677	1.49602592	4.55E-10
ENSG00000148700	-1.2952652	1.78E-05
ENSG00000148773	-1.6359894	1.45E-08
ENSG00000148842	1.42016104	0.00043355
ENSG00000148926	-1.447859	0.00015788
ENSG00000149212	1.56678935	2.05E-09
ENSG00000149269	1.36064751	0.00327525
ENSG00000149380	-1.2422782	6.8381E-05
ENSG00000149503	-1.4365852	1.50E-06
ENSG00000149531	1.20140352	0.03450535
ENSG00000149582	1.24946545	0.0007017
ENSG00000149798	-1.2678694	2.30E-07
ENSG00000149809	-1.3131769	0.02870696
ENSG00000149929	-1.2709113	0.00325812
ENSG00000150347	1.55926941	0.00017831
ENSG00000150551	1.34050564	6.29E-05
ENSG00000150593	1.26799211	0.00036092
ENSG00000150630	1.37082888	2.12E-06
ENSG00000150764	1.24792171	0.00316745
ENSG00000151093	1.25796119	0.01314593
ENSG00000151117	1.43379308	0.00120344
ENSG00000151388	1.2407635	0.00602413
ENSG00000151414	1.28451324	0.00800126
ENSG00000151458	1.2544708	0.00146551

Gene ID	FC 0NG - 24HG	adj p-value 0NG-24HG
ENSG00000151466	-1.2502228	5.71E-05
ENSG00000151690	-1.3760933	0.00079277
ENSG00000151725	-1.2595571	0.00438644
ENSG00000151789	1.2790269	0.00809347
ENSG00000152127	-1.2338469	2.79E-08
ENSG00000152137	1.24821979	0.00070283
ENSG00000152217	1.2501258	0.01851261
ENSG00000152229	1.24599341	0.00924879
ENSG00000152409	1.33943944	0.00665351
ENSG00000152778	1.25153101	0.0033002
ENSG00000152952	1.27473576	0.00044822
ENSG00000152953	-1.2164077	0.00443465
ENSG00000153044	-1.2131559	0.02458482
ENSG00000153982	1.28478907	0.00662682
ENSG00000154016	-1.2057323	0.01352054
ENSG00000154127	1.22358945	2.30E-05
ENSG00000154146	-1.2726844	1.35E-05
ENSG00000154258	1.37108245	0.00044702
ENSG00000154310	1.24275812	3.36E-07
ENSG00000154639	-1.295718	0.04025789
ENSG00000154678	1.30897086	0.00076595
ENSG00000154734	1.39764572	0.01611203
ENSG00000154920	-1.3298403	0.00237679
ENSG00000155307	1.25392366	0.03958164
ENSG00000155846	1.24028988	0.03263959
ENSG00000155970	1.23441389	0.04521317
ENSG00000156030	-1.2082189	0.00185021
ENSG00000156049	1.55054105	3.40E-05
ENSG00000156265	-1.2908377	0.00363535
ENSG00000156675	-1.372858	0.0036978
ENSG00000156711	1.48544863	0.00048373
ENSG00000156970	-1.4514125	5.59E-06

Gene ID	FC 0NG - 24HG	adj p-value 0NG-24HG
ENSG00000157168	1.59816297	2.65E-12
ENSG00000157214	1.52830193	2.78E-06
ENSG00000157240	1.2627328	0.02128507
ENSG00000157404	-1.6996626	8.41E-05
ENSG00000157456	-1.4434573	1.51E-07
ENSG00000157510	-1.2681207	8.38E-08
ENSG00000157557	-1.3797792	1.92E-12
ENSG00000157613	-1.9501731	1.80E-06
ENSG00000157693	1.23044467	0.0211893
ENSG00000158023	1.24206921	0.0172326
ENSG00000158186	1.30827875	0.00050415
ENSG00000158195	-1.2662903	1.59E-06
ENSG00000158286	1.48706038	0.00946952
ENSG00000158292	1.34199484	0.00015095
ENSG00000158402	-1.4937317	1.45E-06
ENSG00000158683	1.40871255	0.00112769
ENSG00000158716	1.42019858	5.55E-06
ENSG00000158856	-1.2699829	0.00012487
ENSG00000158859	-1.2229292	0.00540019
ENSG00000159167	-1.6765126	4.35E-05
ENSG00000159231	-1.2147989	0.00133358
ENSG00000160013	-1.38578	0.00438728
ENSG00000160285	-1.3294909	7.21E-06
ENSG00000160298	-1.3498722	0.01917261
ENSG00000160447	-1.2382122	4.00E-05
ENSG00000160752	-1.2755426	1.11E-07
ENSG00000160796	-1.2526107	0.00011847
ENSG00000160799	-1.2313266	0.00038217
ENSG00000161243	1.28440715	0.00721138
ENSG00000161618	-1.2232288	0.00035886
ENSG00000161642	1.22131478	0.00265638
ENSG00000161682	1.2382807	0.02196193

Gene ID	FC 0NG - 24HG	adj p-value 0NG-24HG
ENSG00000161791	-1.2703957	1.07E-09
ENSG00000161800	-1.5385099	3.67E-08
ENSG00000161888	-1.4254054	1.12E-06
ENSG00000161940	-1.2491292	1.40E-06
ENSG00000162063	-1.5093138	1.27E-06
ENSG00000162174	1.26447624	4.02E-06
ENSG00000162437	1.21827359	0.00584978
ENSG00000162496	1.35202661	0.00080127
ENSG00000162576	1.27268699	0.00029156
ENSG00000162614	1.38032519	1.30E-05
ENSG00000162639	1.23993094	0.00721019
ENSG00000162645	1.2328707	0.04279052
ENSG00000162692	1.47830926	0.00145234
ENSG00000162944	1.33325971	0.04158712
ENSG00000163132	-1.2540667	0.01188928
ENSG00000163171	-1.4283874	6.28E-09
ENSG00000163328	1.34252726	0.01563674
ENSG00000163344	-1.2074713	0.00105694
ENSG00000163633	1.26649697	0.00373776
ENSG00000163637	1.43424614	3.92E-06
ENSG00000163659	-1.2493811	1.64E-07
ENSG00000163661	1.31647204	0.00024582
ENSG00000163686	1.2033886	0.03188488
ENSG00000163739	1.43606276	0.00143564
ENSG00000163751	1.55594503	0.00023563
ENSG00000163814	-1.2293191	0.00015107
ENSG00000163867	1.3268282	0.0216739
ENSG00000163884	1.33341121	0.00046033
ENSG00000163947	-1.2350471	0.00171145
ENSG00000164035	1.25056602	0.04903336
ENSG00000164087	-1.3393912	0.00056229
ENSG00000164104	-1.5732975	1.43E-08
ENSG00000164109	-1.2409302	0.02876038
ENSG00000164125	1.3711679	0.00011094
ENSG00000164161	1.20215437	0.00052521
ENSG00000164283	1.28213867	0.00117058
ENSG00000164292	1.24560719	0.00017849
ENSG00000164463	1.21623666	0.03484785
ENSG00000164638	1.28471142	0.0338656
ENSG00000164647	1.24579441	0.0177879
ENSG00000164659	1.20235348	0.00053041
ENSG00000164663	1.45855177	8.31E-06
ENSG00000164687	1.50302313	1.81E-07
ENSG00000164692	1.30689311	0.01725159
ENSG00000164823	1.24260648	0.00354283
ENSG00000164849	1.51413226	0.01040997
ENSG00000164850	1.30678553	0.00052679
ENSG00000164949	1.33443799	0.01338644
ENSG00000164951	1.24724892	0.00028517
ENSG00000165029	2.70173502	2.92E-12
ENSG00000165092	2.42650549	5.24E-14
ENSG00000165240	1.21157348	0.01056009
ENSG00000165304	-1.3119009	8.49E-06
ENSG00000165312	1.27135396	0.02682146
ENSG00000165568	-1.2244009	0.01610906
ENSG00000165821	1.45410021	6.72E-05
ENSG00000165895	1.38208766	0.00039298
ENSG00000165899	1.30005675	0.00245383
ENSG00000165912	1.30483713	0.00010351
ENSG00000166046	1.3045942	0.02600242
ENSG00000166250	1.22847096	0.03029694
ENSG00000166265	1.31149177	0.00018912
ENSG00000166510	1.34909923	0.02107163
ENSG00000166670	-1.4959677	5.41E-06
ENSG00000166741	1.20561007	8.96E-05
ENSG00000166803	-1.2442709	0.00160786
ENSG00000166851	-1.5290425	1.59E-06
ENSG00000166897	1.44610923	9.66E-05
ENSG00000166900	1.23417539	1.75E-06
ENSG00000166963	1.41760974	0.00067798
ENSG00000167077	-1.3362062	0.00067343
ENSG00000167107	1.34907017	0.00011286
ENSG00000167508	-1.5183769	3.11E-08
ENSG00000167553	-1.2585983	3.54E-05
ENSG00000167644	-1.2331186	0.03247945
ENSG00000167772	1.47606069	0.00037116
ENSG00000167799	-1.2845024	0.00043546
ENSG00000167874	1.4109864	0.00021718
ENSG00000167900	-1.2269444	0.00158526
ENSG00000168016	1.300943	0.00016058
ENSG00000168077	-1.3251661	2.59E-08
ENSG00000168078	-1.2987162	0.01053397
ENSG00000168300	1.33971101	3.20E-05
ENSG00000168386	1.59908773	2.49E-06
ENSG00000168476	-1.3550749	1.59E-07
ENSG00000168528	-1.2544966	0.00036365
ENSG00000168672	1.47586864	4.98E-07
ENSG00000168679	1.26721114	0.01009639
ENSG00000168685	1.48533623	0.01378293
ENSG00000169126	1.2585389	0.01565267
ENSG00000169169	1.2025142	0.03730001
ENSG00000169282	1.20964887	0.01650588
ENSG00000169429	1.57331156	0.0290742
ENSG00000169604	-1.2506292	0.00024933
ENSG00000169607	-1.5379397	6.99E-06
ENSG00000169679	-1.5770327	1.68E-08

Gene ID	FC 0NG - 24HG	adj p-value 0NG-24HG
ENSG00000169710	-1.2669603	7.36E-05
ENSG00000169744	1.21371457	0.00023633
ENSG00000169760	1.33774398	0.00021955
ENSG00000169851	1.36383405	0.00151231
ENSG00000169908	-1.2530446	3.47E-05
ENSG00000170175	-1.2895652	0.00097213
ENSG00000170264	1.20810854	0.02754372
ENSG00000170271	1.34062064	0.00748497
ENSG00000170312	-1.5063779	8.12E-05
ENSG00000170323	2.2518535	1.73E-08
ENSG00000170364	1.21726963	0.01656674
ENSG00000170365	-1.321995	1.32E-05
ENSG00000170373	-1.3211984	0.00489932
ENSG00000170379	1.20667117	0.00100347
ENSG00000170385	1.34032851	0.00853636
ENSG00000170425	1.22867214	0.03163784
ENSG00000170456	1.3455333	1.37E-05
ENSG00000170522	-1.3091149	3.07E-06
ENSG00000170540	-1.2692033	0.03809109
ENSG00000170743	1.29835784	0.04271051
ENSG00000170775	1.27226087	1.73E-05
ENSG00000170915	-1.2586271	0.00118986
ENSG00000170921	1.26701164	0.00097738
ENSG00000171241	-1.2458765	0.0145395
ENSG00000171388	-1.5389821	3.64E-06
ENSG00000171729	-1.2153667	0.00899093
ENSG00000171791	-1.3063878	0.01685316
ENSG00000171843	1.30786056	0.00355136
ENSG00000171848	-1.2582169	0.00167363
ENSG00000172183	-1.5089823	4.39E-06
ENSG00000172260	1.25377074	0.02164442
ENSG00000172733	1.32056117	0.0081848

Gene ID	FC 0NG - 24HG	adj p-value 0NG-24HG
ENSG00000172819	1.30218734	0.00167475
ENSG00000172893	-1.3259915	1.45E-07
ENSG00000172915	1.27995677	0.01405612
ENSG00000173068	1.25009354	2.40E-05
ENSG00000173207	-1.4297345	3.71E-08
ENSG00000173334	-1.3079453	0.01126589
ENSG00000173597	1.44617659	0.00090246
ENSG00000174370	-1.3365142	0.0065334
ENSG00000174442	-1.2231791	0.00741384
ENSG00000174516	1.42517229	0.00017253
ENSG00000174718	1.25458625	0.02763499
ENSG00000174738	1.28680907	0.00054179
ENSG00000174951	-1.2057841	0.01945428
ENSG00000175048	1.23310149	0.00243076
ENSG00000175063	-1.4536666	2.13E-05
ENSG00000175155	1.27965854	0.00426028
ENSG00000175216	-1.2084016	9.72E-06
ENSG00000175264	-1.4108608	1.89E-07
ENSG00000175274	-1.3271209	2.76E-06
ENSG00000175414	1.26737553	3.93E-06
ENSG00000175471	1.57730848	9.94E-09
ENSG00000175567	-1.3253729	0.00127056
ENSG00000175591	1.26938651	0.03163892
ENSG00000175745	1.39363637	2.67E-09
ENSG00000175764	1.20113705	0.01977373
ENSG00000175832	-1.3473628	1.13E-06
ENSG00000175899	1.36007594	0.03069274
ENSG00000176046	1.46060059	0.00109726
ENSG00000176273	1.31136205	0.03950677
ENSG00000176438	-1.2617509	0.00011609
ENSG00000176658	-1.20335	0.00010592
ENSG00000176692	1.21178464	0.01506613

Gene ID	FC 0NG - 24HG	adj p-value 0NG-24HG
ENSG00000176697	1.64011873	0.00060215
ENSG00000176749	1.22593577	0.03888157
ENSG00000176890	-1.2077568	0.00043544
ENSG00000176973	1.21138707	0.01867714
ENSG00000177084	-1.2126957	0.0029349
ENSG00000177374	-1.2712093	0.02432301
ENSG00000177675	1.45817038	1.08E-06
ENSG00000178031	1.26859039	1.25E-06
ENSG00000178209	-1.2747908	0.00056834
ENSG00000178498	1.2585015	0.00785487
ENSG00000178695	1.22430438	0.00064708
ENSG00000178882	1.88006985	4.26E-08
ENSG00000178922	1.26397482	0.00015003
ENSG00000178999	-1.3556064	0.00098259
ENSG00000179094	1.24546875	0.00114854
ENSG00000179403	1.30513691	0.0387334
ENSG00000179750	-1.3254953	0.00135009
ENSG00000179855	1.27486971	0.04857813
ENSG00000180229	1.32026361	0.01355792
ENSG00000180263	1.22803303	0.00069381
ENSG00000180340	1.28411532	0.02062901
ENSG00000180354	1.29638189	0.00956482
ENSG00000180596	-1.2100927	0.02899808
ENSG00000180730	-1.2613698	0.00207842
ENSG00000180938	1.21712495	0.02466572
ENSG00000181104	1.25466048	0.0001223
ENSG00000181381	1.30846912	0.03326211
ENSG00000181444	1.23242249	0.04958126
ENSG00000181634	2.00349439	8.02E-06
ENSG00000182013	1.40557875	2.13E-05
ENSG00000182022	-1.3273434	4.98E-10
ENSG00000182118	-1.329218	0.02710505

Gene ID	FC 0NG - 24HG	adj p-value 0NG-24HG
ENSG00000182134	-1.2193241	0.00636572
ENSG00000182158	-1.2197547	3.31E-14
ENSG00000182481	-1.3239478	1.23E-09
ENSG00000182568	1.64663566	5.52E-06
ENSG00000182621	-1.2463244	0.02515424
ENSG00000182809	-1.2899221	1.06E-07
ENSG00000182836	1.29650861	0.04764788
ENSG00000182916	1.37417076	0.0006014
ENSG00000183287	1.24548775	0.01819096
ENSG00000183763	-1.4231112	0.00091359
ENSG00000183856	-1.520852	9.51E-08
ENSG00000183873	-1.505355	5.72E-08
ENSG00000184113	-1.2451507	0.00442512
ENSG00000184226	1.32644997	0.00366996
ENSG00000184254	-1.4379346	2.81E-11
ENSG00000184292	1.48927322	0.00192355
ENSG00000184357	-1.2236059	0.04427214
ENSG00000184371	1.25838645	0.00493215
ENSG00000184515	1.20425372	0.00680743
ENSG00000184661	-1.5056335	8.52E-06
ENSG00000184702	1.26183538	0.03628682
ENSG00000184792	-1.2906534	0.00061191
ENSG00000184867	1.25484902	0.00016974
ENSG00000185022	-1.205051	0.00120396
ENSG00000185070	1.40776267	3.28E-06
ENSG00000185215	1.36200519	2.06E-05
ENSG00000185262	-1.3011008	1.15E-05
ENSG00000185291	1.28008903	0.03395407
ENSG00000185338	-1.4462526	0.00101335
ENSG00000185477	1.32584138	0.00012107
ENSG00000185813	-1.3744451	1.15E-08
ENSG00000185860	1.24627866	0.03587815

Gene ID	FC 0NG - 24HG	adj p-value 0NG-24HG
ENSG00000185909	1.2172884	0.04860953
ENSG00000186185	-1.4226139	0.00021834
ENSG00000186193	-1.5326676	3.82E-09
ENSG00000186281	-1.4620605	1.18E-05
ENSG00000186377	1.26452003	0.04139588
ENSG00000186480	-1.3134676	0.00479493
ENSG00000186603	1.45762248	1.3572E-05
ENSG00000186638	-1.3175326	0.01334952
ENSG00000187266	-1.2673991	0.00082533
ENSG00000187479	1.38653493	0.0064777
ENSG00000187678	-1.4263116	7.85E-08
ENSG00000187741	-1.2231705	0.03207096
ENSG00000187840	-1.239478	0.00016622
ENSG00000187951	-1.4556327	0.00115617
ENSG00000188211	-1.2035991	9.85E-05
ENSG00000188229	-1.2298128	0.00012202
ENSG00000188277	-1.6815772	0.00590842
ENSG00000188343	1.24163453	6.61E-05
ENSG00000188486	-1.3269432	3.41E-05
ENSG00000189221	1.31727573	0.00021174
ENSG00000189319	1.20216043	0.00055613
ENSG00000196139	1.25752512	0.00879496
ENSG00000196155	1.30147331	2.22E-05
ENSG00000196411	1.24130044	2.09E-06
ENSG00000196421	-1.2675469	0.00592284
ENSG00000196549	-1.2215714	0.04968723
ENSG00000196569	1.27705525	0.00706543
ENSG00000196611	-1.9207645	5.86E-09
ENSG00000196632	1.23579321	0.00388769
ENSG00000196747	-1.2443341	0.03454226
ENSG00000196890	-1.2370508	0.03120443
ENSG00000197061	-1.2250351	0.02563221

Gene ID	FC 0NG - 24HG	adj p-value 0NG-24HG
ENSG00000197062	1.24709429	0.00422785
ENSG00000197119	1.20286886	0.02229233
ENSG00000197343	1.25089311	0.02017949
ENSG00000197555	1.27103891	2.17E-05
ENSG00000197566	1.24990938	0.01439674
ENSG00000197632	1.29217113	0.04474436
ENSG00000198721	1.27709118	1.16E-08
ENSG00000198743	1.36277879	0.00378358
ENSG00000198774	1.32169653	0.00632345
ENSG00000198816	1.21486841	0.00036697
ENSG00000198830	-1.2091539	9.25E-07
ENSG00000198832	1.2466879	0.00836184
ENSG00000198844	-1.2757921	0.00025538
ENSG00000198846	1.90566188	5.51E-09
ENSG00000198873	-1.3876756	1.74E-09
ENSG00000198901	-1.4533612	5.15E-08
ENSG00000198911	-1.2047134	1.31E-06
ENSG00000198947	1.20038614	0.02360634
ENSG00000203760	-1.2796864	2.16E-05
ENSG00000203805	1.31533097	0.00584911
ENSG00000203814	-1.2061147	0.01552576
ENSG00000203883	-1.4376828	0.00131915
ENSG00000204103	1.47662803	5.00E-05
ENSG00000204403	1.28922814	0.00036732
ENSG00000204442	1.2360544	0.01834328
ENSG00000204682	1.2985045	0.03729949
ENSG00000205181	1.24154239	0.04752812
ENSG00000205978	1.20714506	0.01307721
ENSG00000206538	1.4482249	1.39E-05
ENSG00000213190	1.27074924	0.00035732
ENSG00000213347	-1.5696488	2.65E-07
ENSG00000213462	1.24742167	0.04797021

Gene ID	FC 0NG - 24HG	adj p-value 0NG-24HG
ENSG00000214357	-1.7408713	2.76E-05
ENSG00000215223	-1.2021634	0.01121131
ENSG00000215845	-1.2028989	0.00162743
ENSG00000216775	-1.5065542	6.43E-06
ENSG00000221829	-1.2404687	0.00240822
ENSG00000221866	1.42692785	0.00172892
ENSG00000222009	1.22645052	0.02092716
ENSG00000225554	1.2206105	0.02737791
ENSG00000225830	1.3103759	5.51E-06
ENSG00000226476	1.32079415	0.01970672
ENSG00000226702	1.29958454	0.00216652
ENSG00000226823	1.2225873	0.04111842
ENSG00000227517	1.39665314	0.00168175
ENSG00000228495	1.27397534	2.2302E-05
ENSG00000228672	1.22459489	0.02196924
ENSG00000229372	1.28886685	0.02617536
ENSG00000230330	-1.292221	0.01087435
ENSG00000230753	-1.2430057	0.00674921
ENSG00000232527	1.25109402	0.00249538
ENSG00000232931	-1.5463909	0.003739
ENSG00000233117	-1.4628793	0.0004878
ENSG00000233223	1.24474342	0.03532405
ENSG00000233450	-1.2188066	0.00404991
ENSG00000233822	-1.2911222	0.00675623
ENSG00000234964	1.23265624	0.03865191
ENSG00000235109	1.35634645	0.00053271
ENSG00000235217	1.60597572	8.84E-07
ENSG00000235423	1.20090637	0.00473199
ENSG00000235505	1.41074732	0.00561509
ENSG00000235750	-1.4345009	2.30E-08
ENSG00000235770	1.30979371	6.27E-06
ENSG00000235823	-1.2576647	0.02457634

Gene ID	FC 0NG - 24HG	adj p-value 0NG-24HG
ENSG00000237172	1.23665724	0.00717234
ENSG00000239899	1.22531176	0.03391972
ENSG00000240694	-1.7413348	2.73E-15
ENSG00000241015	-1.2983341	0.0005522
ENSG00000242265	-1.4531791	8.65E-13
ENSG00000242779	1.20338688	0.02215626
ENSG00000244300	1.30731569	0.00012286
ENSG00000248187	1.24457772	0.01333882
ENSG00000249279	-1.2292366	0.04993753
ENSG00000249992	-1.2107071	0.00736157
ENSG00000250303	1.44213908	7.34E-06
ENSG00000250510	-1.2046032	0.04085811
ENSG00000251602	1.22461983	0.0402264
ENSG00000253522	1.21612667	0.01271316
ENSG00000254004	1.22329363	0.01526903
ENSG00000254481	-1.2188923	0.03854915
ENSG00000254726	1.26307952	0.00025789
ENSG00000255248	1.46466189	9.35E-05
ENSG00000257913	1.27845749	0.02995909
ENSG00000261253	-1.3587698	0.00046588
ENSG00000261373	1.20545695	0.0049171
ENSG00000261377	1.26282829	0.0065013
ENSG00000263155	-1.2627579	0.0374254
ENSG00000263528	1.25017094	0.00011387
ENSG00000265972	1.25335258	0.00356109
ENSG00000267194	1.38790686	0.00148403
ENSG00000267365	1.37826289	0.04971274
ENSG00000267534	1.26089978	0.00971549
ENSG00000270547	1.57891458	1.3328E-05
ENSG00000272168	1.3958814	0.00278847
ENSG00000272405	-1.386492	0.0180131
ENSG00000272941	1.29084721	0.00496817

Gene ID	FC 0NG - 24HG	adj p-value 0NG-24HG
ENSG00000273466	1.23447214	0.01280256
ENSG00000274712	1.30065343	0.00292697
ENSG00000275126	-1.2381101	0.02906482
ENSG00000275342	-1.2149346	0.01353073
ENSG00000275713	-1.3662786	0.00483026
ENSG00000276550	1.29724427	0.03572866
ENSG00000276849	-1.3934633	0.02387868
ENSG00000277734	-1.3003977	0.00021578
ENSG00000277775	-1.4827593	7.49E-05
ENSG00000279369	1.23298311	0.03342398

Supplementary Table 4a. Differentially expressed genes between 0.5hr high glucose and 1hr high glucose samples

Ensembl gene IDs, Fold change (0.5HG-1HG) and FDR adjusted p-values of DEGs

Gene ID	FC 0.5HG - 1HG	adj p-value 0.5HG - 1HG
ENSG00000005513	-2.9145479	1.26E-12
ENSG00000006327	-1.2285406	0.00012568
ENSG00000006459	-1.5672156	8.49E-07
ENSG00000013441	-1.2825791	0.03196299
ENSG00000019144	-1.2604589	2.14E-05
ENSG00000019549	-1.8468986	7.54E-08
ENSG00000023445	-1.7147061	0.00347919
ENSG00000054598	-1.700264	6.62E-09
ENSG00000054967	-1.5714074	3.27E-06
ENSG00000059728	-1.2547118	0.00390412
ENSG00000067082	1.26677282	0.00014667
ENSG00000069399	-1.3572465	0.0003181
ENSG00000069667	-1.3573211	0.02163223
ENSG00000070404	-1.2658619	0.00326745
ENSG00000070610	-1.2716377	0.0306172
ENSG00000070669	1.25265568	0.00497177
ENSG00000073756	-2.3510258	0.0004569
ENSG00000075426	-1.538974	2.78E-10
ENSG00000079337	-1.3458659	0.0019662
ENSG00000081386	1.28132923	9.04E-05
ENSG00000084093	-1.4489206	4.40E-07
ENSG00000087448	-1.2337365	0.00069603
ENSG00000087494	-1.5938582	0.00027571
ENSG00000088826	-1.3489971	1.12E-05
ENSG00000095739	-1.2563613	0.0278508
ENSG00000095752	-2.0628752	1.22E-07
ENSG00000097096	-1.2880156	0.03727972
ENSG00000099860	-1.6674098	1.58E-06
ENSG00000100276	-1.2609155	0.01896074
ENSG00000100311	-1.399743	0.0025836
ENSG00000100625	-1.2574933	0.04891469
ENSG00000100906	-1.6212528	2.36E-09

Gene ID	FC 0.5HG - 1HG	adj p-value 0.5HG - 1HG
ENSG00000101384	-1.4203264	4.14E-05
ENSG00000101665	-7.8715358	3.52E-18
ENSG00000102034	-1.2335431	0.00014971
ENSG00000102890	-1.5351781	0.00222341
ENSG00000103257	-1.334721	1.84E-06
ENSG00000104081	-1.6269174	0.01327738
ENSG00000105072	1.25977182	0.01225061
ENSG00000105656	-1.2143734	0.00180744
ENSG00000105722	-1.531858	6.01E-09
ENSG00000105991	1.25821682	0.02105972
ENSG00000106003	-2.1986391	2.67E-08
ENSG00000106089	-1.6789894	1.73E-06
ENSG00000106560	1.24183068	0.01489298
ENSG00000107807	-1.3088164	0.03190493
ENSG00000107821	-1.5654479	0.00181935
ENSG00000107968	-1.463746	0.00967675
ENSG00000108551	-1.2610186	0.00882901
ENSG00000109118	1.20774686	0.00028038
ENSG00000109787	-1.327116	3.98E-08
ENSG00000110330	-1.2680809	0.01143787
ENSG00000110888	-1.2594876	0.00882777
ENSG00000111276	1.20487244	0.0133137
ENSG00000111859	-1.7365672	4.37E-07
ENSG00000112406	-1.2467564	0.01049915
ENSG00000112715	-2.1313542	6.14E-10
ENSG00000113070	-1.3451996	0.0022835
ENSG00000113369	1.43399094	2.73E-05
ENSG00000113742	-1.3410505	1.04E-05
ENSG00000114019	-1.4084468	1.38E-07
ENSG00000114315	-2.2130668	4.92E-06
ENSG00000114529	-1.2946979	0.01508162
ENSG00000115548	-1.2307251	0.00022383

Gene ID	FC 0.5HG - 1HG	adj p-value 0.5HG - 1HG
ENSG00000115738	-2.9183407	2.97E-09
ENSG00000115844	-2.7439416	9.16E-14
ENSG00000116205	1.22779203	0.0104546
ENSG00000116285	-1.6342141	6.36E-09
ENSG00000116604	-1.2438275	0.00104202
ENSG00000116717	1.30665533	1.41E-05
ENSG00000116731	-1.2731665	6.11E-07
ENSG00000116741	1.30716545	0.00698346
ENSG00000117036	-1.2432745	2.71E-06
ENSG00000117318	-1.6292731	1.04E-09
ENSG00000117479	-1.2914167	0.00890874
ENSG00000118263	-1.3799781	4.26E-07
ENSG00000118503	-1.3151182	0.03573442
ENSG00000118515	-1.4548391	1.55E-13
ENSG00000119508	-2.8713781	6.53E-09
ENSG00000119669	-1.6509136	2.85E-09
ENSG00000120696	1.32679884	0.03697574
ENSG00000121966	1.3693816	0.00011559
ENSG00000122863	-1.6864683	3.73E-09
ENSG00000123095	-1.4755253	0.00194654
ENSG00000123358	-2.5241345	2.61E-07
ENSG00000124019	1.33624958	0.00014101
ENSG00000124216	-2.4127166	1.33E-13
ENSG00000124762	-1.2151395	8.93E-08
ENSG00000125266	-1.2400309	0.02389437
ENSG00000125378	1.3448071	0.00294773
ENSG00000125740	-1.973717	0.01272246
ENSG00000125812	-1.278994	0.00005039
ENSG00000125945	1.2537369	0.00087662
ENSG00000125968	-1.9953464	5.43E-07
ENSG00000126368	1.36430304	0.00286324
ENSG00000126603	-1.3581156	0.00025177

Gene ID	FC 0.5HG - 1HG	adj p-value 0.5HG - 1HG
ENSG00000127080	-1.469602	2.85E-09
ENSG00000128016	1.42423781	0.00571941
ENSG00000128283	-1.2531925	1.33E-05
ENSG00000128594	-3.6896106	3.70E-14
ENSG00000128917	-1.3159967	0.03841575
ENSG00000130222	-1.4421077	0.00059229
ENSG00000130513	-1.2785266	1.82E-05
ENSG00000131016	-1.2281407	5.05E-05
ENSG00000131759	-1.4294527	8.39E-05
ENSG00000132326	-1.4536554	0.00030472
ENSG00000132481	-1.2495372	0.01337384
ENSG00000132510	-2.102206	1.66E-14
ENSG00000133561	1.26298487	1.25E-05
ENSG00000133985	-1.3187634	0.00194451
ENSG00000134107	-1.9636282	2.48E-15
ENSG00000134668	-1.2533504	0.01339746
ENSG00000135547	-2.3179947	7.33E-08
ENSG00000135625	-1.4183117	5.17E-05
ENSG00000135723	-1.2046225	0.00013812
ENSG00000136603	-1.837295	7.17E-10
ENSG00000136826	-1.8902241	9.89E-09
ENSG00000137331	1.21111552	0.02462989
ENSG00000137834	-2.797161	1.98E-08
ENSG00000138166	-1.6510865	1.79E-08
ENSG00000138449	1.21894655	0.01627947
ENSG00000138623	-1.9239497	9.66E-06
ENSG00000138675	-1.344949	0.00671491
ENSG00000138835	-1.2928474	0.00361203
ENSG00000139263	1.24925147	0.00017937
ENSG00000139289	1.23691477	0.00752985
ENSG00000141034	1.20428865	0.00026174
ENSG00000141526	-1.2571338	9.83E-06

Gene ID	FC 0.5HG - 1HG	adj p-value 0.5HG - 1HG
ENSG00000141858	-1.2302864	0.00040912
ENSG00000142178	-1.4318244	0.00160645
ENSG00000142279	-1.3815501	3.71E-05
ENSG00000142408	-1.5291031	2.77E-06
ENSG00000142627	-1.2520209	4.55E-05
ENSG00000143067	-1.6074722	4.93E-07
ENSG00000143322	-1.2898634	1.87E-07
ENSG00000143367	-1.3055546	0.00330669
ENSG00000143384	-1.2246994	1.24E-05
ENSG00000143878	-1.30561	0.00472446
ENSG00000144026	1.24072574	0.01353972
ENSG00000144136	-1.213879	7.94E-05
ENSG00000144655	-2.1544379	1.80E-16
ENSG00000145632	2.26504107	3.81E-16
ENSG00000146070	1.20071988	0.01333278
ENSG00000146232	-1.7023382	1.75E-11
ENSG00000148339	-1.3154188	0.00097787
ENSG00000148677	-1.3371097	4.59E-06
ENSG00000148841	-1.2071918	6.93E-05
ENSG00000148926	1.63957615	3.94E-06
ENSG00000149289	-1.3747741	1.68E-05
ENSG00000149591	-1.2459783	0.01530385
ENSG00000151014	-1.3157591	1.25E-07
ENSG00000151748	-1.3589996	4.83E-06
ENSG00000151967	-1.322939	0.01316666
ENSG00000153714	1.35291768	0.01042308
ENSG00000153879	-1.2191849	0.0049904
ENSG00000153885	-1.4475233	7.81E-09
ENSG00000154309	1.30885412	0.01595028
ENSG00000155090	-1.8962658	3.81E-09
ENSG00000155760	-1.3845409	2.73E-06
ENSG00000156030	-1.2374605	0.00109726

Gene ID	FC 0.5HG - 1HG	adj p-value 0.5HG - 1HG
ENSG00000156273	-1.5591143	1.34E-08
ENSG00000156983	-1.2073291	0.0001524
ENSG00000157240	-1.5345954	2.86E-05
ENSG00000158859	-1.2823731	0.00113619
ENSG00000159216	-1.2008301	0.01368157
ENSG00000160888	-1.2726825	7.15E-05
ENSG00000161835	-1.6956194	4.81E-10
ENSG00000161940	-1.222164	4.08E-05
ENSG00000162407	-1.3754675	0.00447256
ENSG00000162413	-1.3953945	1.09E-08
ENSG00000162702	-1.2756623	0.00014946
ENSG00000163376	-1.3244711	0.04811837
ENSG00000163545	-1.7565318	3.41E-05
ENSG00000163635	-1.2036846	1.17E-05
ENSG00000163659	-1.3068719	1.38E-08
ENSG00000163874	-1.7788887	8.17E-07
ENSG00000164056	1.93372495	1.18E-06
ENSG00000164442	1.28522795	0.02319588
ENSG00000164683	-3.9099122	7.74E-15
ENSG00000164855	-1.2606347	0.03561896
ENSG00000165507	2.57913935	1.07E-08
ENSG00000165572	1.22052863	0.00053948
ENSG00000165655	-1.4154058	0.00106417
ENSG00000166289	1.41117747	4.80E-05
ENSG00000166401	-1.2394184	2.02E-06
ENSG00000166471	-1.2170592	0.02540346
ENSG00000166483	-1.3305211	0.00114005
ENSG00000166886	-2.790299	5.54E-16
ENSG00000166924	1.28236827	0.04596447
ENSG00000167470	-1.7578297	8.33E-15
ENSG00000167565	-1.3293748	3.50E-05
ENSG00000167772	-1.6812154	1.20E-05

Gene ID	FC 0.5HG - 1HG	adj p-value 0.5HG - 1HG
ENSG00000168209	-1.3965546	0.01101328
ENSG00000168286	1.20138855	0.00293588
ENSG00000168386	-1.4188529	0.0010458
ENSG00000168646	1.38547187	0.00570229
ENSG00000168672	1.23403015	0.01439732
ENSG00000168874	-1.9237739	5.56E-08
ENSG00000168994	-1.2626155	8.33E-06
ENSG00000169242	-1.3832984	0.02255575
ENSG00000169641	-1.2127238	2.89E-09
ENSG00000169826	-1.2013359	0.00932545
ENSG00000169926	-1.2672069	0.00036536
ENSG00000169991	-1.2513403	7.36E-05
ENSG00000170345	2.68009941	6.14E-08
ENSG00000170385	-1.2890998	0.04947993
ENSG00000170525	-1.2702425	2.04E-07
ENSG00000170852	-1.3820691	5.17E-08
ENSG00000171017	1.24868993	0.04494838
ENSG00000171223	-1.9056705	2.52E-13
ENSG00000171246	-2.037146	1.73E-07
ENSG00000171604	-1.7655446	3.02E-06
ENSG00000171617	-1.3657224	3.8711E-05
ENSG00000171621	-1.3726799	0.01985822
ENSG00000171889	-1.259695	0.02817567
ENSG00000172059	-1.3864072	0.00031596
ENSG00000172216	-1.2955512	0.00055541
ENSG00000172602	-1.3199539	0.0417958
ENSG00000173011	-1.2289583	6.21E-05
ENSG00000173013	-1.3048173	0.03025651
ENSG00000173276	-1.2079066	0.00889643
ENSG00000173281	-1.3145892	9.59E-07
ENSG00000173846	-1.2302243	0.01124756
ENSG00000174738	-1.2024265	0.0312219

Gene ID	FC 0.5HG - 1HG	adj p-value 0.5HG - 1HG
ENSG00000175105	-1.6177268	0.00059323
ENSG00000175505	-1.3021523	0.01700536
ENSG00000175592	-1.4857631	1.57E-06
ENSG00000175906	1.53330826	4.16E-06
ENSG00000176170	-1.2200526	0.00275343
ENSG00000176692	-1.8302439	5.78E-11
ENSG00000176749	-1.3022429	0.00866366
ENSG00000176845	-1.3764794	0.00057473
ENSG00000176907	-1.7182232	5.14E-05
ENSG00000177125	-1.424103	6.70E-07
ENSG00000177283	-1.2699072	0.00080197
ENSG00000177426	1.24094809	0.00021
ENSG00000177679	-1.547914	7.24E-05
ENSG00000178996	-1.236951	4.80E-05
ENSG00000179144	1.25986191	0.00025755
ENSG00000179348	-1.8135742	3.04E-09
ENSG00000179361	-1.3542091	0.00075762
ENSG00000179388	-2.1693055	0.00038071
ENSG00000179604	-1.2153133	0.00424616
ENSG00000180592	-1.3921238	0.00236579
ENSG00000180787	1.37550102	0.00146021
ENSG00000180884	1.31725582	0.00100553
ENSG00000181274	1.32340205	0.00102353
ENSG00000181472	-1.6303057	3.19E-07
ENSG00000182196	-1.2053229	0.02105698
ENSG00000182310	-1.5016898	0.00020242
ENSG00000183072	-1.2175258	0.03515754
ENSG00000183196	1.21794999	0.02748142
ENSG00000183208	1.24776746	0.04942574
ENSG00000183307	-1.2867184	0.03567388
ENSG00000183337	-2.1106457	8.35E-10
ENSG00000183691	-2.3918739	1.19E-09

Gene ID	FC 0.5HG - 1HG	adj p-value 0.5HG - 1HG
ENSG00000184058	-1.2048823	0.04124431
ENSG00000184307	-1.5755809	0.0002551
ENSG00000184402	-1.2544874	0.0008836
ENSG00000184557	-1.6652957	7.92E-07
ENSG00000184588	1.38473781	8.15E-08
ENSG00000184898	1.27595	0.01161894
ENSG00000184916	-1.2596967	0.00027685
ENSG00000185015	-1.2411699	0.03713989
ENSG00000185022	-1.2986062	2.4337E-05
ENSG00000185112	1.20181748	0.00291475
ENSG00000185252	1.31306502	0.00051506
ENSG00000186480	-1.3276939	0.00708715
ENSG00000187479	-1.3178162	0.04895706
ENSG00000187566	1.29230871	0.00285779
ENSG00000187626	1.38871935	0.0009534
ENSG00000187678	-1.8471284	2.90E-13
ENSG00000188033	1.29839592	0.00872866
ENSG00000188206	-1.4576689	0.00837391
ENSG00000188290	-1.2642837	0.01952587
ENSG00000188295	-1.3576429	0.00188702
ENSG00000188483	-2.3781657	9.09E-18
ENSG00000188522	-1.5357817	1.05E-07
ENSG00000188997	1.23886485	0.01107718
ENSG00000189114	-1.2351501	0.01309005
ENSG00000189298	1.31454996	0.01493574
ENSG00000196428	-1.3798193	1.46E-09
ENSG00000196449	-1.2359258	5.70E-05
ENSG00000196843	-1.5351695	1.83E-06
ENSG00000197019	-1.7729149	1.78E-12
ENSG00000197063	-1.2522673	1.32E-05
ENSG00000197182	-1.4259714	0.00759349
ENSG00000197183	-1.2829344	8.34E-08

Gene ID	FC 0.5HG - 1HG	adj p-value 0.5HG - 1HG
ENSG00000197461	-1.9935658	1.82E-12
ENSG00000197566	1.24543663	0.03047403
ENSG00000197951	1.21079923	0.0278796
ENSG00000198142	-1.2331985	0.04939127
ENSG00000198182	1.28594366	0.00746925
ENSG00000198324	-1.2186154	0.0016796
ENSG00000198331	1.21286395	0.02328372
ENSG00000198435	-1.8171789	1.04E-08
ENSG00000198517	-1.7619273	2.59E-13
ENSG00000198774	1.27189302	0.04187639
ENSG00000203709	1.88200457	6.81E-06
ENSG00000203883	-1.4440326	0.00287578
ENSG00000204611	1.22086844	0.00113125
ENSG00000206448	-1.2327287	0.02098112
ENSG00000213203	1.22392212	0.01642822
ENSG00000218891	-1.2193802	0.03123354
ENSG00000228672	-1.2986339	0.00418616
ENSG00000229729	-1.5312521	1.72E-05
ENSG00000230082	1.27688279	0.04312771
ENSG00000239218	-1.2253844	0.02736703
ENSG00000251493	-1.4810866	0.00107317
ENSG00000253368	-1.3073836	8.34E-06
ENSG00000253669	-1.2191681	0.0416177
ENSG00000253958	-1.3634141	0.00111658
ENSG00000254470	-1.2695613	2.33E-06
ENSG00000255176	-1.7039789	0.00115016
ENSG00000256448	-1.2264358	0.00041414
ENSG00000256771	1.25436433	0.04177322
ENSG00000257453	1.33193682	0.02870243
ENSG00000261659	1.2953579	0.02635366
ENSG00000267882	-1.2188092	0.03234773
ENSG00000272398	-1.2385482	0.0165911

Gene ID	FC 0.5HG - 1HG	adj p-value 0.5HG - 1HG
ENSG00000272489	-1.2053642	0.03662026
ENSG00000272574	-1.2873254	0.00306722
ENSG00000273489	-1.8769958	4.26E-06
ENSG00000273820	-1.448724	0.00040913
ENSG00000274349	1.29946149	0.02056771
ENSG00000274636	-1.7469322	5.59E-05
ENSG00000274995	-1.8089201	1.58E-05
ENSG00000275538	1.2955828	0.04951477
ENSG00000275993	-1.9381058	5.82E-09
ENSG00000276649	-1.3834813	0.00104953
ENSG00000277013	-1.2713829	0.01376452
ENSG00000277400	1.20354755	0.04385285
ENSG00000278921	-1.2480208	0.04747297
ENSG00000279227	-1.2506979	0.04715625
ENSG00000279838	1.26810307	0.03050534
ENSG00000280138	-1.5073969	0.00579924
ENSG00000280543	-1.2495789	0.02061941
ENSG00000281332	-1.2612036	0.03044032
ENSG00000281766	-1.2751107	0.02215626

Supplementary Table 4b. Differentially expressed genes between 1hr high glucose and 4hr high glucose samples

Ensembl gene IDs, Fold change (1HG-4HG) and FDR adjusted p-values of DEGs

Gene ID	FC 1HG - 4HG	adj p-value 1HG - 4HG
ENSG00000001167	1.42409031	3.68E-10
ENSG00000001460	-1.2523766	0.0250699
ENSG00000002587	1.82814178	8.50E-11
ENSG00000003400	-1.3577278	1.58E-08
ENSG00000003989	1.7004672	2.85E-06
ENSG00000004139	-1.2287733	0.01109619
ENSG00000004660	-1.6604222	7.46E-08
ENSG00000005108	-1.241783	0.02811289
ENSG00000005513	-2.2862279	2.05E-10
ENSG00000005700	1.21168557	0.04714978
ENSG00000006062	-1.2835819	0.00705679
ENSG00000006468	-1.3829652	8.85E-05
ENSG00000006576	1.26463954	0.0122206
ENSG00000006652	1.31572531	0.00126944
ENSG00000007384	-1.5252637	3.55E-07
ENSG00000007908	8.48800745	9.43E-14
ENSG00000007968	-1.6876793	1.1034E-05
ENSG00000008083	-1.5617518	1.79E-08
ENSG00000008294	1.5099386	4.37E-18
ENSG00000008441	-1.2281188	8.77E-08
ENSG00000010318	-1.2437402	0.03628777
ENSG00000010810	-1.3302175	9.20E-10
ENSG00000011260	1.20925199	3.86E-05
ENSG00000011405	1.23618643	0.00427274
ENSG00000011422	-1.3420857	3.91E-06
ENSG00000011523	-1.2807647	1.44E-07
ENSG00000012048	-1.2475166	0.01332533
ENSG00000013293	-1.2974732	0.00863452
ENSG00000013441	-1.3015095	0.0105524
ENSG00000013573	-1.2579192	0.00388983
ENSG00000013588	-1.4529502	7.46E-07
ENSG00000013810	-1.327084	0.00107327

Gene ID	FC 1HG - 4HG	adj p-value 1HG - 4HG
ENSG00000015133	-2.454096	1.68E-17
ENSG00000015568	1.41932481	0.00091576
ENSG00000019144	1.28408748	1.12E-06
ENSG00000019549	-1.4020941	0.00085539
ENSG00000020577	1.30919651	1.49E-11
ENSG00000021355	-1.2088802	0.00623676
ENSG00000021645	1.22021743	0.04730423
ENSG00000021762	-1.3835179	1.94E-08
ENSG00000026559	1.26115791	0.01715718
ENSG00000026652	-1.2000662	0.0063828
ENSG00000026950	-1.2280861	0.00089215
ENSG00000028137	-1.2927784	0.00054833
ENSG00000028277	-1.5495939	2.80E-06
ENSG00000031081	1.44833931	3.62E-15
ENSG00000033327	-1.460428	4.31E-14
ENSG00000034533	1.23202571	0.01910318
ENSG00000034677	-1.3168419	0.00061685
ENSG00000035499	-1.2818137	0.00231304
ENSG00000038295	1.69221737	8.49E-08
ENSG00000039319	1.2465474	0.03023402
ENSG00000043143	-1.9285371	4.58E-13
ENSG00000047188	1.25005755	4.06E-05
ENSG00000047579	-1.2195011	0.00045729
ENSG00000047617	-1.346892	0.00033638
ENSG00000047932	1.3457012	0.00058015
ENSG00000048052	1.69322712	5.30E-05
ENSG00000049130	1.85972239	7.13E-05
ENSG00000049192	1.41266732	1.12E-08
ENSG00000049283	-1.7243612	0.00011816
ENSG00000049759	1.76138841	3.93E-13
ENSG00000049883	1.23766694	0.003424
ENSG00000050820	-1.3252456	6.31E-09

Gene ID	FC 1HG - 4HG	adj p-value 1HG - 4HG
ENSG00000051180	-1.3672886	0.00144242
ENSG00000051341	-1.2218271	0.04037422
ENSG00000052749	1.21998684	3.09E-05
ENSG00000053747	-1.250317	0.00080887
ENSG00000054598	-1.5626198	6.51E-08
ENSG00000054967	-1.9947334	2.02E-11
ENSG00000055813	1.21887743	0.04474436
ENSG00000056558	2.0150458	0.00017421
ENSG00000056972	-1.2751942	0.00364558
ENSG00000057019	1.38090484	4.64E-06
ENSG00000057252	1.22693457	0.00857857
ENSG00000057294	-1.5335803	0.00017788
ENSG00000057704	-1.9686509	4.18E-17
ENSG00000058091	1.32531749	4.85E-06
ENSG00000058453	-1.3205652	0.00019425
ENSG00000059378	-1.2752516	1.82E-05
ENSG00000059728	-1.2406522	0.00292222
ENSG00000060491	-1.2184787	0.0002181
ENSG00000061938	-1.2475866	4.46E-06
ENSG00000062282	-1.2617746	0.04630962
ENSG00000063438	-1.2376114	0.00381988
ENSG00000064692	-2.0856542	6.76E-07
ENSG00000064989	1.62153902	1.34E-05
ENSG00000064999	-1.4454882	7.24E-08
ENSG00000065054	1.25561464	0.00070937
ENSG00000065183	1.25229534	3.47E-09
ENSG00000065308	-1.599023	3.62E-15
ENSG00000065328	-1.2649958	0.01562412
ENSG00000065559	1.20377422	0.00136888
ENSG00000065802	-1.3520175	2.70E-12
ENSG00000066027	-1.3207536	0.00011947
ENSG00000066084	1.25390964	3.64E-05

Gene ID	FC 1HG - 4HG	adj p-value 1HG - 4HG
ENSG00000066629	1.21488283	1.21E-05
ENSG00000066651	1.24798434	0.02661467
ENSG00000066735	-1.7513509	1.37E-11
ENSG00000067182	-1.2127455	1.77E-06
ENSG00000067798	1.67640482	4.40E-17
ENSG00000067955	1.8842319	2.52E-11
ENSG00000068024	-1.2722199	3.74E-06
ENSG00000068078	1.6704118	3.39E-09
ENSG00000068137	-1.5992029	7.53E-06
ENSG00000068305	1.37146571	1.81E-06
ENSG00000068366	1.26124238	1.85E-05
ENSG00000068724	-1.2178845	0.00129711
ENSG00000069399	-1.4003099	1.93E-05
ENSG00000069702	-1.4541307	1.0476E-05
ENSG00000069812	-1.3395773	5.11E-07
ENSG00000070444	-1.2657173	0.00050732
ENSG00000070476	-1.2310608	1.48E-05
ENSG00000070610	-1.9404218	3.46E-10
ENSG00000070614	-1.3051656	1.46E-09
ENSG00000070961	1.67772753	3.91E-09
ENSG00000071073	-1.2679188	7.47E-05
ENSG00000071246	-1.3914362	0.00119943
ENSG00000071282	-1.3235975	0.00772374
ENSG00000072201	-1.4235619	3.41E-05
ENSG00000072401	1.27750832	0.01228617
ENSG00000072422	1.24160854	1.43E-05
ENSG00000072840	-1.2725266	3.98E-06
ENSG00000072952	-1.2515834	0.011108
ENSG00000073008	1.36771412	8.70E-11
ENSG00000073111	-1.2384548	0.00354736
ENSG00000073331	-1.3972209	3.59E-06
ENSG00000073417	1.69490379	3.10E-17
ENSG00000073711	-1.3085741	0.00051582
ENSG00000073712	1.25645356	5.41E-08
ENSG00000073921	1.40892167	1.01E-06
ENSG00000074047	2.7111617	3.30E-15
ENSG00000074855	-1.3362401	0.00055472
ENSG00000075218	-1.2593185	0.01002836
ENSG00000075340	1.28733662	0.02241529
ENSG00000075426	-1.8086676	2.27E-15
ENSG00000075539	1.29871388	0.00011405
ENSG00000075643	-1.292746	0.00665499
ENSG00000075651	-1.6601034	5.08E-13
ENSG00000075702	-1.3812476	0.00036633
ENSG00000075711	-1.2812735	1.75E-05
ENSG00000075790	1.26685734	0.03528104
ENSG00000076351	-1.5351308	1.83E-07
ENSG00000076356	1.21300701	1.20E-05
ENSG00000077254	1.21618264	0.01189527
ENSG00000077585	1.2152711	0.04774712
ENSG00000078401	-1.3381014	3.31E-06
ENSG00000078687	-1.201622	0.00027235
ENSG00000078747	1.27089865	1.21E-06
ENSG00000079102	1.66058407	2.13E-07
ENSG00000079337	-1.4126846	8.12E-05
ENSG00000079432	-1.2238081	1.47E-05
ENSG00000079435	-1.2414385	0.0293323
ENSG00000079482	-1.2777609	3.35E-05
ENSG00000079616	-1.2679052	0.00064041
ENSG00000080561	-1.343184	4.75E-08
ENSG00000080608	1.28960526	1.32E-07
ENSG00000080839	-1.2443117	0.0235111
ENSG00000080845	-1.2008098	1.14E-05
ENSG00000080986	-1.2492223	0.0180566
ENSG00000081041	1.46894251	0.04262267
ENSG00000081059	1.59712967	3.45E-06
ENSG00000081386	1.34059575	1.01E-06
ENSG00000081913	1.2739374	0.01994426
ENSG00000081923	-1.26183	0.01588503
ENSG00000082126	-1.308208	1.6851E-05
ENSG00000082438	1.26203632	0.00029173
ENSG00000082512	-1.4276543	0.00021255
ENSG00000082516	1.22352632	9.86E-05
ENSG00000084093	1.22707264	0.00226688
ENSG00000084112	-1.2729692	1.07E-06
ENSG00000084710	1.51276571	8.05E-05
ENSG00000084734	-1.2484449	0.00807442
ENSG00000085117	-1.2377178	0.00068131
ENSG00000085185	-1.3555658	0.00020364
ENSG00000086015	-1.2604163	4.02E-06
ENSG00000086696	-1.277921	0.03062469
ENSG00000086848	1.20755908	0.0416177
ENSG00000086991	1.23703261	0.00548589
ENSG00000087074	-1.3735311	1.21E-09
ENSG00000087095	1.20606761	0.00069587
ENSG00000087266	-1.4542385	4.96E-09
ENSG00000087903	1.45298298	0.00250422
ENSG00000088179	-1.3629291	1.08E-05
ENSG00000088325	-1.2502151	0.00139601
ENSG00000088387	-1.3552865	1.05E-10
ENSG00000088451	1.25238368	0.02511904
ENSG00000088836	-1.2763046	0.00060114
ENSG00000088876	1.32445163	0.00014254
ENSG00000089041	-2.0571825	1.54E-08
ENSG00000089048	1.26944932	9.85E-05
ENSG00000089177	-1.2057456	0.00470308

Gene ID	FC 1HG - 4HG	adj p-value 1HG - 4HG
ENSG00000089335	1.27850561	0.00351656
ENSG00000089351	-1.2254054	0.00125218
ENSG00000089505	-1.2367112	0.01690866
ENSG00000090238	-1.3471647	4.80E-05
ENSG00000090376	-1.2045227	0.02614018
ENSG00000090776	-1.4244764	1.60E-09
ENSG00000090889	-1.2739063	0.00183178
ENSG00000091129	1.44808683	3.18E-09
ENSG00000091428	1.33219539	0.00037451
ENSG00000091483	1.28043041	5.06E-07
ENSG00000091622	1.4185278	0.01337271
ENSG00000091656	1.66427498	7.30E-10
ENSG00000091844	-1.4687716	4.77E-05
ENSG00000091879	1.36309176	1.20E-07
ENSG00000091972	-1.4015522	0.00017547
ENSG00000092470	-1.2450971	0.00748264
ENSG00000092969	1.43283187	0.00604864
ENSG00000093009	-1.3306957	0.00814974
ENSG00000094804	-1.3680875	9.44E-05
ENSG00000095015	1.37809728	1.84E-07
ENSG00000095370	-1.6585797	1.23E-12
ENSG00000095383	-1.4045845	5.95E-05
ENSG00000095397	1.21340457	0.02323425
ENSG00000095637	-1.2877555	0.02781794
ENSG00000095752	1.50599402	0.00080256
ENSG00000096093	-1.2320845	0.00502463
ENSG00000096433	-1.2522032	1.34E-07
ENSG00000097046	-1.2282054	0.02365703
ENSG00000097096	-1.2927755	0.01849512
ENSG00000099204	-1.3027999	6.14E-06
ENSG00000099282	-1.2428492	0.00683651
ENSG00000099822	1.45924514	0.00181839

Gene ID	FC 1HG - 4HG	adj p-value 1HG - 4HG
ENSG00000099860	-3.1201528	6.06E-17
ENSG00000099875	-1.3612401	7.00E-08
ENSG00000099889	-1.6762067	2.20E-10
ENSG00000099910	-1.2163548	0.01586997
ENSG00000100027	-1.3615212	0.00949273
ENSG00000100034	-1.3467473	1.19E-10
ENSG00000100036	-1.6357825	6.16E-11
ENSG00000100060	-1.2032	0.00010424
ENSG00000100065	-1.4730493	5.65E-09
ENSG00000100221	-1.2159369	4.08E-09
ENSG00000100292	-1.5786916	5.12E-09
ENSG00000100297	-1.2037884	0.00139787
ENSG00000100425	-1.2142491	7.40E-06
ENSG00000100439	-1.2822836	0.0027739
ENSG00000100490	-1.3248254	0.00106
ENSG00000100523	1.23274421	0.00470599
ENSG00000100580	1.23164662	0.00010307
ENSG00000100592	1.29687924	4.91E-08
ENSG00000100625	-1.3036753	0.00810695
ENSG00000100629	-1.3089844	0.00635593
ENSG00000100647	-1.2949773	1.61E-10
ENSG00000100697	1.21206187	0.00040385
ENSG00000100889	-1.219266	0.00567705
ENSG00000100906	-1.8636132	2.36E-13
ENSG00000101003	-1.2571619	0.0121892
ENSG00000101040	-1.2869637	3.52E-08
ENSG00000101096	-1.5888977	4.87E-10
ENSG00000101132	1.36155858	0.01527478
ENSG00000101188	-1.2522666	0.00057138
ENSG00000101190	-1.2514328	0.00098097
ENSG00000101224	-1.3999611	4.29E-08
ENSG00000101236	1.25536589	9.23E-06

Gene ID	FC 1HG - 4HG	adj p-value 1HG - 4HG
ENSG00000101255	-1.6281171	3.20E-12
ENSG00000101265	-1.6676855	3.83E-11
ENSG00000101331	-1.3125956	8.00E-05
ENSG00000101412	-1.3853938	0.00072623
ENSG00000101445	-2.1864556	2.17E-15
ENSG00000101447	-1.2247245	0.01566858
ENSG00000101544	-1.2131197	4.67E-05
ENSG00000101577	-1.3841465	2.68E-11
ENSG00000101665	-3.7508419	4.11E-13
ENSG00000101670	2.2638682	1.22E-17
ENSG00000101695	-1.2052384	0.04886809
ENSG00000101871	-1.7733923	7.67E-16
ENSG00000102010	-1.52151	9.09E-10
ENSG00000102096	-1.282835	0.00931822
ENSG00000102098	-1.253036	0.00997555
ENSG00000102312	1.29077942	0.00078183
ENSG00000102384	-1.2442224	0.01695742
ENSG00000102401	1.22511838	0.0001568
ENSG00000102531	1.53643184	9.67E-16
ENSG00000102554	1.35175553	3.84E-05
ENSG00000102606	-1.2057201	1.56E-08
ENSG00000102699	-1.238284	8.68E-10
ENSG00000102755	2.03445735	6.95E-18
ENSG00000102890	-1.7527217	1.27E-05
ENSG00000102934	-1.4449285	0.00013277
ENSG00000102935	2.06446899	8.49E-09
ENSG00000103111	-1.3304386	1.82E-11
ENSG00000103199	1.23711659	0.02866745
ENSG00000103241	-1.3162577	0.02454158
ENSG00000103257	-1.604112	1.19E-12
ENSG00000103319	-1.2400001	6.95E-09
ENSG00000103528	1.21074903	0.02648292

Gene ID	FC 1HG - 4HG	adj p-value 1HG - 4HG
ENSG00000103707	1.24057279	0.00052217
ENSG00000103888	1.29672731	0.00016503
ENSG00000103994	1.20649111	6.49E-06
ENSG00000104081	-2.4568141	4.29E-07
ENSG00000104131	1.25830775	0.02405603
ENSG00000104312	1.3469664	3.17E-06
ENSG00000104388	1.20344842	0.00256806
ENSG00000104643	1.20535074	0.00652928
ENSG00000104689	-1.3484234	9.32E-10
ENSG00000104695	1.20105875	5.16E-05
ENSG00000104856	1.29518578	0.00745127
ENSG00000105011	-1.2459394	0.00551824
ENSG00000105072	1.22529762	0.01898323
ENSG00000105088	2.56867469	6.14E-17
ENSG00000105287	-1.5377684	4.29E-12
ENSG00000105321	-1.2345295	0.00198505
ENSG00000105329	1.20446251	0.00012934
ENSG00000105339	-1.2974633	1.93E-07
ENSG00000105443	-1.2597988	0.00066888
ENSG00000105656	-1.3415722	5.16E-07
ENSG00000105662	-1.4467563	1.41E-06
ENSG00000105722	-1.2192075	0.0019012
ENSG00000105738	-1.2478253	2.68E-06
ENSG00000105793	1.27768778	0.01358463
ENSG00000105810	1.73458795	2.00E-10
ENSG00000105825	1.46796963	2.69E-13
ENSG00000105849	1.27650993	0.00482757
ENSG00000105851	-1.4517521	1.94E-08
ENSG00000105855	2.25656763	1.37E-13
ENSG00000105856	-1.2148251	0.00353734
ENSG00000105976	-1.2056888	6.05E-06
ENSG00000105991	1.34318586	0.00063493

Gene ID	FC 1HG - 4HG	adj p-value 1HG - 4HG
ENSG00000106003	-2.2196894	3.10E-09
ENSG00000106069	-1.4220485	2.9556E-05
ENSG00000106070	2.37336358	2.58E-22
ENSG00000106089	-1.62503	1.35E-06
ENSG00000106100	-1.454899	3.59E-09
ENSG00000106351	-1.7110863	7.16E-11
ENSG00000106537	1.42782257	9.17E-08
ENSG00000106546	1.37459872	1.94E-09
ENSG00000106571	-1.5288632	5.62E-12
ENSG00000106688	-1.2744067	4.49E-08
ENSG00000106723	1.2064235	0.03608557
ENSG00000106829	-1.2967743	1.19E-07
ENSG00000106852	-1.699158	3.33E-08
ENSG00000106948	-1.364829	1.07E-05
ENSG00000106991	1.20570401	0.00038525
ENSG00000106993	1.20040648	0.01733598
ENSG00000107077	-1.2886509	0.00171815
ENSG00000107099	1.30462067	0.00552885
ENSG00000107104	1.20112803	3.20E-06
ENSG00000107130	1.21034575	4.57E-07
ENSG00000107185	-1.2658324	2.17E-06
ENSG00000107282	1.26493768	0.03046905
ENSG00000107485	-1.5333465	9.19E-09
ENSG00000107551	-1.4869064	0.00026383
ENSG00000107562	3.90594034	2.54E-21
ENSG00000107611	-1.2066133	0.0077897
ENSG00000107672	-1.2069244	4.46E-05
ENSG00000107679	1.25219327	3.54E-06
ENSG00000107719	-1.6441172	2.59E-08
ENSG00000107731	2.48166456	3.25E-12
ENSG00000107771	1.36378738	7.01E-06
ENSG00000107779	1.20323721	0.00344543

Gene ID	FC 1HG - 4HG	adj p-value 1HG - 4HG
ENSG00000107789	1.29918137	0.00016879
ENSG00000107863	1.57581365	2.75E-17
ENSG00000107872	-1.2065047	0.02968264
ENSG00000107949	1.21444245	6.47E-05
ENSG00000107957	-1.2240597	1.98E-08
ENSG00000107984	2.17283965	6.00E-14
ENSG00000108219	-1.3591913	1.37E-12
ENSG00000108262	-1.4116707	3.38E-11
ENSG00000108306	-1.2262864	0.01139882
ENSG00000108312	1.3106281	2.93E-08
ENSG00000108342	-1.3666749	0.01296467
ENSG00000108433	1.24711068	5.82E-05
ENSG00000108551	-1.597663	2.08E-08
ENSG00000108556	1.23531474	0.03831631
ENSG00000108557	-1.2037098	4.30E-05
ENSG00000108622	-1.2311036	7.04E-08
ENSG00000108733	1.26585688	0.00575351
ENSG00000108932	-1.22867	0.00277016
ENSG00000108960	1.69048004	1.83E-07
ENSG00000108984	-1.2768792	0.03349597
ENSG00000109046	-1.3214863	5.41E-05
ENSG00000109133	1.21690477	0.02212584
ENSG00000109189	1.25145987	0.00151003
ENSG00000109320	1.33445872	9.97E-14
ENSG00000109321	1.33731757	8.65E-06
ENSG00000109458	-1.5641055	2.09E-07
ENSG00000109501	-1.443198	2.10E-07
ENSG00000109674	-1.2704405	0.02683852
ENSG00000109685	-1.2155394	9.54E-07
ENSG00000109689	1.28318688	1.36E-05
ENSG00000109787	-1.6159468	1.65E-15
ENSG00000109971	1.21313969	1.60E-09

Gene ID	FC 1HG - 4HG	adj p-value 1HG - 4HG
ENSG00000110047	-1.2910762	4.47E-07
ENSG00000110080	-1.3227814	2.78E-07
ENSG00000110172	1.21854779	0.00043297
ENSG00000110330	1.33504144	0.000505
ENSG00000110675	1.3096871	0.0069545
ENSG00000110852	1.42315301	0.00321091
ENSG00000110931	-1.3120341	9.47E-12
ENSG00000111012	-1.2410748	0.02735957
ENSG00000111057	-1.2502283	7.51E-05
ENSG00000111077	-1.4498046	1.60E-09
ENSG00000111110	-1.7247251	0.00013887
ENSG00000111145	-1.321481	3.18E-11
ENSG00000111247	-1.2697055	0.00326308
ENSG00000111331	-1.2063672	0.00184818
ENSG00000111335	-1.3474285	0.01056485
ENSG00000111339	1.61611667	5.13E-09
ENSG00000111341	1.20471416	0.00271667
ENSG00000111490	1.39501842	6.03E-06
ENSG00000111647	1.4621024	0.00033498
ENSG00000111665	-1.2570662	0.04020515
ENSG00000111671	1.27628361	0.0126106
ENSG00000111674	-1.283067	0.01248545
ENSG00000111725	-1.4309776	3.88E-07
ENSG00000111727	1.24030838	0.00597177
ENSG00000111817	1.34697578	3.63E-07
ENSG00000111846	-1.541788	8.96E-11
ENSG00000111875	1.22167557	0.01708585
ENSG00000111907	1.22929585	0.04478375
ENSG00000111961	-1.8336735	3.51E-25
ENSG00000112033	1.3985005	5.11E-10
ENSG00000112137	-1.4768484	1.50E-08
ENSG00000112208	1.2845005	0.00478138

Gene ID	FC 1HG - 4HG	adj p-value 1HG - 4HG
ENSG00000112210	1.51136907	3.97E-09
ENSG00000112245	1.28108332	3.79E-05
ENSG00000112297	-1.7377424	1.08E-08
ENSG00000112305	1.39175861	1.31E-08
ENSG00000112379	1.20800983	0.00058454
ENSG00000112541	1.70161513	5.33E-07
ENSG00000112559	1.4364811	6.10E-06
ENSG00000112701	1.23936899	0.01743956
ENSG00000112715	-1.9994684	8.21E-10
ENSG00000112773	1.87542922	7.64E-05
ENSG00000112837	1.36379895	5.05E-06
ENSG00000112851	1.25488813	0.00631962
ENSG00000112855	1.26029468	1.94E-06
ENSG00000112984	-1.2336159	0.00149949
ENSG00000113070	-1.8751939	2.58E-10
ENSG00000113368	-1.2575667	4.50E-06
ENSG00000113369	2.34144098	1.17E-15
ENSG00000113448	-1.2433617	0.0065947
ENSG00000113532	1.83006146	3.33E-07
ENSG00000113555	-1.81305	1.66E-07
ENSG00000113575	1.21669358	0.00439147
ENSG00000113580	1.88364739	8.48E-14
ENSG00000113594	1.27622602	0.00105743
ENSG00000113595	1.29382335	0.01258274
ENSG00000113749	-1.3389493	0.02559951
ENSG00000113966	1.30212033	0.03695427
ENSG00000114019	-1.6556422	7.39E-13
ENSG00000114166	-1.3424825	0.00036394
ENSG00000114302	1.24554749	3.14E-08
ENSG00000114315	-14.965404	3.12E-22
ENSG00000114346	-1.2168275	0.01537312
ENSG00000114378	-1.2668299	0.01049469

Gene ID	FC 1HG - 4HG	adj p-value 1HG - 4HG
ENSG00000114423	1.50831564	5.89E-08
ENSG00000114480	1.2386226	1.91E-06
ENSG00000114626	-1.2818149	0.04227905
ENSG00000114698	-1.3273045	0.00021343
ENSG00000114739	1.27999196	0.00617356
ENSG00000114767	1.22411964	0.00222911
ENSG00000114812	-1.6682434	0.00012163
ENSG00000114841	-1.4235487	0.00066502
ENSG00000114999	1.24465646	3.89E-12
ENSG00000115084	1.22811849	0.00766832
ENSG00000115163	-1.248535	0.01591066
ENSG00000115170	1.24436871	5.64E-08
ENSG00000115183	-1.2903847	2.63E-08
ENSG00000115233	1.22120272	1.60E-05
ENSG00000115290	1.27862888	0.00210669
ENSG00000115368	1.26988795	7.06E-05
ENSG00000115414	1.20396536	0.00034939
ENSG00000115525	-1.5109278	7.63E-05
ENSG00000115602	2.3807764	3.05E-08
ENSG00000115604	1.39855577	0.00023735
ENSG00000115641	-1.5820809	8.49E-14
ENSG00000115687	-1.3375416	0.00028062
ENSG00000115738	-2.8702782	6.30E-10
ENSG00000115844	-2.5447484	9.30E-14
ENSG00000115896	1.5066957	0.00048498
ENSG00000115902	-1.2473841	9.1403E-05
ENSG00000115946	1.29456123	6.17E-07
ENSG00000116017	-1.6491465	8.56E-07
ENSG00000116106	-1.2356844	3.60E-05
ENSG00000116273	-1.4444085	4.56E-09
ENSG00000116285	-1.8439675	2.15E-12
ENSG00000116514	-1.4034136	2.30E-09

Gene ID	FC 1HG - 4HG	adj p-value 1HG - 4HG
ENSG00000116584	-1.3079935	9.06E-09
ENSG00000116604	-1.3966486	1.53E-07
ENSG00000116641	1.44438649	5.75E-07
ENSG00000116704	1.23396078	0.0010732
ENSG00000116711	2.30071458	5.50E-10
ENSG00000116731	-1.231417	2.66E-06
ENSG00000116741	1.42014968	7.79E-05
ENSG00000116815	1.34327336	0.00932512
ENSG00000116991	-1.3668446	2.65E-06
ENSG00000117114	1.24469225	9.45E-07
ENSG00000117133	1.21946185	0.00404625
ENSG00000117143	1.32667454	3.16E-08
ENSG00000117152	1.57157694	2.64E-09
ENSG00000117174	1.31028253	0.00002593
ENSG00000117226	-1.2490878	4.46E-07
ENSG00000117318	-2.0113723	3.14E-15
ENSG00000117399	-1.2753132	0.00155847
ENSG00000117525	-1.7648904	1.28E-06
ENSG00000117595	2.9574584	1.68E-11
ENSG00000117597	1.23179252	6.96E-06
ENSG00000117650	-1.3377853	0.00035035
ENSG00000117724	-1.2834544	0.00128847
ENSG00000118193	-1.4093084	6.17E-05
ENSG00000118402	1.45282189	0.00042021
ENSG00000118407	-1.3047953	0.00929589
ENSG00000118473	-1.397392	0.00651715
ENSG00000118515	-1.3830095	1.20E-12
ENSG00000118689	-1.2870198	5.94E-06
ENSG00000118707	1.2005233	0.00062695
ENSG00000118922	-1.4437809	5.60E-06
ENSG00000118946	3.66072646	1.06E-09
ENSG00000118971	-1.3059677	0.02097265

Gene ID	FC 1HG - 4HG	adj p-value 1HG - 4HG
ENSG00000118985	1.51474986	1.84E-11
ENSG00000119138	1.27740537	2.9791E-05
ENSG00000119139	1.28941825	8.46E-08
ENSG00000119397	-1.2648905	0.00010303
ENSG00000119508	-3.0750201	1.82E-10
ENSG00000119522	-1.3321361	2.60E-07
ENSG00000119599	1.3274694	8.49E-06
ENSG00000119630	-1.6318655	3.84E-10
ENSG00000119661	1.21161071	0.01807704
ENSG00000119669	-2.1206157	2.54E-15
ENSG00000119673	-1.2454829	0.03362368
ENSG00000119771	1.31226023	3.56E-08
ENSG00000119862	-1.2323182	0.01493023
ENSG00000119900	-1.2575671	0.00674189
ENSG00000119938	2.11997831	3.55E-11
ENSG00000119950	-1.2626224	0.03754095
ENSG00000119965	1.22208675	0.01367369
ENSG00000120129	-2.6883522	1.78E-17
ENSG00000120278	-2.6186492	5.38E-23
ENSG00000120539	-1.2595407	0.00308903
ENSG00000120549	-1.3718646	0.00097364
ENSG00000120693	3.18548797	2.58E-17
ENSG00000120694	1.23618445	9.61E-06
ENSG00000120696	1.50875603	0.00031683
ENSG00000120738	-9.5491792	4.72E-16
ENSG00000120784	1.41297387	0.00010436
ENSG00000120800	1.22905148	0.00026499
ENSG00000120875	-1.6074674	3.49E-15
ENSG00000121060	-1.2643404	3.25E-08
ENSG00000121152	-1.3232863	0.00150744
ENSG00000121390	1.27504741	1.82E-09
ENSG00000121406	1.42029321	1.60E-05

Gene ID	FC 1HG - 4HG	adj p-value 1HG - 4HG
ENSG00000121417	1.27009954	0.00551223
ENSG00000121481	1.21735094	0.00020559
ENSG00000121797	-1.4763019	5.00E-07
ENSG00000121858	-2.0192827	8.64E-08
ENSG00000121903	1.29601227	0.01571318
ENSG00000121957	-1.5684749	7.88E-09
ENSG00000121964	-1.2769019	0.00060757
ENSG00000121966	-1.8645177	3.79E-12
ENSG00000121988	-1.2257793	0.02292555
ENSG00000122068	1.27602483	4.82E-07
ENSG00000122257	-1.3313288	3.46E-10
ENSG00000122378	-1.2226658	2.91E-07
ENSG00000122420	1.22208448	0.04514954
ENSG00000122435	1.20646212	0.04486538
ENSG00000122641	1.90039926	1.28E-10
ENSG00000122786	1.26265769	3.50E-09
ENSG00000122861	-2.2150774	1.34E-14
ENSG00000122870	1.6940852	1.55E-08
ENSG00000122877	-4.5460994	7.21E-14
ENSG00000122966	-1.2666879	0.00060847
ENSG00000123066	1.31396511	1.02E-09
ENSG00000123080	-1.3197716	0.00011378
ENSG00000123094	1.21626951	0.00277607
ENSG00000123146	-1.2496093	0.00625263
ENSG00000123178	1.24249313	0.00017587
ENSG00000123358	-5.9884933	3.56E-16
ENSG00000123473	-1.2437661	0.0028711
ENSG00000123485	-1.4463247	0.00073627
ENSG00000123545	1.25257556	0.03095146
ENSG00000123700	1.99620854	3.82E-06
ENSG00000124216	-5.135789	1.95E-23
ENSG00000124225	1.5842393	5.80E-06

Gene ID	FC 1HG - 4HG	adj p-value 1HG - 4HG
ENSG00000124459	1.24625454	0.00030069
ENSG00000124496	-1.3044563	0.00556627
ENSG00000124762	-1.2707785	7.37E-11
ENSG00000124772	1.46922728	0.00010018
ENSG00000124875	1.34357375	0.01647773
ENSG00000125089	-1.4733921	1.15E-08
ENSG00000125257	1.23352364	0.00016939
ENSG00000125266	1.4659415	3.96E-06
ENSG00000125347	-1.4985929	0.00010607
ENSG00000125378	1.90964236	2.10E-10
ENSG00000125457	-1.2358154	0.01411364
ENSG00000125459	1.22796221	0.00515334
ENSG00000125740	-5.0466125	9.68E-10
ENSG00000125814	1.29104138	0.01128841
ENSG00000125845	1.48276956	9.42E-06
ENSG00000125871	-1.244116	7.31E-05
ENSG00000125945	1.39635976	2.53E-07
ENSG00000125966	1.43770252	0.00052635
ENSG00000125968	-2.532375	6.08E-11
ENSG00000126070	1.20389141	0.02377624
ENSG00000126351	-1.398197	5.51E-09
ENSG00000126603	-1.4394643	3.42E-06
ENSG00000126705	-1.6145635	1.25E-08
ENSG00000126790	1.2809004	1.96E-05
ENSG00000126878	-1.2429966	0.00132448
ENSG00000126882	-1.8327198	1.61E-07
ENSG00000127124	1.22985452	0.02690632
ENSG00000127314	1.29239591	0.00751567
ENSG00000127328	-1.2481209	1.65E-05
ENSG00000127418	1.2303415	0.00135658
ENSG00000127419	-1.2023175	0.02429357
ENSG00000127511	-1.2340904	2.58E-07

Gene ID	FC 1HG - 4HG	adj p-value 1HG - 4HG
ENSG00000127533	-3.1141308	6.14E-17
ENSG00000127564	-1.2479743	0.03673232
ENSG00000127666	-1.263935	8.29E-06
ENSG00000127824	-1.3736881	7.27E-05
ENSG00000128016	-2.1387014	1.56E-09
ENSG00000128052	-1.2049071	4.80E-06
ENSG00000128059	1.21655068	0.01371207
ENSG00000128272	-1.2128552	3.19E-07
ENSG00000128284	-1.6058061	4.08E-08
ENSG00000128294	-1.2236471	1.64E-08
ENSG00000128342	-1.4319235	0.00050176
ENSG00000128567	1.45339336	3.38E-11
ENSG00000128606	1.20372213	0.00498252
ENSG00000128641	-1.2687581	0.000761
ENSG00000128805	-1.533894	1.93E-13
ENSG00000128815	-1.562087	4.87E-07
ENSG00000128833	-1.2900997	0.0049126
ENSG00000128849	-1.3497184	2.89E-07
ENSG00000128881	-1.3498685	1.88E-05
ENSG00000128917	-5.1624444	3.77E-20
ENSG00000128923	-1.3398453	1.49E-05
ENSG00000129116	1.25365207	0.00417318
ENSG00000129173	-1.3882005	6.77E-05
ENSG00000129195	-1.2022274	0.03592893
ENSG00000129422	-1.5024965	1.45E-11
ENSG00000129474	-1.3044784	2.16E-06
ENSG00000129514	-1.2140146	0.01142126
ENSG00000129667	-1.2632467	3.23E-05
ENSG00000130066	1.63257479	4.76E-08
ENSG00000130201	1.96057928	1.92E-10
ENSG00000130222	-1.5282364	1.75E-05
ENSG00000130300	1.51719761	0.00239433

Gene ID	FC 1HG - 4HG	adj p-value 1HG - 4HG
ENSG00000130338	1.29649095	2.42E-06
ENSG00000130449	-1.5294062	6.90E-20
ENSG00000130479	-1.3647732	6.88E-10
ENSG00000130513	-1.7326124	3.14E-15
ENSG00000130517	-1.3620687	2.97E-06
ENSG00000130590	-1.3869757	0.00042141
ENSG00000130684	1.21695268	0.00454037
ENSG00000130702	-1.4461497	8.59E-09
ENSG00000130751	1.2984768	3.53E-05
ENSG00000130772	1.27835805	0.00768931
ENSG00000130956	1.39294506	2.02E-07
ENSG00000131127	1.34959453	0.00564379
ENSG00000131149	1.81963811	9.21E-12
ENSG00000131153	-1.2065666	0.03392925
ENSG00000131370	-1.854744	1.96E-18
ENSG00000131378	1.38394492	8.80E-11
ENSG00000131386	-1.38422	0.01103255
ENSG00000131409	3.28469519	3.01E-09
ENSG00000131459	1.27737936	0.00456487
ENSG00000131634	1.24872535	0.00077461
ENSG00000131669	-1.2603855	2.23E-05
ENSG00000131724	1.37087299	1.85E-06
ENSG00000131725	1.30339085	2.68E-07
ENSG00000131759	-1.8942815	4.01E-11
ENSG00000131791	1.24735281	0.00172169
ENSG00000131831	1.76551731	7.10E-09
ENSG00000131979	1.31722533	0.0117564
ENSG00000132003	-1.4663953	2.45E-07
ENSG00000132205	-1.2301764	0.03360393
ENSG00000132294	1.37317673	1.07E-06
ENSG00000132334	-1.3325597	1.20E-08
ENSG00000132388	1.22303473	0.00581011

Gene ID	FC 1HG - 4HG	adj p-value 1HG - 4HG
ENSG00000132405	-1.2009871	4.61E-06
ENSG00000132436	-1.3124478	0.00188084
ENSG00000132470	-1.3700038	0.0004355
ENSG00000132481	-1.2961497	0.00113252
ENSG00000132510	-2.5530123	7.99E-19
ENSG00000132570	-1.2892305	0.00544077
ENSG00000132589	-1.2132176	3.82E-05
ENSG00000132613	-1.3472855	7.42E-07
ENSG00000132622	-1.4099111	1.87E-05
ENSG00000132640	1.28054718	5.28E-07
ENSG00000132669	-1.2583668	2.07E-05
ENSG00000132694	-1.2798289	1.06E-07
ENSG00000132819	-1.2148263	0.00023619
ENSG00000132825	1.30213286	0.00021689
ENSG00000132906	-1.2721676	0.00022221
ENSG00000133056	-1.7147301	2.91E-10
ENSG00000133110	1.23224678	0.00076494
ENSG00000133401	-1.4678111	0.00040436
ENSG00000133612	-1.3514872	7.56E-09
ENSG00000133657	2.19631328	4.79E-11
ENSG00000133789	1.26017927	2.66E-07
ENSG00000133794	1.3360916	3.15E-05
ENSG00000133805	-1.5411745	1.08E-05
ENSG00000133818	1.27901071	1.23E-08
ENSG00000133858	1.28468764	0.00286193
ENSG00000133985	-1.2451187	0.01017739
ENSG00000134070	-1.3113115	6.30E-05
ENSG00000134107	-3.9124712	1.07E-26
ENSG00000134138	-1.3662837	1.24E-05
ENSG00000134222	-1.3157282	0.00622746
ENSG00000134245	-1.2164161	0.04201011
ENSG00000134278	1.27366455	8.54E-10

Gene ID	FC 1HG - 4HG	adj p-value 1HG - 4HG
ENSG00000134294	1.66661382	1.56E-05
ENSG00000134323	1.44756179	0.00033081
ENSG00000134324	-1.2245754	3.27E-06
ENSG00000134352	1.37999336	8.35E-05
ENSG00000134363	1.94540344	1.32E-11
ENSG00000134375	1.22329629	0.00117025
ENSG00000134461	1.26319248	0.00307412
ENSG00000134508	-1.3718204	5.38E-05
ENSG00000134574	-1.2328314	4.60E-06
ENSG00000134690	-1.3006008	0.0030699
ENSG00000134769	1.22733949	0.00227793
ENSG00000134780	-1.2872537	0.00103154
ENSG00000134802	-1.3119102	1.50E-06
ENSG00000134852	1.21794663	0.01397648
ENSG00000135074	-1.388194	2.12E-06
ENSG00000135083	-1.3169655	0.00018047
ENSG00000135269	1.21827801	0.0001091
ENSG00000135338	1.3356423	0.01082754
ENSG00000135363	-1.2650922	0.00279094
ENSG00000135365	-1.3710791	4.01E-08
ENSG00000135476	-1.3249676	0.01223074
ENSG00000135482	1.22985658	0.00542333
ENSG00000135547	1.47817082	0.00608708
ENSG00000135604	-1.5099001	4.80E-09
ENSG00000135617	1.25198609	0.00065401
ENSG00000135625	-1.471323	2.00E-06
ENSG00000135842	-1.213092	0.00151417
ENSG00000135919	1.232909	1.57E-05
ENSG00000135966	-1.9269354	2.53E-17
ENSG00000136021	1.25917832	0.00772749
ENSG00000136044	1.27558098	1.71E-05
ENSG00000136048	1.21504226	0.00022087

Gene ID	FC 1HG - 4HG	adj p-value 1HG - 4HG
ENSG00000136052	1.31645672	0.02448454
ENSG00000136114	-1.6203083	2.14E-11
ENSG00000136153	-1.2429708	4.37E-05
ENSG00000136158	-1.5911223	1.22E-08
ENSG00000136237	-1.9219363	8.92E-14
ENSG00000136244	2.42105083	1.82E-10
ENSG00000136383	1.43004024	1.23E-07
ENSG00000136404	1.35043655	1.14E-05
ENSG00000136449	-1.3287769	0.03114406
ENSG00000136451	-1.2283817	7.12E-10
ENSG00000136490	1.35067092	1.42E-06
ENSG00000136560	-1.2855238	0.00013787
ENSG00000136603	-1.4339186	1.40E-05
ENSG00000136630	1.54402594	0.00074817
ENSG00000136717	-1.3814918	2.80E-07
ENSG00000136770	1.2124555	3.66E-05
ENSG00000136826	-3.3182003	8.78E-18
ENSG00000136859	-1.252656	1.42E-05
ENSG00000136861	-1.2395212	3.61E-05
ENSG00000136869	1.46912184	2.66E-09
ENSG00000136870	1.250404	0.00023034
ENSG00000136935	-1.2065801	2.04E-05
ENSG00000136940	-1.2218335	4.79E-06
ENSG00000136997	-1.2938202	1.72E-05
ENSG00000137177	1.50843809	8.25E-15
ENSG00000137193	-1.813256	2.49E-06
ENSG00000137203	1.63814166	1.26E-08
ENSG00000137218	1.25357127	0.01313847
ENSG00000137266	-1.29111	0.00060068
ENSG00000137393	1.36512762	6.16E-06
ENSG00000137414	1.29961179	0.00087318
ENSG00000137486	-1.542809	2.00E-11

Gene ID	FC 1HG - 4HG	adj p-value 1HG - 4HG
ENSG00000137494	1.36993729	4.99E-07
ENSG00000137502	1.56161105	7.75E-07
ENSG00000137522	1.29772802	8.33E-06
ENSG00000137656	-1.2039426	0.00010511
ENSG00000137692	1.29766885	6.23E-08
ENSG00000137693	1.48780509	6.47E-07
ENSG00000137727	1.75069493	1.86E-09
ENSG00000137760	1.35535152	0.00001412
ENSG00000137764	-1.3642979	7.46E-07
ENSG00000137801	1.25495319	7.73E-07
ENSG00000137802	-1.371742	7.88E-09
ENSG00000137804	-1.2356702	0.00715535
ENSG00000137807	-1.4046105	4.86E-06
ENSG00000137825	-1.2195451	0.02902679
ENSG00000137831	1.53543332	1.40E-07
ENSG00000137834	1.9033528	5.63E-05
ENSG00000137872	3.15089063	1.01E-16
ENSG00000137878	-1.509676	5.25E-05
ENSG00000137992	1.23850878	0.00201363
ENSG00000138018	1.31316627	0.00540884
ENSG00000138101	-1.2334592	0.00952261
ENSG00000138134	-1.6203042	1.51E-07
ENSG00000138135	1.54352011	3.18E-12
ENSG00000138162	-1.648844	2.54E-09
ENSG00000138166	-2.447339	1.08E-16
ENSG00000138180	-1.2116488	0.03250735
ENSG00000138347	-1.5765746	6.93E-05
ENSG00000138376	-1.3152347	0.00054847
ENSG00000138386	-1.3100381	5.31E-06
ENSG00000138411	1.39891467	4.76E-09
ENSG00000138448	1.44411945	9.32E-07
ENSG00000138449	1.78222827	8.15E-12

Gene ID	FC 1HG - 4HG	adj p-value 1HG - 4HG
ENSG00000138496	-1.2591453	0.01000626
ENSG00000138639	1.26924961	1.46E-08
ENSG00000138641	1.21766073	0.00116522
ENSG00000138650	1.47624099	2.08E-05
ENSG00000138675	1.37066727	0.0014114
ENSG00000138678	-1.33049	5.91E-05
ENSG00000138835	-1.7957654	1.08E-10
ENSG00000139117	-1.2212991	0.0027483
ENSG00000139154	1.24973015	5.82E-05
ENSG00000139163	1.3129045	0.00052216
ENSG00000139218	1.26154557	0.00025377
ENSG00000139263	1.32611495	5.84E-07
ENSG00000139266	-1.4086322	0.01555982
ENSG00000139318	-1.2622898	5.72E-06
ENSG00000139354	-1.5201685	3.87E-07
ENSG00000139675	1.21786272	0.01991255
ENSG00000139687	1.34682185	0.00017651
ENSG00000139722	-1.2302519	3.60E-07
ENSG00000139874	-1.4427104	0.00125031
ENSG00000139926	-1.4545719	3.42E-11
ENSG00000140006	1.20719116	0.04050285
ENSG00000140280	1.24001241	0.03946005
ENSG00000140416	1.27316695	4.39E-09
ENSG00000140450	1.46308044	7.34E-05
ENSG00000140451	-1.3466766	0.01314504
ENSG00000140534	-1.2352637	0.03657476
ENSG00000140564	1.24313608	1.27E-05
ENSG00000140691	-1.5684633	7.15E-10
ENSG00000140853	-1.4111537	3.46E-05
ENSG00000140873	-1.4593563	4.33E-06
ENSG00000141040	1.26450532	0.00302683
ENSG00000141068	-1.6154593	5.88E-12

Gene ID	FC 1HG - 4HG	adj p-value 1HG - 4HG
ENSG00000141298	-1.2132727	4.35E-05
ENSG00000141401	-1.3630347	0.00510631
ENSG00000141441	1.34920137	0.00086305
ENSG00000141480	-1.2888302	0.00140262
ENSG00000141655	-1.4476098	4.24E-06
ENSG00000141664	-1.336554	1.29E-07
ENSG00000141668	2.0375169	6.12E-06
ENSG00000141858	1.27261135	1.00E-05
ENSG00000142178	-1.8528779	2.37E-08
ENSG00000142197	-1.2012205	0.00053152
ENSG00000142528	1.2503746	0.00364825
ENSG00000142627	-1.7881658	1.61E-16
ENSG00000142661	1.51810876	9.93E-05
ENSG00000142871	-1.2300423	0.0001375
ENSG00000142945	-1.3254171	0.00207673
ENSG00000143067	-1.6147484	7.33E-08
ENSG00000143153	1.4265657	1.32E-08
ENSG00000143341	1.53470073	6.2594E-05
ENSG00000143344	-1.7686164	1.11E-15
ENSG00000143367	-1.5766541	1.14E-07
ENSG00000143369	-1.2294171	0.0312898
ENSG00000143375	-1.2563275	0.02620923
ENSG00000143376	1.21336045	1.17E-07
ENSG00000143384	-1.4011643	1.33E-11
ENSG00000143409	-1.2928242	0.00178584
ENSG00000143443	1.22811135	0.02615688
ENSG00000143458	-1.2360749	0.00322058
ENSG00000143476	-1.3548899	0.00190935
ENSG00000143578	-1.2668347	0.01347362
ENSG00000143643	-1.2770687	0.00022064
ENSG00000143669	1.23361124	0.03073376
ENSG00000143751	-1.2021275	0.00048552

Gene ID	FC 1HG - 4HG	adj p-value 1HG - 4HG
ENSG00000143772	-2.6846138	8.12E-21
ENSG00000143816	-2.8608501	1.04E-11
ENSG00000143851	1.4128584	0.00772147
ENSG00000143995	1.31438033	5.74E-06
ENSG00000144026	1.24752333	0.00505532
ENSG00000144040	-1.2010805	0.00580948
ENSG00000144063	1.73163473	2.17E-13
ENSG00000144120	1.25963232	0.002658
ENSG00000144161	1.22245078	0.01899318
ENSG00000144320	1.27240581	0.00035468
ENSG00000144354	-1.233865	0.00385525
ENSG00000144366	1.97392569	2.05E-12
ENSG00000144560	-1.541706	4.94E-14
ENSG00000144644	1.53214604	0.000272
ENSG00000144645	-1.322175	1.98E-09
ENSG00000144655	-4.5938031	1.37E-27
ENSG00000144677	-1.4647557	5.40E-11
ENSG00000144712	-1.2357271	0.00289558
ENSG00000144749	-1.9372567	4.76E-15
ENSG00000144824	-1.593566	1.73E-10
ENSG00000144893	1.40222202	3.85E-07
ENSG00000145014	-1.4888721	1.20E-10
ENSG00000145016	-1.2505649	6.12E-07
ENSG00000145365	1.27231438	0.00292015
ENSG00000145386	-1.2193372	0.00707097
ENSG00000145414	1.21901617	0.00057296
ENSG00000145555	1.23551302	1.70E-06
ENSG00000145632	1.7853615	1.57E-12
ENSG00000145723	1.22705923	0.0494515
ENSG00000145740	1.2171113	0.0105757
ENSG00000145817	1.57665415	2.34E-09
ENSG00000145860	1.29358195	2.09E-08

Gene ID	FC 1HG - 4HG	adj p-value 1HG - 4HG
ENSG00000145911	-3.4926662	6.72E-20
ENSG00000145916	-1.5210277	6.67E-09
ENSG00000146021	-1.7859782	1.44E-10
ENSG00000146094	-1.5507834	0.00011519
ENSG00000146143	-1.2531988	0.00119789
ENSG00000146232	-1.2491567	0.00041904
ENSG00000146374	1.86494811	1.36E-06
ENSG00000146414	1.26876221	0.00076888
ENSG00000146426	-1.2330653	0.00052216
ENSG00000146476	1.2573279	0.03299031
ENSG00000146587	1.35444229	0.00103388
ENSG00000146670	-1.2895175	0.00645518
ENSG00000146776	-1.28287	0.00035271
ENSG00000146830	-1.228243	0.00502411
ENSG00000146834	-1.3338086	5.15E-08
ENSG00000146859	1.27157251	0.0195072
ENSG00000146950	-1.4663128	5.49E-10
ENSG00000147119	-1.4209717	2.10E-07
ENSG00000147130	-1.2355559	0.00012522
ENSG00000147180	1.31915144	0.00329238
ENSG00000147408	-1.2432842	0.0227917
ENSG00000147454	-1.3373174	5.05E-06
ENSG00000147459	-1.2096918	1.05E-05
ENSG00000147536	-1.2009937	0.02729157
ENSG00000147650	1.3444557	1.14E-05
ENSG00000147789	1.21900077	0.00274693
ENSG00000147852	-1.3396235	0.00501252
ENSG00000147862	-1.2004124	0.00056306
ENSG00000148219	-1.2617554	0.01690099
ENSG00000148339	-1.562315	3.37E-08
ENSG00000148400	-1.2262808	2.25E-05
ENSG00000148429	-1.3060125	5.45E-09

Gene ID	FC 1HG - 4HG	adj p-value 1HG - 4HG
ENSG00000148468	1.35534982	1.02E-14
ENSG00000148660	-1.2449405	2.89E-06
ENSG00000148773	-1.3300823	0.0004757
ENSG00000148841	-1.6896776	3.40E-17
ENSG00000148848	1.57233217	0.00010187
ENSG00000148926	-1.6663166	4.20E-07
ENSG00000149150	-1.2427481	0.01789711
ENSG00000149177	1.29549786	1.83E-06
ENSG00000149289	-1.3859213	2.46E-06
ENSG00000149313	1.29959771	0.00091592
ENSG00000149503	-1.3527439	4.41E-05
ENSG00000149636	-1.2251932	0.00072629
ENSG00000149646	-1.4046629	0.00126002
ENSG00000149798	-1.3170529	7.26E-09
ENSG00000149948	1.52960318	1.56E-07
ENSG00000150347	1.81313538	9.30E-07
ENSG00000150457	-1.4807749	6.33E-13
ENSG00000150477	-1.3761793	0.00406618
ENSG00000150551	1.22190205	0.00851846
ENSG00000150630	2.02546641	9.72E-16
ENSG00000150764	1.62405876	1.72E-09
ENSG00000150787	1.24053319	0.00099921
ENSG00000150907	-1.3698758	2.56E-10
ENSG00000151012	1.82967278	4.83E-09
ENSG00000151023	1.42685282	0.00206325
ENSG00000151136	2.28912575	1.95E-10
ENSG00000151150	-1.2244729	0.00120888
ENSG00000151229	1.38443222	0.0014193
ENSG00000151247	1.39193781	9.31E-07
ENSG00000151414	1.50186013	8.92E-06
ENSG00000151458	1.27605884	0.00057145
ENSG00000151474	-1.2754089	4.04E-16

Gene ID	FC 1HG - 4HG	adj p-value 1HG - 4HG
ENSG00000151689	-1.3354615	1.24E-07
ENSG00000151692	-1.4400778	0.00090784
ENSG00000151702	-1.2023321	3.91E-07
ENSG00000151718	1.57940769	6.91E-19
ENSG00000151725	-1.2987233	0.00100491
ENSG00000151789	-1.2189774	0.04700091
ENSG00000151849	-1.3047643	0.00028438
ENSG00000151967	-1.9966894	4.06E-10
ENSG00000152133	1.24610223	8.10E-05
ENSG00000152229	1.20934132	0.03140321
ENSG00000152284	-1.5442378	2.69E-09
ENSG00000152409	-1.4220673	0.00076118
ENSG00000152413	1.29926084	0.00187341
ENSG00000152433	1.31498454	0.003071
ENSG00000152465	1.43328976	3.94E-10
ENSG00000152558	1.22493449	0.00555271
ENSG00000152689	1.86505791	5.89E-11
ENSG00000152782	1.24498999	0.03942266
ENSG00000152784	1.22533444	0.01853385
ENSG00000152804	1.20641676	0.00224819
ENSG00000152953	-1.2129274	0.00518387
ENSG00000153048	-1.2590621	0.00020515
ENSG00000153071	1.23135918	4.65E-09
ENSG00000153094	-2.3149883	1.14E-10
ENSG00000153162	1.26075043	1.35E-09
ENSG00000153179	-1.2437112	4.08E-06
ENSG00000153201	1.34593715	0.0001113
ENSG00000153208	-1.7522041	8.17E-11
ENSG00000153234	-1.7615725	0.03523152
ENSG00000153391	-1.2450108	0.00023723
ENSG00000153767	1.20199217	0.00205542
ENSG00000153814	1.65353108	3.80E-10

Gene ID	FC 1HG - 4HG	adj p-value 1HG - 4HG
ENSG00000153815	-1.2348486	1.42E-05
ENSG00000153896	1.24239208	0.01526706
ENSG00000154016	1.202049	0.01561768
ENSG00000154122	1.57506733	1.21E-11
ENSG00000154127	1.54797098	1.49E-13
ENSG00000154174	1.20149923	0.00441613
ENSG00000154217	1.3914929	0.00014856
ENSG00000154258	-1.2828825	0.00741629
ENSG00000154380	1.54297261	2.23E-17
ENSG00000154529	-1.2320074	1.26E-09
ENSG00000154639	-1.4687827	0.00082341
ENSG00000154734	2.93112934	4.55E-12
ENSG00000154760	-1.2558759	0.03097076
ENSG00000154803	-1.2713088	2.30E-05
ENSG00000154930	-1.2666212	0.01134234
ENSG00000154957	1.28532332	0.00021585
ENSG00000155011	1.70628944	5.73E-07
ENSG00000155090	-3.6601044	2.14E-19
ENSG00000155307	1.39344957	0.00093048
ENSG00000155438	1.20011915	0.00438892
ENSG00000155465	-1.2710239	0.00010274
ENSG00000155744	1.21819675	0.00987887
ENSG00000155760	-1.2789486	0.00010042
ENSG00000155868	1.24070286	0.003839
ENSG00000155966	1.28898553	0.00735166
ENSG00000155975	1.27026891	7.60E-06
ENSG00000156030	-1.8934842	1.49E-15
ENSG00000156049	1.35775814	0.00484275
ENSG00000156239	1.26999025	6.16E-05
ENSG00000156273	-1.4937863	2.39E-08
ENSG00000156381	-1.2361339	0.00019842
ENSG00000156463	-1.3609165	0.00401908

Gene ID	FC 1HG - 4HG	adj p-value 1HG - 4HG
ENSG00000156671	1.20247196	0.01293933
ENSG00000156675	-1.4936775	0.00017041
ENSG00000156928	1.22732581	3.48E-06
ENSG00000156970	-1.3811553	7.01E-05
ENSG00000156983	-1.3182856	1.74E-08
ENSG00000157111	-1.2952192	0.00024077
ENSG00000157168	1.36496191	1.09E-07
ENSG00000157214	1.55915272	1.09E-06
ENSG00000157224	1.35369337	0.00338391
ENSG00000157240	1.31998067	0.00416048
ENSG00000157404	-5.1223501	2.35E-17
ENSG00000157456	-1.2020294	0.0084823
ENSG00000157483	1.55344919	1.99E-15
ENSG00000157510	-1.4725804	1.81E-13
ENSG00000157514	1.22970205	0.01480092
ENSG00000157554	-1.2537503	1.69E-10
ENSG00000157557	-1.6048377	1.11E-17
ENSG00000157613	-1.5153287	0.00300426
ENSG00000157617	-1.8710635	4.07E-17
ENSG00000157764	1.20072699	0.00223338
ENSG00000157933	1.23238295	2.38E-08
ENSG00000157985	-1.2753522	0.00027619
ENSG00000158050	-1.337056	0.00280953
ENSG00000158106	-1.307184	0.04566385
ENSG00000158169	-1.2027499	0.00738375
ENSG00000158186	1.23665072	0.00795657
ENSG00000158195	-1.4173917	1.57E-10
ENSG00000158352	1.3484749	2.32E-09
ENSG00000158402	-1.4182901	2.02E-05
ENSG00000158458	-1.3750329	0.00020865
ENSG00000158555	-1.3867304	4.85E-10
ENSG00000158615	-1.2179105	6.16E-08

Gene ID	FC 1HG - 4HG	adj p-value 1HG - 4HG
ENSG00000158691	1.3136253	0.004746
ENSG00000158716	1.51314135	1.79E-07
ENSG00000158769	-1.2002734	0.00010416
ENSG00000158805	-1.238027	0.00191553
ENSG00000158859	-1.5260086	1.60E-08
ENSG00000158966	1.58508848	1.66E-10
ENSG00000159167	-2.1048767	2.60E-08
ENSG00000159231	-1.2167931	0.00120052
ENSG00000159259	-1.2261357	0.01118329
ENSG00000159433	-1.2891049	0.00274229
ENSG00000159733	-1.7360938	8.22E-07
ENSG00000159788	-1.2633859	6.79E-09
ENSG00000159860	1.21587971	0.04099346
ENSG00000159917	1.24875222	0.04247189
ENSG00000160007	-1.2605599	1.75E-08
ENSG00000160013	-1.718914	1.86E-06
ENSG00000160051	1.30038586	0.00907856
ENSG00000160179	-1.8445259	2.39E-09
ENSG00000160190	-1.2124036	0.03005537
ENSG00000160193	1.32467257	3.01E-08
ENSG00000160223	-1.2859126	0.00047029
ENSG00000160271	-1.5219254	1.02E-07
ENSG00000160299	-1.2034083	0.00038935
ENSG00000160336	1.29650515	0.00056742
ENSG00000160447	-1.263534	7.96E-06
ENSG00000160685	-1.2134032	0.00032423
ENSG00000160703	-1.2573671	0.00048177
ENSG00000160712	-1.4182458	0.0001143
ENSG00000160796	-1.4419685	6.32E-09
ENSG00000160799	-1.2348201	0.00031488
ENSG00000160813	-1.2089603	0.00421293
ENSG00000160888	-3.1698883	4.74E-25

Gene ID	FC 1HG - 4HG	adj p-value 1HG - 4HG
ENSG00000160949	-1.3410644	0.00026034
ENSG00000161642	-1.2433007	0.00092909
ENSG00000161714	-1.2975347	6.43E-05
ENSG00000161800	-1.2582209	0.00227855
ENSG00000161835	-1.2709781	0.00071536
ENSG00000161888	-1.2152493	0.00857839
ENSG00000161914	-1.2450445	0.04479842
ENSG00000161940	-1.9819934	1.51E-20
ENSG00000162063	-1.2288667	0.02082225
ENSG00000162066	-1.2207159	0.00702988
ENSG00000162073	-1.2806577	1.05E-05
ENSG00000162367	-1.3062118	1.95E-10
ENSG00000162390	1.51603683	3.41E-06
ENSG00000162407	1.85293665	1.17E-08
ENSG00000162409	-1.2145006	0.04738226
ENSG00000162413	-1.6819348	4.09E-15
ENSG00000162434	1.28812784	1.05E-09
ENSG00000162437	1.34875639	1.77E-05
ENSG00000162496	-1.5626291	1.07E-06
ENSG00000162522	-1.8231366	8.13E-16
ENSG00000162542	-1.2111652	0.03042744
ENSG00000162551	1.29638913	0.00252524
ENSG00000162599	-1.2610418	0.00070068
ENSG00000162614	1.44636834	9.27E-07
ENSG00000162616	1.52282291	8.40E-06
ENSG00000162636	1.36150018	0.00041669
ENSG00000162692	5.84599292	6.02E-20
ENSG00000162695	1.31579171	0.00468764
ENSG00000162714	-1.2970747	2.91E-07
ENSG00000162757	1.62777393	1.87E-07
ENSG00000162772	-2.2596462	0.00032739
ENSG00000162775	-1.2531118	3.08E-06

Gene ID	FC 1HG - 4HG	adj p-value 1HG - 4HG
ENSG00000162783	-1.4448282	6.09E-09
ENSG00000162817	-1.3666764	3.06E-07
ENSG00000162944	-1.3791297	0.01795518
ENSG00000163026	1.29136851	2.91E-05
ENSG00000163050	-1.2623658	0.02378194
ENSG00000163083	-1.3798263	0.00184791
ENSG00000163110	1.56845177	4.08E-18
ENSG00000163155	1.51017878	6.11E-05
ENSG00000163162	-1.2769764	9.07E-07
ENSG00000163171	-2.0850743	3.16E-18
ENSG00000163219	-1.6941021	0.00041822
ENSG00000163235	2.13178454	1.88E-10
ENSG00000163251	-1.444615	0.00014549
ENSG00000163297	1.23285047	7.40E-05
ENSG00000163491	-1.5913199	0.00013431
ENSG00000163527	1.21366489	0.0058554
ENSG00000163545	-1.9639037	2.16E-07
ENSG00000163554	-1.45634	0.00057751
ENSG00000163577	1.29443346	0.01254578
ENSG00000163637	1.24458096	0.00601977
ENSG00000163638	-1.5438018	1.27E-08
ENSG00000163644	-1.2584021	0.00199475
ENSG00000163659	-1.8982774	4.62E-21
ENSG00000163660	-1.405262	2.22E-05
ENSG00000163788	-1.2666852	2.86E-06
ENSG00000163814	-1.2558494	3.0363E-05
ENSG00000163840	-1.2530376	0.00338035
ENSG00000163874	-2.4484814	1.70E-12
ENSG00000163918	-1.2894091	0.00529932
ENSG00000163935	1.53325114	3.63E-05
ENSG00000163947	-1.2397114	0.00137529
ENSG00000164011	1.2122152	0.02157205

Gene ID	FC 1HG - 4HG	adj p-value 1HG - 4HG
ENSG00000164022	1.2723507	1.37E-06
ENSG00000164024	1.21941156	1.50E-05
ENSG00000164035	1.30064376	0.01437475
ENSG00000164038	1.20324439	0.00018438
ENSG00000164056	2.48695568	8.05E-11
ENSG00000164080	1.2315979	2.73E-08
ENSG00000164087	-1.2516659	0.01128014
ENSG00000164104	-1.3849985	1.52E-05
ENSG00000164125	1.32156003	0.00067411
ENSG00000164134	1.22335037	0.01993872
ENSG00000164161	1.3187089	3.99E-07
ENSG00000164220	1.32139702	0.03139197
ENSG00000164283	1.86403594	4.57E-12
ENSG00000164284	1.23822027	0.0424889
ENSG00000164292	1.54639036	7.09E-11
ENSG00000164294	1.21765715	0.00325806
ENSG00000164305	-1.2711689	1.00E-05
ENSG00000164306	-1.2181762	0.00609408
ENSG00000164323	-1.2337135	0.00498607
ENSG00000164338	1.27652475	8.37E-06
ENSG00000164442	1.51212463	1.52E-05
ENSG00000164484	1.28168616	4.32E-05
ENSG00000164506	1.35238463	4.36E-07
ENSG00000164512	2.10357318	1.08E-09
ENSG00000164649	-1.3748809	2.24E-13
ENSG00000164683	-4.4474632	3.64E-17
ENSG00000164684	2.97888309	3.46E-17
ENSG00000164736	1.36668914	1.02E-07
ENSG00000164741	-1.348488	1.47E-10
ENSG00000164743	1.31472478	0.00463132
ENSG00000164823	1.69048142	1.70E-10
ENSG00000164849	-1.9442799	1.87E-05

Gene ID	FC 1HG - 4HG	adj p-value 1HG - 4HG
ENSG00000164867	-1.3757232	6.22E-07
ENSG00000164877	-1.3098765	0.00214445
ENSG00000164975	1.22992775	0.00020015
ENSG00000165156	1.22640415	0.00160958
ENSG00000165233	-1.2487766	8.0684E-05
ENSG00000165240	1.2185128	0.00798013
ENSG00000165244	-1.5668152	1.38E-06
ENSG00000165271	1.30126433	1.52E-07
ENSG00000165304	-1.2707536	7.50E-05
ENSG00000165322	-1.2407328	1.18E-05
ENSG00000165338	1.51919192	0.00016147
ENSG00000165424	-1.2300255	0.00531454
ENSG00000165434	1.30676647	0.00074823
ENSG00000165494	-1.2216922	7.50E-05
ENSG00000165507	1.70992669	0.00018137
ENSG00000165572	1.38610298	9.88E-09
ENSG00000165655	-1.3703848	0.00138134
ENSG00000165732	1.2868103	2.90E-06
ENSG00000165757	-1.3615959	2.31E-14
ENSG00000165801	1.35915579	0.00199369
ENSG00000165806	-1.3569214	6.33E-14
ENSG00000165886	-1.3597708	2.80E-08
ENSG00000165891	1.31009192	3.77E-05
ENSG00000165895	1.35585861	0.0008968
ENSG00000165899	1.32533461	0.00102898
ENSG00000165959	-1.5166114	4.24E-09
ENSG00000166073	1.35639412	5.86E-07
ENSG00000166140	-1.2423494	0.00303222
ENSG00000166188	1.2252098	0.00045727
ENSG00000166250	1.28658651	0.00499701
ENSG00000166292	1.3075525	0.02823126
ENSG00000166398	1.20163028	0.00106803

Gene ID	FC 1HG - 4HG	adj p-value 1HG - 4HG
ENSG00000166439	-1.3480241	1.57E-09
ENSG00000166446	1.35962425	6.29E-11
ENSG00000166471	1.22943291	0.00843034
ENSG00000166483	-1.5099463	6.41E-07
ENSG00000166503	1.35643508	4.12E-07
ENSG00000166508	-1.2294605	0.00026275
ENSG00000166510	2.00786931	4.64E-08
ENSG00000166670	1.2376476	0.02382467
ENSG00000166704	1.49653318	1.97E-06
ENSG00000166801	-1.3817163	2.31E-05
ENSG00000166831	1.30630434	0.00061677
ENSG00000166851	-1.2810599	0.00566684
ENSG00000166881	-1.2271678	0.00876916
ENSG00000166886	-1.8879303	1.00E-10
ENSG00000166925	-1.6114833	6.41E-12
ENSG00000166949	-1.4412739	3.98E-14
ENSG00000166971	-1.2974534	7.16E-06
ENSG00000167081	1.30964045	3.84E-07
ENSG00000167083	-1.2288244	0.01716692
ENSG00000167106	-1.9757336	1.01E-14
ENSG00000167107	-1.2578165	0.00385642
ENSG00000167173	-1.2007538	0.00099262
ENSG00000167191	-1.7038084	4.81E-08
ENSG00000167202	-1.2434738	2.92E-07
ENSG00000167363	-1.3002655	0.00378231
ENSG00000167378	1.27757444	1.38E-09
ENSG00000167470	-2.4819028	1.17E-22
ENSG00000167487	-1.2731013	0.03812033
ENSG00000167513	-1.3273877	0.00487307
ENSG00000167562	1.20643662	0.03567105
ENSG00000167565	-1.7299521	1.04E-12
ENSG00000167600	-1.3555553	0.0043741

Gene ID	FC 1HG - 4HG	adj p-value 1HG - 4HG
ENSG00000167625	1.38195014	2.17E-07
ENSG00000167635	1.21953788	0.00096088
ENSG00000167670	-1.3407081	0.00164427
ENSG00000167680	-1.4354758	2.77E-09
ENSG00000167720	-1.2216904	0.00482828
ENSG00000167767	-1.2375172	0.0011428
ENSG00000167772	-1.6554662	5.03E-06
ENSG00000167861	-1.4433519	8.31E-07
ENSG00000167972	-1.2881112	3.46E-07
ENSG00000168010	-1.3302147	0.02957662
ENSG00000168016	-1.4305101	5.76E-07
ENSG00000168172	1.21612925	0.00022113
ENSG00000168209	-2.4407224	2.86E-11
ENSG00000168297	1.21703651	0.0039415
ENSG00000168309	-1.4271656	3.96E-07
ENSG00000168310	-1.3169954	1.72E-07
ENSG00000168386	1.29423728	0.0124836
ENSG00000168461	1.64752421	2.68E-15
ENSG00000168476	-1.2229804	0.00030843
ENSG00000168497	-1.8223905	2.52E-16
ENSG00000168502	1.20021187	0.00051963
ENSG00000168556	-1.2175294	0.03837388
ENSG00000168575	1.28778121	5.79E-07
ENSG00000168675	1.34734525	0.00186521
ENSG00000168685	1.54368466	0.00567637
ENSG00000168763	-1.3747582	1.5808E-05
ENSG00000168769	1.26930296	6.33E-08
ENSG00000168792	-1.3142926	4.13E-05
ENSG00000169131	1.23116305	0.01076668
ENSG00000169174	1.26387661	0.00364009
ENSG00000169184	-1.6122641	3.24E-05
ENSG00000169220	-1.2399578	0.03423624
ENSG00000169242	-3.5029162	5.33E-15
ENSG00000169247	-1.43131	0.00085851
ENSG00000169255	-1.3703404	2.38E-05
ENSG00000169258	-1.248719	0.00020462
ENSG00000169429	1.70503867	0.00705216
ENSG00000169439	1.27458305	0.00112894
ENSG00000169554	1.43881156	1.85E-13
ENSG00000169594	-1.3176203	3.87E-09
ENSG00000169607	-1.3143058	0.00515201
ENSG00000169629	1.39996867	0.00028836
ENSG00000169679	-1.2946618	0.00069603
ENSG00000169756	1.26412009	0.00382645
ENSG00000169851	1.47445977	6.11E-05
ENSG00000169902	1.20266623	0.00039998
ENSG00000169926	-1.2103175	0.00221036
ENSG00000169991	-1.2996698	9.80E-07
ENSG00000170011	-1.3227289	0.00453929
ENSG00000170017	1.27114676	3.66E-07
ENSG00000170085	1.22221321	0.00656209
ENSG00000170214	1.29759362	0.01538832
ENSG00000170271	-1.6509734	2.92E-06
ENSG00000170293	1.76803889	1.64E-09
ENSG00000170323	1.49469488	0.00367428
ENSG00000170345	-3.5415779	1.45E-11
ENSG00000170365	-1.6592706	1.13E-11
ENSG00000170425	-1.2956899	0.00399092
ENSG00000170485	-1.3353858	1.03E-09
ENSG00000170525	-1.5704703	5.51E-16
ENSG00000170537	1.28567982	0.00212035
ENSG00000170558	1.39697985	1.47E-12
ENSG00000170775	1.84108066	6.22E-16
ENSG00000170779	-1.2051923	0.00248796
ENSG00000170852	-1.3050953	6.96E-07
ENSG00000170873	-1.3812974	1.13E-06
ENSG00000170903	1.21645545	0.00450596
ENSG00000170921	1.38523705	6.07E-06
ENSG00000170962	1.29638574	0.01928399
ENSG00000171045	-1.2445899	0.04211967
ENSG00000171056	-1.344882	7.08E-06
ENSG00000171105	-1.3124182	0.00200726
ENSG00000171130	-1.2105456	0.00639533
ENSG00000171206	-1.327603	4.41E-10
ENSG00000171223	-5.6865462	1.06E-28
ENSG00000171227	-1.2877827	0.00677471
ENSG00000171246	1.92653059	1.91E-07
ENSG00000171295	1.31670883	0.00014455
ENSG00000171310	1.47211135	9.95E-07
ENSG00000171316	1.26207172	5.90E-05
ENSG00000171388	-1.6607632	1.20E-07
ENSG00000171435	-1.3960494	1.31E-06
ENSG00000171488	1.30834464	0.00021779
ENSG00000171552	-1.4335617	1.01E-10
ENSG00000171621	-2.9418349	1.68E-13
ENSG00000171658	1.33643828	0.00691503
ENSG00000171729	-1.4852128	1.19E-07
ENSG00000171823	-1.2085105	0.00142031
ENSG00000171827	1.40036284	0.00167049
ENSG00000171940	1.2626304	1.55E-05
ENSG00000171988	-1.284985	4.65E-05
ENSG00000172006	1.27920682	0.01501875
ENSG00000172031	-1.5260949	3.92E-05
ENSG00000172059	-1.4505069	1.07E-05
ENSG00000172159	1.37400661	4.24E-06
ENSG00000172216	-1.2923714	0.00021188

Gene ID	FC 1HG - 4HG	adj p-value 1HG - 4HG
ENSG00000172331	1.3114792	0.00173246
ENSG00000172572	1.22459268	0.00048798
ENSG00000172578	1.33596837	3.71E-09
ENSG00000172602	1.32366248	0.02196193
ENSG00000172716	-1.2619627	3.07E-10
ENSG00000172731	-1.2494716	0.00946142
ENSG00000172765	-1.3360218	8.16E-10
ENSG00000172819	1.3112779	0.00121005
ENSG00000172985	-1.2522858	0.0004684
ENSG00000173011	-1.2319986	1.32E-05
ENSG00000173040	-1.2499437	0.00642835
ENSG00000173068	-1.328601	2.01E-07
ENSG00000173210	-1.2168025	4.38E-09
ENSG00000173276	-1.3554393	2.99E-06
ENSG00000173281	-1.2519917	9.98E-06
ENSG00000173334	-2.1558908	3.87E-11
ENSG00000173530	-1.4500773	1.24E-10
ENSG00000173535	-1.4918805	1.20E-08
ENSG00000173548	-1.2209768	3.51E-05
ENSG00000173597	1.5894543	2.88E-05
ENSG00000173611	-1.2155234	0.04735043
ENSG00000173681	-1.2683397	0.01483788
ENSG00000173715	-1.2073268	0.00141008
ENSG00000173868	1.22470782	0.00114374
ENSG00000174004	-1.7006173	6.28E-07
ENSG00000174059	-1.8691682	3.73E-05
ENSG00000174130	-1.3509136	0.00158274
ENSG00000174136	-1.2533387	3.93E-06
ENSG00000174370	-1.4460754	0.00035378
ENSG00000174669	-1.2265537	0.04966702
ENSG00000174705	-1.275307	6.02E-08
ENSG00000174796	1.32402026	0.00836891

Gene ID	FC 1HG - 4HG	adj p-value 1HG - 4HG
ENSG00000174804	1.30427866	5.42E-11
ENSG00000175040	1.40100391	1.20E-07
ENSG00000175155	1.21692907	0.03269149
ENSG00000175264	-2.0808556	1.19E-16
ENSG00000175274	-1.2999869	1.16E-05
ENSG00000175426	1.22765074	0.04402005
ENSG00000175505	-1.4258146	0.0002328
ENSG00000175591	-1.2621944	0.03765359
ENSG00000175592	-1.9096323	9.91E-13
ENSG00000175746	1.29919788	0.00378731
ENSG00000175832	-1.3334934	2.33E-06
ENSG00000175906	1.51446513	1.61E-06
ENSG00000175938	-1.2530035	0.0320676
ENSG00000176055	1.27465946	0.00965656
ENSG00000176125	1.25806861	0.03528128
ENSG00000176155	-1.2863835	0.02136358
ENSG00000176209	-1.2123033	0.04386966
ENSG00000176371	1.22602157	0.04056165
ENSG00000176399	1.28027537	0.03188295
ENSG00000176438	-1.3698476	4.13E-07
ENSG00000176641	1.22747228	0.01043239
ENSG00000176692	-1.5102244	1.19E-07
ENSG00000176697	1.38813419	0.03837115
ENSG00000176714	1.29430734	0.01120962
ENSG00000176749	-1.8763078	3.21E-10
ENSG00000176834	1.24428277	5.11E-07
ENSG00000176842	-1.3080199	0.00443782
ENSG00000176907	-1.6229188	9.01E-05
ENSG00000176974	-1.251754	3.46E-06
ENSG00000177000	-1.2675227	0.00086717
ENSG00000177030	-1.2480574	0.00038395
ENSG00000177076	-1.3016918	0.00327238

Gene ID	FC 1HG - 4HG	adj p-value 1HG - 4HG
ENSG00000177125	-1.390253	5.82E-07
ENSG00000177173	-1.2202802	0.0473057
ENSG00000177283	-1.5051087	7.04E-09
ENSG00000177374	-2.5782286	4.22E-14
ENSG00000177409	-1.3254847	0.00517018
ENSG00000177464	1.21072724	0.00055531
ENSG00000177479	-1.232768	3.35E-08
ENSG00000177602	-1.3636231	0.00106424
ENSG00000177606	-1.8011837	5.05E-19
ENSG00000177679	1.55573951	1.58E-05
ENSG00000177683	1.32950952	0.01509246
ENSG00000177706	1.31913363	2.49E-10
ENSG00000177732	-1.2537685	0.00022728
ENSG00000177842	-1.316729	0.00319853
ENSG00000177888	1.38549396	0.03433188
ENSG00000177946	1.25733614	0.00130005
ENSG00000178031	1.20965277	7.57E-05
ENSG00000178033	1.31699799	3.95E-06
ENSG00000178038	-1.3861936	0.00019974
ENSG00000178104	1.25691973	5.0508E-05
ENSG00000178105	1.26099891	4.12E-07
ENSG00000178150	1.3249282	0.0188819
ENSG00000178229	1.24026473	0.01790159
ENSG00000178343	1.54270632	3.95E-07
ENSG00000178397	1.26696951	0.00107133
ENSG00000178401	1.3523809	0.00624572
ENSG00000178409	1.30790469	2.99E-06
ENSG00000178445	-1.2667244	0.00294936
ENSG00000178567	1.23552749	0.03709564
ENSG00000178568	1.22173882	0.02101367
ENSG00000178607	-1.2090516	0.00048645
ENSG00000178685	-1.2285714	0.0080755

Gene ID	FC 1HG - 4HG	adj p-value 1HG - 4HG
ENSG00000178700	1.27065416	0.00528776
ENSG00000178718	-1.3228968	7.86E-07
ENSG00000178726	-1.5950014	1.12E-09
ENSG00000178773	-1.3002729	0.00982052
ENSG00000178878	-1.6473514	0.00086976
ENSG00000178882	1.40282257	0.00230479
ENSG00000178935	1.31992172	0.00195166
ENSG00000178971	-1.2684455	0.00019161
ENSG00000178999	-1.2865678	0.00825057
ENSG00000179044	1.29666845	0.00645552
ENSG00000179219	1.26746003	2.10E-05
ENSG00000179314	-1.6190228	2.37E-09
ENSG00000179348	-2.023051	3.96E-12
ENSG00000179361	-1.5558718	2.62E-07
ENSG00000179388	-11.474376	2.36E-17
ENSG00000179403	1.61256122	0.00004451
ENSG00000179428	1.54593564	0.00062448
ENSG00000179456	-1.3841517	6.72E-09
ENSG00000179532	-1.2823126	0.00815643
ENSG00000179546	-1.4150208	0.00061324
ENSG00000179627	-1.2164228	0.03018073
ENSG00000179630	1.34272478	0.00704013
ENSG00000179715	-1.3822847	0.00629567
ENSG00000179862	-1.3840639	0.0014812
ENSG00000179941	1.48623922	0.0001482
ENSG00000179981	-1.5311341	4.11E-12
ENSG00000180035	-1.3221763	3.50E-05
ENSG00000180257	1.55665343	1.71E-06
ENSG00000180318	1.20812901	0.00386563
ENSG00000180448	-1.442965	0.00207114
ENSG00000180479	1.27076775	0.02305144
ENSG00000180530	-1.4362131	3.05E-07

Gene ID	FC 1HG - 4HG	adj p-value 1HG - 4HG
ENSG00000180592	-1.4072147	0.00059626
ENSG00000180626	1.29198877	0.0075145
ENSG00000180694	1.24615505	0.00344899
ENSG00000180776	1.21314969	0.0147288
ENSG00000180787	1.50728963	9.19E-06
ENSG00000180891	-1.2109268	0.00163756
ENSG00000180938	1.39584314	4.04E-05
ENSG00000180998	2.89261546	4.85E-13
ENSG00000181004	1.23484844	0.04834912
ENSG00000181007	1.30734267	0.00217847
ENSG00000181035	-1.351718	0.00506126
ENSG00000181274	1.24062376	0.00821782
ENSG00000181315	1.20203265	0.01717384
ENSG00000181409	-1.360889	0.00906294
ENSG00000181541	2.791983	4.65E-16
ENSG00000181634	-1.4467957	0.02707291
ENSG00000181638	1.2585704	0.00582301
ENSG00000181744	1.29763786	0.00042974
ENSG00000181751	1.39404286	1.16E-05
ENSG00000181773	-1.4822016	0.00013289
ENSG00000181852	1.21794665	2.92E-05
ENSG00000181896	1.2493334	0.00064722
ENSG00000181982	-1.2785459	4.61E-07
ENSG00000182022	-1.941036	1.46E-21
ENSG00000182118	-1.8281318	7.51E-07
ENSG00000182134	-1.2334649	0.00349904
ENSG00000182197	1.6165551	3.95E-22
ENSG00000182253	1.55729821	6.39E-08
ENSG00000182310	-1.3473556	0.00406136
ENSG00000182378	-1.244149	0.01955105
ENSG00000182489	1.25443046	0.02288711
ENSG00000182541	-1.3730996	1.84E-08

Gene ID	FC 1HG - 4HG	adj p-value 1HG - 4HG
ENSG00000182568	1.3257067	0.01393511
ENSG00000182742	1.25493708	8.31E-06
ENSG00000183049	2.04154876	2.40E-10
ENSG00000183054	1.33262065	0.0138145
ENSG00000183077	-1.2046985	0.01977082
ENSG00000183090	1.36242574	2.84E-05
ENSG00000183161	1.33142165	0.00081183
ENSG00000183208	-1.2327468	0.04301182
ENSG00000183337	-2.5373192	1.83E-13
ENSG00000183340	1.40461049	5.04E-09
ENSG00000183386	-1.3152857	1.3472E-05
ENSG00000183496	-1.4829094	1.25E-05
ENSG00000183578	1.29674212	3.1598E-05
ENSG00000183580	-1.4332635	5.00E-13
ENSG00000183638	-1.2921211	0.01603716
ENSG00000183691	-1.5143469	0.00061203
ENSG00000183779	-1.606387	1.55E-07
ENSG00000183808	1.30885379	9.30E-05
ENSG00000183856	-1.2517719	0.00346104
ENSG00000183873	-1.2013292	0.01586167
ENSG00000183971	1.29140727	0.02617533
ENSG00000184113	-1.8447986	5.27E-12
ENSG00000184226	2.32114679	4.71E-13
ENSG00000184304	1.45899741	8.44E-10
ENSG00000184307	1.38179168	0.00657372
ENSG00000184384	1.205398	0.00259402
ENSG00000184465	-1.2991812	0.00690107
ENSG00000184557	-4.0414729	2.21E-20
ENSG00000184635	-1.3676984	0.02675001
ENSG00000184640	-1.2026552	3.67E-06
ENSG00000184661	-1.3040167	0.00459229
ENSG00000184675	1.2656445	0.00543467

Gene ID	FC 1HG - 4HG	adj p-value 1HG - 4HG
ENSG00000184792	-1.2536266	0.00278035
ENSG00000184863	-1.2021763	1.85E-08
ENSG00000184916	1.24097821	0.00023901
ENSG00000185022	-2.6534658	2.76E-22
ENSG00000185033	-1.3637086	9.14E-05
ENSG00000185112	-1.6393385	5.53E-13
ENSG00000185187	-1.23623	0.00095435
ENSG00000185252	1.31364923	0.00016471
ENSG00000185262	-1.4930332	1.05E-09
ENSG00000185269	1.71682486	1.52E-10
ENSG00000185338	-1.4552331	0.0008165
ENSG00000185361	-1.8534942	6.36E-14
ENSG00000185567	-1.2353925	0.01010431
ENSG00000185614	-1.4241921	0.00097982
ENSG00000185920	-1.2583171	0.04261999
ENSG00000185950	-1.4956326	0.00598279
ENSG00000186020	1.2167109	0.03464954
ENSG00000186026	1.32377512	0.00798061
ENSG00000186185	-1.3412704	0.00244701
ENSG00000186193	-1.3976088	9.90E-07
ENSG00000186300	1.31235548	0.00247094
ENSG00000186432	1.20169692	0.00163279
ENSG00000186479	-1.458438	6.16E-06
ENSG00000186480	-1.2542301	0.02579225
ENSG00000186591	-1.3261606	5.04E-11
ENSG00000186635	-1.2729462	2.06E-06
ENSG00000186642	-1.4290967	1.0792E-05
ENSG00000186665	-1.2854709	0.00382645
ENSG00000186812	1.39889374	0.00122429
ENSG00000186871	-1.2458275	0.02930564
ENSG00000186918	-1.780933	4.58E-10
ENSG00000186994	-1.3116031	0.00052555

Gene ID	FC 1HG - 4HG	adj p-value 1HG - 4HG
ENSG00000187049	1.24715957	0.0037538
ENSG00000187189	1.20311931	0.00011895
ENSG00000187266	-1.5446131	8.89E-09
ENSG00000187479	-1.7209336	3.88E-06
ENSG00000187498	1.22043883	0.00010601
ENSG00000187535	-1.2017835	0.01625549
ENSG00000187555	1.22002401	2.12E-08
ENSG00000187566	1.42504456	7.03E-06
ENSG00000187605	-1.3902415	4.51E-09
ENSG00000187607	1.21489295	0.00579475
ENSG00000187626	1.51052869	7.75E-06
ENSG00000187634	1.41155532	0.00377865
ENSG00000187676	1.21479269	0.00170276
ENSG00000187678	-2.4752475	8.79E-20
ENSG00000187741	-1.240348	0.01908892
ENSG00000187764	-1.9534862	1.39E-11
ENSG00000187800	-1.4594468	3.25E-12
ENSG00000187815	1.40559964	0.00059096
ENSG00000188033	1.33680362	0.00113682
ENSG00000188042	-2.9155486	9.48E-15
ENSG00000188206	-1.3938434	0.01326194
ENSG00000188215	-1.3451592	2.14E-07
ENSG00000188277	-2.0812365	6.22E-05
ENSG00000188283	1.28433754	0.0081631
ENSG00000188295	-1.4443485	4.45E-05
ENSG00000188312	-1.2376155	0.01814377
ENSG00000188315	-1.2083086	0.04994792
ENSG00000188428	1.7840506	3.59E-09
ENSG00000188483	-2.7891576	2.41E-21
ENSG00000188522	-1.4879224	1.04E-07
ENSG00000188549	-1.2128437	0.01499741
ENSG00000188596	-1.2696517	0.01808919

Gene ID	FC 1HG - 4HG	adj p-value 1HG - 4HG
ENSG00000188732	-1.4123349	0.01406342
ENSG00000188811	-1.3001399	0.00502012
ENSG00000188997	1.38631244	1.29E-05
ENSG00000189067	1.31801708	0.00719826
ENSG00000189120	-1.2102366	0.03257225
ENSG00000189144	1.30290975	0.01152023
ENSG00000189298	1.28622872	0.01570898
ENSG00000196083	1.24188038	0.00571714
ENSG00000196110	1.33276249	0.00319432
ENSG00000196169	-1.8714164	3.02E-09
ENSG00000196187	-1.2252022	0.0055083
ENSG00000196209	-1.3026752	0.00944608
ENSG00000196214	1.2348411	0.00270358
ENSG00000196227	1.29490968	0.00785033
ENSG00000196268	1.20602982	0.04730983
ENSG00000196352	1.27002	3.74E-05
ENSG00000196371	-1.2452898	1.30E-05
ENSG00000196378	1.23137215	0.04790295
ENSG00000196411	1.27095839	2.38E-07
ENSG00000196421	-1.3063823	0.00153413
ENSG00000196422	-1.2097817	0.01932793
ENSG00000196458	1.21295474	0.03884729
ENSG00000196526	1.2365102	3.09E-10
ENSG00000196544	1.24115793	0.01016532
ENSG00000196646	1.28062127	0.00350648
ENSG00000196670	1.40761078	2.73E-07
ENSG00000196730	-1.6334856	5.00E-18
ENSG00000196781	-1.4628344	1.83E-13
ENSG00000196782	-1.7647987	1.59E-17
ENSG00000196792	1.24799602	6.81E-05
ENSG00000196812	-1.3623354	0.01875442
ENSG00000196843	-1.5583681	1.73E-07

Gene ID	FC 1HG - 4HG	adj p-value 1HG - 4HG
ENSG00000196876	-1.495755	4.75E-07
ENSG00000196890	-1.2422473	0.02693276
ENSG00000196981	1.27572206	0.0189239
ENSG00000197016	1.4424245	0.00040885
ENSG00000197019	-2.0742061	8.06E-17
ENSG00000197044	1.34409649	0.01316884
ENSG00000197050	1.25825384	0.01062857
ENSG00000197063	-1.4058924	1.97E-10
ENSG00000197119	-1.2519673	0.0034817
ENSG00000197128	1.31078273	0.00023183
ENSG00000197162	1.2844311	0.000877
ENSG00000197182	-1.3271445	0.02764388
ENSG00000197183	-1.3719701	2.84E-11
ENSG00000197256	-1.4048735	2.32E-12
ENSG00000197261	-1.3198802	0.00893491
ENSG00000197321	-1.2057036	4.04E-07
ENSG00000197329	-1.2299376	0.00670858
ENSG00000197380	1.34543809	0.01717969
ENSG00000197442	1.25103352	0.00055472
ENSG00000197536	-1.3388381	0.00350388
ENSG00000197557	1.21419147	0.03978371
ENSG00000197566	1.33482755	0.00086875
ENSG00000197619	1.3398991	0.00448595
ENSG00000197622	-1.2015123	2.63E-09
ENSG00000197852	-1.7147783	1.34E-13
ENSG00000197857	1.51092582	6.05E-07
ENSG00000197860	1.32079364	6.33E-05
ENSG00000197872	-1.2831572	1.80E-08
ENSG00000197903	-1.2454624	0.02208832
ENSG00000197905	-1.2303206	0.00010388
ENSG00000197928	1.28237659	0.00080735
ENSG00000197971	-1.24693	0.00357557

Gene ID	FC 1HG - 4HG	adj p-value 1HG - 4HG
ENSG00000198018	1.34992173	3.31E-07
ENSG00000198042	1.25459946	3.34E-06
ENSG00000198055	-1.2247528	0.00018819
ENSG00000198060	1.30959454	5.74E-07
ENSG00000198108	3.53080354	1.22E-16
ENSG00000198142	-1.3965649	0.00015458
ENSG00000198182	1.51121065	1.40E-06
ENSG00000198205	1.36124234	1.84E-06
ENSG00000198208	-1.4312943	0.00030313
ENSG00000198298	1.41292111	0.00044171
ENSG00000198324	-1.2529711	9.02E-05
ENSG00000198342	1.41889914	0.00010747
ENSG00000198346	1.44867888	1.1877E-05
ENSG00000198355	-2.299016	6.76E-19
ENSG00000198369	-1.4831147	1.05E-09
ENSG00000198431	1.22525008	0.00079499
ENSG00000198435	-3.6130475	1.16E-19
ENSG00000198440	1.30231075	0.01072617
ENSG00000198455	1.26210803	1.75E-05
ENSG00000198517	-1.9457958	1.36E-16
ENSG00000198521	1.34858856	0.00153335
ENSG00000198538	1.32591721	5.50E-05
ENSG00000198576	-3.0717592	7.44E-10
ENSG00000198604	1.34373761	1.36E-07
ENSG00000198648	1.23487945	0.00118963
ENSG00000198722	-1.2522646	1.28E-09
ENSG00000198743	1.29496232	0.02087841
ENSG00000198753	-1.2374982	0.02278901
ENSG00000198774	2.010544	2.86E-10
ENSG00000198799	1.20405544	0.03961839
ENSG00000198846	1.4550281	0.00025538
ENSG00000198873	-1.4309408	1.69E-10

Gene ID	FC 1HG - 4HG	adj p-value 1HG - 4HG
ENSG00000198909	-1.2558491	2.85E-09
ENSG00000198929	-1.4229248	0.00045154
ENSG00000198933	-1.219166	0.00215414
ENSG00000198945	1.24157768	1.76E-08
ENSG00000203326	1.25724835	0.02031938
ENSG00000203485	-1.3313553	5.08E-08
ENSG00000203709	1.66280461	8.02E-05
ENSG00000203805	1.64439861	5.19E-07
ENSG00000203883	-3.5827943	1.67E-16
ENSG00000204054	-1.2218211	0.00024131
ENSG00000204103	-1.4701282	6.04E-05
ENSG00000204131	-1.2450275	1.43E-05
ENSG00000204217	2.02078195	1.19E-14
ENSG00000204403	1.24200015	0.00281039
ENSG00000204406	-1.4103802	4.96E-07
ENSG00000204442	1.36865756	0.00019783
ENSG00000204520	1.24296587	0.0037399
ENSG00000204604	1.21582506	0.00060104
ENSG00000204611	1.62143022	1.20E-12
ENSG00000204634	-1.304711	2.65E-06
ENSG00000204767	1.32581476	8.70E-09
ENSG00000204856	1.23057186	0.01129661
ENSG00000204954	1.2238856	0.028221
ENSG00000204977	1.22398275	0.02851553
ENSG00000205138	1.31425236	0.00013651
ENSG00000205181	1.29354691	0.01240956
ENSG00000205213	-1.3168061	0.00061277
ENSG00000205269	-1.6648051	8.57E-05
ENSG00000205356	-1.2782685	1.09E-06
ENSG00000205476	-1.4480201	2.13E-09
ENSG00000205683	-1.5741208	1.18E-09
ENSG00000205808	1.3375461	0.00054926

Gene ID	FC 1HG - 4HG	adj p-value 1HG - 4HG
ENSG00000206532	1.22326565	0.01427304
ENSG00000206538	2.67413718	9.37E-17
ENSG00000206560	-1.3558801	3.46E-09
ENSG00000207808	-1.2452235	0.00644278
ENSG00000207980	-1.6324781	2.38E-05
ENSG00000211455	1.6640718	2.51E-07
ENSG00000213064	1.29964519	0.00010566
ENSG00000213089	-1.2562238	0.01403624
ENSG00000213160	-1.6523802	3.79E-06
ENSG00000213190	1.22740767	0.00267243
ENSG00000213347	-1.5272197	1.01E-06
ENSG00000213445	-1.2253196	1.47E-05
ENSG00000213626	1.5125295	6.21E-05
ENSG00000213672	-1.2369836	0.00509248
ENSG00000213694	-1.3870537	8.52E-10
ENSG00000213853	-1.2474746	0.00117826
ENSG00000214106	-1.2438101	0.0087453
ENSG00000214357	-1.7616548	1.90E-05
ENSG00000214595	1.47318656	5.32E-05
ENSG00000214654	1.25710117	0.02729367
ENSG00000214826	-1.3516906	0.02265405
ENSG00000214944	-1.854924	1.55E-15
ENSG00000215012	-1.2831353	0.00023305
ENSG00000215447	1.20310344	0.00326692
ENSG00000215788	-1.3966149	0.00206186
ENSG00000215883	-1.4167341	0.00090136
ENSG00000216775	-1.499657	7.95E-06
ENSG00000221829	-1.3191231	7.42E-05
ENSG00000221909	1.28226609	0.00541577
ENSG00000221926	-1.3341611	7.69E-08
ENSG00000221963	-1.2257682	0.01991903
ENSG00000222047	-1.3117054	0.0097767

Gene ID	FC 1HG - 4HG	adj p-value 1HG - 4HG
ENSG00000222455	-1.2447611	0.00666309
ENSG00000223547	1.34261325	0.0022131
ENSG00000223768	1.22435533	0.00475498
ENSG00000224531	1.20329507	0.00944342
ENSG00000224959	-1.3573434	0.0013994
ENSG00000225206	1.48654718	0.00012078
ENSG00000225484	1.2591178	0.00106179
ENSG00000225614	-1.4374247	1.26E-09
ENSG00000225828	-1.3266267	0.04329922
ENSG00000225830	1.58729394	1.60E-11
ENSG00000225968	2.98676861	1.03E-13
ENSG00000226348	-1.2031765	0.00701147
ENSG00000226752	1.22970484	0.00647537
ENSG00000226808	1.39489115	5.44E-05
ENSG00000226823	1.25018846	0.0185915
ENSG00000227051	1.2022931	0.00784185
ENSG00000227124	1.3554026	0.00135185
ENSG00000227517	1.33391477	0.00843152
ENSG00000228594	-1.2140182	0.04860529
ENSG00000229619	1.24550784	0.03020856
ENSG00000229953	-1.2927422	0.01053918
ENSG00000230183	-1.217553	0.00424812
ENSG00000230266	-1.5285849	7.22E-06
ENSG00000230438	-1.565841	1.42E-05
ENSG00000230479	-1.400405	8.78E-10
ENSG00000230701	-1.4070132	0.00511098
ENSG00000230753	-1.2877899	0.00122482
ENSG00000232098	1.21063262	0.03719973
ENSG00000232759	1.3289926	0.01077968
ENSG00000233237	1.69205055	2.78E-05
ENSG00000233370	1.46056456	3.45E-07
ENSG00000233611	1.23860957	0.02716902

Gene ID	FC 1HG - 4HG	adj p-value 1HG - 4HG
ENSG00000233757	1.43212747	0.00655459
ENSG00000233818	-1.2049325	0.0036903
ENSG00000234072	1.35648059	0.00337994
ENSG00000234284	1.32974621	0.00236846
ENSG00000234814	-1.2980521	0.00538552
ENSG00000234912	1.26373596	0.01885401
ENSG00000235194	1.31860415	0.02236385
ENSG00000235217	1.28334497	0.01157931
ENSG00000235501	-1.2095895	0.03020674
ENSG00000235831	-1.3171924	0.00029658
ENSG00000235944	-1.2038331	0.0362408
ENSG00000236144	1.29489327	0.0036073
ENSG00000236287	1.24005955	0.03072297
ENSG00000236515	1.25100959	0.02008315
ENSG00000237440	1.41609823	6.46E-05
ENSG00000237928	-1.2338614	0.00179735
ENSG00000239306	1.30944333	8.30E-10
ENSG00000239779	1.24398361	0.03992374
ENSG00000239887	-1.5307614	3.01E-07
ENSG00000240225	1.31371638	1.21E-05
ENSG00000240694	-1.6227913	1.95E-13
ENSG00000240857	1.25754641	0.00047879
ENSG00000240891	-1.2930043	0.00744463
ENSG00000241839	-1.3103771	1.45E-06
ENSG00000241852	-1.3670795	0.00029245
ENSG00000241978	-1.3194617	0.00031939
ENSG00000242193	-1.268005	0.0143146
ENSG00000242615	1.25103179	0.01453902
ENSG00000243244	1.30769761	0.03636843
ENSG00000244462	1.21253931	0.00018658
ENSG00000245213	-1.2366391	0.02866498
ENSG00000247626	1.2286794	0.00191759

Gene ID	FC 1HG - 4HG	adj p-value 1HG - 4HG
ENSG00000248008	1.27361062	0.00913508
ENSG00000248610	1.20820907	1.26E-06
ENSG00000249087	1.30286873	0.00185178
ENSG00000249279	-1.5981028	1.08E-06
ENSG00000249471	1.29538702	0.00449066
ENSG00000250303	1.46389931	3.40E-06
ENSG00000250510	-1.3421238	0.000358
ENSG00000250588	-1.212565	0.04182706
ENSG00000250899	-1.276074	8.23E-05
ENSG00000253210	-1.2113926	0.00717525
ENSG00000253276	1.45337886	4.77E-09
ENSG00000253368	1.52880358	2.90E-11
ENSG00000253661	1.42151699	0.00012348
ENSG00000253669	-1.4530806	5.51E-06
ENSG00000253738	1.23989217	0.02028134
ENSG00000253958	-1.2320528	0.02753746
ENSG00000254004	1.28825718	0.00138415
ENSG00000254087	1.37743574	1.03E-09
ENSG00000254470	-1.2544359	1.38E-06
ENSG00000255112	-1.207874	7.04E-05
ENSG00000255176	-1.7979673	9.81E-05
ENSG00000255248	1.39535867	0.00068234
ENSG00000255517	1.24699096	0.02441339
ENSG00000255583	1.25816464	0.01011111
ENSG00000255690	-1.2864787	0.01555473
ENSG00000256294	1.31293347	0.00054443
ENSG00000256448	-1.2291209	0.00011247
ENSG00000256594	1.2136987	0.01842198
ENSG00000256771	1.27216532	0.01443636
ENSG00000257167	-1.298368	0.02691422
ENSG00000257800	1.25274109	0.00615315
ENSG00000257923	1.34052131	8.37E-09

Gene ID	FC 1HG - 4HG	adj p-value 1HG - 4HG
ENSG00000258634	-1.2366136	0.00323546
ENSG00000259330	-1.3239568	0.00056215
ENSG00000259426	-1.2219982	0.00394766
ENSG00000259726	1.25497965	0.02085455
ENSG00000260628	-1.2746137	0.02605978
ENSG00000260822	-1.2105565	0.0225878
ENSG00000260920	1.27383877	0.00709371
ENSG00000260996	-1.3506985	0.00447199
ENSG00000261253	-1.4020073	0.00011044
ENSG00000261373	1.29927501	5.16E-05
ENSG00000261572	1.32270561	0.0035945
ENSG00000262074	-1.2458729	0.02507718
ENSG00000262292	-1.3393827	0.02145746
ENSG00000263002	1.29790591	0.00049569
ENSG00000263155	-1.6462209	1.57E-06
ENSG00000263528	1.24837165	0.00012585
ENSG00000265666	-1.2609301	0.03965484
ENSG00000265972	2.88453738	2.16E-19
ENSG00000265982	-1.2164766	0.04520102
ENSG00000266028	-1.2294073	1.73E-10
ENSG00000266208	-1.2511898	0.02876854
ENSG00000266236	-1.2180676	9.71E-05
ENSG00000267002	1.21492491	0.00510525
ENSG00000267041	-1.5693084	6.17E-06
ENSG00000267045	1.45457877	1.80E-06
ENSG00000267365	1.43979119	0.01953058
ENSG00000267508	1.30976344	0.01599121
ENSG00000267519	-2.4758438	8.18E-09
ENSG00000267583	-1.4638866	2.46E-09
ENSG00000267598	-2.9382847	9.07E-10
ENSG00000269019	-1.244726	0.02312426
ENSG00000269318	-1.3470172	0.00453373

Gene ID	FC 1HG - 4HG	adj p-value 1HG - 4HG
ENSG00000269343	1.21230469	0.00624125
ENSG00000269609	1.25702586	0.01371068
ENSG00000269890	1.37845517	0.00259944
ENSG00000269934	1.32884764	0.01298813
ENSG00000269973	-1.267221	0.02366224
ENSG00000270504	-1.2996045	0.00013036
ENSG00000270607	-1.2140873	0.03090157
ENSG00000272398	1.22102067	0.01549696
ENSG00000272405	-1.8088713	6.06E-06
ENSG00000272574	-1.2799082	0.0016248
ENSG00000272886	1.21631673	1.59E-05
ENSG00000272913	1.22886627	0.01715768
ENSG00000273489	-1.6477833	6.74E-05
ENSG00000273780	-1.3274857	7.38E-05
ENSG00000273888	1.32916607	0.00669998
ENSG00000274349	1.41998838	0.00034316
ENSG00000274636	-1.831444	3.18E-06
ENSG00000275074	1.31811461	0.00740196
ENSG00000275111	1.48791708	6.81E-06
ENSG00000275160	-1.226936	0.0311768
ENSG00000275342	-1.3955304	9.58E-06
ENSG00000275793	-1.2953799	4.39E-05
ENSG00000275993	-4.0436114	9.74E-20
ENSG00000276043	-1.2833416	0.00011377
ENSG00000276340	1.34682602	2.99E-05
ENSG00000276386	-1.2707784	4.82E-05
ENSG00000276600	1.40911344	0.00014439
ENSG00000276644	1.31277059	0.01221993
ENSG00000276649	-1.226322	0.04351787
ENSG00000276836	-1.3360775	0.00996827
ENSG00000277152	-1.3398463	0.0119221
ENSG00000277534	1.38430754	0.00236154

Gene ID	FC 1HG - 4HG	adj p-value 1HG - 4HG
ENSG00000277775	-1.2860764	0.01736074
ENSG00000278318	1.25758738	7.9667E-05
ENSG00000278384	1.24439684	0.033545
ENSG00000278921	-1.260428	0.01950026
ENSG00000279095	1.21180952	0.00042161
ENSG00000279117	-1.612368	3.14E-13
ENSG00000279175	-1.2650984	0.04690569
ENSG00000279236	-1.200118	0.04476911
ENSG00000279289	1.51992992	0.00084662
ENSG00000279496	-1.2601658	0.02025846
ENSG00000279583	-1.214935	0.02581419
ENSG00000279608	-1.2869221	0.00556809
ENSG00000279821	-1.3407885	0.00417005
ENSG00000279822	-1.2356131	0.01597362
ENSG00000280138	-1.6466164	0.00018707
ENSG00000280187	1.42025301	0.00058421
ENSG00000280339	1.24323749	0.0153552
ENSG00000280381	-1.3663405	0.00980425
ENSG00000281005	1.30609133	0.00139165
ENSG00000281614	-1.3280277	0.00083754
ENSG00000281881	-1.9343321	5.13E-06
ENSG00000282057	1.33789201	0.00967898

Supplementary Table 4c. Differentially expressed genes between 4hr high glucose and 8hr high glucose samples

Ensembl gene IDs, Fold change (4HG-8HG) and FDR adjusted p-values of DEGs

Gene ID	FC 4HG - 8HG	adj p-value 4HG - 8HG
ENSG00000001167	-1.2614257	5.40E-06
ENSG00000001460	1.24218429	0.0330805
ENSG00000002587	-1.3983225	4.11E-05
ENSG00000003400	1.20811855	0.0001975
ENSG00000004660	1.53124767	3.03E-06
ENSG00000004799	1.25160983	0.02912451
ENSG00000006459	-1.7000201	3.18E-09
ENSG00000006468	1.4736867	3.69E-06
ENSG00000006607	-1.2562024	1.20E-07
ENSG00000006652	-1.3386008	0.00057366
ENSG00000007384	1.29565795	0.00141017
ENSG00000007908	-6.4555364	5.59E-12
ENSG00000007944	1.35222397	3.51E-05
ENSG00000007968	2.44619528	4.53E-11
ENSG00000008083	1.30180148	0.00040301
ENSG00000008294	-1.2899427	1.39E-11
ENSG00000008405	-1.5065295	1.82E-09
ENSG00000010818	-1.6463664	6.73E-07
ENSG00000011143	1.23997472	0.02314613
ENSG00000011332	1.29021789	0.01023084
ENSG00000011426	-1.2502193	0.00221402
ENSG00000013016	1.28243002	0.01036667
ENSG00000013293	1.44916185	9.81E-05
ENSG00000013573	1.32349338	0.00030979
ENSG00000013588	1.33160084	0.00010043
ENSG00000015133	2.08626185	9.71E-15
ENSG00000015532	1.23728064	4.25E-06
ENSG00000015568	-1.7142497	5.83E-07
ENSG00000019102	1.52083137	0.00039595
ENSG00000019144	-1.294679	5.69E-07
ENSG00000020256	1.26079669	2.62E-06
ENSG00000020577	-1.2463611	3.17E-09

Gene ID	FC 4HG - 8HG	adj p-value 4HG - 8HG
ENSG00000021645	1.42335929	8.71E-05
ENSG00000021762	1.43891815	1.18E-09
ENSG00000022567	1.50902804	4.29E-05
ENSG00000023445	-2.762074	1.28E-08
ENSG00000023839	1.22025832	0.02686027
ENSG00000023902	-1.3251975	6.38E-06
ENSG00000025708	1.24256445	0.01918287
ENSG00000028137	1.41202654	4.20E-06
ENSG00000028277	1.63360398	2.61E-07
ENSG00000029993	-1.3581348	6.24E-09
ENSG00000031081	-1.3881771	1.66E-13
ENSG00000034152	-1.3335607	1.22E-05
ENSG00000037280	-1.2339026	0.04548534
ENSG00000040199	-1.2672755	3.34E-05
ENSG00000042286	1.2543782	0.00247516
ENSG00000043143	2.1551882	3.48E-15
ENSG00000047579	1.23959833	0.00014355
ENSG00000047617	1.37078908	0.00014392
ENSG00000047648	1.30418017	0.00936324
ENSG00000047932	-1.2123059	0.04377388
ENSG00000049130	-2.4604284	4.40E-08
ENSG00000049283	1.49936738	0.00549072
ENSG00000049618	-1.2375378	1.30E-07
ENSG00000049759	-1.2529709	0.00039584
ENSG00000050327	1.22566601	0.02761271
ENSG00000050820	1.27823297	1.58E-07
ENSG00000052723	1.20930759	0.01625573
ENSG00000052802	-1.4811487	0.02122119
ENSG00000056558	-2.6433911	4.28E-07
ENSG00000057252	-1.2444795	0.00434
ENSG00000057294	2.03194671	7.38E-09
ENSG00000057704	1.55982007	1.89E-11

Gene ID	FC 4HG - 8HG	adj p-value 4HG - 8HG
ENSG00000058453	1.21747604	0.01210252
ENSG00000059122	1.22573737	0.00033605
ENSG00000060491	1.23630307	7.25E-05
ENSG00000061938	1.32026235	3.38E-08
ENSG00000063438	1.35828769	2.25E-05
ENSG00000064225	-1.4534963	0.00011188
ENSG00000064309	1.21384782	0.0440746
ENSG00000064999	1.4066539	3.96E-07
ENSG00000065308	1.2096411	2.84E-05
ENSG00000065328	1.43112197	9.35E-05
ENSG00000065559	-1.267746	3.69E-05
ENSG00000065809	1.31971619	1.59E-10
ENSG00000065911	-1.2167579	6.37E-05
ENSG00000066084	-1.3048102	1.78E-06
ENSG00000066735	2.23817189	1.89E-16
ENSG00000066933	1.22303207	0.00060688
ENSG00000067064	-1.5107574	6.97E-05
ENSG00000067221	1.26619827	0.00085825
ENSG00000067606	1.21766733	0.04282063
ENSG00000067715	1.41969627	0.0003103
ENSG00000067798	-1.6443978	1.51E-16
ENSG00000067955	-2.0482743	6.87E-13
ENSG00000068024	1.31056992	3.41E-07
ENSG00000068078	-1.3417223	0.00023784
ENSG00000068137	1.8672824	1.67E-08
ENSG00000068305	-1.3128194	2.95E-05
ENSG00000068489	-1.2646197	8.34E-06
ENSG00000068971	1.24480218	0.00197269
ENSG00000069702	1.64357543	2.69E-08
ENSG00000069812	1.22343479	0.00037419
ENSG00000070961	-1.2776762	0.00305867
ENSG00000071205	-1.283219	4.59E-06

Gene ID	FC 4HG - 8HG	adj p-value 4HG - 8HG
ENSG00000071539	-1.2049737	0.00276215
ENSG00000072071	1.24123079	0.00195873
ENSG00000072422	-1.3083072	1.84E-07
ENSG00000073331	1.30097586	0.00022173
ENSG00000073417	-1.5051703	1.03E-13
ENSG00000073464	1.20901068	0.00205407
ENSG00000073605	1.4042664	0.00768931
ENSG00000073712	-1.3214389	3.97E-10
ENSG00000073756	-5.3121027	7.86E-11
ENSG00000073910	1.22899174	2.65E-05
ENSG00000074047	-2.6088991	1.14E-14
ENSG00000074855	1.58794795	1.17E-07
ENSG00000075240	1.20034617	0.01703302
ENSG00000075643	1.23848893	0.03215891
ENSG00000075651	1.58761084	7.62E-12
ENSG00000076248	1.305472	5.53E-08
ENSG00000076351	1.51256771	3.94E-07
ENSG00000076382	-1.2405676	0.00113828
ENSG00000077044	-1.2038808	0.00409943
ENSG00000077514	1.26530558	4.92E-05
ENSG00000078018	1.56594278	1.04E-05
ENSG00000078401	1.38733426	2.94E-07
ENSG00000078687	1.26050941	5.55E-06
ENSG00000078747	-1.2654691	1.75E-06
ENSG00000079102	-1.4985885	1.93E-05
ENSG00000079308	-1.393073	2.63E-08
ENSG00000079335	-1.2002388	0.00806011
ENSG00000080561	1.21200077	0.00018002
ENSG00000080608	-1.3401813	4.76E-09
ENSG00000080839	1.21782149	0.04944387
ENSG00000081059	-1.3820225	0.00126568
ENSG00000081189	-1.3110285	3.66E-06

Gene ID	FC 4HG - 8HG	adj p-value 4HG - 8HG
ENSG00000081665	-1.2357498	0.02931525
ENSG00000081803	1.25749783	3.30E-09
ENSG00000081913	-1.3167036	0.00619817
ENSG00000081923	1.27326153	0.01134656
ENSG00000084093	-1.8442798	1.16E-13
ENSG00000084710	-1.2901999	0.02428394
ENSG00000084734	1.25637042	0.00610933
ENSG00000085185	1.77147144	3.91E-10
ENSG00000085449	-1.2448988	7.83E-06
ENSG00000085840	1.51509968	5.27E-05
ENSG00000086544	1.2491382	3.78E-05
ENSG00000087266	1.37053903	2.73E-07
ENSG00000087494	-1.4542383	0.00181861
ENSG00000087586	-1.2543001	0.00073736
ENSG00000087903	-1.4931606	0.00105663
ENSG00000088387	1.40341055	5.00E-12
ENSG00000088543	1.44659257	0.00029116
ENSG00000088826	-1.2828544	7.13E-05
ENSG00000089041	1.68376044	1.47E-05
ENSG00000089048	-1.2721416	8.56E-05
ENSG00000089177	1.22584053	0.00179289
ENSG00000089685	-1.231042	0.00081642
ENSG00000090447	1.28705149	0.00238662
ENSG00000090612	-1.241873	0.00327662
ENSG00000090889	-1.2539998	0.00396052
ENSG00000091129	1.21152611	0.00092133
ENSG00000091428	-1.3958432	3.56E-05
ENSG00000091844	1.25658858	0.0249356
ENSG00000091879	1.25930817	4.09E-05
ENSG00000092470	1.42810562	7.76E-06
ENSG00000092621	-1.2966863	0.00036113
ENSG00000092929	1.21141799	0.0429347

Gene ID	FC 4HG - 8HG	adj p-value 4HG - 8HG
ENSG00000092969	-1.3940455	0.0129331
ENSG00000094804	1.50827435	6.15E-07
ENSG00000094975	-1.2115574	0.02440687
ENSG00000095015	-1.3087235	6.78E-06
ENSG00000095370	1.55625618	5.80E-11
ENSG00000095383	1.62811491	4.43E-08
ENSG00000095637	1.26810489	0.04361606
ENSG00000095739	-1.2958767	0.00436199
ENSG00000095752	-2.4746147	6.68E-11
ENSG00000096070	-1.2339766	2.66E-07
ENSG00000099251	-1.2917834	0.02782574
ENSG00000099625	1.38080341	0.00032729
ENSG00000099849	1.3139492	0.00033502
ENSG00000099860	1.24523768	0.0401155
ENSG00000099875	1.49536674	8.96E-11
ENSG00000099889	1.2760325	0.00080887
ENSG00000099910	1.35467275	7.43E-05
ENSG00000100036	1.38274224	1.38E-06
ENSG00000100065	1.47107781	6.16E-09
ENSG00000100221	1.21958836	2.82E-09
ENSG00000100292	-1.2276511	0.00605108
ENSG00000100417	1.22718275	0.02765029
ENSG00000100490	1.27130747	0.00650871
ENSG00000100592	-1.4025143	6.37E-11
ENSG00000100731	-1.2318322	0.00029563
ENSG00000100814	-1.206577	0.00018298
ENSG00000101040	1.35602007	3.31E-10
ENSG00000101096	-1.2477113	0.00100901
ENSG00000101187	1.41001415	0.00034939
ENSG00000101191	1.31327097	5.16E-12
ENSG00000101236	-1.3424181	4.07E-08
ENSG00000101255	1.25010446	0.00014419

Gene ID	FC 4HG - 8HG	adj p-value 4HG - 8HG
ENSG00000101265	1.32331776	3.2075E-05
ENSG00000101298	1.21921234	0.00023007
ENSG00000101331	1.55981627	2.99E-09
ENSG00000101384	-1.7551405	2.43E-10
ENSG00000101412	1.37503503	0.00098347
ENSG00000101445	1.53696284	4.16E-08
ENSG00000101577	1.25468251	2.11E-07
ENSG00000101665	-1.9616462	7.03E-06
ENSG00000101670	-1.8505286	9.80E-14
ENSG00000101751	1.27424372	0.0126512
ENSG00000101871	1.23021769	8.48E-05
ENSG00000102096	1.29724449	0.00604962
ENSG00000102178	1.33585247	1.12E-07
ENSG00000102221	-1.2217794	3.70E-05
ENSG00000102287	1.3770883	0.01448082
ENSG00000102349	1.20184034	0.04697311
ENSG00000102385	1.26018898	0.02413897
ENSG00000102531	-1.3981924	2.09E-12
ENSG00000102755	-1.4871718	3.23E-10
ENSG00000102796	1.28236553	0.02644405
ENSG00000102879	1.25051765	0.02678298
ENSG00000102886	1.38909204	0.00434098
ENSG00000102935	-1.7946824	1.07E-06
ENSG00000102967	1.22456612	0.0373002
ENSG00000103034	1.28230017	0.00056861
ENSG00000103254	1.24021926	0.01296042
ENSG00000103319	1.22173655	3.83E-08
ENSG00000103528	-1.2041852	0.03288913
ENSG00000103647	-1.3539726	4.63E-07
ENSG00000104154	1.26533358	0.01790643
ENSG00000104419	-1.4335202	1.73E-10
ENSG00000104447	-1.2175587	0.0211893

Gene ID	FC 4HG - 8HG	adj p-value 4HG - 8HG
ENSG00000104450	-1.44688	1.70E-05
ENSG00000104549	-1.3754841	7.63E-08
ENSG00000104738	1.23153976	0.00117646
ENSG00000104856	-1.5404963	4.58E-06
ENSG00000105088	-1.26346	0.00453703
ENSG00000105173	1.23644649	0.01586359
ENSG00000105245	1.28573819	7.87E-06
ENSG00000105281	-1.2132716	0.00036259
ENSG00000105287	1.27222509	5.42E-06
ENSG00000105538	1.3039356	5.06E-08
ENSG00000105639	1.3469079	0.02236754
ENSG00000105662	1.41295592	5.25E-06
ENSG00000105793	-1.2824022	0.01188506
ENSG00000105810	-1.2905227	0.00098622
ENSG00000105889	1.23953786	0.00190074
ENSG00000105939	1.36063574	6.64E-11
ENSG00000106070	-2.0176817	2.57E-19
ENSG00000106100	1.21671598	0.00086902
ENSG00000106351	1.70052808	9.85E-11
ENSG00000106404	1.38494762	5.49E-05
ENSG00000106479	1.51673962	1.02E-05
ENSG00000106546	-1.4062075	3.26E-10
ENSG00000106571	1.31641279	3.31E-07
ENSG00000106688	1.28084911	2.77E-08
ENSG00000106733	1.2646704	0.00929241
ENSG00000106799	-1.3070553	0.00069334
ENSG00000106948	1.36942039	8.76E-06
ENSG00000106993	-1.209645	0.01212154
ENSG00000107077	1.25719574	0.00546204
ENSG00000107099	-1.5472737	3.61E-06
ENSG00000107104	-1.5976761	2.58E-17
ENSG00000107185	1.26726598	1.97E-06

Gene ID	FC 4HG - 8HG	adj p-value 4HG - 8HG
ENSG00000107249	-1.4949417	5.4881E-05
ENSG00000107485	1.2866204	0.000284
ENSG00000107551	1.39479806	0.00262297
ENSG00000107562	-1.3990223	8.64E-05
ENSG00000107643	-1.2069627	0.00073458
ENSG00000107731	-4.189297	2.02E-18
ENSG00000107863	-1.6214846	3.91E-18
ENSG00000107968	-1.3939552	0.01657217
ENSG00000107984	-1.4659475	4.00E-06
ENSG00000108262	1.31083934	1.72E-08
ENSG00000108312	-1.7358148	3.50E-17
ENSG00000108352	1.39492159	0.00018524
ENSG00000108389	1.20465453	4.99E-08
ENSG00000108433	-1.2861745	5.68E-06
ENSG00000108551	1.20873754	0.02578576
ENSG00000108654	-1.2040506	3.36E-05
ENSG00000108691	-1.7293349	8.48E-06
ENSG00000108854	-1.2717633	5.84E-07
ENSG00000108960	-1.66657	3.34E-07
ENSG00000109084	-1.2097268	0.00046926
ENSG00000109189	-1.2604882	0.00101458
ENSG00000109265	-1.2359212	0.01279138
ENSG00000109320	-1.4199377	2.01E-16
ENSG00000109321	-1.2656823	0.00028092
ENSG00000109458	1.22340035	0.0243488
ENSG00000109466	-1.4237814	7.06E-11
ENSG00000109686	1.24411168	2.42E-07
ENSG00000109756	-1.3445335	2.44E-08
ENSG00000109906	1.84800639	3.68E-08
ENSG00000109929	-1.4836505	0.0008709
ENSG00000110330	-1.4761029	3.53E-06
ENSG00000110675	1.2702574	0.02059365

Gene ID	FC 4HG - 8HG	adj p-value 4HG - 8HG
ENSG00000110721	-1.2099205	0.00291278
ENSG00000110876	1.22619468	0.00084691
ENSG00000111077	1.54354767	2.51E-11
ENSG00000111199	-1.2290789	0.04425841
ENSG00000111490	-1.458999	4.68E-07
ENSG00000111647	-1.3496174	0.0060654
ENSG00000111674	1.23835453	0.04167709
ENSG00000111725	1.48879289	3.68E-08
ENSG00000111846	1.52247587	2.00E-10
ENSG00000111859	-1.3903043	0.00084616
ENSG00000111912	-1.2016369	0.00041427
ENSG00000111961	1.53714705	3.99E-20
ENSG00000112033	-1.2610076	2.53E-06
ENSG00000112039	1.29542424	8.63E-05
ENSG00000112183	-1.6117228	9.09E-05
ENSG00000112210	-1.3902722	7.09E-07
ENSG00000112305	-1.2084049	0.0005292
ENSG00000112312	1.24536696	0.00014752
ENSG00000112320	-1.2191447	0.00039506
ENSG00000112379	-1.2241021	0.00022323
ENSG00000112406	-1.2846152	0.00111013
ENSG00000112541	-1.6879091	7.40E-07
ENSG00000112559	-1.4131835	1.4261E-05
ENSG00000112561	1.2040385	0.01059713
ENSG00000112658	-1.2928772	9.49E-11
ENSG00000112773	-1.4639747	0.02662628
ENSG00000112851	-1.2820831	0.00236781
ENSG00000112972	-1.655923	3.60E-06
ENSG00000112984	-1.2905837	9.72E-05
ENSG00000113161	-1.5207764	7.74E-07
ENSG00000113369	-1.4774955	1.41E-06
ENSG00000113522	1.22480039	1.87E-05

Gene ID	FC 4HG - 8HG	adj p-value 4HG - 8HG
ENSG00000113532	-1.5056001	0.00036599
ENSG00000113580	-1.9005061	5.44E-14
ENSG00000113742	-1.211879	0.00195109
ENSG00000114107	-1.2288596	0.04762135
ENSG00000114268	1.26046739	0.04929325
ENSG00000114315	1.54120928	0.00917509
ENSG00000114423	-1.3563296	2.54E-05
ENSG00000114529	-1.3598328	0.00102245
ENSG00000114670	1.26058427	0.03956
ENSG00000114698	1.26019681	0.00301075
ENSG00000114735	1.21511599	0.01016357
ENSG00000114739	-1.2161671	0.04319355
ENSG00000114796	-1.4694668	4.35E-06
ENSG00000114841	1.56350699	1.67E-05
ENSG00000114999	-1.2881334	4.85E-14
ENSG00000115163	-1.2633101	0.0100174
ENSG00000115170	-1.3811062	2.06E-12
ENSG00000115183	1.25664325	3.17E-07
ENSG00000115525	-1.3458261	0.00577563
ENSG00000115540	-1.2291467	0.02626498
ENSG00000115548	-1.4870778	3.68E-11
ENSG00000115657	1.32656981	0.00210473
ENSG00000115687	1.21539628	0.02349105
ENSG00000115738	-1.4824008	0.01568408
ENSG00000115825	-1.2210375	0.00085226
ENSG00000115896	-1.5500966	0.00018407
ENSG00000115946	-1.2275525	4.90E-05
ENSG00000116017	1.26206163	0.03153682
ENSG00000116062	1.29497501	9.20E-13
ENSG00000116128	-1.2995028	8.94E-06
ENSG00000116273	1.31109754	4.00E-06
ENSG00000116584	1.38972058	4.66E-11

Gene ID	FC 4HG - 8HG	adj p-value 4HG - 8HG
ENSG00000116641	-1.3005089	0.00024291
ENSG00000116741	-1.3934715	0.00018536
ENSG00000116815	-1.3736691	0.00447002
ENSG00000116833	1.20440558	0.03077068
ENSG00000117143	-1.3782268	1.45E-09
ENSG00000117174	-1.2110148	0.00344101
ENSG00000117228	-1.2298238	0.00018117
ENSG00000117479	-1.2109911	0.0486045
ENSG00000117595	-1.5402494	0.0020926
ENSG00000117724	-1.3176111	0.00033741
ENSG00000118260	-1.2038044	0.0048099
ENSG00000118402	-1.2943783	0.02323438
ENSG00000118503	-1.9159478	2.47E-08
ENSG00000118515	-1.2485095	2.85E-08
ENSG00000118655	1.21436965	0.01399773
ENSG00000118689	1.27649779	1.10E-05
ENSG00000118900	1.20954915	1.02E-08
ENSG00000118922	1.27878874	0.00251845
ENSG00000118985	-1.3922392	7.10E-09
ENSG00000119139	-1.3164932	1.34E-08
ENSG00000119673	1.28104475	0.01273019
ENSG00000119771	-1.3353119	8.12E-09
ENSG00000119787	-1.2592228	0.01243028
ENSG00000119862	1.39091334	4.84E-05
ENSG00000119938	-1.613131	1.84E-06
ENSG00000119965	-1.2041111	0.02570936
ENSG00000120129	1.21316789	0.027513
ENSG00000120278	1.93169156	1.48E-17
ENSG00000120279	-1.3282346	8.87E-06
ENSG00000120658	1.29337766	0.0355611
ENSG00000120693	-2.5232961	3.39E-14
ENSG00000120699	-1.2435042	0.00041029

Gene ID	FC 4HG - 8HG	adj p-value 4HG - 8HG
ENSG00000120738	-1.9252994	0.00152454
ENSG00000120875	1.74231435	2.30E-17
ENSG00000120899	1.30495396	0.00146001
ENSG00000121039	-1.2729427	0.00010779
ENSG00000121060	1.28433955	6.90E-09
ENSG00000121390	-1.2610984	5.75E-09
ENSG00000121406	-1.3117543	0.00085644
ENSG00000121797	1.24240948	0.00512471
ENSG00000121931	-1.2619869	0.00033271
ENSG00000121966	2.01343012	1.17E-13
ENSG00000121988	1.26280306	0.00677142
ENSG00000122068	-1.2734159	5.77E-07
ENSG00000122257	1.34860653	1.05E-10
ENSG00000122376	1.2086868	0.04211896
ENSG00000122386	1.30161319	0.00130582
ENSG00000122420	-1.2802917	0.00830672
ENSG00000122641	-1.2304891	0.03086818
ENSG00000122863	-1.4935988	2.96E-07
ENSG00000122870	-1.6731008	2.72E-08
ENSG00000122966	-1.2845223	0.00027104
ENSG00000123066	-1.3654952	2.96E-11
ENSG00000123094	-1.3672723	1.75E-06
ENSG00000123146	1.23760743	0.00964565
ENSG00000123700	-1.6313888	0.0009943
ENSG00000123815	1.28863118	0.00334388
ENSG00000123933	1.27271098	9.25E-05
ENSG00000123975	-1.3004292	0.00049008
ENSG00000124006	1.27905281	0.01114332
ENSG00000124019	1.20253193	0.01303616
ENSG00000124191	1.34739708	1.85E-05
ENSG00000124225	-2.1372632	4.57E-11
ENSG00000124440	1.31334975	0.01584465

Gene ID	FC 4HG - 8HG	adj p-value 4HG - 8HG
ENSG00000124444	-1.280827	0.00144713
ENSG00000124593	1.47145846	0.00032739
ENSG00000124762	-1.2011444	6.24E-08
ENSG00000124766	-1.2504494	3.15E-05
ENSG00000125089	1.34454882	4.12E-06
ENSG00000125266	-1.3192672	0.00075746
ENSG00000125454	1.25440656	0.00309643
ENSG00000125458	1.23454203	0.00742414
ENSG00000125629	1.26054252	0.04594044
ENSG00000125845	-2.0283762	8.92E-12
ENSG00000125898	-1.4034355	6.04E-05
ENSG00000125962	-1.2392255	0.00476596
ENSG00000125968	-1.5369342	0.00055982
ENSG00000126351	1.2749597	6.34E-06
ENSG00000126705	1.5978412	2.11E-08
ENSG00000126787	-1.3111476	0.00918971
ENSG00000126882	1.49376367	0.0003118
ENSG00000127080	-1.2686639	1.74E-05
ENSG00000127124	-1.4667659	1.06E-05
ENSG00000127419	1.33705483	0.00012964
ENSG00000127533	1.4329103	0.0001976
ENSG00000127824	1.21573643	0.0231848
ENSG00000128284	1.24295243	0.01134141
ENSG00000128394	1.24394253	0.02517411
ENSG00000128408	1.50794102	0.00101038
ENSG00000128594	-6.4012271	3.36E-20
ENSG00000128641	-1.3308968	4.96E-05
ENSG00000128656	-1.2193516	3.9524E-05
ENSG00000128805	1.37160898	1.01E-09
ENSG00000128833	-1.3381235	0.00101967
ENSG00000128917	2.41304064	1.32E-11
ENSG00000128923	1.36207644	5.42E-06

Gene ID	FC 4HG - 8HG	adj p-value 4HG - 8HG
ENSG00000128944	-1.2021209	0.00370319
ENSG00000129028	1.30796905	0.00223087
ENSG00000129055	1.21803524	0.0013931
ENSG00000129116	-1.2038139	0.02570395
ENSG00000129173	1.62592619	2.50E-08
ENSG00000129355	1.26058313	0.02461937
ENSG00000129422	1.51917491	6.69E-12
ENSG00000129474	1.60717392	1.13E-12
ENSG00000129595	-1.2166443	0.00126947
ENSG00000130021	1.25191204	4.22E-05
ENSG00000130024	-1.2869719	0.00016185
ENSG00000130066	-1.5042279	2.46E-06
ENSG00000130164	-1.5081674	8.13E-09
ENSG00000130201	-1.5940706	1.15E-06
ENSG00000130299	1.23160789	0.01098667
ENSG00000130305	1.59737827	5.00E-05
ENSG00000130338	-1.4980214	6.67E-11
ENSG00000130363	1.21387728	0.02429482
ENSG00000130449	1.25567074	2.28E-11
ENSG00000130517	1.33470824	1.08E-05
ENSG00000130590	1.49550375	1.52E-05
ENSG00000130702	1.42340979	2.45E-08
ENSG00000130758	1.24130222	0.00984402
ENSG00000130772	-1.2735003	0.00895678
ENSG00000130956	-1.3572869	1.14E-06
ENSG00000130997	1.27786617	0.00623191
ENSG00000131149	-1.8425812	5.04E-12
ENSG00000131378	-1.3484601	8.16E-10
ENSG00000131386	1.56302491	0.0002538
ENSG00000131669	-1.3017093	1.90E-06
ENSG00000131747	-1.2931932	0.00524078
ENSG00000131759	1.32357469	0.00092006

Gene ID	FC 4HG - 8HG	adj p-value 4HG - 8HG
ENSG00000131773	-1.3380298	0.00119695
ENSG00000131831	-1.3150129	0.00331321
ENSG00000131943	1.4463849	4.27E-09
ENSG00000131979	-1.426634	0.00067878
ENSG00000132205	1.62153499	1.77E-07
ENSG00000132286	-1.2839207	4.01E-06
ENSG00000132294	-1.2780698	0.00011443
ENSG00000132326	-1.3496136	0.00203247
ENSG00000132510	-1.3255467	5.40E-05
ENSG00000132613	1.24436457	0.00019798
ENSG00000132622	1.21902677	0.02066315
ENSG00000132623	1.39709668	0.00024458
ENSG00000132694	1.25818976	5.06E-07
ENSG00000132906	1.28334568	0.00012942
ENSG00000133056	1.34035352	0.00012488
ENSG00000133065	-1.2118495	1.93E-06
ENSG00000133101	1.20623132	0.01033564
ENSG00000133401	1.46772116	0.0004053
ENSG00000133424	1.52914359	5.58E-06
ENSG00000133561	1.29565395	3.39E-07
ENSG00000133574	1.23206916	0.01648067
ENSG00000133612	1.2868498	4.44E-07
ENSG00000133657	-1.5003864	9.94E-05
ENSG00000133773	-1.2515294	0.00049144
ENSG00000133794	-1.2364129	0.00268467
ENSG00000133805	1.29511548	0.01167436
ENSG00000133858	-1.2743533	0.00411505
ENSG00000133874	-1.5059525	1.91E-07
ENSG00000134057	-1.3198049	0.00010436
ENSG00000134138	1.23634342	0.00344381
ENSG00000134215	-1.228385	0.048796
ENSG00000134245	1.35367582	0.00051895

Gene ID	FC 4HG - 8HG	adj p-value 4HG - 8HG
ENSG00000134294	-1.4244527	0.00321742
ENSG00000134323	-1.2608644	0.04198965
ENSG00000134363	-1.2943292	0.00259688
ENSG00000134480	-1.2433908	0.00070829
ENSG00000134698	-1.2051693	0.00016384
ENSG00000134780	1.43378188	3.31E-06
ENSG00000134909	-1.2108777	0.01049605
ENSG00000135083	1.2222204	0.00877931
ENSG00000135269	-1.2179508	0.00011139
ENSG00000135272	-1.3633228	1.34E-05
ENSG00000135338	-1.4414406	0.00079102
ENSG00000135365	1.24557482	5.70E-05
ENSG00000135537	1.20627554	0.04655247
ENSG00000135547	-3.1210578	3.81E-12
ENSG00000135604	1.44937451	5.85E-08
ENSG00000135709	1.27219122	0.00032693
ENSG00000135723	-1.2119202	2.27E-05
ENSG00000135736	1.21536659	0.03177204
ENSG00000135966	1.50664338	4.70E-11
ENSG00000136044	-1.3401651	4.52E-07
ENSG00000136052	-1.2956062	0.03811436
ENSG00000136108	-1.2508136	0.02252509
ENSG00000136114	1.44079294	2.79E-08
ENSG00000136122	-1.2148943	0.0309302
ENSG00000136237	1.35229278	1.69E-05
ENSG00000136244	-1.5536016	0.00034883
ENSG00000136274	1.37557821	0.0070544
ENSG00000136379	-1.3459936	4.61E-05
ENSG00000136383	-1.3167223	2.37E-05
ENSG00000136404	-1.2514867	0.00105921
ENSG00000136490	-1.2674626	0.00010569
ENSG00000136560	1.31060773	4.15E-05

Gene ID	FC 4HG - 8HG	adj p-value 4HG - 8HG
ENSG00000136630	-1.6674962	6.75E-05
ENSG00000136709	1.26209065	3.09E-10
ENSG00000136732	-1.2178425	0.03043574
ENSG00000136869	-1.2473378	0.00019706
ENSG00000136935	1.37805685	1.18E-10
ENSG00000136997	1.2160876	0.00113875
ENSG00000137094	-1.4338271	2.46E-08
ENSG00000137145	1.31675847	1.29E-07
ENSG00000137177	-1.2046487	6.11E-06
ENSG00000137185	-1.2166942	0.02794582
ENSG00000137193	1.39200594	0.01125135
ENSG00000137203	-1.666378	5.51E-09
ENSG00000137331	1.32227863	0.00013491
ENSG00000137486	1.20277809	0.00098299
ENSG00000137494	-1.2974168	2.03E-05
ENSG00000137502	-1.3833664	0.00022728
ENSG00000137692	-1.2019221	6.13E-05
ENSG00000137693	-1.3342786	0.00020963
ENSG00000137700	1.25325459	0.01980844
ENSG00000137713	-1.2322057	1.47E-06
ENSG00000137727	-1.3954947	9.21E-05
ENSG00000137764	1.24985261	0.00027144
ENSG00000137804	-1.285989	0.00099756
ENSG00000137812	-1.3304322	0.01032585
ENSG00000137834	-5.3957084	1.92E-15
ENSG00000137872	-1.5851671	4.40E-06
ENSG00000137877	1.71384633	1.64E-06
ENSG00000137936	1.20345612	0.00015283
ENSG00000138101	1.21050793	0.02156493
ENSG00000138134	1.53045718	2.16E-06
ENSG00000138135	-1.5107334	1.38E-11
ENSG00000138623	-1.8556161	6.64E-06

Gene ID	FC 4HG - 8HG	adj p-value 4HG - 8HG
ENSG00000138650	-1.2267255	0.04338759
ENSG00000138675	-1.5104722	2.61E-05
ENSG00000138678	1.46430019	2.22E-07
ENSG00000138764	-1.3138184	0.01881191
ENSG00000138771	1.34537773	0.00034404
ENSG00000138944	-1.2146305	0.00728048
ENSG00000139083	-1.2633956	1.94E-07
ENSG00000139154	-1.27179	1.57E-05
ENSG00000139163	-1.2616679	0.00368172
ENSG00000139266	1.53198626	0.00159772
ENSG00000139289	1.21454019	0.0085357
ENSG00000139372	-1.2128637	0.00149816
ENSG00000139505	-1.2058881	0.03644073
ENSG00000139514	-1.2292593	8.10E-10
ENSG00000139517	-1.2853525	2.54E-05
ENSG00000139625	1.34422307	0.00056328
ENSG00000139651	1.279153	2.42E-05
ENSG00000139687	-1.2193577	0.01931313
ENSG00000139719	1.22332116	5.75E-05
ENSG00000139734	-1.256794	0.00199739
ENSG00000139946	-1.3574082	0.00168295
ENSG00000139998	-1.2218363	0.01021194
ENSG00000140450	-1.3050776	0.00745938
ENSG00000140464	1.22383087	1.21E-06
ENSG00000140691	1.21994985	0.00312776
ENSG00000140950	1.21243645	0.00014173
ENSG00000141068	1.35835176	4.61E-07
ENSG00000141384	-1.2477387	0.02195195
ENSG00000141391	1.23922459	0.03910885
ENSG00000141458	-1.2585475	3.75E-06
ENSG00000141480	1.22020977	0.01740144
ENSG00000141503	1.22206329	4.62E-06

Gene ID	FC 4HG - 8HG	adj p-value 4HG - 8HG
ENSG00000141655	1.38193671	4.79E-05
ENSG00000141668	1.42469142	0.03961777
ENSG00000142279	-1.695638	1.22E-10
ENSG00000142556	-1.2005077	0.00287164
ENSG00000142627	1.29010887	1.02E-06
ENSG00000143228	-1.2691188	0.02150269
ENSG00000143324	-1.2369091	8.81E-05
ENSG00000143341	-1.2862991	0.03065127
ENSG00000143344	1.4908602	5.23E-11
ENSG00000143375	1.26758743	0.01936363
ENSG00000143409	1.29529625	0.00163406
ENSG00000143434	1.23148776	0.00443694
ENSG00000143476	1.71531564	9.09E-08
ENSG00000143479	-1.4670877	1.85E-06
ENSG00000143507	-1.2184832	0.01919823
ENSG00000143578	1.23069274	0.03776018
ENSG00000143702	-1.2914762	0.00531582
ENSG00000143772	1.64310675	2.30E-11
ENSG00000143793	1.21984505	0.00385639
ENSG00000143816	1.48251771	0.00347541
ENSG00000143850	-1.3851316	1.9712E-05
ENSG00000143851	-1.3353722	0.03475348
ENSG00000143889	-1.2274439	0.00019705
ENSG00000144040	1.20070078	0.00591234
ENSG00000144283	-1.2011931	1.21E-06
ENSG00000144354	1.49027717	8.15E-08
ENSG00000144366	-1.311568	0.00082646
ENSG00000144485	-1.2775464	0.01677986
ENSG00000144642	1.20276998	0.00678003
ENSG00000144712	1.22093112	0.00548215
ENSG00000144749	1.28504879	9.94E-05
ENSG00000144802	-1.3962614	0.03676214

Gene ID	FC 4HG - 8HG	adj p-value 4HG - 8HG
ENSG00000144843	1.38599718	0.0002483
ENSG00000144893	-1.3578632	3.03E-06
ENSG00000145014	1.34668261	1.55E-07
ENSG00000145016	1.24989986	6.44E-07
ENSG00000145365	-1.3835905	4.4501E-05
ENSG00000145391	-1.2111461	5.15E-06
ENSG00000145431	-1.2612312	0.0440746
ENSG00000145555	-1.2123495	1.04E-05
ENSG00000145632	1.20210599	0.0097235
ENSG00000145685	-1.2302142	0.00025814
ENSG00000145817	-1.3866576	3.89E-06
ENSG00000145911	2.33709928	1.86E-14
ENSG00000145916	1.38886269	1.60E-06
ENSG00000146021	1.3323874	0.00033975
ENSG00000146054	1.23324616	0.00129866
ENSG00000146094	1.42360628	0.00223674
ENSG00000146232	-1.6098753	4.50E-11
ENSG00000146242	-1.2042769	0.00023057
ENSG00000146374	-1.3678804	0.020666
ENSG00000146469	1.23568916	0.00266362
ENSG00000146555	1.58926169	0.00214483
ENSG00000146676	-1.2337199	0.0231552
ENSG00000146678	1.62304524	6.62E-08
ENSG00000146776	1.3108214	0.00010147
ENSG00000146830	1.27001574	0.00084517
ENSG00000146834	1.2969198	5.00E-07
ENSG00000146859	-1.2422393	0.0418954
ENSG00000147119	1.2723605	0.00022492
ENSG00000147130	1.36523094	7.24E-08
ENSG00000147202	1.28215495	0.0088085
ENSG00000147408	-1.4013359	0.00014928
ENSG00000147642	1.4120032	0.00121305

Gene ID	FC 4HG - 8HG	adj p-value 4HG - 8HG
ENSG00000147650	-1.3747119	2.87E-06
ENSG00000147854	1.20020921	2.44E-05
ENSG00000147872	1.35126124	9.90E-07
ENSG00000148082	1.25140663	0.00097139
ENSG00000148411	1.24914357	1.20E-10
ENSG00000148737	-1.267351	3.66E-06
ENSG00000148773	-1.2189744	0.02398249
ENSG00000148926	1.87306538	3.51E-09
ENSG00000149177	-1.2844326	3.59E-06
ENSG00000149346	1.24823429	0.00176291
ENSG00000149548	-1.2398554	0.02798362
ENSG00000149636	1.33144807	2.48E-06
ENSG00000149798	1.21999062	8.40E-06
ENSG00000150347	-1.6336692	3.58E-05
ENSG00000150457	1.29438598	4.42E-08
ENSG00000150510	1.26728752	0.00094289
ENSG00000150551	1.24542804	0.00331417
ENSG00000150630	-1.6532054	2.90E-11
ENSG00000151012	-1.2992717	0.00844478
ENSG00000151014	-1.3228745	1.31E-08
ENSG00000151136	-2.0218441	1.19E-08
ENSG00000151150	1.27843277	7.59E-05
ENSG00000151151	-1.3835041	0.01106704
ENSG00000151229	-1.3296724	0.00631491
ENSG00000151247	-1.3352468	1.28E-05
ENSG00000151304	-1.2365163	0.00376278
ENSG00000151338	1.25244709	0.00512997
ENSG00000151689	1.29145775	1.71E-06
ENSG00000151702	1.27688066	4.64E-10
ENSG00000151718	-1.5178833	1.37E-17
ENSG00000151748	-1.5818904	6.40E-11
ENSG00000151789	1.23859764	0.0270565

Gene ID	FC 4HG - 8HG	adj p-value 4HG - 8HG
ENSG00000151929	-1.3004736	2.19E-08
ENSG00000152137	1.28373574	0.00012595
ENSG00000152256	-1.2076362	0.0017511
ENSG00000152348	1.37290247	0.00963301
ENSG00000152409	1.29103695	0.02228855
ENSG00000152518	1.39703945	4.40E-09
ENSG00000152689	-1.4345874	1.58E-05
ENSG00000152784	-1.2285209	0.01665907
ENSG00000152944	-1.2439824	0.04345436
ENSG00000152953	-1.3009421	8.80E-05
ENSG00000153029	1.21599521	0.02015734
ENSG00000153165	-1.3948362	0.00108381
ENSG00000153201	-1.2426191	0.00624647
ENSG00000153551	-1.4040749	5.22E-09
ENSG00000153558	-1.227119	0.00018659
ENSG00000153707	1.3699031	0.00748579
ENSG00000153814	-1.3702122	1.34E-05
ENSG00000153885	-1.2953878	3.72E-06
ENSG00000153993	1.34748624	0.00199369
ENSG00000154016	-1.5519803	1.08E-08
ENSG00000154122	-1.5922846	6.09E-12
ENSG00000154127	-1.3083116	6.91E-08
ENSG00000154217	-1.8378189	3.85E-10
ENSG00000154258	1.24340196	0.02420395
ENSG00000154309	1.32035433	0.0057216
ENSG00000154380	-1.3298724	1.02E-11
ENSG00000154556	1.60858033	2.84E-07
ENSG00000154734	-1.9750012	4.63E-07
ENSG00000155034	1.30706231	1.43E-05
ENSG00000155287	1.21526907	0.000998
ENSG00000155330	-1.2340189	0.01009812
ENSG00000155849	-1.3337368	4.53E-13

Gene ID	FC 4HG - 8HG	adj p-value 4HG - 8HG
ENSG00000155868	-1.2661302	0.00133595
ENSG00000155975	-1.2014795	0.0005531
ENSG00000156030	1.31037098	7.88E-06
ENSG00000156049	-1.346438	0.00643518
ENSG00000156273	-1.2762826	0.00032535
ENSG00000156313	1.35362019	3.64E-07
ENSG00000156671	-1.2651824	0.00089847
ENSG00000156675	1.34630952	0.00718351
ENSG00000157111	1.45036014	3.21E-07
ENSG00000157216	-1.262805	9.48E-07
ENSG00000157240	-1.4946415	2.25E-05
ENSG00000157404	2.86237015	1.93E-11
ENSG00000157456	-1.3110168	5.63E-05
ENSG00000157483	-1.3043519	4.18E-09
ENSG00000157514	1.24199061	0.00966701
ENSG00000157554	1.20471713	2.23E-08
ENSG00000157557	1.35124967	1.34E-11
ENSG00000157570	-1.2976832	3.08E-08
ENSG00000157613	-1.9576298	1.60E-06
ENSG00000157617	1.39894237	3.91E-09
ENSG00000157933	-1.2601775	2.03E-09
ENSG00000158106	1.30856618	0.04447275
ENSG00000158402	-1.2520738	0.00809045
ENSG00000158458	1.23187191	0.02420319
ENSG00000158683	1.29905975	0.01885377
ENSG00000158805	1.2110549	0.0063682
ENSG00000158856	-1.210836	0.00253746
ENSG00000158859	-1.2123978	0.00836824
ENSG00000158966	-1.5356886	1.07E-09
ENSG00000159259	1.38535379	2.24E-05
ENSG00000159261	-1.4005176	0.00086979
ENSG00000159733	2.25821115	6.64E-11

Gene ID	FC 4HG - 8HG	adj p-value 4HG - 8HG
ENSG00000160062	-1.2552265	0.02073711
ENSG00000160113	1.29554937	8.55E-06
ENSG00000160117	1.38754494	0.0005531
ENSG00000160172	1.21361952	0.02207712
ENSG00000160179	1.25637972	0.0244052
ENSG00000160193	-1.2369755	1.07E-05
ENSG00000160200	1.21730569	0.03061542
ENSG00000160271	1.37895955	2.2302E-05
ENSG00000160293	1.21061828	9.17E-06
ENSG00000160685	1.36268509	4.57E-08
ENSG00000160703	1.34714908	6.48E-06
ENSG00000160712	1.39930041	0.00020848
ENSG00000160796	1.22484411	0.0005494
ENSG00000160838	1.27671156	0.00091359
ENSG00000160949	1.39385518	3.68E-05
ENSG00000161653	1.72892574	1.40E-07
ENSG00000161791	-1.2208941	8.61E-08
ENSG00000161835	-1.3173982	9.35E-05
ENSG00000161888	-1.2050722	0.01293528
ENSG00000162066	1.21674552	0.00825767
ENSG00000162073	1.50032353	1.31E-10
ENSG00000162139	-1.2420617	6.20E-05
ENSG00000162194	1.29037284	0.04647825
ENSG00000162298	1.2395639	3.15E-05
ENSG00000162367	1.22571456	1.52E-07
ENSG00000162390	-1.2637334	0.01165399
ENSG00000162407	-2.8890953	2.37E-15
ENSG00000162520	1.21044771	0.00130517
ENSG00000162522	1.6337161	4.53E-13
ENSG00000162542	1.2142073	0.02756554
ENSG00000162636	-1.6028189	1.91E-07
ENSG00000162692	-2.7889284	1.98E-12
ENSG00000162772	-1.7689425	0.01821159
ENSG00000162783	1.34734566	7.42E-07
ENSG00000162817	1.39347827	8.04E-08
ENSG00000162944	1.40830562	0.01028551
ENSG00000163013	1.22509675	0.02079103
ENSG00000163072	1.28395823	0.03262837
ENSG00000163132	-1.351652	0.0004485
ENSG00000163171	1.91593975	1.59E-16
ENSG00000163219	1.55872808	0.00359821
ENSG00000163235	-1.9936604	2.09E-09
ENSG00000163251	1.48108237	5.09E-05
ENSG00000163257	-1.3101283	4.93E-06
ENSG00000163297	-1.3210006	3.30E-07
ENSG00000163462	1.24007585	0.04645204
ENSG00000163491	1.45552006	0.00239743
ENSG00000163637	-1.6579887	2.01E-09
ENSG00000163638	1.47045028	1.99E-07
ENSG00000163661	1.44326843	1.57E-06
ENSG00000163686	1.34652471	0.00016809
ENSG00000163788	1.20873314	0.00013912
ENSG00000163918	1.24734828	0.01939785
ENSG00000163935	-1.4180482	0.00076519
ENSG00000164010	1.24910064	0.00041819
ENSG00000164024	-1.2046396	4.55E-05
ENSG00000164045	1.28889346	0.00057105
ENSG00000164080	-1.2619592	1.86E-09
ENSG00000164093	-1.2112857	7.17E-05
ENSG00000164142	1.2597246	0.00044527
ENSG00000164197	-1.3440788	0.00179902
ENSG00000164211	-1.384009	0.01145504
ENSG00000164236	1.3235347	0.00071431
ENSG00000164296	-1.2335204	0.04862376
ENSG00000164323	1.2446404	0.00318536
ENSG00000164484	-1.2252897	0.0008532
ENSG00000164506	-1.2515905	0.00010945
ENSG00000164512	-1.5938598	2.69E-05
ENSG00000164532	-1.2591293	0.00584742
ENSG00000164543	1.23294241	0.00468774
ENSG00000164611	-1.2791995	2.21E-06
ENSG00000164649	1.21865068	7.49E-08
ENSG00000164663	1.22015749	0.02924452
ENSG00000164683	-1.8563074	1.24E-06
ENSG00000164684	-2.3220804	1.31E-13
ENSG00000164736	-1.2130211	0.00061358
ENSG00000164741	1.20189327	9.74E-06
ENSG00000164758	-1.2025324	0.04758704
ENSG00000164823	-1.5822836	5.37E-09
ENSG00000164867	1.42610099	5.92E-08
ENSG00000165029	-1.3605691	0.01474952
ENSG00000165030	-1.280682	0.00457667
ENSG00000165113	1.22278649	0.0210188
ENSG00000165244	1.74789113	1.06E-08
ENSG00000165338	-1.3454736	0.01054489
ENSG00000165494	1.48893907	1.46E-11
ENSG00000165626	-1.2060013	0.00648156
ENSG00000165671	1.23575543	4.57E-06
ENSG00000165724	1.20067773	0.0019851
ENSG00000165732	-1.2660026	1.03E-05
ENSG00000165757	1.21112765	1.36E-08
ENSG00000165801	-1.4363116	0.00021802
ENSG00000165891	-1.5389145	1.74E-09
ENSG00000166068	-1.3555008	5.62E-07
ENSG00000166140	1.20750568	0.01256522
ENSG00000166169	1.25635663	0.00253375

Gene ID	FC 4HG - 8HG	adj p-value 4HG - 8HG
ENSG00000166289	1.64305251	4.57E-09
ENSG00000166398	-1.3824654	3.68E-08
ENSG00000166401	-1.4337677	6.47E-13
ENSG00000166436	1.33288425	0.00418166
ENSG00000166446	-1.4085879	2.86E-12
ENSG00000166471	-1.4061458	7.34E-06
ENSG00000166741	-1.2624495	1.64E-06
ENSG00000166831	-1.2052436	0.02642088
ENSG00000166833	-1.6534826	5.82E-12
ENSG00000166851	-1.2623377	0.01032873
ENSG00000166886	-1.3020158	0.00273725
ENSG00000166900	-1.2962428	1.71E-08
ENSG00000166924	1.40756392	0.00105336
ENSG00000166925	1.62227933	4.28E-12
ENSG00000166938	1.26898557	8.15E-06
ENSG00000166963	1.43512909	0.0004222
ENSG00000166971	1.20265632	0.00148937
ENSG00000167081	-1.3890666	3.92E-09
ENSG00000167106	1.27040124	0.00044129
ENSG00000167258	-1.2428755	5.61E-09
ENSG00000167378	-1.2617978	5.08E-09
ENSG00000167513	1.50755808	0.00002853
ENSG00000167536	1.49140437	0.00015739
ENSG00000167562	-1.4447322	7.44E-06
ENSG00000167600	1.46661134	0.00023329
ENSG00000167657	-1.2095544	4.81E-06
ENSG00000167670	1.39471163	0.00030611
ENSG00000167702	1.35501235	0.01647482
ENSG00000167716	1.3120842	0.00218727
ENSG00000167772	1.32493059	0.01492168
ENSG00000167861	1.20615819	0.01480751
ENSG00000167962	1.22182423	0.00037908

Gene ID	FC 4HG - 8HG	adj p-value 4HG - 8HG
ENSG00000168016	1.69171049	4.89E-11
ENSG00000168209	1.8096636	6.37E-07
ENSG00000168234	-1.2659964	0.00413158
ENSG00000168386	-1.4110695	0.0004631
ENSG00000168461	-1.5326973	3.57E-13
ENSG00000168497	1.74977319	2.38E-15
ENSG00000168575	-1.217356	6.58E-05
ENSG00000168672	1.56906924	1.83E-08
ENSG00000168769	-1.3375912	4.87E-10
ENSG00000168792	1.55248231	1.61E-09
ENSG00000168827	-1.2165912	7.16E-07
ENSG00000168874	-1.7244124	5.76E-07
ENSG00000168916	-1.2254571	0.00408993
ENSG00000169083	-1.3045632	2.97E-06
ENSG00000169184	1.65274963	1.32E-05
ENSG00000169220	1.23205556	0.04223866
ENSG00000169242	1.77495674	3.825E-06
ENSG00000169247	1.45668797	0.0004511
ENSG00000169252	1.43352506	0.00107902
ENSG00000169255	1.26602396	0.0017092
ENSG00000169429	-2.8082089	1.94E-07
ENSG00000169554	-1.4668486	3.78E-14
ENSG00000169629	-1.3698938	0.00072906
ENSG00000169679	-1.2774336	0.00138233
ENSG00000169715	1.23186474	0.00457161
ENSG00000169744	1.36909851	1.98E-08
ENSG00000169760	1.38520949	3.61E-05
ENSG00000169762	-1.2133796	0.01937232
ENSG00000169871	1.23876448	4.68E-09
ENSG00000169891	-1.3455782	0.00684001
ENSG00000170264	1.2226191	0.01689924
ENSG00000170293	-1.574966	3.41E-07

Gene ID	FC 4HG - 8HG	adj p-value 4HG - 8HG
ENSG00000170312	-1.4142229	0.00091775
ENSG00000170540	-1.2730964	0.03482399
ENSG00000170775	-1.4975224	1.50E-10
ENSG00000170921	-1.4287775	1.00E-06
ENSG00000171017	1.22867545	0.04447789
ENSG00000171115	1.21444053	0.00016151
ENSG00000171132	-1.2549448	2.61E-05
ENSG00000171163	1.33245623	0.02387057
ENSG00000171223	-1.2039726	0.00656528
ENSG00000171227	1.69111199	3.52E-08
ENSG00000171295	-1.258578	0.00169359
ENSG00000171310	-1.7503414	1.47E-10
ENSG00000171316	-1.5828002	1.20E-11
ENSG00000171345	1.24208776	0.01372421
ENSG00000171435	1.61465114	2.63E-10
ENSG00000171448	-1.3439703	0.0035892
ENSG00000171488	-1.2043446	0.0158048
ENSG00000171552	1.3484324	1.18E-08
ENSG00000171604	-1.7771539	4.89E-07
ENSG00000171617	-1.5745924	2.61E-09
ENSG00000171621	1.42566482	0.00317413
ENSG00000171813	1.24937364	0.01554297
ENSG00000171940	-1.3200855	5.00E-07
ENSG00000172081	1.2917747	9.01E-09
ENSG00000172175	-1.2342927	0.00071604
ENSG00000172349	1.21651132	0.04915814
ENSG00000172578	-1.2548234	9.74E-07
ENSG00000172602	-1.7861504	7.09E-07
ENSG00000172613	1.31639467	0.00022065
ENSG00000172716	1.27783862	7.43E-11
ENSG00000172738	-1.3935705	0.00380057
ENSG00000172765	1.32069979	2.27E-09

Gene ID	FC 4HG - 8HG	adj p-value 4HG - 8HG
ENSG00000172780	1.20288856	0.04660537
ENSG00000172889	1.26168463	0.00024531
ENSG00000173040	1.24653449	0.0072668
ENSG00000173068	1.44987706	3.03E-10
ENSG00000173193	-1.2072891	0.00704945
ENSG00000173530	1.53549384	2.18E-12
ENSG00000173614	1.34368837	0.00128933
ENSG00000173681	1.27523363	0.01215792
ENSG00000173868	-1.2755568	8.32E-05
ENSG00000173875	-1.2230893	0.00045055
ENSG00000173894	1.29480836	0.00027093
ENSG00000174004	1.4836994	0.00014428
ENSG00000174306	1.31819759	4.37E-08
ENSG00000174327	1.20627444	0.02266303
ENSG00000174456	-1.237052	0.00687658
ENSG00000174791	1.53651029	2.73E-06
ENSG00000174796	-1.2620438	0.03922792
ENSG00000174804	-1.2146223	1.31E-07
ENSG00000174951	1.22892045	0.0081817
ENSG00000175040	-1.3198982	6.73E-06
ENSG00000175063	-1.2422659	0.02091301
ENSG00000175105	-1.3641511	0.02622723
ENSG00000175264	1.30867293	2.50E-05
ENSG00000175274	-1.2281292	0.00057177
ENSG00000175305	1.65437592	4.79E-06
ENSG00000175544	1.38141524	0.00011773
ENSG00000175591	1.51441337	4.64E-05
ENSG00000175643	1.25937803	0.00958888
ENSG00000175662	1.22375587	6.41E-06
ENSG00000175745	1.26275094	6.46E-06
ENSG00000175746	-1.2931294	0.00461255
ENSG00000176102	1.39649059	3.09E-07

Gene ID	FC 4HG - 8HG	adj p-value 4HG - 8HG
ENSG00000176155	1.40464545	0.00093766
ENSG00000176170	-1.6108477	1.37E-11
ENSG00000176438	1.61898999	9.81E-12
ENSG00000176714	-1.2476184	0.03847526
ENSG00000176749	1.74583698	6.97E-09
ENSG00000176834	-1.2223103	2.94E-06
ENSG00000176845	-1.2653712	0.00821401
ENSG00000176907	-2.5327318	5.38E-11
ENSG00000177000	1.43364365	7.00E-07
ENSG00000177076	1.24999995	0.01676152
ENSG00000177303	1.26547581	4.29E-06
ENSG00000177374	1.64252035	9.37E-07
ENSG00000177383	1.31466325	2.96E-05
ENSG00000177606	1.2362268	1.46E-06
ENSG00000177679	-1.8214483	2.73E-08
ENSG00000177706	-1.2979908	1.19E-09
ENSG00000177732	1.32398968	5.90E-06
ENSG00000177842	1.25396916	0.02086693
ENSG00000177854	1.23039508	0.04383526
ENSG00000177943	1.30920326	0.02872741
ENSG00000178038	1.58650417	3.50E-07
ENSG00000178150	-1.3131803	0.02442449
ENSG00000178401	-1.347921	0.00697267
ENSG00000178409	-1.4689061	8.21E-10
ENSG00000178695	1.33242018	1.84E-06
ENSG00000178718	1.35958231	1.04E-07
ENSG00000178726	1.52564521	1.32E-08
ENSG00000178904	1.20078361	0.04488652
ENSG00000178951	1.21030914	2.63E-06
ENSG00000178971	1.27476685	0.00013978
ENSG00000179219	-1.3075361	2.05E-06
ENSG00000179456	1.43197505	5.55E-10

Gene ID	FC 4HG - 8HG	adj p-value 4HG - 8HG
ENSG00000179532	1.65154182	1.06E-07
ENSG00000179546	1.25993254	0.03764799
ENSG00000179588	-1.2890577	0.00020815
ENSG00000179598	1.44063079	4.63E-06
ENSG00000179630	-1.2840632	0.02926552
ENSG00000179912	1.21025516	2.63E-05
ENSG00000179979	1.26068193	0.02814128
ENSG00000180448	2.05361862	1.03E-08
ENSG00000180694	-1.2940519	0.00048055
ENSG00000180730	-1.381013	1.55E-05
ENSG00000180758	1.23063888	0.0136695
ENSG00000180884	1.3959877	1.63E-05
ENSG00000180902	1.27941909	0.00166274
ENSG00000180938	-1.3038838	0.00117175
ENSG00000180998	-2.0653946	1.47E-08
ENSG00000181035	1.59346329	9.58E-06
ENSG00000181409	1.35726206	0.00984056
ENSG00000181444	1.67742661	1.68E-07
ENSG00000181450	-1.3146071	0.02376825
ENSG00000181472	-1.6919937	1.01E-08
ENSG00000181541	-1.7041567	5.07E-08
ENSG00000181588	-1.2162949	1.35E-06
ENSG00000181751	-1.2250592	0.00987549
ENSG00000181754	1.29825553	0.01314324
ENSG00000181896	-1.2536551	0.00052528
ENSG00000181938	1.27685875	0.00402117
ENSG00000182022	1.38821997	8.84E-12
ENSG00000182141	-1.3175876	0.00922626
ENSG00000182197	-1.5949495	1.01E-21
ENSG00000182253	-1.3751543	4.57E-05
ENSG00000182378	1.26941391	0.00895443
ENSG00000182568	-1.4646722	0.00045467

Gene ID	FC 4HG - 8HG	adj p-value 4HG - 8HG
ENSG00000182621	1.31332755	0.00340745
ENSG00000182685	1.24278272	0.03081519
ENSG00000182704	-1.2303064	0.00024745
ENSG00000182749	1.23774996	0.00174913
ENSG00000182796	1.27968273	0.0197702
ENSG00000182901	1.22733929	0.0376456
ENSG00000183044	1.36896843	0.00126093
ENSG00000183054	-1.3621657	0.00691254
ENSG00000183090	-1.2232375	0.00864256
ENSG00000183208	1.42417026	0.00014927
ENSG00000183287	1.29532668	0.00373776
ENSG00000183337	1.26542054	0.03526029
ENSG00000183578	-1.2261704	0.00116522
ENSG00000183621	1.31297805	3.81E-07
ENSG00000183691	-3.095408	8.70E-14
ENSG00000183779	1.416103	6.2594E-05
ENSG00000183808	-1.2918569	0.00020094
ENSG00000183826	1.25052781	6.12E-07
ENSG00000183856	-1.2728121	0.00149443
ENSG00000184083	1.23677957	0.00091576
ENSG00000184208	1.61384412	3.65E-05
ENSG00000184226	-1.6819269	1.48E-07
ENSG00000184271	1.28780909	0.00866764
ENSG00000184304	-1.5424199	2.06E-11
ENSG00000184307	-1.8436936	2.73E-07
ENSG00000184371	-1.2674029	0.00353628
ENSG00000184465	1.27763317	0.01290701
ENSG00000184992	1.25596721	0.00045045
ENSG00000185015	-1.2396836	0.02166729
ENSG00000185022	1.43938459	3.97E-09
ENSG00000185033	1.39169715	3.19E-05
ENSG00000185112	1.82033963	1.35E-15

Gene ID	FC 4HG - 8HG	adj p-value 4HG - 8HG
ENSG00000185215	1.2525182	0.00214886
ENSG00000185269	-1.5475567	3.20E-08
ENSG00000185352	1.63761612	8.38E-07
ENSG00000185361	1.2508178	0.0006354
ENSG00000185453	1.35576874	0.00039066
ENSG00000185483	-1.3294706	0.00050797
ENSG00000185551	1.29419793	2.46E-08
ENSG00000185614	1.33930365	0.00843042
ENSG00000185670	-1.2621601	0.01129181
ENSG00000185728	-1.2668715	7.04E-06
ENSG00000185909	1.22836999	0.03574028
ENSG00000186354	1.31745967	0.01267963
ENSG00000186432	-1.223098	0.00049642
ENSG00000186480	-1.7113971	5.50E-08
ENSG00000186532	1.25595414	2.25E-05
ENSG00000186567	1.47352965	8.52E-07
ENSG00000186635	1.23000541	3.55E-05
ENSG00000186812	-1.2987316	0.01690069
ENSG00000186814	1.21617798	0.00262923
ENSG00000186918	1.59378095	8.46E-08
ENSG00000186951	1.475668	2.73E-07
ENSG00000187555	-1.2441956	2.15E-09
ENSG00000187605	1.33349506	1.11E-07
ENSG00000187634	-1.6036852	5.00E-05
ENSG00000187764	1.52200543	1.25E-06
ENSG00000187800	1.38365843	2.31E-10
ENSG00000187994	1.32108078	0.01448451
ENSG00000188042	1.89229389	1.83E-08
ENSG00000188177	1.27095009	0.01414032
ENSG00000188315	1.36173347	0.00034879
ENSG00000188428	-1.2482462	0.02245288
ENSG00000188549	1.5460822	3.65E-08

Gene ID	FC 4HG - 8HG	adj p-value 4HG - 8HG
ENSG00000188559	1.2003304	0.00031424
ENSG00000188566	1.2375729	0.0043078
ENSG00000188596	1.53754206	6.93E-06
ENSG00000188811	1.23649658	0.03260048
ENSG00000188827	1.2558441	0.00030307
ENSG00000189057	1.78060992	3.61E-06
ENSG00000189067	-1.2509582	0.04112144
ENSG00000189079	-1.3161905	6.64E-08
ENSG00000189120	1.30962315	0.0010475
ENSG00000189221	1.33829865	8.95E-05
ENSG00000189241	-1.2001011	4.76E-09
ENSG00000189337	1.37038063	0.00917721
ENSG00000196072	-1.2083718	0.00597491
ENSG00000196083	-1.3110218	0.00037331
ENSG00000196110	-1.4376288	0.00014341
ENSG00000196372	1.22908587	0.03565281
ENSG00000196378	-1.3144056	0.00476442
ENSG00000196387	-1.2360481	0.02491442
ENSG00000196449	-1.3079453	1.23E-07
ENSG00000196468	-1.405239	0.00078455
ENSG00000196576	1.22927581	1.99E-05
ENSG00000196584	1.32292109	0.00638325
ENSG00000196700	1.32706081	3.58E-10
ENSG00000196730	1.26052821	1.75E-08
ENSG00000196781	1.36686398	5.59E-11
ENSG00000196782	1.61765766	3.26E-15
ENSG00000196792	-1.235788	0.00014002
ENSG00000196814	-1.224954	0.00862806
ENSG00000196843	-1.2150112	0.0278796
ENSG00000196876	1.48575356	6.80E-07
ENSG00000196922	1.2432513	0.00233724
ENSG00000197119	1.42031383	4.13E-06

Gene ID	FC 4HG - 8HG	adj p-value 4HG - 8HG
ENSG00000197256	1.43517668	3.79E-13
ENSG00000197355	1.2787461	0.00021515
ENSG00000197375	1.28094005	0.00749131
ENSG00000197380	-1.7601474	2.07E-06
ENSG00000197442	-1.3766185	1.28E-06
ENSG00000197461	-1.3008992	0.00050989
ENSG00000197496	1.29860582	0.00050192
ENSG00000197608	-1.2569149	0.0077897
ENSG00000197822	1.25503155	0.03752335
ENSG00000197852	1.34872702	6.15E-07
ENSG00000197857	-1.207029	0.03244397
ENSG00000197905	1.30836647	8.83E-07
ENSG00000197927	-1.2303929	0.04890652
ENSG00000197928	-1.3277485	0.00012355
ENSG00000197948	1.20035919	0.00690668
ENSG00000197971	1.29310759	0.00054319
ENSG00000198018	-1.4078122	1.66E-08
ENSG00000198042	-1.2140251	5.67E-05
ENSG00000198056	1.27443597	0.01741004
ENSG00000198060	-1.2184325	0.00016806
ENSG00000198089	1.20689425	0.00664017
ENSG00000198108	-3.199415	1.66E-15
ENSG00000198205	-1.2909927	5.86E-05
ENSG00000198208	1.4904549	5.81E-05
ENSG00000198342	-1.2556532	0.01797424
ENSG00000198346	-1.243099	0.01436461
ENSG00000198355	1.68185986	1.99E-12
ENSG00000198369	1.27999078	2.6843E-05
ENSG00000198393	-1.2644168	0.01087576
ENSG00000198435	1.27202829	0.01096534
ENSG00000198466	-1.2072231	1.35E-05
ENSG00000198483	1.30369943	0.03882011

Gene ID	FC 4HG - 8HG	adj p-value 4HG - 8HG
ENSG00000198538	-1.2148792	0.00719907
ENSG00000198585	1.22574059	0.00063341
ENSG00000198604	-1.3030592	1.42E-06
ENSG00000198742	-1.3006341	9.08E-10
ENSG00000198753	1.29410095	0.00381514
ENSG00000198774	-1.4320123	0.00028252
ENSG00000198805	-1.3009277	2.77E-10
ENSG00000198846	1.32393893	0.00854433
ENSG00000198901	-1.2999803	5.91E-05
ENSG00000198933	1.25917881	0.00029631
ENSG00000203485	1.29326987	5.44E-07
ENSG00000203709	1.43837606	0.00636706
ENSG00000203727	-1.2462374	0.02309087
ENSG00000203883	1.43344239	0.00146211
ENSG00000203896	1.34815579	0.00714419
ENSG00000204103	1.27196241	0.01881844
ENSG00000204131	1.29092951	7.19E-07
ENSG00000204152	-1.2391846	0.00210255
ENSG00000204217	-1.4960197	5.60E-08
ENSG00000204282	1.24524038	0.03196639
ENSG00000204406	1.49900011	1.21E-08
ENSG00000204442	1.25873099	0.00878255
ENSG00000204604	-1.2831439	1.26E-05
ENSG00000204634	1.41487424	7.18E-09
ENSG00000204682	-1.3008326	0.03551904
ENSG00000204776	1.43905094	4.07E-05
ENSG00000204991	1.33437028	0.00015821
ENSG00000205177	1.55681118	8.92E-06
ENSG00000205269	1.69334382	5.02E-05
ENSG00000205356	1.30002301	2.65E-07
ENSG00000205659	1.2545716	0.002503
ENSG00000205683	1.56505726	1.63E-09

Gene ID	FC 4HG - 8HG	adj p-value 4HG - 8HG
ENSG00000206538	-1.7027596	5.76E-09
ENSG00000206560	1.28031907	4.37E-07
ENSG00000211455	-1.738058	3.92E-08
ENSG00000212766	1.21111654	0.0022837
ENSG00000213064	-1.2107793	0.00615563
ENSG00000213096	-1.3421999	0.01145481
ENSG00000213160	1.32142951	0.0138218
ENSG00000213203	1.38964259	8.79E-06
ENSG00000213398	1.27536817	0.01448733
ENSG00000213462	1.25527304	0.03969894
ENSG00000213626	-1.5780269	0.00001136
ENSG00000213694	1.49788307	2.48E-12
ENSG00000213903	1.34552248	0.00754434
ENSG00000214941	1.22348482	0.00627911
ENSG00000214944	1.59334568	8.89E-12
ENSG00000215012	1.40040538	1.13E-06
ENSG00000215458	1.28974961	0.03129514
ENSG00000215788	1.34155028	0.00820347
ENSG00000223749	1.38629405	0.00282417
ENSG00000224051	1.26539287	0.00016597
ENSG00000224531	-1.2595786	0.00077504
ENSG00000224870	-1.207483	0.00959733
ENSG00000224982	-1.5000369	8.34E-05
ENSG00000225264	-1.284956	0.00361308
ENSG00000225828	1.43140915	0.00582912
ENSG00000225968	-2.2707507	3.98E-10
ENSG00000227158	1.21916707	0.02003249
ENSG00000229372	1.46504129	0.00030095
ENSG00000229729	-1.2739617	0.01161043
ENSG00000229953	1.27143135	0.01894004
ENSG00000230266	1.29352866	0.00828069
ENSG00000230438	2.14585975	7.31E-11

Gene ID	FC 4HG - 8HG	adj p-value 4HG - 8HG
ENSG00000230487	1.3868098	0.00053739
ENSG00000231290	-1.298529	0.01601891
ENSG00000232434	1.34590753	0.00160958
ENSG00000232527	1.24714065	0.00293673
ENSG00000233117	1.34414054	0.00913688
ENSG00000233251	1.36924598	4.17E-05
ENSG00000234409	1.32685498	0.0157049
ENSG00000235257	1.35204213	8.28E-05
ENSG00000235770	1.48580348	1.18E-09
ENSG00000236901	1.31689486	0.00336495
ENSG00000237036	-1.2198677	0.02540283
ENSG00000237440	-1.403314	9.89E-05
ENSG00000237870	-1.3397909	0.00362087
ENSG00000239305	-1.3454833	7.87E-06
ENSG00000239445	-1.2207065	0.02047158
ENSG00000239887	1.21628159	0.02374152
ENSG00000241852	1.21555532	0.04064626
ENSG00000241978	1.22923657	0.01038411
ENSG00000244462	-1.3169198	2.52E-07
ENSG00000245025	1.29783679	0.00041698
ENSG00000246922	1.27961376	0.03259806
ENSG00000248610	-1.203556	1.92E-06
ENSG00000249242	1.26187057	0.00748579
ENSG00000249279	1.30625567	0.00592284
ENSG00000249992	-1.3953852	1.94E-06
ENSG00000250317	1.21038118	0.0144871
ENSG00000250474	1.25196587	0.01688476
ENSG00000250899	1.47164671	6.65E-09
ENSG00000251314	1.27508355	0.00423334
ENSG00000251348	-1.2423542	0.01654367
ENSG00000251493	-1.4359454	0.0011446
ENSG00000253250	-1.3101104	0.00986043

Gene ID	FC 4HG - 8HG	adj p-value 4HG - 8HG
ENSG00000253738	-1.2601857	0.01078917
ENSG00000253958	-1.3365207	0.00097421
ENSG00000254004	-1.2779135	0.0020554
ENSG00000254087	-1.5628392	8.22E-14
ENSG00000254726	-1.2694099	0.0001886
ENSG00000255112	-1.2949872	1.64E-07
ENSG00000255583	-1.2547992	0.01127297
ENSG00000255624	1.25437297	0.01805975
ENSG00000256663	1.29361353	0.02961972
ENSG00000257219	1.20439878	0.00139277
ENSG00000257923	-1.282608	3.58E-07
ENSG00000258441	1.62075829	9.59E-08
ENSG00000258634	1.22537625	0.0052111
ENSG00000259071	1.32139675	0.00973832
ENSG00000260231	-1.3225956	0.00080253
ENSG00000260401	1.32331147	0.0442547
ENSG00000260461	1.21583457	0.04258124
ENSG00000260628	1.31990513	0.00813047
ENSG00000260804	1.25698877	0.00088134
ENSG00000260941	1.28199174	0.00177482
ENSG00000261221	1.20234876	0.00138996
ENSG00000261253	1.22687536	0.03207773
ENSG00000261286	1.25160446	0.04815469
ENSG00000261377	1.2623863	0.00659484
ENSG00000261468	1.38054192	0.0093128
ENSG00000261490	1.39932729	0.01605541
ENSG00000261534	1.36548574	0.0115431
ENSG00000261572	-1.4092001	0.00026298
ENSG00000262580	1.40483464	0.0002026
ENSG00000262601	-1.4115837	0.00066254
ENSG00000264112	1.41773241	0.03659769
ENSG00000265972	-1.6292772	3.09E-09

Gene ID	FC 4HG - 8HG	adj p-value 4HG - 8HG
ENSG00000266208	1.23673677	0.04210517
ENSG00000267045	-1.3845829	2.56E-05
ENSG00000267100	-1.23358	0.01271805
ENSG00000267317	1.30593376	0.00764499
ENSG00000268279	1.21040099	0.02650537
ENSG00000269019	1.2297068	0.03550129
ENSG00000269343	-1.2584787	0.00078662
ENSG00000269973	1.34004805	0.00317784
ENSG00000270157	-1.2550199	0.02564358
ENSG00000270504	1.2535811	0.00105317
ENSG00000271122	1.23533778	0.01316981
ENSG00000271430	1.33589708	0.00169454
ENSG00000272269	-1.3187523	0.00026812
ENSG00000272398	-1.2721195	0.00234452
ENSG00000272405	1.58266941	0.00041051
ENSG00000272447	-1.2342463	0.00827484
ENSG00000272886	-1.2445833	1.93E-06
ENSG00000272914	1.28337071	0.00691591
ENSG00000273329	1.3226228	0.04454005
ENSG00000273780	1.3152307	0.00012501
ENSG00000274995	-1.4415541	0.00495067
ENSG00000275160	1.26570433	0.00954
ENSG00000275322	1.3420005	0.00214021
ENSG00000275832	1.34701386	0.00029974
ENSG00000275993	1.33470274	0.00386049
ENSG00000276043	1.45947106	3.16E-08
ENSG00000276293	1.29483691	2.51E-05
ENSG00000276836	1.35284304	0.00661407
ENSG00000277152	1.27831502	0.04662741
ENSG00000277400	1.20744453	0.02192877
ENSG00000278133	-1.2372332	0.04179687
ENSG00000278600	1.25852015	0.03659218

Gene ID	FC 4HG - 8HG	adj p-value 4HG - 8HG
ENSG00000279117	1.22248286	0.00016036
ENSG00000279207	1.54523733	1.68E-07
ENSG00000279457	1.28652081	0.00423637
ENSG00000279692	-1.2295932	0.04618779
ENSG00000279821	1.30876814	0.00992348
ENSG00000279822	1.44840873	8.99E-06
ENSG00000280187	-1.3034172	0.01347576
ENSG00000280355	1.35320866	0.01475952
ENSG00000281930	-1.2212878	0.02973847

Supplementary Table 4d. Differentially expressed genes between 8hr high glucose and 24hr high glucose samples

Ensembl gene IDs, Fold change (8HG-24HG) and FDR adjusted p-values of DEGs

Gene ID	FC 8HG - 24HG	adj p-value 8HG - 24HG
ENSG00000000003	-1.2081734	0.0032628
ENSG00000000971	-1.2083779	0.02248497
ENSG00000004799	-1.5711013	2.63E-06
ENSG00000005187	-1.3113457	0.00089471
ENSG00000005882	-1.2207379	0.03883114
ENSG00000006042	-1.2359309	3.51E-08
ENSG00000006831	1.24156491	1.29E-07
ENSG00000008294	1.26157348	1.73E-10
ENSG00000008441	-1.2409271	2.78E-08
ENSG00000010361	-1.2101042	0.02074039
ENSG00000010818	-1.4252757	0.00029323
ENSG00000011007	1.20768806	5.60E-08
ENSG00000011523	-1.2175082	1.4702E-05
ENSG00000013588	-1.6032278	3.46E-09
ENSG00000019144	1.36094471	1.02E-08
ENSG00000021355	-1.2533469	0.00081843
ENSG00000021645	1.24753759	0.02196193
ENSG00000023171	-1.285223	0.00658135
ENSG00000023445	-1.5703238	0.00977953
ENSG00000033327	-1.2038462	3.69E-06
ENSG00000033867	1.29159066	0.00120888
ENSG00000037280	-1.312379	0.00516671
ENSG00000038295	1.31529944	0.00443589
ENSG00000048052	1.88619246	1.70E-06
ENSG00000049759	1.37711995	8.74E-07
ENSG00000051128	-1.2868833	1.55E-06
ENSG00000055813	1.22282373	0.04005258
ENSG00000056972	-1.3047588	0.001257
ENSG00000057294	1.34666014	0.01332987
ENSG00000058091	1.23412171	0.00057751
ENSG00000059378	-1.3397809	4.87E-07
ENSG00000063180	-1.2314059	0.00994744

Gene ID	FC 8HG - 24HG	adj p-value 8HG - 24HG
ENSG00000064042	-1.8935998	1.58E-09
ENSG00000064651	1.29117741	0.01638415
ENSG00000064692	-1.4438956	0.01640347
ENSG00000064989	1.32587861	0.01602734
ENSG00000065054	1.25266759	0.00081351
ENSG00000065057	-1.2210482	0.00147665
ENSG00000065618	-1.2359256	0.03060976
ENSG00000065809	1.24400019	6.17E-08
ENSG00000066735	1.28287744	0.00046435
ENSG00000067221	1.29483395	0.00024415
ENSG00000067798	1.20181514	2.54E-05
ENSG00000068078	1.34436485	0.00021572
ENSG00000068366	1.20825222	0.00047282
ENSG00000069122	-1.6076718	1.51E-06
ENSG00000069482	1.26251783	0.01645147
ENSG00000070495	1.22124937	0.00038205
ENSG00000070759	-1.3026959	0.00188008
ENSG00000071073	1.21781653	0.00109332
ENSG00000071246	-1.2971016	0.01514258
ENSG00000071575	-1.2264653	0.01896602
ENSG00000072786	1.2072052	4.69E-05
ENSG00000073849	-1.3523144	3.28E-09
ENSG00000073921	1.28449937	0.00026241
ENSG00000074370	-1.3849646	0.03599459
ENSG00000074527	-1.6254671	1.54E-07
ENSG00000074590	1.2695258	0.00977024
ENSG00000075711	-1.289306	1.12E-05
ENSG00000076356	1.22576775	4.49E-06
ENSG00000076928	1.24589541	9.68E-07
ENSG00000077092	-1.8141713	9.15E-10
ENSG00000077348	-1.2129008	0.02239615
ENSG00000078018	1.79114308	4.36E-08

Gene ID	FC 8HG - 24HG	adj p-value 8HG - 24HG
ENSG00000078081	-1.281147	0.00346069
ENSG00000079102	-1.2330855	0.04596357
ENSG00000079335	1.28059488	0.00019094
ENSG00000079385	-1.5392226	5.49E-06
ENSG00000079691	-1.4058439	0.02080737
ENSG00000081181	-1.2391709	0.00369097
ENSG00000081913	-1.2387685	0.04900968
ENSG00000082512	-1.3147695	0.00578207
ENSG00000084444	-1.2308889	0.0009302
ENSG00000084731	-1.2558319	8.91E-07
ENSG00000086696	-1.4763169	0.00018407
ENSG00000087303	1.24035366	0.0002135
ENSG00000088280	-1.3246424	5.25E-05
ENSG00000088367	-1.3785806	0.0005157
ENSG00000088448	-1.2385485	0.01245473
ENSG00000088986	1.25575477	4.02E-10
ENSG00000089041	-1.3726292	0.01168587
ENSG00000089127	-1.752559	0.00020231
ENSG00000089351	-1.2364216	0.00071862
ENSG00000090238	-1.6135943	2.15E-09
ENSG00000090339	-1.4566705	0.00659816
ENSG00000091129	1.34504038	5.45E-07
ENSG00000091409	1.33180047	8.37E-08
ENSG00000091656	1.31430083	0.00025477
ENSG00000091879	1.46309576	8.45E-10
ENSG00000091972	-1.4310951	6.84E-05
ENSG00000092108	1.25109121	0.00274415
ENSG00000092871	-1.2197633	0.01482
ENSG00000092969	-1.3940395	0.0129342
ENSG00000095539	-1.2156674	0.02759791
ENSG00000096654	-1.2467511	0.03739206
ENSG00000099204	-1.4621635	1.96E-09

Gene ID	FC 8HG - 24HG	adj p-value 8HG - 24HG
ENSG00000099256	-1.224403	0.01340139
ENSG00000099889	-1.3061414	0.00022554
ENSG00000099937	-1.6770086	7.16E-06
ENSG00000100027	-1.37573	0.00687511
ENSG00000100100	-2.3105885	2.89E-12
ENSG00000100292	-1.3213371	0.00011769
ENSG00000100311	1.26013378	0.0389783
ENSG00000100321	-1.3806859	0.00595423
ENSG00000100342	-1.2919063	0.00105488
ENSG00000100350	-1.2686026	0.00013679
ENSG00000100439	-1.2821752	0.00278329
ENSG00000100697	1.26312739	1.82E-05
ENSG00000100784	-1.5029158	1.5502E-05
ENSG00000100906	-1.2281924	0.00340858
ENSG00000100918	-1.2273297	0.00612147
ENSG00000101096	-1.5079714	9.80E-09
ENSG00000101188	-1.2997755	5.71E-05
ENSG00000101311	-1.2796186	0.0058922
ENSG00000101331	1.34736805	1.67E-05
ENSG00000101333	-1.30083	0.01307727
ENSG00000101445	-1.4237153	2.86E-06
ENSG00000101670	1.26940421	0.00030432
ENSG00000101871	-1.4192289	1.42E-09
ENSG00000102010	-1.3898661	2.90E-07
ENSG00000102024	1.21210529	1.19E-05
ENSG00000102032	-1.2818926	0.04150449
ENSG00000102302	-1.2487067	0.00026549
ENSG00000102452	-1.297666	0.0026825
ENSG00000102755	1.41852752	7.95E-09
ENSG00000102802	-1.8046753	1.05E-08
ENSG00000102908	-1.2613782	0.0018837
ENSG00000102934	-1.2825925	0.01452298

Gene ID	FC 8HG - 24HG	adj p-value 8HG - 24HG
ENSG00000103021	1.29783398	0.01841288
ENSG00000103024	-1.2607562	5.59E-05
ENSG00000103034	-1.4388223	7.95E-07
ENSG00000103647	-1.3060711	6.00E-06
ENSG00000104081	-1.5205842	0.02425451
ENSG00000104361	-1.256355	0.00473058
ENSG00000104447	-1.2188022	0.02033008
ENSG00000104450	-1.2064953	0.04982778
ENSG00000104497	-1.2734243	0.0020237
ENSG00000104611	-1.2664693	0.01709751
ENSG00000105088	2.00420423	9.05E-13
ENSG00000105122	-1.2680537	0.01063433
ENSG00000105255	-1.2228331	0.01004816
ENSG00000105270	-1.5258218	4.80E-05
ENSG00000105327	-1.3251736	1.90E-05
ENSG00000105810	1.37496563	3.60E-05
ENSG00000105825	1.50302308	4.27E-14
ENSG00000105856	-1.2316564	0.00159499
ENSG00000105967	-1.2493837	0.04597223
ENSG00000105996	-1.2291594	0.04849369
ENSG00000105997	-1.3913511	0.00649909
ENSG00000106004	-1.2827189	0.00366109
ENSG00000106049	-1.2475508	0.00016046
ENSG00000106537	1.36103516	2.01E-06
ENSG00000106560	-1.2478877	0.0058189
ENSG00000106780	1.21514819	0.00250163
ENSG00000106852	-1.4721814	2.21E-05
ENSG00000106853	-1.2006526	1.37E-06
ENSG00000107201	-1.2313067	0.01254788
ENSG00000107249	-1.3842643	0.00120032
ENSG00000107438	-1.2173406	5.89E-06
ENSG00000107562	3.94669166	2.04E-21

Gene ID	FC 8HG - 24HG	adj p-value 8HG - 24HG
ENSG00000107738	-1.2303	0.02925064
ENSG00000107758	1.2087619	0.0164837
ENSG00000108001	-1.2636836	0.00261276
ENSG00000108306	-1.2481178	0.00505532
ENSG00000108448	-1.3444761	0.00010511
ENSG00000108551	1.55640985	7.99E-08
ENSG00000108556	1.30736236	0.00487512
ENSG00000108622	-1.2775429	1.34E-09
ENSG00000108691	-2.2304769	1.85E-09
ENSG00000108984	-2.1882293	6.75E-11
ENSG00000109046	-1.2604176	0.00084691
ENSG00000109738	-1.2952271	0.01013796
ENSG00000109906	1.83003932	5.37E-08
ENSG00000110002	-1.2109245	0.01027428
ENSG00000110042	-1.3311388	8.22E-06
ENSG00000110090	-1.2928402	1.35E-12
ENSG00000110092	1.37867653	3.71E-10
ENSG00000110455	-1.3078074	0.00421689
ENSG00000110799	-1.2499122	8.66E-05
ENSG00000110888	1.24778329	0.0062069
ENSG00000110900	-1.2852508	0.00337355
ENSG00000110987	-1.206004	0.02602398
ENSG00000111110	-1.374363	0.04586877
ENSG00000111145	-1.2763971	1.12E-09
ENSG00000111335	-1.5141226	0.00019706
ENSG00000111341	1.32561917	4.92E-06
ENSG00000111653	-1.2926736	0.00040981
ENSG00000111799	-1.3259773	0.01244445
ENSG00000111801	-1.4052984	0.00043981
ENSG00000111880	1.2388952	0.00120236
ENSG00000112406	-1.2090074	0.01985451
ENSG00000112414	-1.344888	0.00019917

Gene ID	FC 8HG - 24HG	adj p-value 8HG - 24HG
ENSG00000112425	1.36104165	0.00330296
ENSG00000113319	1.29467847	3.20E-05
ENSG00000113552	-1.2473018	1.02E-08
ENSG00000114126	-1.3964217	1.49E-07
ENSG00000114166	-1.2463024	0.01086334
ENSG00000114480	1.3807202	1.16E-10
ENSG00000114698	-1.6128526	6.39E-09
ENSG00000114735	1.23212993	0.0051441
ENSG00000114796	-1.6033159	5.73E-08
ENSG00000115091	1.20976554	9.89E-07
ENSG00000115109	-1.2620283	0.0003108
ENSG00000115163	-1.2108078	0.0486593
ENSG00000115267	-1.5379959	0.00141059
ENSG00000115415	-1.3090169	1.54E-06
ENSG00000115525	-1.4508306	0.0003666
ENSG00000115556	-1.2784523	0.01673213
ENSG00000115884	-1.3140827	0.00276124
ENSG00000116096	-1.2175189	0.00289036
ENSG00000116133	1.25925642	1.26E-07
ENSG00000116191	-1.2707765	0.01441625
ENSG00000116473	1.24527051	1.05E-07
ENSG00000116711	2.13715119	5.65E-09
ENSG00000116852	-1.2227989	0.00827774
ENSG00000116990	-1.2900046	1.79E-05
ENSG00000117226	-1.2059557	1.34E-05
ENSG00000117394	1.25340865	1.39E-05
ENSG00000117399	-1.2089721	0.01948408
ENSG00000117595	1.52183938	0.00290489
ENSG00000118407	-1.6755655	3.41E-07
ENSG00000118503	-1.6296726	0.0000104
ENSG00000118777	-1.3268914	0.00220711
ENSG00000118946	2.38487737	8.07E-06

Gene ID	FC 8HG - 24HG	adj p-value 8HG - 24HG
ENSG00000119138	1.20940883	0.00127091
ENSG00000119227	-1.2391043	0.01424129
ENSG00000119326	-1.2414606	0.00064846
ENSG00000119431	-1.2356188	0.03604838
ENSG00000119686	-1.3557496	0.00021501
ENSG00000119714	-1.2423005	0.0219322
ENSG00000119917	-1.3628635	0.00667074
ENSG00000119922	-1.3177047	0.00029825
ENSG00000119938	-1.2450323	0.04549726
ENSG00000119950	-1.4858276	0.00010144
ENSG00000120093	-1.2449938	0.00216053
ENSG00000120217	1.3651889	8.97E-06
ENSG00000120437	1.380053	4.21E-07
ENSG00000120549	-1.5914981	1.68E-06
ENSG00000120910	1.22712903	0.0001464
ENSG00000121210	-1.2690889	9.94E-07
ENSG00000121858	-2.6806031	1.55E-11
ENSG00000122378	-1.2642608	8.31E-09
ENSG00000122644	-1.2709637	0.00460706
ENSG00000122778	-1.270931	3.34E-05
ENSG00000122861	-1.512091	5.12E-07
ENSG00000123689	-1.3785325	0.01253918
ENSG00000123908	1.2395695	8.47E-08
ENSG00000123977	-1.3905502	0.00218023
ENSG00000124145	-1.2502288	0.00018214
ENSG00000124225	-1.3280383	0.00627724
ENSG00000124406	-1.2571774	0.02427422
ENSG00000124444	-1.2388284	0.00724341
ENSG00000124508	-1.3882868	1.06E-05
ENSG00000124575	-1.3325949	0.00044073
ENSG00000124610	-1.5235564	7.71E-07
ENSG00000124635	-1.2962581	0.01589183

Gene ID	FC 8HG - 24HG	adj p-value 8HG - 24HG
ENSG00000124766	-1.3592369	5.11E-08
ENSG00000124875	-1.5275572	0.00026165
ENSG00000125347	-1.2808851	0.02896024
ENSG00000125657	-1.2934759	0.00951397
ENSG00000125772	1.23302637	0.01881928
ENSG00000125848	-1.3628456	0.03648711
ENSG00000125864	-1.2730141	0.00804606
ENSG00000125945	-1.2206036	0.00163038
ENSG00000125966	1.49798885	0.00010868
ENSG00000126016	-1.2933822	0.01601081
ENSG00000126351	-1.316291	5.27E-07
ENSG00000126368	-1.2927592	0.00932376
ENSG00000126785	-1.3062598	8.27E-11
ENSG00000127220	-1.3310054	0.00237404
ENSG00000127314	1.23029337	0.0434494
ENSG00000127528	1.40648149	0.0003307
ENSG00000128203	-1.3092115	0.00314325
ENSG00000128284	-1.2219622	0.02270329
ENSG00000128294	-1.2575816	7.08E-10
ENSG00000128567	1.81914712	1.73E-17
ENSG00000128602	-1.3196695	0.00377954
ENSG00000128656	-1.370964	2.04E-09
ENSG00000128815	-1.2994849	0.0028395
ENSG00000128849	-1.3996379	2.13E-08
ENSG00000129116	-1.2981068	0.00074206
ENSG00000129538	-1.2765724	0.00102348
ENSG00000129675	-1.217956	0.02255371
ENSG00000130052	1.27991994	8.12E-05
ENSG00000130270	-1.2228502	0.01748795
ENSG00000130300	1.58438944	0.00069447
ENSG00000130305	1.43241492	0.0021403
ENSG00000130513	-1.5316685	7.30E-12

Gene ID	FC 8HG - 24HG	adj p-value 8HG - 24HG
ENSG00000130775	-1.2117384	0.02455722
ENSG00000130827	1.22894955	7.8022E-05
ENSG00000130830	-1.229985	3.00E-05
ENSG00000130956	1.29495858	2.74E-05
ENSG00000130988	-1.2670229	0.02103226
ENSG00000131016	1.88238346	7.04E-19
ENSG00000131069	1.20698815	0.02584384
ENSG00000131389	-1.3235484	1.09E-11
ENSG00000131409	1.9101716	0.00046455
ENSG00000131634	1.26367332	0.00037952
ENSG00000131669	-1.3201251	6.51E-07
ENSG00000131831	1.26055657	0.01705951
ENSG00000131943	1.20024362	0.00192366
ENSG00000132003	-1.2798893	0.00059532
ENSG00000132510	-1.4185775	9.55E-07
ENSG00000132530	-1.436719	0.00597088
ENSG00000132622	-1.2513387	0.0067238
ENSG00000132846	-1.2354743	0.0004102
ENSG00000132879	-1.2058004	0.03881603
ENSG00000132970	-1.2757107	2.06E-07
ENSG00000133056	-1.4478943	1.91E-06
ENSG00000133216	-1.3274886	3.04E-09
ENSG00000133424	1.55303183	2.79E-06
ENSG00000133639	-1.4324501	1.48E-09
ENSG00000133657	1.34403729	0.00600741
ENSG00000133800	-1.5895564	3.46E-05
ENSG00000133874	-1.3551031	6.10E-05
ENSG00000134138	-1.2179552	0.00741013
ENSG00000134291	-1.2046823	0.00385359
ENSG00000134363	1.36120722	0.00025113
ENSG00000134508	-1.8675515	9.79E-12
ENSG00000134531	1.3309733	4.92E-16

Gene ID	FC 8HG - 24HG	adj p-value 8HG - 24HG
ENSG00000134574	-1.2602237	6.08E-07
ENSG00000134668	1.21842205	0.02157251
ENSG00000134775	-1.466357	0.02316256
ENSG00000134909	-1.2478588	0.00227078
ENSG00000134986	-1.2873114	5.12E-05
ENSG00000135074	-1.3348804	2.31E-05
ENSG00000135312	1.24577676	0.00020041
ENSG00000135363	-1.245708	0.00582583
ENSG00000135365	-1.2256846	0.0001895
ENSG00000135643	-1.351062	0.00182439
ENSG00000135709	1.20755776	0.00642846
ENSG00000135842	-1.2097129	0.00180799
ENSG00000135914	-1.6957823	1.59E-05
ENSG00000135919	1.21801403	4.49E-05
ENSG00000135929	-1.2604697	0.00409423
ENSG00000136111	1.24242083	4.97E-07
ENSG00000136205	1.28044733	2.03E-05
ENSG00000136231	1.24649449	2.49E-06
ENSG00000136305	-1.3448452	0.00382211
ENSG00000136383	-1.2484201	0.00065898
ENSG00000136717	-1.2922682	2.64E-05
ENSG00000136826	1.2647464	0.02279422
ENSG00000136859	-1.2585612	9.79E-06
ENSG00000137094	-1.3131883	9.20E-06
ENSG00000137198	-1.2180854	0.00495217
ENSG00000137266	-1.2657827	0.00169453
ENSG00000137463	-1.2597414	0.04211846
ENSG00000137486	-1.245406	8.10E-05
ENSG00000137507	-1.3262311	0.00010254
ENSG00000137752	-1.4559942	3.35E-05
ENSG00000137801	1.21815849	1.21E-05
ENSG00000137831	1.35099031	0.00012007

Gene ID	FC 8HG - 24HG	adj p-value 8HG - 24HG
ENSG00000137877	1.40739662	0.00228984
ENSG00000137941	-1.2188688	0.03509106
ENSG00000137960	-1.3120292	0.03046232
ENSG00000137965	-1.3207275	0.00779363
ENSG00000138185	-1.4505657	0.000272
ENSG00000138356	-1.274352	0.00306722
ENSG00000138411	1.2311639	8.88E-05
ENSG00000138448	1.24860953	0.00291819
ENSG00000138449	-1.2875107	0.00045302
ENSG00000138496	-1.4290792	3.16E-05
ENSG00000138640	-1.2479077	0.01725639
ENSG00000138764	-1.6100469	1.36E-05
ENSG00000138767	1.26108901	0.00077836
ENSG00000138771	1.618888	3.56E-08
ENSG00000138794	-1.2390872	0.00014345
ENSG00000138796	-1.2062004	0.01662598
ENSG00000139112	-1.2806866	1.20E-05
ENSG00000139187	-1.5444002	2.24E-05
ENSG00000139211	-1.5651158	1.75E-06
ENSG00000139354	-1.2267145	0.01607626
ENSG00000139428	1.20487793	4.54E-05
ENSG00000139508	-1.2943039	0.03067539
ENSG00000139517	-1.2413418	0.00027726
ENSG00000139899	-1.2974025	0.02080737
ENSG00000139946	-1.4621743	7.81E-05
ENSG00000140092	1.28850895	7.62E-05
ENSG00000140263	-1.2549585	6.72E-06
ENSG00000140416	1.23992887	7.00E-08
ENSG00000140465	-1.3059128	0.01729967
ENSG00000140564	1.26505117	2.96E-06
ENSG00000140678	1.20383369	0.04706275
ENSG00000140873	-1.967966	2.90E-12

Gene ID	FC 8HG - 24HG	adj p-value 8HG - 24HG
ENSG00000141068	-1.2441042	0.00021515
ENSG00000141179	1.24185052	6.69E-06
ENSG00000141338	-1.3986614	0.00048991
ENSG00000141447	-1.2126781	0.00767254
ENSG00000141505	-1.2376142	0.04144931
ENSG00000141574	-1.4284557	0.00041691
ENSG00000141668	2.1205446	2.11E-06
ENSG00000141858	1.23293663	0.00011144
ENSG00000141934	-1.4906805	1.87E-06
ENSG00000142661	1.40687368	0.00165131
ENSG00000143067	-1.2304172	0.02323172
ENSG00000143127	1.20362071	0.00101186
ENSG00000143153	1.23196635	0.00037309
ENSG00000143324	-1.2066909	0.00055029
ENSG00000143344	1.34344613	1.01E-07
ENSG00000143387	-1.3605097	0.00029304
ENSG00000143409	-1.2316186	0.01548044
ENSG00000143434	-1.4154592	1.72E-06
ENSG00000143443	-1.2360412	0.02054617
ENSG00000143603	-1.3109312	0.00794551
ENSG00000143819	-1.5014993	3.00E-08
ENSG00000143850	-1.2036505	0.02350458
ENSG00000143891	-1.2884711	0.00231532
ENSG00000144063	2.02290993	8.06E-17
ENSG00000144366	1.37826822	7.05E-05
ENSG00000144476	-1.3949816	0.00177758
ENSG00000144560	-1.3201853	1.09E-08
ENSG00000144589	1.22107929	0.00027158
ENSG00000144642	1.29262519	9.79E-05
ENSG00000144644	1.35361704	0.01447758
ENSG00000144730	-1.243448	0.04197982
ENSG00000144821	-1.2049393	0.0070544

Gene ID	FC 8HG - 24HG	adj p-value 8HG - 24HG
ENSG00000144824	-1.2198846	0.00252752
ENSG00000145365	-1.3092073	0.00073185
ENSG00000145431	-1.2908042	0.0219178
ENSG00000145736	1.27611211	0.02361607
ENSG00000145990	1.24480478	0.00016434
ENSG00000146021	-1.2848434	0.00200398
ENSG00000146083	-1.2860817	3.93E-06
ENSG00000146243	-1.2413785	0.03235792
ENSG00000146278	-1.3260607	8.0019E-05
ENSG00000146386	1.27605126	1.13E-06
ENSG00000146409	-1.2086762	0.02084936
ENSG00000146859	-1.4748226	5.5473E-05
ENSG00000147027	1.23735411	0.01013844
ENSG00000147113	-1.58166	1.74E-07
ENSG00000147202	1.39917434	0.00022728
ENSG00000147408	-1.5120169	4.40E-06
ENSG00000148803	1.21766688	0.0022385
ENSG00000148841	-1.2320129	2.70E-06
ENSG00000148842	-1.3888417	0.00104147
ENSG00000149212	-1.59749	7.01E-10
ENSG00000149269	-1.522068	4.33E-05
ENSG00000149573	-1.3150193	3.41E-05
ENSG00000149582	-1.3124392	3.39E-05
ENSG00000149591	1.24655842	0.0073418
ENSG00000149798	1.22189418	7.26E-06
ENSG00000149948	1.34165896	0.00016165
ENSG00000150593	-1.2314606	0.00200737
ENSG00000151012	1.27179137	0.01816368
ENSG00000151023	1.62559873	2.09E-05
ENSG00000151117	-1.4564218	0.00069376
ENSG00000151322	1.25602372	0.00926349
ENSG00000151388	-1.4442997	2.03E-06

Gene ID	FC 8HG - 24HG	adj p-value 8HG - 24HG
ENSG00000151414	1.25266939	0.02054099
ENSG00000151500	-1.2151304	7.75E-06
ENSG00000151692	-1.4540877	0.00064343
ENSG00000151917	-1.2084316	0.0450957
ENSG00000152284	-1.3384578	1.53E-05
ENSG00000152601	1.37592287	2.14E-07
ENSG00000152661	1.23555656	3.12E-06
ENSG00000152689	1.24870514	0.01084296
ENSG00000152953	-1.2525976	0.00083974
ENSG00000153094	-1.6727941	8.72E-06
ENSG00000153208	-1.2562019	0.00317993
ENSG00000153291	-1.2433638	0.00936209
ENSG00000153707	1.67818772	6.21E-06
ENSG00000153956	1.2478294	0.00082987
ENSG00000153993	1.30040787	0.00786911
ENSG00000154217	-1.3966876	0.00012484
ENSG00000154258	-1.2926859	0.00548897
ENSG00000154262	-1.293847	0.03374454
ENSG00000154358	-1.2390318	0.02648219
ENSG00000154556	1.40534894	0.00014438
ENSG00000154930	-1.7043716	1.78E-08
ENSG00000155011	1.36319004	0.00356309
ENSG00000155111	-1.2488975	0.01605881
ENSG00000155324	-1.3000396	0.00075828
ENSG00000155657	1.4299182	0.00337832
ENSG00000155755	-1.2361941	0.00015944
ENSG00000155849	-1.2252808	6.29E-09
ENSG00000155966	1.33272264	0.00191088
ENSG00000156049	-1.4824478	0.00019304
ENSG00000156265	1.50720395	2.55E-06
ENSG00000156313	1.35202381	3.96E-07
ENSG00000156463	-1.4750785	0.00020238

Gene ID	FC 8HG - 24HG	adj p-value 8HG - 24HG
ENSG00000156504	1.21849542	0.00010981
ENSG00000156587	-1.2859064	3.66E-05
ENSG00000156711	-1.354524	0.01035041
ENSG00000157483	1.25745003	1.38E-07
ENSG00000157570	-1.2322983	3.63E-06
ENSG00000157617	-1.2035513	0.0004438
ENSG00000157693	-1.3684182	0.00018773
ENSG00000157827	-1.2436863	6.19E-06
ENSG00000158270	-1.3890645	0.00018034
ENSG00000158373	-1.3126574	0.00211518
ENSG00000158406	-1.3051489	0.00081375
ENSG00000158555	-1.4655767	6.52E-12
ENSG00000158715	-1.2353347	0.0044282
ENSG00000159388	-1.2312169	8.95E-05
ENSG00000159733	1.30363665	0.02428807
ENSG00000159761	-1.2287015	0.00863641
ENSG00000159899	-1.2499895	0.00014489
ENSG00000160013	-1.3176263	0.02107477
ENSG00000160145	-1.3480095	0.00078695
ENSG00000160179	-1.7505765	2.09E-08
ENSG00000160219	-1.2407607	0.03349348
ENSG00000160285	1.22006298	0.00178582
ENSG00000160752	1.22674059	4.32E-06
ENSG00000161011	-1.3181551	8.60E-08
ENSG00000161542	-1.2343662	8.39E-09
ENSG00000161682	-1.4701601	1.0645E-05
ENSG00000161714	-1.2178201	0.00300353
ENSG00000161921	-1.2360169	0.00392261
ENSG00000162174	-1.3204953	1.15E-07
ENSG00000162337	-1.2355392	8.43E-05
ENSG00000162407	-1.3541065	0.00333528
ENSG00000162434	1.51047731	6.01E-16

Gene ID	FC 8HG - 24HG	adj p-value 8HG - 24HG
ENSG00000162496	-2.2031077	5.24E-13
ENSG00000162551	1.32385266	0.00097234
ENSG00000162576	-1.2605254	0.00051862
ENSG00000162599	-1.320981	4.51E-05
ENSG00000162614	1.22198892	0.00883352
ENSG00000162636	-1.2474223	0.01719478
ENSG00000162639	-1.2076079	0.02310143
ENSG00000162733	-1.2826649	3.51E-10
ENSG00000162924	-1.2526585	0.02472624
ENSG00000163050	-1.3858772	0.0006862
ENSG00000163110	1.35985487	7.04E-13
ENSG00000163171	1.26197022	4.55E-05
ENSG00000163346	-1.3359356	2.6864E-05
ENSG00000163412	-1.2732635	0.00743126
ENSG00000163545	-1.3422154	0.03167049
ENSG00000163554	-1.4604225	0.00052226
ENSG00000163565	-1.2178408	0.00034075
ENSG00000163637	-1.6440083	3.04E-09
ENSG00000163739	-1.2896848	0.04061247
ENSG00000163751	-1.3631708	0.0148236
ENSG00000163840	-1.2303259	0.00814308
ENSG00000163945	1.21932728	0.03930324
ENSG00000164038	1.21416867	8.79E-05
ENSG00000164056	1.36255597	0.01828527
ENSG00000164070	-1.2554539	0.00098084
ENSG00000164142	1.28489796	0.00013286
ENSG00000164161	1.26678507	9.28E-06
ENSG00000164237	-1.3108638	4.34E-06
ENSG00000164283	1.36173993	4.91E-05
ENSG00000164284	1.37499135	0.00086338
ENSG00000164342	-1.3294501	0.00927961
ENSG00000164463	-1.2891747	0.00328108

Gene ID	FC 8HG - 24HG	adj p-value 8HG - 24HG
ENSG00000164615	-1.2040329	0.00110396
ENSG00000164649	-1.2044414	2.92E-07
ENSG00000164687	-1.4480933	1.39E-06
ENSG00000164692	-1.3224789	0.01181355
ENSG00000164736	1.32274591	1.10E-06
ENSG00000164849	-2.0752104	3.27E-06
ENSG00000164850	-1.2563421	0.00375151
ENSG00000164932	-1.2037491	0.00197494
ENSG00000164938	-1.2354093	0.018256
ENSG00000164949	-1.2883578	0.03742456
ENSG00000164985	-1.2054298	0.00028389
ENSG00000165029	-3.1471554	3.89E-14
ENSG00000165092	-2.3662458	1.28E-13
ENSG00000165244	1.29171201	0.00665875
ENSG00000165312	-1.3570996	0.00276883
ENSG00000165494	1.22460778	6.19E-05
ENSG00000165626	-1.2345825	0.00175252
ENSG00000165804	-1.2807426	0.01435358
ENSG00000165821	-1.5134723	1.15E-05
ENSG00000165861	-1.201864	0.00021457
ENSG00000165912	-1.3914683	2.14E-06
ENSG00000165959	-1.4964042	9.43E-09
ENSG00000166046	-1.5653607	5.12E-05
ENSG00000166073	1.26061852	9.68E-05
ENSG00000166265	-1.3225463	0.00011846
ENSG00000166313	-1.2515475	3.27E-06
ENSG00000166439	-1.3809092	2.08E-10
ENSG00000166503	1.20490035	0.00160722
ENSG00000166670	1.61151711	1.62E-07
ENSG00000166741	-1.2634142	1.53E-06
ENSG00000166897	-1.2525236	0.02821847
ENSG00000167106	-1.3094115	7.34E-05

Gene ID	FC 8HG - 24HG	adj p-value 8HG - 24HG
ENSG00000167107	-1.3807062	3.32E-05
ENSG00000167191	-1.3617175	0.00093899
ENSG00000167508	1.31539311	0.00012203
ENSG00000167550	-1.2147708	0.04676694
ENSG00000167552	1.26815433	8.63E-07
ENSG00000167680	-1.2149468	0.00051093
ENSG00000167693	-1.3200234	3.15E-09
ENSG00000167767	-1.2271046	0.0018876
ENSG00000167874	-1.4168176	0.00018178
ENSG00000167889	1.33162703	0.02044757
ENSG00000168061	-1.2546715	0.0050803
ENSG00000168234	-1.3746135	7.38E-05
ENSG00000168298	-1.3583574	2.55E-05
ENSG00000168300	-1.3062881	0.00014
ENSG00000168309	-1.3742459	3.95E-06
ENSG00000168374	1.21617871	1.99E-06
ENSG00000168528	1.31444501	1.81E-05
ENSG00000168575	1.37401624	2.97E-09
ENSG00000168672	-1.2822749	0.00103654
ENSG00000168679	-1.3260689	0.00151371
ENSG00000168906	1.33584907	1.64E-13
ENSG00000168916	-1.327141	4.32E-05
ENSG00000168939	-1.2660954	0.03091071
ENSG00000168961	-1.4339189	0.00041492
ENSG00000169083	-1.4481398	1.65E-09
ENSG00000169169	-1.3355077	0.00032901
ENSG00000169174	1.27301171	0.00259117
ENSG00000169242	-1.3526539	0.02158737
ENSG00000169429	-1.5451064	0.03902312
ENSG00000169504	1.28624516	0.00512471
ENSG00000169710	1.35393949	7.31E-07
ENSG00000169740	-1.2676233	0.00023675

Gene ID	FC 8HG - 24HG	adj p-value 8HG - 24HG
ENSG00000169891	-1.3667593	0.00400939
ENSG00000169908	1.21969294	0.00026129
ENSG00000170011	-1.344729	0.00239039
ENSG00000170214	1.31259718	0.01044602
ENSG00000170271	-1.7875764	1.32E-07
ENSG00000170323	-1.3616492	0.03838411
ENSG00000170425	-1.3326931	0.00118318
ENSG00000170456	-1.3611008	6.82E-06
ENSG00000170485	-1.2201465	4.72E-06
ENSG00000170522	1.29443504	6.98E-06
ENSG00000170558	1.24512739	9.04E-08
ENSG00000170915	1.2129129	0.00840198
ENSG00000170921	-1.2255064	0.00569996
ENSG00000171132	-1.3470507	1.14E-07
ENSG00000171246	3.66143102	1.26E-15
ENSG00000171345	-1.2045284	0.0445964
ENSG00000171425	-1.2020233	0.00571782
ENSG00000171444	-1.2540547	1.33E-05
ENSG00000171488	1.2227468	0.00767671
ENSG00000171617	-1.2733907	0.00057954
ENSG00000171843	-1.3197056	0.00244524
ENSG00000171889	1.34721002	0.00097329
ENSG00000172031	-1.4325768	0.00047562
ENSG00000172086	1.28518802	9.58E-05
ENSG00000172183	1.26159336	0.0126512
ENSG00000172572	1.350335	4.74E-07
ENSG00000172731	-1.247654	0.01006393
ENSG00000172738	-1.3821264	0.00499012
ENSG00000172915	-1.3196189	0.00453711
ENSG00000172985	-1.2182395	0.00247564
ENSG00000173166	1.20420718	0.02214716
ENSG00000173193	-1.3806804	2.53E-06

Gene ID	FC 8HG - 24HG	adj p-value 8HG - 24HG
ENSG00000173221	-1.2290844	0.00695367
ENSG00000173511	-1.2582417	2.33E-06
ENSG00000173535	-1.4796486	1.98E-08
ENSG00000173597	1.33919353	0.0118698
ENSG00000173599	-1.2307607	0.00153051
ENSG00000174059	-1.8256611	7.09E-05
ENSG00000174130	-1.230342	0.04893613
ENSG00000174327	1.23005549	0.00961446
ENSG00000174456	-1.2856022	0.00102999
ENSG00000174501	1.63837203	4.82E-05
ENSG00000174516	-1.289314	0.00994729
ENSG00000174684	-1.2323344	0.00199774
ENSG00000174718	-1.3104221	0.00583003
ENSG00000174738	-1.2022915	0.01710821
ENSG00000174951	1.20883244	0.01740794
ENSG00000175274	-1.2185889	0.00095363
ENSG00000175348	1.21033701	0.00001373
ENSG00000175471	-1.2161161	0.01216076
ENSG00000175544	1.2037447	0.04767685
ENSG00000175556	-1.3348542	0.00364214
ENSG00000175906	-1.2160915	0.03588243
ENSG00000176046	-1.4818612	0.00067409
ENSG00000176102	1.25397366	0.00034404
ENSG00000176170	-1.2349174	0.00049316
ENSG00000176371	-1.3212683	0.0023157
ENSG00000176428	-1.2645297	0.02017304
ENSG00000176438	1.4667385	4.38E-09
ENSG00000176658	1.25029048	3.86E-06
ENSG00000176907	-1.3375934	0.03080585
ENSG00000177119	1.25066673	1.34E-05
ENSG00000177169	-1.2844969	2.39E-06
ENSG00000177303	1.25739302	7.26E-06

Gene ID	FC 8HG - 24HG	adj p-value 8HG - 24HG
ENSG00000177337	-1.3041289	0.0038208
ENSG00000177663	1.21353499	0.00010935
ENSG00000177675	-1.4391906	2.22E-06
ENSG00000177868	-1.2163946	0.00313947
ENSG00000178026	-1.2245167	0.02349654
ENSG00000178409	-1.2352682	0.00019129
ENSG00000178445	-1.2133451	0.02059943
ENSG00000178685	-1.2458994	0.00411505
ENSG00000178922	-1.380859	4.05E-07
ENSG00000179044	1.73214346	2.06E-08
ENSG00000179094	1.22440043	0.00304046
ENSG00000179242	-1.3040697	0.00068546
ENSG00000179314	-1.3181582	0.00019043
ENSG00000179387	1.3128131	0.01629958
ENSG00000179403	1.33682062	0.02022867
ENSG00000179532	1.21994971	0.04927172
ENSG00000179598	1.23620637	0.01003497
ENSG00000179715	-1.318355	0.02565178
ENSG00000179820	1.36622686	2.45E-12
ENSG00000179855	-1.3017434	0.02707447
ENSG00000180263	-1.2849337	3.35E-05
ENSG00000180304	-1.2395257	2.05E-07
ENSG00000180340	-1.5417476	1.93E-05
ENSG00000180448	1.31862074	0.0304641
ENSG00000180530	-1.3160969	6.03E-05
ENSG00000180573	-1.2373466	0.00256385
ENSG00000180596	-1.2113419	0.02781498
ENSG00000180611	-1.3419319	0.00261856
ENSG00000180758	1.21108975	0.02640447
ENSG00000180938	-1.2405547	0.01128378
ENSG00000181350	-1.2936624	0.01364477
ENSG00000181634	-2.1685963	9.51E-07

Gene ID	FC 8HG - 24HG	adj p-value 8HG - 24HG
ENSG00000181826	-1.2132312	6.38E-09
ENSG00000182013	-1.3840918	4.74E-05
ENSG00000182165	-1.3672133	4.37E-05
ENSG00000182325	1.2423118	0.02190331
ENSG00000182489	1.33153798	0.00236035
ENSG00000182568	-1.7057874	1.42E-06
ENSG00000182575	-1.353274	0.0033924
ENSG00000182621	1.49743037	1.07E-05
ENSG00000182809	1.38937926	1.94E-10
ENSG00000182901	1.25721241	0.01589078
ENSG00000182916	-1.3729921	0.0006238
ENSG00000183023	-1.2791753	0.01825762
ENSG00000183044	1.25691586	0.02934233
ENSG00000183049	1.45855559	0.00016858
ENSG00000183496	-1.626927	1.74E-07
ENSG00000183578	1.22217648	0.00142698
ENSG00000183580	-1.3442679	1.36E-10
ENSG00000183598	-1.3617131	8.78E-05
ENSG00000183615	-1.5409636	0.00021805
ENSG00000184113	-1.5784021	1.26E-08
ENSG00000184260	-1.2269558	0.01234974
ENSG00000184270	-1.2729042	0.03826348
ENSG00000184349	1.24546723	0.01548998
ENSG00000184357	-1.3584299	0.00073386
ENSG00000184371	-1.4816454	1.26E-06
ENSG00000184384	1.21028576	0.00201655
ENSG00000184584	1.34662242	0.00031281
ENSG00000184719	-1.2144842	0.04192766
ENSG00000184897	-1.2737315	6.64E-07
ENSG00000184916	1.31339545	4.52E-06
ENSG00000184988	1.20852616	0.00667035
ENSG00000185015	1.21843826	0.04041413

Gene ID	FC 8HG - 24HG	adj p-value 8HG - 24HG
ENSG00000185070	-1.3903388	6.71E-06
ENSG00000185130	-1.3844751	0.00062822
ENSG00000185352	1.76288129	3.85E-08
ENSG00000185420	-1.2086598	0.00346367
ENSG00000185432	-1.4244741	6.36E-09
ENSG00000185477	-1.4343023	1.44E-06
ENSG00000185483	-1.2132276	0.02951313
ENSG00000185630	-1.5468543	3.01E-06
ENSG00000185753	1.25156902	7.45E-06
ENSG00000185813	1.26765681	6.54E-06
ENSG00000185909	-1.2520418	0.01804352
ENSG00000186281	1.33260911	0.000928
ENSG00000186377	-1.386266	0.00194423
ENSG00000186470	-1.26458	3.82E-05
ENSG00000186479	-1.3851189	8.12E-05
ENSG00000186567	1.51515867	1.92E-07
ENSG00000186642	-1.3075228	0.00094455
ENSG00000186918	-1.2046754	0.04360288
ENSG00000187239	-1.2207076	2.35E-05
ENSG00000187479	-1.3327408	0.02087019
ENSG00000187678	1.29546121	4.24E-05
ENSG00000187764	-1.2374613	0.01855654
ENSG00000187837	-1.334618	0.00022342
ENSG00000187994	1.28314225	0.03476427
ENSG00000188060	-1.2481418	0.01433187
ENSG00000188191	-1.2104683	0.00270358
ENSG00000188596	1.41052129	0.00028841
ENSG00000188827	1.25705071	0.00028491
ENSG00000189056	1.3386962	0.00094712
ENSG00000189060	-1.3386672	1.83E-08
ENSG00000189319	-1.2307731	9.4269E-05
ENSG00000196169	-1.5835922	3.14E-06

Gene ID	FC 8HG - 24HG	adj p-value 8HG - 24HG
ENSG00000196209	-1.3600111	0.00187335
ENSG00000196352	1.27615755	2.6441E-05
ENSG00000196468	-1.2737958	0.02642622
ENSG00000196476	-1.2384617	0.00588799
ENSG00000196569	-1.2429786	0.02034188
ENSG00000196611	1.74065124	2.63E-07
ENSG00000196730	-1.2126358	1.04E-06
ENSG00000196747	-1.2429183	0.0358554
ENSG00000196787	-1.2614171	0.01866944
ENSG00000196812	-1.3549598	0.02164764
ENSG00000196814	-1.3555123	4.25E-05
ENSG00000196866	-1.3119365	0.00060997
ENSG00000196878	-1.384302	0.00800396
ENSG00000197061	-1.2542094	0.01008659
ENSG00000197062	-1.2427253	0.00501968
ENSG00000197093	-1.2797822	0.01794053
ENSG00000197147	1.2960925	0.00141862
ENSG00000197153	-1.3631456	0.00525894
ENSG00000197238	-1.2406383	0.00395235
ENSG00000197261	-1.3812093	0.00173087
ENSG00000197409	-1.3451359	0.00147471
ENSG00000197461	1.35825128	5.07E-05
ENSG00000197555	-1.310976	2.22E-06
ENSG00000197837	-1.3137802	4.46E-05
ENSG00000198108	1.42584182	0.00134958
ENSG00000198720	-1.2896531	2.26E-06
ENSG00000198721	-1.2502724	9.77E-08
ENSG00000198774	-1.2993048	0.01162689
ENSG00000198794	-1.3780748	0.00011048
ENSG00000198795	-1.2339325	2.60E-06
ENSG00000198816	-1.2224837	0.00023213
ENSG00000198844	1.3112094	4.81E-05

Gene ID	FC 8HG - 24HG	adj p-value 8HG - 24HG
ENSG00000198929	-1.4662097	0.00013649
ENSG00000198947	-1.4186928	4.07E-06
ENSG00000198959	-1.5776561	1.61E-14
ENSG00000203727	-1.324705	0.00209465
ENSG00000203814	-1.347438	4.61E-05
ENSG00000204103	-1.2381136	0.0455676
ENSG00000204217	1.25368851	0.00152284
ENSG00000204262	-1.2448093	4.19E-07
ENSG00000204282	1.27604824	0.0136867
ENSG00000204403	-1.2196413	0.00713448
ENSG00000204442	1.70894391	4.27E-09
ENSG00000204634	1.24374501	9.20E-05
ENSG00000204776	1.38488247	0.00023716
ENSG00000205177	1.39181078	0.00088736
ENSG00000205213	-1.2299409	0.01444349
ENSG00000205362	-1.2589623	0.03726824
ENSG00000205476	-1.2321583	0.000228
ENSG00000205978	-1.3736965	1.17E-05
ENSG00000214719	-1.2136451	0.03609915
ENSG00000217128	-1.2080922	0.00168446
ENSG00000221926	-1.2575239	8.98E-06
ENSG00000222041	-1.2418008	0.00011815
ENSG00000222047	-1.2746702	0.02535922
ENSG00000224635	1.22695689	0.0476443
ENSG00000224914	1.27308413	0.0034849
ENSG00000224982	-1.3654069	0.00303457
ENSG00000225177	-1.3059127	0.00404991
ENSG00000225511	-1.2250339	0.00250812
ENSG00000225830	1.24645791	0.00018182
ENSG00000226049	1.22471196	0.00603339
ENSG00000226180	1.23578158	0.01707697
ENSG00000226476	-1.3105201	0.02471183

Gene ID	FC 8HG - 24HG	adj p-value 8HG - 24HG
ENSG00000226808	1.34366363	0.00034137
ENSG00000227158	1.24296279	0.00877931
ENSG00000228794	1.22371414	0.00932592
ENSG00000229644	1.21113793	0.02334005
ENSG00000230266	-1.3580644	0.00119983
ENSG00000230606	1.46898483	0.01248545
ENSG00000231113	-1.2040998	0.03171062
ENSG00000232931	1.94452128	7.30E-06
ENSG00000233117	1.88704303	3.31E-08
ENSG00000233223	-1.2740951	0.01600217
ENSG00000233251	1.23486618	0.00801561
ENSG00000233369	-1.2629181	2.29E-05
ENSG00000233695	-1.2166277	0.04837356
ENSG00000233822	-1.2377087	0.03205382
ENSG00000234409	1.29008896	0.03562553
ENSG00000235109	-1.3442886	0.00079334
ENSG00000235162	1.21868838	0.00013166
ENSG00000235505	-1.3529159	0.01868453
ENSG00000235750	1.31600375	7.72E-06
ENSG00000237424	-1.2690313	0.02747251
ENSG00000237870	-1.3122661	0.00777851
ENSG00000239521	-1.3590149	0.01219397
ENSG00000239887	-1.2744004	0.00312338
ENSG00000240583	-1.2819867	0.01961851
ENSG00000241015	1.23430737	0.00699876
ENSG00000241749	1.29926623	0.00999112
ENSG00000242265	1.37000979	1.15E-10
ENSG00000244716	1.20392754	0.04738758
ENSG00000249464	1.35510097	0.00194195
ENSG00000250067	1.24859253	0.03195985
ENSG00000251580	-1.2162425	0.01780895
ENSG00000253368	1.70757355	3.00E-14

Gene ID	FC 8HG - 24HG	adj p-value 8HG - 24HG
ENSG00000253522	-1.2523883	0.00309989
ENSG00000253552	-1.3779385	0.00103181
ENSG00000253661	1.24701358	0.02573296
ENSG00000254027	-1.2584091	0.0035099
ENSG00000254531	-1.3974195	0.00184983
ENSG00000254726	-1.3949372	4.24E-07
ENSG00000254838	-1.2230869	0.02435808
ENSG00000254858	1.22839727	0.00155325
ENSG00000255690	-1.3003114	0.010724
ENSG00000257219	1.20236219	0.0015599
ENSG00000258441	1.46539544	1.16E-05
ENSG00000259071	1.28041517	0.02692129
ENSG00000260804	1.24131386	0.00179075
ENSG00000260822	-1.2858527	0.00141153
ENSG00000260920	1.23262166	0.02561992
ENSG00000261308	-1.2263048	0.03043126
ENSG00000261468	1.32654644	0.02848672
ENSG00000261490	1.37914064	0.02308599
ENSG00000262001	-1.3342428	0.00025809
ENSG00000266074	-1.2307989	0.0387941
ENSG00000267194	-1.3597439	0.0031832
ENSG00000267508	-1.29513	0.0226957
ENSG00000267583	-1.2506948	0.00013589
ENSG00000270547	-1.7470708	2.41E-07
ENSG00000271737	-1.2186572	0.02092188
ENSG00000272168	-1.3438405	0.00979619
ENSG00000273230	1.33221396	0.02147784
ENSG00000273489	1.33174548	0.03777065
ENSG00000273703	-1.4609275	0.00013497
ENSG00000273802	-1.3628146	0.00020435
ENSG00000273983	-1.2739463	0.01475224
ENSG00000274180	-1.439139	0.00017735
ENSG00000274213	1.24441907	0.02441719
ENSG00000274267	-1.289086	0.01647773
ENSG00000274290	-1.4133097	2.58E-05
ENSG00000274618	-1.2727113	0.03407391
ENSG00000274641	-1.315533	0.00787327
ENSG00000274712	-1.2802429	0.00572943
ENSG00000274750	-1.3588837	0.00058775
ENSG00000274997	-1.3516139	0.00373688
ENSG00000275126	-1.24646	0.02290579
ENSG00000275379	-1.4434341	0.00015167
ENSG00000275713	-1.298407	0.02454753
ENSG00000275714	-1.3386487	5.20E-05
ENSG00000276180	-1.3658871	0.00430756
ENSG00000276368	-1.2888198	0.01369041
ENSG00000276410	-1.3907889	0.00046218
ENSG00000276644	1.29583057	0.01862864
ENSG00000276903	-1.3270751	0.00854934
ENSG00000276966	-1.2032719	0.00192231
ENSG00000277075	-1.3530701	0.00044656
ENSG00000277157	-1.3387929	0.00261504
ENSG00000277224	-1.4250356	0.00014579
ENSG00000277656	-1.3132175	0.00499667
ENSG00000277775	-1.2828898	0.018851
ENSG00000278272	-1.2743109	0.01605881
ENSG00000278463	-1.3292602	0.00222911
ENSG00000278588	-1.4348278	0.00010261
ENSG00000278637	-1.2443791	0.00676589
ENSG00000278705	-1.2920539	0.00088848
ENSG00000278828	-1.3000995	0.00435945
ENSG00000279095	1.22005184	0.00025615
ENSG00000279117	-1.2837723	3.62E-06
ENSG00000279207	1.30209427	0.00105843
ENSG00000279822	1.32225549	0.00078857

Supplementary Table 5. Ingenuity pathway analysis (IPA) -log10(p-values) and z-scores for DEGs of normal and high glucose conditions comparing the 0.5h time point to baseline (0NG)

	NG		HG	
**IPA Pathways**	**logPvalue**	**Zscore**	**logPvalue**	**Zscore**
Protein Kinase A Signaling	1.28	-1.342	3.6	-2.121
Estrogen Receptor Signaling	1.02	2.236	1.38	2.236
Cardiac Hypertrophy Signaling (Enhanced)	1.43	2.449	1.01	2
Cardiac Hypertrophy Signaling	1.56	2	1.01	2
Endothelin-1 Signaling	2.15	2	2.6	2
PI3K Signaling in B Lymphocytes	2.52	2	3.92	1.342
ILK Signaling	2.59	2.449	2.35	2.236
CXCR4 Signaling	2.86	2	2.57	NA
Senescence Pathway	2.87	2.121	2.18	1.633
GNRH Signaling	2.91	2	3.47	2
PPAR Signaling	2.98	-2	3.46	-2
Tumor Microenvironment Pathway	3.87	2.646	2.72	2.236
MIF Regulation of Innate Immunity	3.89	2	4.3	2
HMGB1 Signaling	4	2.449	2.82	2
Regulation Of The Epithelial Mesenchymal Transition By Growth Factors Pathway	4.2	2.121	3.17	1.633
Hepatic Fibrosis Signaling Pathway	4.24	2.111	3.02	2.121
IL-17A Signaling in Gastric Cells	4.38	2	4.8	2
IL-8 Signaling	4.8	2.828	4.72	2.646

Supplementary Table 6. IPA -log10(p-values) and z-scores for DEGs of normal and high glucose conditions comparing the 1h time point to baseline (0NG)

	NG		HG	
**IPA Pathways**	**logPvalue**	**Zscore**	**logPvalue**	**Zscore**
IL-6 Signaling	3.97	2.309	3.34	2.714
Regulation Of The Epithelial Mesenchymal Transition In Development Pathway	3.33	3	2.66	2.828
Regulation Of The Epithelial Mesenchymal Transition By Growth Factors Pathway	3.33	2.673	2.82	3.051
PCP pathway	3.07	2.646	1.69	2.236
Cardiac Hypertrophy Signaling (Enhanced)	2.93	3.838	1.69	3.3
PPAR Signaling	2.9	-3	2.33	-2.121
HMGB1 Signaling	2.81	3	2.78	3
MIF Regulation of Innate Immunity	2.39	2.236	2.38	2.236
Neuroinflammation Signaling Pathway	2.26	2.138	1.85	2.496
IL-8 Signaling	2.12	3.317	2.5	3.464
ERK5 Signaling	2.09	2.449	2.07	2.449
White Adipose Tissue Browning Pathway	2.03	2.121	1.53	2.646
Cardiac Hypertrophy Signaling	1.64	2.53	1.3	3
PDGF Signaling	2.06	1.89	2.58	2.121
ILK Signaling	1.24	2.828	1.92	3.162

Supplementary Table 7. IPA -log10(p-values) and z-scores for DEGs of normal and high glucose conditions comparing the 4h time point to baseline (0NG)

	NG		HG	
**IPA Pathways**	**logPvalue**	**Zscore**	**logPvalue**	**Zscore**
Hepatic Fibrosis Signaling Pathway	5.65	2.794	4.69	3.395
Regulation Of The Epithelial Mesenchymal Transition By Growth Factors Pathway	5.42	2.082	4.97	2.714
Apelin Cardiac Fibroblast Signaling Pathway	5.18	-2.714	3.59	-3.162
Cardiac Hypertrophy Signaling (Enhanced)	4.22	2.769	2.58	3.536
Kinetochore Metaphase Signaling Pathway	4.13	-2.858	2.52	-2.558
Mitotic Roles of Polo-Like Kinase	4.08	-2.887	2.61	-2.333
Cell Cycle Control of Chromosomal Replication	3.72	-3.3	3.69	-3.578
STAT3 Pathway	3.5	3.13	2.93	2.711
HMGB1 Signaling	3.47	2.2	2.82	2.117
Glioma Signaling	3.4	2.324	3.63	2.982
PPAR Signaling	3.2	-2.294	2.17	-1.877
HIF1α Signaling	3.06	2	3.62	2.592
PPARα/RXRα Activation	2.9	-3.138	1.71	-2.502
Apoptosis Signaling	2.14	-2.4	1.59	-1.279
Retinoic acid Mediated Apoptosis Signaling	1.94	-2.714	2.69	-2.673
TREM1 Signaling	1.87	2.309	0.754	1.265
PDGF Signaling	1.86	2.357	2	2.837
NGF Signaling	1.72	2.4	1.45	2.294
NF-κB Activation by Viruses	1.63	2.324	1.92	2.828
Role of NFAT in Cardiac Hypertrophy	1.56	2.646	1.79	3.772
MIF Regulation of Innate Immunity	1.48	2.121	1.09	2.121
Factors Promoting Cardiogenesis in Vertebrates	5.2	1.461	5.34	2.058
BMP signaling pathway	3.1	1.886	3.38	2.4
p70S6K Signaling	2.05	1.46	2.88	2.414
VEGF Family Ligand-Receptor Interactions	2.27	1.5	2.56	2.236
HGF Signaling	2.3	1.606	2.49	2.294
LPS-stimulated MAPK Signaling	2.1	1.213	2.28	2.236
ErbB4 Signaling	1.78	1.732	2.18	2.324
IL-6 Signaling	3.11	1.961	2.02	2.353
PAK Signaling	1.32	1.604	1.99	2.065
Type II Diabetes Mellitus Signaling	1.97	0.728	1.97	2.065
IGF-1 Signaling	0.883	1.941	1.86	2.683
ErbB Signaling	1.55	1.698	1.7	2.683
P2Y Purigenic Receptor Signaling Pathway	0.666	1.698	1.45	2.132
Insulin Secretion Signaling Pathway	0.421	2.2	1.41	3.124
Renin-Angiotensin Signaling	1.13	1.414	1.39	2.558

Supplementary Table 8. IPA -log10(p-values) and z-scores for DEGs of normal and high glucose conditions comparing the 8h time point to baseline (0NG)

	NG		HG	
**IPA Pathways**	**logPvalue**	**Zscore**	**logPvalue**	**Zscore**
Kinetochore Metaphase Signaling Pathway	12.2	-3.78	11.9	-4.017
Mitotic Roles of Polo-Like Kinase	6.37	-2.138	7.66	-1.291
Cell Cycle: G2/M DNA Damage Checkpoint Regulation	4.05	2.309	4.61	1.941
Cardiac Hypertrophy Signaling (Enhanced)	3.58	2.534	5.09	1.54
Hepatic Fibrosis Signaling Pathway	3.15	2.795	5.8	2.949
PI3K/AKT Signaling	2.41	2.309	2.28	1.941
IL-6 Signaling	1.89	2.324	2.13	1.5
Apelin Cardiac Fibroblast Signaling Pathway	1.71	-2.236	1.67	-2.236
STAT3 Pathway	1.7	3	2.27	2.714
NER Pathway	1.64	2.138	2.6	2.5
HOTAIR Regulatory Pathway	1.56	-2	1.78	0.153
PTEN Signaling	1.53	-2.138	1.45	-2.138
Coagulation System	2.83	-1.633	2	-2.236
Cell Cycle: G1/S Checkpoint Regulation	0.959	-3	1.94	-2.309
Actin Nucleation by ARP-WASP Complex	0.58	2.236	1.7	2.828

Supplementary Table 9. IPA -log10(p-values) and z-scores for DEGs of normal and high glucose conditions comparing the 24h time point to baseline (0NG)

	NG		HG	
**IPA Pathways**	**logPvalue**	**Zscore**	**logPvalue**	**Zscore**
Kinetochore Metaphase Signaling Pathway	14.9	-3.78	12.7	-3.53
Mitotic Roles of Polo-Like Kinase	5.75	-2.887	3.19	-2.714
Cell Cycle: G2/M DNA Damage Checkpoint Regulation	4.82	2.714	4.44	2.714
LXR/RXR Activation	3.07	-2.236	2.71	-2.449
Cardiac Hypertrophy Signaling (Enhanced)	2.53	2.268	4.93	3.795
Senescence Pathway	2.34	2.132	2.65	2.646
STAT3 Pathway	2.07	2.236	3.12	2.333
Hepatic Fibrosis Signaling Pathway	1.94	2.711	5.93	3.124
p38 MAPK Signaling	1.56	2.828	0.959	2.828
HMGB1 Signaling	0.824	1.89	2.64	2.496
IL-8 Signaling	0.355	2.333	2.58	2.236
IL-6 Signaling	0.821	2.828	2.48	3.207
Endothelin-1 Signaling	0.631	1.414	1.81	2.138
Acute Phase Response Signaling	0.854	2.646	1.63	2.714
LPS-stimulated MAPK Signaling	0.277	2	1.59	2.333

Supplementary Table 10. IPA -log10(p-values) and z-scores for K-Means late cluster genes

	NG		HG		
IPA Pathways	logPvalue	Zscore	logPvalue	Zscore	Pathway Genes
HMGB1 Signaling	3.66	2	3.48	2.236	CXCL8,IL11,IL6,SELE,VCAM1
Hepatic Fibrosis Signaling Pathway	3.26	2.236	4.19	2.646	BMPR2,CXCL8,FLT1,FZD1,GLI2,IL1RL1,VCAM1,VEGFC
Cardiac Hypertrophy Signaling (Enhanced)	1.92	1	5.78	2.714	BMPR2,CXCL8,FGF5,FGFR3,FZD1,HDAC9,IL11,IL1RL1,IL6,NOTUM,PDE10A
STAT3 Pathway	1.61		3.89	2	BMPR2,FGFR3,FLT1,IL1RL1,TGFA
Tumor Microenvironment Pathway	2.41		3.35	2.236	CXCL12,CXCL8,FGF5,IL6,VEGFC
HIF1α Signaling	2.22		3.05	2.236	CAMK1D,FLT1,IL6,TGFA,VEGFC
Role of NFAT in Cardiac Hypertrophy	2.17		2.97	2	CAMK1D,HDAC9,IL11,IL6,NOTUM
NF-κB Signaling	0.558		2.39	2	BMPR2,FGFR3,FLT1,TGFA
IL-8 Signaling	2.25		2.22	2	CXCL8,FLT1,VCAM1,VEGFC

Supplementary Table 11. IPA -log10(p-values) and z-scores for K-Means 4-hour downregulated cluster genes

	NG		HG		
IPA Pathways	logPvalue	Zscore	logPvalue	Zscore	Pathway Genes
Apelin Endothelial Signaling Pathway	2.06	-2.236	1.39	-2	VCAM1
Cardiac Hypertrophy Signaling (Enhanced)	1.35	-2.887	0.703	-2.449	BMPR2,CXCL8,FGF5,FGFR3,FZD1,HDAC9,IL11,IL1RL1,IL6,NOTUM,PDE10A
Cell Cycle: G2/M DNA Damage Checkpoint Regulation	4.28	0.378	2.65	2	CDK6,HDAC9
Chemokine Signaling	1.65	-2	1.21		CAMK1D,CXCL12
CXCR4 Signaling	0.364		1.39	-2	CXCL12
Cyclins and Cell Cycle Regulation	1.63	2.236	0.221		CDK6,HDAC9
Endothelin-1 Signaling	0.484	-2	1.7	-2.449	NOTUM,PLA2G4A
ErbB Signaling	1.39	-1	1.66	-2	TGFA
ERK5 Signaling	3.22	-2.449	0.71		FOSL1, GAB1, LIF, MAP3K8, MEF2D, RASD1,SGK1
Gα12/13 Signaling	1.77	-2	1.78	-2	F2RL3, JUN, MEF2D, NFKBIA, NFKBIE,PIK3CG, RASD1
Gαq Signaling	0.672		2.04	-2.236	HMOX1, NFATC2, NFKBIA, PIK3C2B, PIK3CG, PLD1
Hepatic Fibrosis Signaling Pathway	2.02	-1.291	1.56	-2.121	BMPR2,CXCL8,FLT1,FZD1,GLI2,IL1RL1,VCAM1,VEGFC
HGF Signaling	2.53	-1.342	2.62	-2	IL6
HIF1α Signaling	1.25	-2.828	1.54	-2.449	CAMK1D,FLT1,IL6,TGFA,VEGFC
IL-15 Production	1.01	-2.236	1.92	-2.236	FGFR3,FLT1,IL6
IL-8 Signaling	1.69	-2.121	2.11	-2.646	CXCL8,FLT1,VCAM1,VEGFC
LPS-stimulated MAPK Signaling	1.59	-2	1.84	-2	JUN, NFKBIA, PIK3C2B, PIK3CG
mTOR Signaling	1.57	0	1.49	-2	VEGFC
Rac Signaling	1.45	-2.236	2.6	-2.236	IQGAP3, ITGB4, JUN, PIK3C2B, PIK3CG, PLD1
Regulation Of The Epithelial Mesenchymal Transition By Growth Factors Pathway	2.81	-3.162	1.21	-2.236	FGF5,FGFR3,IL6
Retinoic acid Mediated Apoptosis Signaling	2.15	-2.236	3.24	-2.236	BMPR2,CDK6,CXCL8,IL6,SMAD6,SMAD9
Senescence Pathway	3.81	-2.5	1.88	-1.414	ATF3,CXCL8,GADD45B,JUN,RASD1,SMAD6
Signaling by Rho Family GTPases	0.398	-2.449	1.6	-2.449	CDC42EP3, CIT,ITGB4, JUN, PIK3C2B, PIK3CG, PLD1
TGF-β Signaling	1.35	-2	0.532		BMPR2,INHBA,SMAD6,SMAD9,ZNF423
Tumor Microenvironment Pathway	3.04	-2.111	1.83	-2.449	CXCL12,CXCL8,FGF5,IL6,VEGFC

Supplementary Table 12. List of K-Means cluster genes in high and normal glucose (Early, Middle, Late, 4-hour downregulated)

K-Means Cluster	Time Point	Condition	Gene ID
Early	0-0.5	NG	ENSG00000114315
Early	0-0.5	NG	ENSG00000120738
Early	0-0.5	NG	ENSG00000122877
Early	0-0.5	NG	ENSG00000123358
Early	0-0.5	NG	ENSG00000125740
Early	0-0.5	NG	ENSG00000170345
Early	0-0.5	NG	ENSG00000171223
Early	0-0.5	NG	ENSG00000179388
Early	0-0.5	HG	ENSG00000114315
Early	0-0.5	HG	ENSG00000122877
Early	0-0.5	HG	ENSG00000123358
Early	0-0.5	HG	ENSG00000124216
Early	0-0.5	HG	ENSG00000125740
Early	0-0.5	HG	ENSG00000128016
Early	0-0.5	HG	ENSG00000144655
Early	0-0.5	HG	ENSG00000155090
Early	0-0.5	HG	ENSG00000160888
Early	0-0.5	HG	ENSG00000162772
Early	0-0.5	HG	ENSG00000170345
Early	0-0.5	HG	ENSG00000171223
Early	0-0.5	HG	ENSG00000179388
Early	0-0.5	HG	ENSG00000184557
Middle	0-1	NG	ENSG00000073756
Middle	0-1	NG	ENSG00000101665
Middle	0-1	NG	ENSG00000115738
Middle	0-1	NG	ENSG00000124216
Middle	0-1	NG	ENSG00000125968
Middle	0-1	NG	ENSG00000128594
Middle	0-1	NG	ENSG00000137834
Middle	0-1	NG	ENSG00000162772
Middle	0-1	NG	ENSG00000164683
Middle	0-1	NG	ENSG00000176907

K-Means Cluster	Time Point	Condition	Gene ID
Middle	0-1	NG	ENSG00000183691
Middle	0-1	HG	ENSG00000005513
Middle	0-1	HG	ENSG00000023445
Middle	0-1	HG	ENSG00000073756
Middle	0-1	HG	ENSG00000099860
Middle	0-1	HG	ENSG00000101665
Middle	0-1	HG	ENSG00000106003
Middle	0-1	HG	ENSG00000112715
Middle	0-1	HG	ENSG00000115738
Middle	0-1	HG	ENSG00000115844
Middle	0-1	HG	ENSG00000116285
Middle	0-1	HG	ENSG00000117318
Middle	0-1	HG	ENSG00000118515
Middle	0-1	HG	ENSG00000119508
Middle	0-1	HG	ENSG00000125968
Middle	0-1	HG	ENSG00000128594
Middle	0-1	HG	ENSG00000132510
Middle	0-1	HG	ENSG00000136826
Middle	0-1	HG	ENSG00000138166
Middle	0-1	HG	ENSG00000138623
Middle	0-1	HG	ENSG00000146232
Middle	0-1	HG	ENSG00000164683
Middle	0-1	HG	ENSG00000166886
Middle	0-1	HG	ENSG00000176907
Middle	0-1	HG	ENSG00000183691
Middle	0-1	HG	ENSG00000187678
Middle	0-1	HG	ENSG00000188483
Middle	0-1	HG	ENSG00000197019
Middle	0-1	HG	ENSG00000197461
Late	0-4	NG	ENSG00000003989
Late	0-4	NG	ENSG00000007908
Late	0-4	NG	ENSG00000048052

K-Means Cluster	Time Point	Condition	Gene ID
Late	0-4	NG	ENSG00000049130
Late	0-4	NG	ENSG00000056558
Late	0-4	NG	ENSG00000067955
Late	0-4	NG	ENSG00000074047
Late	0-4	NG	ENSG00000095752
Late	0-4	NG	ENSG00000101670
Late	0-4	NG	ENSG00000102755
Late	0-4	NG	ENSG00000105088
Late	0-4	NG	ENSG00000105810
Late	0-4	NG	ENSG00000105855
Late	0-4	NG	ENSG00000106070
Late	0-4	NG	ENSG00000107562
Late	0-4	NG	ENSG00000107731
Late	0-4	NG	ENSG00000115602
Late	0-4	NG	ENSG00000116711
Late	0-4	NG	ENSG00000117595
Late	0-4	NG	ENSG00000118946
Late	0-4	NG	ENSG00000120693
Late	0-4	NG	ENSG00000130201
Late	0-4	NG	ENSG00000131409
Late	0-4	NG	ENSG00000133657
Late	0-4	NG	ENSG00000135547
Late	0-4	NG	ENSG00000136244
Late	0-4	NG	ENSG00000137872
Late	0-4	NG	ENSG00000144366
Late	0-4	NG	ENSG00000150630
Late	0-4	NG	ENSG00000151136
Late	0-4	NG	ENSG00000152689
Late	0-4	NG	ENSG00000154734
Late	0-4	NG	ENSG00000162407
Late	0-4	NG	ENSG00000162692
Late	0-4	NG	ENSG00000164512

K-Means Cluster	Time Point	Condition	Gene ID
Late	0-4	NG	ENSG00000164684
Late	0-4	NG	ENSG00000171246
Late	0-4	NG	ENSG00000179403
Late	0-4	NG	ENSG00000180998
Late	0-4	NG	ENSG00000198108
Late	0-4	NG	ENSG00000206538
Late	0-4	NG	ENSG00000225968
Late	0-4	HG	ENSG00000003989
Late	0-4	HG	ENSG00000007908
Late	0-4	HG	ENSG00000048052
Late	0-4	HG	ENSG00000049130
Late	0-4	HG	ENSG00000049759
Late	0-4	HG	ENSG00000056558
Late	0-4	HG	ENSG00000067798
Late	0-4	HG	ENSG00000067955
Late	0-4	HG	ENSG00000068078
Late	0-4	HG	ENSG00000074047
Late	0-4	HG	ENSG00000095752
Late	0-4	HG	ENSG00000101670
Late	0-4	HG	ENSG00000102755
Late	0-4	HG	ENSG00000102935
Late	0-4	HG	ENSG00000105088
Late	0-4	HG	ENSG00000105810
Late	0-4	HG	ENSG00000105855
Late	0-4	HG	ENSG00000106070
Late	0-4	HG	ENSG00000107562
Late	0-4	HG	ENSG00000107731
Late	0-4	HG	ENSG00000107984
Late	0-4	HG	ENSG00000112541
Late	0-4	HG	ENSG00000112773
Late	0-4	HG	ENSG00000113532
Late	0-4	HG	ENSG00000113580

K-Means Cluster	Time Point	Condition	Gene ID
Late	0-4	HG	ENSG00000115602
Late	0-4	HG	ENSG00000116711
Late	0-4	HG	ENSG00000117595
Late	0-4	HG	ENSG00000118946
Late	0-4	HG	ENSG00000119938
Late	0-4	HG	ENSG00000120693
Late	0-4	HG	ENSG00000122641
Late	0-4	HG	ENSG00000130201
Late	0-4	HG	ENSG00000131149
Late	0-4	HG	ENSG00000131409
Late	0-4	HG	ENSG00000131831
Late	0-4	HG	ENSG00000133657
Late	0-4	HG	ENSG00000134294
Late	0-4	HG	ENSG00000134363
Late	0-4	HG	ENSG00000135547
Late	0-4	HG	ENSG00000136244
Late	0-4	HG	ENSG00000137203
Late	0-4	HG	ENSG00000137834
Late	0-4	HG	ENSG00000137872
Late	0-4	HG	ENSG00000138675
Late	0-4	HG	ENSG00000141668
Late	0-4	HG	ENSG00000144063
Late	0-4	HG	ENSG00000144366
Late	0-4	HG	ENSG00000146374
Late	0-4	HG	ENSG00000150630
Late	0-4	HG	ENSG00000151012
Late	0-4	HG	ENSG00000151136
Late	0-4	HG	ENSG00000152689
Late	0-4	HG	ENSG00000153814
Late	0-4	HG	ENSG00000154734
Late	0-4	HG	ENSG00000157240
Late	0-4	HG	ENSG00000162407

K-Means Cluster	Time Point	Condition	Gene ID
Late	0-4	HG	ENSG00000162692
Late	0-4	HG	ENSG00000163235
Late	0-4	HG	ENSG00000164283
Late	0-4	HG	ENSG00000164512
Late	0-4	HG	ENSG00000164684
Late	0-4	HG	ENSG00000164823
Late	0-4	HG	ENSG00000166510
Late	0-4	HG	ENSG00000168874
Late	0-4	HG	ENSG00000169429
Late	0-4	HG	ENSG00000170293
Late	0-4	HG	ENSG00000170775
Late	0-4	HG	ENSG00000171246
Late	0-4	HG	ENSG00000177679
Late	0-4	HG	ENSG00000179403
Late	0-4	HG	ENSG00000180998
Late	0-4	HG	ENSG00000181541
Late	0-4	HG	ENSG00000183049
Late	0-4	HG	ENSG00000184226
Late	0-4	HG	ENSG00000184307
Late	0-4	HG	ENSG00000185269
Late	0-4	HG	ENSG00000198108
Late	0-4	HG	ENSG00000204217
Late	0-4	HG	ENSG00000206538
Late	0-4	HG	ENSG00000225968
Late	0-4	HG	ENSG00000253368
Late	0-4	HG	ENSG00000265972
4hr	1-4	NG	ENSG00000002079
4hr	1-4	NG	ENSG00000005513
4hr	1-4	NG	ENSG00000006459
4hr	1-4	NG	ENSG00000007384
4hr	1-4	NG	ENSG00000008083
4hr	1-4	NG	ENSG00000008405

K-Means Cluster	Time Point	Condition	Gene ID
4hr	1-4	NG	ENSG00000011422
4hr	1-4	NG	ENSG00000011426
4hr	1-4	NG	ENSG00000013810
4hr	1-4	NG	ENSG00000015133
4hr	1-4	NG	ENSG00000019549
4hr	1-4	NG	ENSG00000023445
4hr	1-4	NG	ENSG00000023902
4hr	1-4	NG	ENSG00000033327
4hr	1-4	NG	ENSG00000035499
4hr	1-4	NG	ENSG00000054598
4hr	1-4	NG	ENSG00000054967
4hr	1-4	NG	ENSG00000057704
4hr	1-4	NG	ENSG00000058404
4hr	1-4	NG	ENSG00000059728
4hr	1-4	NG	ENSG00000064692
4hr	1-4	NG	ENSG00000065308
4hr	1-4	NG	ENSG00000066027
4hr	1-4	NG	ENSG00000066279
4hr	1-4	NG	ENSG00000067064
4hr	1-4	NG	ENSG00000068489
4hr	1-4	NG	ENSG00000069399
4hr	1-4	NG	ENSG00000069667
4hr	1-4	NG	ENSG00000070610
4hr	1-4	NG	ENSG00000070614
4hr	1-4	NG	ENSG00000070669
4hr	1-4	NG	ENSG00000071246
4hr	1-4	NG	ENSG00000072201
4hr	1-4	NG	ENSG00000073711
4hr	1-4	NG	ENSG00000075218
4hr	1-4	NG	ENSG00000075426
4hr	1-4	NG	ENSG00000075702
4hr	1-4	NG	ENSG00000076382

K-Means Cluster	Time Point	Condition	Gene ID
4hr	1-4	NG	ENSG00000079337
4hr	1-4	NG	ENSG00000079616
4hr	1-4	NG	ENSG00000080986
4hr	1-4	NG	ENSG00000082126
4hr	1-4	NG	ENSG00000087074
4hr	1-4	NG	ENSG00000087494
4hr	1-4	NG	ENSG00000088325
4hr	1-4	NG	ENSG00000089685
4hr	1-4	NG	ENSG00000089820
4hr	1-4	NG	ENSG00000090776
4hr	1-4	NG	ENSG00000090889
4hr	1-4	NG	ENSG00000092621
4hr	1-4	NG	ENSG00000095066
4hr	1-4	NG	ENSG00000095370
4hr	1-4	NG	ENSG00000097096
4hr	1-4	NG	ENSG00000099282
4hr	1-4	NG	ENSG00000099860
4hr	1-4	NG	ENSG00000099889
4hr	1-4	NG	ENSG00000100027
4hr	1-4	NG	ENSG00000100034
4hr	1-4	NG	ENSG00000100036
4hr	1-4	NG	ENSG00000100276
4hr	1-4	NG	ENSG00000100292
4hr	1-4	NG	ENSG00000100311
4hr	1-4	NG	ENSG00000100526
4hr	1-4	NG	ENSG00000100625
4hr	1-4	NG	ENSG00000100629
4hr	1-4	NG	ENSG00000100647
4hr	1-4	NG	ENSG00000100650
4hr	1-4	NG	ENSG00000100906
4hr	1-4	NG	ENSG00000101096
4hr	1-4	NG	ENSG00000101224

K-Means Cluster	Time Point	Condition	Gene ID
4hr	1-4	NG	ENSG00000101255
4hr	1-4	NG	ENSG00000101265
4hr	1-4	NG	ENSG00000101384
4hr	1-4	NG	ENSG00000101445
4hr	1-4	NG	ENSG00000101447
4hr	1-4	NG	ENSG00000101850
4hr	1-4	NG	ENSG00000101871
4hr	1-4	NG	ENSG00000102010
4hr	1-4	NG	ENSG00000102034
4hr	1-4	NG	ENSG00000102760
4hr	1-4	NG	ENSG00000102890
4hr	1-4	NG	ENSG00000103241
4hr	1-4	NG	ENSG00000103257
4hr	1-4	NG	ENSG00000104081
4hr	1-4	NG	ENSG00000104147
4hr	1-4	NG	ENSG00000105287
4hr	1-4	NG	ENSG00000105321
4hr	1-4	NG	ENSG00000105443
4hr	1-4	NG	ENSG00000105656
4hr	1-4	NG	ENSG00000105722
4hr	1-4	NG	ENSG00000105851
4hr	1-4	NG	ENSG00000106003
4hr	1-4	NG	ENSG00000106089
4hr	1-4	NG	ENSG00000106236
4hr	1-4	NG	ENSG00000106829
4hr	1-4	NG	ENSG00000106852
4hr	1-4	NG	ENSG00000107551
4hr	1-4	NG	ENSG00000107719
4hr	1-4	NG	ENSG00000107807
4hr	1-4	NG	ENSG00000107821
4hr	1-4	NG	ENSG00000107968
4hr	1-4	NG	ENSG00000108551

K-Means Cluster	Time Point	Condition	Gene ID
4hr	1-4	NG	ENSG00000108691
4hr	1-4	NG	ENSG00000109458
4hr	1-4	NG	ENSG00000109501
4hr	1-4	NG	ENSG00000109787
4hr	1-4	NG	ENSG00000109805
4hr	1-4	NG	ENSG00000111057
4hr	1-4	NG	ENSG00000111110
4hr	1-4	NG	ENSG00000111206
4hr	1-4	NG	ENSG00000111252
4hr	1-4	NG	ENSG00000111665
4hr	1-4	NG	ENSG00000111859
4hr	1-4	NG	ENSG00000111961
4hr	1-4	NG	ENSG00000112137
4hr	1-4	NG	ENSG00000112297
4hr	1-4	NG	ENSG00000112658
4hr	1-4	NG	ENSG00000112715
4hr	1-4	NG	ENSG00000112742
4hr	1-4	NG	ENSG00000112972
4hr	1-4	NG	ENSG00000112984
4hr	1-4	NG	ENSG00000113070
4hr	1-4	NG	ENSG00000113555
4hr	1-4	NG	ENSG00000113749
4hr	1-4	NG	ENSG00000113763
4hr	1-4	NG	ENSG00000114019
4hr	1-4	NG	ENSG00000114166
4hr	1-4	NG	ENSG00000114812
4hr	1-4	NG	ENSG00000115163
4hr	1-4	NG	ENSG00000115525
4hr	1-4	NG	ENSG00000115548
4hr	1-4	NG	ENSG00000115641
4hr	1-4	NG	ENSG00000115844
4hr	1-4	NG	ENSG00000116017

K-Means Cluster	Time Point	Condition	Gene ID
4hr	1-4	NG	ENSG00000116285
4hr	1-4	NG	ENSG00000116604
4hr	1-4	NG	ENSG00000116991
4hr	1-4	NG	ENSG00000117036
4hr	1-4	NG	ENSG00000117318
4hr	1-4	NG	ENSG00000117399
4hr	1-4	NG	ENSG00000117525
4hr	1-4	NG	ENSG00000117650
4hr	1-4	NG	ENSG00000117724
4hr	1-4	NG	ENSG00000118193
4hr	1-4	NG	ENSG00000118503
4hr	1-4	NG	ENSG00000118515
4hr	1-4	NG	ENSG00000119403
4hr	1-4	NG	ENSG00000119508
4hr	1-4	NG	ENSG00000119630
4hr	1-4	NG	ENSG00000119669
4hr	1-4	NG	ENSG00000119718
4hr	1-4	NG	ENSG00000119900
4hr	1-4	NG	ENSG00000120129
4hr	1-4	NG	ENSG00000120278
4hr	1-4	NG	ENSG00000121152
4hr	1-4	NG	ENSG00000121858
4hr	1-4	NG	ENSG00000121957
4hr	1-4	NG	ENSG00000122861
4hr	1-4	NG	ENSG00000122863
4hr	1-4	NG	ENSG00000122966
4hr	1-4	NG	ENSG00000123080
4hr	1-4	NG	ENSG00000123485
4hr	1-4	NG	ENSG00000124762
4hr	1-4	NG	ENSG00000125089
4hr	1-4	NG	ENSG00000125347
4hr	1-4	NG	ENSG00000125812

K-Means Cluster	Time Point	Condition	Gene ID
4hr	1-4	NG	ENSG00000126705
4hr	1-4	NG	ENSG00000126778
4hr	1-4	NG	ENSG00000126787
4hr	1-4	NG	ENSG00000126878
4hr	1-4	NG	ENSG00000127080
4hr	1-4	NG	ENSG00000127528
4hr	1-4	NG	ENSG00000127533
4hr	1-4	NG	ENSG00000128016
4hr	1-4	NG	ENSG00000128294
4hr	1-4	NG	ENSG00000128342
4hr	1-4	NG	ENSG00000128641
4hr	1-4	NG	ENSG00000128833
4hr	1-4	NG	ENSG00000128849
4hr	1-4	NG	ENSG00000128917
4hr	1-4	NG	ENSG00000128944
4hr	1-4	NG	ENSG00000129195
4hr	1-4	NG	ENSG00000129514
4hr	1-4	NG	ENSG00000129810
4hr	1-4	NG	ENSG00000130024
4hr	1-4	NG	ENSG00000130164
4hr	1-4	NG	ENSG00000130222
4hr	1-4	NG	ENSG00000130449
4hr	1-4	NG	ENSG00000130479
4hr	1-4	NG	ENSG00000130513
4hr	1-4	NG	ENSG00000131370
4hr	1-4	NG	ENSG00000131652
4hr	1-4	NG	ENSG00000131669
4hr	1-4	NG	ENSG00000131747
4hr	1-4	NG	ENSG00000131759
4hr	1-4	NG	ENSG00000131773
4hr	1-4	NG	ENSG00000132003
4hr	1-4	NG	ENSG00000132326

K-Means Cluster	Time Point	Condition	Gene ID
4hr	1-4	NG	ENSG00000132334
4hr	1-4	NG	ENSG00000132481
4hr	1-4	NG	ENSG00000132510
4hr	1-4	NG	ENSG00000132563
4hr	1-4	NG	ENSG00000133800
4hr	1-4	NG	ENSG00000134057
4hr	1-4	NG	ENSG00000134107
4hr	1-4	NG	ENSG00000134222
4hr	1-4	NG	ENSG00000134291
4hr	1-4	NG	ENSG00000134508
4hr	1-4	NG	ENSG00000134690
4hr	1-4	NG	ENSG00000135074
4hr	1-4	NG	ENSG00000135363
4hr	1-4	NG	ENSG00000135451
4hr	1-4	NG	ENSG00000135476
4hr	1-4	NG	ENSG00000135604
4hr	1-4	NG	ENSG00000135625
4hr	1-4	NG	ENSG00000135966
4hr	1-4	NG	ENSG00000136108
4hr	1-4	NG	ENSG00000136114
4hr	1-4	NG	ENSG00000136153
4hr	1-4	NG	ENSG00000136158
4hr	1-4	NG	ENSG00000136237
4hr	1-4	NG	ENSG00000136603
4hr	1-4	NG	ENSG00000136717
4hr	1-4	NG	ENSG00000136826
4hr	1-4	NG	ENSG00000136861
4hr	1-4	NG	ENSG00000137193
4hr	1-4	NG	ENSG00000137266
4hr	1-4	NG	ENSG00000137486
4hr	1-4	NG	ENSG00000137804
4hr	1-4	NG	ENSG00000137807
4hr	1-4	NG	ENSG00000137812
4hr	1-4	NG	ENSG00000137825
4hr	1-4	NG	ENSG00000137878
4hr	1-4	NG	ENSG00000138160
4hr	1-4	NG	ENSG00000138162
4hr	1-4	NG	ENSG00000138166
4hr	1-4	NG	ENSG00000138180
4hr	1-4	NG	ENSG00000138347
4hr	1-4	NG	ENSG00000138386
4hr	1-4	NG	ENSG00000138623
4hr	1-4	NG	ENSG00000138835
4hr	1-4	NG	ENSG00000139354
4hr	1-4	NG	ENSG00000139926
4hr	1-4	NG	ENSG00000139998
4hr	1-4	NG	ENSG00000140451
4hr	1-4	NG	ENSG00000140691
4hr	1-4	NG	ENSG00000140853
4hr	1-4	NG	ENSG00000140873
4hr	1-4	NG	ENSG00000141582
4hr	1-4	NG	ENSG00000142178
4hr	1-4	NG	ENSG00000142279
4hr	1-4	NG	ENSG00000142396
4hr	1-4	NG	ENSG00000142627
4hr	1-4	NG	ENSG00000142731
4hr	1-4	NG	ENSG00000142871
4hr	1-4	NG	ENSG00000142945
4hr	1-4	NG	ENSG00000143067
4hr	1-4	NG	ENSG00000143228
4hr	1-4	NG	ENSG00000143344
4hr	1-4	NG	ENSG00000143367
4hr	1-4	NG	ENSG00000143384
4hr	1-4	NG	ENSG00000143772
4hr	1-4	NG	ENSG00000143816
4hr	1-4	NG	ENSG00000143878
4hr	1-4	NG	ENSG00000144560
4hr	1-4	NG	ENSG00000144645
4hr	1-4	NG	ENSG00000144655
4hr	1-4	NG	ENSG00000144677
4hr	1-4	NG	ENSG00000144749
4hr	1-4	NG	ENSG00000144802
4hr	1-4	NG	ENSG00000144824
4hr	1-4	NG	ENSG00000145386
4hr	1-4	NG	ENSG00000145911
4hr	1-4	NG	ENSG00000146021
4hr	1-4	NG	ENSG00000146094
4hr	1-4	NG	ENSG00000146232
4hr	1-4	NG	ENSG00000146918
4hr	1-4	NG	ENSG00000146950
4hr	1-4	NG	ENSG00000147408
4hr	1-4	NG	ENSG00000147852
4hr	1-4	NG	ENSG00000148339
4hr	1-4	NG	ENSG00000148700
4hr	1-4	NG	ENSG00000148773
4hr	1-4	NG	ENSG00000148841
4hr	1-4	NG	ENSG00000148926
4hr	1-4	NG	ENSG00000149289
4hr	1-4	NG	ENSG00000149503
4hr	1-4	NG	ENSG00000149646
4hr	1-4	NG	ENSG00000149798
4hr	1-4	NG	ENSG00000149809
4hr	1-4	NG	ENSG00000150907
4hr	1-4	NG	ENSG00000151014
4hr	1-4	NG	ENSG00000151690
4hr	1-4	NG	ENSG00000151725

K-Means Cluster	Time Point	Condition	Gene ID
4hr	1-4	NG	ENSG00000151967
4hr	1-4	NG	ENSG00000152284
4hr	1-4	NG	ENSG00000152454
4hr	1-4	NG	ENSG00000152953
4hr	1-4	NG	ENSG00000153094
4hr	1-4	NG	ENSG00000153208
4hr	1-4	NG	ENSG00000153234
4hr	1-4	NG	ENSG00000153443
4hr	1-4	NG	ENSG00000153551
4hr	1-4	NG	ENSG00000154639
4hr	1-4	NG	ENSG00000155090
4hr	1-4	NG	ENSG00000156030
4hr	1-4	NG	ENSG00000156273
4hr	1-4	NG	ENSG00000156466
4hr	1-4	NG	ENSG00000156970
4hr	1-4	NG	ENSG00000156983
4hr	1-4	NG	ENSG00000157404
4hr	1-4	NG	ENSG00000157456
4hr	1-4	NG	ENSG00000157510
4hr	1-4	NG	ENSG00000157557
4hr	1-4	NG	ENSG00000157613
4hr	1-4	NG	ENSG00000157617
4hr	1-4	NG	ENSG00000158050
4hr	1-4	NG	ENSG00000158106
4hr	1-4	NG	ENSG00000158195
4hr	1-4	NG	ENSG00000158402
4hr	1-4	NG	ENSG00000158555
4hr	1-4	NG	ENSG00000158856
4hr	1-4	NG	ENSG00000158859
4hr	1-4	NG	ENSG00000159167
4hr	1-4	NG	ENSG00000159200
4hr	1-4	NG	ENSG00000159261

K-Means Cluster	Time Point	Condition	Gene ID
4hr	1-4	NG	ENSG00000159640
4hr	1-4	NG	ENSG00000160013
4hr	1-4	NG	ENSG00000160179
4hr	1-4	NG	ENSG00000160447
4hr	1-4	NG	ENSG00000160796
4hr	1-4	NG	ENSG00000160888
4hr	1-4	NG	ENSG00000161800
4hr	1-4	NG	ENSG00000161835
4hr	1-4	NG	ENSG00000161888
4hr	1-4	NG	ENSG00000161940
4hr	1-4	NG	ENSG00000161960
4hr	1-4	NG	ENSG00000162413
4hr	1-4	NG	ENSG00000162496
4hr	1-4	NG	ENSG00000162522
4hr	1-4	NG	ENSG00000163083
4hr	1-4	NG	ENSG00000163171
4hr	1-4	NG	ENSG00000163219
4hr	1-4	NG	ENSG00000163491
4hr	1-4	NG	ENSG00000163535
4hr	1-4	NG	ENSG00000163545
4hr	1-4	NG	ENSG00000163659
4hr	1-4	NG	ENSG00000163660
4hr	1-4	NG	ENSG00000163874
4hr	1-4	NG	ENSG00000164087
4hr	1-4	NG	ENSG00000164104
4hr	1-4	NG	ENSG00000164611
4hr	1-4	NG	ENSG00000164849
4hr	1-4	NG	ENSG00000164877
4hr	1-4	NG	ENSG00000165071
4hr	1-4	NG	ENSG00000165655
4hr	1-4	NG	ENSG00000165886
4hr	1-4	NG	ENSG00000165959

K-Means Cluster	Time Point	Condition	Gene ID
4hr	1-4	NG	ENSG00000166483
4hr	1-4	NG	ENSG00000166592
4hr	1-4	NG	ENSG00000166851
4hr	1-4	NG	ENSG00000166886
4hr	1-4	NG	ENSG00000167106
4hr	1-4	NG	ENSG00000167173
4hr	1-4	NG	ENSG00000167191
4hr	1-4	NG	ENSG00000167470
4hr	1-4	NG	ENSG00000167508
4hr	1-4	NG	ENSG00000167565
4hr	1-4	NG	ENSG00000167680
4hr	1-4	NG	ENSG00000167772
4hr	1-4	NG	ENSG00000167874
4hr	1-4	NG	ENSG00000168026
4hr	1-4	NG	ENSG00000168078
4hr	1-4	NG	ENSG00000168209
4hr	1-4	NG	ENSG00000168476
4hr	1-4	NG	ENSG00000168758
4hr	1-4	NG	ENSG00000168874
4hr	1-4	NG	ENSG00000169242
4hr	1-4	NG	ENSG00000169429
4hr	1-4	NG	ENSG00000169607
4hr	1-4	NG	ENSG00000169679
4hr	1-4	NG	ENSG00000169891
4hr	1-4	NG	ENSG00000169926
4hr	1-4	NG	ENSG00000169991
4hr	1-4	NG	ENSG00000170011
4hr	1-4	NG	ENSG00000170175
4hr	1-4	NG	ENSG00000170312
4hr	1-4	NG	ENSG00000170365
4hr	1-4	NG	ENSG00000170421
4hr	1-4	NG	ENSG00000170485

K-Means Cluster	Time Point	Condition	Gene ID
4hr	1-4	NG	ENSG00000170525
4hr	1-4	NG	ENSG00000170852
4hr	1-4	NG	ENSG00000170873
4hr	1-4	NG	ENSG00000171056
4hr	1-4	NG	ENSG00000171388
4hr	1-4	NG	ENSG00000171604
4hr	1-4	NG	ENSG00000171617
4hr	1-4	NG	ENSG00000171621
4hr	1-4	NG	ENSG00000171729
4hr	1-4	NG	ENSG00000171791
4hr	1-4	NG	ENSG00000172031
4hr	1-4	NG	ENSG00000172059
4hr	1-4	NG	ENSG00000172183
4hr	1-4	NG	ENSG00000173013
4hr	1-4	NG	ENSG00000173207
4hr	1-4	NG	ENSG00000173276
4hr	1-4	NG	ENSG00000173281
4hr	1-4	NG	ENSG00000173334
4hr	1-4	NG	ENSG00000173535
4hr	1-4	NG	ENSG00000173846
4hr	1-4	NG	ENSG00000174004
4hr	1-4	NG	ENSG00000174059
4hr	1-4	NG	ENSG00000174136
4hr	1-4	NG	ENSG00000174370
4hr	1-4	NG	ENSG00000175063
4hr	1-4	NG	ENSG00000175105
4hr	1-4	NG	ENSG00000175264
4hr	1-4	NG	ENSG00000175274
4hr	1-4	NG	ENSG00000175505
4hr	1-4	NG	ENSG00000175592
4hr	1-4	NG	ENSG00000175832
4hr	1-4	NG	ENSG00000176170

K-Means Cluster	Time Point	Condition	Gene ID
4hr	1-4	NG	ENSG00000176692
4hr	1-4	NG	ENSG00000176749
4hr	1-4	NG	ENSG00000176842
4hr	1-4	NG	ENSG00000177125
4hr	1-4	NG	ENSG00000177191
4hr	1-4	NG	ENSG00000177374
4hr	1-4	NG	ENSG00000177606
4hr	1-4	NG	ENSG00000178445
4hr	1-4	NG	ENSG00000178878
4hr	1-4	NG	ENSG00000178999
4hr	1-4	NG	ENSG00000179314
4hr	1-4	NG	ENSG00000179348
4hr	1-4	NG	ENSG00000179361
4hr	1-4	NG	ENSG00000179431
4hr	1-4	NG	ENSG00000179750
4hr	1-4	NG	ENSG00000179862
4hr	1-4	NG	ENSG00000180035
4hr	1-4	NG	ENSG00000180530
4hr	1-4	NG	ENSG00000180592
4hr	1-4	NG	ENSG00000180730
4hr	1-4	NG	ENSG00000181472
4hr	1-4	NG	ENSG00000181634
4hr	1-4	NG	ENSG00000181773
4hr	1-4	NG	ENSG00000182022
4hr	1-4	NG	ENSG00000182118
4hr	1-4	NG	ENSG00000182541
4hr	1-4	NG	ENSG00000182704
4hr	1-4	NG	ENSG00000183072
4hr	1-4	NG	ENSG00000183307
4hr	1-4	NG	ENSG00000183337
4hr	1-4	NG	ENSG00000183386
4hr	1-4	NG	ENSG00000183496

K-Means Cluster	Time Point	Condition	Gene ID
4hr	1-4	NG	ENSG00000183580
4hr	1-4	NG	ENSG00000183856
4hr	1-4	NG	ENSG00000183873
4hr	1-4	NG	ENSG00000184113
4hr	1-4	NG	ENSG00000184254
4hr	1-4	NG	ENSG00000184281
4hr	1-4	NG	ENSG00000184557
4hr	1-4	NG	ENSG00000184635
4hr	1-4	NG	ENSG00000184661
4hr	1-4	NG	ENSG00000184792
4hr	1-4	NG	ENSG00000185022
4hr	1-4	NG	ENSG00000185262
4hr	1-4	NG	ENSG00000185338
4hr	1-4	NG	ENSG00000185347
4hr	1-4	NG	ENSG00000185361
4hr	1-4	NG	ENSG00000185920
4hr	1-4	NG	ENSG00000186185
4hr	1-4	NG	ENSG00000186193
4hr	1-4	NG	ENSG00000186479
4hr	1-4	NG	ENSG00000186480
4hr	1-4	NG	ENSG00000187266
4hr	1-4	NG	ENSG00000187479
4hr	1-4	NG	ENSG00000187513
4hr	1-4	NG	ENSG00000187678
4hr	1-4	NG	ENSG00000187764
4hr	1-4	NG	ENSG00000188042
4hr	1-4	NG	ENSG00000188277
4hr	1-4	NG	ENSG00000188295
4hr	1-4	NG	ENSG00000188483
4hr	1-4	NG	ENSG00000188522
4hr	1-4	NG	ENSG00000188732
4hr	1-4	NG	ENSG00000189152

K-Means Cluster	Time Point	Condition	Gene ID
4hr	1-4	NG	ENSG00000196169
4hr	1-4	NG	ENSG00000196371
4hr	1-4	NG	ENSG00000196428
4hr	1-4	NG	ENSG00000196449
4hr	1-4	NG	ENSG00000196468
4hr	1-4	NG	ENSG00000196611
4hr	1-4	NG	ENSG00000196730
4hr	1-4	NG	ENSG00000196843
4hr	1-4	NG	ENSG00000197019
4hr	1-4	NG	ENSG00000197063
4hr	1-4	NG	ENSG00000197182
4hr	1-4	NG	ENSG00000197183
4hr	1-4	NG	ENSG00000197329
4hr	1-4	NG	ENSG00000197380
4hr	1-4	NG	ENSG00000197461
4hr	1-4	NG	ENSG00000197536
4hr	1-4	NG	ENSG00000197622
4hr	1-4	NG	ENSG00000197852
4hr	1-4	NG	ENSG00000198055
4hr	1-4	NG	ENSG00000198142
4hr	1-4	NG	ENSG00000198324
4hr	1-4	NG	ENSG00000198355
4hr	1-4	NG	ENSG00000198435
4hr	1-4	NG	ENSG00000198517
4hr	1-4	NG	ENSG00000198576
4hr	1-4	NG	ENSG00000198873
4hr	1-4	NG	ENSG00000198901
4hr	1-4	NG	ENSG00000203804
4hr	1-4	NG	ENSG00000203883
4hr	1-4	NG	ENSG00000205476
4hr	1-4	NG	ENSG00000206573
4hr	1-4	NG	ENSG00000207808
4hr	1-4	NG	ENSG00000207980
4hr	1-4	NG	ENSG00000213347
4hr	1-4	NG	ENSG00000214176
4hr	1-4	NG	ENSG00000214357
4hr	1-4	NG	ENSG00000214944
4hr	1-4	NG	ENSG00000215417
4hr	1-4	NG	ENSG00000215788
4hr	1-4	NG	ENSG00000216775
4hr	1-4	NG	ENSG00000220785
4hr	1-4	NG	ENSG00000221883
4hr	1-4	NG	ENSG00000222047
4hr	1-4	NG	ENSG00000222455
4hr	1-4	NG	ENSG00000223855
4hr	1-4	NG	ENSG00000224959
4hr	1-4	NG	ENSG00000225614
4hr	1-4	NG	ENSG00000226380
4hr	1-4	NG	ENSG00000229729
4hr	1-4	NG	ENSG00000229953
4hr	1-4	NG	ENSG00000230701
4hr	1-4	NG	ENSG00000230753
4hr	1-4	NG	ENSG00000235750
4hr	1-4	NG	ENSG00000235831
4hr	1-4	NG	ENSG00000237973
4hr	1-4	NG	ENSG00000240694
4hr	1-4	NG	ENSG00000241852
4hr	1-4	NG	ENSG00000242829
4hr	1-4	NG	ENSG00000249992
4hr	1-4	NG	ENSG00000250510
4hr	1-4	NG	ENSG00000250588
4hr	1-4	NG	ENSG00000251493
4hr	1-4	NG	ENSG00000253669
4hr	1-4	NG	ENSG00000253958
4hr	1-4	NG	ENSG00000254470
4hr	1-4	NG	ENSG00000255112
4hr	1-4	NG	ENSG00000255176
4hr	1-4	NG	ENSG00000259518
4hr	1-4	NG	ENSG00000259863
4hr	1-4	NG	ENSG00000260942
4hr	1-4	NG	ENSG00000260992
4hr	1-4	NG	ENSG00000260996
4hr	1-4	NG	ENSG00000261069
4hr	1-4	NG	ENSG00000261253
4hr	1-4	NG	ENSG00000262292
4hr	1-4	NG	ENSG00000263155
4hr	1-4	NG	ENSG00000263934
4hr	1-4	NG	ENSG00000264395
4hr	1-4	NG	ENSG00000264635
4hr	1-4	NG	ENSG00000265185
4hr	1-4	NG	ENSG00000265666
4hr	1-4	NG	ENSG00000265982
4hr	1-4	NG	ENSG00000267041
4hr	1-4	NG	ENSG00000267519
4hr	1-4	NG	ENSG00000267583
4hr	1-4	NG	ENSG00000267598
4hr	1-4	NG	ENSG00000269929
4hr	1-4	NG	ENSG00000270069
4hr	1-4	NG	ENSG00000270607
4hr	1-4	NG	ENSG00000272405
4hr	1-4	NG	ENSG00000272574
4hr	1-4	NG	ENSG00000273489
4hr	1-4	NG	ENSG00000273729
4hr	1-4	NG	ENSG00000273820
4hr	1-4	NG	ENSG00000274636
4hr	1-4	NG	ENSG00000274995

K-Means Cluster	Time Point	Condition	Gene ID
4hr	1-4	NG	ENSG00000275031
4hr	1-4	NG	ENSG00000275202
4hr	1-4	NG	ENSG00000275793
4hr	1-4	NG	ENSG00000275993
4hr	1-4	NG	ENSG00000277462
4hr	1-4	NG	ENSG00000277775
4hr	1-4	NG	ENSG00000278546
4hr	1-4	NG	ENSG00000278921
4hr	1-4	NG	ENSG00000279117
4hr	1-4	NG	ENSG00000279520
4hr	1-4	NG	ENSG00000280138
4hr	1-4	NG	ENSG00000281881
4hr	1-4	HG	ENSG00000007384
4hr	1-4	HG	ENSG00000008083
4hr	1-4	HG	ENSG00000013810
4hr	1-4	HG	ENSG00000015133
4hr	1-4	HG	ENSG00000035499
4hr	1-4	HG	ENSG00000043143
4hr	1-4	HG	ENSG00000049283
4hr	1-4	HG	ENSG00000054967
4hr	1-4	HG	ENSG00000057704
4hr	1-4	HG	ENSG00000064692
4hr	1-4	HG	ENSG00000065308
4hr	1-4	HG	ENSG00000070610
4hr	1-4	HG	ENSG00000071246
4hr	1-4	HG	ENSG00000072201
4hr	1-4	HG	ENSG00000075426
4hr	1-4	HG	ENSG00000075651
4hr	1-4	HG	ENSG00000075702
4hr	1-4	HG	ENSG00000080986
4hr	1-4	HG	ENSG00000082126
4hr	1-4	HG	ENSG00000089041

K-Means Cluster	Time Point	Condition	Gene ID
4hr	1-4	HG	ENSG00000090889
4hr	1-4	HG	ENSG00000095370
4hr	1-4	HG	ENSG00000099889
4hr	1-4	HG	ENSG00000100036
4hr	1-4	HG	ENSG00000100292
4hr	1-4	HG	ENSG00000100629
4hr	1-4	HG	ENSG00000100906
4hr	1-4	HG	ENSG00000101096
4hr	1-4	HG	ENSG00000101224
4hr	1-4	HG	ENSG00000101255
4hr	1-4	HG	ENSG00000101265
4hr	1-4	HG	ENSG00000101445
4hr	1-4	HG	ENSG00000101871
4hr	1-4	HG	ENSG00000102010
4hr	1-4	HG	ENSG00000102890
4hr	1-4	HG	ENSG00000104081
4hr	1-4	HG	ENSG00000105287
4hr	1-4	HG	ENSG00000105851
4hr	1-4	HG	ENSG00000106351
4hr	1-4	HG	ENSG00000106852
4hr	1-4	HG	ENSG00000107719
4hr	1-4	HG	ENSG00000109458
4hr	1-4	HG	ENSG00000109501
4hr	1-4	HG	ENSG00000111110
4hr	1-4	HG	ENSG00000111335
4hr	1-4	HG	ENSG00000111665
4hr	1-4	HG	ENSG00000111961
4hr	1-4	HG	ENSG00000112137
4hr	1-4	HG	ENSG00000112297
4hr	1-4	HG	ENSG00000112742
4hr	1-4	HG	ENSG00000112984
4hr	1-4	HG	ENSG00000113070

K-Means Cluster	Time Point	Condition	Gene ID
4hr	1-4	HG	ENSG00000113555
4hr	1-4	HG	ENSG00000114019
4hr	1-4	HG	ENSG00000114812
4hr	1-4	HG	ENSG00000115163
4hr	1-4	HG	ENSG00000115525
4hr	1-4	HG	ENSG00000115641
4hr	1-4	HG	ENSG00000116017
4hr	1-4	HG	ENSG00000117399
4hr	1-4	HG	ENSG00000117525
4hr	1-4	HG	ENSG00000117650
4hr	1-4	HG	ENSG00000117724
4hr	1-4	HG	ENSG00000118193
4hr	1-4	HG	ENSG00000119630
4hr	1-4	HG	ENSG00000119669
4hr	1-4	HG	ENSG00000120129
4hr	1-4	HG	ENSG00000120278
4hr	1-4	HG	ENSG00000120875
4hr	1-4	HG	ENSG00000121152
4hr	1-4	HG	ENSG00000121797
4hr	1-4	HG	ENSG00000121858
4hr	1-4	HG	ENSG00000121957
4hr	1-4	HG	ENSG00000121966
4hr	1-4	HG	ENSG00000122861
4hr	1-4	HG	ENSG00000122966
4hr	1-4	HG	ENSG00000123080
4hr	1-4	HG	ENSG00000123485
4hr	1-4	HG	ENSG00000125347
4hr	1-4	HG	ENSG00000126705
4hr	1-4	HG	ENSG00000126882
4hr	1-4	HG	ENSG00000127533
4hr	1-4	HG	ENSG00000128284
4hr	1-4	HG	ENSG00000128641

K-Means Cluster	Time Point	Condition	Gene ID
4hr	1-4	HG	ENSG00000128805
4hr	1-4	HG	ENSG00000128815
4hr	1-4	HG	ENSG00000128833
4hr	1-4	HG	ENSG00000128917
4hr	1-4	HG	ENSG00000130513
4hr	1-4	HG	ENSG00000131370
4hr	1-4	HG	ENSG00000131747
4hr	1-4	HG	ENSG00000131759
4hr	1-4	HG	ENSG00000132003
4hr	1-4	HG	ENSG00000132470
4hr	1-4	HG	ENSG00000133056
4hr	1-4	HG	ENSG00000134107
4hr	1-4	HG	ENSG00000134222
4hr	1-4	HG	ENSG00000134690
4hr	1-4	HG	ENSG00000135074
4hr	1-4	HG	ENSG00000135476
4hr	1-4	HG	ENSG00000135966
4hr	1-4	HG	ENSG00000136114
4hr	1-4	HG	ENSG00000136158
4hr	1-4	HG	ENSG00000136237
4hr	1-4	HG	ENSG00000136717
4hr	1-4	HG	ENSG00000137193
4hr	1-4	HG	ENSG00000137486
4hr	1-4	HG	ENSG00000137804
4hr	1-4	HG	ENSG00000137807
4hr	1-4	HG	ENSG00000137878
4hr	1-4	HG	ENSG00000138162
4hr	1-4	HG	ENSG00000138347
4hr	1-4	HG	ENSG00000138835
4hr	1-4	HG	ENSG00000139354
4hr	1-4	HG	ENSG00000140451
4hr	1-4	HG	ENSG00000140691

K-Means Cluster	Time Point	Condition	Gene ID
4hr	1-4	HG	ENSG00000140873
4hr	1-4	HG	ENSG00000141068
4hr	1-4	HG	ENSG00000142178
4hr	1-4	HG	ENSG00000142627
4hr	1-4	HG	ENSG00000142945
4hr	1-4	HG	ENSG00000143344
4hr	1-4	HG	ENSG00000143367
4hr	1-4	HG	ENSG00000143772
4hr	1-4	HG	ENSG00000143816
4hr	1-4	HG	ENSG00000144560
4hr	1-4	HG	ENSG00000144677
4hr	1-4	HG	ENSG00000144749
4hr	1-4	HG	ENSG00000144824
4hr	1-4	HG	ENSG00000145911
4hr	1-4	HG	ENSG00000146021
4hr	1-4	HG	ENSG00000146950
4hr	1-4	HG	ENSG00000147408
4hr	1-4	HG	ENSG00000147852
4hr	1-4	HG	ENSG00000148773
4hr	1-4	HG	ENSG00000148841
4hr	1-4	HG	ENSG00000148926
4hr	1-4	HG	ENSG00000149503
4hr	1-4	HG	ENSG00000151692
4hr	1-4	HG	ENSG00000151967
4hr	1-4	HG	ENSG00000152284
4hr	1-4	HG	ENSG00000153094
4hr	1-4	HG	ENSG00000153208
4hr	1-4	HG	ENSG00000153234
4hr	1-4	HG	ENSG00000154639
4hr	1-4	HG	ENSG00000156030
4hr	1-4	HG	ENSG00000156970
4hr	1-4	HG	ENSG00000157404

K-Means Cluster	Time Point	Condition	Gene ID
4hr	1-4	HG	ENSG00000157510
4hr	1-4	HG	ENSG00000157557
4hr	1-4	HG	ENSG00000157613
4hr	1-4	HG	ENSG00000157617
4hr	1-4	HG	ENSG00000158195
4hr	1-4	HG	ENSG00000158402
4hr	1-4	HG	ENSG00000158555
4hr	1-4	HG	ENSG00000158859
4hr	1-4	HG	ENSG00000159167
4hr	1-4	HG	ENSG00000160013
4hr	1-4	HG	ENSG00000160179
4hr	1-4	HG	ENSG00000161800
4hr	1-4	HG	ENSG00000161940
4hr	1-4	HG	ENSG00000162413
4hr	1-4	HG	ENSG00000162496
4hr	1-4	HG	ENSG00000162522
4hr	1-4	HG	ENSG00000163171
4hr	1-4	HG	ENSG00000163219
4hr	1-4	HG	ENSG00000163545
4hr	1-4	HG	ENSG00000163554
4hr	1-4	HG	ENSG00000163659
4hr	1-4	HG	ENSG00000163874
4hr	1-4	HG	ENSG00000164104
4hr	1-4	HG	ENSG00000164849
4hr	1-4	HG	ENSG00000165886
4hr	1-4	HG	ENSG00000165959
4hr	1-4	HG	ENSG00000166851
4hr	1-4	HG	ENSG00000166925
4hr	1-4	HG	ENSG00000167106
4hr	1-4	HG	ENSG00000167191
4hr	1-4	HG	ENSG00000167470
4hr	1-4	HG	ENSG00000167565

K-Means Cluster	Time Point	Condition	Gene ID
4hr	1-4	HG	ENSG00000167680
4hr	1-4	HG	ENSG00000168209
4hr	1-4	HG	ENSG00000168309
4hr	1-4	HG	ENSG00000168497
4hr	1-4	HG	ENSG00000169184
4hr	1-4	HG	ENSG00000169242
4hr	1-4	HG	ENSG00000169607
4hr	1-4	HG	ENSG00000169679
4hr	1-4	HG	ENSG00000170271
4hr	1-4	HG	ENSG00000170365
4hr	1-4	HG	ENSG00000170525
4hr	1-4	HG	ENSG00000171388
4hr	1-4	HG	ENSG00000171621
4hr	1-4	HG	ENSG00000171729
4hr	1-4	HG	ENSG00000172031
4hr	1-4	HG	ENSG00000173334
4hr	1-4	HG	ENSG00000173535
4hr	1-4	HG	ENSG00000174004
4hr	1-4	HG	ENSG00000174059
4hr	1-4	HG	ENSG00000174370
4hr	1-4	HG	ENSG00000175264
4hr	1-4	HG	ENSG00000175274
4hr	1-4	HG	ENSG00000175592
4hr	1-4	HG	ENSG00000177374
4hr	1-4	HG	ENSG00000177606
4hr	1-4	HG	ENSG00000178878
4hr	1-4	HG	ENSG00000178999
4hr	1-4	HG	ENSG00000179314
4hr	1-4	HG	ENSG00000179348
4hr	1-4	HG	ENSG00000179981
4hr	1-4	HG	ENSG00000182022
4hr	1-4	HG	ENSG00000182118

K-Means Cluster	Time Point	Condition	Gene ID
4hr	1-4	HG	ENSG00000183337
4hr	1-4	HG	ENSG00000183496
4hr	1-4	HG	ENSG00000183856
4hr	1-4	HG	ENSG00000184113
4hr	1-4	HG	ENSG00000184661
4hr	1-4	HG	ENSG00000185022
4hr	1-4	HG	ENSG00000185112
4hr	1-4	HG	ENSG00000185262
4hr	1-4	HG	ENSG00000185338
4hr	1-4	HG	ENSG00000185361
4hr	1-4	HG	ENSG00000186185
4hr	1-4	HG	ENSG00000186193
4hr	1-4	HG	ENSG00000186479
4hr	1-4	HG	ENSG00000186642
4hr	1-4	HG	ENSG00000186918
4hr	1-4	HG	ENSG00000187266
4hr	1-4	HG	ENSG00000187479
4hr	1-4	HG	ENSG00000187764
4hr	1-4	HG	ENSG00000188042
4hr	1-4	HG	ENSG00000188277
4hr	1-4	HG	ENSG00000196169
4hr	1-4	HG	ENSG00000196730
4hr	1-4	HG	ENSG00000196782
4hr	1-4	HG	ENSG00000197852
4hr	1-4	HG	ENSG00000198355
4hr	1-4	HG	ENSG00000198435
4hr	1-4	HG	ENSG00000198517
4hr	1-4	HG	ENSG00000198576
4hr	1-4	HG	ENSG00000198873
4hr	1-4	HG	ENSG00000203883
4hr	1-4	HG	ENSG00000205476
4hr	1-4	HG	ENSG00000207980

K-Means Cluster	Time Point	Condition	Gene ID
4hr	1-4	HG	ENSG00000213160
4hr	1-4	HG	ENSG00000213347
4hr	1-4	HG	ENSG00000214357
4hr	1-4	HG	ENSG00000214944
4hr	1-4	HG	ENSG00000216775
4hr	1-4	HG	ENSG00000230266
4hr	1-4	HG	ENSG00000239887
4hr	1-4	HG	ENSG00000240694
4hr	1-4	HG	ENSG00000249279
4hr	1-4	HG	ENSG00000255176
4hr	1-4	HG	ENSG00000261253
4hr	1-4	HG	ENSG00000263155
4hr	1-4	HG	ENSG00000267041
4hr	1-4	HG	ENSG00000267519
4hr	1-4	HG	ENSG00000267583
4hr	1-4	HG	ENSG00000267598
4hr	1-4	HG	ENSG00000272405
4hr	1-4	HG	ENSG00000274636
4hr	1-4	HG	ENSG00000275993
4hr	1-4	HG	ENSG00000277775
4hr	1-4	HG	ENSG00000279117
4hr	1-4	HG	ENSG00000280138
4hr	1-4	HG	ENSG00000281881

Supplementary Table 13. RT-qPCR data for the validation of 12 DEGs

Sample_Name	Condition	Time_Point	SMAD6 qPCR (1/Δct)	CXCL8 qPCR (1/Δct)	TGFA qPCR (1/Δct)	CXCL12 qPCR (1/Δct)	IRF6 qPCR (1/Δct)	IL11 qPCR (1/Δct)	VEGFC qPCR (1/Δct)	CCL2 qPCR (1/Δct)	CXXC5 qPCR (1/Δct)	PDGFA qPCR (1/Δct)	WNT2B qPCR (1/Δct)	EDN1 qPCR (1/Δct)
HG05_2	HG	0.5	0.171239823	0.215576701	0.190995463	0.131687316	0.162777091	0.071600542	0.191361515	0.183382006	0.140499111	0.260709719	0.160920043	0.252705307
HG05_3	HG	0.5	0.170989138	0.220486156	0.192260624	0.131102895	0.166125323	0.073924428	0.188901171		0.139158196	0.260870594	0.155745181	0.251216421
HG05_4	HG	0.5	0.175588168	0.221177094	0.1890418	0.133042062		0.071617473	0.189886395	0.180762903	0.136795519	0.262105157	0.163047338	0.251496943
HG1_1	HG	1	0.196296738		0.190736239	0.162019065	0.165207944	0.088290056	0.195405742	0.178220936	0.155676307	0.283750434		0.247995883
HG1_2	HG	1	0.200579372	0.211875013	0.195453041	0.161030922		0.093504452	0.196864489	0.181638148	0.155166064	0.287238032	0.16064114	
HG1_3	HG	1	0.19533248	0.216699673	0.195221227		0.161196099	0.092981674	0.190606741	0.177787684	0.158396093	0.282163288	0.163287557	0.25332927
HG1_4	HG	1	0.19581965	0.226874628	0.192717014	0.154798689	0.166359821	0.089649995	0.192475791	0.183546334	0.161474439	0.284743798	0.161116911	0.249983543
HG4_1	HG	4	0.21217663	0.244225281	0.235072922	0.170233306	0.19749874	0.110997832	0.21202962	0.172612664	0.151543588	0.277933031	0.155056656	0.240596323
HG4_2	HG	4	0.212609744	0.243141995	0.238078951	0.172672538	0.200927736	0.109747768	0.221297183	0.172207791	0.161332586	0.285100341	0.160130016	0.244046798
HG4_3	HG	4	0.212011976	0.239618662	0.229188418	0.171444143	0.1980405	0.104093684	0.213630157	0.177648662	0.151577861	0.274865681	0.155234958	0.243473452
HG4_4	HG	4	0.213414578	0.230718246	0.234635752	0.169741079	0.198387934	0.105281943	0.217635038	0.176232626	0.15194545	0.277559484	0.155943412	0.247305189
HG8_1	HG	8	0.175452388	0.20342358	0.190047845	0.132805749		0.076593227	0.203267513	0.161780168	0.132989744	0.268005556	0.165689634	0.255457406
HG8_2	HG	8	0.169673706	0.201895375	0.181679316	0.127307091	0.192893159	0.073711254	0.197342378	0.158475761	0.136511625	0.266852707	0.171305681	0.251679008
HG8_3	HG	8	0.170985562	0.201632376		0.13054595	0.186888145	0.076467235	0.197359567			0.274159919	0.17430718	0.252997837
HG8_4	HG	8	0.170489231	0.192574836	0.18630848	0.130898507	0.190345318	0.076039943	0.201257263	0.171036174	0.140045319	0.274451508	0.167457178	0.254669834
HG24_1	HG	24	0.165743276	0.211733406	0.17939681	0.124974902	0.179464302	0.074312816	0.205076743	0.186560722	0.145442484	0.263031993	0.159306519	0.252311464
HG24_2	HG	24		0.209031628	0.184143606	0.124832547	0.176292457	0.069075048	0.206597127		0.138494389	0.258923565	0.156968881	0.243397583
HG24_3	HG	24	0.167096602	0.216471928		0.130174775	0.168815377	0.075206176	0.2026832	0.177041531	0.139927255	0.267793248	0.162793754	0.246134511
HG24_4	HG	24	0.170510985	0.216447526	0.186422094	0.1335419		0.074086246	0.209482121	0.174982665	0.144066063	0.262889246	0.161156309	0.247621311
NG0_1	NG	0	0.148236125	0.203512612	0.175299224	0.121511847	0.166488205	0.070369234	0.185474815	0.174769629	0.139860713	0.259376465	0.158246611	0.243785844
NG0_2	NG	0	0.150072236	0.199255234	0.174854532	0.12039259	0.155069844	0.071462824	0.188269633	0.173889905	0.141778334	0.264238451	0.158297018	0.24443602
NG0_3	NG	0	0.155360432	0.199936714	0.178589878		0.16509277	0.071679999	0.186608206	0.176827932	0.135254994	0.261207054	0.160592314	0.24411592
NG0_4	NG	0	0.156578922	0.199325907	0.175947046	0.125537646	0.168173344	0.071788124	0.190883912		0.140242037	0.265753274	0.163502962	
NG05_1	NG	0.5	0.17251706	0.225675777	0.190190338	0.140856153	0.161591845	0.071804874	0.192078487	0.184807997	0.134625939	0.26255722	0.164697307	0.255738698
NG05_2	NG	0.5	0.172287648		0.190714718	0.141783198	0.164224818	0.07080704	0.19199481	0.17972902	0.142225193	0.27362361	0.165861177	0.24867459
NG05_3	NG	0.5	0.168762479	0.217958434	0.184486187	0.127820913	0.162215711	0.072371301	0.191500312	0.181129001	0.136953499	0.26444115	0.156123294	0.251447257
NG05_4	NG	0.5	0.174375587	0.231817022	0.189856776		0.169481362	0.071340091	0.194383698	0.181020871	0.139542846	0.268429968	0.154031374	0.248087108
NG1_1	NG	1	0.207228883	0.217164483	0.201608737	0.160487485	0.166242	0.093649342	0.194573393	0.182194234	0.160404381	0.291270811	0.156167607	0.251481031
NG1_2	NG	1	0.207118647	0.226831549	0.203318612	0.159624788	0.163281154	0.09628375	0.191623071	0.182611855		0.294358923	0.150028561	0.24995399
NG1_3	NG	1	0.20795768	0.218183255	0.204380755	0.16514725	0.167668744	0.089886498	0.1926292	0.187121377	0.165467805	0.291295603	0.148968543	0.245767856
NG1_4	NG	1	0.189753934	0.235624079	0.19916955	0.157932199	0.16576805	0.094118188	0.194450025	0.185756355	0.151549765	0.288661254		0.246799028
NG4_1	NG	4	0.211513059	0.233153935	0.219636865	0.168840384	0.192274099	0.091020606	0.200409475	0.176872984	0.141467381	0.281386562	0.154813894	0.240942513
NG4_2	NG	4	0.211555391	0.240396991	0.213389591	0.169918734	0.197047004	0.095918013	0.215813447	0.176408632	0.142053734	0.281575188	0.165203165	0.23894389
NG4_3	NG	4	0.207992462	0.239152314	0.221476638	0.162922499	0.200271243	0.0993449	0.212320404	0.177512429	0.147168383	0.287253188	0.161589763	0.243350919
NG4_4	NG	4	0.196924616	0.222436456	0.215959852	0.164090408	0.195437917	0.101424575	0.214742617	0.175216302	0.149130567	0.278740818	0.155869529	0.242174397
NG8_1	NG	8	0.167162847	0.204805684	0.185980801	0.130876349	0.177072511	0.070756814	0.198462357	0.157022176	0.135657693	0.268317772	0.15868944	0.248099787
NG8_2	NG	8	0.165526917	0.203640574	0.178526555	0.123861208	0.181439696	0.072083027	0.200673617	0.160376731	0.132328082	0.272261837	0.15956769	0.246951244
NG8_3	NG	8	0.169678358	0.200061109		0.133584947	0.188463092	0.073052745	0.203472653	0.162496854	0.139915688	0.271328206	0.161161041	0.250003823
NG8_4	NG	8	0.171797462	0.198929232	0.185249879	0.126739445	0.181843034	0.07440788		0.165064419	0.145232282	0.279843715	0.159115882	0.244783525
NG24_1	NG	24	0.159141578	0.209799413	0.177491543	0.122374784	0.174594602	0.068978297	0.201084819	0.181797484	0.13449083		0.158801196	0.240376049
NG24_2	NG	24		0.217550851	0.18163683	0.123767247	0.172705506	0.074960746	0.201042139	0.184881309	0.133434785	0.257908695	0.15804869	0.241865432
NG24_3	NG	24	0.162762339	0.209386436	0.183680202	0.123615218	0.167769673	0.072348418	0.200887339			0.261878573	0.161080312	0.243663801
NG24_4	NG	24	0.166919768	0.213136239	0.19002372	0.126459319	0.169165809	0.071591929	0.198790014	0.175692405	0.144826778	0.262971152		0.239079309
